# Microarray-based method for detection of unknown genetic modifications

**DOI:** 10.1186/1472-6750-7-91

**Published:** 2007-12-18

**Authors:** Torstein Tengs, Anja B Kristoffersen, Knut G Berdal, Tage Thorstensen, Melinka A Butenko, Håvard Nesvold, Arne Holst-Jensen

**Affiliations:** 1 National Veterinary Institute, Section of Feed and Food Microbiology, PO Box 8156 Dep, 0033 Oslo, Norway; 2 National Veterinary Institute, Section of Epidemiology, PO Box 8156 Dep, 0033 Oslo, Norway; 3 University of Oslo, Department of Informatics, PO Box 1080, Blindern, 0316 Oslo, Norway; 4 University of Oslo, Department of Molecular Biosciences, PO Box 1041, Blindern, 0316 Oslo, Norway

## Abstract

**Background:**

Due to the increased use of genetic modifications in crop improvement, there is a need to develop effective methods for the detection of both known and unknown transgene constructs in plants. We have developed a strategy for detection and characterization of unknown genetic modifications and we present a proof of concept for this method using *Arabidopsis thaliana *and *Oryza sativa *(rice). The approach relies on direct hybridization of total genomic DNA to high density microarrays designed to have probes tiled throughout a set of reference sequences.

**Results:**

We show that by using arrays with 25 basepair probes covering both strands of a set of 235 vectors (2 million basepairs) we can detect transgene sequences in transformed lines of *A. thaliana *and rice without prior knowledge about the transformation vectors or the T-DNA constructs used to generate the studied plants.

**Conclusion:**

The approach should allow the user to detect the presence of transgene sequences and get sufficient information for further characterization of unknown genetic constructs in plants. The only requirements are access to a small amount of pure transgene plant material, that the genetic construct in question is above a certain size (here ≥ 140 basepairs) and that parts of the construct shows some degree of sequence similarity with published genetic elements.

## Background

Since the first genetically modified (GM) plants were authorized for release and commercial food production in the mid 1990ies, the global GM acreage has increased rapidly every year. For example 57% of all soybean and 25% of all maize grown on the world market in 2006 was GM [1]. The diversity of food crop species and traits introduced is also rapidly increasing, and with the prospective use of food crop plants in for instance production of pharmaceuticals and biofuels, the risk is also increasing for introduction of unauthorized GM material into the foodchain or release of unauthorized GM plants into the environment. Recent examples of such introduction are the presence of event Bt10 in US maize and LL601 rice in US rice exported to Europe [2,3]. International and national regulations require that distribution and release of GM organisms (GMOs) is under strict control and surveillance, and that GMOs are only authorized on the basis that they do not pose a risk to human or animal health or the environment [4]. With the increased global focus on biosafety, and the realistic scenarios of unintended release of unauthorized and potentially unknown nature of the GM materials, there is a need for availability of technology that rapidly can detect and provide information about a possible unknown GMO.

Genetic modifications of plants (and other organisms) involve the stable introduction of a novel genetic construct (usually carried by a vector) into a target organism's genome. In plants, the most popular traits introduced include herbicide tolerance, insect resistance and ripening delay [5]. In addition to the particular gene associated with the trait of interest, other genetic elements are also transferred to the target plant during a transformation event. For correct expression, promoters such as the cauliflower mosaic virus (CaMV) *35S *promoter (P35S) are needed. Among the most popular terminators/polyadenylation signals are the nopaline synthase (*NOS*) terminator from *Agrobacterium tumefaciens *and the CaMV *35S *polyadenylation signal (T35S). In addition, selection markers, polylinkers and transit peptides are often included in GM constructs. Depending on the transformation technique used, as well as other factors, additional parts of the carrier vector may also be co-transformed into the target genome.

The most widely used methods for detection of genetic modifications in plants rely on real-time PCR. Primers can be designed either to be used as screening tools where consensus primers target a commonly used GM element, gene-specific primers can be used to detect the presence of an introduced trait gene, construct specific assays can be used to specifically target a particular configuration of GM elements, or host/transgene junction-specific primers can be designed to make an assay event-specific [6]. In addition to fluorescent real-time PCR, some methods have also been described where amplified products are labeled and hybridized to arrays [7-10] and multiplex setups have been suggested [11,12]. Still, singleplex PCR remains the most commonly used strategy, and if the genetic modification in question is unknown or poorly characterized, multiple individual PCR experiments are set up. Each PCR assay is designed to target a particular sequence, and multiple assays (and controls) have to be performed in order to check for the presence of different genetic constructs.

We have developed a high resolution microarray-based method for the detection of both known and unknown genetic modifications in plants. The method is PCR-independent, applies direct hybridization of total genomic DNA and takes advantage of the high degree of recycling and sequence similarity between elements commonly used in genetic modifications in plants. Using custom designed microarrays, we have analyzed genetically modified lines of *Arabidopsis thaliana *and *Oryza sativa *(rice) as model systems. We show that without prior knowledge about the transgene sequence in question, fragments (≥ 140 bp) of the elements used in the genetic transformation can be detected.

## Results

NimbleExpress arrays (Affymetrix, Santa Clara, CA, USA) were designed to have 25 basepair probes tiled throughout 235 vector sequences downloaded from GenBank (a total of approximately 2 megabases of sequence; for list of sequences used in design [see Additional file 1]. In order to achieve robust signal intensities from positive probes, we decided to use the highest possible quantity (mass) of DNA given the volume of hybridization mixture that could be injected into the Affymetrix array cartridge. Ninety μg seemed to be the upper limit (more DNA resulted in poor array performance), and this corresponds to roughly 1 fmole haploid *A. thaliana *genomes and 267 amoles of haploid rice genomes.

Six arrays were run, representing two transgene *A. thaliana *lines, two experimental duplicates of wildtype *A. thaliana*, as well as one transgene and one wildtype rice line. Average signal intensities for the array probes were 196.92, 209.71, 203.98, 192.95, 260.43 and 226.06 (*ida*, SALK, *A. thaliana *wildtype replicates, wildtype and CecA rice). After normalization of the arrays, signal/background ratios were calculated for all probes using the average signals from the wildtype replicates as an estimate for the level of probe signal background (Table 1). The complete set of signal data is available upon request.

**Table 1:** Array performance.

	***A. thaliana***			***Oryza sativa***
	**ida/WT**	**SALK/WT**	**WT/WT**	**CecA/WT**

Mean signal ratio	0.96	0.95	0.98	1.09
Signal ratio range	0.049–9.64	0.13–18.45	0.085–3.29	0.24–23.58
Standard deviation (signal ratio)	0.35	0.67	0.16	0.72
Total number of positive probes (percent of total number of probes)	854 (2.29%)	1026 (2.75%)	1120 (3.00%)	845 (2.27%)
Number of probes for target sequence	1252	2056	-	910
True positive probes (target sequence)	605	745	-	322
False positives (percent of total number of probes)	249 (0.69%)	281 (0.80%)	1120 (3.00%)	523 (1.40%)
Number of windows detected	145	394	0	152
Number of windows after filtering	11	15	-	8
Number of probes per window	33–63	32–59	-	32–49
Length of windows	176–325	167–312	-	147–255

**Figure 1:**
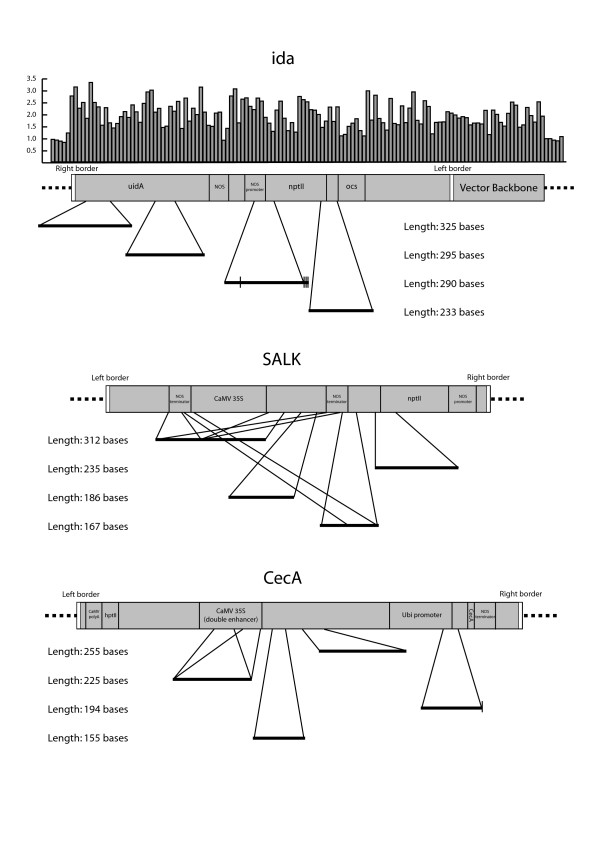
Sequences detected by the arrays. The detected sequence windows were ranked according to the number of probes that defined them and the four highest ranked windows with non-overlapping hits to the T-DNA sequences are shown (for a complete list of positive windows, [see Additional file 2]. The scale for the detected windows is 4× the scale for the T-DNA constructs. Hatch marks correspond to point mutations. The bar diagram showing signal/noise ratio for matching probes (averaged across sets of 10 probes) has been included for the *ida *dataset as an example. The 1,239 bp vector backbone fragment in *ida *has been described previously [28]. NOS – nopaline synthase. oct – octopine synthase. nptII – neomycin phosphotransferase II. CaMV 35S – cauliflower mosaic virus *35S *promoter. uidA – beta glucuronidase. CaMV polyA – cauliflower mosaic virus 3' UTR polyadenylation signal. hptII – hygromycin phosphotransferase. CaMV 35S (double enhancer) – cauliflower mosaic virus *35S *promoter, double enhancer version. Ubi – maize ubiquitin. CecA – cecropin A.

The analysis did not yield any false positives looking at wildtype vs. wildtype *A. thaliana *DNA (Table 1). As expected for the other experiments, a large fraction of the sequence windows detected formed small sets of overlapping and nearly identical sequences. The initial analysis gave 394 positive windows for SALK/WT, 145 for *ida*/WT and 152 for CecA/WT (Table 1). After sequence filtering (see description in Materials and Methods) of data from the *ida *experiment, we were left with 11 unique sequences. In the SALK experiment, 15 sequences were reported and analysis of the rice DNA yielded 8 unique windows. Detected windows ranged in size from 147 to 325 bases, representing 32 to 63 probes (Table 1; for a complete list of detected sequence windows, [see Additional file 2]). All of the high ranking matches could easily be matched back to their corresponding T-DNA loci (Figure 1). For SALK, the highest ranking windows were dominated by elements containing the *NOS *terminator. This element is present twice in the SALK insert and this might lead to a bias due to stronger signals from these probes. This same phenomenon was observed for the double enhancer version of the CaMV *35S *promoter in the CecA rice. All of the sequences detected matched close to 100% perfectly with the target sequences except for some single basepair differences (Figure 1).

Subsequent Southern blotting of DNA from the SALK line using probes targeting the insertion cassette indicated that this was indeed a single copy line, and analysis of sequence data showed that the DNA transferred during the transformation appeared to be limited to the T-DNA region (data not shown). Neither the T-DNA tagging vector pMHA2 [13] used to generate the *ida *plant nor the SALK insertion line T-DNA (from the pBIN-pROK2 vector; [14]) were part of our set of CaMV P35S-containing vectors used for array design. The rice construct contained a large fragment derived from the pCAMBIA-1300 vector, which was included in our target sequence compilation.

## Discussion

The recent increase in feature density and lowered cost associated with the use of microarrays has prompted several groups to develop whole-genome based protocols for characterization of eukaryotic genomic content [15,16]. Most of these groups have relied on currently available (expression) microarrays. Using a single color system, we decided to see if this approach would work using custom designed arrays and unfiltered probes tiled throughout a set of reference sequences in the context of genetically modified plants.

Neither of the transgene plant lines tested were transformed using constructs where complete sequence information had been submitted to GenBank, so in the context of this study they were treated as unknown genetic modifications. But, the method does make some assumptions about the genetic elements that can be uncovered. For example, with the applied minimum window size of 30 probes, the transgene sequence in question must contain at least one element of a significant size (here ≥ 140 bp) that has a reasonably high degree of sequence similarity with one or more of the elements that were included in our target sequence compilation. In this sense, the method is not able to detect all 'truly unknown GMOs'. An array-based method that makes fewer assumptions has been suggested by our group previously [17], but currently no methods (besides perhaps whole genome sequencing) can be said to guarantee the accurate detection of a genetic modification without making any assumptions. Window sizes of 10 and 20 probes were also tested but these yielded too many false positives. The absolute optimal combination of window size and match cutoff (here 70% of the probes in a window) was not determined in the present study.

In our array design, more than 2 million bases of sequence were used to design the overlapping probes. The fact that less than 40,000 probes were sufficient to get a degree of coverage of on average one probe per five bases (both strands) gives an indication of the level of conservation and reuse when it comes to the elements used in designing genetic constructs for plant transformations. Although a novel, unknown GMO might contain a trait gene not included in a reference sequence database, it is highly likely that other elements, such as promoters, selection markers, terminators etc. will be used that have been described in the literature. The entire GenBank vector sequence collection now includes information from about 3,600 entries (> 20 megabases of sequence), but this only constitutes about 5.24 × 10^6 ^unique 25 basepair sequences (both strands of the 3,600 sequences). The highest number of features currently synthesized on commercially available Affymetrix-type arrays is > 6 × 10^6 ^(500 K Mapping Array Set), so it should be possible to make an array with any degree of probe tiling scheme throughout both DNA strands of any set of cloning vectors in GenBank.

Even though our probes were designed using a set of 235 sequences, our window-based approach for detecting GM elements could be used with reference to any set of relevant sequences. We have only screened our results using the same reference sequences we used for the array design, but as long as the probe coverage is reasonably high, data from an array design such as the one described in this study may be compared against a much broader sampling of transgene sequences.

With the current design, it cannot be assumed that very long stretches of target sequence can be detected in their entirety (neither can it be assumed that the exact construct structure one wants to detect is part of the sequence compilation used to match probe signal intensities against). For instance, the vector used in the generation of T-DNA SALK insertion lines was not included in the target sequence compilation used for our array design, but it has a T-DNA sequence with a stretch of 3,475 bases with 100% match to one of the vectors in our target sequence compilation (binary vector pBI121; GenBank accession number AF485783). Still, we were not able to detect a continuous window for this part of the T-DNA construct. In our experiments, 35–48% percent of the probes that should yield a positive signal were scored as true positives (from 322 out of 910 true positive probes for rice to 605 out of 1252 for SALK; Table 1). Poor performance of a fraction of the probes was not unexpected since they were designed without *in silico *filtering [18,19] or empirical testing. It is, however, also important to remember that the cutoff value for scoring a probe as positive using our strategy relies on the standard deviation of the signal/noise ratios. The standard deviation will be affected by the number of potentially positive probes in an experiment. If a large number of probes on the array match the GMO analyzed, the standard deviation is likely to increase. So, the number of probes that are scored as positives is linked to the size of the GM element in question. An *A. thaliana *line transformed with for instance a truncated version of the *ida *construct would probably have led to a smaller standard deviation, and would have given a different set of positive probes and probe windows. So, the sensitivity of the method is likely to increase when the number of potentially positive probes decreases. While at first glance, the results for WT/WT in Table 1 seem to indicate that without true positives in the sample, the method becomes oversensitive (3% false positive probes), the window-based approach readily discriminates between false positive individual probes and false positive windows (no windows were detected for WT/WT).

Thus, the presented method should be seen more as a tool for detecting discrete transgene elements than a protocol for detailed characterization of genetically modified plants. By using sequence similarity search tools such as BLAST [20], it is fairly straightforward to at least partly annotate the positive windows. As a starting point for further characterization of the transgene sequence detected, we recommend using defined genetic loci within the hits rather than the raw sequences. The most commonly used protocols for characterization of GM events rely on anchor-PCR followed DNA sequencing of amplified products [21,22], see also [23] and the sequences detected by our method should be sufficient for initiating a more detailed study using these standard approaches.

The general concept described herein should work well for organisms other than the plant lines described herein, albeit it is likely that a significantly larger genome (human or wheat, for instance) size will decrease the signal/noise ratio using our current protocol for DNA labeling and hybridization. Given the upper limit on the amount of DNA that can be hybridized, a larger genome will give a smaller number of target DNA copies per feature on the array. One way to avoid this problem might be to implement a more sensitive labeling strategy, using for instance quantum dot technology [24]. An alternative approach would be to co-hybridize differentially labeled control and sample DNA and use the signal ratios directly.

## Conclusion

The whole genome based concept described herein should be useful for both detection and characterization of known as well as unknown genetic modifications in plants. The method requires access to a small amount of GMO material of high purity, but the only other limitations are availability of sequence data from GMO constructs and the minimal size of the transgene sequence to be detected. It is expected that the upper limit for number of features that can be fitted onto an array will continue to increase, and combined with decreased array cost, we believe that unbiased array-based detection of GM constructs will be a helpful tool not only for research on plants, but for genetically modified organisms in general.

## Methods

### Array design

For the array design, sequences were chosen that contained (parts of) CaMV P35S based on sequence similarity searches such as BLAST [20]. CaMV P35S is among the most widely used genetic elements in genetic modifications of plants [25-27], and its presence was thus used as a proxy to identify cloning vectors and other constructs likely to be relevant for plant transformations. To achieve maximum probe density using a minimum number of probes, the following probe selection strategy was used: first, all 25 basepair fragments were sequentially extracted from the set of 235 sequences. A corresponding set of reverse-complementary probes was also generated. From these ordered lists, all probes containing ambiguous bases were excluded and duplicate probes removed. Finally, every 10^th ^probe was extracted from the probe database. In the array design, the majority of the probes thus had 15 bases overlaps with neighboring probes on both strands of the target sequences. Due to the high degree of similarity between the elements commonly used in vector sequences, 37,257 probes were sufficient to achieve this coverage.

For general assessment of array performance, positive and negative control probes were also included. The array design included 1,000 positive control probes picked from the Affymetrix *A. thaliana *expression array (GeneChip Arabidopsis ATH1 Genome Array) as well as 1000 positive control probes picked from the GeneChip Rice Genome Array (Affymetrix). The positive control probes were selected so that they corresponded to single copy loci in the respective plant genomes. In addition, 1,000 negative control probes were designed (random 25 bp sequences with no matches in the *A. thaliana *or rice nuclear, chloroplast or mitochondrial genomes).

### DNA extraction and whole genome amplification

Wildtype *A. thaliana *ecotype Columbia (Col-0) plants, the *inflorescence deficient in abscission *(*ida*) mutant [28], and the SALK_128444 line purchased from the Arabidopsis Biological Resource Center (Columbus, OH, USA), were all grown under long day greenhouse conditions at 20°C. DNA was extracted using the method described by Dellaporta *et al.*[29] and resuspended in molecular grade water.

For the rice material, an approach was developed to avoid the requirement of access to the relatively large amounts of genomic DNA described for the *A. thaliana *protocol. DNA was isolated from 30 mg of wildtype Mediterranean elite japonica rice (*Oryza sativa *L.) cultivar Senia and rice transformed with the cecropin A (*CecA*) gene [30] using a CTAB based protocol [31]. The DNA was used to perform whole genome amplification (WGA) using the REPLI-g whole genome amplification kit (QIAGEN AB, Sweden) according to the manufacturer's recommendations. The WGA products were extracted using phenol:chloroform:isoamyl alcohol 25:24:1 (saturated with 10 mM Tris, pH 8.0, 1 mM EDTA; Sigma-Aldrich Norway AS, Oslo, Norway) and ammonium acetate/ethanol precipitated. After resuspension in distilled water, the material was processed the same way as the DNA isolated from *A. thaliana*.

### DNA fragmentation, labeling and hybridization

For each experiment, 90 μg of DNA was fragmented using 4 units (8 units for the WGA DNA) of DNase I (New England Biolabs, Ipswich, MA, USA) in a total volume of 500 μl 1× DNase I Reaction Buffer (New England Biolabs). The fragmentation reaction was incubated for 5 minutes at 37°C, followed by 10 minutes at 95°C to inactivate the enzyme. The fragmented DNA was phenol:chloroform:isoamyl alcohol extracted and ammonium acetate/ethanol precipitated and end labeled using 90 units of terminal deoxynucleotidyl transferase (Promega, Madison, WI, USA) and 5 nmoles of biotin-16-2',3'-dideoxy-uridine-5'-triphosphate (biotin-16-ddUTP; Roche Diagnostics Norge AS, Oslo, Norway). The labeling reaction was performed in 300 μl reaction volume with 1× Terminal Deoxynucleotidyl Transferase Buffer (Roche Diagnostics Norge AS) for two hours at 37°C followed by 10 minutes at 95°C (inactivation of the enzyme). The DNA was again phenol:chloroform:isoamyl alcohol extracted and ammonium acetate/ethanol precipitated.

Several different array hybridization, washing and staining protocols were tested for the arrays. Initially, a hybridization buffer containing tetramethylammonium (TMA) was used. The presence of high concentration of TMA has been shown to reduce bias in hybridization efficiency due to differences in GC content [32], and since the probes on our arrays were not filtered according to base composition (or any other criteria), we believed that such an experimental setup could prove to be the most suitable. However, we concluded that the protocol recommended by Affymetrix for hybridization of PCR amplified chromatin immunoprecipitation products (Affymetrix Chromatin Immunoprecipitation Assay protocol) gave the highest average positive control/negative control probe signal ratio. In order to mimic these hybridization reaction conditions, the pellet was thus resuspended in 4.3 μl 10× Fragmentation Buffer (Affymetrix), 12 μl 5× TdT Buffer (Affymetrix), 3.7 μl Control Oligo B2 (Affymetrix), 15.4 μl DMSO, 110 μl 2× Hybridization Buffer (for recipe, see Affymetrix manual for Affymetrix Chromatin Immunoprecipitation Assay; revision 3, Appendix B) and 74.6 μl molecular grade water (total volume: 220 μl). Arrays were otherwise processed and handled as described in the Affymetrix protocol for Affymetrix Chromatin Immunoprecipitation Assays, but the array cartridge rotation speed during hybridization was reduced from 60 rpm to 20 rpm to facilitate efficient mixing of the viscous hybridization mix (due to the relatively high amount of DNA used).

### Data analysis

The sequence for the SALK T-DNA insert was downloaded from the Salk Institute Genomic Analysis Laboratory web page [33] and the *ida *sequence for the pMHA2 T-DNA was kindly provided by Dr. Abul Mandal (University of Skövde). It is noteworthy that the *ida *construct does not contain the CaMV P35S element that was used as a proxy for the initial stage of the array design (Figure 1). The sequence for the CecA rice line was derived from published information (see [28] for references). Sequences were compared to the T-DNA insert by using the tool 'bl2seq' [34].

For the analysis, raw signal intensities were exported from the GeneChip Operating Software (Version 1.2; Affymetrix) using the software tool IntensityExporter [35]. Average background signal for all vector probes was calculated using two experimental duplicates of wildtype *A. thaliana *DNA. Assuming that the fraction of probes corresponding to true positives in any given experiment will be very low, signal intensities for all arrays were normalized to give the same average vector probe signal (absolute value: 200). The probes and their corresponding signal intensities were mapped back to their positions in the 235 vectors used in the array design. For all probe positions, signal/background ratio was calculated, and these ratios were screened using a window-based approach. A probe was considered positive if the signal/background ratio was higher than the mean ratio plus two standard deviations, and a minimum window size of 30 probes was chosen. This window was slid through all the vector sequences, and was scored as positive if more than 70% (21 or more) of the probes were positive. When a positive window was detected, the window was gradually expanded to include more downstream probes. This expansion was continued until the fraction of positive probes fell below 70%. The sequence windows were subsequently filtered by removing windows completely encompassed by other windows, and sequence fragments were trimmed to remove flanking regions corresponding to negative probes. Remaining sequences were tabulated and ranked according to the number of probes they encompassed.

## Authors' contributions

TTengs conceptualized the project, did all the hybridization experiments and wrote the final version of the paper. ABK performed the data analyses, KGB and AH-J contributed to experimental design and data interpretation. TThorstensen did the Southern blotting and characterization of the SALK *A. thaliana *line and MAB provided data for the *ida *line. HN wrote the software for probe selection. All authors read and approved the final manuscript.

## Supplementary Material

Additional file 1 Vector sequence list. A listing of the 235 vector sequences downloaded from GenBank and used in the design of probes on the array. Click here for file

Additional file 2 Detected sequence windows. A complete description of the detected sequence windows, for all three T-DNA inserts. Click here for file
