# Supplementary material for: Microarray-based method for detection of unknown genetic modifications
Source: BMC Biotechnol. 2007 Dec 18;7:91. doi: 10.1186/1472-6750-7-91 (PMC2225397; doi:10.1186/1472-6750-7-91)
Supplement: Additional file 1 — Vector sequence list. A listing of the 235 vector sequences downloaded from GenBank and used in the design of probes on the array. [file 1472-6750-7-91-S1.doc]

>gi|11559657|gb|AF294976.1| Cloning vector pC1300intA, complete sequence

GAATTCGAGCTCGGTACCCGGGGATCCTCTAGAGTCGACCTGCAGGCATGCAAGCTTGGCACTGGCCGTC

GTTTTACAACGTCGTGACTGGGAAAACCCTGGCGTTACCCAACTTAATCGCCTTGCAGCACATCCCCCTT

TCGCCAGCTGGCGTAATAGCGAAGAGGCCCGCACCGATCGCCCTTCCCAACAGTTGCGCAGCCTGAATGG

CGAATGCTAGAGCAGCTTGAGCTTGGATCAGATTGTCGTTTCCCGCCTTCAGTTTAAACTATCAGTGTTT

GACAGGATATATTGGCGGGTAAACCTAAGAGAAAAGAGCGTTTATTAGAATAACGGATATTTAAAAGGGC

GTGAAAAGGTTTATCCGTTCGTCCATTTGTATGTGCATGCCAACCACAGGGTTCCCCTCGGGATCAAAGT

ACTTTGATCCAACCCCTCCGCTGCTATAGTGCAGTCGGCTTCTGACGTTCAGTGCAGCCGTCTTCTGAAA

ACGACATGTCGCACAAGTCCTAAGTTACGCGACAGGCTGCCGCCCTGCCCTTTTCCTGGCGTTTTCTTGT

CGCGTGTTTTAGTCGCATAAAGTAGAATACTTGCGACTAGAACCGGAGACATTACGCCATGAACAAGAGC

GCCGCCGCTGGCCTGCTGGGCTATGCCCGCGTCAGCACCGACGACCAGGACTTGACCAACCAACGGGCCG

AACTGCACGCGGCCGGCTGCACCAAGCTGTTTTCCGAGAAGATCACCGGCACCAGGCGCGACCGCCCGGA

GCTGGCCAGGATGCTTGACCACCTACGCCCTGGCGACGTTGTGACAGTGACCAGGCTAGACCGCCTGGCC

CGCAGCACCCGCGACCTACTGGACATTGCCGAGCGCATCCAGGAGGCCGGCGCGGGCCTGCGTAGCCTGG

CAGAGCCGTGGGCCGACACCACCACGCCGGCCGGCCGCATGGTGTTGACCGTGTTCGCCGGCATTGCCGA

GTTCGAGCGTTCCCTAATCATCGACCGCACCCGGAGCGGGCGCGAGGCCGCCAAGGCCCGAGGCGTGAAG

TTTGGCCCCCGCCCTACCCTCACCCCGGCACAGATCGCGCACGCCCGCGAGCTGATCGACCAGGAAGGCC

GCACCGTGAAAGAGGCGGCTGCACTGCTTGGCGTGCATCGCTCGACCCTGTACCGCGCACTTGAGCGCAG

CGAGGAAGTGACGCCCACCGAGGCCAGGCGGCGCGGTGCCTTCCGTGAGGACGCATTGACCGAGGCCGAC

GCCCTGGCGGCCGCCGAGAATGAACGCCAAGAGGAACAAGCATGAAACCGCACCAGGACGGCCAGGACGA

ACCGTTTTTCATTACCGAAGAGATCGAGGCGGAGATGATCGCGGCCGGGTACGTGTTCGAGCCGCCCGCG

CACGTCTCAACCGTGCGGCTGCATGAAATCCTGGCCGGTTTGTCTGATGCCAAGCTGGCGGCCTGGCCGG

CCAGCTTGGCCGCTGAAGAAACCGAGCGCCGCCGTCTAAAAAGGTGATGTGTATTTGAGTAAAACAGCTT

GCGTCATGCGGTCGCTGCGTATATGATGCGATGAGTAAATAAACAAATACGCAAGGGGAACGCATGAAGG

TTATCGCTGTACTTAACCAGAAAGGCGGGTCAGGCAAGACGACCATCGCAACCCATCTAGCCCGCGCCCT

GCAACTCGCCGGGGCCGATGTTCTGTTAGTCGATTCCGATCCCCAGGGCAGTGCCCGCGATTGGGCGGCC

GTGCGGGAAGATCAACCGCTAACCGTTGTCGGCATCGACCGCCCGACGATTGACCGCGACGTGAAGGCCA

TCGGCCGGCGCGACTTCGTAGTGATCGACGGAGCGCCCCAGGCGGCGGACTTGGCTGTGTCCGCGATCAA

GGCAGCCGACTTCGTGCTGATTCCGGTGCAGCCAAGCCCTTACGACATATGGGCCACCGCCGACCTGGTG

GAGCTGGTTAAGCAGCGCATTGAGGTCACGGATGGAAGGCTACAAGCGGCCTTTGTCGTGTCGCGGGCGA

TCAAAGGCACGCGCATCGGCGGTGAGGTTGCCGAGGCGCTGGCCGGGTACGAGCTGCCCATTCTTGAGTC

CCGTATCACGCAGCGCGTGAGCTACCCAGGCACTGCCGCCGCCGGCACAACCGTTCTTGAATCAGAACCC

GAGGGCGACGCTGCCCGCGAGGTCCAGGCGCTGGCCGCTGAAATTAAATCAAAACTCATTTGAGTTAATG

AGGTAAAGAGAAAATGAGCAAAAGCACAAACACGCTAAGTGCCGGCCGTCCGAGCGCACGCAGCAGCAAG

GCTGCAACGTTGGCCAGCCTGGCAGACACGCCAGCCATGAAGCGGGTCAACTTTCAGTTGCCGGCGGAGG

ATCACACCAAGCTGAAGATGTACGCGGTACGCCAAGGCAAGACCATTACCGAGCTGCTATCTGAATACAT

CGCGCAGCTACCAGAGTAAATGAGCAAATGAATAAATGAGTAGATGAATTTTAGCGGCTAAAGGAGGCGG

CATGGAAAATCAAGAACAACCAGGCACCGACGCCGTGGAATGCCCCATGTGTGGAGGAACGGGCGGTTGG

CCAGGCGTAAGCGGCTGGGTTGTCTGCCGGCCCTGCAATGGCACTGGAACCCCCAAGCCCGAGGAATCGG

CGTGACGGTCGCAAACCATCCGGCCCGGTACAAATCGGCGCGGCGCTGGGTGATGACCTGGTGGAGAAGT

TGAAGGCCGCGCAGGCCGCCCAGCGGCAACGCATCGAGGCAGAAGCACGCCCCGGTGAATCGTGGCAAGC

GGCCGCTGATCGAATCCGCAAAGAATCCCGGCAACCGCCGGCAGCCGGTGCGCCGTCGATTAGGAAGCCG

CCCAAGGGCGACGAGCAACCAGATTTTTTCGTTCCGATGCTCTATGACGTGGGCACCCGCGATAGTCGCA

GCATCATGGACGTGGCCGTTTTCCGTCTGTCGAAGCGTGACCGACGAGCTGGCGAGGTGATCCGCTACGA

GCTTCCAGACGGGCACGTAGAGGTTTCCGCAGGGCCGGCCGGCATGGCCAGTGTGTGGGATTACGACCTG

GTACTGATGGCGGTTTCCCATCTAACCGAATCCATGAACCGATACCGGGAAGGGAAGGGAGACAAGCCCG

GCCGCGTGTTCCGTCCACACGTTGCGGACGTACTCAAGTTCTGCCGGCGAGCCGATGGCGGAAAGCAGAA

AGACGACCTGGTAGAAACCTGCATTCGGTTAAACACCACGCACGTTGCCATGCAGCGTACGAAGAAGGCC

AAGAACGGCCGCCTGGTGACGGTATCCGAGGGTGAAGCCTTGATTAGCCGCTACAAGATCGTAAAGAGCG

AAACCGGGCGGCCGGAGTACATCGAGATCGAGCTAGCTGATTGGATGTACCGCGAGATCACAGAAGGCAA

GAACCCGGACGTGCTGACGGTTCACCCCGATTACTTTTTGATCGATCCCGGCATCGGCCGTTTTCTCTAC

CGCCTGGCACGCCGCGCCGCAGGCAAGGCAGAAGCCAGATGGTTGTTCAAGACGATCTACGAACGCAGTG

GCAGCGCCGGAGAGTTCAAGAAGTTCTGTTTCACCGTGCGCAAGCTGATCGGGTCAAATGACCTGCCGGA

GTACGATTTGAAGGAGGAGGCGGGGCAGGCTGGCCCGATCCTAGTCATGCGCTACCGCAACCTGATCGAG

GGCGAAGCATCCGCCGGTTCCTAATGTACGGAGCAGATGCTAGGGCAAATTGCCCTAGCAGGGGAAAAAG

GTCGAAAAGGTCTCTTTCCTGTGGATAGCACGTACATTGGGAACCCAAAGCCGTACATTGGGAACCGGAA

CCCGTACATTGGGAACCCAAAGCCGTACATTGGGAACCGGTCACACATGTAAGTGACTGATATAAAAGAG

AAAAAAGGCGATTTTTCCGCCTAAAACTCTTTAAAACTTATTAAAACTCTTAAAACCCGCCTGGCCTGTG

CATAACTGTCTGGCCAGCGCACAGCCGAAGAGCTGCAAAAAGCGCCTACCCTTCGGTCGCTGCGCTCCCT

ACGCCCCGCCGCTTCGCGTCGGCCTATCGCGGCCGCTGGCCGCTCAAAAATGGCTGGCCTACGGCCAGGC

AATCTACCAGGGCGCGGACAAGCCGCGCCGTCGCCACTCGACCGCCGGCGCCCACATCAAGGCACCCTGC

CTCGCGCGTTTCGGTGATGACGGTGAAAACCTCTGACACATGCAGCTCCCGGAGACGGTCACAGCTTGTC

TGTAAGCGGATGCCGGGAGCAGACAAGCCCGTCAGGGCGCGTCAGCGGGTGTTGGCGGGTGTCGGGGCGC

AGCCATGACCCAGTCACGTAGCGATAGCGGAGTGTATACTGGCTTAACTATGCGGCATCAGAGCAGATTG

TACTGAGAGTGCACCATATGCGGTGTGAAATACCGCACAGATGCGTAAGGAGAAAATACCGCATCAGGCG

CTCTTCCGCTTCCTCGCTCACTGACTCGCTGCGCTCGGTCGTTCGGCTGCGGCGAGCGGTATCAGCTCAC

TCAAAGGCGGTAATACGGTTATCCACAGAATCAGGGGATAACGCAGGAAAGAACATGTGAGCAAAAGGCC

AGCAAAAGGCCAGGAACCGTAAAAAGGCCGCGTTGCTGGCGTTTTTCCATAGGCTCCGCCCCCCTGACGA

GCATCACAAAAATCGACGCTCAAGTCAGAGGTGGCGAAACCCGACAGGACTATAAAGATACCAGGCGTTT

CCCCCTGGAAGCTCCCTCGTGCGCTCTCCTGTTCCGACCCTGCCGCTTACCGGATACCTGTCCGCCTTTC

TCCCTTCGGGAAGCGTGGCGCTTTCTCATAGCTCACGCTGTAGGTATCTCAGTTCGGTGTAGGTCGTTCG

CTCCAAGCTGGGCTGTGTGCACGAACCCCCCGTTCAGCCCGACCGCTGCGCCTTATCCGGTAACTATCGT

CTTGAGTCCAACCCGGTAAGACACGACTTATCGCCACTGGCAGCAGCCACTGGTAACAGGATTAGCAGAG

CGAGGTATGTAGGCGGTGCTACAGAGTTCTTGAAGTGGTGGCCTAACTACGGCTACACTAGAAGGACAGT

ATTTGGTATCTGCGCTCTGCTGAAGCCAGTTACCTTCGGAAAAAGAGTTGGTAGCTCTTGATCCGGCAAA

CAAACCACCGCTGGTAGCGGTGGTTTTTTTGTTTGCAAGCAGCAGATTACGCGCAGAAAAAAAGGATCTC

AAGAAGATCCTTTGATCTTTTCTACGGGGTCTGACGCTCAGTGGAACGAAAACTCACGTTAAGGGATTTT

GGTCATGCATTCTAGGTACTAAAACAATTCATCCAGTAAAATATAATATTTTATTTTCTCCCAATCAGGC

TTGATCCCCAGTAAGTCAAAAAATAGCTCGACATACTGTTCTTCCCCGATATCCTCCCTGATCGACCGGA

CGCAGAAGGCAATGTCATACCACTTGTCCGCCCTGCCGCTTCTCCCAAGATCAATAAAGCCACTTACTTT

GCCATCTTTCACAAAGATGTTGCTGTCTCCCAGGTCGCCGTGGGAAAAGACAAGTTCCTCTTCGGGCTTT

TCCGTCTTTAAAAAATCATACAGCTCGCGCGGATCTTTAAATGGAGTGTCTTCTTCCCAGTTTTCGCAAT

CCACATCGGCCAGATCGTTATTCAGTAAGTAATCCAATTCGGCTAAGCGGCTGTCTAAGCTATTCGTATA

GGGACAATCCGATATGTCGATGGAGTGAAAGAGCCTGATGCACTCCGCATACAGCTCGATAATCTTTTCA

GGGCTTTGTTCATCTTCATACTCTTCCGAGCAAAGGACGCCATCGGCCTCACTCATGAGCAGATTGCTCC

AGCCATCATGCCGTTCAAAGTGCAGGACCTTTGGAACAGGCAGCTTTCCTTCCAGCCATAGCATCATGTC

CTTTTCCCGTTCCACATCATAGGTGGTCCCTTTATACCGGCTGTCCGTCATTTTTAAATATAGGTTTTCA

TTTTCTCCCACCAGCTTATATACCTTAGCAGGAGACATTCCTTCCGTATCTTTTACGCAGCGGTATTTTT

CGATCAGTTTTTTCAATTCCGGTGATATTCTCATTTTAGCCATTTATTATTTCCTTCCTCTTTTCTACAG

TATTTAAAGATACCCCAAGAAGCTAATTATAACAAGACGAACTCCAATTCACTGTTCCTTGCATTCTAAA

ACCTTAAATACCAGAAAACAGCTTTTTCAAAGTTGTTTTCAAAGTTGGCGTATAACATAGTATCGACGGA

GCCGATTTTGAAACCGCGGTGATCACAGGCAGCAACGCTCTGTCATCGTTACAATCAACATGCTACCCTC

CGCGAGATCATCCGTGTTTCAAACCCGGCAGCTTAGTTGCCGTTCTTCCGAATAGCATCGGTAACATGAG

CAAAGTCTGCCGCCTTACAACGGCTCTCCCGCTGACGCCGTCCCGGACTGATGGGCTGCCTGTATCGAGT

GGTGATTTTGTGCCGAGCTGCCGGTCGGGGAGCTGTTGGCTGGCTGGTGGCAGGATATATTGTGGTGTAA

ACAAATTGACGCTTAGACAACTTAATAACACATTGCGGACGTTTTTAATGTACTGAATTAACGCCGAATT

AATTCGGGGGATCTGGATTTTAGTACTGGATTTTGGTTTTAGGAATTAGAAATTTTATTGATAGAAGTAT

TTTACAAATACAAATACATACTAAGGGTTTCTTATATGCTCAACACATGAGCGAAACCCTATAGGAACCC

TAATTCCCTTATCTGGGAACTACTCACACATTATTATGGAGAAACTCGAGCTTGTCGATCGACAGATCCG

GTCGGCATCTACTCTATTTCTTTGCCCTCGGACGAGTGCTGGGGCGTCGGTTTCCACTATCGGCGAGTAC

TTCTACACAGCCATCGGTCCAGACGGCCGCGCTTCTGCGGGCGATTTGTGTACGCCCGACAGTCCCGGCT

CCGGATCGGACGATTGCGTCGCATCGACCCTGCGCCCAAGCTGCATCATCGAAATTGCCGTCAACCAAGC

TCTGATAGAGTTGGTCAAGACCAATGCGGAGCATATACGCCCGGAGTCGTGGCGATCCTGCAAGCTCCGG

ATGCCTCCGCTCGAAGTAGCGCGTCTGCTGCTCCATACAAGCCAACCACGGCCTCCAGAAGAAGATGTTG

GCGACCTCGTATTGGGAATCCCCGAACATCGCCTCGCTCCAGTCAATGACCGCTGTTATGCGGCCATTGT

CCGTCAGGACATTGTTGGAGCCGAAATCCGCGTGCACGAGGTGCCGGACTTCGGGGCAGTCCTCGGCCCA

AAGCATCAGCTCATCGAGAGCCTGCGCGACGGACGCACTGACGGTGTCGTCCATCACAGTTTGCCAGTGA

TACACATGGGGATCAGCAATCGCGCATATGAAATCACGCCATGTAGTGTATTGACCGATTCCTTGCGGTC

CGAATGGGCCGAACCCGCTCGTCTGGCTAAGATCGGCCGCAGCGATCGCATCCATGGCCTCCGCGACCGG

CTGCAGTTATCATCATCATCATAGACACACGAAATAAAGTAATCAGATTATCAGTTAAAGCTATGTAATA

TTTACACCATAACCAATCAATTAAAAAATAGATCAGTTTAAAGAAAGATCAAAGCTCAAAAAAATAAAAA

GAGAAAAGGGTCCTAACCAAGAAAATGAAGGAGAAAAACTAGAAATTTACCTGCAGAACAGCGGGCAGTT

CGGTTTCAGGCAGGTCTTGCAACGTGACACCCTGTGCACGGCGGGAGATGCAATAGGTCAGGCTCTCGCT

AAACTCCCCAATGTCAAGCACTTCCGGAATCGGGAGCGCGGCCGATGCAAAGTGCCGATAAACATAACGA

TCTTTGTAGAAACCATCGGCGCAGCTATTTACCCGCAGGACATATCCACGCCCTCCTACATCGAAGCTGA

AAGCACGAGATTCTTCGCCCTCCGAGAGCTGCATCAGGTCGGAGACGCTGTCGAACTTTTCGATCAGAAA

CTTCTCGACAGACGTCGCGGTGAGTTCAGGCTTTTTCATATCTCATTGCCCCCCCGGATCTGCGAAAGCT

CGAGAGAGATAGATTTGTAGAGAGAGACTGGTGATTTCAGCGTGTCCTCTCCAAATGAAATGAACTTCCT

TATATAGAGGAAGGTCTTGCGAAGGATAGTGGGATTGTGCGTCATCCCTTACGTCAGTGGAGATATCACA

TCAATCCACTTGCTTTGAAGACGTGGTTGGAACGTCTTCTTTTTCCACGATGCTCCTCGTGGGTGGGGGT

CCATCTTTGGGACCACTGTCGGCAGAGGCATCTTGAACGATAGCCTTTCCTTTATCGCAATGATGGCATT

TGTAGGTGCCACCTTCCTTTTCTACTGTCCTTTTGATGAAGTGACAGATAGCTGGGCAATGGAATCCGAG

GAGGTTTCCCGATATTACCCTTTGTTGAAAAGTCTCAATAGCCCTTTGGTCTTCTGAGACTGTATCTTTG

ATATTCTTGGAGTAGACGAGAGTGTCGTGCTCCACCATGTTATCACATCAATCCACTTGCTTTGAAGACG

TGGTTGGAACGTCTTCTTTTTCCACGATGCTCCTCGTGGGTGGGGGTCCATCTTTGGGACCACTGTCGGC

AGAGGCATCTTGAACGATAGCCTTTCCTTTATCGCAATGATGGCATTTGTAGGTGCCACCTTCCTTTTCT

ACTGTCCTTTTGATGAAGTGACAGATAGCTGGGCAATGGAATCCGAGGAGGTTTCCCGATATTACCCTTT

GTTGAAAAGTCTCAATAGCCCTTTGGTCTTCTGAGACTGTATCTTTGATATTCTTGGAGTAGACGAGAGT

GTCGTGCTCCACCATGTTGGCAAGCTGCTCTAGCCAATACGCAAACCGCCTCTCCCCGCGCGTTGGCCGA

TTCATTAATGCAGCTGGCACGACAGGTTTCCCGACTGGAAAGCGGGCAGTGAGCGCAACGCAATTAATGT

GAGTTAGCTCACTCATTAGGCACCCCAGGCTTTACACTTTATGCTTCCGGCTCGTATGTTGTGTGGAATT

GTGAGCGGATAACAATTTCACACAGGAAACAGCTATGACCATGATTAC

>gi|11559659|gb|AF294977.1| Cloning vector pC1300intB, complete sequence

GAATTCGAGCTCGGTACCCGGGGATCCTCTAGAGTCGACCTGCAGGCATGCAAGCTTGGCACTGGCCGTC

GTTTTACAACGTCGTGACTGGGAAAACCCTGGCGTTACCCAACTTAATCGCCTTGCAGCACATCCCCCTT

TCGCCAGCTGGCGTAATAGCGAAGAGGCCCGCACCGATCGCCCTTCCCAACAGTTGCGCAGCCTGAATGG

CGAATGCTAGAGCAGCTTGAGCTTGGATCAGATTGTCGTTTCCCGCCTTCAGTTTAAACTATCAGTGTTT

GACAGGATATATTGGCGGGTAAACCTAAGAGAAAAGAGCGTTTATTAGAATAACGGATATTTAAAAGGGC

GTGAAAAGGTTTATCCGTTCGTCCATTTGTATGTGCATGCCAACCACAGGGTTCCCCTCGGGATCAAAGT

ACTTTGATCCAACCCCTCCGCTGCTATAGTGCAGTCGGCTTCTGACGTTCAGTGCAGCCGTCTTCTGAAA

ACGACATGTCGCACAAGTCCTAAGTTACGCGACAGGCTGCCGCCCTGCCCTTTTCCTGGCGTTTTCTTGT

CGCGTGTTTTAGTCGCATAAAGTAGAATACTTGCGACTAGAACCGGAGACATTACGCCATGAACAAGAGC

GCCGCCGCTGGCCTGCTGGGCTATGCCCGCGTCAGCACCGACGACCAGGACTTGACCAACCAACGGGCCG

AACTGCACGCGGCCGGCTGCACCAAGCTGTTTTCCGAGAAGATCACCGGCACCAGGCGCGACCGCCCGGA

GCTGGCCAGGATGCTTGACCACCTACGCCCTGGCGACGTTGTGACAGTGACCAGGCTAGACCGCCTGGCC

CGCAGCACCCGCGACCTACTGGACATTGCCGAGCGCATCCAGGAGGCCGGCGCGGGCCTGCGTAGCCTGG

CAGAGCCGTGGGCCGACACCACCACGCCGGCCGGCCGCATGGTGTTGACCGTGTTCGCCGGCATTGCCGA

GTTCGAGCGTTCCCTAATCATCGACCGCACCCGGAGCGGGCGCGAGGCCGCCAAGGCCCGAGGCGTGAAG

TTTGGCCCCCGCCCTACCCTCACCCCGGCACAGATCGCGCACGCCCGCGAGCTGATCGACCAGGAAGGCC

GCACCGTGAAAGAGGCGGCTGCACTGCTTGGCGTGCATCGCTCGACCCTGTACCGCGCACTTGAGCGCAG

CGAGGAAGTGACGCCCACCGAGGCCAGGCGGCGCGGTGCCTTCCGTGAGGACGCATTGACCGAGGCCGAC

GCCCTGGCGGCCGCCGAGAATGAACGCCAAGAGGAACAAGCATGAAACCGCACCAGGACGGCCAGGACGA

ACCGTTTTTCATTACCGAAGAGATCGAGGCGGAGATGATCGCGGCCGGGTACGTGTTCGAGCCGCCCGCG

CACGTCTCAACCGTGCGGCTGCATGAAATCCTGGCCGGTTTGTCTGATGCCAAGCTGGCGGCCTGGCCGG

CCAGCTTGGCCGCTGAAGAAACCGAGCGCCGCCGTCTAAAAAGGTGATGTGTATTTGAGTAAAACAGCTT

GCGTCATGCGGTCGCTGCGTATATGATGCGATGAGTAAATAAACAAATACGCAAGGGGAACGCATGAAGG

TTATCGCTGTACTTAACCAGAAAGGCGGGTCAGGCAAGACGACCATCGCAACCCATCTAGCCCGCGCCCT

GCAACTCGCCGGGGCCGATGTTCTGTTAGTCGATTCCGATCCCCAGGGCAGTGCCCGCGATTGGGCGGCC

GTGCGGGAAGATCAACCGCTAACCGTTGTCGGCATCGACCGCCCGACGATTGACCGCGACGTGAAGGCCA

TCGGCCGGCGCGACTTCGTAGTGATCGACGGAGCGCCCCAGGCGGCGGACTTGGCTGTGTCCGCGATCAA

GGCAGCCGACTTCGTGCTGATTCCGGTGCAGCCAAGCCCTTACGACATATGGGCCACCGCCGACCTGGTG

GAGCTGGTTAAGCAGCGCATTGAGGTCACGGATGGAAGGCTACAAGCGGCCTTTGTCGTGTCGCGGGCGA

TCAAAGGCACGCGCATCGGCGGTGAGGTTGCCGAGGCGCTGGCCGGGTACGAGCTGCCCATTCTTGAGTC

CCGTATCACGCAGCGCGTGAGCTACCCAGGCACTGCCGCCGCCGGCACAACCGTTCTTGAATCAGAACCC

GAGGGCGACGCTGCCCGCGAGGTCCAGGCGCTGGCCGCTGAAATTAAATCAAAACTCATTTGAGTTAATG

AGGTAAAGAGAAAATGAGCAAAAGCACAAACACGCTAAGTGCCGGCCGTCCGAGCGCACGCAGCAGCAAG

GCTGCAACGTTGGCCAGCCTGGCAGACACGCCAGCCATGAAGCGGGTCAACTTTCAGTTGCCGGCGGAGG

ATCACACCAAGCTGAAGATGTACGCGGTACGCCAAGGCAAGACCATTACCGAGCTGCTATCTGAATACAT

CGCGCAGCTACCAGAGTAAATGAGCAAATGAATAAATGAGTAGATGAATTTTAGCGGCTAAAGGAGGCGG

CATGGAAAATCAAGAACAACCAGGCACCGACGCCGTGGAATGCCCCATGTGTGGAGGAACGGGCGGTTGG

CCAGGCGTAAGCGGCTGGGTTGTCTGCCGGCCCTGCAATGGCACTGGAACCCCCAAGCCCGAGGAATCGG

CGTGACGGTCGCAAACCATCCGGCCCGGTACAAATCGGCGCGGCGCTGGGTGATGACCTGGTGGAGAAGT

TGAAGGCCGCGCAGGCCGCCCAGCGGCAACGCATCGAGGCAGAAGCACGCCCCGGTGAATCGTGGCAAGC

GGCCGCTGATCGAATCCGCAAAGAATCCCGGCAACCGCCGGCAGCCGGTGCGCCGTCGATTAGGAAGCCG

CCCAAGGGCGACGAGCAACCAGATTTTTTCGTTCCGATGCTCTATGACGTGGGCACCCGCGATAGTCGCA

GCATCATGGACGTGGCCGTTTTCCGTCTGTCGAAGCGTGACCGACGAGCTGGCGAGGTGATCCGCTACGA

GCTTCCAGACGGGCACGTAGAGGTTTCCGCAGGGCCGGCCGGCATGGCCAGTGTGTGGGATTACGACCTG

GTACTGATGGCGGTTTCCCATCTAACCGAATCCATGAACCGATACCGGGAAGGGAAGGGAGACAAGCCCG

GCCGCGTGTTCCGTCCACACGTTGCGGACGTACTCAAGTTCTGCCGGCGAGCCGATGGCGGAAAGCAGAA

AGACGACCTGGTAGAAACCTGCATTCGGTTAAACACCACGCACGTTGCCATGCAGCGTACGAAGAAGGCC

AAGAACGGCCGCCTGGTGACGGTATCCGAGGGTGAAGCCTTGATTAGCCGCTACAAGATCGTAAAGAGCG

AAACCGGGCGGCCGGAGTACATCGAGATCGAGCTAGCTGATTGGATGTACCGCGAGATCACAGAAGGCAA

GAACCCGGACGTGCTGACGGTTCACCCCGATTACTTTTTGATCGATCCCGGCATCGGCCGTTTTCTCTAC

CGCCTGGCACGCCGCGCCGCAGGCAAGGCAGAAGCCAGATGGTTGTTCAAGACGATCTACGAACGCAGTG

GCAGCGCCGGAGAGTTCAAGAAGTTCTGTTTCACCGTGCGCAAGCTGATCGGGTCAAATGACCTGCCGGA

GTACGATTTGAAGGAGGAGGCGGGGCAGGCTGGCCCGATCCTAGTCATGCGCTACCGCAACCTGATCGAG

GGCGAAGCATCCGCCGGTTCCTAATGTACGGAGCAGATGCTAGGGCAAATTGCCCTAGCAGGGGAAAAAG

GTCGAAAAGGTCTCTTTCCTGTGGATAGCACGTACATTGGGAACCCAAAGCCGTACATTGGGAACCGGAA

CCCGTACATTGGGAACCCAAAGCCGTACATTGGGAACCGGTCACACATGTAAGTGACTGATATAAAAGAG

AAAAAAGGCGATTTTTCCGCCTAAAACTCTTTAAAACTTATTAAAACTCTTAAAACCCGCCTGGCCTGTG

CATAACTGTCTGGCCAGCGCACAGCCGAAGAGCTGCAAAAAGCGCCTACCCTTCGGTCGCTGCGCTCCCT

ACGCCCCGCCGCTTCGCGTCGGCCTATCGCGGCCGCTGGCCGCTCAAAAATGGCTGGCCTACGGCCAGGC

AATCTACCAGGGCGCGGACAAGCCGCGCCGTCGCCACTCGACCGCCGGCGCCCACATCAAGGCACCCTGC

CTCGCGCGTTTCGGTGATGACGGTGAAAACCTCTGACACATGCAGCTCCCGGAGACGGTCACAGCTTGTC

TGTAAGCGGATGCCGGGAGCAGACAAGCCCGTCAGGGCGCGTCAGCGGGTGTTGGCGGGTGTCGGGGCGC

AGCCATGACCCAGTCACGTAGCGATAGCGGAGTGTATACTGGCTTAACTATGCGGCATCAGAGCAGATTG

TACTGAGAGTGCACCATATGCGGTGTGAAATACCGCACAGATGCGTAAGGAGAAAATACCGCATCAGGCG

CTCTTCCGCTTCCTCGCTCACTGACTCGCTGCGCTCGGTCGTTCGGCTGCGGCGAGCGGTATCAGCTCAC

TCAAAGGCGGTAATACGGTTATCCACAGAATCAGGGGATAACGCAGGAAAGAACATGTGAGCAAAAGGCC

AGCAAAAGGCCAGGAACCGTAAAAAGGCCGCGTTGCTGGCGTTTTTCCATAGGCTCCGCCCCCCTGACGA

GCATCACAAAAATCGACGCTCAAGTCAGAGGTGGCGAAACCCGACAGGACTATAAAGATACCAGGCGTTT

CCCCCTGGAAGCTCCCTCGTGCGCTCTCCTGTTCCGACCCTGCCGCTTACCGGATACCTGTCCGCCTTTC

TCCCTTCGGGAAGCGTGGCGCTTTCTCATAGCTCACGCTGTAGGTATCTCAGTTCGGTGTAGGTCGTTCG

CTCCAAGCTGGGCTGTGTGCACGAACCCCCCGTTCAGCCCGACCGCTGCGCCTTATCCGGTAACTATCGT

CTTGAGTCCAACCCGGTAAGACACGACTTATCGCCACTGGCAGCAGCCACTGGTAACAGGATTAGCAGAG

CGAGGTATGTAGGCGGTGCTACAGAGTTCTTGAAGTGGTGGCCTAACTACGGCTACACTAGAAGGACAGT

ATTTGGTATCTGCGCTCTGCTGAAGCCAGTTACCTTCGGAAAAAGAGTTGGTAGCTCTTGATCCGGCAAA

CAAACCACCGCTGGTAGCGGTGGTTTTTTTGTTTGCAAGCAGCAGATTACGCGCAGAAAAAAAGGATCTC

AAGAAGATCCTTTGATCTTTTCTACGGGGTCTGACGCTCAGTGGAACGAAAACTCACGTTAAGGGATTTT

GGTCATGCATTCTAGGTACTAAAACAATTCATCCAGTAAAATATAATATTTTATTTTCTCCCAATCAGGC

TTGATCCCCAGTAAGTCAAAAAATAGCTCGACATACTGTTCTTCCCCGATATCCTCCCTGATCGACCGGA

CGCAGAAGGCAATGTCATACCACTTGTCCGCCCTGCCGCTTCTCCCAAGATCAATAAAGCCACTTACTTT

GCCATCTTTCACAAAGATGTTGCTGTCTCCCAGGTCGCCGTGGGAAAAGACAAGTTCCTCTTCGGGCTTT

TCCGTCTTTAAAAAATCATACAGCTCGCGCGGATCTTTAAATGGAGTGTCTTCTTCCCAGTTTTCGCAAT

CCACATCGGCCAGATCGTTATTCAGTAAGTAATCCAATTCGGCTAAGCGGCTGTCTAAGCTATTCGTATA

GGGACAATCCGATATGTCGATGGAGTGAAAGAGCCTGATGCACTCCGCATACAGCTCGATAATCTTTTCA

GGGCTTTGTTCATCTTCATACTCTTCCGAGCAAAGGACGCCATCGGCCTCACTCATGAGCAGATTGCTCC

AGCCATCATGCCGTTCAAAGTGCAGGACCTTTGGAACAGGCAGCTTTCCTTCCAGCCATAGCATCATGTC

CTTTTCCCGTTCCACATCATAGGTGGTCCCTTTATACCGGCTGTCCGTCATTTTTAAATATAGGTTTTCA

TTTTCTCCCACCAGCTTATATACCTTAGCAGGAGACATTCCTTCCGTATCTTTTACGCAGCGGTATTTTT

CGATCAGTTTTTTCAATTCCGGTGATATTCTCATTTTAGCCATTTATTATTTCCTTCCTCTTTTCTACAG

TATTTAAAGATACCCCAAGAAGCTAATTATAACAAGACGAACTCCAATTCACTGTTCCTTGCATTCTAAA

ACCTTAAATACCAGAAAACAGCTTTTTCAAAGTTGTTTTCAAAGTTGGCGTATAACATAGTATCGACGGA

GCCGATTTTGAAACCGCGGTGATCACAGGCAGCAACGCTCTGTCATCGTTACAATCAACATGCTACCCTC

CGCGAGATCATCCGTGTTTCAAACCCGGCAGCTTAGTTGCCGTTCTTCCGAATAGCATCGGTAACATGAG

CAAAGTCTGCCGCCTTACAACGGCTCTCCCGCTGACGCCGTCCCGGACTGATGGGCTGCCTGTATCGAGT

GGTGATTTTGTGCCGAGCTGCCGGTCGGGGAGCTGTTGGCTGGCTGGTGGCAGGATATATTGTGGTGTAA

ACAAATTGACGCTTAGACAACTTAATAACACATTGCGGACGTTTTTAATGTACTGAATTAACGCCGAATT

AATTCGGGGGATCTGGATTTTAGTACTGGATTTTGGTTTTAGGAATTAGAAATTTTATTGATAGAAGTAT

TTTACAAATACAAATACATACTAAGGGTTTCTTATATGCTCAACACATGAGCGAAACCCTATAGGAACCC

TAATTCCCTTATCTGGGAACTACTCACACATTATTATGGAGAAACTCGAAGATCCGTCGAGCTTGTCGAT

CGACAGATCCGGTCGGCATCTACTCTATTTCTTTGCCCTCGGACGAGTGCTGGGGCGTCGGTTTCCACTA

TCGGCGAGTACTTCTACACAGCCATCGGTCCAGACGGCCGCGCTTCTGCGGGCGATTTGTGTACGCCCGA

CAGTCCCGGCTCCGGATCGGACGATTGCGTCGCATCGACCCTGCGCCCAAGCTGCATCATCGAAATTGCC

GTCAACCAAGCTCTGATAGAGTTGGTCAAGACCAATGCGGAGCATATACGCCCGGAGTCGTGGCGATCCT

GCAAGCTCCGGATGCCTCCGCTCGAAGTAGCGCGTCTGCTGCTCCATACAAGCCAACCACGGCCTCCAGA

AGAAGATGTTGGCGACCTCGTATTGGGAATCCCCGAACATCGCCTCGCTCCAGTCAATGACCGCTGTTAT

GCGGCCATTGTCCGTCAGGACATTGTTGGAGCCGAAATCCGCGTGCACGAGGTGCCGGACTTCGGGGCAG

TCCTCGGCCCAAAGCATCAGCTCATCGAGAGCCTGCGCGACGGACGCACTGACGGTGTCGTCCATCACAG

TTTGCCAGTGATACACATGGGGATCAGCAATCGCGCATATGAAATCACGCCATGTAGTGTATTGACCGAT

TCCTTGCGGTCCGAATGGGCCGAACCCGCTCGTCTGGCTAAGATCGGCCGCAGCGATCGCATCCATGGCC

TCCGCGACCGGCTGCAGTTATCATCATCATCATAGACACACGAAATAAAGTAATCAGATTATCAGTTAAA

GCTATGTAATATTTACACCATAACCAATCAATTAAAAAATAGATCAGTTTAAAGAAAGATCAAAGCTCAA

AAAAATAAAAAGAGAAAAGGGTCCTAACCAAGAAAATGAAGGAGAAAAACTAGAAATTTACCTGCAGAAC

AGCGGGCAGTTCGGTTTCAGGCAGGTCTTGCAACGTGACACCCTGTGCACGGCGGGAGATGCAATAGGTC

AGGCTCTCGCTAAACTCCCCAATGTCAAGCACTTCCGGAATCGGGAGCGCGGCCGATGCAAAGTGCCGAT

AAACATAACGATCTTTGTAGAAACCATCGGCGCAGCTATTTACCCGCAGGACATATCCACGCCCTCCTAC

ATCGAAGCTGAAAGCACGAGATTCTTCGCCCTCCGAGAGCTGCATCAGGTCGGAGACGCTGTCGAACTTT

TCGATCAGAAACTTCTCGACAGACGTCGCGGTGAGTTCAGGCTTTTTCATATCTCATTGCCCCCCCGGAT

CTGCGAAAGCTCGACGGATCTTCGAGAGAGATAGATTTGTAGAGAGAGACTGGTGATTTCAGCGTGTCCT

CTCCAAATGAAATGAACTTCCTTATATAGAGGAAGGTCTTGCGAAGGATAGTGGGATTGTGCGTCATCCC

TTACGTCAGTGGAGATATCACATCAATCCACTTGCTTTGAAGACGTGGTTGGAACGTCTTCTTTTTCCAC

GATGCTCCTCGTGGGTGGGGGTCCATCTTTGGGACCACTGTCGGCAGAGGCATCTTGAACGATAGCCTTT

CCTTTATCGCAATGATGGCATTTGTAGGTGCCACCTTCCTTTTCTACTGTCCTTTTGATGAAGTGACAGA

TAGCTGGGCAATGGAATCCGAGGAGGTTTCCCGATATTACCCTTTGTTGAAAAGTCTCAATAGCCCTTTG

GTCTTCTGAGACTGTATCTTTGATATTCTTGGAGTAGACGAGAGTGTCGTGCTCCACCATGTTATCACAT

CAATCCACTTGCTTTGAAGACGTGGTTGGAACGTCTTCTTTTTCCACGATGCTCCTCGTGGGTGGGGGTC

CATCTTTGGGACCACTGTCGGCAGAGGCATCTTGAACGATAGCCTTTCCTTTATCGCAATGATGGCATTT

GTAGGTGCCACCTTCCTTTTCTACTGTCCTTTTGATGAAGTGACAGATAGCTGGGCAATGGAATCCGAGG

AGGTTTCCCGATATTACCCTTTGTTGAAAAGTCTCAATAGCCCTTTGGTCTTCTGAGACTGTATCTTTGA

TATTCTTGGAGTAGACGAGAGTGTCGTGCTCCACCATGTTGGCAAGCTGCTCTAGCCAATACGCAAACCG

CCTCTCCCCGCGCGTTGGCCGATTCATTAATGCAGCTGGCACGACAGGTTTCCCGACTGGAAAGCGGGCA

GTGAGCGCAACGCAATTAATGTGAGTTAGCTCACTCATTAGGCACCCCAGGCTTTACACTTTATGCTTCC

GGCTCGTATGTTGTGTGGAATTGTGAGCGGATAACAATTTCACACAGGAAACAGCTATGACCATGATTAC

>gi|11559661|gb|AF294978.1| Cloning vector pC1300intC, complete sequence

GAATTCAGATCTCTCGAGGAGCTCGGTACCCGGGGATCCTCTAGAGTCGACCTGCAGGCATGCAAGCTTG

GCACTGGCCGTCGTTTTACAACGTCGTGACTGGGAAAACCCTGGCGTTACCCAACTTAATCGCCTTGCAG

CACATCCCCCTTTCGCCAGCTGGCGTAATAGCGAAGAGGCCCGCACCGATCGCCCTTCCCAACAGTTGCG

CAGCCTGAATGGCGAATGCTAGAGCAGCTTGAGCTTGGATCAGATTGTCGTTTCCCGCCTTCAGTTTAAA

CTATCAGTGTTTGACAGGATATATTGGCGGGTAAACCTAAGAGAAAAGAGCGTTTATTAGAATAACGGAT

ATTTAAAAGGGCGTGAAAAGGTTTATCCGTTCGTCCATTTGTATGTGCATGCCAACCACAGGGTTCCCCT

CGGGATCAAAGTACTTTGATCCAACCCCTCCGCTGCTATAGTGCAGTCGGCTTCTGACGTTCAGTGCAGC

CGTCTTCTGAAAACGACATGTCGCACAAGTCCTAAGTTACGCGACAGGCTGCCGCCCTGCCCTTTTCCTG

GCGTTTTCTTGTCGCGTGTTTTAGTCGCATAAAGTAGAATACTTGCGACTAGAACCGGAGACATTACGCC

ATGAACAAGAGCGCCGCCGCTGGCCTGCTGGGCTATGCCCGCGTCAGCACCGACGACCAGGACTTGACCA

ACCAACGGGCCGAACTGCACGCGGCCGGCTGCACCAAGCTGTTTTCCGAGAAGATCACCGGCACCAGGCG

CGACCGCCCGGAGCTGGCCAGGATGCTTGACCACCTACGCCCTGGCGACGTTGTGACAGTGACCAGGCTA

GACCGCCTGGCCCGCAGCACCCGCGACCTACTGGACATTGCCGAGCGCATCCAGGAGGCCGGCGCGGGCC

TGCGTAGCCTGGCAGAGCCGTGGGCCGACACCACCACGCCGGCCGGCCGCATGGTGTTGACCGTGTTCGC

CGGCATTGCCGAGTTCGAGCGTTCCCTAATCATCGACCGCACCCGGAGCGGGCGCGAGGCCGCCAAGGCC

CGAGGCGTGAAGTTTGGCCCCCGCCCTACCCTCACCCCGGCACAGATCGCGCACGCCCGCGAGCTGATCG

ACCAGGAAGGCCGCACCGTGAAAGAGGCGGCTGCACTGCTTGGCGTGCATCGCTCGACCCTGTACCGCGC

ACTTGAGCGCAGCGAGGAAGTGACGCCCACCGAGGCCAGGCGGCGCGGTGCCTTCCGTGAGGACGCATTG

ACCGAGGCCGACGCCCTGGCGGCCGCCGAGAATGAACGCCAAGAGGAACAAGCATGAAACCGCACCAGGA

CGGCCAGGACGAACCGTTTTTCATTACCGAAGAGATCGAGGCGGAGATGATCGCGGCCGGGTACGTGTTC

GAGCCGCCCGCGCACGTCTCAACCGTGCGGCTGCATGAAATCCTGGCCGGTTTGTCTGATGCCAAGCTGG

CGGCCTGGCCGGCCAGCTTGGCCGCTGAAGAAACCGAGCGCCGCCGTCTAAAAAGGTGATGTGTATTTGA

GTAAAACAGCTTGCGTCATGCGGTCGCTGCGTATATGATGCGATGAGTAAATAAACAAATACGCAAGGGG

AACGCATGAAGGTTATCGCTGTACTTAACCAGAAAGGCGGGTCAGGCAAGACGACCATCGCAACCCATCT

AGCCCGCGCCCTGCAACTCGCCGGGGCCGATGTTCTGTTAGTCGATTCCGATCCCCAGGGCAGTGCCCGC

GATTGGGCGGCCGTGCGGGAAGATCAACCGCTAACCGTTGTCGGCATCGACCGCCCGACGATTGACCGCG

ACGTGAAGGCCATCGGCCGGCGCGACTTCGTAGTGATCGACGGAGCGCCCCAGGCGGCGGACTTGGCTGT

GTCCGCGATCAAGGCAGCCGACTTCGTGCTGATTCCGGTGCAGCCAAGCCCTTACGACATATGGGCCACC

GCCGACCTGGTGGAGCTGGTTAAGCAGCGCATTGAGGTCACGGATGGAAGGCTACAAGCGGCCTTTGTCG

TGTCGCGGGCGATCAAAGGCACGCGCATCGGCGGTGAGGTTGCCGAGGCGCTGGCCGGGTACGAGCTGCC

CATTCTTGAGTCCCGTATCACGCAGCGCGTGAGCTACCCAGGCACTGCCGCCGCCGGCACAACCGTTCTT

GAATCAGAACCCGAGGGCGACGCTGCCCGCGAGGTCCAGGCGCTGGCCGCTGAAATTAAATCAAAACTCA

TTTGAGTTAATGAGGTAAAGAGAAAATGAGCAAAAGCACAAACACGCTAAGTGCCGGCCGTCCGAGCGCA

CGCAGCAGCAAGGCTGCAACGTTGGCCAGCCTGGCAGACACGCCAGCCATGAAGCGGGTCAACTTTCAGT

TGCCGGCGGAGGATCACACCAAGCTGAAGATGTACGCGGTACGCCAAGGCAAGACCATTACCGAGCTGCT

ATCTGAATACATCGCGCAGCTACCAGAGTAAATGAGCAAATGAATAAATGAGTAGATGAATTTTAGCGGC

TAAAGGAGGCGGCATGGAAAATCAAGAACAACCAGGCACCGACGCCGTGGAATGCCCCATGTGTGGAGGA

ACGGGCGGTTGGCCAGGCGTAAGCGGCTGGGTTGTCTGCCGGCCCTGCAATGGCACTGGAACCCCCAAGC

CCGAGGAATCGGCGTGACGGTCGCAAACCATCCGGCCCGGTACAAATCGGCGCGGCGCTGGGTGATGACC

TGGTGGAGAAGTTGAAGGCCGCGCAGGCCGCCCAGCGGCAACGCATCGAGGCAGAAGCACGCCCCGGTGA

ATCGTGGCAAGCGGCCGCTGATCGAATCCGCAAAGAATCCCGGCAACCGCCGGCAGCCGGTGCGCCGTCG

ATTAGGAAGCCGCCCAAGGGCGACGAGCAACCAGATTTTTTCGTTCCGATGCTCTATGACGTGGGCACCC

GCGATAGTCGCAGCATCATGGACGTGGCCGTTTTCCGTCTGTCGAAGCGTGACCGACGAGCTGGCGAGGT

GATCCGCTACGAGCTTCCAGACGGGCACGTAGAGGTTTCCGCAGGGCCGGCCGGCATGGCCAGTGTGTGG

GATTACGACCTGGTACTGATGGCGGTTTCCCATCTAACCGAATCCATGAACCGATACCGGGAAGGGAAGG

GAGACAAGCCCGGCCGCGTGTTCCGTCCACACGTTGCGGACGTACTCAAGTTCTGCCGGCGAGCCGATGG

CGGAAAGCAGAAAGACGACCTGGTAGAAACCTGCATTCGGTTAAACACCACGCACGTTGCCATGCAGCGT

ACGAAGAAGGCCAAGAACGGCCGCCTGGTGACGGTATCCGAGGGTGAAGCCTTGATTAGCCGCTACAAGA

TCGTAAAGAGCGAAACCGGGCGGCCGGAGTACATCGAGATCGAGCTAGCTGATTGGATGTACCGCGAGAT

CACAGAAGGCAAGAACCCGGACGTGCTGACGGTTCACCCCGATTACTTTTTGATCGATCCCGGCATCGGC

CGTTTTCTCTACCGCCTGGCACGCCGCGCCGCAGGCAAGGCAGAAGCCAGATGGTTGTTCAAGACGATCT

ACGAACGCAGTGGCAGCGCCGGAGAGTTCAAGAAGTTCTGTTTCACCGTGCGCAAGCTGATCGGGTCAAA

TGACCTGCCGGAGTACGATTTGAAGGAGGAGGCGGGGCAGGCTGGCCCGATCCTAGTCATGCGCTACCGC

AACCTGATCGAGGGCGAAGCATCCGCCGGTTCCTAATGTACGGAGCAGATGCTAGGGCAAATTGCCCTAG

CAGGGGAAAAAGGTCGAAAAGGTCTCTTTCCTGTGGATAGCACGTACATTGGGAACCCAAAGCCGTACAT

TGGGAACCGGAACCCGTACATTGGGAACCCAAAGCCGTACATTGGGAACCGGTCACACATGTAAGTGACT

GATATAAAAGAGAAAAAAGGCGATTTTTCCGCCTAAAACTCTTTAAAACTTATTAAAACTCTTAAAACCC

GCCTGGCCTGTGCATAACTGTCTGGCCAGCGCACAGCCGAAGAGCTGCAAAAAGCGCCTACCCTTCGGTC

GCTGCGCTCCCTACGCCCCGCCGCTTCGCGTCGGCCTATCGCGGCCGCTGGCCGCTCAAAAATGGCTGGC

CTACGGCCAGGCAATCTACCAGGGCGCGGACAAGCCGCGCCGTCGCCACTCGACCGCCGGCGCCCACATC

AAGGCACCCTGCCTCGCGCGTTTCGGTGATGACGGTGAAAACCTCTGACACATGCAGCTCCCGGAGACGG

TCACAGCTTGTCTGTAAGCGGATGCCGGGAGCAGACAAGCCCGTCAGGGCGCGTCAGCGGGTGTTGGCGG

GTGTCGGGGCGCAGCCATGACCCAGTCACGTAGCGATAGCGGAGTGTATACTGGCTTAACTATGCGGCAT

CAGAGCAGATTGTACTGAGAGTGCACCATATGCGGTGTGAAATACCGCACAGATGCGTAAGGAGAAAATA

CCGCATCAGGCGCTCTTCCGCTTCCTCGCTCACTGACTCGCTGCGCTCGGTCGTTCGGCTGCGGCGAGCG

GTATCAGCTCACTCAAAGGCGGTAATACGGTTATCCACAGAATCAGGGGATAACGCAGGAAAGAACATGT

GAGCAAAAGGCCAGCAAAAGGCCAGGAACCGTAAAAAGGCCGCGTTGCTGGCGTTTTTCCATAGGCTCCG

CCCCCCTGACGAGCATCACAAAAATCGACGCTCAAGTCAGAGGTGGCGAAACCCGACAGGACTATAAAGA

TACCAGGCGTTTCCCCCTGGAAGCTCCCTCGTGCGCTCTCCTGTTCCGACCCTGCCGCTTACCGGATACC

TGTCCGCCTTTCTCCCTTCGGGAAGCGTGGCGCTTTCTCATAGCTCACGCTGTAGGTATCTCAGTTCGGT

GTAGGTCGTTCGCTCCAAGCTGGGCTGTGTGCACGAACCCCCCGTTCAGCCCGACCGCTGCGCCTTATCC

GGTAACTATCGTCTTGAGTCCAACCCGGTAAGACACGACTTATCGCCACTGGCAGCAGCCACTGGTAACA

GGATTAGCAGAGCGAGGTATGTAGGCGGTGCTACAGAGTTCTTGAAGTGGTGGCCTAACTACGGCTACAC

TAGAAGGACAGTATTTGGTATCTGCGCTCTGCTGAAGCCAGTTACCTTCGGAAAAAGAGTTGGTAGCTCT

TGATCCGGCAAACAAACCACCGCTGGTAGCGGTGGTTTTTTTGTTTGCAAGCAGCAGATTACGCGCAGAA

AAAAAGGATCTCAAGAAGATCCTTTGATCTTTTCTACGGGGTCTGACGCTCAGTGGAACGAAAACTCACG

TTAAGGGATTTTGGTCATGCATTCTAGGTACTAAAACAATTCATCCAGTAAAATATAATATTTTATTTTC

TCCCAATCAGGCTTGATCCCCAGTAAGTCAAAAAATAGCTCGACATACTGTTCTTCCCCGATATCCTCCC

TGATCGACCGGACGCAGAAGGCAATGTCATACCACTTGTCCGCCCTGCCGCTTCTCCCAAGATCAATAAA

GCCACTTACTTTGCCATCTTTCACAAAGATGTTGCTGTCTCCCAGGTCGCCGTGGGAAAAGACAAGTTCC

TCTTCGGGCTTTTCCGTCTTTAAAAAATCATACAGCTCGCGCGGATCTTTAAATGGAGTGTCTTCTTCCC

AGTTTTCGCAATCCACATCGGCCAGATCGTTATTCAGTAAGTAATCCAATTCGGCTAAGCGGCTGTCTAA

GCTATTCGTATAGGGACAATCCGATATGTCGATGGAGTGAAAGAGCCTGATGCACTCCGCATACAGCTCG

ATAATCTTTTCAGGGCTTTGTTCATCTTCATACTCTTCCGAGCAAAGGACGCCATCGGCCTCACTCATGA

GCAGATTGCTCCAGCCATCATGCCGTTCAAAGTGCAGGACCTTTGGAACAGGCAGCTTTCCTTCCAGCCA

TAGCATCATGTCCTTTTCCCGTTCCACATCATAGGTGGTCCCTTTATACCGGCTGTCCGTCATTTTTAAA

TATAGGTTTTCATTTTCTCCCACCAGCTTATATACCTTAGCAGGAGACATTCCTTCCGTATCTTTTACGC

AGCGGTATTTTTCGATCAGTTTTTTCAATTCCGGTGATATTCTCATTTTAGCCATTTATTATTTCCTTCC

TCTTTTCTACAGTATTTAAAGATACCCCAAGAAGCTAATTATAACAAGACGAACTCCAATTCACTGTTCC

TTGCATTCTAAAACCTTAAATACCAGAAAACAGCTTTTTCAAAGTTGTTTTCAAAGTTGGCGTATAACAT

AGTATCGACGGAGCCGATTTTGAAACCGCGGTGATCACAGGCAGCAACGCTCTGTCATCGTTACAATCAA

CATGCTACCCTCCGCGAGATCATCCGTGTTTCAAACCCGGCAGCTTAGTTGCCGTTCTTCCGAATAGCAT

CGGTAACATGAGCAAAGTCTGCCGCCTTACAACGGCTCTCCCGCTGACGCCGTCCCGGACTGATGGGCTG

CCTGTATCGAGTGGTGATTTTGTGCCGAGCTGCCGGTCGGGGAGCTGTTGGCTGGCTGGTGGCAGGATAT

ATTGTGGTGTAAACAAATTGACGCTTAGACAACTTAATAACACATTGCGGACGTTTTTAATGTACTGAAT

TAACGCCGAATTAATTCGGGGGATCTGGATTTTAGTACTGGATTTTGGTTTTAGGAATTAGAAATTTTAT

TGATAGAAGTATTTTACAAATACAAATACATACTAAGGGTTTCTTATATGCTCAACACATGAGCGAAACC

CTATAGGAACCCTAATTCCCTTATCTGGGAACTACTCACACATTATTATGGAGAAACTCGAAGATCCGTC

GAGCTTGTCGATCGACAGATCCGGTCGGCATCTACTCTATTTCTTTGCCCTCGGACGAGTGCTGGGGCGT

CGGTTTCCACTATCGGCGAGTACTTCTACACAGCCATCGGTCCAGACGGCCGCGCTTCTGCGGGCGATTT

GTGTACGCCCGACAGTCCCGGCTCCGGATCGGACGATTGCGTCGCATCGACCCTGCGCCCAAGCTGCATC

ATCGAAATTGCCGTCAACCAAGCTCTGATAGAGTTGGTCAAGACCAATGCGGAGCATATACGCCCGGAGT

CGTGGCGATCCTGCAAGCTCCGGATGCCTCCGCTCGAAGTAGCGCGTCTGCTGCTCCATACAAGCCAACC

ACGGCCTCCAGAAGAAGATGTTGGCGACCTCGTATTGGGAATCCCCGAACATCGCCTCGCTCCAGTCAAT

GACCGCTGTTATGCGGCCATTGTCCGTCAGGACATTGTTGGAGCCGAAATCCGCGTGCACGAGGTGCCGG

ACTTCGGGGCAGTCCTCGGCCCAAAGCATCAGCTCATCGAGAGCCTGCGCGACGGACGCACTGACGGTGT

CGTCCATCACAGTTTGCCAGTGATACACATGGGGATCAGCAATCGCGCATATGAAATCACGCCATGTAGT

GTATTGACCGATTCCTTGCGGTCCGAATGGGCCGAACCCGCTCGTCTGGCTAAGATCGGCCGCAGCGATC

GCATCCATGGCCTCCGCGACCGGCTGCAGTTATCATCATCATCATAGACACACGAAATAAAGTAATCAGA

TTATCAGTTAAAGCTATGTAATATTTACACCATAACCAATCAATTAAAAAATAGATCAGTTTAAAGAAAG

ATCAAAGCTCAAAAAAATAAAAAGAGAAAAGGGTCCTAACCAAGAAAATGAAGGAGAAAAACTAGAAATT

TACCTGCAGAACAGCGGGCAGTTCGGTTTCAGGCAGGTCTTGCAACGTGACACCCTGTGCACGGCGGGAG

ATGCAATAGGTCAGGCTCTCGCTAAACTCCCCAATGTCAAGCACTTCCGGAATCGGGAGCGCGGCCGATG

CAAAGTGCCGATAAACATAACGATCTTTGTAGAAACCATCGGCGCAGCTATTTACCCGCAGGACATATCC

ACGCCCTCCTACATCGAAGCTGAAAGCACGAGATTCTTCGCCCTCCGAGAGCTGCATCAGGTCGGAGACG

CTGTCGAACTTTTCGATCAGAAACTTCTCGACAGACGTCGCGGTGAGTTCAGGCTTTTTCATATCTCATT

GCCCCCCCGGATCTGCGAAAGCTCGACGGATCTTCGAGAGAGATAGATTTGTAGAGAGAGACTGGTGATT

TCAGCGTGTCCTCTCCAAATGAAATGAACTTCCTTATATAGAGGAAGGTCTTGCGAAGGATAGTGGGATT

GTGCGTCATCCCTTACGTCAGTGGAGATATCACATCAATCCACTTGCTTTGAAGACGTGGTTGGAACGTC

TTCTTTTTCCACGATGCTCCTCGTGGGTGGGGGTCCATCTTTGGGACCACTGTCGGCAGAGGCATCTTGA

ACGATAGCCTTTCCTTTATCGCAATGATGGCATTTGTAGGTGCCACCTTCCTTTTCTACTGTCCTTTTGA

TGAAGTGACAGATAGCTGGGCAATGGAATCCGAGGAGGTTTCCCGATATTACCCTTTGTTGAAAAGTCTC

AATAGCCCTTTGGTCTTCTGAGACTGTATCTTTGATATTCTTGGAGTAGACGAGAGTGTCGTGCTCCACC

ATGTTATCACATCAATCCACTTGCTTTGAAGACGTGGTTGGAACGTCTTCTTTTTCCACGATGCTCCTCG

TGGGTGGGGGTCCATCTTTGGGACCACTGTCGGCAGAGGCATCTTGAACGATAGCCTTTCCTTTATCGCA

ATGATGGCATTTGTAGGTGCCACCTTCCTTTTCTACTGTCCTTTTGATGAAGTGACAGATAGCTGGGCAA

TGGAATCCGAGGAGGTTTCCCGATATTACCCTTTGTTGAAAAGTCTCAATAGCCCTTTGGTCTTCTGAGA

CTGTATCTTTGATATTCTTGGAGTAGACGAGAGTGTCGTGCTCCACCATGTTGGCAAGCTGCTCTAGCCA

ATACGCAAACCGCCTCTCCCCGCGCGTTGGCCGATTCATTAATGCAGCTGGCACGACAGGTTTCCCGACT

GGAAAGCGGGCAGTGAGCGCAACGCAATTAATGTGAGTTAGCTCACTCATTAGGCACCCCAGGCTTTACA

CTTTATGCTTCCGGCTCGTATGTTGTGTGGAATTGTGAGCGGATAACAATTTCACACAGGAAACAGCTAT

GACCATGATTAC

>gi|11559663|gb|AF294979.1| Binary vector pINDEX1, complete sequence

GAATTCGAGCTCGGTACCCGGGGATCCTCTAGAATCCGAAAAGTTTCTGCACCGTTTTCACCCCCTAACT

AACAATATAGGGAACGTGTGCTAAATATAAAATGAGACCTTATATATGTAGCGCTGATAACTAGAACTAT

GCAAGAAAAACTCATCCACCTACTTTAGTGGCAATCGGGCTAAATAAAAAAGAGTCGCTACACTAGCTAG

TTTCGTTTTCCTTAGTAATTAAGTGGGAAAATGAAATCATTATTGCTTAGAATATACGTTCACATCTCTG

TCATGAAGTTAAATTATTCGAGGTAGCCATAATTGTCATCAAACTCTTCTTGAATAAAAAAATCTTTCTA

GCTGAACTCAATGGGTAAAGAGAGAGATTTTTTTTAAAAAAATAGAATGAAGATATTCTGAACGTATTGG

CAAAGATTTAAACATATAATTATATAATTTTATAGTTTGTGCATTCGTCATATCGCACATCATTAAGGAC

ATGTCTTACTCCATCCCAATTTTTATTTAGTAATTAAAGACAATTGACTTATTTTTATTATTTATCTTTT

TTCGATTAGATGCAAGGTACTTACGCACACACTTTGTGCTCATGTGCATGTGTGAGTGCACCTCCTCAAT

ACACGTTCAACTAGCAACACATCTCTAATATCACTCGCCTATTTAATACATTTAGGTAGCAATATCTGAA

TTCAAGCACTCCACCATCACCAGACCACTTTTAATAATATCTAAAATACAAAAAATAATTTTACAGAATA

GCATGAAAAGTATGAAACGAACTATTTAGGTTTTTCACATACAAAAAAAAAAAGAATTTTGCTCGTGCGC

GAGCGCCAATCTCCCATATTGGGCACACAGGCAACAACAGAGTGGCTGCCCACAGAACAACCCACAAAAA

ACGATGATCTAACGGAGGACAGCAAGTCCGCAACAACCTTTTAACAGCAGGCTTTGCGGCCAGGAGAGAG

GAGGAGAGGCAAAGAAAACCAAGCATCCTCCTCCTCCCATCTATAAATTCCTCCCCCCTTTTCCCCTCTC

TATATAGGAGGCATCCAAGCCAAGAAGAGGGAGAGCACCAAGGACACGCGACTAGCAGAAGCCGAGCGAC

CGCCTTCTTCGATCCATATCTTCCGGTCGAGTTCTTGGTCGATCTCTTCCCTCCTCCACCTCCTCCTCAC

AGGGTATGTGCCCTTCGGTTGTTCTTGGATTTATTGTTCTAGGTTGTGTAGTACGGGCGTTGATGTTAGG

AAAGGGGATCTGTATCTGTGATGATTCCTGTTCTTGGATTTGGGATAGAGGGGTTCTTGATGTTGCATGT

TATCGGTTCGGTTTGATTAGTAGTATGGTTTTCAATCGTCTGGAGAGCTCTATGGAAATGAAATGGTTTA

GGGTACGGAATCTTGCGATTTTGTGAGTACCTTTTGTTTGAGGTAAAATCAGAGCACCGGTGATTTTGCT

TGGTGTAATAAAAGTACGGTTGTTTGGTCCTCGATTCTGGTAGTGATGCTTCTCGATTTGACGAAGCTAT

CCTTTGTTTATTCCCTATTGAACAAAAATAATCCAACTTTGAAGACGGTCCCGTTGATGAGATTGAATGA

TTGATTCTTAAGCCTGTCCAAAATTTCGCAGCTGGCTTGTTTAGATACAGTAGTCCCCATCACGAAATTC

ATGGAAACAGTTATAATCCTCAGGAACAGGGGATTCCCTGTTCTTCCGATTTGCTTTAGTCCCAGAATTT

TTTTTCCCAAATATCTTAAAAAGTCACTTTCTGGTTCAGTTCAATGAATTGATTGCTACAAATAATGCTT

TTATAGCGTTATCCTAGCTGTAGTTCAGTTAATAGGTAATACCCCTATAGTTTAGTCAGGAGAAGAACTT

ATCCGATTTCTGATCTCCATTTTTAATTATATGAAATGAACTGTAGCATAAGCAGTATTCATTTGGATTA

TTTTTTTTATTAGCTCTCACCCCTTCATTATTCTGAGCTGAAAGTCTGGCATGAACTGTCCTCAATTTTG

TTTTCAAATTCACATCGATTATCTATCGATTATCCTCTTGTATCTACCTGTAGAAGTTTCTTTTTGGTTA

TTCCTTGACTGCTTGATTACAGAAAGAAATTTATGAAGCTGTAATCGGGATAGTTATACTGCTTGTTCTT

ATGATTCATTTCCTTTGTGCAGTTCTTGGTGTAGCTTGCCACTTTCACCAGCAAAGTTCCATAAAACAAT

GGCAGATCCAATGAAGCTACTGTCTTCTATCGAACAAGCATGCGATATTTGCCGACTTAAAAAGCTCAAG

TGCTCCAAAGAAAAACCGAAGTGCGCCAAGTGTCTGAAGAACAACTGGGAGTGTCGCTACTCTCCCAAAA

CCAAAAGGTCTCCGCTGACTAGGGCACATCTGACAGAAGTGGAATCAAGGCTAGAAAGACTGGAACAGCT

ATTTCTACTGATTTTTCCTAGGTCGAGCGCCCCCCCGACCGATGTCAGCCTGGGGGACGAGCTCCACTTA

GACGGCGAGGACGTGGCGATGGCGCATGCCGACGCGCTAGACGATTTCGATCTGGACATGTTGGGGGACG

GGGATTCCCCGGGTCCGGGATTTACCCCCCACGACTCCGCCCCCTACGGCGCTCTGGATATGGCCGACTT

CGAGTTTGAGCAGATGTTTACCGATGCCCTTGGAATTGACGAGTACGGTGGGGATCCAATTCAGCAAGCC

ACTGCAGGAGTCTCACAAGACACTTCGGAAAATCCTAACAAAACAATAGTTCCTGCTGCATTACCACAGC

TCACCCCTACCTTGGTGTCACTGCTGGAGGTGATTGAACCCGAGGTGTTGTATGCAGGATATGATAGCTC

TGTTCCAGATTCAGCATGGAGAATTATGACCACACTCAACATGTTAGGTGGGCGTCAAGTGATTGCAGCA

GTGAAATGGGCAAAGGCGATACCAGGCTTCAGAAACTTACACCTGGATGACCAAATGACCCTGCTACAGT

ACTCATGGATGTTTCTCATGGCATTTGCCCTGGGTTGGAGATCATACAGACAATCAAGTGGAAACCTGCT

CTGCTTTGCTCCTGATCTGATTATTAATGAGCAGAGAATGTCTCTACCCTGCATGTATGACCAATGTAAA

CACATGCTGTTTGTCTCCTCTGAATTACAAAGATTGCAGGTATCCTATGAAGAGTATCTCTGTATGAAAA

CCTTACTGCTTCTCTCCTCAGTTCCTAAGGAAGGTCTGAAGAGCCAAGAGTTATTTGATGAGATTCGAAT

GACTTATATCAAAGAGCTAGGAAAAGCCATCGTCAAAAGGGAAGGGAACTCCAGTCAGAACTGGCAACGG

TTTTACCAACTGACAAAGCTTCTGGACTCCATGCATGAGGTGGTTGAGAATCTCCTTACCTACTGCTTCC

AGACATTTTTGGATAAGACCATGAGTATTGAATTCCCAGAGATGTTAGCTGAAATCATCACTAATCAGAT

ACCAAAATATTCAAATGGAAATATCAAAAAGCTTCTGTTTCATCAAAAATGACTCGACCTAACTGAGTAA

GCTAGCTTGTTCGAGTATTATGGCATTGGGAAAACTGTTTTTCTTGTACCATTTGTTGTGCTTGTAATTT

ACTGTGTTTTTTATTCGGTTTTCGCTATCGAACTGTGAAATGGAAATGGATGGAGAAGAGTTAATGAATG

ATATGGTCCTTTTGTTCATTCTCAAATTAATATTATTTGTTTTTTCTCTTATTTGTTGTGTGTTGAATTT

GAAATTATAAGAGATATGCAAACATTTTGTTTTGAGTAAAAATGTGTCAAATCGTGGCCTCTAATGACCG

AAGTTAATATGAGGAGTAAAACACTAGATCCCCAAACAAGCTTGAAACTGAAGGCGGGAAACGACAATCT

GATCATGGGCGGCCGTCTGGACCGATGGCTGTGTAGAAGTACTCGCCGATAGTGGAAACCGACGCCCCAG

CACTCGTCCGGGATCTTGGAGGTGATGTAACATGATCACAAGCTGATCCCCCGAATTTCCCCGATCGTTC

AAACATTTGGCAATAAAGTTTCTTAAGATTGAATCCTGTTGCCGGTCTTGCGATGATTATCATATAATTT

CTGTTGAATTACGTTAAGCATGTAATAATTAACATGTAATGCATGACGTTATTTATGAGATGGGTTTTTA

TGATTAGAGTCCCGCAATTATACATTTAATACGCGATAGAAAACAAAATATAGCGCGCAAACTAGGATAA

ATTATCGCGCGCGGTGTCATCTATGTTACTAGATCGGGAATTGATCCCCCCTCGACAGCTTGCATGCCGG

TCGACTCTAGAGGATCCGGGTGACAGCCCTCCGACGGGTGACAGCCCTCCGACGGGTGACAGCCCTCCGA

CGGGTGACAGCCCTCCGAATTCGAGCTCGGTACCCGGGGATCTGTCGACCTCGATCGAGATCTTCGCAAG

ACCCTTCCTCTATATAAGGAAGTTCATTTCATTTGGAGAGGACACGCTGAAGCTAGTCGACTCTAGCCTC

GAGGCTAGAGGATCGACTAGTCGATCCAGGCCTCCCAGCTTTCGTCCGTATCATCGGTTTCGACAACGTT

CGTCAAGTTCAATGCATCAGTTTCATTGCCCACACACCAGAATCCTACTAAGTTTGAGTATTATGGCATT

GGAAAAGCTGTTTTCTTCTATCATTTGTTCTGCTTGTAATTTACTGTGTTCTTTCAGTTTTTGTTTTCGG

ACATCAAAATGCAAATGGATGGATAAGAGTTAATAAATGATATGGTCCTTTTGTTCATTCTCAAATTATT

ATTATCTGTTGTTTTTACTTTAATGGGTTGAATTTAAGTAAGAAAGGAACTAACAGTGTGATATTAAGGT

GCAATGTTAGACATATAAAACAGTCTTTCACCTCTCTTTGGTTATGTCTTGAATTGGTTTGTTTCTTCAC

TTATCTGTGTAATCAAGTTTACTATGAGTCTATGATCAAGTAATTATGCAATCAAGTTAAGTACAGTATA

GGCTTTTTGTGTCGAGGGGGTACCGAGTCGAGGAATTCGATATCAAGCTTGGCACTGGCCGTCGTTTTAC

AACGTCGTGACTGGGAAAACCCTGGCGTTACCCAACTTAATCGCCTTGCAGCACATCCCCCTTTCGCCAG

CTGGCGTAATAGCGAAGAGGCCCGCACCGATCGCCCTTCCCAACAGTTGCGCAGCCTGAATGGCGAATGC

TAGAGCAGCTTGAGCTTGGATCAGATTGTCGTTTCCCGCCTTCAGTTTAAACTATCAGTGTTTGACAGGA

TATATTGGCGGGTAAACCTAAGAGAAAAGAGCGTTTATTAGAATAACGGATATTTAAAAGGGCGTGAAAA

GGTTTATCCGTTCGTCCATTTGTATGTGCATGCCAACCACAGGGTTCCCCTCGGGATCAAAGTACTTTGA

TCCAACCCCTCCGCTGCTATAGTGCAGTCGGCTTCTGACGTTCAGTGCAGCCGTCTTCTGAAAACGACAT

GTCGCACAAGTCCTAAGTTACGCGACAGGCTGCCGCCCTGCCCTTTTCCTGGCGTTTTCTTGTCGCGTGT

TTTAGTCGCATAAAGTAGAATACTTGCGACTAGAACCGGAGACATTACGCCATGAACAAGAGCGCCGCCG

CTGGCCTGCTGGGCTATGCCCGCGTCAGCACCGACGACCAGGACTTGACCAACCAACGGGCCGAACTGCA

CGCGGCCGGCTGCACCAAGCTGTTTTCCGAGAAGATCACCGGCACCAGGCGCGACCGCCCGGAGCTGGCC

AGGATGCTTGACCACCTACGCCCTGGCGACGTTGTGACAGTGACCAGGCTAGACCGCCTGGCCCGCAGCA

CCCGCGACCTACTGGACATTGCCGAGCGCATCCAGGAGGCCGGCGCGGGCCTGCGTAGCCTGGCAGAGCC

GTGGGCCGACACCACCACGCCGGCCGGCCGCATGGTGTTGACCGTGTTCGCCGGCATTGCCGAGTTCGAG

CGTTCCCTAATCATCGACCGCACCCGGAGCGGGCGCGAGGCCGCCAAGGCCCGAGGCGTGAAGTTTGGCC

CCCGCCCTACCCTCACCCCGGCACAGATCGCGCACGCCCGCGAGCTGATCGACCAGGAAGGCCGCACCGT

GAAAGAGGCGGCTGCACTGCTTGGCGTGCATCGCTCGACCCTGTACCGCGCACTTGAGCGCAGCGAGGAA

GTGACGCCCACCGAGGCCAGGCGGCGCGGTGCCTTCCGTGAGGACGCATTGACCGAGGCCGACGCCCTGG

CGGCCGCCGAGAATGAACGCCAAGAGGAACAAGCATGAAACCGCACCAGGACGGCCAGGACGAACCGTTT

TTCATTACCGAAGAGATCGAGGCGGAGATGATCGCGGCCGGGTACGTGTTCGAGCCGCCCGCGCACGTCT

CAACCGTGCGGCTGCATGAAATCCTGGCCGGTTTGTCTGATGCCAAGCTGGCGGCCTGGCCGGCCAGCTT

GGCCGCTGAAGAAACCGAGCGCCGCCGTCTAAAAAGGTGATGTGTATTTGAGTAAAACAGCTTGCGTCAT

GCGGTCGCTGCGTATATGATGCGATGAGTAAATAAACAAATACGCAAGGGGAACGCATGAAGGTTATCGC

TGTACTTAACCAGAAAGGCGGGTCAGGCAAGACGACCATCGCAACCCATCTAGCCCGCGCCCTGCAACTC

GCCGGGGCCGATGTTCTGTTAGTCGATTCCGATCCCCAGGGCAGTGCCCGCGATTGGGCGGCCGTGCGGG

AAGATCAACCGCTAACCGTTGTCGGCATCGACCGCCCGACGATTGACCGCGACGTGAAGGCCATCGGCCG

GCGCGACTTCGTAGTGATCGACGGAGCGCCCCAGGCGGCGGACTTGGCTGTGTCCGCGATCAAGGCAGCC

GACTTCGTGCTGATTCCGGTGCAGCCAAGCCCTTACGACATATGGGCCACCGCCGACCTGGTGGAGCTGG

TTAAGCAGCGCATTGAGGTCACGGATGGAAGGCTACAAGCGGCCTTTGTCGTGTCGCGGGCGATCAAAGG

CACGCGCATCGGCGGTGAGGTTGCCGAGGCGCTGGCCGGGTACGAGCTGCCCATTCTTGAGTCCCGTATC

ACGCAGCGCGTGAGCTACCCAGGCACTGCCGCCGCCGGCACAACCGTTCTTGAATCAGAACCCGAGGGCG

ACGCTGCCCGCGAGGTCCAGGCGCTGGCCGCTGAAATTAAATCAAAACTCATTTGAGTTAATGAGGTAAA

GAGAAAATGAGCAAAAGCACAAACACGCTAAGTGCCGGCCGTCCGAGCGCACGCAGCAGCAAGGCTGCAA

CGTTGGCCAGCCTGGCAGACACGCCAGCCATGAAGCGGGTCAACTTTCAGTTGCCGGCGGAGGATCACAC

CAAGCTGAAGATGTACGCGGTACGCCAAGGCAAGACCATTACCGAGCTGCTATCTGAATACATCGCGCAG

CTACCAGAGTAAATGAGCAAATGAATAAATGAGTAGATGAATTTTAGCGGCTAAAGGAGGCGGCATGGAA

AATCAAGAACAACCAGGCACCGACGCCGTGGAATGCCCCATGTGTGGAGGAACGGGCGGTTGGCCAGGCG

TAAGCGGCTGGGTTGTCTGCCGGCCCTGCAATGGCACTGGAACCCCCAAGCCCGAGGAATCGGCGTGACG

GTCGCAAACCATCCGGCCCGGTACAAATCGGCGCGGCGCTGGGTGATGACCTGGTGGAGAAGTTGAAGGC

CGCGCAGGCCGCCCAGCGGCAACGCATCGAGGCAGAAGCACGCCCCGGTGAATCGTGGCAAGCGGCCGCT

GATCGAATCCGCAAAGAATCCCGGCAACCGCCGGCAGCCGGTGCGCCGTCGATTAGGAAGCCGCCCAAGG

GCGACGAGCAACCAGATTTTTTCGTTCCGATGCTCTATGACGTGGGCACCCGCGATAGTCGCAGCATCAT

GGACGTGGCCGTTTTCCGTCTGTCGAAGCGTGACCGACGAGCTGGCGAGGTGATCCGCTACGAGCTTCCA

GACGGGCACGTAGAGGTTTCCGCAGGGCCGGCCGGCATGGCCAGTGTGTGGGATTACGACCTGGTACTGA

TGGCGGTTTCCCATCTAACCGAATCCATGAACCGATACCGGGAAGGGAAGGGAGACAAGCCCGGCCGCGT

GTTCCGTCCACACGTTGCGGACGTACTCAAGTTCTGCCGGCGAGCCGATGGCGGAAAGCAGAAAGACGAC

CTGGTAGAAACCTGCATTCGGTTAAACACCACGCACGTTGCCATGCAGCGTACGAAGAAGGCCAAGAACG

GCCGCCTGGTGACGGTATCCGAGGGTGAAGCCTTGATTAGCCGCTACAAGATCGTAAAGAGCGAAACCGG

GCGGCCGGAGTACATCGAGATCGAGCTAGCTGATTGGATGTACCGCGAGATCACAGAAGGCAAGAACCCG

GACGTGCTGACGGTTCACCCCGATTACTTTTTGATCGATCCCGGCATCGGCCGTTTTCTCTACCGCCTGG

CACGCCGCGCCGCAGGCAAGGCAGAAGCCAGATGGTTGTTCAAGACGATCTACGAACGCAGTGGCAGCGC

CGGAGAGTTCAAGAAGTTCTGTTTCACCGTGCGCAAGCTGATCGGGTCAAATGACCTGCCGGAGTACGAT

TTGAAGGAGGAGGCGGGGCAGGCTGGCCCGATCCTAGTCATGCGCTACCGCAACCTGATCGAGGGCGAAG

CATCCGCCGGTTCCTAATGTACGGAGCAGATGCTAGGGCAAATTGCCCTAGCAGGGGAAAAAGGTCGAAA

AGGTCTCTTTCCTGTGGATAGCACGTACATTGGGAACCCAAAGCCGTACATTGGGAACCGGAACCCGTAC

ATTGGGAACCCAAAGCCGTACATTGGGAACCGGTCACACATGTAAGTGACTGATATAAAAGAGAAAAAAG

GCGATTTTTCCGCCTAAAACTCTTTAAAACTTATTAAAACTCTTAAAACCCGCCTGGCCTGTGCATAACT

GTCTGGCCAGCGCACAGCCGAAGAGCTGCAAAAAGCGCCTACCCTTCGGTCGCTGCGCTCCCTACGCCCC

GCCGCTTCGCGTCGGCCTATCGCGGCCGCTGGCCGCTCAAAAATGGCTGGCCTACGGCCAGGCAATCTAC

CAGGGCGCGGACAAGCCGCGCCGTCGCCACTCGACCGCCGGCGCCCACATCAAGGCACCCTGCCTCGCGC

GTTTCGGTGATGACGGTGAAAACCTCTGACACATGCAGCTCCCGGAGACGGTCACAGCTTGTCTGTAAGC

GGATGCCGGGAGCAGACAAGCCCGTCAGGGCGCGTCAGCGGGTGTTGGCGGGTGTCGGGGCGCAGCCATG

ACCCAGTCACGTAGCGATAGCGGAGTGTATACTGGCTTAACTATGCGGCATCAGAGCAGATTGTACTGAG

AGTGCACCATATGCGGTGTGAAATACCGCACAGATGCGTAAGGAGAAAATACCGCATCAGGCGCTCTTCC

GCTTCCTCGCTCACTGACTCGCTGCGCTCGGTCGTTCGGCTGCGGCGAGCGGTATCAGCTCACTCAAAGG

CGGTAATACGGTTATCCACAGAATCAGGGGATAACGCAGGAAAGAACATGTGAGCAAAAGGCCAGCAAAA

GGCCAGGAACCGTAAAAAGGCCGCGTTGCTGGCGTTTTTCCATAGGCTCCGCCCCCCTGACGAGCATCAC

AAAAATCGACGCTCAAGTCAGAGGTGGCGAAACCCGACAGGACTATAAAGATACCAGGCGTTTCCCCCTG

GAAGCTCCCTCGTGCGCTCTCCTGTTCCGACCCTGCCGCTTACCGGATACCTGTCCGCCTTTCTCCCTTC

GGGAAGCGTGGCGCTTTCTCATAGCTCACGCTGTAGGTATCTCAGTTCGGTGTAGGTCGTTCGCTCCAAG

CTGGGCTGTGTGCACGAACCCCCCGTTCAGCCCGACCGCTGCGCCTTATCCGGTAACTATCGTCTTGAGT

CCAACCCGGTAAGACACGACTTATCGCCACTGGCAGCAGCCACTGGTAACAGGATTAGCAGAGCGAGGTA

TGTAGGCGGTGCTACAGAGTTCTTGAAGTGGTGGCCTAACTACGGCTACACTAGAAGGACAGTATTTGGT

ATCTGCGCTCTGCTGAAGCCAGTTACCTTCGGAAAAAGAGTTGGTAGCTCTTGATCCGGCAAACAAACCA

CCGCTGGTAGCGGTGGTTTTTTTGTTTGCAAGCAGCAGATTACGCGCAGAAAAAAAGGATCTCAAGAAGA

TCCTTTGATCTTTTCTACGGGGTCTGACGCTCAGTGGAACGAAAACTCACGTTAAGGGATTTTGGTCATG

CATTCTAGGTACTAAAACAATTCATCCAGTAAAATATAATATTTTATTTTCTCCCAATCAGGCTTGATCC

CCAGTAAGTCAAAAAATAGCTCGACATACTGTTCTTCCCCGATATCCTCCCTGATCGACCGGACGCAGAA

GGCAATGTCATACCACTTGTCCGCCCTGCCGCTTCTCCCAAGATCAATAAAGCCACTTACTTTGCCATCT

TTCACAAAGATGTTGCTGTCTCCCAGGTCGCCGTGGGAAAAGACAAGTTCCTCTTCGGGCTTTTCCGTCT

TTAAAAAATCATACAGCTCGCGCGGATCTTTAAATGGAGTGTCTTCTTCCCAGTTTTCGCAATCCACATC

GGCCAGATCGTTATTCAGTAAGTAATCCAATTCGGCTAAGCGGCTGTCTAAGCTATTCGTATAGGGACAA

TCCGATATGTCGATGGAGTGAAAGAGCCTGATGCACTCCGCATACAGCTCGATAATCTTTTCAGGGCTTT

GTTCATCTTCATACTCTTCCGAGCAAAGGACGCCATCGGCCTCACTCATGAGCAGATTGCTCCAGCCATC

ATGCCGTTCAAAGTGCAGGACCTTTGGAACAGGCAGCTTTCCTTCCAGCCATAGCATCATGTCCTTTTCC

CGTTCCACATCATAGGTGGTCCCTTTATACCGGCTGTCCGTCATTTTTAAATATAGGTTTTCATTTTCTC

CCACCAGCTTATATACCTTAGCAGGAGACATTCCTTCCGTATCTTTTACGCAGCGGTATTTTTCGATCAG

TTTTTTCAATTCCGGTGATATTCTCATTTTAGCCATTTATTATTTCCTTCCTCTTTTCTACAGTATTTAA

AGATACCCCAAGAAGCTAATTATAACAAGACGAACTCCAATTCACTGTTCCTTGCATTCTAAAACCTTAA

ATACCAGAAAACAGCTTTTTCAAAGTTGTTTTCAAAGTTGGCGTATAACATAGTATCGACGGAGCCGATT

TTGAAACCGCGGTGATCACAGGCAGCAACGCTCTGTCATCGTTACAATCAACATGCTACCCTCCGCGAGA

TCATCCGTGTTTCAAACCCGGCAGCTTAGTTGCCGTTCTTCCGAATAGCATCGGTAACATGAGCAAAGTC

TGCCGCCTTACAACGGCTCTCCCGCTGACGCCGTCCCGGACTGATGGGCTGCCTGTATCGAGTGGTGATT

TTGTGCCGAGCTGCCGGTCGGGGAGCTGTTGGCTGGCTGGTGGCAGGATATATTGTGGTGTAAACAAATT

GACGCTTAGACAACTTAATAACACATTGCGGACGTTTTTAATGTACTGAATTAACGCCGAATTAATTCGG

GGGATCTGGATTTTAGTACTGGATTTTGGTTTTAGGAATTAGAAATTTTATTGATAGAAGTATTTTACAA

ATACAAATACATACTAAGGGTTTCTTATATGCTCAACACATGAGCGAAACCCTATAGGAACCCTAATTCC

CTTATCTGGGAACTACTCACACATTATTATGGAGAAACTCGAAGATCCGTCGAGCTTGTCGATCGACAGA

TCCGGTCGGCATCTACTCTATTTCTTTGCCCTCGGACGAGTGCTGGGGCGTCGGTTTCCACTATCGGCGA

GTACTTCTACACAGCCATCGGTCCAGACGGCCGCGCTTCTGCGGGCGATTTGTGTACGCCCGACAGTCCC

GGCTCCGGATCGGACGATTGCGTCGCATCGACCCTGCGCCCAAGCTGCATCATCGAAATTGCCGTCAACC

AAGCTCTGATAGAGTTGGTCAAGACCAATGCGGAGCATATACGCCCGGAGTCGTGGCGATCCTGCAAGCT

CCGGATGCCTCCGCTCGAAGTAGCGCGTCTGCTGCTCCATACAAGCCAACCACGGCCTCCAGAAGAAGAT

GTTGGCGACCTCGTATTGGGAATCCCCGAACATCGCCTCGCTCCAGTCAATGACCGCTGTTATGCGGCCA

TTGTCCGTCAGGACATTGTTGGAGCCGAAATCCGCGTGCACGAGGTGCCGGACTTCGGGGCAGTCCTCGG

CCCAAAGCATCAGCTCATCGAGAGCCTGCGCGACGGACGCACTGACGGTGTCGTCCATCACAGTTTGCCA

GTGATACACATGGGGATCAGCAATCGCGCATATGAAATCACGCCATGTAGTGTATTGACCGATTCCTTGC

GGTCCGAATGGGCCGAACCCGCTCGTCTGGCTAAGATCGGCCGCAGCGATCGCATCCATGGCCTCCGCGA

CCGGCTGCAGTTATCATCATCATCATAGACACACGAAATAAAGTAATCAGATTATCAGTTAAAGCTATGT

AATATTTACACCATAACCAATCAATTAAAAAATAGATCAGTTTAAAGAAAGATCAAAGCTCAAAAAAATA

AAAAGAGAAAAGGGTCCTAACCAAGAAAATGAAGGAGAAAAACTAGAAATTTACCTGCAGAACAGCGGGC

AGTTCGGTTTCAGGCAGGTCTTGCAACGTGACACCCTGTGCACGGCGGGAGATGCAATAGGTCAGGCTCT

CGCTAAACTCCCCAATGTCAAGCACTTCCGGAATCGGGAGCGCGGCCGATGCAAAGTGCCGATAAACATA

ACGATCTTTGTAGAAACCATCGGCGCAGCTATTTACCCGCAGGACATATCCACGCCCTCCTACATCGAAG

CTGAAAGCACGAGATTCTTCGCCCTCCGAGAGCTGCATCAGGTCGGAGACGCTGTCGAACTTTTCGATCA

GAAACTTCTCGACAGACGTCGCGGTGAGTTCAGGCTTTTTCATATCTCATTGCCCCCCCGGATCTGCGAA

AGCTCGACGGATCTTCGAGAGAGATAGATTTGTAGAGAGAGACTGGTGATTTCAGCGTGTCCTCTCCAAA

TGAAATGAACTTCCTTATATAGAGGAAGGTCTTGCGAAGGATAGTGGGATTGTGCGTCATCCCTTACGTC

AGTGGAGATATCACATCAATCCACTTGCTTTGAAGACGTGGTTGGAACGTCTTCTTTTTCCACGATGCTC

CTCGTGGGTGGGGGTCCATCTTTGGGACCACTGTCGGCAGAGGCATCTTGAACGATAGCCTTTCCTTTAT

CGCAATGATGGCATTTGTAGGTGCCACCTTCCTTTTCTACTGTCCTTTTGATGAAGTGACAGATAGCTGG

GCAATGGAATCCGAGGAGGTTTCCCGATATTACCCTTTGTTGAAAAGTCTCAATAGCCCTTTGGTCTTCT

GAGACTGTATCTTTGATATTCTTGGAGTAGACGAGAGTGTCGTGCTCCACCATGTTATCACATCAATCCA

CTTGCTTTGAAGACGTGGTTGGAACGTCTTCTTTTTCCACGATGCTCCTCGTGGGTGGGGGTCCATCTTT

GGGACCACTGTCGGCAGAGGCATCTTGAACGATAGCCTTTCCTTTATCGCAATGATGGCATTTGTAGGTG

CCACCTTCCTTTTCTACTGTCCTTTTGATGAAGTGACAGATAGCTGGGCAATGGAATCCGAGGAGGTTTC

CCGATATTACCCTTTGTTGAAAAGTCTCAATAGCCCTTTGGTCTTCTGAGACTGTATCTTTGATATTCTT

GGAGTAGACGAGAGTGTCGTGCTCCACCATGTTGGCAAGCTGCTCTAGCCAATACGCAAACCGCCTCTCC

CCGCGCGTTGGCCGATTCATTAATGCAGCTGGCACGACAGGTTTCCCGACTGGAAAGCGGGCAGTGAGCG

CAACGCAATTAATGTGAGTTAGCTCACTCATTAGGCACCCCAGGCTTTACACTTTATGCTTCCGGCTCGT

ATGTTGTGTGGAATTGTGAGCGGATAACAATTTCACACAGGAAACAGCTATGACCATGATTAC

>gi|11559666|gb|AF294980.1| Binary vector pINDEX2, complete sequence

GAATTCGAGCTCGGTACCCGGGGATCCTCTAGAATCCGAAAAGTTTCTGCACCGTTTTCACCCCCTAACT

AACAATATAGGGAACGTGTGCTAAATATAAAATGAGACCTTATATATGTAGCGCTGATAACTAGAACTAT

GCAAGAAAAACTCATCCACCTACTTTAGTGGCAATCGGGCTAAATAAAAAAGAGTCGCTACACTAGCTAG

TTTCGTTTTCCTTAGTAATTAAGTGGGAAAATGAAATCATTATTGCTTAGAATATACGTTCACATCTCTG

TCATGAAGTTAAATTATTCGAGGTAGCCATAATTGTCATCAAACTCTTCTTGAATAAAAAAATCTTTCTA

GCTGAACTCAATGGGTAAAGAGAGAGATTTTTTTTAAAAAAATAGAATGAAGATATTCTGAACGTATTGG

CAAAGATTTAAACATATAATTATATAATTTTATAGTTTGTGCATTCGTCATATCGCACATCATTAAGGAC

ATGTCTTACTCCATCCCAATTTTTATTTAGTAATTAAAGACAATTGACTTATTTTTATTATTTATCTTTT

TTCGATTAGATGCAAGGTACTTACGCACACACTTTGTGCTCATGTGCATGTGTGAGTGCACCTCCTCAAT

ACACGTTCAACTAGCAACACATCTCTAATATCACTCGCCTATTTAATACATTTAGGTAGCAATATCTGAA

TTCAAGCACTCCACCATCACCAGACCACTTTTAATAATATCTAAAATACAAAAAATAATTTTACAGAATA

GCATGAAAAGTATGAAACGAACTATTTAGGTTTTTCACATACAAAAAAAAAAAGAATTTTGCTCGTGCGC

GAGCGCCAATCTCCCATATTGGGCACACAGGCAACAACAGAGTGGCTGCCCACAGAACAACCCACAAAAA

ACGATGATCTAACGGAGGACAGCAAGTCCGCAACAACCTTTTAACAGCAGGCTTTGCGGCCAGGAGAGAG

GAGGAGAGGCAAAGAAAACCAAGCATCCTCCTCCTCCCATCTATAAATTCCTCCCCCCTTTTCCCCTCTC

TATATAGGAGGCATCCAAGCCAAGAAGAGGGAGAGCACCAAGGACACGCGACTAGCAGAAGCCGAGCGAC

CGCCTTCTTCGATCCATATCTTCCGGTCGAGTTCTTGGTCGATCTCTTCCCTCCTCCACCTCCTCCTCAC

AGGGTATGTGCCCTTCGGTTGTTCTTGGATTTATTGTTCTAGGTTGTGTAGTACGGGCGTTGATGTTAGG

AAAGGGGATCTGTATCTGTGATGATTCCTGTTCTTGGATTTGGGATAGAGGGGTTCTTGATGTTGCATGT

TATCGGTTCGGTTTGATTAGTAGTATGGTTTTCAATCGTCTGGAGAGCTCTATGGAAATGAAATGGTTTA

GGGTACGGAATCTTGCGATTTTGTGAGTACCTTTTGTTTGAGGTAAAATCAGAGCACCGGTGATTTTGCT

TGGTGTAATAAAAGTACGGTTGTTTGGTCCTCGATTCTGGTAGTGATGCTTCTCGATTTGACGAAGCTAT

CCTTTGTTTATTCCCTATTGAACAAAAATAATCCAACTTTGAAGACGGTCCCGTTGATGAGATTGAATGA

TTGATTCTTAAGCCTGTCCAAAATTTCGCAGCTGGCTTGTTTAGATACAGTAGTCCCCATCACGAAATTC

ATGGAAACAGTTATAATCCTCAGGAACAGGGGATTCCCTGTTCTTCCGATTTGCTTTAGTCCCAGAATTT

TTTTTCCCAAATATCTTAAAAAGTCACTTTCTGGTTCAGTTCAATGAATTGATTGCTACAAATAATGCTT

TTATAGCGTTATCCTAGCTGTAGTTCAGTTAATAGGTAATACCCCTATAGTTTAGTCAGGAGAAGAACTT

ATCCGATTTCTGATCTCCATTTTTAATTATATGAAATGAACTGTAGCATAAGCAGTATTCATTTGGATTA

TTTTTTTTATTAGCTCTCACCCCTTCATTATTCTGAGCTGAAAGTCTGGCATGAACTGTCCTCAATTTTG

TTTTCAAATTCACATCGATTATCTATCGATTATCCTCTTGTATCTACCTGTAGAAGTTTCTTTTTGGTTA

TTCCTTGACTGCTTGATTACAGAAAGAAATTTATGAAGCTGTAATCGGGATAGTTATACTGCTTGTTCTT

ATGATTCATTTCCTTTGTGCAGTTCTTGGTGTAGCTTGCCACTTTCACCAGCAAAGTTCCATAAAACAAT

GGCAGATCCAATGAAGCTACTGTCTTCTATCGAACAAGCATGCGATATTTGCCGACTTAAAAAGCTCAAG

TGCTCCAAAGAAAAACCGAAGTGCGCCAAGTGTCTGAAGAACAACTGGGAGTGTCGCTACTCTCCCAAAA

CCAAAAGGTCTCCGCTGACTAGGGCACATCTGACAGAAGTGGAATCAAGGCTAGAAAGACTGGAACAGCT

ATTTCTACTGATTTTTCCTAGGTCGAGCGCCCCCCCGACCGATGTCAGCCTGGGGGACGAGCTCCACTTA

GACGGCGAGGACGTGGCGATGGCGCATGCCGACGCGCTAGACGATTTCGATCTGGACATGTTGGGGGACG

GGGATTCCCCGGGTCCGGGATTTACCCCCCACGACTCCGCCCCCTACGGCGCTCTGGATATGGCCGACTT

CGAGTTTGAGCAGATGTTTACCGATGCCCTTGGAATTGACGAGTACGGTGGGGATCCAATTCAGCAAGCC

ACTGCAGGAGTCTCACAAGACACTTCGGAAAATCCTAACAAAACAATAGTTCCTGCTGCATTACCACAGC

TCACCCCTACCTTGGTGTCACTGCTGGAGGTGATTGAACCCGAGGTGTTGTATGCAGGATATGATAGCTC

TGTTCCAGATTCAGCATGGAGAATTATGACCACACTCAACATGTTAGGTGGGCGTCAAGTGATTGCAGCA

GTGAAATGGGCAAAGGCGATACCAGGCTTCAGAAACTTACACCTGGATGACCAAATGACCCTGCTACAGT

ACTCATGGATGTTTCTCATGGCATTTGCCCTGGGTTGGAGATCATACAGACAATCAAGTGGAAACCTGCT

CTGCTTTGCTCCTGATCTGATTATTAATGAGCAGAGAATGTCTCTACCCTGCATGTATGACCAATGTAAA

CACATGCTGTTTGTCTCCTCTGAATTACAAAGATTGCAGGTATCCTATGAAGAGTATCTCTGTATGAAAA

CCTTACTGCTTCTCTCCTCAGTTCCTAAGGAAGGTCTGAAGAGCCAAGAGTTATTTGATGAGATTCGAAT

GACTTATATCAAAGAGCTAGGAAAAGCCATCGTCAAAAGGGAAGGGAACTCCAGTCAGAACTGGCAACGG

TTTTACCAACTGACAAAGCTTCTGGACTCCATGCATGAGGTGGTTGAGAATCTCCTTACCTACTGCTTCC

AGACATTTTTGGATAAGACCATGAGTATTGAATTCCCAGAGATGTTAGCTGAAATCATCACTAATCAGAT

ACCAAAATATTCAAATGGAAATATCAAAAAGCTTCTGTTTCATCAAAAATGACTCGACCTAACTGAGTAA

GCTAGCTTGTTCGAGTATTATGGCATTGGGAAAACTGTTTTTCTTGTACCATTTGTTGTGCTTGTAATTT

ACTGTGTTTTTTATTCGGTTTTCGCTATCGAACTGTGAAATGGAAATGGATGGAGAAGAGTTAATGAATG

ATATGGTCCTTTTGTTCATTCTCAAATTAATATTATTTGTTTTTTCTCTTATTTGTTGTGTGTTGAATTT

GAAATTATAAGAGATATGCAAACATTTTGTTTTGAGTAAAAATGTGTCAAATCGTGGCCTCTAATGACCG

AAGTTAATATGAGGAGTAAAACACTAGATCCCCAAACAAGCTTGAAACTGAAGGCGGGAAACGACAATCT

GATCATGGGCGGCCGTCTGGACCGATGGCTGTGTAGAAGTACTCGCCGATAGTGGAAACCGACGCCCCAG

CACTCGTCCGGGATCTTGGAGGTGATGTAACATGATCACAAGCTGATCCCCCGAATTTCCCCGATCGTTC

AAACATTTGGCAATAAAGTTTCTTAAGATTGAATCCTGTTGCCGGTCTTGCGATGATTATCATATAATTT

CTGTTGAATTACGTTAAGCATGTAATAATTAACATGTAATGCATGACGTTATTTATGAGATGGGTTTTTA

TGATTAGAGTCCCGCAATTATACATTTAATACGCGATAGAAAACAAAATATAGCGCGCAAACTAGGATAA

ATTATCGCGCGCGGTGTCATCTATGTTACTAGATCGGGAATTGATCCCCCCTCGACAGCTTGCATGCCGG

TCGACTCTAGAGGATCCGGGTGACAGCCCTCCGACGGGTGACAGCCCTCCGACGGGTGACAGCCCTCCGA

CGGGTGACAGCCCTCCGAATTCGAGCTCGGTACCCGGGGATCTGTCGACCTCGATCGAGATCTTCGCAAG

ACCCTTCCTCTATATAAGGAAGTTCATTTCATTTGGAGAGGACACGCTGAAGCTAGTCGACTCTAGCCTC

GACACGTGTAACTAGTTACCTGCAGGTACTCGAGTATGTACATCTAGTCGATCCAGGCCTCCCAGCTTTC

GTCCGTATCATCGGTTTCGACAACGTTCGTCAAGTTCAATGCATCAGTTTCATTGCCCACACACCAGAAT

CCTACTAAGTTTGAGTATTATGGCATTGGAAAAGCTGTTTTCTTCTATCATTTGTTCTGCTTGTAATTTA

CTGTGTTCTTTCAGTTTTTGTTTTCGGACATCAAAATGCAAATGGATGGATAAGAGTTAATAAATGATAT

GGTCCTTTTGTTCATTCTCAAATTATTATTATCTGTTGTTTTTACTTTAATGGGTTGAATTTAAGTAAGA

AAGGAACTAACAGTGTGATATTAAGGTGCAATGTTAGACATATAAAACAGTCTTTCACCTCTCTTTGGTT

ATGTCTTGAATTGGTTTGTTTCTTCACTTATCTGTGTAATCAAGTTTACTATGAGTCTATGATCAAGTAA

TTATGCAATCAAGTTAAGTACAGTATAGGCTTTTTGTGTCGAGGGGGTACCGAGTCGAGGAATTCGATAT

CAAGCTTGGCACTGGCCGTCGTTTTACAACGTCGTGACTGGGAAAACCCTGGCGTTACCCAACTTAATCG

CCTTGCAGCACATCCCCCTTTCGCCAGCTGGCGTAATAGCGAAGAGGCCCGCACCGATCGCCCTTCCCAA

CAGTTGCGCAGCCTGAATGGCGAATGCTAGAGCAGCTTGAGCTTGGATCAGATTGTCGTTTCCCGCCTTC

AGTTTAAACTATCAGTGTTTGACAGGATATATTGGCGGGTAAACCTAAGAGAAAAGAGCGTTTATTAGAA

TAACGGATATTTAAAAGGGCGTGAAAAGGTTTATCCGTTCGTCCATTTGTATGTGCATGCCAACCACAGG

GTTCCCCTCGGGATCAAAGTACTTTGATCCAACCCCTCCGCTGCTATAGTGCAGTCGGCTTCTGACGTTC

AGTGCAGCCGTCTTCTGAAAACGACATGTCGCACAAGTCCTAAGTTACGCGACAGGCTGCCGCCCTGCCC

TTTTCCTGGCGTTTTCTTGTCGCGTGTTTTAGTCGCATAAAGTAGAATACTTGCGACTAGAACCGGAGAC

ATTACGCCATGAACAAGAGCGCCGCCGCTGGCCTGCTGGGCTATGCCCGCGTCAGCACCGACGACCAGGA

CTTGACCAACCAACGGGCCGAACTGCACGCGGCCGGCTGCACCAAGCTGTTTTCCGAGAAGATCACCGGC

ACCAGGCGCGACCGCCCGGAGCTGGCCAGGATGCTTGACCACCTACGCCCTGGCGACGTTGTGACAGTGA

CCAGGCTAGACCGCCTGGCCCGCAGCACCCGCGACCTACTGGACATTGCCGAGCGCATCCAGGAGGCCGG

CGCGGGCCTGCGTAGCCTGGCAGAGCCGTGGGCCGACACCACCACGCCGGCCGGCCGCATGGTGTTGACC

GTGTTCGCCGGCATTGCCGAGTTCGAGCGTTCCCTAATCATCGACCGCACCCGGAGCGGGCGCGAGGCCG

CCAAGGCCCGAGGCGTGAAGTTTGGCCCCCGCCCTACCCTCACCCCGGCACAGATCGCGCACGCCCGCGA

GCTGATCGACCAGGAAGGCCGCACCGTGAAAGAGGCGGCTGCACTGCTTGGCGTGCATCGCTCGACCCTG

TACCGCGCACTTGAGCGCAGCGAGGAAGTGACGCCCACCGAGGCCAGGCGGCGCGGTGCCTTCCGTGAGG

ACGCATTGACCGAGGCCGACGCCCTGGCGGCCGCCGAGAATGAACGCCAAGAGGAACAAGCATGAAACCG

CACCAGGACGGCCAGGACGAACCGTTTTTCATTACCGAAGAGATCGAGGCGGAGATGATCGCGGCCGGGT

ACGTGTTCGAGCCGCCCGCGCACGTCTCAACCGTGCGGCTGCATGAAATCCTGGCCGGTTTGTCTGATGC

CAAGCTGGCGGCCTGGCCGGCCAGCTTGGCCGCTGAAGAAACCGAGCGCCGCCGTCTAAAAAGGTGATGT

GTATTTGAGTAAAACAGCTTGCGTCATGCGGTCGCTGCGTATATGATGCGATGAGTAAATAAACAAATAC

GCAAGGGGAACGCATGAAGGTTATCGCTGTACTTAACCAGAAAGGCGGGTCAGGCAAGACGACCATCGCA

ACCCATCTAGCCCGCGCCCTGCAACTCGCCGGGGCCGATGTTCTGTTAGTCGATTCCGATCCCCAGGGCA

GTGCCCGCGATTGGGCGGCCGTGCGGGAAGATCAACCGCTAACCGTTGTCGGCATCGACCGCCCGACGAT

TGACCGCGACGTGAAGGCCATCGGCCGGCGCGACTTCGTAGTGATCGACGGAGCGCCCCAGGCGGCGGAC

TTGGCTGTGTCCGCGATCAAGGCAGCCGACTTCGTGCTGATTCCGGTGCAGCCAAGCCCTTACGACATAT

GGGCCACCGCCGACCTGGTGGAGCTGGTTAAGCAGCGCATTGAGGTCACGGATGGAAGGCTACAAGCGGC

CTTTGTCGTGTCGCGGGCGATCAAAGGCACGCGCATCGGCGGTGAGGTTGCCGAGGCGCTGGCCGGGTAC

GAGCTGCCCATTCTTGAGTCCCGTATCACGCAGCGCGTGAGCTACCCAGGCACTGCCGCCGCCGGCACAA

CCGTTCTTGAATCAGAACCCGAGGGCGACGCTGCCCGCGAGGTCCAGGCGCTGGCCGCTGAAATTAAATC

AAAACTCATTTGAGTTAATGAGGTAAAGAGAAAATGAGCAAAAGCACAAACACGCTAAGTGCCGGCCGTC

CGAGCGCACGCAGCAGCAAGGCTGCAACGTTGGCCAGCCTGGCAGACACGCCAGCCATGAAGCGGGTCAA

CTTTCAGTTGCCGGCGGAGGATCACACCAAGCTGAAGATGTACGCGGTACGCCAAGGCAAGACCATTACC

GAGCTGCTATCTGAATACATCGCGCAGCTACCAGAGTAAATGAGCAAATGAATAAATGAGTAGATGAATT

TTAGCGGCTAAAGGAGGCGGCATGGAAAATCAAGAACAACCAGGCACCGACGCCGTGGAATGCCCCATGT

GTGGAGGAACGGGCGGTTGGCCAGGCGTAAGCGGCTGGGTTGTCTGCCGGCCCTGCAATGGCACTGGAAC

CCCCAAGCCCGAGGAATCGGCGTGACGGTCGCAAACCATCCGGCCCGGTACAAATCGGCGCGGCGCTGGG

TGATGACCTGGTGGAGAAGTTGAAGGCCGCGCAGGCCGCCCAGCGGCAACGCATCGAGGCAGAAGCACGC

CCCGGTGAATCGTGGCAAGCGGCCGCTGATCGAATCCGCAAAGAATCCCGGCAACCGCCGGCAGCCGGTG

CGCCGTCGATTAGGAAGCCGCCCAAGGGCGACGAGCAACCAGATTTTTTCGTTCCGATGCTCTATGACGT

GGGCACCCGCGATAGTCGCAGCATCATGGACGTGGCCGTTTTCCGTCTGTCGAAGCGTGACCGACGAGCT

GGCGAGGTGATCCGCTACGAGCTTCCAGACGGGCACGTAGAGGTTTCCGCAGGGCCGGCCGGCATGGCCA

GTGTGTGGGATTACGACCTGGTACTGATGGCGGTTTCCCATCTAACCGAATCCATGAACCGATACCGGGA

AGGGAAGGGAGACAAGCCCGGCCGCGTGTTCCGTCCACACGTTGCGGACGTACTCAAGTTCTGCCGGCGA

GCCGATGGCGGAAAGCAGAAAGACGACCTGGTAGAAACCTGCATTCGGTTAAACACCACGCACGTTGCCA

TGCAGCGTACGAAGAAGGCCAAGAACGGCCGCCTGGTGACGGTATCCGAGGGTGAAGCCTTGATTAGCCG

CTACAAGATCGTAAAGAGCGAAACCGGGCGGCCGGAGTACATCGAGATCGAGCTAGCTGATTGGATGTAC

CGCGAGATCACAGAAGGCAAGAACCCGGACGTGCTGACGGTTCACCCCGATTACTTTTTGATCGATCCCG

GCATCGGCCGTTTTCTCTACCGCCTGGCACGCCGCGCCGCAGGCAAGGCAGAAGCCAGATGGTTGTTCAA

GACGATCTACGAACGCAGTGGCAGCGCCGGAGAGTTCAAGAAGTTCTGTTTCACCGTGCGCAAGCTGATC

GGGTCAAATGACCTGCCGGAGTACGATTTGAAGGAGGAGGCGGGGCAGGCTGGCCCGATCCTAGTCATGC

GCTACCGCAACCTGATCGAGGGCGAAGCATCCGCCGGTTCCTAATGTACGGAGCAGATGCTAGGGCAAAT

TGCCCTAGCAGGGGAAAAAGGTCGAAAAGGTCTCTTTCCTGTGGATAGCACGTACATTGGGAACCCAAAG

CCGTACATTGGGAACCGGAACCCGTACATTGGGAACCCAAAGCCGTACATTGGGAACCGGTCACACATGT

AAGTGACTGATATAAAAGAGAAAAAAGGCGATTTTTCCGCCTAAAACTCTTTAAAACTTATTAAAACTCT

TAAAACCCGCCTGGCCTGTGCATAACTGTCTGGCCAGCGCACAGCCGAAGAGCTGCAAAAAGCGCCTACC

CTTCGGTCGCTGCGCTCCCTACGCCCCGCCGCTTCGCGTCGGCCTATCGCGGCCGCTGGCCGCTCAAAAA

TGGCTGGCCTACGGCCAGGCAATCTACCAGGGCGCGGACAAGCCGCGCCGTCGCCACTCGACCGCCGGCG

CCCACATCAAGGCACCCTGCCTCGCGCGTTTCGGTGATGACGGTGAAAACCTCTGACACATGCAGCTCCC

GGAGACGGTCACAGCTTGTCTGTAAGCGGATGCCGGGAGCAGACAAGCCCGTCAGGGCGCGTCAGCGGGT

GTTGGCGGGTGTCGGGGCGCAGCCATGACCCAGTCACGTAGCGATAGCGGAGTGTATACTGGCTTAACTA

TGCGGCATCAGAGCAGATTGTACTGAGAGTGCACCATATGCGGTGTGAAATACCGCACAGATGCGTAAGG

AGAAAATACCGCATCAGGCGCTCTTCCGCTTCCTCGCTCACTGACTCGCTGCGCTCGGTCGTTCGGCTGC

GGCGAGCGGTATCAGCTCACTCAAAGGCGGTAATACGGTTATCCACAGAATCAGGGGATAACGCAGGAAA

GAACATGTGAGCAAAAGGCCAGCAAAAGGCCAGGAACCGTAAAAAGGCCGCGTTGCTGGCGTTTTTCCAT

AGGCTCCGCCCCCCTGACGAGCATCACAAAAATCGACGCTCAAGTCAGAGGTGGCGAAACCCGACAGGAC

TATAAAGATACCAGGCGTTTCCCCCTGGAAGCTCCCTCGTGCGCTCTCCTGTTCCGACCCTGCCGCTTAC

CGGATACCTGTCCGCCTTTCTCCCTTCGGGAAGCGTGGCGCTTTCTCATAGCTCACGCTGTAGGTATCTC

AGTTCGGTGTAGGTCGTTCGCTCCAAGCTGGGCTGTGTGCACGAACCCCCCGTTCAGCCCGACCGCTGCG

CCTTATCCGGTAACTATCGTCTTGAGTCCAACCCGGTAAGACACGACTTATCGCCACTGGCAGCAGCCAC

TGGTAACAGGATTAGCAGAGCGAGGTATGTAGGCGGTGCTACAGAGTTCTTGAAGTGGTGGCCTAACTAC

GGCTACACTAGAAGGACAGTATTTGGTATCTGCGCTCTGCTGAAGCCAGTTACCTTCGGAAAAAGAGTTG

GTAGCTCTTGATCCGGCAAACAAACCACCGCTGGTAGCGGTGGTTTTTTTGTTTGCAAGCAGCAGATTAC

GCGCAGAAAAAAAGGATCTCAAGAAGATCCTTTGATCTTTTCTACGGGGTCTGACGCTCAGTGGAACGAA

AACTCACGTTAAGGGATTTTGGTCATGCATTCTAGGTACTAAAACAATTCATCCAGTAAAATATAATATT

TTATTTTCTCCCAATCAGGCTTGATCCCCAGTAAGTCAAAAAATAGCTCGACATACTGTTCTTCCCCGAT

ATCCTCCCTGATCGACCGGACGCAGAAGGCAATGTCATACCACTTGTCCGCCCTGCCGCTTCTCCCAAGA

TCAATAAAGCCACTTACTTTGCCATCTTTCACAAAGATGTTGCTGTCTCCCAGGTCGCCGTGGGAAAAGA

CAAGTTCCTCTTCGGGCTTTTCCGTCTTTAAAAAATCATACAGCTCGCGCGGATCTTTAAATGGAGTGTC

TTCTTCCCAGTTTTCGCAATCCACATCGGCCAGATCGTTATTCAGTAAGTAATCCAATTCGGCTAAGCGG

CTGTCTAAGCTATTCGTATAGGGACAATCCGATATGTCGATGGAGTGAAAGAGCCTGATGCACTCCGCAT

ACAGCTCGATAATCTTTTCAGGGCTTTGTTCATCTTCATACTCTTCCGAGCAAAGGACGCCATCGGCCTC

ACTCATGAGCAGATTGCTCCAGCCATCATGCCGTTCAAAGTGCAGGACCTTTGGAACAGGCAGCTTTCCT

TCCAGCCATAGCATCATGTCCTTTTCCCGTTCCACATCATAGGTGGTCCCTTTATACCGGCTGTCCGTCA

TTTTTAAATATAGGTTTTCATTTTCTCCCACCAGCTTATATACCTTAGCAGGAGACATTCCTTCCGTATC

TTTTACGCAGCGGTATTTTTCGATCAGTTTTTTCAATTCCGGTGATATTCTCATTTTAGCCATTTATTAT

TTCCTTCCTCTTTTCTACAGTATTTAAAGATACCCCAAGAAGCTAATTATAACAAGACGAACTCCAATTC

ACTGTTCCTTGCATTCTAAAACCTTAAATACCAGAAAACAGCTTTTTCAAAGTTGTTTTCAAAGTTGGCG

TATAACATAGTATCGACGGAGCCGATTTTGAAACCGCGGTGATCACAGGCAGCAACGCTCTGTCATCGTT

ACAATCAACATGCTACCCTCCGCGAGATCATCCGTGTTTCAAACCCGGCAGCTTAGTTGCCGTTCTTCCG

AATAGCATCGGTAACATGAGCAAAGTCTGCCGCCTTACAACGGCTCTCCCGCTGACGCCGTCCCGGACTG

ATGGGCTGCCTGTATCGAGTGGTGATTTTGTGCCGAGCTGCCGGTCGGGGAGCTGTTGGCTGGCTGGTGG

CAGGATATATTGTGGTGTAAACAAATTGACGCTTAGACAACTTAATAACACATTGCGGACGTTTTTAATG

TACTGAATTAACGCCGAATTAATTCGGGGGATCTGGATTTTAGTACTGGATTTTGGTTTTAGGAATTAGA

AATTTTATTGATAGAAGTATTTTACAAATACAAATACATACTAAGGGTTTCTTATATGCTCAACACATGA

GCGAAACCCTATAGGAACCCTAATTCCCTTATCTGGGAACTACTCACACATTATTATGGAGAAACTCGAA

GATCCGTCGAGCTTGTCGATCGACAGATCCGGTCGGCATCTACTCTATTTCTTTGCCCTCGGACGAGTGC

TGGGGCGTCGGTTTCCACTATCGGCGAGTACTTCTACACAGCCATCGGTCCAGACGGCCGCGCTTCTGCG

GGCGATTTGTGTACGCCCGACAGTCCCGGCTCCGGATCGGACGATTGCGTCGCATCGACCCTGCGCCCAA

GCTGCATCATCGAAATTGCCGTCAACCAAGCTCTGATAGAGTTGGTCAAGACCAATGCGGAGCATATACG

CCCGGAGTCGTGGCGATCCTGCAAGCTCCGGATGCCTCCGCTCGAAGTAGCGCGTCTGCTGCTCCATACA

AGCCAACCACGGCCTCCAGAAGAAGATGTTGGCGACCTCGTATTGGGAATCCCCGAACATCGCCTCGCTC

CAGTCAATGACCGCTGTTATGCGGCCATTGTCCGTCAGGACATTGTTGGAGCCGAAATCCGCGTGCACGA

GGTGCCGGACTTCGGGGCAGTCCTCGGCCCAAAGCATCAGCTCATCGAGAGCCTGCGCGACGGACGCACT

GACGGTGTCGTCCATCACAGTTTGCCAGTGATACACATGGGGATCAGCAATCGCGCATATGAAATCACGC

CATGTAGTGTATTGACCGATTCCTTGCGGTCCGAATGGGCCGAACCCGCTCGTCTGGCTAAGATCGGCCG

CAGCGATCGCATCCATGGCCTCCGCGACCGGCTGCAGTTATCATCATCATCATAGACACACGAAATAAAG

TAATCAGATTATCAGTTAAAGCTATGTAATATTTACACCATAACCAATCAATTAAAAAATAGATCAGTTT

AAAGAAAGATCAAAGCTCAAAAAAATAAAAAGAGAAAAGGGTCCTAACCAAGAAAATGAAGGAGAAAAAC

TAGAAATTTACCTGCAGAACAGCGGGCAGTTCGGTTTCAGGCAGGTCTTGCAACGTGACACCCTGTGCAC

GGCGGGAGATGCAATAGGTCAGGCTCTCGCTAAACTCCCCAATGTCAAGCACTTCCGGAATCGGGAGCGC

GGCCGATGCAAAGTGCCGATAAACATAACGATCTTTGTAGAAACCATCGGCGCAGCTATTTACCCGCAGG

ACATATCCACGCCCTCCTACATCGAAGCTGAAAGCACGAGATTCTTCGCCCTCCGAGAGCTGCATCAGGT

CGGAGACGCTGTCGAACTTTTCGATCAGAAACTTCTCGACAGACGTCGCGGTGAGTTCAGGCTTTTTCAT

ATCTCATTGCCCCCCCGGATCTGCGAAAGCTCGACGGATCTTCGAGAGAGATAGATTTGTAGAGAGAGAC

TGGTGATTTCAGCGTGTCCTCTCCAAATGAAATGAACTTCCTTATATAGAGGAAGGTCTTGCGAAGGATA

GTGGGATTGTGCGTCATCCCTTACGTCAGTGGAGATATCACATCAATCCACTTGCTTTGAAGACGTGGTT

GGAACGTCTTCTTTTTCCACGATGCTCCTCGTGGGTGGGGGTCCATCTTTGGGACCACTGTCGGCAGAGG

CATCTTGAACGATAGCCTTTCCTTTATCGCAATGATGGCATTTGTAGGTGCCACCTTCCTTTTCTACTGT

CCTTTTGATGAAGTGACAGATAGCTGGGCAATGGAATCCGAGGAGGTTTCCCGATATTACCCTTTGTTGA

AAAGTCTCAATAGCCCTTTGGTCTTCTGAGACTGTATCTTTGATATTCTTGGAGTAGACGAGAGTGTCGT

GCTCCACCATGTTATCACATCAATCCACTTGCTTTGAAGACGTGGTTGGAACGTCTTCTTTTTCCACGAT

GCTCCTCGTGGGTGGGGGTCCATCTTTGGGACCACTGTCGGCAGAGGCATCTTGAACGATAGCCTTTCCT

TTATCGCAATGATGGCATTTGTAGGTGCCACCTTCCTTTTCTACTGTCCTTTTGATGAAGTGACAGATAG

CTGGGCAATGGAATCCGAGGAGGTTTCCCGATATTACCCTTTGTTGAAAAGTCTCAATAGCCCTTTGGTC

TTCTGAGACTGTATCTTTGATATTCTTGGAGTAGACGAGAGTGTCGTGCTCCACCATGTTGGCAAGCTGC

TCTAGCCAATACGCAAACCGCCTCTCCCCGCGCGTTGGCCGATTCATTAATGCAGCTGGCACGACAGGTT

TCCCGACTGGAAAGCGGGCAGTGAGCGCAACGCAATTAATGTGAGTTAGCTCACTCATTAGGCACCCCAG

GCTTTACACTTTATGCTTCCGGCTCGTATGTTGTGTGGAATTGTGAGCGGATAACAATTTCACACAGGAA

ACAGCTATGACCATGATTAC

>gi|11559669|gb|AF294981.1| Binary vector pINDEX4, complete sequence

GAATTCGAGCTCGGTACCCGGGGATCCTCTAGATAGTTAACTAGGGCCCTACAATTGTAACGCGTTAATT

TAAATAAACAATGGCAGATCCAATGAAGCTACTGTCTTCTATCGAACAAGCATGCGATATTTGCCGACTT

AAAAAGCTCAAGTGCTCCAAAGAAAAACCGAAGTGCGCCAAGTGTCTGAAGAACAACTGGGAGTGTCGCT

ACTCTCCCAAAACCAAAAGGTCTCCGCTGACTAGGGCACATCTGACAGAAGTGGAATCAAGGCTAGAAAG

ACTGGAACAGCTATTTCTACTGATTTTTCCTAGGTCGAGCGCCCCCCCGACCGATGTCAGCCTGGGGGAC

GAGCTCCACTTAGACGGCGAGGACGTGGCGATGGCGCATGCCGACGCGCTAGACGATTTCGATCTGGACA

TGTTGGGGGACGGGGATTCCCCGGGTCCGGGATTTACCCCCCACGACTCCGCCCCCTACGGCGCTCTGGA

TATGGCCGACTTCGAGTTTGAGCAGATGTTTACCGATGCCCTTGGAATTGACGAGTACGGTGGGGATCCA

ATTCAGCAAGCCACTGCAGGAGTCTCACAAGACACTTCGGAAAATCCTAACAAAACAATAGTTCCTGCTG

CATTACCACAGCTCACCCCTACCTTGGTGTCACTGCTGGAGGTGATTGAACCCGAGGTGTTGTATGCAGG

ATATGATAGCTCTGTTCCAGATTCAGCATGGAGAATTATGACCACACTCAACATGTTAGGTGGGCGTCAA

GTGATTGCAGCAGTGAAATGGGCAAAGGCGATACCAGGCTTCAGAAACTTACACCTGGATGACCAAATGA

CCCTGCTACAGTACTCATGGATGTTTCTCATGGCATTTGCCCTGGGTTGGAGATCATACAGACAATCAAG

TGGAAACCTGCTCTGCTTTGCTCCTGATCTGATTATTAATGAGCAGAGAATGTCTCTACCCTGCATGTAT

GACCAATGTAAACACATGCTGTTTGTCTCCTCTGAATTACAAAGATTGCAGGTATCCTATGAAGAGTATC

TCTGTATGAAAACCTTACTGCTTCTCTCCTCAGTTCCTAAGGAAGGTCTGAAGAGCCAAGAGTTATTTGA

TGAGATTCGAATGACTTATATCAAAGAGCTAGGAAAAGCCATCGTCAAAAGGGAAGGGAACTCCAGTCAG

AACTGGCAACGGTTTTACCAACTGACAAAGCTTCTGGACTCCATGCATGAGGTGGTTGAGAATCTCCTTA

CCTACTGCTTCCAGACATTTTTGGATAAGACCATGAGTATTGAATTCCCAGAGATGTTAGCTGAAATCAT

CACTAATCAGATACCAAAATATTCAAATGGAAATATCAAAAAGCTTCTGTTTCATCAAAAATGACTCGAC

CTAACTGAGTAAGCTAGCTTGTTCGAGTATTATGGCATTGGGAAAACTGTTTTTCTTGTACCATTTGTTG

TGCTTGTAATTTACTGTGTTTTTTATTCGGTTTTCGCTATCGAACTGTGAAATGGAAATGGATGGAGAAG

AGTTAATGAATGATATGGTCCTTTTGTTCATTCTCAAATTAATATTATTTGTTTTTTCTCTTATTTGTTG

TGTGTTGAATTTGAAATTATAAGAGATATGCAAACATTTTGTTTTGAGTAAAAATGTGTCAAATCGTGGC

CTCTAATGACCGAAGTTAATATGAGGAGTAAAACACTAGATCCCCAAACAAGCTTGAAACTGAAGGCGGG

AAACGACAATCTGATCATGGGCGGCCGTCTGGACCGATGGCTGTGTAGAAGTACTCGCCGATAGTGGAAA

CCGACGCCCCAGCACTCGTCCGGGATCTTGGAGGTGATGTAACATGATCACAAGCTGATCCCCCGAATTT

CCCCGATCGTTCAAACATTTGGCAATAAAGTTTCTTAAGATTGAATCCTGTTGCCGGTCTTGCGATGATT

ATCATATAATTTCTGTTGAATTACGTTAAGCATGTAATAATTAACATGTAATGCATGACGTTATTTATGA

GATGGGTTTTTATGATTAGAGTCCCGCAATTATACATTTAATACGCGATAGAAAACAAAATATAGCGCGC

AAACTAGGATAAATTATCGCGCGCGGTGTCATCTATGTTACTAGATCGGGAATTGATCCCCCCTCGACAG

CTTGCATGCCGGTCGACTCTAGAGGATCCGGGTGACAGCCCTCCGACGGGTGACAGCCCTCCGACGGGTG

ACAGCCCTCCGACGGGTGACAGCCCTCCGAATTCGAGCTCGGTACCCGGGGATCTGTCGACCTCGATCGA

GATCTTCGCAAGACCCTTCCTCTATATAAGGAAGTTCATTTCATTTGGAGAGGACACGCTGAAGCTAGTC

GACTCTAGCCTCGACACGTGTAACTAGTTACCTGCAGGTACTCGAGTATGTACATCTAGTCGATCCAGGC

CTCCCAGCTTTCGTCCGTATCATCGGTTTCGACAACGTTCGTCAAGTTCAATGCATCAGTTTCATTGCCC

ACACACCAGAATCCTACTAAGTTTGAGTATTATGGCATTGGAAAAGCTGTTTTCTTCTATCATTTGTTCT

GCTTGTAATTTACTGTGTTCTTTCAGTTTTTGTTTTCGGACATCAAAATGCAAATGGATGGATAAGAGTT

AATAAATGATATGGTCCTTTTGTTCATTCTCAAATTATTATTATCTGTTGTTTTTACTTTAATGGGTTGA

ATTTAAGTAAGAAAGGAACTAACAGTGTGATATTAAGGTGCAATGTTAGACATATAAAACAGTCTTTCAC

CTCTCTTTGGTTATGTCTTGAATTGGTTTGTTTCTTCACTTATCTGTGTAATCAAGTTTACTATGAGTCT

ATGATCAAGTAATTATGCAATCAAGTTAAGTACAGTATAGGCTTTTTGTGTCGAGGGGGTACCGAGTCGA

GGAATTCGATATCAAGCTTGGCACTGGCCGTCGTTTTACAACGTCGTGACTGGGAAAACCCTGGCGTTAC

CCAACTTAATCGCCTTGCAGCACATCCCCCTTTCGCCAGCTGGCGTAATAGCGAAGAGGCCCGCACCGAT

CGCCCTTCCCAACAGTTGCGCAGCCTGAATGGCGAATGCTAGAGCAGCTTGAGCTTGGATCAGATTGTCG

TTTCCCGCCTTCAGTTTAAACTATCAGTGTTTGACAGGATATATTGGCGGGTAAACCTAAGAGAAAAGAG

CGTTTATTAGAATAACGGATATTTAAAAGGGCGTGAAAAGGTTTATCCGTTCGTCCATTTGTATGTGCAT

GCCAACCACAGGGTTCCCCTCGGGATCAAAGTACTTTGATCCAACCCCTCCGCTGCTATAGTGCAGTCGG

CTTCTGACGTTCAGTGCAGCCGTCTTCTGAAAACGACATGTCGCACAAGTCCTAAGTTACGCGACAGGCT

GCCGCCCTGCCCTTTTCCTGGCGTTTTCTTGTCGCGTGTTTTAGTCGCATAAAGTAGAATACTTGCGACT

AGAACCGGAGACATTACGCCATGAACAAGAGCGCCGCCGCTGGCCTGCTGGGCTATGCCCGCGTCAGCAC

CGACGACCAGGACTTGACCAACCAACGGGCCGAACTGCACGCGGCCGGCTGCACCAAGCTGTTTTCCGAG

AAGATCACCGGCACCAGGCGCGACCGCCCGGAGCTGGCCAGGATGCTTGACCACCTACGCCCTGGCGACG

TTGTGACAGTGACCAGGCTAGACCGCCTGGCCCGCAGCACCCGCGACCTACTGGACATTGCCGAGCGCAT

CCAGGAGGCCGGCGCGGGCCTGCGTAGCCTGGCAGAGCCGTGGGCCGACACCACCACGCCGGCCGGCCGC

ATGGTGTTGACCGTGTTCGCCGGCATTGCCGAGTTCGAGCGTTCCCTAATCATCGACCGCACCCGGAGCG

GGCGCGAGGCCGCCAAGGCCCGAGGCGTGAAGTTTGGCCCCCGCCCTACCCTCACCCCGGCACAGATCGC

GCACGCCCGCGAGCTGATCGACCAGGAAGGCCGCACCGTGAAAGAGGCGGCTGCACTGCTTGGCGTGCAT

CGCTCGACCCTGTACCGCGCACTTGAGCGCAGCGAGGAAGTGACGCCCACCGAGGCCAGGCGGCGCGGTG

CCTTCCGTGAGGACGCATTGACCGAGGCCGACGCCCTGGCGGCCGCCGAGAATGAACGCCAAGAGGAACA

AGCATGAAACCGCACCAGGACGGCCAGGACGAACCGTTTTTCATTACCGAAGAGATCGAGGCGGAGATGA

TCGCGGCCGGGTACGTGTTCGAGCCGCCCGCGCACGTCTCAACCGTGCGGCTGCATGAAATCCTGGCCGG

TTTGTCTGATGCCAAGCTGGCGGCCTGGCCGGCCAGCTTGGCCGCTGAAGAAACCGAGCGCCGCCGTCTA

AAAAGGTGATGTGTATTTGAGTAAAACAGCTTGCGTCATGCGGTCGCTGCGTATATGATGCGATGAGTAA

ATAAACAAATACGCAAGGGGAACGCATGAAGGTTATCGCTGTACTTAACCAGAAAGGCGGGTCAGGCAAG

ACGACCATCGCAACCCATCTAGCCCGCGCCCTGCAACTCGCCGGGGCCGATGTTCTGTTAGTCGATTCCG

ATCCCCAGGGCAGTGCCCGCGATTGGGCGGCCGTGCGGGAAGATCAACCGCTAACCGTTGTCGGCATCGA

CCGCCCGACGATTGACCGCGACGTGAAGGCCATCGGCCGGCGCGACTTCGTAGTGATCGACGGAGCGCCC

CAGGCGGCGGACTTGGCTGTGTCCGCGATCAAGGCAGCCGACTTCGTGCTGATTCCGGTGCAGCCAAGCC

CTTACGACATATGGGCCACCGCCGACCTGGTGGAGCTGGTTAAGCAGCGCATTGAGGTCACGGATGGAAG

GCTACAAGCGGCCTTTGTCGTGTCGCGGGCGATCAAAGGCACGCGCATCGGCGGTGAGGTTGCCGAGGCG

CTGGCCGGGTACGAGCTGCCCATTCTTGAGTCCCGTATCACGCAGCGCGTGAGCTACCCAGGCACTGCCG

CCGCCGGCACAACCGTTCTTGAATCAGAACCCGAGGGCGACGCTGCCCGCGAGGTCCAGGCGCTGGCCGC

TGAAATTAAATCAAAACTCATTTGAGTTAATGAGGTAAAGAGAAAATGAGCAAAAGCACAAACACGCTAA

GTGCCGGCCGTCCGAGCGCACGCAGCAGCAAGGCTGCAACGTTGGCCAGCCTGGCAGACACGCCAGCCAT

GAAGCGGGTCAACTTTCAGTTGCCGGCGGAGGATCACACCAAGCTGAAGATGTACGCGGTACGCCAAGGC

AAGACCATTACCGAGCTGCTATCTGAATACATCGCGCAGCTACCAGAGTAAATGAGCAAATGAATAAATG

AGTAGATGAATTTTAGCGGCTAAAGGAGGCGGCATGGAAAATCAAGAACAACCAGGCACCGACGCCGTGG

AATGCCCCATGTGTGGAGGAACGGGCGGTTGGCCAGGCGTAAGCGGCTGGGTTGTCTGCCGGCCCTGCAA

TGGCACTGGAACCCCCAAGCCCGAGGAATCGGCGTGACGGTCGCAAACCATCCGGCCCGGTACAAATCGG

CGCGGCGCTGGGTGATGACCTGGTGGAGAAGTTGAAGGCCGCGCAGGCCGCCCAGCGGCAACGCATCGAG

GCAGAAGCACGCCCCGGTGAATCGTGGCAAGCGGCCGCTGATCGAATCCGCAAAGAATCCCGGCAACCGC

CGGCAGCCGGTGCGCCGTCGATTAGGAAGCCGCCCAAGGGCGACGAGCAACCAGATTTTTTCGTTCCGAT

GCTCTATGACGTGGGCACCCGCGATAGTCGCAGCATCATGGACGTGGCCGTTTTCCGTCTGTCGAAGCGT

GACCGACGAGCTGGCGAGGTGATCCGCTACGAGCTTCCAGACGGGCACGTAGAGGTTTCCGCAGGGCCGG

CCGGCATGGCCAGTGTGTGGGATTACGACCTGGTACTGATGGCGGTTTCCCATCTAACCGAATCCATGAA

CCGATACCGGGAAGGGAAGGGAGACAAGCCCGGCCGCGTGTTCCGTCCACACGTTGCGGACGTACTCAAG

TTCTGCCGGCGAGCCGATGGCGGAAAGCAGAAAGACGACCTGGTAGAAACCTGCATTCGGTTAAACACCA

CGCACGTTGCCATGCAGCGTACGAAGAAGGCCAAGAACGGCCGCCTGGTGACGGTATCCGAGGGTGAAGC

CTTGATTAGCCGCTACAAGATCGTAAAGAGCGAAACCGGGCGGCCGGAGTACATCGAGATCGAGCTAGCT

GATTGGATGTACCGCGAGATCACAGAAGGCAAGAACCCGGACGTGCTGACGGTTCACCCCGATTACTTTT

TGATCGATCCCGGCATCGGCCGTTTTCTCTACCGCCTGGCACGCCGCGCCGCAGGCAAGGCAGAAGCCAG

ATGGTTGTTCAAGACGATCTACGAACGCAGTGGCAGCGCCGGAGAGTTCAAGAAGTTCTGTTTCACCGTG

CGCAAGCTGATCGGGTCAAATGACCTGCCGGAGTACGATTTGAAGGAGGAGGCGGGGCAGGCTGGCCCGA

TCCTAGTCATGCGCTACCGCAACCTGATCGAGGGCGAAGCATCCGCCGGTTCCTAATGTACGGAGCAGAT

GCTAGGGCAAATTGCCCTAGCAGGGGAAAAAGGTCGAAAAGGTCTCTTTCCTGTGGATAGCACGTACATT

GGGAACCCAAAGCCGTACATTGGGAACCGGAACCCGTACATTGGGAACCCAAAGCCGTACATTGGGAACC

GGTCACACATGTAAGTGACTGATATAAAAGAGAAAAAAGGCGATTTTTCCGCCTAAAACTCTTTAAAACT

TATTAAAACTCTTAAAACCCGCCTGGCCTGTGCATAACTGTCTGGCCAGCGCACAGCCGAAGAGCTGCAA

AAAGCGCCTACCCTTCGGTCGCTGCGCTCCCTACGCCCCGCCGCTTCGCGTCGGCCTATCGCGGCCGCTG

GCCGCTCAAAAATGGCTGGCCTACGGCCAGGCAATCTACCAGGGCGCGGACAAGCCGCGCCGTCGCCACT

CGACCGCCGGCGCCCACATCAAGGCACCCTGCCTCGCGCGTTTCGGTGATGACGGTGAAAACCTCTGACA

CATGCAGCTCCCGGAGACGGTCACAGCTTGTCTGTAAGCGGATGCCGGGAGCAGACAAGCCCGTCAGGGC

GCGTCAGCGGGTGTTGGCGGGTGTCGGGGCGCAGCCATGACCCAGTCACGTAGCGATAGCGGAGTGTATA

CTGGCTTAACTATGCGGCATCAGAGCAGATTGTACTGAGAGTGCACCATATGCGGTGTGAAATACCGCAC

AGATGCGTAAGGAGAAAATACCGCATCAGGCGCTCTTCCGCTTCCTCGCTCACTGACTCGCTGCGCTCGG

TCGTTCGGCTGCGGCGAGCGGTATCAGCTCACTCAAAGGCGGTAATACGGTTATCCACAGAATCAGGGGA

TAACGCAGGAAAGAACATGTGAGCAAAAGGCCAGCAAAAGGCCAGGAACCGTAAAAAGGCCGCGTTGCTG

GCGTTTTTCCATAGGCTCCGCCCCCCTGACGAGCATCACAAAAATCGACGCTCAAGTCAGAGGTGGCGAA

ACCCGACAGGACTATAAAGATACCAGGCGTTTCCCCCTGGAAGCTCCCTCGTGCGCTCTCCTGTTCCGAC

CCTGCCGCTTACCGGATACCTGTCCGCCTTTCTCCCTTCGGGAAGCGTGGCGCTTTCTCATAGCTCACGC

TGTAGGTATCTCAGTTCGGTGTAGGTCGTTCGCTCCAAGCTGGGCTGTGTGCACGAACCCCCCGTTCAGC

CCGACCGCTGCGCCTTATCCGGTAACTATCGTCTTGAGTCCAACCCGGTAAGACACGACTTATCGCCACT

GGCAGCAGCCACTGGTAACAGGATTAGCAGAGCGAGGTATGTAGGCGGTGCTACAGAGTTCTTGAAGTGG

TGGCCTAACTACGGCTACACTAGAAGGACAGTATTTGGTATCTGCGCTCTGCTGAAGCCAGTTACCTTCG

GAAAAAGAGTTGGTAGCTCTTGATCCGGCAAACAAACCACCGCTGGTAGCGGTGGTTTTTTTGTTTGCAA

GCAGCAGATTACGCGCAGAAAAAAAGGATCTCAAGAAGATCCTTTGATCTTTTCTACGGGGTCTGACGCT

CAGTGGAACGAAAACTCACGTTAAGGGATTTTGGTCATGCATTCTAGGTACTAAAACAATTCATCCAGTA

AAATATAATATTTTATTTTCTCCCAATCAGGCTTGATCCCCAGTAAGTCAAAAAATAGCTCGACATACTG

TTCTTCCCCGATATCCTCCCTGATCGACCGGACGCAGAAGGCAATGTCATACCACTTGTCCGCCCTGCCG

CTTCTCCCAAGATCAATAAAGCCACTTACTTTGCCATCTTTCACAAAGATGTTGCTGTCTCCCAGGTCGC

CGTGGGAAAAGACAAGTTCCTCTTCGGGCTTTTCCGTCTTTAAAAAATCATACAGCTCGCGCGGATCTTT

AAATGGAGTGTCTTCTTCCCAGTTTTCGCAATCCACATCGGCCAGATCGTTATTCAGTAAGTAATCCAAT

TCGGCTAAGCGGCTGTCTAAGCTATTCGTATAGGGACAATCCGATATGTCGATGGAGTGAAAGAGCCTGA

TGCACTCCGCATACAGCTCGATAATCTTTTCAGGGCTTTGTTCATCTTCATACTCTTCCGAGCAAAGGAC

GCCATCGGCCTCACTCATGAGCAGATTGCTCCAGCCATCATGCCGTTCAAAGTGCAGGACCTTTGGAACA

GGCAGCTTTCCTTCCAGCCATAGCATCATGTCCTTTTCCCGTTCCACATCATAGGTGGTCCCTTTATACC

GGCTGTCCGTCATTTTTAAATATAGGTTTTCATTTTCTCCCACCAGCTTATATACCTTAGCAGGAGACAT

TCCTTCCGTATCTTTTACGCAGCGGTATTTTTCGATCAGTTTTTTCAATTCCGGTGATATTCTCATTTTA

GCCATTTATTATTTCCTTCCTCTTTTCTACAGTATTTAAAGATACCCCAAGAAGCTAATTATAACAAGAC

GAACTCCAATTCACTGTTCCTTGCATTCTAAAACCTTAAATACCAGAAAACAGCTTTTTCAAAGTTGTTT

TCAAAGTTGGCGTATAACATAGTATCGACGGAGCCGATTTTGAAACCGCGGTGATCACAGGCAGCAACGC

TCTGTCATCGTTACAATCAACATGCTACCCTCCGCGAGATCATCCGTGTTTCAAACCCGGCAGCTTAGTT

GCCGTTCTTCCGAATAGCATCGGTAACATGAGCAAAGTCTGCCGCCTTACAACGGCTCTCCCGCTGACGC

CGTCCCGGACTGATGGGCTGCCTGTATCGAGTGGTGATTTTGTGCCGAGCTGCCGGTCGGGGAGCTGTTG

GCTGGCTGGTGGCAGGATATATTGTGGTGTAAACAAATTGACGCTTAGACAACTTAATAACACATTGCGG

ACGTTTTTAATGTACTGAATTAACGCCGAATTAATTCGGGGGATCTGGATTTTAGTACTGGATTTTGGTT

TTAGGAATTAGAAATTTTATTGATAGAAGTATTTTACAAATACAAATACATACTAAGGGTTTCTTATATG

CTCAACACATGAGCGAAACCCTATAGGAACCCTAATTCCCTTATCTGGGAACTACTCACACATTATTATG

GAGAAACTCGAAGATCCGTCGAGCTTGTCGATCGACAGATCCGGTCGGCATCTACTCTATTTCTTTGCCC

TCGGACGAGTGCTGGGGCGTCGGTTTCCACTATCGGCGAGTACTTCTACACAGCCATCGGTCCAGACGGC

CGCGCTTCTGCGGGCGATTTGTGTACGCCCGACAGTCCCGGCTCCGGATCGGACGATTGCGTCGCATCGA

CCCTGCGCCCAAGCTGCATCATCGAAATTGCCGTCAACCAAGCTCTGATAGAGTTGGTCAAGACCAATGC

GGAGCATATACGCCCGGAGTCGTGGCGATCCTGCAAGCTCCGGATGCCTCCGCTCGAAGTAGCGCGTCTG

CTGCTCCATACAAGCCAACCACGGCCTCCAGAAGAAGATGTTGGCGACCTCGTATTGGGAATCCCCGAAC

ATCGCCTCGCTCCAGTCAATGACCGCTGTTATGCGGCCATTGTCCGTCAGGACATTGTTGGAGCCGAAAT

CCGCGTGCACGAGGTGCCGGACTTCGGGGCAGTCCTCGGCCCAAAGCATCAGCTCATCGAGAGCCTGCGC

GACGGACGCACTGACGGTGTCGTCCATCACAGTTTGCCAGTGATACACATGGGGATCAGCAATCGCGCAT

ATGAAATCACGCCATGTAGTGTATTGACCGATTCCTTGCGGTCCGAATGGGCCGAACCCGCTCGTCTGGC

TAAGATCGGCCGCAGCGATCGCATCCATGGCCTCCGCGACCGGCTGCAGTTATCATCATCATCATAGACA

CACGAAATAAAGTAATCAGATTATCAGTTAAAGCTATGTAATATTTACACCATAACCAATCAATTAAAAA

ATAGATCAGTTTAAAGAAAGATCAAAGCTCAAAAAAATAAAAAGAGAAAAGGGTCCTAACCAAGAAAATG

AAGGAGAAAAACTAGAAATTTACCTGCAGAACAGCGGGCAGTTCGGTTTCAGGCAGGTCTTGCAACGTGA

CACCCTGTGCACGGCGGGAGATGCAATAGGTCAGGCTCTCGCTAAACTCCCCAATGTCAAGCACTTCCGG

AATCGGGAGCGCGGCCGATGCAAAGTGCCGATAAACATAACGATCTTTGTAGAAACCATCGGCGCAGCTA

TTTACCCGCAGGACATATCCACGCCCTCCTACATCGAAGCTGAAAGCACGAGATTCTTCGCCCTCCGAGA

GCTGCATCAGGTCGGAGACGCTGTCGAACTTTTCGATCAGAAACTTCTCGACAGACGTCGCGGTGAGTTC

AGGCTTTTTCATATCTCATTGCCCCCCCGGATCTGCGAAAGCTCGACGGATCTTCGAGAGAGATAGATTT

GTAGAGAGAGACTGGTGATTTCAGCGTGTCCTCTCCAAATGAAATGAACTTCCTTATATAGAGGAAGGTC

TTGCGAAGGATAGTGGGATTGTGCGTCATCCCTTACGTCAGTGGAGATATCACATCAATCCACTTGCTTT

GAAGACGTGGTTGGAACGTCTTCTTTTTCCACGATGCTCCTCGTGGGTGGGGGTCCATCTTTGGGACCAC

TGTCGGCAGAGGCATCTTGAACGATAGCCTTTCCTTTATCGCAATGATGGCATTTGTAGGTGCCACCTTC

CTTTTCTACTGTCCTTTTGATGAAGTGACAGATAGCTGGGCAATGGAATCCGAGGAGGTTTCCCGATATT

ACCCTTTGTTGAAAAGTCTCAATAGCCCTTTGGTCTTCTGAGACTGTATCTTTGATATTCTTGGAGTAGA

CGAGAGTGTCGTGCTCCACCATGTTATCACATCAATCCACTTGCTTTGAAGACGTGGTTGGAACGTCTTC

TTTTTCCACGATGCTCCTCGTGGGTGGGGGTCCATCTTTGGGACCACTGTCGGCAGAGGCATCTTGAACG

ATAGCCTTTCCTTTATCGCAATGATGGCATTTGTAGGTGCCACCTTCCTTTTCTACTGTCCTTTTGATGA

AGTGACAGATAGCTGGGCAATGGAATCCGAGGAGGTTTCCCGATATTACCCTTTGTTGAAAAGTCTCAAT

AGCCCTTTGGTCTTCTGAGACTGTATCTTTGATATTCTTGGAGTAGACGAGAGTGTCGTGCTCCACCATG

TTGGCAAGCTGCTCTAGCCAATACGCAAACCGCCTCTCCCCGCGCGTTGGCCGATTCATTAATGCAGCTG

GCACGACAGGTTTCCCGACTGGAAAGCGGGCAGTGAGCGCAACGCAATTAATGTGAGTTAGCTCACTCAT

TAGGCACCCCAGGCTTTACACTTTATGCTTCCGGCTCGTATGTTGTGTGGAATTGTGAGCGGATAACAAT

TTCACACAGGAAACAGCTATGACCATGATTAC

>gi|11559672|gb|AF294982.1| Binary vector pINDEX3, complete sequence

GAATTCAGATCTATCGATGCAGGTCCCCAGATTAGCCTTTTCCAATTTCAGAAAGAATGCTAACCCACAG

ATGGTTAGAGAGGCTTACGCAGCAGGTCTCATCAAGACGATCTACCCGAGCAATAATCTCCAGGAAATCA

AATACCTTCCCAAGAAGGTTAAAGATGCAGTCAAAAGATTCAGGACTAACTGCATCAAGAACACAGAGAA

AGATATATTTCTCAAGATCAGAAGTACTATTCCAGTATGGACGATTCAAGGCTTGCTTCACAAACCAAGG

CAAGTAATAGAGATTGGAGTCTCTAAAAAGGTAGTTCCCACTGAATCAAAGGCCATGGAGTCAAAGATTC

AAATAGAGGACCTAACAGAACTCGCCGTAAAGACTGGCGAACAGTTCATACAGAGTCTCTTACGACTCAA

TGACAAGAAGAAAATCTTCGTCAACATGGTGGAGCACGACACACTTGTCTACTCCAAAAATATCAAAGAT

ACAGTCTCAGAAGACCAAAGGGCAATTGAGACTTTTCAACAAAGGGTAATATCCGGAAACCTCCTCGGAT

TCCATTGCCCAGCTATCTGTCACTTTATTGTGAAGATAGTGGAAAAGGAAGGTGGCTCCTACAAATGCCA

TCATTGCGATAAAGGAAAGGCCATCGTTGAAGATGCCTCTGCCGACAGTGGTCCCAAAGATGGACCCCCA

CCCACGAGGAGCATCGTGGAAAAAGAAGACGTTCCAACCACGTCTTCAAAGCAAGTGGATTGATGTGATA

TCTCCACTGACGTAAGGGATGACGCACAATCCCACTATCCTTCGCAAGACCCTTCCTCTATATAAGGAAG

TTCATTTCATTTGGAGAGAACACGGGGGACTCTAGTTTAAACAATGGCAGATCCAATGAAGCTACTGTCT

TCTATCGAACAAGCATGCGATATTTGCCGACTTAAAAAGCTCAAGTGCTCCAAAGAAAAACCGAAGTGCG

CCAAGTGTCTGAAGAACAACTGGGAGTGTCGCTACTCTCCCAAAACCAAAAGGTCTCCGCTGACTAGGGC

ACATCTGACAGAAGTGGAATCAAGGCTAGAAAGACTGGAACAGCTATTTCTACTGATTTTTCCTAGGTCG

AGCGCCCCCCCGACCGATGTCAGCCTGGGGGACGAGCTCCACTTAGACGGCGAGGACGTGGCGATGGCGC

ATGCCGACGCGCTAGACGATTTCGATCTGGACATGTTGGGGGACGGGGATTCCCCGGGTCCGGGATTTAC

CCCCCACGACTCCGCCCCCTACGGCGCTCTGGATATGGCCGACTTCGAGTTTGAGCAGATGTTTACCGAT

GCCCTTGGAATTGACGAGTACGGTGGGGATCCAATTCAGCAAGCCACTGCAGGAGTCTCACAAGACACTT

CGGAAAATCCTAACAAAACAATAGTTCCTGCTGCATTACCACAGCTCACCCCTACCTTGGTGTCACTGCT

GGAGGTGATTGAACCCGAGGTGTTGTATGCAGGATATGATAGCTCTGTTCCAGATTCAGCATGGAGAATT

ATGACCACACTCAACATGTTAGGTGGGCGTCAAGTGATTGCAGCAGTGAAATGGGCAAAGGCGATACCAG

GCTTCAGAAACTTACACCTGGATGACCAAATGACCCTGCTACAGTACTCATGGATGTTTCTCATGGCATT

TGCCCTGGGTTGGAGATCATACAGACAATCAAGTGGAAACCTGCTCTGCTTTGCTCCTGATCTGATTATT

AATGAGCAGAGAATGTCTCTACCCTGCATGTATGACCAATGTAAACACATGCTGTTTGTCTCCTCTGAAT

TACAAAGATTGCAGGTATCCTATGAAGAGTATCTCTGTATGAAAACCTTACTGCTTCTCTCCTCAGTTCC

TAAGGAAGGTCTGAAGAGCCAAGAGTTATTTGATGAGATTCGAATGACTTATATCAAAGAGCTAGGAAAA

GCCATCGTCAAAAGGGAAGGGAACTCCAGTCAGAACTGGCAACGGTTTTACCAACTGACAAAGCTTCTGG

ACTCCATGCATGAGGTGGTTGAGAATCTCCTTACCTACTGCTTCCAGACATTTTTGGATAAGACCATGAG

TATTGAATTCCCAGAGATGTTAGCTGAAATCATCACTAATCAGATACCAAAATATTCAAATGGAAATATC

AAAAAGCTTCTGTTTCATCAAAAATGACTCGACCTAACTGAGTAAGCTAGCTTGTTCGAGTATTATGGCA

TTGGGAAAACTGTTTTTCTTGTACCATTTGTTGTGCTTGTAATTTACTGTGTTTTTTATTCGGTTTTCGC

TATCGAACTGTGAAATGGAAATGGATGGAGAAGAGTTAATGAATGATATGGTCCTTTTGTTCATTCTCAA

ATTAATATTATTTGTTTTTTCTCTTATTTGTTGTGTGTTGAATTTGAAATTATAAGAGATATGCAAACAT

TTTGTTTTGAGTAAAAATGTGTCAAATCGTGGCCTCTAATGACCGAAGTTAATATGAGGAGTAAAACACT

AGATCCCCAAACAAGCTTGAAACTGAAGGCGGGAAACGACAATCTGATCATGGTACCCGGGAGCTCGAAT

TCGAAGCTTCTGCAGACGCGTCGACGTCATATGGATCCGATATCGCCGTGGCGGCCGTCTGGACCGATGG

CTGTGTAGAAGTACTCGCCGATAGTGGAAACCGACGCCCCAGCACTCGTCCGGGATCTTGGAGGTGATGT

AACATGATCACAAGCTGATCCCCCGAATTTCCCCGATCGTTCAAACATTTGGCAATAAAGTTTCTTAAGA

TTGAATCCTGTTGCCGGTCTTGCGATGATTATCATATAATTTCTGTTGAATTACGTTAAGCATGTAATAA

TTAACATGTAATGCATGACGTTATTTATGAGATGGGTTTTTATGATTAGAGTCCCGCAATTATACATTTA

ATACGCGATAGAAAACAAAATATAGCGCGCAAACTAGGATAAATTATCGCGCGCGGTGTCATCTATGTTA

CTAGATCGGGAATTGATCCCCCCTCGACAGCTTGCATGCCGGTCGACTCTAGAGGATCCGGGTGACAGCC

CTCCGACGGGTGACAGCCCTCCGACGGGTGACAGCCCTCCGACGGGTGACAGCCCTCCGAATTCGAGCTC

GGTACCCGGGGATCTGTCGACCTCGATCGAGATCTTCGCAAGACCCTTCCTCTATATAAGGAAGTTCATT

TCATTTGGAGAGGACACGCTGAAGCTAGTCGACTCTAGCCTCGACACGTGTAACTAGTTACCTGCAGGTA

CTCGAGTATGTACATCTAGTCGATCCAGGCCTCCCAGCTTTCGTCCGTATCATCGGTTTCGACAACGTTC

GTCAAGTTCAATGCATCAGTTTCATTGCCCACACACCAGAATCCTACTAAGTTTGAGTATTATGGCATTG

GAAAAGCTGTTTTCTTCTATCATTTGTTCTGCTTGTAATTTACTGTGTTCTTTCAGTTTTTGTTTTCGGA

CATCAAAATGCAAATGGATGGATAAGAGTTAATAAATGATATGGTCCTTTTGTTCATTCTCAAATTATTA

TTATCTGTTGTTTTTACTTTAATGGGTTGAATTTAAGTAAGAAAGGAACTAACAGTGTGATATTAAGGTG

CAATGTTAGACATATAAAACAGTCTTTCACCTCTCTTTGGTTATGTCTTGAATTGGTTTGTTTCTTCACT

TATCTGTGTAATCAAGTTTACTATGAGTCTATGATCAAGTAATTATGCAATCAAGTTAAGTACAGTATAG

GCTTTTTGTGTCGAGGGGGTACCGAGTCGAGGAATTCGATATCAAGCTTGGCACTGGCCGTCGTTTTACA

ACGTCGTGACTGGGAAAACCCTGGCGTTACCCAACTTAATCGCCTTGCAGCACATCCCCCTTTCGCCAGC

TGGCGTAATAGCGAAGAGGCCCGCACCGATCGCCCTTCCCAACAGTTGCGCAGCCTGAATGGCGAATGCT

AGAGCAGCTTGAGCTTGGATCAGATTGTCGTTTCCCGCCTTCAGTTTAAACTATCAGTGTTTGACAGGAT

ATATTGGCGGGTAAACCTAAGAGAAAAGAGCGTTTATTAGAATAACGGATATTTAAAAGGGCGTGAAAAG

GTTTATCCGTTCGTCCATTTGTATGTGCATGCCAACCACAGGGTTCCCCTCGGGATCAAAGTACTTTGAT

CCAACCCCTCCGCTGCTATAGTGCAGTCGGCTTCTGACGTTCAGTGCAGCCGTCTTCTGAAAACGACATG

TCGCACAAGTCCTAAGTTACGCGACAGGCTGCCGCCCTGCCCTTTTCCTGGCGTTTTCTTGTCGCGTGTT

TTAGTCGCATAAAGTAGAATACTTGCGACTAGAACCGGAGACATTACGCCATGAACAAGAGCGCCGCCGC

TGGCCTGCTGGGCTATGCCCGCGTCAGCACCGACGACCAGGACTTGACCAACCAACGGGCCGAACTGCAC

GCGGCCGGCTGCACCAAGCTGTTTTCCGAGAAGATCACCGGCACCAGGCGCGACCGCCCGGAGCTGGCCA

GGATGCTTGACCACCTACGCCCTGGCGACGTTGTGACAGTGACCAGGCTAGACCGCCTGGCCCGCAGCAC

CCGCGACCTACTGGACATTGCCGAGCGCATCCAGGAGGCCGGCGCGGGCCTGCGTAGCCTGGCAGAGCCG

TGGGCCGACACCACCACGCCGGCCGGCCGCATGGTGTTGACCGTGTTCGCCGGCATTGCCGAGTTCGAGC

GTTCCCTAATCATCGACCGCACCCGGAGCGGGCGCGAGGCCGCCAAGGCCCGAGGCGTGAAGTTTGGCCC

CCGCCCTACCCTCACCCCGGCACAGATCGCGCACGCCCGCGAGCTGATCGACCAGGAAGGCCGCACCGTG

AAAGAGGCGGCTGCACTGCTTGGCGTGCATCGCTCGACCCTGTACCGCGCACTTGAGCGCAGCGAGGAAG

TGACGCCCACCGAGGCCAGGCGGCGCGGTGCCTTCCGTGAGGACGCATTGACCGAGGCCGACGCCCTGGC

GGCCGCCGAGAATGAACGCCAAGAGGAACAAGCATGAAACCGCACCAGGACGGCCAGGACGAACCGTTTT

TCATTACCGAAGAGATCGAGGCGGAGATGATCGCGGCCGGGTACGTGTTCGAGCCGCCCGCGCACGTCTC

AACCGTGCGGCTGCATGAAATCCTGGCCGGTTTGTCTGATGCCAAGCTGGCGGCCTGGCCGGCCAGCTTG

GCCGCTGAAGAAACCGAGCGCCGCCGTCTAAAAAGGTGATGTGTATTTGAGTAAAACAGCTTGCGTCATG

CGGTCGCTGCGTATATGATGCGATGAGTAAATAAACAAATACGCAAGGGGAACGCATGAAGGTTATCGCT

GTACTTAACCAGAAAGGCGGGTCAGGCAAGACGACCATCGCAACCCATCTAGCCCGCGCCCTGCAACTCG

CCGGGGCCGATGTTCTGTTAGTCGATTCCGATCCCCAGGGCAGTGCCCGCGATTGGGCGGCCGTGCGGGA

AGATCAACCGCTAACCGTTGTCGGCATCGACCGCCCGACGATTGACCGCGACGTGAAGGCCATCGGCCGG

CGCGACTTCGTAGTGATCGACGGAGCGCCCCAGGCGGCGGACTTGGCTGTGTCCGCGATCAAGGCAGCCG

ACTTCGTGCTGATTCCGGTGCAGCCAAGCCCTTACGACATATGGGCCACCGCCGACCTGGTGGAGCTGGT

TAAGCAGCGCATTGAGGTCACGGATGGAAGGCTACAAGCGGCCTTTGTCGTGTCGCGGGCGATCAAAGGC

ACGCGCATCGGCGGTGAGGTTGCCGAGGCGCTGGCCGGGTACGAGCTGCCCATTCTTGAGTCCCGTATCA

CGCAGCGCGTGAGCTACCCAGGCACTGCCGCCGCCGGCACAACCGTTCTTGAATCAGAACCCGAGGGCGA

CGCTGCCCGCGAGGTCCAGGCGCTGGCCGCTGAAATTAAATCAAAACTCATTTGAGTTAATGAGGTAAAG

AGAAAATGAGCAAAAGCACAAACACGCTAAGTGCCGGCCGTCCGAGCGCACGCAGCAGCAAGGCTGCAAC

GTTGGCCAGCCTGGCAGACACGCCAGCCATGAAGCGGGTCAACTTTCAGTTGCCGGCGGAGGATCACACC

AAGCTGAAGATGTACGCGGTACGCCAAGGCAAGACCATTACCGAGCTGCTATCTGAATACATCGCGCAGC

TACCAGAGTAAATGAGCAAATGAATAAATGAGTAGATGAATTTTAGCGGCTAAAGGAGGCGGCATGGAAA

ATCAAGAACAACCAGGCACCGACGCCGTGGAATGCCCCATGTGTGGAGGAACGGGCGGTTGGCCAGGCGT

AAGCGGCTGGGTTGTCTGCCGGCCCTGCAATGGCACTGGAACCCCCAAGCCCGAGGAATCGGCGTGACGG

TCGCAAACCATCCGGCCCGGTACAAATCGGCGCGGCGCTGGGTGATGACCTGGTGGAGAAGTTGAAGGCC

GCGCAGGCCGCCCAGCGGCAACGCATCGAGGCAGAAGCACGCCCCGGTGAATCGTGGCAAGCGGCCGCTG

ATCGAATCCGCAAAGAATCCCGGCAACCGCCGGCAGCCGGTGCGCCGTCGATTAGGAAGCCGCCCAAGGG

CGACGAGCAACCAGATTTTTTCGTTCCGATGCTCTATGACGTGGGCACCCGCGATAGTCGCAGCATCATG

GACGTGGCCGTTTTCCGTCTGTCGAAGCGTGACCGACGAGCTGGCGAGGTGATCCGCTACGAGCTTCCAG

ACGGGCACGTAGAGGTTTCCGCAGGGCCGGCCGGCATGGCCAGTGTGTGGGATTACGACCTGGTACTGAT

GGCGGTTTCCCATCTAACCGAATCCATGAACCGATACCGGGAAGGGAAGGGAGACAAGCCCGGCCGCGTG

TTCCGTCCACACGTTGCGGACGTACTCAAGTTCTGCCGGCGAGCCGATGGCGGAAAGCAGAAAGACGACC

TGGTAGAAACCTGCATTCGGTTAAACACCACGCACGTTGCCATGCAGCGTACGAAGAAGGCCAAGAACGG

CCGCCTGGTGACGGTATCCGAGGGTGAAGCCTTGATTAGCCGCTACAAGATCGTAAAGAGCGAAACCGGG

CGGCCGGAGTACATCGAGATCGAGCTAGCTGATTGGATGTACCGCGAGATCACAGAAGGCAAGAACCCGG

ACGTGCTGACGGTTCACCCCGATTACTTTTTGATCGATCCCGGCATCGGCCGTTTTCTCTACCGCCTGGC

ACGCCGCGCCGCAGGCAAGGCAGAAGCCAGATGGTTGTTCAAGACGATCTACGAACGCAGTGGCAGCGCC

GGAGAGTTCAAGAAGTTCTGTTTCACCGTGCGCAAGCTGATCGGGTCAAATGACCTGCCGGAGTACGATT

TGAAGGAGGAGGCGGGGCAGGCTGGCCCGATCCTAGTCATGCGCTACCGCAACCTGATCGAGGGCGAAGC

ATCCGCCGGTTCCTAATGTACGGAGCAGATGCTAGGGCAAATTGCCCTAGCAGGGGAAAAAGGTCGAAAA

GGTCTCTTTCCTGTGGATAGCACGTACATTGGGAACCCAAAGCCGTACATTGGGAACCGGAACCCGTACA

TTGGGAACCCAAAGCCGTACATTGGGAACCGGTCACACATGTAAGTGACTGATATAAAAGAGAAAAAAGG

CGATTTTTCCGCCTAAAACTCTTTAAAACTTATTAAAACTCTTAAAACCCGCCTGGCCTGTGCATAACTG

TCTGGCCAGCGCACAGCCGAAGAGCTGCAAAAAGCGCCTACCCTTCGGTCGCTGCGCTCCCTACGCCCCG

CCGCTTCGCGTCGGCCTATCGCGGCCGCTGGCCGCTCAAAAATGGCTGGCCTACGGCCAGGCAATCTACC

AGGGCGCGGACAAGCCGCGCCGTCGCCACTCGACCGCCGGCGCCCACATCAAGGCACCCTGCCTCGCGCG

TTTCGGTGATGACGGTGAAAACCTCTGACACATGCAGCTCCCGGAGACGGTCACAGCTTGTCTGTAAGCG

GATGCCGGGAGCAGACAAGCCCGTCAGGGCGCGTCAGCGGGTGTTGGCGGGTGTCGGGGCGCAGCCATGA

CCCAGTCACGTAGCGATAGCGGAGTGTATACTGGCTTAACTATGCGGCATCAGAGCAGATTGTACTGAGA

GTGCACCATATGCGGTGTGAAATACCGCACAGATGCGTAAGGAGAAAATACCGCATCAGGCGCTCTTCCG

CTTCCTCGCTCACTGACTCGCTGCGCTCGGTCGTTCGGCTGCGGCGAGCGGTATCAGCTCACTCAAAGGC

GGTAATACGGTTATCCACAGAATCAGGGGATAACGCAGGAAAGAACATGTGAGCAAAAGGCCAGCAAAAG

GCCAGGAACCGTAAAAAGGCCGCGTTGCTGGCGTTTTTCCATAGGCTCCGCCCCCCTGACGAGCATCACA

AAAATCGACGCTCAAGTCAGAGGTGGCGAAACCCGACAGGACTATAAAGATACCAGGCGTTTCCCCCTGG

AAGCTCCCTCGTGCGCTCTCCTGTTCCGACCCTGCCGCTTACCGGATACCTGTCCGCCTTTCTCCCTTCG

GGAAGCGTGGCGCTTTCTCATAGCTCACGCTGTAGGTATCTCAGTTCGGTGTAGGTCGTTCGCTCCAAGC

TGGGCTGTGTGCACGAACCCCCCGTTCAGCCCGACCGCTGCGCCTTATCCGGTAACTATCGTCTTGAGTC

CAACCCGGTAAGACACGACTTATCGCCACTGGCAGCAGCCACTGGTAACAGGATTAGCAGAGCGAGGTAT

GTAGGCGGTGCTACAGAGTTCTTGAAGTGGTGGCCTAACTACGGCTACACTAGAAGGACAGTATTTGGTA

TCTGCGCTCTGCTGAAGCCAGTTACCTTCGGAAAAAGAGTTGGTAGCTCTTGATCCGGCAAACAAACCAC

CGCTGGTAGCGGTGGTTTTTTTGTTTGCAAGCAGCAGATTACGCGCAGAAAAAAAGGATCTCAAGAAGAT

CCTTTGATCTTTTCTACGGGGTCTGACGCTCAGTGGAACGAAAACTCACGTTAAGGGATTTTGGTCATGC

ATTCTAGGTACTAAAACAATTCATCCAGTAAAATATAATATTTTATTTTCTCCCAATCAGGCTTGATCCC

CAGTAAGTCAAAAAATAGCTCGACATACTGTTCTTCCCCGATATCCTCCCTGATCGACCGGACGCAGAAG

GCAATGTCATACCACTTGTCCGCCCTGCCGCTTCTCCCAAGATCAATAAAGCCACTTACTTTGCCATCTT

TCACAAAGATGTTGCTGTCTCCCAGGTCGCCGTGGGAAAAGACAAGTTCCTCTTCGGGCTTTTCCGTCTT

TAAAAAATCATACAGCTCGCGCGGATCTTTAAATGGAGTGTCTTCTTCCCAGTTTTCGCAATCCACATCG

GCCAGATCGTTATTCAGTAAGTAATCCAATTCGGCTAAGCGGCTGTCTAAGCTATTCGTATAGGGACAAT

CCGATATGTCGATGGAGTGAAAGAGCCTGATGCACTCCGCATACAGCTCGATAATCTTTTCAGGGCTTTG

TTCATCTTCATACTCTTCCGAGCAAAGGACGCCATCGGCCTCACTCATGAGCAGATTGCTCCAGCCATCA

TGCCGTTCAAAGTGCAGGACCTTTGGAACAGGCAGCTTTCCTTCCAGCCATAGCATCATGTCCTTTTCCC

GTTCCACATCATAGGTGGTCCCTTTATACCGGCTGTCCGTCATTTTTAAATATAGGTTTTCATTTTCTCC

CACCAGCTTATATACCTTAGCAGGAGACATTCCTTCCGTATCTTTTACGCAGCGGTATTTTTCGATCAGT

TTTTTCAATTCCGGTGATATTCTCATTTTAGCCATTTATTATTTCCTTCCTCTTTTCTACAGTATTTAAA

GATACCCCAAGAAGCTAATTATAACAAGACGAACTCCAATTCACTGTTCCTTGCATTCTAAAACCTTAAA

TACCAGAAAACAGCTTTTTCAAAGTTGTTTTCAAAGTTGGCGTATAACATAGTATCGACGGAGCCGATTT

TGAAACCGCGGTGATCACAGGCAGCAACGCTCTGTCATCGTTACAATCAACATGCTACCCTCCGCGAGAT

CATCCGTGTTTCAAACCCGGCAGCTTAGTTGCCGTTCTTCCGAATAGCATCGGTAACATGAGCAAAGTCT

GCCGCCTTACAACGGCTCTCCCGCTGACGCCGTCCCGGACTGATGGGCTGCCTGTATCGAGTGGTGATTT

TGTGCCGAGCTGCCGGTCGGGGAGCTGTTGGCTGGCTGGTGGCAGGATATATTGTGGTGTAAACAAATTG

ACGCTTAGACAACTTAATAACACATTGCGGACGTTTTTAATGTACTGAATTAACGCCGAATTAATTCGGG

GGATCTGGATTTTAGTACTGGATTTTGGTTTTAGGAATTAGAAATTTTATTGATAGAAGTATTTTACAAA

TACAAATACATACTAAGGGTTTCTTATATGCTCAACACATGAGCGAAACCCTATAGGAACCCTAATTCCC

TTATCTGGGAACTACTCACACATTATTATGGAGAAACTCGAAGATCCGTCGAGCTTGTCGATCGACAGAT

CCGGTCGGCATCTACTCTATTTCTTTGCCCTCGGACGAGTGCTGGGGCGTCGGTTTCCACTATCGGCGAG

TACTTCTACACAGCCATCGGTCCAGACGGCCGCGCTTCTGCGGGCGATTTGTGTACGCCCGACAGTCCCG

GCTCCGGATCGGACGATTGCGTCGCATCGACCCTGCGCCCAAGCTGCATCATCGAAATTGCCGTCAACCA

AGCTCTGATAGAGTTGGTCAAGACCAATGCGGAGCATATACGCCCGGAGTCGTGGCGATCCTGCAAGCTC

CGGATGCCTCCGCTCGAAGTAGCGCGTCTGCTGCTCCATACAAGCCAACCACGGCCTCCAGAAGAAGATG

TTGGCGACCTCGTATTGGGAATCCCCGAACATCGCCTCGCTCCAGTCAATGACCGCTGTTATGCGGCCAT

TGTCCGTCAGGACATTGTTGGAGCCGAAATCCGCGTGCACGAGGTGCCGGACTTCGGGGCAGTCCTCGGC

CCAAAGCATCAGCTCATCGAGAGCCTGCGCGACGGACGCACTGACGGTGTCGTCCATCACAGTTTGCCAG

TGATACACATGGGGATCAGCAATCGCGCATATGAAATCACGCCATGTAGTGTATTGACCGATTCCTTGCG

GTCCGAATGGGCCGAACCCGCTCGTCTGGCTAAGATCGGCCGCAGCGATCGCATCCATGGCCTCCGCGAC

CGGCTGCAGTTATCATCATCATCATAGACACACGAAATAAAGTAATCAGATTATCAGTTAAAGCTATGTA

ATATTTACACCATAACCAATCAATTAAAAAATAGATCAGTTTAAAGAAAGATCAAAGCTCAAAAAAATAA

AAAGAGAAAAGGGTCCTAACCAAGAAAATGAAGGAGAAAAACTAGAAATTTACCTGCAGAACAGCGGGCA

GTTCGGTTTCAGGCAGGTCTTGCAACGTGACACCCTGTGCACGGCGGGAGATGCAATAGGTCAGGCTCTC

GCTAAACTCCCCAATGTCAAGCACTTCCGGAATCGGGAGCGCGGCCGATGCAAAGTGCCGATAAACATAA

CGATCTTTGTAGAAACCATCGGCGCAGCTATTTACCCGCAGGACATATCCACGCCCTCCTACATCGAAGC

TGAAAGCACGAGATTCTTCGCCCTCCGAGAGCTGCATCAGGTCGGAGACGCTGTCGAACTTTTCGATCAG

AAACTTCTCGACAGACGTCGCGGTGAGTTCAGGCTTTTTCATATCTCATTGCCCCCCCGGATCTGCGAAA

GCTCGACGGATCTTCGAGAGAGATAGATTTGTAGAGAGAGACTGGTGATTTCAGCGTGTCCTCTCCAAAT

GAAATGAACTTCCTTATATAGAGGAAGGTCTTGCGAAGGATAGTGGGATTGTGCGTCATCCCTTACGTCA

GTGGAGATATCACATCAATCCACTTGCTTTGAAGACGTGGTTGGAACGTCTTCTTTTTCCACGATGCTCC

TCGTGGGTGGGGGTCCATCTTTGGGACCACTGTCGGCAGAGGCATCTTGAACGATAGCCTTTCCTTTATC

GCAATGATGGCATTTGTAGGTGCCACCTTCCTTTTCTACTGTCCTTTTGATGAAGTGACAGATAGCTGGG

CAATGGAATCCGAGGAGGTTTCCCGATATTACCCTTTGTTGAAAAGTCTCAATAGCCCTTTGGTCTTCTG

AGACTGTATCTTTGATATTCTTGGAGTAGACGAGAGTGTCGTGCTCCACCATGTTATCACATCAATCCAC

TTGCTTTGAAGACGTGGTTGGAACGTCTTCTTTTTCCACGATGCTCCTCGTGGGTGGGGGTCCATCTTTG

GGACCACTGTCGGCAGAGGCATCTTGAACGATAGCCTTTCCTTTATCGCAATGATGGCATTTGTAGGTGC

CACCTTCCTTTTCTACTGTCCTTTTGATGAAGTGACAGATAGCTGGGCAATGGAATCCGAGGAGGTTTCC

CGATATTACCCTTTGTTGAAAAGTCTCAATAGCCCTTTGGTCTTCTGAGACTGTATCTTTGATATTCTTG

GAGTAGACGAGAGTGTCGTGCTCCACCATGTTGGCAAGCTGCTCTAGCCAATACGCAAACCGCCTCTCCC

CGCGCGTTGGCCGATTCATTAATGCAGCTGGCACGACAGGTTTCCCGACTGGAAAGCGGGCAGTGAGCGC

AACGCAATTAATGTGAGTTAGCTCACTCATTAGGCACCCCAGGCTTTACACTTTATGCTTCCGGCTCGTA

TGTTGTGTGGAATTGTGAGCGGATAACAATTTCACACAGGAAACAGCTATGACCATGATTAC

>gi|11968179|gb|AF309825.2|AF309825 Plant expression vector pER8, complete plasmid sequence

GTTTACCCGCCAATATATCCTGTCAAACACTGATAGTTTAAACTGAAGGCGGGAAACGACAATCTGATCC

AAGCTCAAGCTAAGCTTGCATGCCTGCAGGATATCGTGGATCCAAGCTTGCCACGTGCCGCCACGTGCCG

CCACGTGCCGCCACGTGCCTCTAGAGGATCCATCTCCACTGACGTAAGGGATGACGCACAATCCCACTAT

CCTTCGCAAGACCCTTCCTCTATATAAGGAAGTTCATTTCATTTGGAGAGGACACGCTGGGATCCCCAAT

TCCGGGCGGAATGAAAGCGTTAACGGCCAGGCAACAAGAGGTGTTTGATCTCATCCGTGATCACATCAGC

CAGACAGGTATGCCGCCGACGCGTGCGGAAATCGCGCAGCGTTTGGGGTTCCGTTCCCCAAACGCGGCTG

AAGAACATCTGAAGGCGCTGGCACGCAAAGGCGTTATTGAAATTGTTTCCGGCGCATCACGCGGGATTCG

TCTGTTGCAGGAAGAGGAAGAAGGGTTGCCGCTGGTAGGTCGTGTGGCTGCCGGTGAACCGTCGAGCGCC

CCCCCGACCGATGTCAGCCTGGGGGACGAGCTCCACTTAGACGGCGAGGACGTGGCGATGGCGCATGCCG

ACGCGCTAGACGATTTCGATCTGGACATGTTGGGGGACGGGGATTCCCCGGGTCCGGGATTTACCCCCCA

CGACTCCGCCCCCTACGGCGCTCTGGATATGGCCGACTTCGAGTTTGAGCAGATGTTTACCGATGCCCTT

GGAATTGACGAGTACGGTGGGGATCCGTCTGCTGGAGACATGAGAGCTGCCAACCTTTGGCCAAGCCCGC

TCATGATCAAACGCTCTAAGAAGAACAGCCTGGCCTTGTCCCTGACGGCCGACCAGATGGTCAGTGCCTT

GTTGGATGCTGAGCCCCCCATACTCTATTCCGAGTATGATCCTACCAGACCCTTCAGTGAAGCTTCGATG

ATGGGCTTACTGACCAACCTGGCAGACAGGGAGCTGGTTCACATGATCAACTGGGCGAAGAGGGTGCCAG

GCTTTGTGGATTTGACCCTCCATGATCAGGTCCACCTTCTAGAATGTGCCTGGCTAGAGATCCTGATGAT

TGGTCTCGTCTGGCGCTCCATGGAGCACCCAGTGAAGCTACTGTTTGCTCCTAACTTGCTCTTGGACAGG

AACCAGGGAAAATGTGTAGAGGGCATGGTGGAGATCTTCGACATGCTGCTGGCTACATCATCTCGGTTCC

GCATGATGAATCTGCAGGGAGAGGAGTTTGTGTGCCTCAAATCTATTATTTTGCTTAATTCTGGAGTGTA

CACATTTCTGTCCAGCACCCTGAAGTCTCTGGAAGAGAAGGACCATATCCACCGAGTCCTGGACAAGATC

ACAGACACTTTGATCCACCTGATGGCCAAGGCAGGCCTGACCCTGCAGCAGCAGCACCAGCGGCTGGCCC

AGCTCCTCCTCATCCTCTCCCACATCAGGCACATGAGTAACAAAGGCATGGAGCATCTGTACAGCATGAA

GTGCAAGAACGTGGTGCCCCTCTATGACCTGCTGCTGGAGATGCTGGACGCCCACCGCCTACATGCGCCC

ACTAGCCGTGGAGGGGCATCCGTGGAGGAGACGGACCAAAGCCACTTGGCCACTGCGGGCTCTACTTCAT

CGCATTCCTTGCAAAAGTATTACATCACGGGGGAGGCAGAGGGTTTCCCTGCCACAGTCTGAGAGCTCCC

TGGCGAATTCCCAGAGATGTTAGCTGAAATCATCACTAATCAGATACCAAAATATTCAAATGGAAATATC

AAAAAGCTTCTGTTTCATCAAAAATGACTCGACCTAACTGAGTAAGCTAGCTTGTTCGAGTATTATGGCA

TTGGGAAAACTGTTTTTCTTGTACCATTTGTTGTGCTTGTAATTTACTGTGTTTTTTATTCGGTTTTCGC

TATCGAACTGTGAAATGGAAATGGATGGAGAAGAGTTAATGAATGATATGGTCCTTTTGTTCATTCTCAA

ATTAATATTATTTGTTTTTTCTCTTATTTGTTGTGTGTTGAATTTGAAATTATAAGAGATATGCAAACAT

TTTGTTTTGAGTAAAAATGTGTCAAATCGTGGCCTCTAATGACCGAAGTTAATATGAGGAGTAAAACATC

CCAAACAAGCTTGGAAACTGAAGGCGGGAAACGACAATCTGATCATGAGCGGAGAATTAAGGGAGTCACG

TTATGACCCCCGCCGATGACGCGGGACAAGCCGTTTTACGTTTGGAACTGACAGAACCGCAACGATTGAA

GGAGCCACTCAGCCGCGGGTTTCTGGAGTTTAATGAGCTAAGCACATACGTCAGAAACCATTATTGCGCG

TTCAAAAGTCGCCTAAGGTCACTATCAGCTAGCAAATATTTCTTGTCAAAAATGCTCCACTGACGTTCCA

TAAATTCCCCTCGGTATCCAATTAGAGTCTCATATTCACTCTCAATCCAAATAATCTGCACCGGATCCCC

TAGAATGAAAAAGCCTGAACTCACCGCGACGTCTGTCGAGAAGTTTCTGATCGAAAAGTTCGACAGCGTC

TCCGACCTGATGCAGCTCTCGGAGGGCGAAGAATCTCGTGCTTTCAGCTTCGATGTAGGAGGGCGTGGAT

ATGTCCTGCGGGTAAATAGCTGCGCCGATGGTTTCTACAAAGATCGTTATGTTTATCGGCACTTTGCATC

GGCCGCGCTCCCGATTCCGGAAGTGCTTGACATTGGGGAATTCAGCGAGAGCCTGACCTATTGCATCTCC

CGCCGTGCACAGGGTGTCACGTTGCAAGACCTGCCTGAAACCGAACTGCCCGCTGTTCTGCAGCCGGTCG

CGGAGGCCATGGATGCGATCGCTGCGGCCGATCTTAGCCAGACGAGCGGGTTCGGCCCATTCGGACCGCA

AGGAATCGGTCAATACACTACATGGCGTGATTTCATATGCGCGATTGCTGATCCCCATGTGTATCACTGG

CAAACTGTGATGGACGACACCGTCAGTGCGTCCGTCGCGCAGGCTCTCGATGAGCTGATGCTTTGGGCCG

AGGACTGCCCCGAAGTCCGGCACCTCGTGCACGCGGATTTCGGCTCCAACAATGTCCTGACGGACAATGG

CCGCATAACAGCGGTCATTGACTGGAGCGAGGCGATGTTCGGGGATTCCCAATACGAGGTCGCCAACATC

TTCTTCTGGAGGCCGTGGTTGGCTTGTATGGAGCAGCAGACGCGCTACTTCGAGCGGAGGCATCCGGAGC

TTGCAGGATCGCCGCGGCTCCGGGCGTATATGCTCCGCATTGGTCTTGACCAACTCTATCAGAGCTTGGT

TGACGGCAATTTCGATGATGCAGCTTGGGCGCAGGGTCGATGCGACGCAATCGTCCGATCCGGAGCCGGG

ACTGTCGGGCGTACACAAATCGCCCGCAGAAGCGCGGCCGTCTGGACCGATGGCTGTGTAGAAGTACTCG

CCGATAGTGGAAACCGACGCCCCAGCACTCGTCCGAGGGCAAAGGAATAGCGATCGTTCAAACATTTGGC

AATAAAGTTTCTTAAGATTGAATCCTGTTGCCGGTCTTGCGATGATTATCATATAATTTCTGTTGAATTA

CGTTAAGCATGTAATAATTAACATGTAATGCATGACGTTATTTATGAGATGGGTTTTTATGATTAGAGTC

CCGCAATTATACATTTAATACGCGATAGAAAACAAAATATAGCGCGCAAACTAGGATAAATTATCGCGCG

CGGTGTCATCTATGTTACTAGATCGGGGAATTGATCCCCCCTCGACAGCTTGCATGCCAGCTTGGGCTGC

AGGTCGAGGCTAAAAAACTAATCGCATTATCATCCCCTCGACGTACTGTACATATAACCACTGGTTTTAT

ATACAGCAGTACTGTACATATAACCACTGGTTTTATATACAGCAGTCGACGTACTGTACATATAACCACT

GGTTTTATATACAGCAGTACTGTACATATAACCACTGGTTTTATATACAGCAGTCGAGGTAAGATTAGAT

ATGGATATGTATATGGATATGTATATGGTGGTAATGCCATGTAATATGCTCGACTCTAGGATCTTCGCAA

GACCCTTCCTCTATATAAGGAAGTTCATTTCATTTGGAGAGGACACGCTGAAGCTAGTCGACTCTAGCCT

CGAGGCGCGCCGGGCCCAGGCCTACGCGTTTAATTAACTAGTCGATCCAGGCCTCCCAGCTTTCGTCCGT

ATCATCGGTTTCGACAACGTTCGTCAAGTTCAATGCATCAGTTTCATTGCCCACACACCAGAATCCTACT

AAGTTTGAGTATTATGGCATTGGAAAAGCTGTTTTCTTCTATCATTTGTTCTGCTTGTAATTTACTGTGT

TCTTTCAGTTTTTGTTTTCGGACATCAAAATGCAAATGGATGGATAAGAGTTAATAAATGATATGGTCCT

TTTGTTCATTCTCAAATTATTATTATCTGTTGTTTTTACTTTAATGGGTTGAATTTAAGTAAGAAAGGAA

CTAACAGTGTGATATTAAGGTGCAATGTTAGACATATAAAACAGTCTTTCACCTCTCTTTGGTTATGTCT

TGAATTGGTTTGTTTCTTCACTTATCTGTGTAATCAAGTTTACTATGAGTCTATGATCAAGTAATTATGC

AATCAAGTTAAGTACAGTATAGGCTTTTTGTGTCGAGGGGGTACCGAGTCGAGGAATTCACTGGCCGTCG

TTTTACAACGTCGTGACTGGGAAAACCCTGGCGTTACCCAACTTAATCGCCTTGCAGCACATCCCCCTTT

CGCCAGCTGGCGTAATAGCGAAGAGGCCCGCACCGATCGCCCTTCCCAACAGTTGCGCAGCCTGAATGGC

GGGTACCGAGCTCGAATTCAATTCGGCGTTAATTCAGTACATTAAAAACGTCCGCAATGTGTTATTAAGT

TGTCTAAGCGTCAATTTGTTTACACCACAATATATCCTGCCACCAGCCAGCCAACAGCTCCCCGACCGGC

AGCTCGGCACAAAATCACCACTCGATACAGGCAGCCCATCAGTCCGGGACGGCGTCAGCGGGAGAGCCGT

TGTAAGGCGGCAGACTTTGCTCATGTTACCGATGCTATTCGGAAGAACGGCAACTAAGCTGCCGGGTTTG

AAACACGGATGATCTCGCGGAGGGTAGCATGTTGATTGTAACGATGACAGAGCGTTGCTGCCTGTGATCA

ATTCGGGCACGAACCCAGTGGACATAAGCCTCGTTCGGTTCGTAAGCTGTAATGCAAGTAGCGTAACTGC

CGTCACGCAACTGGTCCAGAACCTTGACCGAACGCAGCGGTGGTAACGGCGCAGTGGCGGTTTTCATGGC

TTCTTGTTATGACATGTTTTTTTGGGGTACAGTCTATGCCTCGGGCATCCAAGCAGCAAGCGCGTTACGC

CGTGGGTCGATGTTTGATGTTATGGAGCAGCAACGATGTTACGCAGCAGGGCAGTCGCCCTAAAACAAAG

TTAAACATCATGGGGGAAGCGGTGATCGCCGAAGTATCGACTCAACTATCAGAGGTAGTTGGCGTCATCG

AGCGCCATCTCGAACCGACGTTGCTGGCCGTACATTTGTACGGCTCCGCAGTGGATGGCGGCCTGAAGCC

ACACAGTGATATTGATTTGCTGGTTACGGTGACCGTAAGGCTTGATGAAACAACGCGGCGAGCTTTGATC

AACGACCTTTTGGAAACTTCGGCTTCCCCTGGAGAGAGCGAGATTCTCCGCGCTGTAGAAGTCACCATTG

TTGTGCACGACGACATCATTCCGTGGCGTTATCCAGCTAAGCGCGAACTGCAATTTGGAGAATGGCAGCG

CAATGACATTCTTGCAGGTATCTTCGAGCCAGCCACGATCGACATTGATCTGGCTATCTTGCTGACAAAA

GCAAGAGAACATAGCGTTGCCTTGGTAGGTCCAGCGGCGGAGGAACTCTTTGATCCGGTTCCTGAACAGG

ATCTATTTGAGGCGCTAAATGAAACCTTAACGCTATGGAACTCGCCGCCCGACTGGGCTGGCGATGAGCG

AAATGTAGTGCTTACGTTGTCCCGCATTTGGTACAGCGCAGTAACCGGCAAAATCGCGCCGAAGGATGTC

GCTGCCGACTGGGCAATGGAGCGCCTGCCGGCCCAGTATCAGCCCGTCATACTTGAAGCTAGACAGGCTT

ATCTTGGACAAGAAGAAGATCGCTTGGCCTCGCGCGCAGATCAGTTGGAAGAATTTGTCCACTACGTGAA

AGGCGAGATCACCAAGGTAGTCGGCAAATAATGTCTAGCTAGAAATTCGTTCAAGCCGACGCCGCTTCGC

CGGCGTTAACTCAAGCGATTAGATGCACTAAGCACATAATTGCTCACAGCCAAACTATCAGGTCAAGTCT

GCTTTTATTATTTTTAAGCGTGCATAATAAGCCCTACACAAATTGGGAGATATATCATGCATGACCAAAA

TCCCTTAACGTGAGTTTTCGTTCCACTGAGCGTCAGACCCCGTAGAAAAGATCAAAGGATCTTCTTGAGA

TCCTTTTTTTCTGCGCGTAATCTGCTGCTTGCAAACAAAAAAACCACCGCTACCAGCGGTGGTTTGTTTG

CCGGATCAAGAGCTACCAACTCTTTTTCCGAAGGTAACTGGCTTCAGCAGAGCGCAGATACCAAATACTG

TCCTTCTAGTGTAGCCGTAGTTAGGCCACCACTTCAAGAACTCTGTAGCACCGCCTACATACCTCGCTCT

GCTAATCCTGTTACCAGTGGCTGCTGCCAGTGGCGATAAGTCGTGTCTTACCGGGTTGGACTCAAGACGA

TAGTTACCGGATAAGGCGCAGCGGTCGGGCTGAACGGGGGGTTCGTGCACACAGCCCAGCTTGGAGCGAA

CGACCTACACCGAACTGAGATACCTACAGCGTGAGCTATGAGAAAGCGCCACGCTTCCCGAAGGGAGAAA

GGCGGACAGGTATCCGGTAAGCGGCAGGGTCGGAACAGGAGAGCGCACGAGGGAGCTTCCAGGGGGAAAC

GCCTGGTATCTTTATAGTCCTGTCGGGTTTCGCCACCTCTGACTTGAGCGTCGATTTTTGTGATGCTCGT

CAGGGGGGCGGAGCCTATGGAAAAACGCCAGCAACGCGGCCTTTTTACGGTTCCTGGCCTTTTGCTGGCC

TTTTGCTCACATGTTCTTTCCTGCGTTATCCCCTGATTCTGTGGATAACCGTATTACCGCCTTTGAGTGA

GCTGATACCGCTCGCCGCAGCCGAACGACCGAGCGCAGCGAGTCAGTGAGCGAGGAAGCGGAAGAGCGCC

TGATGCGGTATTTTCTCCTTACGCATCTGTGCGGTATTTCACACCGCATATGGTGCACTCTCAGTACAAT

CTGCTCTGATGCCGCATAGTTAAGCCAGTATACACTCCGCTATCGCTACGTGACTGGGTCATGGCTGCGC

CCCGACACCCGCCAACACCCGCTGACGCGCCCTGACGGGCTTGTCTGCTCCCGGCATCCGCTTACAGACA

AGCTGTGACCGTCTCCGGGAGCTGCATGTGTCAGAGGTTTTCACCGTCATCACCGAAACGCGCGAGGCAG

GGTGCCTTGATGTGGGCGCCGGCGGTCGAGTGGCGACGGCGCGGCTTGTCCGCGCCCTGGTAGATTGCCT

GGCCGTAGGCCAGCCATTTTTGAGCGGCCAGCGGCCGCGATAGGCCGACGCGAAGCGGCGGGGCGTAGGG

AGCGCAGCGACCGAAGGGTAGGCGCTTTTTGCAGCTCTTCGGCTGTGCGCTGGCCAGACAGTTATGCACA

GGCCAGGCGGGTTTTAAGAGTTTTAATAAGTTTTAAAGAGTTTTAGGCGGAAAAATCGCCTTTTTTCTCT

TTTATATCAGTCACTTACATGTGTGACCGGTTCCCAATGTACGGCTTTGGGTTCCCAATGTACGGGTTCC

GGTTCCCAATGTACGGCTTTGGGTTCCCAATGTACGTGCTATCCACAGGAAAGAGACCTTTTCGACCTTT

TTCCCCTGCTAGGGCAATTTGCCCTAGCATCTGCTCCGTACATTAGGAACCGGCGGATGCTTCGCCCTCG

ATCAGGTTGCGGTAGCGCATGACTAGGATCGGGCCAGCCTGCCCCGCCTCCTCCTTCAAATCGTACTCCG

GCAGGTCATTTGACCCGATCAGCTTGCGCACGGTGAAACAGAACTTCTTGAACTCTCCGGCGCTGCCACT

GCGTTCGTAGATCGTCTTGAACAACCATCTGGCTTCTGCCTTGCCTGCGGCGCGGCGTGCCAGGCGGTAG

AGAAAACGGCCGATGCCGGGATCGATCAAAAAGTAATCGGGGTGAACCGTCAGCACGTCCGGGTTCTTGC

CTTCTGTGATCTCGCGGTACATCCAATCAGCTAGCTCGATCTCGATGTACTCCGGCCGCCCGGTTTCGCT

CTTTACGATCTTGTAGCGGCTAATCAAGGCTTCACCCTCGGATACCGTCACCAGGCGGCCGTTCTTGGCC

TTCTTCGTACGCTGCATGGCAACGTGCGTGGTGTTTAACCGAATGCAGGTTTCTACCAGGTCGTCTTTCT

GCTTTCCGCCATCGGCTCGCCGGCAGAACTTGAGTACGTCCGCAACGTGTGGACGGAACACGCGGCCGGG

CTTGTCTCCCTTCCCTTCCCGGTATCGGTTCATGGATTCGGTTAGATGGGAAACCGCCATCAGTACCAGG

TCGTAATCCCACACACTGGCCATGCCGGCCGGCCCTGCGGAAACCTCTACGTGCCCGTCTGGAAGCTCGT

AGCGGATCACCTCGCCAGCTCGTCGGTCACGCTTCGACAGACGGAAAACGGCCACGTCCATGATGCTGCG

ACTATCGCGGGTGCCCACGTCATAGAGCATCGGAACGAAAAAATCTGGTTGCTCGTCGCCCTTGGGCGGC

TTCCTAATCGACGGCGCACCGGCTGCCGGCGGTTGCCGGGATTCTTTGCGGATTCGATCAGCGGCCGCTT

GCCACGATTCACCGGGGCGTGCTTCTGCCTCGATGCGTTGCCGCTGGGCGGCCTGCGCGGCCTTCAACTT

CTCCACCAGGTCATCACCCAGCGCCGCGCCGATTTGTACCGGGCCGGATGGTTTGCGACCGTCACGCCGA

TTCCTCGGGCTTGGGGGTTCCAGTGCCATTGCAGGGCCGGCAGACAACCCAGCCGCTTACGCCTGGCCAA

CCGCCCGTTCCTCCACACATGGGGCATTCCACGGCGTCGGTGCCTGGTTGTTCTTGATTTTCCATGCCGC

CTCCTTTAGCCGCTAAAATTCATCTACTCATTTATTCATTTGCTCATTTACTCTGGTAGCTGCGCGATGT

ATTCAGATAGCAGCTCGGTAATGGTCTTGCCTTGGCGTACCGCGTACATCTTCAGCTTGGTGTGATCCTC

CGCCGGCAACTGAAAGTTGACCCGCTTCATGGCTGGCGTGTCTGCCAGGCTGGCCAACGTTGCAGCCTTG

CTGCTGCGTGCGCTCGGACGGCCGGCACTTAGCGTGTTTGTGCTTTTGCTCATTTTCTCTTTACCTCATT

AACTCAAATGAGTTTTGATTTAATTTCAGCGGCCAGCGCCTGGACCTCGCGGGCAGCGTCGCCCTCGGGT

TCTGATTCAAGAACGGTTGTGCCGGCGGCGGCAGTGCCTGGGTAGCTCACGCGCTGCGTGATACGGGACT

CAAGAATGGGCAGCTCGTACCCGGCCAGCGCCTCGGCAACCTCACCGCCGATGCGCGTGCCTTTGATCGC

CCGCGACACGACAAAGGCCGCTTGTAGCCTTCCATCCGTGACCTCAATGCGCTGCTTAACCAGCTCCACC

AGGTCGGCGGTGGCCCATATGTCGTAAGGGCTTGGCTGCACCGGAATCAGCACGAAGTCGGCTGCCTTGA

TCGCGGACACAGCCAAGTCCGCCGCCTGGGGCGCTCCGTCGATCACTACGAAGTCGCGCCGGCCGATGGC

CTTCACGTCGCGGTCAATCGTCGGGCGGTCGATGCCGACAACGGTTAGCGGTTGATCTTCCCGCACGGCC

GCCCAATCGCGGGCACTGCCCTGGGGATCGGAATCGACTAACAGAACATCGGCCCCGGCGAGTTGCAGGG

CGCGGGCTAGATGGGTTGCGATGGTCGTCTTGCCTGACCCGCCTTTCTGGTTAAGTACAGCGATAACCTT

CATGCGTTCCCCTTGCGTATTTGTTTATTTACTCATCGCATCATATACGCAGCGACCGCATGACGCAAGC

TGTTTTACTCAAATACACATCACCTTTTTAGACGGCGGCGCTCGGTTTCTTCAGCGGCCAAGCTGGCCGG

CCAGGCCGCCAGCTTGGCATCAGACAAACCGGCCAGGATTTCATGCAGCCGCACGGTTGAGACGTGCGCG

GGCGGCTCGAACACGTACCCGGCCGCGATCATCTCCGCCTCGATCTCTTCGGTAATGAAAAACGGTTCGT

CCTGGCCGTCCTGGTGCGGTTTCATGCTTGTTCCTCTTGGCGTTCATTCTCGGCGGCCGCCAGGGCGTCG

GCCTCGGTCAATGCGTCCTCACGGAAGGCACCGCGCCGCCTGGCCTCGGTGGGCGTCACTTCCTCGCTGC

GCTCAAGTGCGCGGTACAGGGTCGAGCGATGCACGCCAAGCAGTGCAGCCGCCTCTTTCACGGTGCGGCC

TTCCTGGTCGATCAGCTCGCGGGCGTGCGCGATCTGTGCCGGGGTGAGGGTAGGGCGGGGGCCAAACTTC

ACGCCTCGGGCCTTGGCGGCCTCGCGCCCGCTCCGGGTGCGGTCGATGATTAGGGAACGCTCGAACTCGG

CAATGCCGGCGAACACGGTCAACACCATGCGGCCGGCCGGCGTGGTGGTGTCGGCCCACGGCTCTGCCAG

GCTACGCAGGCCCGCGCCGGCCTCCTGGATGCGCTCGGCAATGTCCAGTAGGTCGCGGGTGCTGCGGGCC

AGGCGGTCTAGCCTGGTCACTGTCACAACGTCGCCAGGGCGTAGGTGGTCAAGCATCCTGGCCAGCTCCG

GGCGGTCGCGCCTGGTGCCGGTGATCTTCTCGGAAAACAGCTTGGTGCAGCCGGCCGCGTGCAGTTCGGC

CCGTTGGTTGGTCAAGTCCTGGTCGTCGGTGCTGACGCGGGCATAGCCCAGCAGGCCAGCGGCGGCGCTC

TTGTTCATGGCGTAATGTCTCCGGTTCTAGTCGCAAGTATTCTACTTTATGCGACTAAAACACGCGACAA

GAAAACGCCAGGAAAAGGGCAGGGCGGCAGCCTGTCGCGTAACTTAGGACTTGTGCGACATGTCGTTTTC

AGAAGACGGCTGCACTGAACGTCAGAAGCCGACTGCACTATAGCAGCGGAGGGGTTGGATCAAAGTACTT

TGATCCCGAGGGGAACCCTGTGGTTGGCATGCACATACAAATGGACGAACGGATAAACCTTTTCACGCCC

TTTTAAATATCCGTTATTCTAATAAACGCTCTTTTCTCTTAG

>gi|12831187|gb|AF330636.1|AF330636 Plant DNA excision vector pX6-GFP, complete sequence

GTTTACCCGCCAATATATCCTGTCAAACACTGATAGTTTAAACTGAAGGCGGGAAACGACAATCTGATCC

AAGCTCAAGCTAAGCTTGCATGCCTGCAGGATATCGTGGATCCAAGCTTGCCACGTGCCGCCACGTGCCG

CCACGTGCCGCCACGTGCCTCTAGAGGATCCATCTCCACTGACGTAAGGGATGACGCACAATCCCACTAT

CCTTCGCAAGACCCTTCCTCTATATAAGGAAGTTCATTTCATTTGGAGAGGACACGCTGGGATCCCCTAA

TAACTTCGTATAGCATACATTATACGAAGTTATGAATTAAATCCGGGCGGAATGAAAGCGTTAACGGCCA

GGCAACAAGAGGTGTTTGATCTCATCCGTGATCACATCAGCCAGACAGGTATGCCGCCGACGCGTGCGGA

AATCGCGCAGCGTTTGGGGTTCCGTTCCCCAAACGCGGCTGAAGAACATCTGAAGGCGCTGGCACGCAAA

GGCGTTATTGAAATTGTTTCCGGCGCATCACGCGGGATTCGTCTGTTGCAGGAAGAGGAAGAAGGGTTGC

CGCTGGTAGGTCGTGTGGCTGCCGGTGAACCGTCGAGCGCCCCCCCGACCGATGTCAGCCTGGGGGACGA

GCTCCACTTAGACGGCGAGGACGTGGCGATGGCGCATGCCGACGCGCTAGACGATTTCGATCTGGACATG

TTGGGGGACGGGGATTCCCCGGGTCCGGGATTTACCCCCCACGACTCCGCCCCCTACGGCGCTCTGGATA

TGGCCGACTTCGAGTTTGAGCAGATGTTTACCGATGCCCTTGGAATTGACGAGTACGGTGGGGATCCGTC

TGCTGGAGACATGAGAGCTGCCAACCTTTGGCCAAGCCCGCTCATGATCAAACGCTCTAAGAAGAACAGC

CTGGCCTTGTCCCTGACGGCCGACCAGATGGTCAGTGCCTTGTTGGATGCTGAGCCCCCCATACTCTATT

CCGAGTATGATCCTACCAGACCCTTCAGTGAAGCTTCGATGATGGGCTTACTGACCAACCTGGCAGACAG

GGAGCTGGTTCACATGATCAACTGGGCGAAGAGGGTGCCAGGCTTTGTGGATTTGACCCTCCATGATCAG

GTCCACCTTCTAGAATGTGCCTGGCTAGAGATCCTGATGATTGGTCTCGTCTGGCGCTCCATGGAGCACC

CAGTGAAGCTACTGTTTGCTCCTAACTTGCTCTTGGACAGGAACCAGGGAAAATGTGTAGAGGGCATGGT

GGAGATCTTCGACATGCTGCTGGCTACATCATCTCGGTTCCGCATGATGAATCTGCAGGGAGAGGAGTTT

GTGTGCCTCAAATCTATTATTTTGCTTAATTCTGGAGTGTACACATTTCTGTCCAGCACCCTGAAGTCTC

TGGAAGAGAAGGACCATATCCACCGAGTCCTGGACAAGATCACAGACACTTTGATCCACCTGATGGCCAA

GGCAGGCCTGACCCTGCAGCAGCAGCACCAGCGGCTGGCCCAGCTCCTCCTCATCCTCTCCCACATCAGG

CACATGAGTAACAAAGGCATGGAGCATCTGTACAGCATGAAGTGCAAGAACGTGGTGCCCCTCTATGACC

TGCTGCTGGAGATGCTGGACGCCCACCGCCTACATGCGCCCACTAGCCGTGGAGGGGCATCCGTGGAGGA

GACGGACCAAAGCCACTTGGCCACTGCGGGCTCTACTTCATCGCATTCCTTGCAAAAGTATTACATCACG

GGGGAGGCAGAGGGTTTCCCTGCCACAGTCTGAGAGCTCCCTGGCGAATTCCCAGAGATGTTAGCTGAAA

TCATCACTAATCAGATACCAAAATATTCAAATGGAAATATCAAAAAGCTTCTGTTTCATCAAAAATGACT

CGACCTAACTGAGTAAGCTAGCTTGTTCGAGTATTATGGCATTGGGAAAACTGTTTTTCTTGTACCATTT

GTTGTGCTTGTAATTTACTGTGTTTTTTATTCGGTTTTCGCTATCGAACTGTGAAATGGAAATGGATGGA

GAAGAGTTAATGAATGATATGGTCCTTTTGTTCATTCTCAAATTAATATTATTTGTTTTTTCTCTTATTT

GTTGTGTGTTGAATTTGAAATTATAAGAGATATGCAAACATTTTGTTTTGAGTAAAAATGTGTCAAATCG

TGGCCTCTAATGACCGAAGTTAATATGAGGAGTAAAACATCCCAAACAAGCTTGGAAACTGAAGGCGGGA

AACGACAATCTGATCATGAGCGGAGAATTAAGGGAGTCACGTTATGACCCCCGCCGATGACGCGGGACAA

GCCGTTTTACGTTTGGAACTGACAGAACCGCAACGATTGAAGGAGCCACTCAGCCGCGGGTTTCTGGAGT

TTAATGAGCTAAGCACATACGTCAGAAACCATTATTGCGCGTTCAAAAGTCGCCTAAGGTCACTATCAGC

TAGCAAATATTTCTTGTCAAAAATGCTCCACTGACGTTCCATAAATTCCCCTCGGTATCCAATTAGAGTC

TCATATTCACTCTCAATCCAAATAATCTGCACCGGATCTGGATCGTTTCGCATGATTGAACAAGATGGAT

TGCACGCAGGTTCTCCGGCCGCTTGGGTGGAGAGGCTATTCGGCTATGACTGGGCACAACAGACAATCGG

CTGCTCTGATGCCGCCGTGTTCCGGCTGTCAGCGCAGGGGCGCCCGGTTCTTTTTGTCAAGACCGACCTG

TCCGGTGCCCTGAATGAACTGCAGGACGAGGCAGCGCGGCTATCGTGGCTGGCCACGACGGGCGTTCCTT

GCGCAGCTGTGCTCGACGTTGTCACTGAAGCGGGAAGGGACTGGCTGCTATTGGGCGAAGTGCCGGGGCA

GGATCTCCTGTCATCTCACCTTGCTCCTGCCGAGAAAGTATCCATCATGGCTGATGCAATGCGGCGGCTG

CATACGCTTGATCCGGCTACCTGCCCATTCGACCACCAAGCGAAACATCGCATCGAGCGAGCACGTACTC

GGATGGAAGCCGGTCTTGTCGATCAGGATGATCTGGACGAAGAGCATCAGGGGCTCGCGCCAGCCGAACT

GTTCGCCAGGCTCAAGGCGCGCATGCCCGACGGCGATGATCTCGTCGTGACCCATGGCGATGCCTGCTTG

CCGAATATCATGGTGGAAAATGGCCGCTTTTCTGGATTCATCGACTGTGGCCGGCTGGGTGTGGCGGACC

GCTATCAGGACATAGCGTTGGCTACCCGTGATATTGCTGAAGAGCTTGGCGGCGAATGGGCTGACCGCTT

CCTCGTGCTTTACGGTATCGCCGCTCCCGATTCGCAGCGCATCGCCTTCTATCGCCTTCTTGACGAGTTC

TTCTGAGCGGGACTCTGGGGTTCGAAATGACCGACCAAGCGACGCCCAACCTGCCATCACGAGATTTCGA

TTCCACCGCCGCCTTCTATGAAAGGTTGGGCTTCGGAATCGTTTTCCGGGACGCCGGCTGGATGATCCTC

CAGCGCGGGGATCTCATGCTGGAGTTCTTCGCCCACGGGATCTCTGCGGAACAGGCGGTCGAAGGTGCCG

ATATCATTACGACAGCAACGGCCGACAAGCACAACGCCACGATCCTGAGCGACAATATGATCGGGCCCGG

CGTCCACATCAACGGCGTCGGCGGCGACTGCCCAGGCAAGACCGAGATGCACCGCGATATCTTGCTGCGT

TCGGATATTTTCGTGGAGTTCCCGCCACAGACCCGGATGATCCCCGATCGTTCAAACATTTGGCAATAAA

GTTTCTTAAGATTGAATCCTGTTGCCGGTCTTGCGATGATTATCATATAATTTCTGTTGAATTACGTTAA

GCATGTAATAATTAACATGTAATGCATGACGTTATTTATGAGATGGGTTTTTATGATTAGAGTCCCGCAA

TTATACATTTAATACGCGATAGAAAACAAAATATAGCGCGCAAACTAGGATAAATTATCGCGCGCGGTGT

CATCTATGTTACTAGATCGGGGAATTGATCCCCCCTCGACAGCTTGCATGCCAGCTTGGGCTGCAGGTCG

AGGCTAAAAAACTAATCGCATTATCATCCCCTCGACGTACTGTACATATAACCACTGGTTTTATATACAG

CAGTACTGTACATATAACCACTGGTTTTATATACAGCAGTCGACGTACTGTACATATAACCACTGGTTTT

ATATACAGCAGTACTGTACATATAACCACTGGTTTTATATACAGCAGTCGAGGTAAGATTAGATATGGAT

ATGTATATGGATATGTATATGGTGGTAATGCCATGTAATATGCTCGACTCTAGGATCTTCGCAAGACCCT

TCCTCTATATAAGGAAGTTCATTTCATTTGGAGAGGACACGCTGAAGCTAGTCGACTCTAGCCTCGACAT

GTCCAATTTACTGACCGTACACCAAAATTTGCCTGCATTACCGGTCGATGCAACGAGTGATGAGGTTCGC

AAGAACCTGATGGACATGTTCAGGGATCGCCAGGCGTTTTCTGAGCATACCTGGAAAATGCTTCTGTCCG

TTTGCCGGTCGTGGGCGGCATGGTGCAAGTTGAATAACCGGAAATGGTTTCCCGCAGAACCTGAAGATGT

TCGCGATTATCTTCTATATCTTCAGGCGCGCGGTCTGGCAGTAAAAACTATCCAGCAACATTTGGGCCAG

CTAAACATGCTTCATCGTCGGTCCGGGCTGCCACGACCAAGTGACAGCAATGCTGTTTCACTGGTTATGC

GGCGGATCCGAAAAGAAAACGTTGATGCCGGTGAACGTGCAAAACAGGCTCTAGCGTTCGAACGCACTGA

TTTCGACCAGGTAAGTCTTCTTTTCCTTTACTCTTTACAGAAATGGTAATCTCAGATATAGTAATGGATA

AGATCCAAAAATGACACTTTTAACCAAGATTGTACGAAGATCTTTTTAAACTCCATTTTTTATTTTGACA

TCTAAATTGGATTTAACTCGGCCTTGCTGTATTTTGGCAGGTTCGTTCACTCATGGAAAATAGCGATCGC

TGCCAGGATATACGTAATCTGGCATTTCTGGGGATTGCTTATAACACCCTGTTACGTATAGCCGAAATTG

CCAGGATCAGGGTTAAAGATATCTCACGTACTGACGGTGGGAGAATGTTAATCCATATTGGCAGAACGAA

AACGCTGGTTAGCACCGCAGGTGTAGAGAAGGCACTTAGCCTGGGGGTAACTAAACTGGTCGAGCGATGG

ATTTCCGTCTCTGGTGTAGCTGATGATCCGAATAACTACCTGTTTTGCCGGGTCAGAAAAAATGGTGTTG

CCGCGCCATCTGCCACCAGCCAGCTATCAACTCGCGCCCTGGAAGGGATTTTTGAAGCAACTCATCGATT

GATTTACGGCGCTAAGGATGACTCTGGTCAGAGATACCTGGCCTGGTCTGGACACAGTGCCCGTGTCGGA

GCCGCGCGAGATATGGCCCGCGCTGGAGTTTCAATACCGGAGATCATGCAAGCTGGTGGCTGGACCAATG

TAAATATTGTCATGAACTATATCCGTAACCTGGATAGTGAAACAGGGGCAATGGTGCGCCTGCTGGAAGA

TGGCGATTAGTAAGAATTCGCATGCATTCATCAATATTATTCATGCGGGGAAAGGCAAGATTAATCCAAC

TGGCAAATCATCCAGCGTGATTGGTAACTTCAGTTCCAGCGACTTGATTCGTTTTGGTGCTACCCACGTT

TTCAATAAGGACGAGATGGTGGAGTAAAGAAGGAGTGCGTCGAAGCAGATCGTTCAAACATTTGGCAATA

AAGTTTCTTAAGATTGAATCCTGTTGCCGGTCTTGCGATGATTATCATATAATTTCTGTTGAATTACGTT

AAGCATGTAATAATTAACATGTAATGCATGACGTTATTTATGAGATGGGTTTTTATGATTAGAGTCCCGC

AATTATACATTTAATACGCGATAGAAAACAAAATATAGCGCGCAAACTAGGATAAATTATCGCGCGCGGT

GTCATCTATGTTACTAGATCATAACTTCGTATAGCATACATTATACGAAGTTATAGATCTTCGACCTCGA

GCGGGGTACCATGGGTAAGGGAGAAGAACTTTTCACTGGAGTTGTCCCAATTCTTGTTGAATTAGATGGT

GATGTTAATGGGCACAAATTTTCTGTCAGTGGAGAGGGTGAAGGTGATGCAACATACGGAAAACTTACCC

TTAAATTTATTTGCACTACTGGAAAGCTTCCTGTTCCTTGGCCAACACTTGTCACTACTCTTACTTATGG

TGTTCAATGCTTTTCAAGATACCCAGATCATATGAAGCGGCACGACTTCTTCAAGAGCGCCATGCCTGAG

GGATACGTGCAGGAAAGGACCATCTTCTTCAAGGACGACGGGAACTACAAGACACGTGCTGAAGTCAAGT

TTGAGGGAGACACCCTTGTCAACAGGATCGAGCTTAAGGGAATCGATTTCAAGGAGGACGGAAACATCCT

CGGCCACAAGTTGGAATACAACTACAACTCCCACAACGTATACATCATGGCAGACAAACAAAAGAATGGA

ATCAAAGTTAACTTCAAAATTAGACACAACATTGAAGATGGAAGCGTTCAACTAGCAGACCATTATCAAC

AAAATACTCCAATTGGCGATGGCCCTGTCCTTTTACCAGACAACCATTACCTGTCCACACAATCTGCCCT

TTCGAAAGATCCCAACGAAAAGAGAGACCACATGGTCCTTCTTGAGTTTGTAACAGCTGCTGGGATTACA

CATGGCATGGATGAACTATACAAATAAACTAGTCGATCCAGGCCTCCCAGCTTTCGTCCGTATCATCGGT

TTCGACAACGTTCGTCAAGTTCAATGCATCAGTTTCATTGCCCACACACCAGAATCCTACTAAGTTTGAG

TATTATGGCATTGGAAAAGCTGTTTTCTTCTATCATTTGTTCTGCTTGTAATTTACTGTGTTCTTTCAGT

TTTTGTTTTCGGACATCAAAATGCAAATGGATGGATAAGAGTTAATAAATGATATGGTCCTTTTGTTCAT

TCTCAAATTATTATTATCTGTTGTTTTTACTTTAATGGGTTGAATTTAAGTAAGAAAGGAACTAACAGTG

TGATATTAAGGTGCAATGTTAGACATATAAAACAGTCTTTCACCTCTCTTTGGTTATGTCTTGAATTGGT

TTGTTTCTTCACTTATCTGTGTAATCAAGTTTACTATGAGTCTATGATCAAGTAATTATGCAATCAAGTT

AAGTACAGTATAGGCTTTTTGTGTCGAGGGGGTACCGAGTCGAGGAATTCACTGGCCGTCGTTTTACAAC

GTCGTGACTGGGAAAACCCTGGCGTTACCCAACTTAATCGCCTTGCAGCACATCCCCCTTTCGCCAGCTG

GCGTAATAGCGAAGAGGCCCGCACCGATCGCCCTTCCCAACAGTTGCGCAGCCTGAATGGCGGGTACCGA

GCTCGAATTCAATTCGGCGTTAATTCAGTACATTAAAAACGTCCGCAATGTGTTATTAAGTTGTCTAAGC

GTCAATTTGTTTACACCACAATATATCCTGCCACCAGCCAGCCAACAGCTCCCCGACCGGCAGCTCGGCA

CAAAATCACCACTCGATACAGGCAGCCCATCAGTCCGGGACGGCGTCAGCGGGAGAGCCGTTGTAAGGCG

GCAGACTTTGCTCATGTTACCGATGCTATTCGGAAGAACGGCAACTAAGCTGCCGGGTTTGAAACACGGA

TGATCTCGCGGAGGGTAGCATGTTGATTGTAACGATGACAGAGCGTTGCTGCCTGTGATCAATTCGGGCA

CGAACCCAGTGGACATAAGCCTCGTTCGGTTCGTAAGCTGTAATGCAAGTAGCGTAACTGCCGTCACGCA

ACTGGTCCAGAACCTTGACCGAACGCAGCGGTGGTAACGGCGCAGTGGCGGTTTTCATGGCTTCTTGTTA

TGACATGTTTTTTTGGGGTACAGTCTATGCCTCGGGCATCCAAGCAGCAAGCGCGTTACGCCGTGGGTCG

ATGTTTGATGTTATGGAGCAGCAACGATGTTACGCAGCAGGGCAGTCGCCCTAAAACAAAGTTAAACATC

ATGGGGGAAGCGGTGATCGCCGAAGTATCGACTCAACTATCAGAGGTAGTTGGCGTCATCGAGCGCCATC

TCGAACCGACGTTGCTGGCCGTACATTTGTACGGCTCCGCAGTGGATGGCGGCCTGAAGCCACACAGTGA

TATTGATTTGCTGGTTACGGTGACCGTAAGGCTTGATGAAACAACGCGGCGAGCTTTGATCAACGACCTT

TTGGAAACTTCGGCTTCCCCTGGAGAGAGCGAGATTCTCCGCGCTGTAGAAGTCACCATTGTTGTGCACG

ACGACATCATTCCGTGGCGTTATCCAGCTAAGCGCGAACTGCAATTTGGAGAATGGCAGCGCAATGACAT

TCTTGCAGGTATCTTCGAGCCAGCCACGATCGACATTGATCTGGCTATCTTGCTGACAAAAGCAAGAGAA

CATAGCGTTGCCTTGGTAGGTCCAGCGGCGGAGGAACTCTTTGATCCGGTTCCTGAACAGGATCTATTTG

AGGCGCTAAATGAAACCTTAACGCTATGGAACTCGCCGCCCGACTGGGCTGGCGATGAGCGAAATGTAGT

GCTTACGTTGTCCCGCATTTGGTACAGCGCAGTAACCGGCAAAATCGCGCCGAAGGATGTCGCTGCCGAC

TGGGCAATGGAGCGCCTGCCGGCCCAGTATCAGCCCGTCATACTTGAAGCTAGACAGGCTTATCTTGGAC

AAGAAGAAGATCGCTTGGCCTCGCGCGCAGATCAGTTGGAAGAATTTGTCCACTACGTGAAAGGCGAGAT

CACCAAGGTAGTCGGCAAATAATGTCTAGCTAGAAATTCGTTCAAGCCGACGCCGCTTCGCCGGCGTTAA

CTCAAGCGATTAGATGCACTAAGCACATAATTGCTCACAGCCAAACTATCAGGTCAAGTCTGCTTTTATT

ATTTTTAAGCGTGCATAATAAGCCCTACACAAATTGGGAGATATATCATGCATGACCAAAATCCCTTAAC

GTGAGTTTTCGTTCCACTGAGCGTCAGACCCCGTAGAAAAGATCAAAGGATCTTCTTGAGATCCTTTTTT

TCTGCGCGTAATCTGCTGCTTGCAAACAAAAAAACCACCGCTACCAGCGGTGGTTTGTTTGCCGGATCAA

GAGCTACCAACTCTTTTTCCGAAGGTAACTGGCTTCAGCAGAGCGCAGATACCAAATACTGTCCTTCTAG

TGTAGCCGTAGTTAGGCCACCACTTCAAGAACTCTGTAGCACCGCCTACATACCTCGCTCTGCTAATCCT

GTTACCAGTGGCTGCTGCCAGTGGCGATAAGTCGTGTCTTACCGGGTTGGACTCAAGACGATAGTTACCG

GATAAGGCGCAGCGGTCGGGCTGAACGGGGGGTTCGTGCACACAGCCCAGCTTGGAGCGAACGACCTACA

CCGAACTGAGATACCTACAGCGTGAGCTATGAGAAAGCGCCACGCTTCCCGAAGGGAGAAAGGCGGACAG

GTATCCGGTAAGCGGCAGGGTCGGAACAGGAGAGCGCACGAGGGAGCTTCCAGGGGGAAACGCCTGGTAT

CTTTATAGTCCTGTCGGGTTTCGCCACCTCTGACTTGAGCGTCGATTTTTGTGATGCTCGTCAGGGGGGC

GGAGCCTATGGAAAAACGCCAGCAACGCGGCCTTTTTACGGTTCCTGGCCTTTTGCTGGCCTTTTGCTCA

CATGTTCTTTCCTGCGTTATCCCCTGATTCTGTGGATAACCGTATTACCGCCTTTGAGTGAGCTGATACC

GCTCGCCGCAGCCGAACGACCGAGCGCAGCGAGTCAGTGAGCGAGGAAGCGGAAGAGCGCCTGATGCGGT

ATTTTCTCCTTACGCATCTGTGCGGTATTTCACACCGCATATGGTGCACTCTCAGTACAATCTGCTCTGA

TGCCGCATAGTTAAGCCAGTATACACTCCGCTATCGCTACGTGACTGGGTCATGGCTGCGCCCCGACACC

CGCCAACACCCGCTGACGCGCCCTGACGGGCTTGTCTGCTCCCGGCATCCGCTTACAGACAAGCTGTGAC

CGTCTCCGGGAGCTGCATGTGTCAGAGGTTTTCACCGTCATCACCGAAACGCGCGAGGCAGGGTGCCTTG

ATGTGGGCGCCGGCGGTCGAGTGGCGACGGCGCGGCTTGTCCGCGCCCTGGTAGATTGCCTGGCCGTAGG

CCAGCCATTTTTGAGCGGCCAGCGGCCGCGATAGGCCGACGCGAAGCGGCGGGGCGTAGGGAGCGCAGCG

ACCGAAGGGTAGGCGCTTTTTGCAGCTCTTCGGCTGTGCGCTGGCCAGACAGTTATGCACAGGCCAGGCG

GGTTTTAAGAGTTTTAATAAGTTTTAAAGAGTTTTAGGCGGAAAAATCGCCTTTTTTCTCTTTTATATCA

GTCACTTACATGTGTGACCGGTTCCCAATGTACGGCTTTGGGTTCCCAATGTACGGGTTCCGGTTCCCAA

TGTACGGCTTTGGGTTCCCAATGTACGTGCTATCCACAGGAAAGAGACCTTTTCGACCTTTTTCCCCTGC

TAGGGCAATTTGCCCTAGCATCTGCTCCGTACATTAGGAACCGGCGGATGCTTCGCCCTCGATCAGGTTG

CGGTAGCGCATGACTAGGATCGGGCCAGCCTGCCCCGCCTCCTCCTTCAAATCGTACTCCGGCAGGTCAT

TTGACCCGATCAGCTTGCGCACGGTGAAACAGAACTTCTTGAACTCTCCGGCGCTGCCACTGCGTTCGTA

GATCGTCTTGAACAACCATCTGGCTTCTGCCTTGCCTGCGGCGCGGCGTGCCAGGCGGTAGAGAAAACGG

CCGATGCCGGGATCGATCAAAAAGTAATCGGGGTGAACCGTCAGCACGTCCGGGTTCTTGCCTTCTGTGA

TCTCGCGGTACATCCAATCAGCTAGCTCGATCTCGATGTACTCCGGCCGCCCGGTTTCGCTCTTTACGAT

CTTGTAGCGGCTAATCAAGGCTTCACCCTCGGATACCGTCACCAGGCGGCCGTTCTTGGCCTTCTTCGTA

CGCTGCATGGCAACGTGCGTGGTGTTTAACCGAATGCAGGTTTCTACCAGGTCGTCTTTCTGCTTTCCGC

CATCGGCTCGCCGGCAGAACTTGAGTACGTCCGCAACGTGTGGACGGAACACGCGGCCGGGCTTGTCTCC

CTTCCCTTCCCGGTATCGGTTCATGGATTCGGTTAGATGGGAAACCGCCATCAGTACCAGGTCGTAATCC

CACACACTGGCCATGCCGGCCGGCCCTGCGGAAACCTCTACGTGCCCGTCTGGAAGCTCGTAGCGGATCA

CCTCGCCAGCTCGTCGGTCACGCTTCGACAGACGGAAAACGGCCACGTCCATGATGCTGCGACTATCGCG

GGTGCCCACGTCATAGAGCATCGGAACGAAAAAATCTGGTTGCTCGTCGCCCTTGGGCGGCTTCCTAATC

GACGGCGCACCGGCTGCCGGCGGTTGCCGGGATTCTTTGCGGATTCGATCAGCGGCCGCTTGCCACGATT

CACCGGGGCGTGCTTCTGCCTCGATGCGTTGCCGCTGGGCGGCCTGCGCGGCCTTCAACTTCTCCACCAG

GTCATCACCCAGCGCCGCGCCGATTTGTACCGGGCCGGATGGTTTGCGACCGTCACGCCGATTCCTCGGG

CTTGGGGGTTCCAGTGCCATTGCAGGGCCGGCAGACAACCCAGCCGCTTACGCCTGGCCAACCGCCCGTT

CCTCCACACATGGGGCATTCCACGGCGTCGGTGCCTGGTTGTTCTTGATTTTCCATGCCGCCTCCTTTAG

CCGCTAAAATTCATCTACTCATTTATTCATTTGCTCATTTACTCTGGTAGCTGCGCGATGTATTCAGATA

GCAGCTCGGTAATGGTCTTGCCTTGGCGTACCGCGTACATCTTCAGCTTGGTGTGATCCTCCGCCGGCAA

CTGAAAGTTGACCCGCTTCATGGCTGGCGTGTCTGCCAGGCTGGCCAACGTTGCAGCCTTGCTGCTGCGT

GCGCTCGGACGGCCGGCACTTAGCGTGTTTGTGCTTTTGCTCATTTTCTCTTTACCTCATTAACTCAAAT

GAGTTTTGATTTAATTTCAGCGGCCAGCGCCTGGACCTCGCGGGCAGCGTCGCCCTCGGGTTCTGATTCA

AGAACGGTTGTGCCGGCGGCGGCAGTGCCTGGGTAGCTCACGCGCTGCGTGATACGGGACTCAAGAATGG

GCAGCTCGTACCCGGCCAGCGCCTCGGCAACCTCACCGCCGATGCGCGTGCCTTTGATCGCCCGCGACAC

GACAAAGGCCGCTTGTAGCCTTCCATCCGTGACCTCAATGCGCTGCTTAACCAGCTCCACCAGGTCGGCG

GTGGCCCATATGTCGTAAGGGCTTGGCTGCACCGGAATCAGCACGAAGTCGGCTGCCTTGATCGCGGACA

CAGCCAAGTCCGCCGCCTGGGGCGCTCCGTCGATCACTACGAAGTCGCGCCGGCCGATGGCCTTCACGTC

GCGGTCAATCGTCGGGCGGTCGATGCCGACAACGGTTAGCGGTTGATCTTCCCGCACGGCCGCCCAATCG

CGGGCACTGCCCTGGGGATCGGAATCGACTAACAGAACATCGGCCCCGGCGAGTTGCAGGGCGCGGGCTA

GATGGGTTGCGATGGTCGTCTTGCCTGACCCGCCTTTCTGGTTAAGTACAGCGATAACCTTCATGCGTTC

CCCTTGCGTATTTGTTTATTTACTCATCGCATCATATACGCAGCGACCGCATGACGCAAGCTGTTTTACT

CAAATACACATCACCTTTTTAGACGGCGGCGCTCGGTTTCTTCAGCGGCCAAGCTGGCCGGCCAGGCCGC

CAGCTTGGCATCAGACAAACCGGCCAGGATTTCATGCAGCCGCACGGTTGAGACGTGCGCGGGCGGCTCG

AACACGTACCCGGCCGCGATCATCTCCGCCTCGATCTCTTCGGTAATGAAAAACGGTTCGTCCTGGCCGT

CCTGGTGCGGTTTCATGCTTGTTCCTCTTGGCGTTCATTCTCGGCGGCCGCCAGGGCGTCGGCCTCGGTC

AATGCGTCCTCACGGAAGGCACCGCGCCGCCTGGCCTCGGTGGGCGTCACTTCCTCGCTGCGCTCAAGTG

CGCGGTACAGGGTCGAGCGATGCACGCCAAGCAGTGCAGCCGCCTCTTTCACGGTGCGGCCTTCCTGGTC

GATCAGCTCGCGGGCGTGCGCGATCTGTGCCGGGGTGAGGGTAGGGCGGGGGCCAAACTTCACGCCTCGG

GCCTTGGCGGCCTCGCGCCCGCTCCGGGTGCGGTCGATGATTAGGGAACGCTCGAACTCGGCAATGCCGG

CGAACACGGTCAACACCATGCGGCCGGCCGGCGTGGTGGTGTCGGCCCACGGCTCTGCCAGGCTACGCAG

GCCCGCGCCGGCCTCCTGGATGCGCTCGGCAATGTCCAGTAGGTCGCGGGTGCTGCGGGCCAGGCGGTCT

AGCCTGGTCACTGTCACAACGTCGCCAGGGCGTAGGTGGTCAAGCATCCTGGCCAGCTCCGGGCGGTCGC

GCCTGGTGCCGGTGATCTTCTCGGAAAACAGCTTGGTGCAGCCGGCCGCGTGCAGTTCGGCCCGTTGGTT

GGTCAAGTCCTGGTCGTCGGTGCTGACGCGGGCATAGCCCAGCAGGCCAGCGGCGGCGCTCTTGTTCATG

GCGTAATGTCTCCGGTTCTAGTCGCAAGTATTCTACTTTATGCGACTAAAACACGCGACAAGAAAACGCC

AGGAAAAGGGCAGGGCGGCAGCCTGTCGCGTAACTTAGGACTTGTGCGACATGTCGTTTTCAGAAGACGG

CTGCACTGAACGTCAGAAGCCGACTGCACTATAGCAGCGGAGGGGTTGGATCAAAGTACTTTGATCCCGA

GGGGAACCCTGTGGTTGGCATGCACATACAAATGGACGAACGGATAAACCTTTTCACGCCCTTTTAAATA

TCCGTTATTCTAATAAACGCTCTTTTCTCTTAG

>gi|13359266|dbj|AB055064.1| Synthetic transposable element dAc-I-RS DNA, complete sequence

CAGGGATGAAAGTAGGATGGGAAAATCCCGTACCGACCGTTATCGTATAACCGATTTTGTTAGTTTTATC

CCGATCGATTTCGAACCCGAGGTAAAAAACGAAAACGGAACGGAAACGGGATATACAAAACGGTAAACGG

AAACGGAAACGGTAGAGCTAGTTTCCCGACCGTTTCACCGGGATCCCGTTTTTAATCGGGATGATCCCGT

TTCGTTACCGTATTTTCTAATTCGGGATGACTGCAATATGGCCAGCTCCAACTCCCATCCATAACCACTG

AGGCCCAGCCCATGTAAGAAATACCTAGCGAACGCTGCTCTGCCTCTCTCCCAGGCGGCCAGGCACCACA

CGAGTAACAGCATCACACATTCACACGCCGCCACGCGCCCACGCCGGAGTCCGGACGCCGCCAGCCGCAC

GCCGACGCCGGCGACGCGTCTCGCTCTCGCCTGCTCTCTCCGACTCTCCCTGTCTCCCAGCCGGCCGGCC

GCTGGGCTGCACCAGGCACCACACGCGGTGACGGCCGTGACGCGGCACGCCGGACGCAGACGCCGCCATC

CACGGTCCGCCCTCCACTCCACTGCTCGCGACTCGCCCATCCGCGCCGCGGTCCGCCCATCCGCCCAGAC

CTCCACTCCACTGCTCGCCCATCCGCGGTCCGCCCATCCGCCATCCGCCATCTGCGGTCAGCGGTCCTCC

AGACCTCCACTGCTCGGCGCTCGCCCATCCGGCCATCCGCGGTCTCCCTGTCTCCACGGCTGCTCACAGG

CTCACAGCACTTAGCAGTACAGCACGTCAGCACCATTGCACCAAGCTGTTGTGTCATTTGTGTGCTGTCC

AGGGGCTCTGCAACACCTGCTGATTGCTGTCCAGCCGTCCAGGTGCTCACAAGTCACAGCAGTACAGCAC

CAAGCTGATTGCTGAACACCTGCTGTCCAGGGCTCTGCTCTCCACTTCGGCTAGCCGGCTACGACTCCAT

TCCTCAGATGACGCCTCCGGTTGGAAATAATCCTCCCTCAGGCTCAGCCATAAGATTGGCCAAGTTGATG

TCTACCACAAGAGCGCCTTCTACTCGCAAAACAAATTCCGTATTCTCTGCATATGCTCAAGGTATATATT

AGAAAAACAGTAGCAATAGCATTAGCATTACTAATTGGTTGTAGATTGGGAAGCATCATATTGACTGTAG

AATAATACGAAAAATCTGTTTATAACAGGGTTGAAAAGAAAAGCTGAAGCCTCTTCTAGTCGGATTCAGA

ATGTACGTGCACGTGCGCGTGGGCATGGATGTGGCCGCACATCACCATCATCATCAACAGCTGAGGCCGA

GAGGCATTTTATTCAGAGTGTAAGCAGTAGTAATGCAAATGGTACAGCTACAGATCCGAGTCAAGATGAT

ATGGCTATTGTTCATGAACCACAACCACAACCACAACCACAACCAGAACCACAACCACAGCCACAACCTG

AACCCGAAGAAGAAGCACCACAGAAGAGGGCAAAGAAGTGCACATCGGATGTATGGCAGCATTTCACCAA

GAAGGAAATTGAAGTGGAGGTCGATGGAAAGAAATACGTTCAGGTATGGGGACATTGCAACTTTCCTAAT

TGCAAGGCTAAGTATAGGGCTGAGGGTCATCATGGAACAAGCGGATTTCGAAATCACTTGAGAACATCAC

ATAGTTTAGTTAAAGGTCAGTTGTGTCTAAAAAGTGAAAAGGATCATGGCAAAGACATAAATCTCATTGA

GCCTTATAAGTACGATGAAGTGGTTAGCCTAAAGAAGCTCTAGGATACCGTCGACCCGAGATCATATCAC

TGTGGACGTTGATGAAAGAATACGTTATTCTTTCATCAAATCGTGGTCGACCTCGACCACCGCGGTGGCG

GCCGCTCTAGAACTAGTGGATCCCCCGGGCTGCAGGAATTCGATATCAAGCTCGCGTCCTAGAGATCCGT

CAACATGGTGGAGCACGACACTCTCGTCTACTCCAAGAATATCAAAGATACAGTCTCAGAAGACCAAAGG

GCTATTGAGACTTTTCAACAAAGGGTAATATCGGGAAACCTCCTCGGATTCCATTGCCCAGCTATCTGTC

ACTTCATCAAAAGGACAGTAGAAAAGGAAGGTGGCACCTACAAATGCCATCATTGCGATAAAGGAAAGGC

TATCGTTCAAGATGCCTCTGCCGACAGTGGTCCCAAAGATGGACCCCCACCCACGAGGAGCATCGTGGAA

AAAGAAGACGTTCCAACCACGTCTTCAAAGCAAGTGGATTGATGTGATATCTCCACTGACGTAAGGGATG

ACGCACAATCCCACTATCCTTCGCAAGACCCTTCCTCTATATAAGGAAGTTCATTTCATTTGGAGAGGAC

GACCCCGATATGAAAAAGCCTGAACTCACCGCGACGTCTGTCGAGAAGTTTCTGATCGAAAAGTTCGACA

GCGTCTCCGACCTGATGCAGCTCTCGGAGGGCGAAGAATCTCGTGCTTTCAGCTTCGATGTAGGAGGGCG

TGGATATGTCCTGCGGGTAAATAGCTGCGCCGATGGTTTCTACAAAGATCGTTATGTTTATCGGCACTTT

GCATCGGCCGCGCTCCCGATTCCGGAAGTGCTTGACATTGGGGAATTCAGCGAGAGCCTGACCTATTGCA

TCTCCCGCCGTGCACAGGGTGTCACGTTGCAAGACCTGCCTGAAACCGAACTGCCCGCTGTTCTGCAGCC

GGTCGCGGAGGCCATGGATGCGATCGCTGCGGCCGATCTTAGCCAGACGAGCGGGTTCGGCCCATTCGGA

CCGCAAGGAATCGGTCAATACACTACATGGCGTGATTTCATATGCGCGATTGCTGATCCCCATGTGTATC

ACTGGCAAACTGTGATGGACGACACCGTCAGTGCGTCCGTCGCGCAGGCTCTCGATGAGCTGATGCTTTG

GGCCGAGGACTGCCCCGAAGTCCGGCACCTCGTGCACGCGGATTTCGGCTCCAACAATGTCCTGACGGAC

AATGGCCGCATAACAGCGGTCATTGACTGGAGCGAGGCGATGTTCGGGGATTCCCAATACGAGGTCGCCA

ACATCTTCTTCTGGAGGCCGTGGTTGGCTTGTATGGAGCAGCAGACGCGCTACTTCGAGCGGAGGCATCC

GGAGCTTGCAGGATCGCCGCGGCTCCGGGCGTATATGCTCCGCATTGGTCTTGACCAACTCTATCAGAGC

TTGGTTGACGGCAATTTCGATGATGCAGCTTGGGCGCAGGGTCGATGCGACGCAATCGTCCGATCCGGAG

CCGGGACTGTCGGGCGTACACAAATCGCCCGCAGAAGCGCGGCCGTCTGGACCGATGGCTGTGTAGAAGT

ACTCGCCGATAGTGGAAACCGACGCCCCAGCACTCGTCCGAGGGCAAAGGAATAGAGTAGATGCCGACCG

GGATCGATCCAACACTTACGTTTGCAACGTCCAAGAGCAAATAGACCACGAACGCCGGAAGGTTGCCGCA

GCGTGTGGATTGCGTCTCAATTCTCTCTTGCAGGAATGCAATGATGAATATGATACTGACTATGAAACTT

TGAGGGAATACTGCCTAGCACCGTCACCTCATAACGTGCATCATGCATGCCCTGACAACATGGAACATCG

CTATTTTTCTGAAGAATTATGCTCGTTGGAGGATGTCGCGGCAATTGCAGCTATTGCCAACATCGAACTA

CCCCTCACGCATGCATTCATCAATATTATTCATGCGGGGAAAGGCAAGATTAATCCAACTGGCAAATCAT

CCAGCGTGATTGGTAACTTCAGTTCCAGCGACTTGATTCGTTTTGGTGCTACCCACGTTTTCAATAAGGA

CGAGATGGTGGAGTAAAGAAGGAGTGCGTCGAAGCAGATCGTTCAAACATTTGGCAATAAAGTTTCTTAA

GATTGAATCCTGTTGCCGGTCTTGCGATGATTATCATATAATTTCTGTTGAATTACGTTAAGCATGTAAT

AATTAACATGTAATGCATGACGTTATTTATGAGATGGGTTTTTATGATTAGAGTCCCGCAATTATACATT

TAATACGCGATAGAAAACAAAATATAGCGCGCAAACTAGGATAAATTATCGCGCGCGGTGTCATCTATGT

TACTAGATCGATCAAACTTCGGTACTGTGTAATGACGATGAGCAATCGAGAGGCTGACTAACAAAAGGTA

TGCCCAAAAACAACCTCTCCAAACTGTTTCGAATTGGAAGTTTCTGCTCATGCCGACAGGCATAACTTAG

ATATTCGCGGGCTATTCCCACTAATTCGTCCTGCTGGTTTGCGCCAAGATAAATCAGTGCATCTCCTTAC

AAGTTCCTCTGTCTTGTGAAATGAACTGCTGACTGCCCCCCAAGAAAGCCTCCTCATCTCCCAGTTGGCG

GCGGCTGATACACCATCGAAAACCCACGTCCGAACACTTGATACATGTGCCTGAGAAATAGGCCTACGTC

CAAGAGCAAGTCCTTTCTGTGCTCGTCGGAAATTCCTCTCCTGTCAGACGGTCGTGCGCATGTCTTGCGT

TGATGAAGCTCGATACCGTCGACCACGATTTGATGAAAGAATAACGTATTCTTTCATCAACGTCCACAGT

GATATGATCTCGGGTCGACCTCGCCACCGCGGTGGCGGCCGCTCTAGAACTAGTGGATCCGCTAGGGATA

ACAGGGTAATATAGATCCCCGGGCTGCAGGAATTCGATATCAAGCTCTAGAACTAGTGGATCTGCCTCGC

TGGCCTGCCGCAGTTCTTCAACCTCCCGGCGCAGCTTTTCGTTCTCAATTTCAGCATCCCTTTCGGCATA

CCATTTTATGACGGCGGCAGAGTCATAAAGCACCTCATTACCCTTGCCACCGCCTCGCAGAACGGGCATT

CCCTGTTCCTGCCAGTTCTGAATGGTACGGATACTCGCACCGAAAATGTCAGCCAGCTGCTTTTTGTTGA

CTTCCATTGTTCATTCCACGGACAAAAACAGAGAAAGGAAACGACAGAGGCCAAAAAGCTCGCTTTCAGC

ACCTGTCGTTTCCTTTCTTTTCAGAGGGTATTTTAAATAAAAACATTAAGTTATGACGAAGAAGAACGGA

AACGCCTTAAACCGGAAAATTTTCATAAATAGCGAAAACCCGCGAGGTCGCCGCCCCGTAACAAGGCGGA

TCGCCGGAAAGGACCCGCAAATGATAATAATTATCAATTGCATACTATCGACGGCACTGCTGCCAGATAA

CACCACCGGGGAAACATTCCATCATGATGGCCGTGCGGACATAGGAAGCCAGTTCATCCATCGCTTTCTT

GTCTGCTGCCATTTGCTTTGTGACATCCAGCGCCGCACATTCAGCAGCGTTTTTCAGCGCGTTTTCGATC

AACGTTTCAATGTTGGTATCAACACCAGGTTTAACTTTGAACTTATCGGCACTGACGGTTACCTTGTTCT

GCGCTGGCTCATCACGCTGGATACCAAGGCTGATGTTGTAGATATTGGTCACCGGCTGAGGTGTTTCGAT

TGCCGCTGCGTGGATAGCACCATTTGCGATAGCGGCGTCCTTGATGAATGACACTCCATTGCGAATAAGT

TCGAAGGAGACGGTGTCACGAATGCGCTGGTCCAGCTCGTCGATTGCCTTTTGTGCAGCAGAGGTATCAA

TCTCAACGCCAAGCGTCATCGAAGCGCAATATTGCTGCTCACCAAAACGCGTATTGACCAGGTGTTCAAC

GGCAAATTTCTGCCCTTCTGATGTCAGAAAGGTAAAGTGATTTTCTTTCTGGTATTCAGTTGCTGTGTGT

CTGGTTTCAGCAAAACCAAGCTCGCGCAATTCGGCTGTGCCAGATTTAGAAGGCAGATCACCAGACAGCA

ACGCGCCACGGAAAAACAGCGCATACAGAACATCCGTCGCCGCGCCGGACAACGTGATAATTTTATGACC

CATGATTTATTTCCTTTTAGACGTGAGCCTGTCGCACAGCAAAGCCGCCGAAAGTTAACGGTTTGCCCAG

GCTCACAACTGAAAGACTTTCTACGGTGTGCGCGTGCGATGCGCGTAGAAGACTGATTTATCAACCTGTC

TTTATATCAGGATTCATTACCTGACTATTTGTGGGTAAAGTTCGTAGTGCGCTGATCGTGCAAAATGATT

TTAGTTGGGAACAGTTCGCAACTCTGTCCCATAAAAATCAGCATATTCCCATCTATCCCATATCCAGCGC

ATTGACCATCGGGATACTGAAGGGAGATTCCATCATCTCTTAGAAAGATCACCATCTCTTTTGTTTCAAT

TTGCATATAGCTACCTGGAGGATTTATGAATACAAGGATTTTCATGGACTATTACCATGAGATTGATTTT

CCATCTTTATTCGCGAGAGCAGTGGAAAGCGATGACGATGTGGGTACTACATTGCGCATTCACCTACTTT

GTGAGCGCATGGTCGAAGCATGGATATGCGCATGCTGTGACTGCCAAGATCCCCCCTAGAAAAGGTGAAT

GCATATATGTTATAATGAAGTTCCAATTTATAGTTATTCAACAATTATTTTACTTATATTGATGCATATT

TGTGTCATTCAAGGTGCTACATATTTTCCAACAATGATTGGTGATCTCGAGGTGCTAGACTCTGTTATTG

CTGCTGCAACAAATCATGAGAATCATATGGATGAGGTATTTAAAGATTATTATTTACTTCGTGCATGGGC

TATTAATTTGCTATTATTCACTACTGTTTTGATGCATGGGCTGTTTGCTGTCGCCTTGTTTTGATGCATG

CGCCTTGCTGCCCAGCCGTGTTATACTCCCTGCATGGCTGGCATTAACAGATTTTTGATCTCACTGCATG

CGCCTTGTCGCCTTGTTTTGATTGGCTGCTAGCTGCTAGCTGTTAGGCTCCCAGCTGTTAGGCGCTAGCT

GCTAGCTGCCTAGCTCCCAGCCGTGTTAGTTCACAGATTCATGTTCTCCTATATGTATTTATTTATACTC

CCTGCATATCAATTCATGTTCTTCTATATGTATTTATTTATCCAAAACTGACTTATTTTTGTGTATTAAC

AGGATGAAGACGCAATAGAATTTTCTAAGAATAATGAAGATGTAGCAAGTGGCTCCTCTCCATGAGCAAT

GTGTCTTATGTTTGTTGACAGATGAGCCTTGGTTGTAATAGTTTATGCATGCTAAGTGATCCAGATGTGA

GCAAGTGATTATGAATATGTGTTTTAAACTTTATATTGTGTCATGTGTGCTAGTAGACTTATATGGCTTC

TTATGTTAGCCAAGAGCCCAAGACTTATCACTTATGTGCTACATTAAACTATGTGTGCTCCAGATTTATA

TGGATTTTATCTATGTTTAATTAAGACTTGTGTTTACAATTTTTTATATTTGTTTTTAAGTTTTGAATAT

ATGTTTTCATGTGTGATTTTACCGAACAAAAATACCGGTTCCCGTCCGATTTCGACTTTAACCCGACCGG

ATCGTATCGGTTTTCGATTACCGTATTTATCCCGTTCGTTTTCGTTACCGGTATATCCCGTTTTCGTTTC

CGTCCCGCAAGTTAAATATGAAAATGAAAACGGTAGAGGTATTTTACCGACCGTTACCGACCGTTTTCAT

CCCTA

>gi|13540346|gb|AF354045.1|AF354045 Binary vector pCAMBIA-1305.1, complete sequence

CATGGTAGATCTGAGGGTAAATTTCTAGTTTTTCTCCTTCATTTTCTTGGTTAGGACCCTTTTCTCTTTT

TATTTTTTTGAGCTTTGATCTTTCTTTAAACTGATCTATTTTTTAATTGATTGGTTATGGTGTAAATATT

ACATAGCTTTAACTGATAATCTGATTACTTTATTTCGTGTGTCTATGATGATGATGATAGTTACAGAACC

GACGAACTAGTCTGTACCCGATCAACACCGAGACCCGTGGCGTCTTCGACCTCAATGGCGTCTGGAACTT

CAAGCTGGACTACGGGAAAGGACTGGAAGAGAAGTGGTACGAAAGCAAGCTGACCGACACTATTAGTATG

GCCGTCCCAAGCAGTTACAATGACATTGGCGTGACCAAGGAAATCCGCAACCATATCGGATATGTCTGGT

ACGAACGTGAGTTCACGGTGCCGGCCTATCTGAAGGATCAGCGTATCGTGCTCCGCTTCGGCTCTGCAAC

TCACAAAGCAATTGTCTATGTCAATGGTGAGCTGGTCGTGGAGCACAAGGGCGGATTCCTGCCATTCGAA

GCGGAAATCAACAACTCGCTGCGTGATGGCATGAATCGCGTCACCGTCGCCGTGGACAACATCCTCGACG

ATAGCACCCTCCCGGTGGGGCTGTACAGCGAGCGCCACGAAGAGGGCCTCGGAAAAGTCATTCGTAACAA

GCCGAACTTCGACTTCTTCAACTATGCAGGCCTGCACCGTCCGGTGAAAATCTACACGACCCCGTTTACG

TACGTCGAGGACATCTCGGTTGTGACCGACTTCAATGGCCCAACCGGGACTGTGACCTATACGGTGGACT

TTCAAGGCAAAGCCGAGACCGTGAAAGTGTCGGTCGTGGATGAGGAAGGCAAAGTGGTCGCAAGCACCGA

GGGCCTGAGCGGTAACGTGGAGATTCCGAATGTCATCCTCTGGGAACCACTGAACACGTATCTCTACCAG

ATCAAAGTGGAACTGGTGAACGACGGACTGACCATCGATGTCTATGAAGAGCCGTTCGGCGTGCGGACCG

TGGAAGTCAACGACGGCAAGTTCCTCATCAACAACAAACCGTTCTACTTCAAGGGCTTTGGCAAACATGA

GGACACTCCTATCAACGGCCGTGGCTTTAACGAAGCGAGCAATGTGATGGATTTCAATATCCTCAAATGG

ATCGGCGCCAACAGCTTCCGGACCGCACACTATCCGTACTCTGAAGAGTTGATGCGTCTTGCGGATCGCG

AGGGTCTGGTCGTGATCGACGAGACTCCGGCAGTTGGCGTGCACCTCAACTTCATGGCCACCACGGGACT

CGGCGAAGGCAGCGAGCGCGTCAGTACCTGGGAGAAGATTCGGACGTTTGAGCACCATCAAGACGTTCTC

CGTGAACTGGTGTCTCGTGACAAGAACCATCCAAGCGTCGTGATGTGGAGCATCGCCAACGAGGCGGCGA

CTGAGGAAGAGGGCGCGTACGAGTACTTCAAGCCGTTGGTGGAGCTGACCAAGGAACTCGACCCACAGAA

GCGTCCGGTCACGATCGTGCTGTTTGTGATGGCTACCCCGGAGACGGACAAAGTCGCCGAACTGATTGAC

GTCATCGCGCTCAATCGCTATAACGGATGGTACTTCGATGGCGGTGATCTCGAAGCGGCCAAAGTCCATC

TCCGCCAGGAATTTCACGCGTGGAACAAGCGTTGCCCAGGAAAGCCGATCATGATCACTGAGTACGGCGC

AGACACCGTTGCGGGCTTTCACGACATTGATCCAGTGATGTTCACCGAGGAATATCAAGTCGAGTACTAC

CAGGCGAACCACGTCGTGTTCGATGAGTTTGAGAACTTCGTGGGTGAGCAAGCGTGGAACTTCGCGGACT

TCGCGACCTCTCAGGGCGTGATGCGCGTCCAAGGAAACAAGAAGGGCGTGTTCACTCGTGACCGCAAGCC

GAAGCTCGCCGCGCACGTCTTTCGCGAGCGCTGGACCAACATTCCAGATTTCGGCTACAAGAACGCTAGC

CATCACCATCACCATCACGTGTGAATTGGTGACCAGCTCGAATTTCCCCGATCGTTCAAACATTTGGCAA

TAAAGTTTCTTAAGATTGAATCCTGTTGCCGGTCTTGCGATGATTATCATATAATTTCTGTTGAATTACG

TTAAGCATGTAATAATTAACATGTAATGCATGACGTTATTTATGAGATGGGTTTTTATGATTAGAGTCCC

GCAATTATACATTTAATACGCGATAGAAAACAAAATATAGCGCGCAAACTAGGATAAATTATCGCGCGCG

GTGTCATCTATGTTACTAGATCGGGAATTAAACTATCAGTGTTTGACAGGATATATTGGCGGGTAAACCT

AAGAGAAAAGAGCGTTTATTAGAATAACGGATATTTAAAAGGGCGTGAAAAGGTTTATCCGTTCGTCCAT

TTGTATGTGCATGCCAACCACAGGGTTCCCCTCGGGATCAAAGTACTTTGATCCAACCCCTCCGCTGCTA

TAGTGCAGTCGGCTTCTGACGTTCAGTGCAGCCGTCTTCTGAAAACGACATGTCGCACAAGTCCTAAGTT

ACGCGACAGGCTGCCGCCCTGCCCTTTTCCTGGCGTTTTCTTGTCGCGTGTTTTAGTCGCATAAAGTAGA

ATACTTGCGACTAGAACCGGAGACATTACGCCATGAACAAGAGCGCCGCCGCTGGCCTGCTGGGCTATGC

CCGCGTCAGCACCGACGACCAGGACTTGACCAACCAACGGGCCGAACTGCACGCGGCCGGCTGCACCAAG

CTGTTTTCCGAGAAGATCACCGGCACCAGGCGCGACCGCCCGGAGCTGGCCAGGATGCTTGACCACCTAC

GCCCTGGCGACGTTGTGACAGTGACCAGGCTAGACCGCCTGGCCCGCAGCACCCGCGACCTACTGGACAT

TGCCGAGCGCATCCAGGAGGCCGGCGCGGGCCTGCGTAGCCTGGCAGAGCCGTGGGCCGACACCACCACG

CCGGCCGGCCGCATGGTGTTGACCGTGTTCGCCGGCATTGCCGAGTTCGAGCGTTCCCTAATCATCGACC

GCACCCGGAGCGGGCGCGAGGCCGCCAAGGCCCGAGGCGTGAAGTTTGGCCCCCGCCCTACCCTCACCCC

GGCACAGATCGCGCACGCCCGCGAGCTGATCGACCAGGAAGGCCGCACCGTGAAAGAGGCGGCTGCACTG

CTTGGCGTGCATCGCTCGACCCTGTACCGCGCACTTGAGCGCAGCGAGGAAGTGACGCCCACCGAGGCCA

GGCGGCGCGGTGCCTTCCGTGAGGACGCATTGACCGAGGCCGACGCCCTGGCGGCCGCCGAGAATGAACG

CCAAGAGGAACAAGCATGAAACCGCACCAGGACGGCCAGGACGAACCGTTTTTCATTACCGAAGAGATCG

AGGCGGAGATGATCGCGGCCGGGTACGTGTTCGAGCCGCCCGCGCACGTCTCAACCGTGCGGCTGCATGA

AATCCTGGCCGGTTTGTCTGATGCCAAGCTGGCGGCCTGGCCGGCCAGCTTGGCCGCTGAAGAAACCGAG

CGCCGCCGTCTAAAAAGGTGATGTGTATTTGAGTAAAACAGCTTGCGTCATGCGGTCGCTGCGTATATGA

TGCGATGAGTAAATAAACAAATACGCAAGGGGAACGCATGAAGGTTATCGCTGTACTTAACCAGAAAGGC

GGGTCAGGCAAGACGACCATCGCAACCCATCTAGCCCGCGCCCTGCAACTCGCCGGGGCCGATGTTCTGT

TAGTCGATTCCGATCCCCAGGGCAGTGCCCGCGATTGGGCGGCCGTGCGGGAAGATCAACCGCTAACCGT

TGTCGGCATCGACCGCCCGACGATTGACCGCGACGTGAAGGCCATCGGCCGGCGCGACTTCGTAGTGATC

GACGGAGCGCCCCAGGCGGCGGACTTGGCTGTGTCCGCGATCAAGGCAGCCGACTTCGTGCTGATTCCGG

TGCAGCCAAGCCCTTACGACATATGGGCCACCGCCGACCTGGTGGAGCTGGTTAAGCAGCGCATTGAGGT

CACGGATGGAAGGCTACAAGCGGCCTTTGTCGTGTCGCGGGCGATCAAAGGCACGCGCATCGGCGGTGAG

GTTGCCGAGGCGCTGGCCGGGTACGAGCTGCCCATTCTTGAGTCCCGTATCACGCAGCGCGTGAGCTACC

CAGGCACTGCCGCCGCCGGCACAACCGTTCTTGAATCAGAACCCGAGGGCGACGCTGCCCGCGAGGTCCA

GGCGCTGGCCGCTGAAATTAAATCAAAACTCATTTGAGTTAATGAGGTAAAGAGAAAATGAGCAAAAGCA

CAAACACGCTAAGTGCCGGCCGTCCGAGCGCACGCAGCAGCAAGGCTGCAACGTTGGCCAGCCTGGCAGA

CACGCCAGCCATGAAGCGGGTCAACTTTCAGTTGCCGGCGGAGGATCACACCAAGCTGAAGATGTACGCG

GTACGCCAAGGCAAGACCATTACCGAGCTGCTATCTGAATACATCGCGCAGCTACCAGAGTAAATGAGCA

AATGAATAAATGAGTAGATGAATTTTAGCGGCTAAAGGAGGCGGCATGGAAAATCAAGAACAACCAGGCA

CCGACGCCGTGGAATGCCCCATGTGTGGAGGAACGGGCGGTTGGCCAGGCGTAAGCGGCTGGGTTGTCTG

CCGGCCCTGCAATGGCACTGGAACCCCCAAGCCCGAGGAATCGGCGTGACGGTCGCAAACCATCCGGCCC

GGTACAAATCGGCGCGGCGCTGGGTGATGACCTGGTGGAGAAGTTGAAGGCCGCGCAGGCCGCCCAGCGG

CAACGCATCGAGGCAGAAGCACGCCCCGGTGAATCGTGGCAAGCGGCCGCTGATCGAATCCGCAAAGAAT

CCCGGCAACCGCCGGCAGCCGGTGCGCCGTCGATTAGGAAGCCGCCCAAGGGCGACGAGCAACCAGATTT

TTTCGTTCCGATGCTCTATGACGTGGGCACCCGCGATAGTCGCAGCATCATGGACGTGGCCGTTTTCCGT

CTGTCGAAGCGTGACCGACGAGCTGGCGAGGTGATCCGCTACGAGCTTCCAGACGGGCACGTAGAGGTTT

CCGCAGGGCCGGCCGGCATGGCCAGTGTGTGGGATTACGACCTGGTACTGATGGCGGTTTCCCATCTAAC

CGAATCCATGAACCGATACCGGGAAGGGAAGGGAGACAAGCCCGGCCGCGTGTTCCGTCCACACGTTGCG

GACGTACTCAAGTTCTGCCGGCGAGCCGATGGCGGAAAGCAGAAAGACGACCTGGTAGAAACCTGCATTC

GGTTAAACACCACGCACGTTGCCATGCAGCGTACGAAGAAGGCCAAGAACGGCCGCCTGGTGACGGTATC

CGAGGGTGAAGCCTTGATTAGCCGCTACAAGATCGTAAAGAGCGAAACCGGGCGGCCGGAGTACATCGAG

ATCGAGCTAGCTGATTGGATGTACCGCGAGATCACAGAAGGCAAGAACCCGGACGTGCTGACGGTTCACC

CCGATTACTTTTTGATCGATCCCGGCATCGGCCGTTTTCTCTACCGCCTGGCACGCCGCGCCGCAGGCAA

GGCAGAAGCCAGATGGTTGTTCAAGACGATCTACGAACGCAGTGGCAGCGCCGGAGAGTTCAAGAAGTTC

TGTTTCACCGTGCGCAAGCTGATCGGGTCAAATGACCTGCCGGAGTACGATTTGAAGGAGGAGGCGGGGC

AGGCTGGCCCGATCCTAGTCATGCGCTACCGCAACCTGATCGAGGGCGAAGCATCCGCCGGTTCCTAATG

TACGGAGCAGATGCTAGGGCAAATTGCCCTAGCAGGGGAAAAAGGTCGAAAAGGTCTCTTTCCTGTGGAT

AGCACGTACATTGGGAACCCAAAGCCGTACATTGGGAACCGGAACCCGTACATTGGGAACCCAAAGCCGT

ACATTGGGAACCGGTCACACATGTAAGTGACTGATATAAAAGAGAAAAAAGGCGATTTTTCCGCCTAAAA

CTCTTTAAAACTTATTAAAACTCTTAAAACCCGCCTGGCCTGTGCATAACTGTCTGGCCAGCGCACAGCC

GAAGAGCTGCAAAAAGCGCCTACCCTTCGGTCGCTGCGCTCCCTACGCCCCGCCGCTTCGCGTCGGCCTA

TCGCGGCCGCTGGCCGCTCAAAAATGGCTGGCCTACGGCCAGGCAATCTACCAGGGCGCGGACAAGCCGC

GCCGTCGCCACTCGACCGCCGGCGCCCACATCAAGGCACCCTGCCTCGCGCGTTTCGGTGATGACGGTGA

AAACCTCTGACACATGCAGCTCCCGGAGACGGTCACAGCTTGTCTGTAAGCGGATGCCGGGAGCAGACAA

GCCCGTCAGGGCGCGTCAGCGGGTGTTGGCGGGTGTCGGGGCGCAGCCATGACCCAGTCACGTAGCGATA

GCGGAGTGTATACTGGCTTAACTATGCGGCATCAGAGCAGATTGTACTGAGAGTGCACCATATGCGGTGT

GAAATACCGCACAGATGCGTAAGGAGAAAATACCGCATCAGGCGCTCTTCCGCTTCCTCGCTCACTGACT

CGCTGCGCTCGGTCGTTCGGCTGCGGCGAGCGGTATCAGCTCACTCAAAGGCGGTAATACGGTTATCCAC

AGAATCAGGGGATAACGCAGGAAAGAACATGTGAGCAAAAGGCCAGCAAAAGGCCAGGAACCGTAAAAAG

GCCGCGTTGCTGGCGTTTTTCCATAGGCTCCGCCCCCCTGACGAGCATCACAAAAATCGACGCTCAAGTC

AGAGGTGGCGAAACCCGACAGGACTATAAAGATACCAGGCGTTTCCCCCTGGAAGCTCCCTCGTGCGCTC

TCCTGTTCCGACCCTGCCGCTTACCGGATACCTGTCCGCCTTTCTCCCTTCGGGAAGCGTGGCGCTTTCT

CATAGCTCACGCTGTAGGTATCTCAGTTCGGTGTAGGTCGTTCGCTCCAAGCTGGGCTGTGTGCACGAAC

CCCCCGTTCAGCCCGACCGCTGCGCCTTATCCGGTAACTATCGTCTTGAGTCCAACCCGGTAAGACACGA

CTTATCGCCACTGGCAGCAGCCACTGGTAACAGGATTAGCAGAGCGAGGTATGTAGGCGGTGCTACAGAG

TTCTTGAAGTGGTGGCCTAACTACGGCTACACTAGAAGGACAGTATTTGGTATCTGCGCTCTGCTGAAGC

CAGTTACCTTCGGAAAAAGAGTTGGTAGCTCTTGATCCGGCAAACAAACCACCGCTGGTAGCGGTGGTTT

TTTTGTTTGCAAGCAGCAGATTACGCGCAGAAAAAAAGGATCTCAAGAAGATCCTTTGATCTTTTCTACG

GGGTCTGACGCTCAGTGGAACGAAAACTCACGTTAAGGGATTTTGGTCATGCATTCTAGGTACTAAAACA

ATTCATCCAGTAAAATATAATATTTTATTTTCTCCCAATCAGGCTTGATCCCCAGTAAGTCAAAAAATAG

CTCGACATACTGTTCTTCCCCGATATCCTCCCTGATCGACCGGACGCAGAAGGCAATGTCATACCACTTG

TCCGCCCTGCCGCTTCTCCCAAGATCAATAAAGCCACTTACTTTGCCATCTTTCACAAAGATGTTGCTGT

CTCCCAGGTCGCCGTGGGAAAAGACAAGTTCCTCTTCGGGCTTTTCCGTCTTTAAAAAATCATACAGCTC

GCGCGGATCTTTAAATGGAGTGTCTTCTTCCCAGTTTTCGCAATCCACATCGGCCAGATCGTTATTCAGT

AAGTAATCCAATTCGGCTAAGCGGCTGTCTAAGCTATTCGTATAGGGACAATCCGATATGTCGATGGAGT

GAAAGAGCCTGATGCACTCCGCATACAGCTCGATAATCTTTTCAGGGCTTTGTTCATCTTCATACTCTTC

CGAGCAAAGGACGCCATCGGCCTCACTCATGAGCAGATTGCTCCAGCCATCATGCCGTTCAAAGTGCAGG

ACCTTTGGAACAGGCAGCTTTCCTTCCAGCCATAGCATCATGTCCTTTTCCCGTTCCACATCATAGGTGG

TCCCTTTATACCGGCTGTCCGTCATTTTTAAATATAGGTTTTCATTTTCTCCCACCAGCTTATATACCTT

AGCAGGAGACATTCCTTCCGTATCTTTTACGCAGCGGTATTTTTCGATCAGTTTTTTCAATTCCGGTGAT

ATTCTCATTTTAGCCATTTATTATTTCCTTCCTCTTTTCTACAGTATTTAAAGATACCCCAAGAAGCTAA

TTATAACAAGACGAACTCCAATTCACTGTTCCTTGCATTCTAAAACCTTAAATACCAGAAAACAGCTTTT

TCAAAGTTGTTTTCAAAGTTGGCGTATAACATAGTATCGACGGAGCCGATTTTGAAACCGCGGTGATCAC

AGGCAGCAACGCTCTGTCATCGTTACAATCAACATGCTACCCTCCGCGAGATCATCCGTGTTTCAAACCC

GGCAGCTTAGTTGCCGTTCTTCCGAATAGCATCGGTAACATGAGCAAAGTCTGCCGCCTTACAACGGCTC

TCCCGCTGACGCCGTCCCGGACTGATGGGCTGCCTGTATCGAGTGGTGATTTTGTGCCGAGCTGCCGGTC

GGGGAGCTGTTGGCTGGCTGGTGGCAGGATATATTGTGGTGTAAACAAATTGACGCTTAGACAACTTAAT

AACACATTGCGGACGTTTTTAATGTACTGAATTAACGCCGAATTAATTCGGGGGATCTGGATTTTAGTAC

TGGATTTTGGTTTTAGGAATTAGAAATTTTATTGATAGAAGTATTTTACAAATACAAATACATACTAAGG

GTTTCTTATATGCTCAACACATGAGCGAAACCCTATAGGAACCCTAATTCCCTTATCTGGGAACTACTCA

CACATTATTATGGAGAAACTCGAGCTTGTCGATCGACAGATCCGGTCGGCATCTACTCTATTTCTTTGCC

CTCGGACGAGTGCTGGGGCGTCGGTTTCCACTATCGGCGAGTACTTCTACACAGCCATCGGTCCAGACGG

CCGCGCTTCTGCGGGCGATTTGTGTACGCCCGACAGTCCCGGCTCCGGATCGGACGATTGCGTCGCATCG

ACCCTGCGCCCAAGCTGCATCATCGAAATTGCCGTCAACCAAGCTCTGATAGAGTTGGTCAAGACCAATG

CGGAGCATATACGCCCGGAGTCGTGGCGATCCTGCAAGCTCCGGATGCCTCCGCTCGAAGTAGCGCGTCT

GCTGCTCCATACAAGCCAACCACGGCCTCCAGAAGAAGATGTTGGCGACCTCGTATTGGGAATCCCCGAA

CATCGCCTCGCTCCAGTCAATGACCGCTGTTATGCGGCCATTGTCCGTCAGGACATTGTTGGAGCCGAAA

TCCGCGTGCACGAGGTGCCGGACTTCGGGGCAGTCCTCGGCCCAAAGCATCAGCTCATCGAGAGCCTGCG

CGACGGACGCACTGACGGTGTCGTCCATCACAGTTTGCCAGTGATACACATGGGGATCAGCAATCGCGCA

TATGAAATCACGCCATGTAGTGTATTGACCGATTCCTTGCGGTCCGAATGGGCCGAACCCGCTCGTCTGG

CTAAGATCGGCCGCAGCGATCGCATCCATAGCCTCCGCGACCGGTTGTAGAACAGCGGGCAGTTCGGTTT

CAGGCAGGTCTTGCAACGTGACACCCTGTGCACGGCGGGAGATGCAATAGGTCAGGCTCTCGCTAAACTC

CCCAATGTCAAGCACTTCCGGAATCGGGAGCGCGGCCGATGCAAAGTGCCGATAAACATAACGATCTTTG

TAGAAACCATCGGCGCAGCTATTTACCCGCAGGACATATCCACGCCCTCCTACATCGAAGCTGAAAGCAC

GAGATTCTTCGCCCTCCGAGAGCTGCATCAGGTCGGAGACGCTGTCGAACTTTTCGATCAGAAACTTCTC

GACAGACGTCGCGGTGAGTTCAGGCTTTTTCATATCTCATTGCCCCCCGGGATCTGCGAAAGCTCGAGAG

AGATAGATTTGTAGAGAGAGACTGGTGATTTCAGCGTGTCCTCTCCAAATGAAATGAACTTCCTTATATA

GAGGAAGGTCTTGCGAAGGATAGTGGGATTGTGCGTCATCCCTTACGTCAGTGGAGATATCACATCAATC

CACTTGCTTTGAAGACGTGGTTGGAACGTCTTCTTTTTCCACGATGCTCCTCGTGGGTGGGGGTCCATCT

TTGGGACCACTGTCGGCAGAGGCATCTTGAACGATAGCCTTTCCTTTATCGCAATGATGGCATTTGTAGG

TGCCACCTTCCTTTTCTACTGTCCTTTTGATGAAGTGACAGATAGCTGGGCAATGGAATCCGAGGAGGTT

TCCCGATATTACCCTTTGTTGAAAAGTCTCAATAGCCCTTTGGTCTTCTGAGACTGTATCTTTGATATTC

TTGGAGTAGACGAGAGTGTCGTGCTCCACCATGTTATCACATCAATCCACTTGCTTTGAAGACGTGGTTG

GAACGTCTTCTTTTTCCACGATGCTCCTCGTGGGTGGGGGTCCATCTTTGGGACCACTGTCGGCAGAGGC

ATCTTGAACGATAGCCTTTCCTTTATCGCAATGATGGCATTTGTAGGTGCCACCTTCCTTTTCTACTGTC

CTTTTGATGAAGTGACAGATAGCTGGGCAATGGAATCCGAGGAGGTTTCCCGATATTACCCTTTGTTGAA

AAGTCTCAATAGCCCTTTGGTCTTCTGAGACTGTATCTTTGATATTCTTGGAGTAGACGAGAGTGTCGTG

CTCCACCATGTTGGCAAGCTGCTCTAGCCAATACGCAAACCGCCTCTCCCCGCGCGTTGGCCGATTCATT

AATGCAGCTGGCACGACAGGTTTCCCGACTGGAAAGCGGGCAGTGAGCGCAACGCAATTAATGTGAGTTA

GCTCACTCATTAGGCACCCCAGGCTTTACACTTTATGCTTCCGGCTCGTATGTTGTGTGGAATTGTGAGC

GGATAACAATTTCACACAGGAAACAGCTATGACCATGATTACGAATTCGAGCTCGGTACCCGGGGATCCT

CTAGAGTCGACCTGCAGGCATGCAAGCTTGGCACTGGCCGTCGTTTTACAACGTCGTGACTGGGAAAACC

CTGGCGTTACCCAACTTAATCGCCTTGCAGCACATCCCCCTTTCGCCAGCTGGCGTAATAGCGAAGAGGC

CCGCACCGATCGCCCTTCCCAACAGTTGCGCAGCCTGAATGGCGAATGCTAGAGCAGCTTGAGCTTGGAT

CAGATTGTCGTTTCCCGCCTTCAGTTTAGCTTCATGGAGTCAAAGATTCAAATAGAGGACCTAACAGAAC

TCGCCGTAAAGACTGGCGAACAGTTCATACAGAGTCTCTTACGACTCAATGACAAGAAGAAAATCTTCGT

CAACATGGTGGAGCACGACACACTTGTCTACTCCAAAAATATCAAAGATACAGTCTCAGAAGACCAAAGG

GCAATTGAGACTTTTCAACAAAGGGTAATATCCGGAAACCTCCTCGGATTCCATTGCCCAGCTATCTGTC

ACTTTATTGTGAAGATAGTGGAAAAGGAAGGTGGCTCCTACAAATGCCATCATTGCGATAAAGGAAAGGC

CATCGTTGAAGATGCCTCTGCCGACAGTGGTCCCAAAGATGGACCCCCACCCACGAGGAGCATCGTGGAA

AAAGAAGACGTTCCAACCACGTCTTCAAAGCAAGTGGATTGATGTGATATCTCCACTGACGTAAGGGATG

ACGCACAATCCCACTATCCTTCGCAAGACCCTTCCTCTATATAAGGAAGTTCATTTCATTTGGAGAGAAC

ACGGGGGACTCTTGAC

>gi|13540351|gb|AF354046.1|AF354046 Binary vector pCAMBIA-1305.2, complete sequence

CATGGCTACTACTAAGCATTTGGCTCTTGCCATCCTTGTCCTCCTTAGCATTGGTATGACCACCAGTGCA

AGAACCCTCCTAGATCTGAGGGTAAATTTCTAGTTTTTCTCCTTCATTTTCTTGGTTAGGACCCTTTTCT

CTTTTTATTTTTTTGAGCTTTGATCTTTCTTTAAACTGATCTATTTTTTAATTGATTGGTTATGGTGTAA

ATATTACATAGCTTTAACTGATAATCTGATTACTTTATTTCGTGTGTCTATGATGATGATGATAGTTACA

GAACCGACGAACTAGTCTGTACCCGATCAACACCGAGACCCGTGGCGTCTTCGACCTCAATGGCGTCTGG

AACTTCAAGCTGGACTACGGGAAAGGACTGGAAGAGAAGTGGTACGAAAGCAAGCTGACCGACACTATTA

GTATGGCCGTCCCAAGCAGTTACAATGACATTGGCGTGACCAAGGAAATCCGCAACCATATCGGATATGT

CTGGTACGAACGTGAGTTCACGGTGCCGGCCTATCTGAAGGATCAGCGTATCGTGCTCCGCTTCGGCTCT

GCAACTCACAAAGCAATTGTCTATGTCAATGGTGAGCTGGTCGTGGAGCACAAGGGCGGATTCCTGCCAT

TCGAAGCGGAAATCAACAACTCGCTGCGTGATGGCATGAATCGCGTCACCGTCGCCGTGGACAACATCCT

CGACGATAGCACCCTCCCGGTGGGGCTGTACAGCGAGCGCCACGAAGAGGGCCTCGGAAAAGTCATTCGT

AACAAGCCGAACTTCGACTTCTTCAACTATGCAGGCCTGCACCGTCCGGTGAAAATCTACACGACCCCGT

TTACGTACGTCGAGGACATCTCGGTTGTGACCGACTTCAATGGCCCAACCGGGACTGTGACCTATACGGT

GGACTTTCAAGGCAAAGCCGAGACCGTGAAAGTGTCGGTCGTGGATGAGGAAGGCAAAGTGGTCGCAAGC

ACCGAGGGCCTGAGCGGTAACGTGGAGATTCCGAATGTCATCCTCTGGGAACCACTGAACACGTATCTCT

ACCAGATCAAAGTGGAACTGGTGAACGACGGACTGACCATCGATGTCTATGAAGAGCCGTTCGGCGTGCG

GACCGTGGAAGTCAACGACGGCAAGTTCCTCATCAACAACAAACCGTTCTACTTCAAGGGCTTTGGCAAA

CATGAGGACACTCCTATCAACGGCCGTGGCTTTAACGAAGCGAGCAATGTGATGGATTTCAATATCCTCA

AATGGATCGGCGCCAACAGCTTCCGGACCGCACACTATCCGTACTCTGAAGAGTTGATGCGTCTTGCGGA

TCGCGAGGGTCTGGTCGTGATCGACGAGACTCCGGCAGTTGGCGTGCACCTCAACTTCATGGCCACCACG

GGACTCGGCGAAGGCAGCGAGCGCGTCAGTACCTGGGAGAAGATTCGGACGTTTGAGCACCATCAAGACG

TTCTCCGTGAACTGGTGTCTCGTGACAAGAACCATCCAAGCGTCGTGATGTGGAGCATCGCCAACGAGGC

GGCGACTGAGGAAGAGGGCGCGTACGAGTACTTCAAGCCGTTGGTGGAGCTGACCAAGGAACTCGACCCA

CAGAAGCGTCCGGTCACGATCGTGCTGTTTGTGATGGCTACCCCGGAGACGGACAAAGTCGCCGAACTGA

TTGACGTCATCGCGCTCAATCGCTATAACGGATGGTACTTCGATGGCGGTGATCTCGAAGCGGCCAAAGT

CCATCTCCGCCAGGAATTTCACGCGTGGAACAAGCGTTGCCCAGGAAAGCCGATCATGATCACTGAGTAC

GGCGCAGACACCGTTGCGGGCTTTCACGACATTGATCCAGTGATGTTCACCGAGGAATATCAAGTCGAGT

ACTACCAGGCGAACCACGTCGTGTTCGATGAGTTTGAGAACTTCGTGGGTGAGCAAGCGTGGAACTTCGC

GGACTTCGCGACCTCTCAGGGCGTGATGCGCGTCCAAGGAAACAAGAAGGGCGTGTTCACTCGTGACCGC

AAGCCGAAGCTCGCCGCGCACGTCTTTCGCGAGCGCTGGACCAACATTCCAGATTTCGGCTACAAGAACG

CTAGCCATCACCATCACCATCACGTGTGAATTGGTGACCAGCTCGAATTTCCCCGATCGTTCAAACATTT

GGCAATAAAGTTTCTTAAGATTGAATCCTGTTGCCGGTCTTGCGATGATTATCATATAATTTCTGTTGAA

TTACGTTAAGCATGTAATAATTAACATGTAATGCATGACGTTATTTATGAGATGGGTTTTTATGATTAGA

GTCCCGCAATTATACATTTAATACGCGATAGAAAACAAAATATAGCGCGCAAACTAGGATAAATTATCGC

GCGCGGTGTCATCTATGTTACTAGATCGGGAATTAAACTATCAGTGTTTGACAGGATATATTGGCGGGTA

AACCTAAGAGAAAAGAGCGTTTATTAGAATAACGGATATTTAAAAGGGCGTGAAAAGGTTTATCCGTTCG

TCCATTTGTATGTGCATGCCAACCACAGGGTTCCCCTCGGGATCAAAGTACTTTGATCCAACCCCTCCGC

TGCTATAGTGCAGTCGGCTTCTGACGTTCAGTGCAGCCGTCTTCTGAAAACGACATGTCGCACAAGTCCT

AAGTTACGCGACAGGCTGCCGCCCTGCCCTTTTCCTGGCGTTTTCTTGTCGCGTGTTTTAGTCGCATAAA

GTAGAATACTTGCGACTAGAACCGGAGACATTACGCCATGAACAAGAGCGCCGCCGCTGGCCTGCTGGGC

TATGCCCGCGTCAGCACCGACGACCAGGACTTGACCAACCAACGGGCCGAACTGCACGCGGCCGGCTGCA

CCAAGCTGTTTTCCGAGAAGATCACCGGCACCAGGCGCGACCGCCCGGAGCTGGCCAGGATGCTTGACCA

CCTACGCCCTGGCGACGTTGTGACAGTGACCAGGCTAGACCGCCTGGCCCGCAGCACCCGCGACCTACTG

GACATTGCCGAGCGCATCCAGGAGGCCGGCGCGGGCCTGCGTAGCCTGGCAGAGCCGTGGGCCGACACCA

CCACGCCGGCCGGCCGCATGGTGTTGACCGTGTTCGCCGGCATTGCCGAGTTCGAGCGTTCCCTAATCAT

CGACCGCACCCGGAGCGGGCGCGAGGCCGCCAAGGCCCGAGGCGTGAAGTTTGGCCCCCGCCCTACCCTC

ACCCCGGCACAGATCGCGCACGCCCGCGAGCTGATCGACCAGGAAGGCCGCACCGTGAAAGAGGCGGCTG

CACTGCTTGGCGTGCATCGCTCGACCCTGTACCGCGCACTTGAGCGCAGCGAGGAAGTGACGCCCACCGA

GGCCAGGCGGCGCGGTGCCTTCCGTGAGGACGCATTGACCGAGGCCGACGCCCTGGCGGCCGCCGAGAAT

GAACGCCAAGAGGAACAAGCATGAAACCGCACCAGGACGGCCAGGACGAACCGTTTTTCATTACCGAAGA

GATCGAGGCGGAGATGATCGCGGCCGGGTACGTGTTCGAGCCGCCCGCGCACGTCTCAACCGTGCGGCTG

CATGAAATCCTGGCCGGTTTGTCTGATGCCAAGCTGGCGGCCTGGCCGGCCAGCTTGGCCGCTGAAGAAA

CCGAGCGCCGCCGTCTAAAAAGGTGATGTGTATTTGAGTAAAACAGCTTGCGTCATGCGGTCGCTGCGTA

TATGATGCGATGAGTAAATAAACAAATACGCAAGGGGAACGCATGAAGGTTATCGCTGTACTTAACCAGA

AAGGCGGGTCAGGCAAGACGACCATCGCAACCCATCTAGCCCGCGCCCTGCAACTCGCCGGGGCCGATGT

TCTGTTAGTCGATTCCGATCCCCAGGGCAGTGCCCGCGATTGGGCGGCCGTGCGGGAAGATCAACCGCTA

ACCGTTGTCGGCATCGACCGCCCGACGATTGACCGCGACGTGAAGGCCATCGGCCGGCGCGACTTCGTAG

TGATCGACGGAGCGCCCCAGGCGGCGGACTTGGCTGTGTCCGCGATCAAGGCAGCCGACTTCGTGCTGAT

TCCGGTGCAGCCAAGCCCTTACGACATATGGGCCACCGCCGACCTGGTGGAGCTGGTTAAGCAGCGCATT

GAGGTCACGGATGGAAGGCTACAAGCGGCCTTTGTCGTGTCGCGGGCGATCAAAGGCACGCGCATCGGCG

GTGAGGTTGCCGAGGCGCTGGCCGGGTACGAGCTGCCCATTCTTGAGTCCCGTATCACGCAGCGCGTGAG

CTACCCAGGCACTGCCGCCGCCGGCACAACCGTTCTTGAATCAGAACCCGAGGGCGACGCTGCCCGCGAG

GTCCAGGCGCTGGCCGCTGAAATTAAATCAAAACTCATTTGAGTTAATGAGGTAAAGAGAAAATGAGCAA

AAGCACAAACACGCTAAGTGCCGGCCGTCCGAGCGCACGCAGCAGCAAGGCTGCAACGTTGGCCAGCCTG

GCAGACACGCCAGCCATGAAGCGGGTCAACTTTCAGTTGCCGGCGGAGGATCACACCAAGCTGAAGATGT

ACGCGGTACGCCAAGGCAAGACCATTACCGAGCTGCTATCTGAATACATCGCGCAGCTACCAGAGTAAAT

GAGCAAATGAATAAATGAGTAGATGAATTTTAGCGGCTAAAGGAGGCGGCATGGAAAATCAAGAACAACC

AGGCACCGACGCCGTGGAATGCCCCATGTGTGGAGGAACGGGCGGTTGGCCAGGCGTAAGCGGCTGGGTT

GTCTGCCGGCCCTGCAATGGCACTGGAACCCCCAAGCCCGAGGAATCGGCGTGACGGTCGCAAACCATCC

GGCCCGGTACAAATCGGCGCGGCGCTGGGTGATGACCTGGTGGAGAAGTTGAAGGCCGCGCAGGCCGCCC

AGCGGCAACGCATCGAGGCAGAAGCACGCCCCGGTGAATCGTGGCAAGCGGCCGCTGATCGAATCCGCAA

AGAATCCCGGCAACCGCCGGCAGCCGGTGCGCCGTCGATTAGGAAGCCGCCCAAGGGCGACGAGCAACCA

GATTTTTTCGTTCCGATGCTCTATGACGTGGGCACCCGCGATAGTCGCAGCATCATGGACGTGGCCGTTT

TCCGTCTGTCGAAGCGTGACCGACGAGCTGGCGAGGTGATCCGCTACGAGCTTCCAGACGGGCACGTAGA

GGTTTCCGCAGGGCCGGCCGGCATGGCCAGTGTGTGGGATTACGACCTGGTACTGATGGCGGTTTCCCAT

CTAACCGAATCCATGAACCGATACCGGGAAGGGAAGGGAGACAAGCCCGGCCGCGTGTTCCGTCCACACG

TTGCGGACGTACTCAAGTTCTGCCGGCGAGCCGATGGCGGAAAGCAGAAAGACGACCTGGTAGAAACCTG

CATTCGGTTAAACACCACGCACGTTGCCATGCAGCGTACGAAGAAGGCCAAGAACGGCCGCCTGGTGACG

GTATCCGAGGGTGAAGCCTTGATTAGCCGCTACAAGATCGTAAAGAGCGAAACCGGGCGGCCGGAGTACA

TCGAGATCGAGCTAGCTGATTGGATGTACCGCGAGATCACAGAAGGCAAGAACCCGGACGTGCTGACGGT

TCACCCCGATTACTTTTTGATCGATCCCGGCATCGGCCGTTTTCTCTACCGCCTGGCACGCCGCGCCGCA

GGCAAGGCAGAAGCCAGATGGTTGTTCAAGACGATCTACGAACGCAGTGGCAGCGCCGGAGAGTTCAAGA

AGTTCTGTTTCACCGTGCGCAAGCTGATCGGGTCAAATGACCTGCCGGAGTACGATTTGAAGGAGGAGGC

GGGGCAGGCTGGCCCGATCCTAGTCATGCGCTACCGCAACCTGATCGAGGGCGAAGCATCCGCCGGTTCC

TAATGTACGGAGCAGATGCTAGGGCAAATTGCCCTAGCAGGGGAAAAAGGTCGAAAAGGTCTCTTTCCTG

TGGATAGCACGTACATTGGGAACCCAAAGCCGTACATTGGGAACCGGAACCCGTACATTGGGAACCCAAA

GCCGTACATTGGGAACCGGTCACACATGTAAGTGACTGATATAAAAGAGAAAAAAGGCGATTTTTCCGCC

TAAAACTCTTTAAAACTTATTAAAACTCTTAAAACCCGCCTGGCCTGTGCATAACTGTCTGGCCAGCGCA

CAGCCGAAGAGCTGCAAAAAGCGCCTACCCTTCGGTCGCTGCGCTCCCTACGCCCCGCCGCTTCGCGTCG

GCCTATCGCGGCCGCTGGCCGCTCAAAAATGGCTGGCCTACGGCCAGGCAATCTACCAGGGCGCGGACAA

GCCGCGCCGTCGCCACTCGACCGCCGGCGCCCACATCAAGGCACCCTGCCTCGCGCGTTTCGGTGATGAC

GGTGAAAACCTCTGACACATGCAGCTCCCGGAGACGGTCACAGCTTGTCTGTAAGCGGATGCCGGGAGCA

GACAAGCCCGTCAGGGCGCGTCAGCGGGTGTTGGCGGGTGTCGGGGCGCAGCCATGACCCAGTCACGTAG

CGATAGCGGAGTGTATACTGGCTTAACTATGCGGCATCAGAGCAGATTGTACTGAGAGTGCACCATATGC

GGTGTGAAATACCGCACAGATGCGTAAGGAGAAAATACCGCATCAGGCGCTCTTCCGCTTCCTCGCTCAC

TGACTCGCTGCGCTCGGTCGTTCGGCTGCGGCGAGCGGTATCAGCTCACTCAAAGGCGGTAATACGGTTA

TCCACAGAATCAGGGGATAACGCAGGAAAGAACATGTGAGCAAAAGGCCAGCAAAAGGCCAGGAACCGTA

AAAAGGCCGCGTTGCTGGCGTTTTTCCATAGGCTCCGCCCCCCTGACGAGCATCACAAAAATCGACGCTC

AAGTCAGAGGTGGCGAAACCCGACAGGACTATAAAGATACCAGGCGTTTCCCCCTGGAAGCTCCCTCGTG

CGCTCTCCTGTTCCGACCCTGCCGCTTACCGGATACCTGTCCGCCTTTCTCCCTTCGGGAAGCGTGGCGC

TTTCTCATAGCTCACGCTGTAGGTATCTCAGTTCGGTGTAGGTCGTTCGCTCCAAGCTGGGCTGTGTGCA

CGAACCCCCCGTTCAGCCCGACCGCTGCGCCTTATCCGGTAACTATCGTCTTGAGTCCAACCCGGTAAGA

CACGACTTATCGCCACTGGCAGCAGCCACTGGTAACAGGATTAGCAGAGCGAGGTATGTAGGCGGTGCTA

CAGAGTTCTTGAAGTGGTGGCCTAACTACGGCTACACTAGAAGGACAGTATTTGGTATCTGCGCTCTGCT

GAAGCCAGTTACCTTCGGAAAAAGAGTTGGTAGCTCTTGATCCGGCAAACAAACCACCGCTGGTAGCGGT

GGTTTTTTTGTTTGCAAGCAGCAGATTACGCGCAGAAAAAAAGGATCTCAAGAAGATCCTTTGATCTTTT

CTACGGGGTCTGACGCTCAGTGGAACGAAAACTCACGTTAAGGGATTTTGGTCATGCATTCTAGGTACTA

AAACAATTCATCCAGTAAAATATAATATTTTATTTTCTCCCAATCAGGCTTGATCCCCAGTAAGTCAAAA

AATAGCTCGACATACTGTTCTTCCCCGATATCCTCCCTGATCGACCGGACGCAGAAGGCAATGTCATACC

ACTTGTCCGCCCTGCCGCTTCTCCCAAGATCAATAAAGCCACTTACTTTGCCATCTTTCACAAAGATGTT

GCTGTCTCCCAGGTCGCCGTGGGAAAAGACAAGTTCCTCTTCGGGCTTTTCCGTCTTTAAAAAATCATAC

AGCTCGCGCGGATCTTTAAATGGAGTGTCTTCTTCCCAGTTTTCGCAATCCACATCGGCCAGATCGTTAT

TCAGTAAGTAATCCAATTCGGCTAAGCGGCTGTCTAAGCTATTCGTATAGGGACAATCCGATATGTCGAT

GGAGTGAAAGAGCCTGATGCACTCCGCATACAGCTCGATAATCTTTTCAGGGCTTTGTTCATCTTCATAC

TCTTCCGAGCAAAGGACGCCATCGGCCTCACTCATGAGCAGATTGCTCCAGCCATCATGCCGTTCAAAGT

GCAGGACCTTTGGAACAGGCAGCTTTCCTTCCAGCCATAGCATCATGTCCTTTTCCCGTTCCACATCATA

GGTGGTCCCTTTATACCGGCTGTCCGTCATTTTTAAATATAGGTTTTCATTTTCTCCCACCAGCTTATAT

ACCTTAGCAGGAGACATTCCTTCCGTATCTTTTACGCAGCGGTATTTTTCGATCAGTTTTTTCAATTCCG

GTGATATTCTCATTTTAGCCATTTATTATTTCCTTCCTCTTTTCTACAGTATTTAAAGATACCCCAAGAA

GCTAATTATAACAAGACGAACTCCAATTCACTGTTCCTTGCATTCTAAAACCTTAAATACCAGAAAACAG

CTTTTTCAAAGTTGTTTTCAAAGTTGGCGTATAACATAGTATCGACGGAGCCGATTTTGAAACCGCGGTG

ATCACAGGCAGCAACGCTCTGTCATCGTTACAATCAACATGCTACCCTCCGCGAGATCATCCGTGTTTCA

AACCCGGCAGCTTAGTTGCCGTTCTTCCGAATAGCATCGGTAACATGAGCAAAGTCTGCCGCCTTACAAC

GGCTCTCCCGCTGACGCCGTCCCGGACTGATGGGCTGCCTGTATCGAGTGGTGATTTTGTGCCGAGCTGC

CGGTCGGGGAGCTGTTGGCTGGCTGGTGGCAGGATATATTGTGGTGTAAACAAATTGACGCTTAGACAAC

TTAATAACACATTGCGGACGTTTTTAATGTACTGAATTAACGCCGAATTAATTCGGGGGATCTGGATTTT

AGTACTGGATTTTGGTTTTAGGAATTAGAAATTTTATTGATAGAAGTATTTTACAAATACAAATACATAC

TAAGGGTTTCTTATATGCTCAACACATGAGCGAAACCCTATAGGAACCCTAATTCCCTTATCTGGGAACT

ACTCACACATTATTATGGAGAAACTCGAGCTTGTCGATCGACAGATCCGGTCGGCATCTACTCTATTTCT

TTGCCCTCGGACGAGTGCTGGGGCGTCGGTTTCCACTATCGGCGAGTACTTCTACACAGCCATCGGTCCA

GACGGCCGCGCTTCTGCGGGCGATTTGTGTACGCCCGACAGTCCCGGCTCCGGATCGGACGATTGCGTCG

CATCGACCCTGCGCCCAAGCTGCATCATCGAAATTGCCGTCAACCAAGCTCTGATAGAGTTGGTCAAGAC

CAATGCGGAGCATATACGCCCGGAGTCGTGGCGATCCTGCAAGCTCCGGATGCCTCCGCTCGAAGTAGCG

CGTCTGCTGCTCCATACAAGCCAACCACGGCCTCCAGAAGAAGATGTTGGCGACCTCGTATTGGGAATCC

CCGAACATCGCCTCGCTCCAGTCAATGACCGCTGTTATGCGGCCATTGTCCGTCAGGACATTGTTGGAGC

CGAAATCCGCGTGCACGAGGTGCCGGACTTCGGGGCAGTCCTCGGCCCAAAGCATCAGCTCATCGAGAGC

CTGCGCGACGGACGCACTGACGGTGTCGTCCATCACAGTTTGCCAGTGATACACATGGGGATCAGCAATC

GCGCATATGAAATCACGCCATGTAGTGTATTGACCGATTCCTTGCGGTCCGAATGGGCCGAACCCGCTCG

TCTGGCTAAGATCGGCCGCAGCGATCGCATCCATAGCCTCCGCGACCGGTTGTAGAACAGCGGGCAGTTC

GGTTTCAGGCAGGTCTTGCAACGTGACACCCTGTGCACGGCGGGAGATGCAATAGGTCAGGCTCTCGCTA

AACTCCCCAATGTCAAGCACTTCCGGAATCGGGAGCGCGGCCGATGCAAAGTGCCGATAAACATAACGAT

CTTTGTAGAAACCATCGGCGCAGCTATTTACCCGCAGGACATATCCACGCCCTCCTACATCGAAGCTGAA

AGCACGAGATTCTTCGCCCTCCGAGAGCTGCATCAGGTCGGAGACGCTGTCGAACTTTTCGATCAGAAAC

TTCTCGACAGACGTCGCGGTGAGTTCAGGCTTTTTCATATCTCATTGCCCCCCGGGATCTGCGAAAGCTC

GAGAGAGATAGATTTGTAGAGAGAGACTGGTGATTTCAGCGTGTCCTCTCCAAATGAAATGAACTTCCTT

ATATAGAGGAAGGTCTTGCGAAGGATAGTGGGATTGTGCGTCATCCCTTACGTCAGTGGAGATATCACAT

CAATCCACTTGCTTTGAAGACGTGGTTGGAACGTCTTCTTTTTCCACGATGCTCCTCGTGGGTGGGGGTC

CATCTTTGGGACCACTGTCGGCAGAGGCATCTTGAACGATAGCCTTTCCTTTATCGCAATGATGGCATTT

GTAGGTGCCACCTTCCTTTTCTACTGTCCTTTTGATGAAGTGACAGATAGCTGGGCAATGGAATCCGAGG

AGGTTTCCCGATATTACCCTTTGTTGAAAAGTCTCAATAGCCCTTTGGTCTTCTGAGACTGTATCTTTGA

TATTCTTGGAGTAGACGAGAGTGTCGTGCTCCACCATGTTATCACATCAATCCACTTGCTTTGAAGACGT

GGTTGGAACGTCTTCTTTTTCCACGATGCTCCTCGTGGGTGGGGGTCCATCTTTGGGACCACTGTCGGCA

GAGGCATCTTGAACGATAGCCTTTCCTTTATCGCAATGATGGCATTTGTAGGTGCCACCTTCCTTTTCTA

CTGTCCTTTTGATGAAGTGACAGATAGCTGGGCAATGGAATCCGAGGAGGTTTCCCGATATTACCCTTTG

TTGAAAAGTCTCAATAGCCCTTTGGTCTTCTGAGACTGTATCTTTGATATTCTTGGAGTAGACGAGAGTG

TCGTGCTCCACCATGTTGGCAAGCTGCTCTAGCCAATACGCAAACCGCCTCTCCCCGCGCGTTGGCCGAT

TCATTAATGCAGCTGGCACGACAGGTTTCCCGACTGGAAAGCGGGCAGTGAGCGCAACGCAATTAATGTG

AGTTAGCTCACTCATTAGGCACCCCAGGCTTTACACTTTATGCTTCCGGCTCGTATGTTGTGTGGAATTG

TGAGCGGATAACAATTTCACACAGGAAACAGCTATGACCATGATTACGAATTCGAGCTCGGTACCCGGGG

ATCCTCTAGAGTCGACCTGCAGGCATGCAAGCTTGGCACTGGCCGTCGTTTTACAACGTCGTGACTGGGA

AAACCCTGGCGTTACCCAACTTAATCGCCTTGCAGCACATCCCCCTTTCGCCAGCTGGCGTAATAGCGAA

GAGGCCCGCACCGATCGCCCTTCCCAACAGTTGCGCAGCCTGAATGGCGAATGCTAGAGCAGCTTGAGCT

TGGATCAGATTGTCGTTTCCCGCCTTCAGTTTAGCTTCATGGAGTCAAAGATTCAAATAGAGGACCTAAC

AGAACTCGCCGTAAAGACTGGCGAACAGTTCATACAGAGTCTCTTACGACTCAATGACAAGAAGAAAATC

TTCGTCAACATGGTGGAGCACGACACACTTGTCTACTCCAAAAATATCAAAGATACAGTCTCAGAAGACC

AAAGGGCAATTGAGACTTTTCAACAAAGGGTAATATCCGGAAACCTCCTCGGATTCCATTGCCCAGCTAT

CTGTCACTTTATTGTGAAGATAGTGGAAAAGGAAGGTGGCTCCTACAAATGCCATCATTGCGATAAAGGA

AAGGCCATCGTTGAAGATGCCTCTGCCGACAGTGGTCCCAAAGATGGACCCCCACCCACGAGGAGCATCG

TGGAAAAAGAAGACGTTCCAACCACGTCTTCAAAGCAAGTGGATTGATGTGATATCTCCACTGACGTAAG

GGATGACGCACAATCCCACTATCCTTCGCAAGACCCTTCCTCTATATAAGGAAGTTCATTTCATTTGGAG

AGAACACGGGGGACTCTTGAC

>gi|15982214|emb|AJ311872.1|CVE311872 Cloning vector pHANNIBAL

TGACCAAGTCAGCTTGGCACTGGCCGTCGTTTTACAACGTCGTGACTGGGAAAACCCTGGCGTTACCCAA

CTTAATCGCCTTGCAGCACATCCCCCTTTCGCCAGCTGGCGTAATAGCGAAGAGGCCCGCACCGATCGCC

CTTCCCAACAGTTGCGCAGCCTGAATGGCGAATGGGAAATTGTAAACGTTAATATTTTGTTAATATTTTG

TTAAAATTCGCGTTAAATTTTTGTTAAATCAGCTCATTTTTTAACCAATAGGCCGAAATCGGCAAAATCC

CTTATAAATCAAAAGAATAGACCGAGATAGGGTTGAGTGTTGTTCCAGTTTGGAACAAGAGTCCACTATT

AAAGAACGTGGACTCCAACGTCAAAGGGCGAAAAACCGTCTATCAGGGCGATGGCCCACTACGTGAACCA

TCACCCTAATCAAGTTTTTTGGGGTCGAGGTGCCGTAAAGCACTAAATCGGAACCCTAAAGGGATGCCCC

GATTTAGAGCTTGACGGGGAAAGCCGGCGAACGTGGCGAGAAAGGAAGGGAAGAAAGCGAAAGGAGCGGG

CGCTAGGGCGCTGGCAAGTGTAGCGGTCACGCTGCGCGTAACCACCACACCCGCCGCGCTTAATGCGCCG

CTACAGGGCGCGTCAGGTGGCACTTTTCGGGGAAATGTGCGCGGAACCCCTATTTGTTTATTTTTCTAAA

TACATTCAAATATGTATCCGCTCATGAGACAATAACCCTGATAAATGCTTCAATAATATTGAAAAAGGAA

GAGTATGAGTATTCAACATTTCCGTGTCGCCCTTATTCCCTTTTTTGCGGCATTTTGCCTTCCTGTTTTT

GCTCACCCAGAAACGCTGGTGAAAGTAAAAGATGCTGAAGATCAGTTGGGTGCACGAGTGGGTTACATCG

AACTGGATCTCAACAGCGGTAAGATCCTTGAGAGTTTTCGCCCCGAAGAACGTTTTCCAATGATGAGCAC

TTTTAAAGTTCTGCTATGTGGCGCGGTATTATCCCGTATTGACGCCGGGCAAGAGCAACTCGGTCGCCGC

ATACACTATTCTCAGAATGACTTGGTTGAGTACTCACCAGTCACAGAAAAGCATCTTACGGATGGCATGA

CAGTAAGAGAATTATGCAGTGCTGCCATAACCATGAGTGATAACACTGCGGCCAACTTACTTCTGACAAC

GATCGGAGGACCGAAGGAGCTAACCGCTTTTTTGCACAACATGGGGGATCATGTAACTCGCCTTGATCGT

TGGGAACCGGAGCTGAATGAAGCCATACCAAACGACGAGCGTGACACCACGATGCCTGTAGCAATGGCAA

CAACGTTGCGCAAACTATTAACTGGCGAACTACTTACTCTAGCTTCCCGGCAACAATTAATAGACTGGAT

GGAGGCGGATAAAGTTGCAGGACCACTTCTGCGCTCGGCCCTTCCGGCTGGCTGGTTTATTGCTGATAAA

TCTGGAGCCGGTGAGCGTGGGTCTCGCGGTATCATTGCAGCACTGGGGCCAGATGGTAAGCCCTCCCGTA

TCGTAGTTATCTACACGACGGGGAGTCAGGCAACTATGGATGAACGAAATAGACAGATCGCTGAGATAGG

TGCCTCACTGATTAAGCATTGGTAACTGTCAGACCAAGTTTACTCATATATACTTTAGATTGATTTAAAA

CTTCATTTTTAATTTAAAAGGATCTAGGTGAAGATCCTTTTTGATAATCTCATGACCAAAATCCCTTAAC

GTGAGTTTTCGTTCCACTGAGCGTCAGACCCCGTAGAAAAGATCAAAGGATCTTCTTGAGATCCTTTTTT

TCTGCGCGTAATCTGCTGCTTGCAAACAAAAAAACCACCGCTACCAGCGGTGGTTTGTTTGCCGGATCAA

GAGCTACCAACTCTTTTTCCGAAGGTAACTGGCTTCAGCAGAGCGCAGATACCAAATACTGTCCTTCTAG

TGTAGCCGTAGTTAGGCCACCACTTCAAGAACTCTGTAGCACCGCCTACATACCTCGCTCTGCTAATCCT

GTTACCAGTGGCTGCTGCCAGTGGCGATAAGTCGTGTCTTACCGGGTTGGACTCAAGACGATAGTTACCG

GATAAGGCGCAGCGGTCGGGCTGAACGGGGGGTTCGTGCACACAGCCCAGCTTGGAGCGAACGACCTACA

CCGAACTGAGATACCTACAGCGTGAGCTATGAGAAAGCGCCACGCTTCCCGAAGGGAGAAAGGCGGACAG

GTATCCGGTAAGCGGCAGGGTCGGAACAGGAGAGCGCACGAGGGAGCTTCCAGGGGGAAACGCCTGGTAT

CTTTATAGTCCTGTCGGGTTTCGCCACCTCTGACTTGAGCGTCGATTTTTGTGATGCTCGTCAGGGGGGC

GGAGCCTATGGAAAAACGCCAGCAACGCGGCCTTTTTACGGTTCCTGGCCTTTTGCTGGCCTTTTGCTCA

CATGTTCTTTCCTGCGTTATCCCCTGATTCTGTGGATAACCGTATTACCGCCTTTGAGTGAGCTGATACC

GCTCGCCGCAGCCGAACGACCGAGCGCAGCGAGTCAGTGAGCGAGGAAGCGGAAGAGCGCCCAATACGCA

AACCGCCTCTCCCCGCGCGTTGGCCGATTCATTAATGCAGCTGGCACGACAGGTTTCCCGACTGGAAAGC

GGGCAGTGAGCGCAACGCAATTAATGTGAGTTAGCTCACTCATTAGGCACCCCAGGCTTTACACTTTATG

CTTCCGGCTCGTATGTTGTGTGGAATTGTGAGCGGATAACAATTTCACACAGGAAACAGCTATGACCATG

ATTACGAATTTGGCCAAGTCGGCCTCTAATACGACTCACTATAGGGAGCTCGTCGAGCGGCCGCTCGACG

AATTAATTCCAATCCCACAAAAATCTGAGCTTAACAGCACAGTTGCTCCTCTCAGAGCAGAATCGGGTAT

TCAACACCCTCATATCAACTACTACGTTGTGTATAACGGTCCACATGCCGGTATATACGATGACTGGGGT

TGTACAAAGGCGGCAACAAACGGCGTTCCCGGAGTTGCACACAAGAAATTTGCCACTATTACAGAGGCAA

GAGCAGCAGCTGACGCGTACACAACAAGTCAGCAAACAGACAGGTTGAACTTCATCCCCAAAGGAGAAGC

TCAACTCAAGCCCAAGAGCTTTGCTAAGGCCCTAACAAGCCCACCAAAGCAAAAAGCCCACTGGCTCACG

CTAGGAACCAAAAGGCCCAGCAGTGATCCAGCCCCAAAAGAGATCTCCTTTGCCCCGGAGATTACAATGG

ACGATTTCCTCTATCTTTACGATCTAGGAAGGAAGTTCGAAGGTGAAGGTGACGACACTATGTTCACCAC

TGATAATGAGAAGGTTAGCCTCTTCAATTTCAGAAAGAATGCTGACCCACAGATGGTTAGAGAGGCCTAC

GCAGCAGGTCTCATCAAGACGATCTACCCGAGTAACAATCTCCAGGAGATCAAATACCTTCCCAAGAAGG

TTAAAGATGCAGTCAAAAGATTCAGGACTAATTGCATCAAGAACACAGAGAAAGACATATTTCTCAAGAT

CAGAAGTACTATTCCAGTATGGACGATTCAAGGCTTGCTTCATAAACCAAGGCAAGTAATAGAGATTGGA

GTCTCTAAAAAGGTAGTTCCTACTGAATCTAAGGCCATGCATGGAGTCTAAGATTCAAATCGAGGATCTA

ACAGAACTCGCCGTGAAGACTGGCGAACAGTTCATACAGAGTCTTTTACGACTCAATGACAAGAAGAAAA

TCTTCGTCAACATGGTGGAGCACGACACTCTGGTCTACTCCAAAAATGTCAAAGATACAGTCTCAGAAGA

CCAAAGGGCTATTGAGACTTTTCAACAAAGGATAATTTCGGGAAACCTCCTCGGATTCCATTGCCCAGCT

ATCTGTCACTTCATCGAAAGGACAGTAGAAAAGGAAGGTGGCTCCTACAAATGCCATCATTGCGATAAAG

GAAAGGCTATCATTCAAGATCTCTCTGCCGACAGTGGTCCCAAAGATGGACCCCCACCCACGAGGAGCAT

CGTGGAAAAAGAAGACGTTCCAACCACGTCTTCAAAGCAAGTGGATTGATGTGACATCTCCACTGACGTA

AGGGATGACGCACAATCCCACTATCCTTCGCAAGACCCTTCCTCTATATAAGGAAGTTCATTTCATTTGG

AGAGGACACGCTCGAGGAATTCGGTACCCCAATTGGTAAGGAAATAATTATTTTCTTTTTTCCTTTTAGT

ATAAAATAGTTAAGTGATGTTAATTAGTATGATTATAATAATATAGTTGTTATAATTGTGAAAAAATAAT

TTATAAATATATTGTTTACATAAACAACATAGTAATGTAAAAAAATATGACAAGTGATGTGTAAGACGAA

GAAGATAAAAGTTGAGAGTAAGTATATTATTTTTAATGAATTTGATCGAACATGTAAGATGATATACTAG

CATTAATATTTGTTTTAATCATAATAGTAATTCTAGCTGGTTTGATGAATTAAATATCAATGATAAAATA

CTATAGTAAAAATAAGAATAAATAAATTAAAATAATATTTTTTTATGATTAATAGTTTATTATATAATTA

AATATCTATACCATTACTAAATATTTTAGTTTAAAAGTTAATAAATATTTTGTTAGAAATTCCAATCTGC

TTGTAATTTATCAATAAACAAAATATTAAATAACAAGCTAAAGTAACAAATAATATCAAACTAATAGAAA

CAGTAATCTAATGTAACAAAACATAATCTAATGCTAATATAACAAAGCGCAAGATCTATCATTTTATATA

GTATTATTTTCAATCAACATTCTTATTAATTTCTAAATAATACTTGTAGTTTTATTAACTTCTAAATGGA

TTGACTATTAATTAAATGAATTAGTCGAACATGAATAAACAAGGTAACATGATAGATCATGTCATTGTGT

TATCATTGATCTTACATTTGGATTGATTACAGTTGGGAAATTGGGTTCGAAATCGATAAGCTTGGATCCT

CTAGAGTCCTGCTTTAATGAGATATGCGAGACGCCTATGATCGCATGATATTTGCTTTCAATTCTGTTGT

GCACGTTGTAAAAAACCTGAGCATGTGTAGCTCAGATCCTTACCGCCGGTTTCGGTTCATTCTAATGAAT

ATATCACCCGTTACTATCGTATTTTTATGAATAATATTCTCCGTTCAATTTACTGATTGTACCCTACTAC

TTATATGTACAATATTAAAATGAAAACAATATATTGTGCTGAATAGGTTTATAGCGACATCTATGATAGA

GCGCCACAATAACAAACAATTGCGTTTTATTATTACAAATCCAATTTTAAAAAAAGCGGCAGAACCGGTC

AAACCTAAAAGACTGATTACATAAATCTTATTCAAATTTCAAAAGGCCCCAGGGGCTAGTATCTACGACA

CACCGAGCGGCGAACTAATAACGTTCACTGAAGGGAACTCCGGTTCCCCGCCGGCGCGCATGGGTGAGAT

TCCTTGAAGTTGAGTATTGGCCGTCCGCTCTACCGAAAGTTACGGGCACCATTCAACCCGGTCCAGCACG

GCGGCCGGGTAACCGACTTGCTGCCCCGAGAATTATGCAGCATTTTTTTGGTGTATGTGGGCCCCAAATG

AAGTGCAGGTCAAACCTTGACAGTGACGACAAATCGTTGGGCGGGTCCAGGGCGAATTTTGCGACAACAT

GTCGAGGCTCAGCAGGACCTGCAGGCATGCAAGCTAGCTTACTAGTGATGCATATTCTATAGTGTCACCT

AAATCTGCGGCCGC

>gi|15982216|emb|AJ311873.1|CVE311873 Cloning vector pKANNIBAL

TGACCAAGTCAGCTTGGCACTGGCCGTCGTTTTACAACGTCGTGACTGGGAAAACCCTGGCGTTACCCAA

CTTAATCGCCTTGCAGCACATCCCCCTTTCGCCAGCTGGCGTAATAGCGAAGAGGCCCGCACCGATCGCC

CTTCCCAACAGTTGCGCAGCCTGAATGGCGAATGGGAAATTGTAAACGTTAATATTTTGTTAATATTTTG

TTAAAATTCGCGTTAAATTTTTGTTAAATCAGCTCATTTTTTAACCAATAGGCCGAAATCGGCAAAATCC

CTTATAAATCAAAAGAATAGACCGAGATAGGGTTGAGTGTTGTTCCAGTTTGGAACAAGAGTCCACTATT

AAAGAACGTGGACTCCAACGTCAAAGGGCGAAAAACCGTCTATCAGGGCGATGGCCCACTACGTGAACCA

TCACCCTAATCAAGTTTTTTGGGGTCGAGGTGCCGTAAAGCACTAAATCGGAACCCTAAAGGGATGCCCC

GATTTAGAGCTTGACGGGGAAAGCCGGCGAACGTGGCGAGAAAGGAAGGGAAGAAAGCGAAAGGAGCGGG

CGCTAGGGCGCTGGCAAGTGTAGCGGTCACGCTGCGCGTAACCACCACACCCGCCGCGCTTAATGCGCCG

CTACAGGGCGCGTCAGGTGGCACTTTTCGGGGAAATGTGCGCGGAACCCCTATTTGTTTATTTTTCTAAA

TACATTCAAATATGTATCCGCTCATGAGACAATAACCCTGATAAATGCTTCAATAATATTGAAAAAGGAA

GAGTATGAGTATTCAACATTTCCGTGTCGCCCTTATTCCCTTTTTTGCGGCATTTTGCCTTCCTGTTTTT

GCTCACCCAGAAACGCTGGTGAAAGTAAAAGATGCTGAAGATCAGTTGGGTGCACGAGTGGGTTACATCG

AACTGGATCTCAACAGCGGTAAGATCCTTGAGAGTTTTCGCCCCGAAGAACGTTTTCCAATGATGAGCAC

TTTTTGCAAGGAACAGTGAATTGGAGTTCGTCTTGTTATAATTAGCTTCTTGGGGTATCTTTAAATACTG

TAGAAAAGAGGAAGGAAATAATAAATGGCTAAAATGAGAATATCACCGGAATTGAAAAAACTGATCGAAA

AATACCGCTGCGTAAAAGATACGGAAGGAATGTCTCCTGCTAAGGTATATAAGCTGGTGGGAGAAAATGA

AAACCTATATTTAAAAATGACGGACAGCCGGTATAAAGGGACCACCTATGATGTGGAACGGGAAAAGGAC

ATGATGCTATGGCTGGAAGGAAAGCTGCCTGTTCCAAAGGTCCTGCACTTTGAACGGCATGATGGCTGGA

GCAATCTGCTCATGAGTGAGGCCGATGGCGTCCTTTGCTCGGAAGAGTATGAAGATGAACAAAGCCCTGA

AAAGATTATCGAGCTGTATGCGGAGTGCATCAGGCTCTTTCACTCCATCGACATATCGGATTGTCCCTAT

ACGAATAGCTTAGACAGCCGCTTAGCCGAATTGGATTACTTACTGAATAACGATCTGGCCGATGTGGATT

GCGAAAACTGGGAAGAAGACACTCCATTTAAAGATCCGCGCGAGCTGTATGATTTTTTAAAGACGGAAAA

GCCCGAAGAGGAACTTGTCTTTTCCCACGGCGACCTGGGAGACAGCAACATCTTTGTGAAAGATGGCAAA

GTAAGTGGCTTTATTGATCTTGGGAGAAGCGGCAGGGCGGACAAGTGGTATGACATTGCCTTCTGCGTCC

GGTCGATCAGGGAGGATATCGGGGAAGAACAGTATGTCGAGCTATTTTTTGACTTACTGGGGATCAAGCC

TGATTGGGAGAAAATAAAATATTATATTTTACTGGATGAATTGTTTTAGTACCTAGAATGCATGACCAAA

ATCCCTTAACGTGAGTTTTCGTTCCACTGAGCGTCAGACCCCGTAAAAGGATCTAGGTGAAGATCCTTTT

TGATAATCTCATGACCAAAATCCCTTAACGTGAGTTTTCGTTCCACTGAGCGTCAGACCCCGTAGAAAAG

ATCAAAGGATCTTCTTGAGATCCTTTTTTTCTGCGCGTAATCTGCTGCTTGCAAACAAAAAAACCACCGC

TACCAGCGGTGGTTTGTTTGCCGGATCAAGAGCTACCAACTCTTTTTCCGAAGGTAACTGGCTTCAGCAG

AGCGCAGATACCAAATACTGTCCTTCTAGTGTAGCCGTAGTTAGGCCACCACTTCAAGAACTCTGTAGCA

CCGCCTACATACCTCGCTCTGCTAATCCTGTTACCAGTGGCTGCTGCCAGTGGCGATAAGTCGTGTCTTA

CCGGGTTGGACTCAAGACGATAGTTACCGGATAAGGCGCAGCGGTCGGGCTGAACGGGGGGTTCGTGCAC

ACAGCCCAGCTTGGAGCGAACGACCTACACCGAACTGAGATACCTACAGCGTGAGCTATGAGAAAGCGCC

ACGCTTCCCGAAGGGAGAAAGGCGGACAGGTATCCGGTAAGCGGCAGGGTCGGAACAGGAGAGCGCACGA

GGGAGCTTCCAGGGGGAAACGCCTGGTATCTTTATAGTCCTGTCGGGTTTCGCCACCTCTGACTTGAGCG

TCGATTTTTGTGATGCTCGTCAGGGGGGCGGAGCCTATGGAAAAACGCCAGCAACGCGGCCTTTTTACGG

TTCCTGGCCTTTTGCTGGCCTTTTGCTCACATGTTCTTTCCTGCGTTATCCCCTGATTCTGTGGATAACC

GTATTACCGCCTTTGAGTGAGCTGATACCGCTCGCCGCAGCCGAACGACCGAGCGCAGCGAGTCAGTGAG

CGAGGAAGCGGAAGAGCGCCCAATACGCAAACCGCCTCTCCCCGCGCGTTGGCCGATTCATTAATGCAGC

TGGCACGACAGGTTTCCCGACTGGAAAGCGGGCAGTGAGCGCAACGCAATTAATGTGAGTTAGCTCACTC

ATTAGGCACCCCAGGCTTTACACTTTATGCTTCCGGCTCGTATGTTGTGTGGAATTGTGAGCGGATAACA

ATTTCACACAGGAAACAGCTATGACCATGATTACGAATTTGGCCAAGTCGGCCTCTAATACGACTCACTA

TAGGGAGCTCGTCGAGCGGCCGCTCGACGAATTAATTCCAATCCCACAAAAATCTGAGCTTAACAGCACA

GTTGCTCCTCTCAGAGCAGAATCGGGTATTCAACACCCTCATATCAACTACTACGTTGTGTATAACGGTC

CACATGCCGGTATATACGATGACTGGGGTTGTACAAAGGCGGCAACAAACGGCGTTCCCGGAGTTGCACA

CAAGAAATTTGCCACTATTACAGAGGCAAGAGCAGCAGCTGACGCGTACACAACAAGTCAGCAAACAGAC

AGGTTGAACTTCATCCCCAAAGGAGAAGCTCAACTCAAGCCCAAGAGCTTTGCTAAGGCCCTAACAAGCC

CACCAAAGCAAAAAGCCCACTGGCTCACGCTAGGAACCAAAAGGCCCAGCAGTGATCCAGCCCCAAAAGA

GATCTCCTTTGCCCCGGAGATTACAATGGACGATTTCCTCTATCTTTACGATCTAGGAAGGAAGTTCGAA

GGTGAAGGTGACGACACTATGTTCACCACTGATAATGAGAAGGTTAGCCTCTTCAATTTCAGAAAGAATG

CTGACCCACAGATGGTTAGAGAGGCCTACGCAGCAGGTCTCATCAAGACGATCTACCCGAGTAACAATCT

CCAGGAGATCAAATACCTTCCCAAGAAGGTTAAAGATGCAGTCAAAAGATTCAGGACTAATTGCATCAAG

AACACAGAGAAAGACATATTTCTCAAGATCAGAAGTACTATTCCAGTATGGACGATTCAAGGCTTGCTTC

ATAAACCAAGGCAAGTAATAGAGATTGGAGTCTCTAAAAAGGTAGTTCCTACTGAATCTAAGGCCATGCA

TGGAGTCTAAGATTCAAATCGAGGATCTAACAGAACTCGCCGTGAAGACTGGCGAACAGTTCATACAGAG

TCTTTTACGACTCAATGACAAGAAGAAAATCTTCGTCAACATGGTGGAGCACGACACTCTGGTCTACTCC

AAAAATGTCAAAGATACAGTCTCAGAAGACCAAAGGGCTATTGAGACTTTTCAACAAAGGATAATTTCGG

GAAACCTCCTCGGATTCCATTGCCCAGCTATCTGTCACTTCATCGAAAGGACAGTAGAAAAGGAAGGTGG

CTCCTACAAATGCCATCATTGCGATAAAGGAAAGGCTATCATTCAAGATCTCTCTGCCGACAGTGGTCCC

AAAGATGGACCCCCACCCACGAGGAGCATCGTGGAAAAAGAAGACGTTCCAACCACGTCTTCAAAGCAAG

TGGATTGATGTGACATCTCCACTGACGTAAGGGATGACGCACAATCCCACTATCCTTCGCAAGACCCTTC

CTCTATATAAGGAAGTTCATTTCATTTGGAGAGGACACGCTCGAGGAATTCGGTACCCCAATTGGTAAGG

AAATAATTATTTTCTTTTTTCCTTTTAGTATAAAATAGTTAAGTGATGTTAATTAGTATGATTATAATAA

TATAGTTGTTATAATTGTGAAAAAATAATTTATAAATATATTGTTTACATAAACAACATAGTAATGTAAA

AAAATATGACAAGTGATGTGTAAGACGAAGAAGATAAAAGTTGAGAGTAAGTATATTATTTTTAATGAAT

TTGATCGAACATGTAAGATGATATACTAGCATTAATATTTGTTTTAATCATAATAGTAATTCTAGCTGGT

TTGATGAATTAAATATCAATGATAAAATACTATAGTAAAAATAAGAATAAATAAATTAAAATAATATTTT

TTTATGATTAATAGTTTATTATATAATTAAATATCTATACCATTACTAAATATTTTAGTTTAAAAGTTAA

TAAATATTTTGTTAGAAATTCCAATCTGCTTGTAATTTATCAATAAACAAAATATTAAATAACAAGCTAA

AGTAACAAATAATATCAAACTAATAGAAACAGTAATCTAATGTAACAAAACATAATCTAATGCTAATATA

ACAAAGCGCAAGATCTATCATTTTATATAGTATTATTTTCAATCAACATTCTTATTAATTTCTAAATAAT

ACTTGTAGTTTTATTAACTTCTAAATGGATTGACTATTAATTAAATGAATTAGTCGAACATGAATAAACA

AGGTAACATGATAGATCATGTCATTGTGTTATCATTGATCTTACATTTGGATTGATTACAGTTGGGAAAT

TGGGTTCGAAATCGATAAGCTTGGATCCTCTAGAGTCCTGCTTTAATGAGATATGCGAGACGCCTATGAT

CGCATGATATTTGCTTTCAATTCTGTTGTGCACGTTGTAAAAAACCTGAGCATGTGTAGCTCAGATCCTT

ACCGCCGGTTTCGGTTCATTCTAATGAATATATCACCCGTTACTATCGTATTTTTATGAATAATATTCTC

CGTTCAATTTACTGATTGTACCCTACTACTTATATGTACAATATTAAAATGAAAACAATATATTGTGCTG

AATAGGTTTATAGCGACATCTATGATAGAGCGCCACAATAACAAACAATTGCGTTTTATTATTACAAATC

CAATTTTAAAAAAAGCGGCAGAACCGGTCAAACCTAAAAGACTGATTACATAAATCTTATTCAAATTTCA

AAAGGCCCCAGGGGCTAGTATCTACGACACACCGAGCGGCGAACTAATAACGTTCACTGAAGGGAACTCC

GGTTCCCCGCCGGCGCGCATGGGTGAGATTCCTTGAAGTTGAGTATTGGCCGTCCGCTCTACCGAAAGTT

ACGGGCACCATTCAACCCGGTCCAGCACGGCGGCCGGGTAACCGACTTGCTGCCCCGAGAATTATGCAGC

ATTTTTTTGGTGTATGTGGGCCCCAAATGAAGTGCAGGTCAAACCTTGACAGTGACGACAAATCGTTGGG

CGGGTCCAGGGCGAATTTTGCGACAACATGTCGAGGCTCAGCAGGACCTGCAGGCATGCAAGCTAGCTTA

CTAGTGATGCATATTCTATAGTGTCACCTAAATCTGCGGCCGC

>gi|15982218|emb|AJ311874.1|CVE311874 Cloning vector pHELLSGATE

GGCCGCACTAGTGATATCCCGCGGCCATGGCGGCCGGGAGCATGCGACGTCGGGCCCAATTCGCCCTATA

GTGAGTCGTATTACAATTCACTGGCCGTCGTTTTACAACGTCGTGACTGGGAAAACCCTGGCGTTACCCA

ACTTAATCGCCTTGCAGCACATCCCCCTTTCGCCAGCTGGCGTAATAGCGAAGAGGCCCGCACCGATCGC

CCTTCCCAACAGTTGCGCAGCCTGAATGGCGAATGGAAATTGTAAACGTTAATGGGTTTCTGGAGTTTAA

TGAGCTAAGCACATACGTCAGAAACCATTATTGCGCGTTCAAAAGTCGCCTAAGGTCACTATCAGCTAGC

AAATATTTCTTGTCAAAAATGCTCCACTGACGTTCCATAAATTCCCCTCGGTATCCAATTAGAGTCTCAT

ATTCACTCTCAATCCAAATAATCTGCAATGGCAATTACCTTATCCGCAACTTCTTTACCTATTTCCGCCC

GGATCCGGGCAGGTTCTCCGGCCGCTTGGGTGGAGAGGCTATTCGGCTATGACTGGGCACAACAGACAAT

CGGCTGCTCTGATGCCGCCGTGTTCCGGCTGTCAGCGCAGGGGCGCCCGGTTCTTTTTGTCAAGACCGAC

CTGTCCGGTGCCCTGAATGAACTGCAGGACGAGGCAGCGCGGCTATCGTGGCTGGCCACGACGGGCGTTC

CTTGCGCAGCTGTGCTCGACGTTGTCACTGAAGCGGGAAGGGACTGGCTGCTATTGGGCGAAGTGCCGGG

GCAGGATCTCCTGTCATCTCACCTTGCTCCTGCCGAGAAAGTATCCATCATGGCTGATGCAATGCGGCGG

CTGCATACGCTTGATCCGGCTACCTGCCCATTCGACCACCAAGCGAAACATCGCATCGAGCGAGCACGTA

CTCGGATGGAAGCCGGTCTTGTCGATCAGGATGATCTGGACGAAGAGCATCAGGGGCTCGCGCCAGCCGA

ACTGTTCGCCAGGCTCAAGGCGCGCATGCCCGACGGCGAGGATCTCGTCGTGACCCATGGCGATGCCTGC

TTGCCGAATATCATGGTGGAAAATGGCCGCTTTTCTGGATTCATCGACTGTGGCCGGCTGGGTGTGGCGG

ACCGCTATCAGGACATAGCGTTGGCTACCCGTGATATTGCTGAAGAGCTTGGCGGCGAATGGGCTGACCG

CTTCCTCGTGCTTTACGGTATCGCCGCTCCCGATTCGCAGCGCATCGCCTTCTATCGCCTTCTTGACGAG

TTCTTCTGAGCGGGACTCTGGGGTTCGAAATGACCGACCAAGCGACGCCCAACCTGCCATCACGAGATTT

CGATTCCACCGCCGCCTTCTATGAAAGGTTGGGCTTCGGAATCGTTTTCCGGGACGCCGGCTGGATGATC

CTCCAGCGCGGGGATCTCATGCTGGAGTTCTTCGCCCACCCCGATCCAACACTTACGTTTGCAACGTCCA

AGAGCAAATAGACCACGAACGCCGGAAGGTTGCCGCAGCGTGTGGATTGCGTCTCAATTCTCTCTTGCAG

GAATGCAATGATGAATATGATACTGACTATGAAACTTTGAGGGAATACTGCCTAGCACCGTCACCTCATA

ACGTGCATCATGCATGCCCTGACAACATGGAACATCGCTATTTTTCTGAAGAATTATGCTCGTTGGAGGA

TGTCGCGGCAATTGCAGCTATTGCCAACATCGAACTACCCCTCACGCATGCATTCATCAATATTATTCAT

GCGGGGAAAGGCAAGATTAATCCAACTGGCAAATCATCCAGCGTGATTGGTAACTTCAGTTCCAGCGACT

TGATTCGTTTTGGTGCTACCCACGTTTTCAATAAGGACGAGATGGTGGAGTAAAGAAGGAGTGCGTCGAA

GCAGATCGTTCAAACATTTGGCAATAAAGTTTCTTAAGATTGAATCCTGTTGCCGGTCTTGCGATGATTA

TCATATAATTTCTGTTGAATTACGTTAAGCATGTAATAATTAACATGTAATGCATGACGTTATTTATGAG

ATGGGTTTTTATGATTAGAGTCCCGCAATTATACATTTAATACGCGATAGAAAACAAAATATAGCGCGCA

AACTAGGATAAATTATCGCGCGCGGTGTCATCTATGTTACTAGATCGAATTAATTCCAGGCGGTGAAGGG

CAATCAGCTGTTGCCCGTCTCACTGGTGAAAAGAAAAACCACCCCAGTACATTAAAAACGTCCGCAATGT

GTTATTAAGTTGTCTAAGCGTCAATTTGTTTACACCACAATATATCCTGCCACCAGCCAGCCAACAGCTC

CCCGACCGGCAGCTCGGCACAAAATCACCACTCGATACAGGCAGCCCATCAGTCCGGGACGGCGTCAGCG

GGAGAGCCGTTGTAAGGCGGCAGACTTTGCTCATGTTACCGATGCTATTCGGAAGAACGGCAACTAAGCT

GCCGGGTTTGAAACACGGATGATCTCGCGGAGGGTAGCATGTTGATTGTAACGATGACAGAGCGTTGCTG

CCTGTGATCAAATATCATCTCCCTCGCAGAGATCCGAATTATCAGCCTTCTTATTCATTTCTCGCTTAAC

CGTGACAGGCTGTCGATCTTGAGAACTATGCCGACATAATAGGAAATCGCTGGATAAAGCCGCTGAGGAA

GCTGAGTGGCGCTATTTCTTTAGAAGTGAACGTTGACGATGTCGACGGATCTTTTCCGCTGCATAACCCT

GCTTCGGGGTCATTATAGCGATTTTTTCGGTATATCCATCCTTTTTCGCACGATATACAGGATTTTGCCA

AAGGGTTCGTGTAGACTTTCCTTGGTGTATCCAACGGCGTCAGCCGGGCAGGATAGGTGAAGTAGGCCCA

CCCGCGAGCGGGTGTTCCTTCTTCACTGTCCCTTATTCGCACCTGGCGGTGCTCAACGGGAATCCTGCTC

TGCGAGGCTGGCCGGCTACCGCCGGCGTAACAGATGAGGGCAAGCGGATGGCTGATGAAACCAAGCCAAC

CAGGGGTGATGCTGCCAACTTACTGATTTAGTGTATGATGGTGTTTTTGAGGTGCTCCAGTGGCTTCTGT

TTCTATCAGCTGTCCCTCCTGTTCAGCTACTGACGGGGTGGTGCGTAACGGCAAAAGCACCGCCGGACAT

CAGCGCTATCTCTGCTCTCACTGCCGTAAAACATGGCAACTGCAGTTCACTTACACCGCTTCTCAACCCG

GTACGCACCAGAAAATCATTGATATGGCCATGAATGGCGTTGGATGCCGGGCAACAGCCCGCATTATGGG

CGTTGGCCTCAACACGATTTTACGTCACTTAAAAAACTCAGGCCGCAGTCGGTAACCTCGCGCATACAGC

CGGGCAGTGACGTCATCGTCTGCGCGGAAATGGACGAACAGTGGGGCTATGTCGGGGCTAAATCGCGCCA

GCGCTGGCTGTTTTACGCGTATGACAGTCTCCGGAAGACGGTTGTTGCGCACGTATTCGGTGAACGCACT

ATGGCGACGCTGGGGCGTCTTATGAGCCTGCTGTCACCCTTTGACGTGGTGATATGGATGACGGATGGCT

GGCCGCTGTATGAATCCCGCCTGAAGGGAAAGCTGCACGTAATCAGCAAGCGATATACGCAGCGAATTGA

GCGGCATAACCTGAATCTGAGGCAGCACCTGGCACGGCTGGGACGGAAGTCGCTGTCGTTCTCAAAATCG

GTGGAGCTGCATGACAAAGTCATCGGGCATTATCTGAACATAAAACACTATCAATAAGTTGGAGTCATTA

CCCAACCAGGAAGGGCAGCCCACCTATCAAGGTGTACTGCCTTCCAGACGAACGAAGAGCGATTGAGGAA

AAGGCGGCGGCGGCCGGCATGAGCCTGTCGGCCTACCTGCTGGCCGTCGGCCAGGGCTACAAAATCACGG

GCGTCGTGGACTATGAGCACGTCCGCGAGCTGGCCCGCATCAATGGCGACCTGGGCCGCCTGGGCGGCCT

GCTGAAACTCTGGCTCACCGACGACCCGCGCACGGCGCGGTTCGGTGATGCCACGATCCTCGCCCTGCTG

GCGAAGATCGAAGAGAAGCAGGACGAGCTTGGCAAGGTCATGATGGGCGTGGTCCGCCCGAGGGCAGAGC

CATGACTTTTTTAGCCGCTAAAACGGCCGGGGGGTGCGCGTGATTGCCAAGCACGTCCCCATGCGCTCCA

TCAAGAAGAGCGACTTCGCGGAGCTGGTATTCGTGCAGGGCAAGATTCGGAATACCAAGTACGAGAAGGA

CGGCCAGACGGTCTACGGGACCGACTTCATTGCCGATAAGGTGGATTATCTGGACACCAAGGCACCAGGC

GGGTCAAATCAGGAATAAGGGCACATTGCCCCGGCGTGAGTCGGGGCAATCCCGCAAGGAGGGTGAATGA

ATCGGACGTTTGACCGGAAGGCATACAGGCAAGAACTGATCGACGCGGGGTTTTCCGCCGAGGATGCCGA

AACCATCGCAAGCCGCACCGTCATGCGTGCGCCCCGCGAAACCTTCCAGTCCGTCGGCTCGATGGTCCAG

CAAGCTACGGCCAAGATCGAGCGCGACAGCGTGCAACTGGCTCCCCCTGCCCTGCCCGCGCCATCGGCCG

CCGTGGAGCGTTCGCGTCGTCTCGAACAGGAGGCGGCAGGTTTGGCGAAGTCGATGACCATCGACACGCG

AGGAACTATGACGACCAAGAAGCGAAAAACCGCCGGCGAGGACCTGGCAAAACAGGTCAGCGAGGCCAAG

CAGGCCGCGTTGCTGAAACACACGAAGCAGCAGATCAAGGAAATGCAGCTTTCCTTGTTCGATATTGCGC

CGTGGCCGGACACGATGCGAGCGATGCCAAACGACACGGCCCGCTCTGCCCTGTTCACCACGCGCAACAA

GAAAATCCCGCGCGAGGCGCTGCAAAACAAGGTCATTTTCCACGTCAACAAGGACGTGAAGATCACCTAC

ACCGGCGTCGAGCTGCGGGCCGACGATGACGAACTGGTGTGGCAGCAGGTGTTGGAGTACGCGAAGCGCA

CCCCTATCGGCGAGCCGATCACCTTCACGTTCTACGAGCTTTGCCAGGACCTGGGCTGGTCGATCAATGG

CCGGTATTACACGAAGGCCGAGGAATGCCTGTCGCGCCTACAGGCGACGGCGATGGGCTTCACGTCCGAC

CGCGTTGGGCACCTGGAATCGGTGTCGCTGCTGCACCGCTTCCGCGTCCTGGACCGTGGCAAGAAAACGT

CCCGTTGCCAGGTCCTGATCGACGAGGAAATCGTCGTGCTGTTTGCTGGCGACCACTACACGAAATTCAT

ATGGGAGAAGTACCGCAAGCTGTCGCCGACGGCCCGACGGATGTTCGACTATTTCAGCTCGCACCGGGAG

CCGTACCCGCTCAAGCTGGAAACCTTCCGCCTCATGTGCGGATCGGATTCCACCCGCGTGAAGAAGTGGC

GCGAGCAGGTCGGCGAAGCCTGCGAAGAGTTGCGAGGCAGCGGCCTGGTGGAACACGCCTGGGTCAATGA

TGACCTGGTGCATTGCAAACGCTAGGGCCTTGTGGGGTCAGTTCCGGCTGGGGGTTCAGCAGCCAGCGCT

TTACTGGCATTTCAGGAACAAGCGGGCACTGCTCGACGCACTTGCTTCGCTCAGTATCGCTCGGGACGCA

CGGCGCGCTCTACGAACTGCCGATAAACAGAGGATTAAAATTGACAATTGTGATTAAGGCTCAGATTCGA

CGGCTTGGAGCGGCCGACGTGCAGGATTTCCGCGAGATCCGATTGTCGGCCCTGAAGAAAGCTCCAGAGA

TGTTCGGGTCCGTTTACGAGCACGAGGAGAAAAAGCCCATGGAGGCGTTCGCTGAACGGTTGCGAGATGC

CGTGGCATTCGGCGCCTACATCGACGGCGAGATCATTGGGCTGTCGGTCTTCAAACAGGAGGACGGCCCC

AAGGACGCTCACAAGGCGCATCTGTCCGGCGTTTTCGTGGAGCCCGAACAGCGAGGCCGAGGGGTCGCCG

GTATGCTGCTGCGGGCGTTGCCGGCGGGTTTATTGCTCGTGATGATCGTCCGACAGATTCCAACGGGAAT

CTGGTGGATGCGCATCTTCATCCTCGGCGCACTTAATATTTCGCTATTCTGGAGCTTGTTGTTTATTTCG

GTCTACCGCCTGCCGGGCGGGGTCGCGGCGACGGTAGGCGCTGTGCAGCCGCTGATGGTCGTGTTCATCT

CTGCCGCTCTGCTAGGTAGCCCGATACGATTGATGGCGGTCCTGGGGGCTATTTGCGGAACTGCGGGCGT

GGCGCTGTTGGTGTTGACACCAAACGCAGCGCTAGATCCTGTCGGCGTCGCAGCGGGCCTGGCGGGGGCG

GTTTCCATGGCGTTCGGAACCGTGCTGACCCGCAAGTGGCAACCTCCCGTGCCTCTGCTCACCTTTACCG

CCTGGCAACTGGCGGCCGGAGGACTTCTGCTCGTTCCAGTAGCTTTAGTGTTTGATCCGCCAATCCCGAT

GCCTACAGGAACCAATGTTCTCGGCCTGGCGTGGCTCGGCCTGATCGGAGCGGGTTTAACCTACTTCCTT

TGGTTCCGGGGGATCTCGCGACTCGAACCTACAGTTGTTTCCTTACTGGGCTTTCTCAGCCGGGATGGCG

CTAAGAAGCTATTGCCGCCGATCTTCATATGCGGTGTGAAATACCGCACAGATGCGTAAGGAGAAAATAC

CGCATCAGGCGCTCTTCCGCTTCCTCGCTCACTGACTCGCTGCGCTCGGTCGTTCGGCTGCGGCGAGCGG

TATCAGCTCACTCAAAGGCGGTAATACGGTTATCCACAGAATCAGGGGATAACGCAGGAAAGAACATGTG

AGCAAAAGGCCAGCAAAAGGCCAGGAACCGTAAAAAGGCCGCGTTGCTGGCGTTTTTCCATAGGCTCCGC

CCCCCTGACGAGCATCACAAAAATCGACGCTCAAGTCAGAGGTGGCGAAACCCGACAGGACTATAAAGAT

ACCAGGCGTTTCCCCCTGGAAGCTCCCTCGTGCGCTCTCCTGTTCCGACCCTGCCGCTTACCGGATACCT

GTCCGCCTTTCTCCCTTCGGGAAGCGTGGCGCTTTCTCAATGCTCACGCTGTAGGTATCTCAGTTCGGTG

TAGGTCGTTCGCTCCAAGCTGGGCTGTGTGCACGAACCCCCCGTTCAGCCCGACCGCTGCGCCTTATCCG

GTAACTATCGTCTTGAGTCCAACCCGGTAAGACACGACTTATCGCCACTGGCAGCAGCCACTGGTAACAG

GATTAGCAGAGCGAGGTATGTAGGCGGTGCTACAGAGTTCTTGAAGTGGTGGCCTAACTACGGCTACACT

AGAAGGACAGTATTTGGTATCTGCGCTCTGCTGAAGCCAGTTACCTTCGGAAAAAGAGTTGGTAGCTCTT

GATCCGGCAAACAAACCACCGCTGGTAGCGGTGGTTTTTTTGTTTGCAAGCAGCAGATTACGCGCAGAAA

AAAAGGATATCAAGAAGATCCTTTGATCTTTTCTACGGGGTCTGACGCTCAGTGGAACGAAAACTCACGT

TAAGGGATTTTGGTCATGAGATTATCAAAAAGGATCTTCACCTAGATCCTTTTAAATTAAAAATGAAGTT

TTAAATCAATCTAAAGTATATATGAGTAAACTTGGTCTGACAGTTACCAATGCTTAATCAGTGAGGCACC

TATCTCAGCGATCTGTCTATTTCGTTCATCCATAGTTGCCTGACTCCCCGTCGTGTAGATAACTACGATA

CGGGAGGGCTTACCATCTGGCCCCAGTGCTGCAATGATACCGCGAGACCCACGCTCACCGGCTCCAGATT

TATCAGCAATAAACCAGCCAGCCGGAAGGGCCGAGCGCAGAAGTGGTCCTGCAACTTTATCCGCCTCCAT

CCAGTCTATTAAACAAGTGGCAGCAACGGATTCGCAAACCTGTCACGCCTTTTGTGCCAAAAGCCGCGCC

AGGTTTGCGATCCGCTGTGCCAGGCGTTAGGCGTCATATGAAGATTTCGGTGATCCCTGAGCAGGTGGCG

GAAACATTGGATGCTGAGAACCATTTCATTGTTCGTGAAGTGTTCGATGTGCACCTATCCGACCAAGGCT

TTGAACTATCTACCAGAAGTGTGAGCCCCTACCGGAAGGATTACATCTCGGATGATGACTCTGATGAAGA

CTCTGCTTGCTATGGCGCATTCATCGACCAAGAGCTTGTCGGGAAGATTGAACTCAACTCAACATGGAAC

GATCTAGCCTCTATCGAACACATTGTTGTGTCGCACACGCACCGAGGCAAAGGAGTCGCGCACAGTCTCA

TCGAATTTGCGAAAAAGTGGGCACTAAGCAGACAGCTCCTTGGCATACGATTAGAGACACAAACGAACAA

TGTACCTGCCTGCAATTTGTACGCAAAATGTGGCTTTACTCTCGGCGGCATTGACCTGTTCACGTATAAA

ACTAGACCTCAAGTCTCGAACGAAACAGCGATGTACTGGTACTGGTTCTCGGGAGCACAGGATGACGCCT

AACAATTCATTCAAGCCGACACCGCTTCGCGGCGCGGCTTAATTCAGGAGTTAAACATCATGAGGGAAGC

GGTGATCGCCGAAGTATCGACTCAACTATCAGAGGTAGTTGGCGTCATCGAGCGCCATCTCGAACCGACG

TTGCTGGCCGTACATTTGTACGGCTCCGCAGTGGATGGCGGCCTGAAGCCACACAGTGATATTGATTTGC

TGGTTACGGTGACCGTAAGGCTTGATGAAACAACGCGGCGAGCTTTGATCAACGACCTTTTGGAAACTTC

GGCTTCCCCTGGAGAGAGCGAGATTCTCCGCGCTGTAGAAGTCACCATTGTTGTGCACGACGACATCATT

CCGTGGCGTTATCCAGCTAAGCGCGAACTGCAATTTGGAGAATGGCAGCGCAATGACATTCTTGCAGGTA

TCTTCGAGCCAGCCACGATCGACATTGATCTGGCTATCTTGCTGACAAAAGCAAGAGAACATAGCGTTGC

CTTGGTAGGTCCAGCGGCGGAGGAACTCTTTGATCCGGTTCCTGAACAGGATCTATTTGAGGCGCTAAAT

GAAACCTTAACGCTATGGAACTCGCCGCCCGACTGGGCTGGCGATGAGCGAAATGTAGTGCTTACGTTGT

CCCGCATTTGGTACAGCGCAGTAACCGGCAAAATCGCGCCGAAGGATGTCGCTGCCGACTGGGCAATGGA

GCGCCTGCCGGCCCAGTATCAGCCCGTCATACTTGAAGCTAGGCAGGCTTATCTTGGACAAGAAGATCGC

TTGGCCTCGCGCGCAGATCAGTTGGAAGAATTTGTTCACTACGTGAAAGGCGAGATCACCAAGGTAGTCG

GCAAATAATGTCTAACAATTCGTTCAAGCCGACGCCGCTTCGCGGCGCGGCTTAACTCAAGCGTTAGAGA

GCTGGGGAAGACTATGCGCGATCTGTTGAAGGTGGTTCTAAGCCTCGTACTTGCGATGGCATCGGGGCAG

GCACTTGCTGACCTGCCAATTGTTTTAGTGGATGAAGCTCGTCTTCCCTATGACTACTCCCCATCCAACT

ACGACATTTCTCCAAGCAACTACGACAACTCCATAAGCAATTACGACAATAGTCCATCAAATTACGACAA

CTCTGAGAGCAACTACGATAATAGTTCATCCAATTACGACAATAGTCGCAACGGAAATCGTAGGCTTATA

TATAGCGCAAATGGGTCTCGCACTTTCGCCGGCTACTACGTCATTGCCAACAATGGGACAACGAACTTCT

TTTCCACATCTGGCAAAAGGATGTTCTACACCCCAAAAGGGGGGCGCGGCGTCTATGGCGGCAAAGATGG

GAGCTTCTGCGGGGCATTGGTCGTCATAAATGGCCAATTTTCGCTTGCCCTGACAGATAACGGCCTGAAG

ATCATGTATCTAAGCAACTAGCCTGCTCTCTAATAAAATGTTAGGAGCTTGGCTGCCATTTTTGGGGTGA

GGCCGTTCGCGGCCGAGGGGCGCAGCCCCTGGGGGGATGGGAGGCCCGCGTTAGCGGGCCGGGAGGGTTC

GAGAAGGGGGGGCACCCCCCTTCGGCGTGCGCGGTCACGCGCCAGGGCGCAGCCCTGGTTAAAAACAAGG

TTTATAAATATTGGTTTAAAAGCAGGTTAAAAGACAGGTTAGCGGTGGCCGAAAAACGGGCGGAAACCCT

TGCAAATGCTGGATTTTCTGCCTGTGGACAGCCCCTCAAATGTCAATAGGTGCGCCCCTCATCTGTCAGC

ACTCTGCCCCTCAAGTGTCAAGGATCGCGCCCCTCATCTGTCAGTAGTCGCGCCCCTCAAGTGTCAATAC

CGCAGGGCACTTATCCCCAGGCTTGTCCACATCATCTGTGGGAAACTCGCGTAAAATCAGGCGTTTTCGC

CGATTTGCGAGGCTGGCCAGCTCCACGTCGCCGGCCGAAATCGAGCCTGCCCCTCATCTGTCAACGCCGC

GCCGGGTGAGTCGGCCCCTCAAGTGTCAACGTCCGCCCCTCATCTGTCAGTGAGGGCCAAGTTTTCCGCG

AGGTATCCACAACGCCGGCGGCCGGCCGCGGTGTCTCGCACACGGCTTCGACGGCGTTTCTGGCGCGTTT

GCAGGGCCATAGACGGCCGCCAGCCCAGCGGCGAGGGCAACCAGCCCGGTGAGCGTCGGAAAGGGTCGAC

ATCTTGCTGCGTTCGGATATTTTCGTGGAGTTCCCGCCACAGACCCGGATTGAAGGCGAGATCCAGCAAC

TCGCGCCAGATCATCCTGTGACGGAACTTTGGCGCGTGATGACTGGCCAGGACGTCGGCCGAAAGAGCGA

CAAGCAGATCACGATTTTCGACAGCGTCGGATTTGCGATCGAGGATTTTTCGGCGCTGCGCTACGTCCGC

GACCGCGTTGAGGGATCAAGCCACAGCAGCCCACTCGACCTTCTAGCCGACCCAGACGAGCCAAGGGATC

TTTTTGGAATGCTGCTCCGTCGTCAGGCTTTCCGACGTTTGGGTGGTTGAACAGAAGTCATTATCGTACG

GAATGCCAGCACTCCCGAGGGGAACCCTGTGGTTGGCATGCACATACAAATGGACGAACGGATAAACCTT

TTCACGCCCTTTTAAATATCCGTTATTCTAATAAACGCTCTTTTCTCTTAGGTTTACCCGCCAATATATC

CTGTCAAACACTGATAGTTTAAACTGAAGGCGGGAAACGACAATCTGATCATGAGCGGAGAATTAAGGGA

GTCACGTTATGACCCCCGCCGATGACGCGGGACAAGCCGTTTTACGTTTGGAACTGACAGAACCGCAACG

ATTGAAGGAGCCACTCAGCCCCAATACGCAAACCGCCTCTCCCCGCGCGTTGGCCGATTCATTAATGCAG

CTGGCACGACAGGTTTCCCGACTGGAAAGCGGGCAGTGAGCGCAACGCAATTAATGTGAGTTAGCTCACT

CATTAGGCACCCCAGGCTTTACACTTTATGCTTCCGGCTCGTATGTTGTGTGGAATTGTGAGCGGATAAC

AATTTCACACAGGAAACAGCTATGACCATGATTACGCCAAGCTATTTAGGTGACACTATAGAATACTCAA

GCTATGCATCCAACGCGTTGGGAGCTCTCCCATATCGACCTGCAGGCGGCCGCTCGACGAATTAATTCCA

ATCCCACAAAAATCTGAGCTTAACAGCACAGTTGCTCCTCTCAGAGCAGAATCGGGTATTCAACACCCTC

ATATCAACTACTACGTTGTGTATAACGGTCCACATGCCGGTATATACGATGACTGGGGTTGTACAAAGGC

GGCAACAAACGGCGTTCCCGGAGTTGCACACAAGAAATTTGCCACTATTACAGAGGCAAGAGCAGCAGCT

GACGCGTACACAACAAGTCAGCAAACAGACAGGTTGAACTTCATCCCCAAAGGAGAAGCTCAACTCAAGC

CCAAGAGCTTTGCTAAGGCCCTAACAAGCCCACCAAAGCAAAAAGCCCACTGGCTCACGCTAGGAACCAA

AAGGCCCAGCAGTGATCCAGCCCCAAAAGAGATCTCCTTTGCCCCGGAGATTACAATGGACGATTTCCTC

TATCTTTACGATCTAGGAAGGAAGTTCGAAGGTGAAGGTGACGACACTATGTTCACCACTGATAATGAGA

AGGTTAGCCTCTTCAATTTCAGAAAGAATGCTGACCCACAGATGGTTAGAGAGGCCTACGCAGCAGGTCT

CATCAAGACGATCTACCCGAGTAACAATCTCCAGGAGATCAAATACCTTCCCAAGAAGGTTAAAGATGCA

GTCAAAAGATTCAGGACTAATTGCATCAAGAACACAGAGAAAGACATATTTCTCAAGATCAGAAGTACTA

TTCCAGTATGGACGATTCAAGGCTTGCTTCATAAACCAAGGCAAGTAATAGAGATTGGAGTCTCTAAAAA

GGTAGTTCCTACTGAATCTAAGGCCATGCATGGAGTCTAAGATTCAAATCGAGGATCTAACAGAACTCGC

CGTGAAGACTGGCGAACAGTTCATACAGAGTCTTTTACGACTCAATGACAAGAAGAAAATCTTCGTCAAC

ATGGTGGAGCACGACACTCTGGTCTACTCCAAAAATGTCAAAGATACAGTCTCAGAAGACCAAAGGGCTA

TTGAGACTTTTCAACAAAGGATAATTTCGGGAAACCTCCTCGGATTCCATTGCCCAGCTATCTGTCACTT

CATCGAAAGGACAGTAGAAAAGGAAGGTGGCTCCTACAAATGCCATCATTGCGATAAAGGAAAGGCTATC

ATTCAAGATCTCTCTGCCGACAGTGGTCCCAAAGATGGACCCCCACCCACGAGGAGCATCGTGGAAAAAG

AAGACGTTCCAACCACGTCTTCAAAGCAAGTGGATTGATGTGACATCTCCACTGACGTAAGGGATGACGC

ACAATCCCACTATCCTTCGCAAGACCCTTCCTCTATATAAGGAAGTTCATTTCATTTGGAGAGGACACGC

TCGAGGCTAGCATGGATCTCGGGCCCCAAATAATGATTTTATTTTGACTGATAGTGACCTGTTCGTTGCA

ACAAATTGATGAGCAATGCTTTTTTATAATGCCAACTTTGTACAAAAAAGCTGAACGAGAAACGTAAAAT

GATATAAATATCAATATATTAAATTAGATTTTGCATAAAAAACAGACTACATAATACTGTAAAACACAAC

ATATCCAGTCACTATGAATCAACTACTTAGATGGTATTAGTGACCTGTAGTCGACCGACAGCCTTCCAAA

TGTTCTTCGGGTGATGCTGCCAACTTAGTCGACCGACAGCCTTCCAAATGTTCTTCTCAAACGGAATCGT

CGTATCCAGCCTACTCGCTATTGTCCTCAATGCCGTATTAAATCATAAAAAGAAATAAGAAAAAGAGGTG

CGAGCCTCTTTTTTGTGTGACAAAATAAAAACATCTACCTATTCATATACGCTAGTGTCATAGTCCTGAA

AATCATCTGCATCAAGAACAATTTCACAACTCTTATACTTTTCTCTTACAAGTCGTTCGGCTTCATCTGG

ATTTTCAGCCTCTATACTTACTAAACGTGATAAAGTTTCTGTAATTTCTACTGTATCGACCTGCAGACTG

GCTGTGTATAAGGGAGCCTGACATTTATATTCCCCAGAACATCAGGTTAATGGCGTTTTTGATGTCATTT

TCGCGGTGGCTGAGATCAGCCACTTCTTCCCCGATAACGGAGACCGGCACACTGGCCATATCGGTGGTCA

TCATGCGCCAGCTTTCATCCCCGATATGCACCACCGGGTAAAGTTCACGGGAGACTTTATCTGACAGCAG

ACGTGCACTGGCCAGGGGGATCACCATCCGTCGCCCGGGCGTGTCAATAATATCACTCTGTACATCCACA

AACAGACGATAACGGCTCTCTCTTTTATAGGTGTAAACCTTAAACTGCATTTCACCAGTCCCTGTTCTCG

TCAGCAAAAGAGCCGTTCATTTCAATAAACCGGGCGACCTCAGCCATCCCTTCCTGATTTTCCGCTTTCC

AGCGTTCGGCACGCAGACGACGGGCTTCATTCTGCATGGTTGTGCTTACCAGACCGGAGATATTGACATC

ATATATGCCTTGAGCAACTGATAGCTGTCGCTGTCAACTGTCACTGTAATACGCTGCTTCATAGCACACC

TCTTTTTGACATACTTCGGGTAGTGCCGATCAACGTCTCATTTTCGCCAAAAGTTGGCCCAGGGCTTCCC

GGTATCAACAGGGACACCAGGATTTATTTATTCTGCGAAGTGATCTTCCGTCACAGGTATTTATTCGGCG

CAAAGTGCGTCGGGTGATGCTGCCAACTTAGTCGACTACAGGTCACTAATACCATCTAAGTAGTTGATTC

ATAGTGACTGGATATGTTGTGTTTTACAGTATTATGTAGTCTGTTTTTTATGCAAAATCTAATTTAATAT

ATTGATATTTATATCATTTTACGTTTCTCGTTCAGCTTTCTTGTACAAAGTTGGCATTATAAGAAAGCAT

TGCTTATCAATTTGTTGCAACGAACAGGTCACTATCAGTCAAAATAAAATCATTATTTGCCATCCAGCTG

CAGCTCCTCGAGGAATTCGGTACCCCAATTGGTAAGGAAATAATTATTTTCTTTTTTCCTTTTAGTATAA

AATAGTTAAGTGATGTTAATTAGTATGATTATAATAATATAGTTGTTATAATTGTGAAAAAATAATTTAT

AAATATATTGTTTACATAAACAACATAGTAATGTAAAAAAATATGACAAGTGATGTGTAAGACGAAGAAG

ATAAAAGTTGAGAGTAAGTATATTATTTTTAATGAATTTGATCGAACATGTAAGATGATATACGGCCGGT

AAGAGGTTCCAACTTTCACCATAATGAAATAAGATCACTACCGGGCGTATTTTTTGAGTTATCGAGATTT

TCAGGAGCTAAGGAAGCTAAAATGGAGAAAAAAATCACTGGATATACCACCGTTGATATATCCCAATGGC

ATCGTAAAGAACATTTTGAGGCATTTCAGTCAGTTGCTCAATGTACCTATAACCAGACCGTTCAGCTGGA

TATTACGGCCTTTTTAAAGACCGTAAAGAAAAATAAGCACAAGTTTTATCCGGCCTTTATTCACATTCTT

GCCCGCCTGATGAATGCTCATCCGGAATTCCGTATGGCAATGAAAGACGGTGAGCTGGTGATATGGGATA

GTGTTCACCCTTGTTACACCGTTTTCCATGAGCAAACTGAAACGTTTTCATCGCTCTGGAGTGAATACCA

CGACGATTTCCGGCAGTTTCTACACATATATTCGCAAGATGTGGCGTGTTACGGTGAAAACCTGGCCTAT

TTCCCTAAAGGGTTTATTGAGAATATGTTTTTCGTCTCAGCCAATCCCTGGGTGAGTTTCACCAGTTTTG

ATTTAAACGTGGCCAATATGGACAACTTCTTCGCCCCCGTTTTCACCATGGGCAAATATTATACGCAAGG

CGACAAGGTGCTGATGCCGCTGGCGATTCAGGTTCATCATGCCGTCTGTGATGGCTTCCATGTCGGCAGA

ATGCTTAATGAATTACAACAGTACTGCGATGAGTGGCAGGGCGGGGCGTAATCGCGTGGATCCGGCTTAC

TAAAAGCCAGATAACAGTATGCGTATTTGCGCGCTGATTTTTGCGGTATAAGAATATATACTGATATGTC

GGGCCCATAATAGTAATTCTAGCTGGTTTGATGAATTAAATATCAATGATAAAATACTATAGTAAAAATA

AGAATAAATAAATTAAAATAATATTTTTTTATGATTAATAGTTTATTATATAATTAAATATCTATACCAT

TACTAAATATTTTAGTTTAAAAGTTAATAAATATTTTGTTAGAAATTCCAATCTGCTTGTAATTTATCAA

TAAACAAAATATTAAATAACAAGCTAAAGTAACAAATAATATCAAACTAATAGAAACAGTAATCTAATGT

AACAAAACATAATCTAATGCTAATATAACAAAGCGCAAGATCTATCATTTTATATAGTATTATTTTCAAT

CAACATTCTTATTAATTTCTAAATAATACTTGTAGTTTTATTAACTTCTAAATGGATTGACTATTAATTA

AATGAATTAGTCGAACATGAATAAACAAGGTAACATGATAGATCATGTCATTGTGTTATCATTGATCTTA

CATTTGGATTGATTACAGTTGGGAAATTGGGTTCGAAATCGATAAGCTTGGATCCTCTAGAGAGCTGCAG

CTGGATGGCAAATAATGATTTTATTTTGACTGATAGTGACCTGTTCGTTGCAACAAATTGATAAGCAATG

CTTTCTTATAATGCCAACTTTGTACAAGAAAGCTGAACGAGAAACGTAAAATGATATAAATATCAATATA

TTAAATTAGATTTTGCATAAAAAACAGACTACATAATACTGTAAAACACAACATATCCAGTCACTATGAA

TCAACTACTTAGATGGTATTAGTGACCTGTAGTCGACTAAGTTGGCAGCATCACCCGACGCACTTTGCGC

CGAATAAATACCTGTGACGGAAGATCACTTCGCAGAATAAATAAATCCTGGTGTCCCTGTTGATACCGGG

AAGCCCTGGGCCAACTTTTGGCGAAAATGAGACGTTGATCGGCACTACCCATTTCACAACTCTTATACTT

TTCTCTTACAAGTCGTTCGGCTTCATCTGGATTTTCAGCCTCTATACTTACTAAACGTGATAAAGTTTCT

GTAATTTCTACTGTATCGACCTGCAGACTGGCTGTGTATAAGGGAGCCTGACATTTATATTCCCCAGAAC

ATCAGGTTAATGGCGTTTTTGATGTCATTTTCGCGGTGGCTGAGATCAGCCACTTCTTCCCCGATAACGG

AGACCGGCACACTGGCCATATCGGTGGTCATCATGCGCCAGCTTTCATCCCCGATATGCACCACCGGGTA

AAGTTCACGGGAGACTTTATCTGACAGCAGACGTGCACTGGCCAGGGGGATCACCATCCGTCGCCCGGGC

GTGTCAATAATATCACTCTGTACATCCACAAACAGACGATAACGGCTCTCTCTTTTATAGGTGTAAACCT

TAAACTGCATTTCACCAGTCCCTGTTCTCGTCAGCAAAAGAGCCGTTCATTTCAATAAACCGGGCGACCT

CAGCCATCCCTTCCTGATTTTCCGCTTTCCAGCGTTCGGCACGCAGACGACGGGCTTCATTCTGCATGGT

TGTGCTTACCAGACCGGAGATATTGACATCATATATGCCTTGAGCAACTGATAGCTGTCGCTGTCAACTG

TCACTGTAATACGCTGCTTCATAGCACACCTCTTTTTGACATACTTCTGTTCTTGATGCAGATGATTTTC

AGGACTATGACACTAGCGTATATGAATAGGTAGATGTTTTTATTTTGTCACACAAAAAAGAGGCTCGCAC

CTCTTTTTCTTATTTCTTTTTATGATTTAATACGGCATTGAGGACAATAGCGAGTAGGCTGGATACGACG

ATTCCGTTTGAGAAGAACATTTGGAAGGCTGTCGGTCGACTAAGTTGGCAGCATCACCCGAAGAACATTT

GGAAGGCTGTCGGTCGACTACAGGTCACTAATACCATCTAAGTAGTTGATTCATAGTGACTGGATATGTT

GTGTTTTACAGTATTATGTAGTCTGTTTTTTATGCAAAATCTAATTTAATATATTGATATTTATATCATT

TTACGTTTCTCGTTCAGCTTTTTTGTACAAAGTTGGCATTATAAAAAAGCATTGCTCATCAATTTGTTGC

AACGAACAGGTCACTATCAGTCAAAATAAAATCATTATTTGGGGCCCGAGATCCATGCTAGCTCTAGAGT

CCTGCTTTAATGAGATATGCGAGACGCCTATGATCGCATGATATTTGCTTTCAATTCTGTTGTGCACGTT

GTAAAAAACCTGAGCATGTGTAGCTCAGATCCTTACCGCCGGTTTCGGTTCATTCTAATGAATATATCAC

CCGTTACTATCGTATTTTTATGAATAATATTCTCCGTTCAATTTACTGATTGTACCCTACTACTTATATG

TACAATATTAAAATGAAAACAATATATTGTGCTGAATAGGTTTATAGCGACATCTATGATAGAGCGCCAC

AATAACAAACAATTGCGTTTTATTATTACAAATCCAATTTTAAAAAAAGCGGCAGAACCGGTCAAACCTA

AAAGACTGATTACATAAATCTTATTCAAATTTCAAAAGGCCCCAGGGGCTAGTATCTACGACACACCGAG

CGGCGAACTAATAACGTTCACTGAAGGGAACTCCGGTTCCCCGCCGGCGCGCATGGGTGAGATTCCTTGA

AGTTGAGTATTGGCCGTCCGCTCTACCGAAAGTTACGGGCACCATTCAACCCGGTCCAGCACGGCGGCCG

GGTAACCGACTTGCTGCCCCGAGAATTATGCAGCATTTTTTTGGTGTATGTGGGCCCCAAATGAAGTGCA

GGTCAAACCTTGACAGTGACGACAAATCGTTGGGCGGGTCCAGGGCGAATTTTGCGACAACATGTCGAGG

CTCAGCAGGACCTGCAGGCATGCAAGCTAGCTTACTAGTGATGCATATTCTATAGTGTCACCTAAATCTG

C

>gi|16903089|gb|AF433042.1|AF433042 Cloning vector pWS31, complete sequence

GAATTCGAGCTCGGTACCCCGCGCGCGGAGGGGGAGGCTGTACGTGGGGCGCGTTGCGTGACCCAGGGAT

GAAAGTAGGATGGGAAAATCCCGTACCGACCGTTATCGTATAACCGATTTTGTTAGTTTTATCCCGATCG

ATTTCGAACCCGAGGTAAAAAACGAAAACGGAACGGAAACGGGATATACAAAACGGTAAACGGAAACGGA

AACGGTAGAGCTAGTTTCCCGACCGTTTCACCGGGATCCCGTTTTTAATCGGGATGATCCCGTTTCGTTA

CCGTATTTTCTAATTCGGGATGACTGCAATATGGCCAGCTCCAACTCCCATCCATAACCACTGAGGCCCA

GCCCATGTAAGAAATACCTAGCGAACGCTGCTCTGCCTCTCTCCCAGGCGGCCAGGCACCACACGAGTAA

CAGCATCACACATTCACACGCCGCCACGCGCCCACGCCGGAGTCCGGACGCCGCCAGCCGCACGCCGACG

CCGGCGACGCGTCTCGCTCTCGCCTGCTCTCTCCGACTCTCCCTGTCTCCCAGCCGGCCGGCCGCTGGGC

TGCACCAGGCACCACACGCGGTGACGGCCGTGACGCGGCACGCCGGACGCAGACGCCGCCATCCACGGTC

CGCCCTCCACTCCACTGCTCGCGACTCGCCCATCCGCGCCGCGGTCCGCCCATCCGCCCAGACCTCCACT

CCACTGCTCGCCCATCCGCGGTCCGCCCATCCGCCATCCGCCATCTGCGGTCAGCGGTCCTCCAGACCTC

CACTGCTCGGCGCTCGCCCATCCGGCCATCCGCGGTCTCCCTGTCTCCACGGCTGCTCACAGGCTCACAG

CACTTAGCAGTACAGCACGTCAGCACCATTGCACCAAGCTGTTGTGTCATTTGTGTGCTGTCCAGGGGCT

CTGCAACACCTGCTGATTGCTGTCCAGCCGTCCAGGTGCTCACAAGTCACAGCAGTACAGCACCAAGCTG

ATTGCTGAACACCTGCTGTCCAGGGCTCTGCTCTCCACTTCGGCTAGCCGGCTACGACTCCATTCCTCAG

ATGACGCCTCCGGTTGGAAATAATCCTCCCTCAGGCTCAGCCATAAGATTGGCCAAGTTGATGTCTACCA

CAAGAGCGCCTTCTACTCGCAAAACAAATTCCGTATTCTCTGCATATGCTCAAGGTATATATTAGAAAAA

CAGTAGCAATAGCATTAGCATTACTAATTGGTTGTAGATTGGGAAGCATCATATTGACTGTAGAATAATA

CGAAAAATCTGTTTATAACAGGGTTGAAAAGAAAAGCTGAAGCCTCTTCTAGTCGGATTCAGAATGTACG

TGCACGTGCGCGTGGGCATGGATGTGGCCGCACATCACCATCATCATCAACAGCTGAGGCCGAGAGGCAT

TTTATTCAGAGTGTAAGCAGTAGTAATGCAAATGGTACAGCTACAGATCCGAGTCAAGATGATATGGCTA

TTGTTCATGAACCACAACCACAACCACAACCACAACCAGAACCACAACCACAGCCACAACCTGAACCCGA

AGAAGAAGCACCACAGAAGAGGGCAAAGAAGTGCACATCGGATGTATGGCAGCATTTCACCAAGAAGGAA

ATTGAAGTGGAGGTCGATGGAAAGAAATACGTTCAGGTATGGGGACATTGCAACTTTCCTAATTGCAAGG

CTAAGTATAGGGCTGAGGGTCATCATGGAACAAGCGGATTTCGAAATCACTTGAGAACATCACATAGTTT

AGTTAAAGGTCAGTTGTGTCTAAAAAGTGAAAAGGATCATGGCAAAGACATAAATCTCATTGAGCCTTAT

AAGTACGATGAAGTGGTTAGCCTAAAGAAGCTAGATCCCCCGGGCTGCAGGAATTCGATATCAAGCTTAT

CGATTTGGTGTATCGAGATTGGTTATGAAATTCAGATGCTAGTGTAATGTATTGGTAATTTGGGAAGATA

TAATAGGAAGCAAGGCTATTTATCCATTTCTGAAAAGGCGAAATGGCGTCACCGCGAGCGTCACGCGCAT

TCCGTTCTTGCTGTAAAGCGTTGTTTGGTACACTTTTGACTAGCGAGGCTTGGCGTGTCAGCGTATCTAT

TCAAAAGTCGTTAATGGCTGCGGATCAAGAAAAAGTTGGAATAGAAACAGAATACCCGCGAAATTCAGGC

CCGGTTGCCATGTCCTACACGCCGAAATAAACGACCAAATTAGTAGAAAAATAAAAACTGACTCGGATAC

TTACGTCACGTCTTGCGCACTGATTTGAAAAATCTCAATATAAACAAAGACGGCCACAAGAAAAAACCAA

AACACCGATATTCATTAATCTTATCTAGTTTCTCAAAAAAATTCATATCTTCCACACGTGGATCTATGAT

TGAACAAGATGGATTGCACGCAGGTTCTCCGGCCGCTTGGGTGGAGAGGCTATTCGGCTATGACTGGGCA

CAACAGACAATCGGCTGCTCTGATGCCGCCGTGTTCCGGCTGTCAGCGCAGGGGCGCCCGGTTCTTTTTG

TCAAGACCGACCTGTCCGGTGCCCTGAATGAACTGCAGGACGAGGCAGCGCGGCTATCGTGGCTGGCCAC

GACGGGCGTTCCTTGCGCAGCTGTGCTCGACGTTGTCACTGAAGCGGGAAGGGACTGGCTGCTATTGGGC

GAAGTGCCGGGGCAGGATCTCCTGTCATCTCACCTTGCTCCTGCCGAGAAAGTATCCATCAGGCTGAATG

CAATGCGGCGGCTGCATACGCTTGATCCGGCTACCTGCCCATTCGACCACCAAGCGAAACATCGCATCGA

GCGAGCACGTACTCGGATGGAAGCCGGTCTTGTCGATCAGGATGATCTGGACGAAGAGCATCAGGGGCTC

GCGCCAGCCGAACTGTTCGCCAGGCTCAAGGCGCGCATGCCCGACGGCGAGGATCTCGTCGTGACCCATG

GCGATGCCTGCTTGCCGAATATCATGGTGGAAAATGGCCGCTTTTCTGGATTCATCGACTGTGGCCGGCT

GGGTGTGGCGGACCGCTATCAGGACATAGCGTTGGCTACCCGTGATATTGCTGAAGAGCTTGGCGGCGAA

TGGGCTGACCGCTTCCTCGTGCTTTACGGTATCGCCGCTCCCGATTCGCAGCGCATCGCCTTCTATCGCC

TTCTTGACGAGTTCTTCTGAGCGGGACTCTGGGGTTCGAAATGACCGACCAAGCGACGCCCAACCTGCCA

TCACGAGATTTCGATTCCACCGCCGCCTTCTATGAAAGGTTGGGCTTCGGAATCGTTTTCCGGGACGCCG

GCTGGATGATCCTCCAGCGCGGGGATCTCATGCTGGAGTTCTTCGCCCACCCCCTGCTTTAATGAGATAT

GCGAGACGCCTATGATCGCATGATATTTGCTTTCAATTCTGTTGTGCACGTTGTAAAAAACCTGAGCATG

TGTAGCTCAGATCCTTACCGCCGGTTTCGGTTCATTCTAATGAATATATCACCCGTTACTATCGTATTTT

TATGAATAATATTCTCCGTTCAATTTACTGATTGTACCCTACTACTTATATGTACAATATTAAAATGAAA

ACAATATATTGTGCTGAATAGGTTTATAGCGACATCTATGATAGAGCGCCACAATAACAAACAATTGCGT

TTTATTATTACAAATCCAATTTTAAAAAAAGCGGCAGAACCGGTCAAACCTAAAAGACTGATTACATAAA

TCTTATTCAAATTTCAAAAGTGCCCCAGGGGCTAGTATCTACGACACACCGAGCGGCGAACTAATAACGC

TCACTGAAGGGAACTCCGGTTCCCCGCCGGCGCGCATGGGTGAGATTCCTTGAAGTTGAGTATTGGCCGT

CCGCTCTACCGAAAGTTACGGGCACCATTCAACCCGGTCCAGCACGGCGGCCGGGTAACCGACTTGCTGC

CCCGAGAATTATGCAGCATTTTTTTGGTGTATGTGGGCCCCAAATGAAGTGCAGGTCAAACCTTGACAGT

GACGACAAATCGTTGGGCGGGTCCAGGGCGAATTTTGCGACAACATGTCGAGGCTCAGCAGGGGCTCGAT

CCCCTCGCGAGTTGGTTCAGCTGCTGCCTGAGGCTGGACGACCTCGCGGAGTTCTACCGGCAGTGCAAAT

CCGTCGGCATCCAGGAAACCAGCAGCGGCTATCCGCGCATCCATGCCCCCGAACTGCAGGAGTGGGGAGG

CACGATGGCCGCTTTGGTCGAGCTTCTAGAGATCTAGTAACATAGATGACACCGCGCGCGATAATTTATC

CTAGTTTGCGCGCTATATTTTGTTTTCTATCGCGTATTAAATGTATAATTGCGGGACTCTAATCATAAAA

ACCCATCTCATAAATAACGTCATGCATTACATGTTAATTATTACATGCTTAACGTAATTCAACAGAAATT

ATATGATAATCATCGCAAGACCGGCAACAGGATTCAATCTTAAGAAACTTTATTGCCAAATGTTTGAACG

ATCTGCAGGTCGACGGATCTGAATTCCCGAGGCTGTAGCCGACGATGGTGCGCCAGGAGAGTTGTTGATT

CATTGTTTGCCTCCCTGCTGCGGTTTTTCACCGAAGTTCATGCCAGTCCAGCGTTTTTGCAGCAGAAAAG

CCGCCGACTTCGGTTTGCGGTCGCGAGTGAAGATCCCTTTCTTGTTACCGCCAACGCGCAATATGCCTTG

CGAGGTCGCAAAATCGGCGAAATTCCATACCTGTTCACCGACGACGGCGCTGACGCGATCAAAGACGCGG

TGATACATATCCAGCCATGCACACTGATACTCTTCACTCCACATGTCGGTGTACATTGAGTGCAGCCCGG

CTAACGTATCCACGCCGTATTCGGTGATGATAATCGGCTGATGCAGTTTCTCCTGCCAGGCCAGAAGTTC

TTTTTCCAGTACCTTCTCTGCCGTTTCCAAATCGCCGCTTTGGACATACCATCCGTAATAACGGTTCAGG

CACAGCACATCAAAGAGATCGCTGATGGTATCGGTGTGAGCGTCGCAGAACATTACATTGACGCAGGTGA

TCGGACGCGTCGGGTCGAGTTTACGCGTTGCTTCCGCCAGTGGCGCGAAATATTCCCGTGCACCTTGCGG

ACGGGTATCCGGTTCGTTGGCAATACTCCACATCACCACGCTTGGGTGGTTTTTGTCACGCGCTATCAGC

TCTTTAATCGCCTGTAAGTGCGCTTGCTGAGTTTCCCCGTTGACTGCCTCTTCGCTGTACAGTTCTTTCG

GCTTGTTGCCCGCTTCGAAACCAATGCCTAAAGAGAGGTTAAAGCCGACAGCAGCAGTTTCATCAATCAC

CACGATGCCATGTTCATCTGCCCAGTCGAGCATCTCTTCAGCGTAAGGGTAATGCGAGGTACGGTAGGAG

TTGGCCCCAATCCAGTCCATTAATGCGTGGTCGTGCACCATCAGCACGTTATCGAATCCTTTGCCACGCA

AGTCCGCATCTTCATGACGACCAAAGCCAGTAAAGTAGAACGGTTTGTGGTTAATCAGGAACTGTTCGCC

CTTCACTGCCACTGACCGGATGCCGACGCGAAGCGGGTAGATATCACACTCTGTCTGGCTTTTGGCTGTG

ACGCACAGTTCATAGAGATAACCTTCACCCGGTTGCCAGAGGTGCGGATTCACCACTTGCAAAGTCCCGC

TAGTGCCTTGTCCAGTTGCAACCACCTGTTGATCCGCATCACGCAGTTCAACGCTGACATCACCATTGGC

CACCACCTGCCAGTCAACAGACGCGTGGTTACAGTCTTGCGCGACATGCGTCACCACGGTGATATCGTCC

ACCCAGGTGTTCGGCGTGGTGTAGAGCATTACGCTGCGATGGATTCCGGCATAGTTAAAGAAATCATGGA

AGTAAGACTGCTTTTTCTTGCCGTTTTCGTCGGTAATCACCATTCCCGGCGGGATAGTCTGCCAGTTCAG

TTCGTTGTTCACACAAACGGTGATACGTACACTTTTCCCGGCAATAACATACGGCGTGACATCGGCTTCA

AATGGCGTATAGCCGCCCTGATGCTCCATCACTTCCTGATTATTGACCCACACTTTGCCGTAATGAGTGA

CCGCATCGAAACGCAGCACGATACGCTGGCCTGCCCAACCTTTCGGTATAAAGACTTCGCGCTGATACCA

GACGTTGCCCGCATAATTACGAATATCTGCATCGGCGAACTGATCGTTAAAACTGCCTGGCACAGCAATT

GCCCGGCTTTCTTGTAACGCGCTTTCCCACCAACGCTGATCAATTCCACAGTTTTCGCGATCCAGACTGA

ATGCCCACAGGCCGTCGAGTTTTTTGATTTCACGGGTTGGGGTTTCTACAGGACGGACCATGGTCGACAG

ATCCGGTTCTCTCCAAATGAAATGAACTTCCTTATATAGAGGAGGGTCTTGCGGATCTGAATATATGTTT

TCATGTGTGATTTTACCGAACAAAAATACCGGTTCCCGTCCGATTTCGACTTTAACCCGACCGGATCGTA

TCGGTTTTCGATTACCGTATTTATCCCGTTCGTTTTCGTTACCGGTATATCCCGTTTTCGTTTCCGTCCC

GCAAGTTAAATATGAAAATGAAAACGGTAGAGGTATTTTACCGACCGTTACCGACCGTTTTCATCCCTAG

AGCTCGGATCCTCTAGACCGAACAAAAATACCGGTTCCCGTCCGATTTCGACTTTAACCCGACCGGATCG

TATCGGTTTTCGATTACCGTATTTATCCCGTTCGTTTTCGTTACCGGTATATCCCGTTTTCGTTTCCGTC

CCGCAAGTTAAATATGAAAATGAAAACGGTAGAGGTATTTTACCGACCGTTACCGACCGTTTTCATCCCT

AGAGCTCGGATCCTC

>gi|16903540|gb|AF404854.1|AF404854 Binary vector pJawohl3-RNAi, complete sequence

CTCGAGAAAGAGGATCCACCTGAGGCCTCCGTTCCATGGGCTAGAAGCTTCTCCTCCTCTGCTAACGTAA

GCCTCTCTGTTTTTTTTCTCTGTTTCTTTTGAAATGAATCCAATTAGTGATGATAATCTGTGTTTGATGT

ATCATTGATTTAACATCTTGACAATGAATCGTGATCGGAAGTGATAAAGTTATGGGTCAACGGTTTCAAA

GAGAGAGAAAGACTTTTAGAGTCAACTCTCGACTCTTTCTTAATTATGTTATTGCTATTTGTCTCTTTTC

TTGAAGTCTGAACAATTCTTGGGATTGTTTTGCAGGTTCTAGCTTCTCCAACCACAGGAATTCATCGCCC

GGGACTGGGTTAACTTCCGATCGATTCTAGACTAGTTCTAGAGTCCGCAAAAATCACCAGTCTCTCTCTA

CAAATCTATCTCTCTCTATTTTTCTCCAGAATAATGTGTGAGTAGTTCCCAGATAAGGGAATTAGGGTTC

TTATAGGGTTTCGCTCATGTGTTGAGCATATAAGAAACCCTTAGTATGTATTTGTATTTGTAAAATACTT

CTATCAATAAAATTTCTAATTCCTAAAACCAAAATCCAGTGACCGGGCGGCCGCCACCGCGGTGGAGGGG

GATCAGATTGTCGTTTCCCGCCTTCAGTTTAAACTATCAGTGTTTGACAGGATATATTGGCGGGTAAACC

TAAGAGAAAAGAGCGTTTATTAGAATAATCGGATATTTAAAAGGGCGTGAAAAGGTTTATCCGTTCGTCC

ATTTGTATGTGTACATCACCGACGAGCAAGGCAAGACCGAGCGCCTTTCCGACGCTCACCGGGCTGGTTG

CCCTCGCCGCTGGGCTGGCGGCCGTCTATGGCCCTGCAAACGCGCCAGAAACGCCGTCGAAGCCGTGTGC

GAGACACCGGCCGCCGGCGTTGTGGATACCTCGCGGAAAACTTGGCCCTCACTGACAGATGAGGGGCGGA

CGTTGACACTTGAGGGGCCGACTCACCCGGCGCGGCGTTGACAGATGAGGGGCAGGCTCGATTTCGGCCG

GCGACGTGGAGCTGGCCAGCCTCGCAAATCGGCGAAAACGCCTGATTTTACGCGAGTTTCCCACAGATGA

TGTGGACAAGCCTGGGGATAAGTGCCCTGCGGTATTGACACTTGAGGGGCGCGACTACTGACAGATGAGG

GGCGCGATCCTTGACACTTGAGGGGCAGAGTGCTGACAGATGAGGGGCGCACCTATTGACATTTGAGGGG

CTGTCCACAGGCAGAAAATCCAGCATTTGCAAGGGTTTCCGCCCGTTTTTCGGCCACCGCTAACCTGTCT

TTTAACCTGCTTTTAAACCAATATTTATAAACCTTGTTTTTAACCAGGGCTGCGCCCTGTGCGCGTGACC

GCGCACGCCGAAGGGGGGTGCCCCCCCTTCTCGAACCCTCCCGGCCCGCTAACGCGGGCCTCCCATCCCC

CCAGGGGCTGCGCCCCTCGGCCGCGAACGGCCTCACCCCAAAAATGGCAGCGCTGGCAGTCCTTGCCATT

GCCGGGATCGGGGCAGTAACGGGATGGGCGATCAGCCCGACAAGCTACCCCTATTTGTTTATTTTTCTAA

ATACATTCAAATATGTATCCGCTCATGAGACAATAACCCTGATAAATGCTTCAATAATATTGAAAAAGGA

AGAGTATGAGTATTCAACATTTCCGTGTCGCCCTTATTCCCTTTTTTGCGGCATTTTGCCTTCCTGTTTT

TGCTCACCCAGAAACGCTGGTGAAAGTAAAAGATGCTGAAGATCAGTTGGGTGCACGAGTGGGTTACATC

GAACTGGATCTCAACAGCGGTAAGATCCTTGAGAGTTTTCGCCCCGAAGAACGTTTTCCAATGATGAGCA

CTTTTAAAGTTCTGCTATGTGGCGCGGTATTATCCCGTATTGACGCCGGGCAAGAGCAACTCGGTCGCCG

CATACACTATTCTCAGAATGACTTGGTTGAGTACTCACCAGTCACAGAAAAGCATCTTACGGATGGCATG

ACAGTAAGAGAATTATGCAGTGCTGCCATAACCATGAGTGATAACACTGCGGCCAACTTACTTCTGACAA

CGATCGGAGGACCGAAGGAGCTAACCGCTTTTTTGCACAACATGGGGGATCATGTAACTCGCCTTGATCG

TTGGGAACCGGAGCTGAATGAAGCCATACCAAACGACGAGCGTGACACCACGATGCCTGTAGCAATGGCA

ACAACGTTGCGCAAACTATTAACTGGCGAACTACTTACTCTAGCTTCCCGGCAACAATTAATAGACTGGA

TGGAGGCGGATAAAGTTGCAGGACCACTTCTGCGCTCGGCCCTTCCGGCTGGCTGGTTTATTGCTGATAA

ATCTGGAGCCGGTGAGCGTGGGTCTCGCGGTATCATTGCAGCACTGGGGCCAGATGGTAAGCCCTCCCGT

ATCGTAGTTATCTACACGACGGGGAGTCAGGCAACTATGGATGAACGAAATAGACAGATCGCTGAGATAG

GTGCCTCACTGATTAAGCATTGGTAACTGTCAGACCAAGTTTACTCATATATACTTTAGATTGATTTAAA

ACTTCATTTTTAATTTAAAAGGATCTAGGTGAAGATCCTTTTTGATAATCTCATGACCAAAATCCCTTAA

CGTGAGTTTTCGTTCCACTGAGCGTCAGACCCCGTAGAAAAGATCAAAGGATCTTCTTGAGATCCTTTTT

TTCTGCGCGTAATCTGCTGCTTGCAAACAAAAAAACCACCGCTACCAGCGGTGGTTTGTTTGCCGGATCA

AGAGCTACCAACTCTTTTTCCGAAGGTAACTGGCTTCAGCAGAGCGCAGATACCAAATACTGTCCTTCTA

GTGTAGCCGTAGTTAGGCCACCACTTCAAGAACTCTGTAGCACCGCCTACATACCTCGCTCTGCTAATCC

TGTTACCAGTGGCTGCTGCCAGTGGCGATAAGTCGTGTCTTACCGGGTTGGACTCAAGACGATAGTTACC

GGATAAGGCGCAGCGGTCGGGCTGAACGGGGGGTTCGTGCACACAGCCCAGCTTGGAGCGAACGACCTAC

ACCGAACTGAGATACCTACAGCGTGAGCTATGAGAAAGCGCCACGCTTCCCGAAGGGAGAAAGGCGGACA

GGTATCCGGTAAGCGGCAGGGTCGGAACAGGAGAGCGCACGAGGGAGCTTCCAGGGGGAAACGCCTGGTA

TCTTTATAGTCCTGTCGGGTTTCGCCACCTCTGACTTGAGCGTCGATTTTTGTGATGCTCGTCAGGGGGG

CGGAGCCTATGGAAAAACGCCAGCAACGCGGCCTTTTTACGGTTCCTGGCCTTTTGCTGGCCTTTTGCTC

ACATGGACTCTAGCTAGAGGATCACAGGCAGCAACGCTCTGTCATCGTTACAATCAACATGCTACCCTCC

GCGAGATCATCCGTGTTTCAAACCCGGCAGCTTAGTTGCCGTTCTTCCGAATAGCATCGGTAACATGAGC

AAAGTCTGCCGCCTTACAACGGCTCTCCCGCTGACGCCGTCCCGGACTGATGGGCTGCCTGTATCGAGTG

GTGATTTTGTGCCGAGCTGCCGGTCGGGGAGCTGTTGGCTGGCTGGTGGCAGGATATATTGTGGTGTAAA

CAAATTGACGCTTAGACAACTTAATAACACATTGCGGACGTTTTTAATGATCGAATACTAACGTCTCTAC

CAGATATCAGCTTGCATGCCGGTCGATCTAGTAACATAGATGACACCGCGCGCGATAATTTATCCTAGTT

TGCGCGCTATATTTTGTTTTCTATCGCGTATTAAATGTATAATTGCGGGACTCTAATCATAAAAACCCAT

CTCATAAATAACGTCATGCATTACATGTTAATTATTACATGCTTAACGTAATTCAACAGAAATTATATGA

TAATCATCGCAAGACCGGCAACAGGATTCAATCTTAAGAAACTTTATTGCCAAATGTTTGAACGATCTGC

TTGACTCTAGGGGTCATCAGATTTCGGTGACGGGCAGGACCGGACGGGGCGGCACCGGCAGGCTGAAGTC

CAGCTGCCAGAAACCCACGTCATGCCAGTTCCCGTGCTTGAAGCCGGCCGCCCGCAGCATGCCGCGGGGG

GCATATCCGAGCGCCTCGTGCATGCGCACGCTCGGGTCGTTGGGCAGCCCGATGACAGCGACCACGCTCT

TGAAGCCCTGTGCCTCCAGGGACTTCAGCAGGTGGGTGTAGAGCGTGGAGCCCAGTCCCGTCCGCTGGTG

GCGGGGGGAGACGTACACGGTCGACTCGGCCGTCCAGTCGTAGGCGTTGCGTGCCTTCCAGGGGCCCGCG

TAGGCGATGCCGGCGACCTCGCCGTCCACCTCGGCGACGAGCCAGGGATAGCGCTCCCGCAGACGGACGA

GGTCGTCCGTCCACTCCTGCGGTTCCTGCGGCTCGGTACGGAAGTTGACCGTGCTTGTCTCGATGTAGTG

GTTGACGATGGTGCAGACCGCCGGCATGTCCGCCTCGGTGGCACGGCGGATGTCGGCCGGGCGTCGTTCT

GGGCTCATGGTAGATCCCCCTCGATCGAGTTGAGAGTGAATATGAGACTCTAATTGGATACCGAGGGGAA

TTTATGGAACGTCAGTGGAGCATTTTTGACAAGAAATATTTGCTAGCTGATAGTGACCTTAGGCGACTTT

TGAACGCGCAATAATGGTTTCTGACGTATGTGCTTAGCTCATTAAACTCCAGAAACCCGCGGCTCAGTGG

CTCCTTCAACGTTGCGGTTCTGTCAGTTCCAAACGTAAAACGGCTTGTCCCGCGTCATCGGCGGGGGTCA

TAACGTGACTCCCTTAATTCTCCGCTCATGATCAGATTGTCGTTTCCCGCCTTCGGTTTGGGCGCGCCCG

GTCTCAGAAGACCAGAGGGCTATTGAGACTTTTCAACAAAGGGTAATATCGGGAAACCTCCTCGGATTCC

ATTGCCCAGCTATCTGTCACTTCATCGAAAGGACAGTAGAAAAGGAAGATGGCTTCTACAAATGCCATCA

TTGCGATAAAGGAAAGGCTATCGTTCAAGAATGCCTCTACCGACAGTGGTCCCAAAGATGGACCCCCACC

CACGAGGAACATCGTGGAAAAAGAAGACGTTCCAACCACGTCTTCAAAGCAAGTGGATTGATGTGATAAC

ATGGTGGAGCACGACACTCTCGTCTACTCCAAGAATATCAAAGATACAGTCTCAGAAGACCAGAGGGCTA

TTGAGACTTTTCAACAAAGGGTAATATCGGGAAACCTCCTCGGATTCCATTGCCCAGCTATCTGTCACTT

CATCGAAAGGACAGTAGAAAAGGAAGATGGCTTCTACAAATGCCATCATTGCGATAAAGGAAAGGCTATC

GTTCAAGAATGCCTCTACCGACAGTGGTCCCAAAGATGGACCCCCACCCACGAGGAACATCGTGGAAAAA

GAAGACGTTCCAACCACGTCTTCAAAGCAAGTGGATTGATGTGATATCTCCACTGACGTAAGGGATGACG

CACAATCCCACTATCCTTCGCAAGACCCTTCCTCTATATAAGGAAGTTCATTTCATTTGGAGAGGAC

>gi|18766327|gb|AF458478.1| Binary vector pLH9000, complete sequence

TCTAGAACATGGTGGAGCACGACACTCTCGTCTACTCCAAGAATATCAAAGATACAGTCTCAGAAGACCA

GAGGGCTATTGAGACTTTTCAACAAAGGGTAATATCGGGAAACCTCCTCGGATTCCATTGCCCAGCTATC

TGTCACTTCATCGAAAGGACAGTAGAAAAGGAAGATGGCTTCTACAAATGCCATCATTGCGATAAAGGAA

AGGCTATCGTTCAAGATGCCTCTACCGACAGTGGTCCCAAAGATGGACCCCCACCCACGAGGAACATCGT

GGAAAAAGAAGACGTTCCAACCACGTCTTCAAAGCAAGTGGATTGATGTGATATCTCCACTGACGTAAGG

GATGACGCACAATCCCACTATCCTTCGCAAGACCCTTCCTCTATATAAGGAAGTTCATTTCATTTGGAGA

GGACCTCGAGTGGCCACCATGGGAGCTTGGATTGAACAAGATGGATTGCACGCAGGTTCTCCGGCCGCTT

GGGTGGAGAGGCTATTCGGCTATGACTGGGCACAACAGACAATCGGCTGCTCTGATGCCGCCGTGTTCCG

GCTGTCAGCGCAGGGGCGCCCGGTTCTTTTTGTCAAGACCGACCTGTCCGGTGCCCTGAATGAACTGCAG

GACGAGGCAGCGCGGCTATCGTGGCTGGCCACGACGGGCGTTCCTTGCGCAGCTGTGCTCGACGTTGTCA

CTGAAGCGGGAAGGGACTGGCTGCTATTGGGCGAAGTGCCGGGGCAGGATCTCCTGTCATCTCACCTTGC

TCCTGCCGAGAAAGTATCCATCATGGCTGATGCAATGCGGCGGCTGCATACGCTTGATCCGGCTACCTGC

CCATTCGACCACCAAGCGAAACATCGCATCGAGCGAGCACGTACTCGGATGGAAGCCGGTCTTGTCGATC

AGGATGATCTGGACGAAGAGCATCAGGGGCTCGCGCCAGCCGAACTGTTCGCCAGGCTCAAGGCGCGCAT

GCCCGACGGCGAGGATCTCGTCGTGACCCATGGCGATGCCTGCTTGCCGAATATCATGGTGGAAAATGGC

CGCTTTTCTGGATTCATCGACTGTGGCCGGCTGGGTGTGGCGGACCGCTATCAGGACATAGCGTTGGCTA

CCCGTGATATTGCTGAAGAGCTTGGCGGCGAATGGGCTGACCGCTTCCTCGTGCTTTACGGTATCGCCGC

TCCCGATTCGCAGCGCATCGCCTTCTATCGCCTTCTTGACGAGTTCTTCTGAGCGGGACCCGCAAAAATC

ACCAGTCTCTCTCTACAAATCTATCTCTCTCTATTTTTCTCCAGAATAATGTGTGAGTAGTTCCCAGATA

AGGGAATTAGGGTTCTTATAGGGTTTCGCTCATGTGTTGAGCATATAAGAAACCCTTAGTATGTATTTGT

ATTTGTAAAATACTTCTATCAATAAAATTTCTAATTCCTAAAACCAAAATCCAGTGACCTGCAGGCATGC

AAGCTGATCCACTAGAGGCCATGGCGGCCGCACTAGTGGATCCCCCGGGATGCATGAATTCAAGCTTATC

GATGTCGACAGGCCTTAAGGGCCAGATCTTGGGCCCGGTACCCGATCAGATTGTCGTTTCCCGCCTTCGG

TTTAAACTATCAGTGTTTGACAGGATATATTGGCGGGTAAACCTAAGAGAAAAGAGCGTTTATTAGAATA

ATCGGATATTTAAAAGGGCGTGAAAAGGTTTATCCGTTCGTCCATTTGTATGTGCATGCCAACCACAGGG

TTCCCCTCGGGAGTGCTTGGCATTCCGTGCGATAATGACTTCTGTTCAACCACCCAAACGTCGGAAAGCC

TGACGACGGAGCAGCATTCCAAAAAGATCCCTTGGCTCGTCTGGGTCGGCTAGAAGGTCGAGTGGGCTGC

TGTGGCTTGATCCCTCAACGCGGTCGCGGACGTAGCGCAGCGCCGAAAAATCCTCGATCGCAAATCCGAC

GCTGTCGAAAAGCGTGATCTGCTTGTCGCTCTTTCGGCCGACGTCCTGGCCAGTCATCACGCGCCAAAGT

TCCGTCACAGGATGATCTGGCGCGAGTTGCTGGATCTCGCCTTCAATCCGGGTCTGTGGCGGGAACTCCA

CGAAAATATCCGAACGCAGCAAGATATCGCGGTGCATCTCGGTCTTGCCTGGGCAGTCGCCGCCGACGCC

GTTGATGTGGACGCCGAAAAGGATCTAGGTGAAGATCCTTTTTGATAATCTCATGACCAAAATCCCTTAA

CGTGAGTTTTCGTTCCACTGAGCGTCAGACCCCGTAGAAAAGATCAAAGGATCTTCTTGAGATCCTTTTT

TTCTGCGCGTAATCTGCTGCTTGCAAACAAAAAAACCACCGCTACCAGCGGTGGTTTGTTTGCCGGATCA

AGAGCTACCAACTCTTTTTCCGAAGGTAACTGGCTTCAGCAGAGCGCAGATACCAAATACTGTTCTTCTA

GTGTAGCCGTAGTTAGGCCACCACTTCAAGAACTCTGTAGCACCGCCTACATACCTCGCTCTGCTAATCC

TGTTACCAGTGGCTGCTGCCAGTGGCGATAAGTCGTGTCTTACCGGGTTGGACTCAAGACGATAGTTACC

GGATAAGGCGCAGCGGTCGGGCTGAACGGGGGGTTCGTGCACACAGCCCAGCTTGGAGCGAACGACCTAC

ACCGAACTGAGATACCTACAGCGTGAGCTATGAGAAAGCGCCACGCTTCCCGAAGGGAGAAAGGCGGACA

GGTATCCGGTAAGCGGCAGGGTCGGAACAGGAGAGCGCACGAGGGAGCTTCCAGGGGGAAACGCCTGGTA

TCTTTATAGTCCTGTCGGGTTTCGCCACCTCTGACTTGAGCGTCGATTTTTGTGATGCTCGTCAGGGGGG

CGGAGCCTATGGAAAAACGCCAGCAACGCGGCCTTTTTACGGTTCCTGGCCTTTTGCTGGCCTTTTGCTC

ACATGTTCTTTCCTGCGTTATCCCCTGATTCTGTGGATAACCGATTACCGCCTTTGAGTGAGCTGATACC

GCTCGCCGCAGCCGAACGACCGAGCGCAGCGAGTCAGTGAGCGAGGAAGCGGAAGAGCGCCTGATGCGGT

ATTTTCTCCTTACGCATCTGTGCGGTATTTCACACCGCATATGGTGCACTCTCAGTACAATCTGCTCTGA

TGCCGCATAGTTAAGCCAGTATACACTCCGCTATCGCTACGTGACTGGGTCATGGCTGCGCCCCGACACC

CGCCAACACCCGCTGACGCGCCCTGACGGGCTTGTCTGCTCCCGGCATCCGCTTACAGACAAGCTGTGAC

CGTCTCCGGGAGCTGCATGTGTCAGAGGTTTTCACCGTCATCACCGAAACGCGCGAGGCAGGGGTACGTC

GAGGTCGATCCAACCCCTCCGCTGCTATAGTGCAGTCGGCTTCTGACGTTCAGTGCAGCCGTCTTCTGAA

AACGACATGTCGCACAAGTCCTAAGTTACGCGACAGGCTGCCGCCCTGCCCTTTTCCTGGCGTTTTCTTG

TCGCGTGTTTTAGTCGCATAAAGTAGAATACTTGCGACTAGAACCGGAGACATTACGCCATGAACAAGAG

CGCCGCCGCTGGCCTGCTGGGCTATGCCCGCGTCAGCACCGACGACCAGGACTTGACCAACCAACGGGCC

GAACTGCACGCGGCCGGCTGCACCAAGCTGTTTTCCGAGAAGATCACCGGCACCAGGCGCGACCGCCCGG

AGCTGGCCAGGATGCTTGACCACCTACGCCCTGGCGACGTTGTGACAGTGACCAGGCTAGACCGCCTGGC

CCGCAGCACCCGCGACCTACTGGACATTGCCGAGCGCATCCAGGAGGCCGGCGCGGGCCTGCGTAGCCTG

GCAGAGCCGTGGGCCGACACCACCACGCCGGCCGGCCGCATGGTGTTGACCGTGTTCGCCGGCATTGCCG

AGTTCGAGCGTTCCCTAATCATCGACCGCACCCGGAGCGGGCGCGAGGCCGCCAAGGCGCGAGGCGTGAA

GTTTGGCCCCCGCCCTACCCTCACCCCGGCACAGATCGCGCACGCCCGCGAGCTGATCGACCAGGAAGGC

CGCACCGTGAAAGAGGCGGCTGCACTGCTTGGCGTGCATCGCTCGACCCTGTACCGCGCACTTGAGCGCA

GCGAGGAAGTGACGCCCACCGAGGCCAGGCGGCGCGGTGCCTTCCGTGAGGACGCATTGACCGAGGCCGA

CGCCCTGGCGGCCGCCGAGAATGAACGCCAAGAGGAACAAGCATGAAACCGCACCAGGACGGCCAGGACG

AACCGTTTTTCATTACCGAAGAGATCGAGGCGGAGATGATCGCGGCCGGGTACGTGTTCGAGCCGCCCGC

GCACGTCTCAACCGTGCGGCTGCATGAAATCCTGGCCGGTTTGTCTGATGCCAAGCTCGCGGCCTGGCCG

GCGAGCTTGGCCGCTGAAGAAACCGAGCGCCGCCGTCTAAAAAGGTGATGTGTATTTGAGTAAAACAGCT

TGCGTCATGCGGTCGCTGCGTATATGATGCGATGAGTAAATAAACAAATACGCAAGGGGAACGCATGAAG

GTTATCGCTGTACTTAACCAGAAAGGCGGGTCAGGCAAGACGACCATCGCAACCCATCTAGCCCGCGCCC

TGCAACTCGCCGGGGCCGATGTTCTGTTAGTCGATTCCGATCCCCAGGGCAGTGCCCGCGATTGGGCGGC

CGTGCGGGAAGATCAACCGCTAACCGTTGTCGGCATCGACCGCCCGACGATTGACCGCGACGTGAAGGCC

ATCGGCCGGCGCGACTTCGTAGTGATCGACGGAGCGCCCCAGGCGGCGGACTTGGCTGTGTCCGCGATCA

AGGCAGCCGACTTCGTGCTGATTCCGGTGCAGCCAAGCCCTTACGACATATGGGCCACCGCCGACCTGGT

GGAGCTGGTTAAGCAGCGCATTGAGGTCACGGATGGAAGGCTACAAGCGGCCTTTGTCGTGTCGCGGGCG

ATCAAAGGCACGCGCATCGGCGGTGAGGTTGCCGAGGCGCTGGCCGGGTACGAGCTGCCCATTCTTGAGT

CCCGTATCACGCAGCGCGTGAGCTACCCAGGCACTGCCGCCGCCGGCACAACCGTTCTTGAATCAGAACC

CGAGGGCGACGCTGCCCGCGAGGTCCAGGCGCTGGCCGCTGAAATTAAATCAAAACTCATTTGAGTTAAT

GAGGTAAAGAGAAAATGAGCAAAAGCACAAACACGCTAAGTGCCGGCCGTCCGAGCGCACGCAGCAGCAA

GGCTGCAACGTTGGCCAGCCTGGCAGACACGCCAGCCATGAAGCGGGTCAACTTTCAGTTGCCGGCGGAG

GATCACACCAAGCTGAAGATGTACGCGGTACGCCAAGGCAAGACCATTACCGAGCTGCTATCTGAATACA

TCGCGCAGCTACCAGAGTAAATGAGCAAATGAATAAATGAGTAGATGAATTTTAGCGGCTAAAGGAGGCG

GCATGGAAAATCAAGAACAACCAGGCACCGACGCCGTGGAATGCCCCATGTGTGGAGGAACGGGCGGTTG

GCCAGGCGTAAGCGGCTGGGTTGTCTGCCGGCCCTGCAATGGCACTGGAACCCCCAAGCCCGAGGAATCG

GCGTGAGCGGTCGCAAACCATCCGGCCCGGTACAAATCGGCGCGGCGCTGGGTGATGACCTGGTGGAGAA

GTTGAAGGCGGCGCAGGCCGCCCAGCGGCAACGCATCGAGGCAGAAGCACGCCCCGGTGAATCGTGGCAA

GCGGCCGCTGATCGAATCCGCAAAGAATCCCGGCAACCGCCGGCAGCCGGTGCGCCGTCGATTAGGAAGC

CGCCCAAGGGCGACGAGCAACCAGATTTTTTCGTTCCGATGCTCTATGACGTGGGCACCCGCGATAGTCG

CAGCATCATGGACGTGGCCGTTTTCCGTCTGTCGAAGCGTGACCGACGAGCTGGCGAGGTGATCCGCTAC

GAGCTTCCAGACGGGCACGTAGAGGTTTCCGCAGGGCCGGCCGGCATGGCGAGTGTGTGGGATTACGACC

TGGTACTGATGGCGGTTTCCCATCTAACCGAATCCATGAACCGATACCGGGAAGGGAAGGGAGACAAGCC

CGGCCGCGTGTTCCGTCCACACGTTGCGGACGTACTCAAGTTCTGCCGGCGAGCCGATGGCGGAAAGCAG

AAAGACGACCTGGTAGAAACCTGCATTCGGTTAAACACCACGCACGTTGCCATGCAGCGTACGAAGAAGG

CCAAGAACGGCCGCCTGGTGACGGTATCCGAGGGTGAAGCCTTGATTAGCCGCTACAAGATCGTAAAGAG

CGAAACCGGGCGGCCGGAGTACATCGAGATCGAGCTAGCTGATTGGATGTACCGCGAGATCACAGAAGGC

AAGAACCCGGACGTGCTGACGGTTCACCCCGATTACTTTTTGATCGATCCCGGCATCGGCCGTTTTCTCT

ACCGCCTGGCACGCCGCGCCGCAGGCAAGGCAGAAGCCAGATGGTTGTTCAAGACGATCTACGAACGCAG

TGGCAGCGCCGGAGAGTTCAAGAAGTTCTGTTTCACCGTGCGCAAGCTGATCGGGTCAAATGACCTGCCG

GAGTACGATTTGAAGGAGGAGGCGGGGCAGGCTGGCCCGATCCTAGTCATGCGCTACCGCAACCTGATCG

AGGGCGAAGCATCCGCCGGTTCCTAATGTACGGAGCAGATGCTAGGGCAAATTGCCCTAGCAGGGGAAAA

AGGTCGAAAAGGTCTCTTTCCTGTGGATAGCACGTACATTGGGAACCCAAAGCCGTACATTGGGAACCGG

AACCCGTACATTGGGAACCCAAAGCCGTACATTGGGAACCGGTCACACATGTAAGTGACTGATATAAAAG

AGAAAAAAGGCGATTTTTCCGCCTAAAACTCTTTAAAACTTATTAAAACTCTTAAAACCCGCCTGGCCTG

TGCATAACTGTCTGGCCAGCGCACAGCCGAAGAGCTGCAAAAAGCGCCTACCCTTCGGTCGCTGCGCTCC

CTACGCCCCGCCGCTTCGCGTCGGCCTATCGCGGCCGCTGGCCGCTCAAAAATGGCTGGCCTACGGCCAG

GCAATCTACCAGGGCGCGGACAAGCCGCGCCGTCGCCACTCGACCGCCGGCGCCCACATCAAGGCACCGG

TGGGTATGCCTGACGATGCGTGGAGACCGAAACCTTGCGCTCGTTCGCCAGCCAGGACAGAAATGCCTCG

ACTTCGCTGCTGCCCAAGGTTGCCGGGTGACGCACACCGTGGAAACGGATGAAGGCACGAACCCAGTGGA

CATAAGCCTGTTCGGTTCGTAAGCTGTAATGCAAGTAGCGTATGCGCTCACGCAACTGGTCCAGAACCTT

GACCGAACGCAGCGGTGGTAACGGCGCAGTGGCGGTTTTCATGGCTTGTTATGACTGTTTTTTTGGGGTA

CAGTCTATGCCTCGGGCATCCAAGCAGCAAGCGCGTTACGCCGTGGGTCGATGTTTGATGTTATGGAGCA

GCAACGATGTTACGCAGCAGGGCAGTCGCCCTAAAACAAAGTTAAACATCATGAGGGAAGCGGTGATCGC

CGAAGTATCGACTCAACTATCAGAGGTAGTTGGCGTCATCGAGCGCCATCTCGAACCGACGTTGCTGGCC

GTACATTTGTACGGCTCCGCAGTGGATGGCGGCCTGAAGCCACACAGTGATATTGATTTGCTGGTTACGG

TGACCGTAAGGCTTGATGAAACAACGCGGCGAGCTTTGATCAACGACCTTTTGGAAACTTCGGCTTCCCC

TGGAGAGAGCGAGATTCTCCGCGCTGTAGAAGTCACCATTGTTGTGCACGACGACATCATTCCGTGGCGT

TATCCAGCTAAGCGCGAACTGCAATTTGGAGAATGGCAGCGCAATGACATTCTTGCAGGTATCTTCGAGC

CAGCCACGATCGACATTGATCTGGCTATCTTGCTGACAAAAGCAAGAGAACATAGCGTTGCCTTGGTAGG

TCCAGCGGCGGAGGAACTCTTTGATCCGGTTCCTGAACAGGATCTATTTGAGGCGCTAAATGAAACCTTA

ACGCTATGGAACTCGCCGCCCGACTGGGCTGGCGATGAGCGAAATGTAGTGCTTACGTTGTCCCGCATTT

GGTACAGCGCAGTAACCGGCAAAATCGCGCCGAAGGATGTCGCTGCCGACTGGGCAATGGAGCGCCTGCC

GGCCCAGTATCAGCCCGTCATACTTGAAGCTAGACAGGCTTATCTTGGACAAGAAGAAGATCGCTTGGCC

TCGCGCGCAGATCAGTTGGAAGAATTTGTCCACTACGTGAAAGGCGAGATCACCAAGGTAGTCGGCAAAT

AATGTCTAACAATTCGTTCAAGCCGACGCCGCTTCGCGGCGCGGCTTAACTCAAGCGTTAGATGCACTAA

GCACATAATTGCTCACAGCCAAACTATCAGGTCAAGTCTGCTTTTATTATTTTTAAGCGTGCATAATAAG

CCCTACACAAATTGGGAGATATATCATGAAAGGCTGGCTTTTTCTTGTTATCGCAATAGTTGGCGAAGTA

ATCGCAACATAGCTTGCTTGGTCGTTCCGCGTGAACGTCGGCTCGATTGTACCTGCGTTCAAATACTTTG

CGATCGTGTTGCGCGCCTGCCCGGTGCGTCGGCTGATCTCACGGATCGACTGCTTCTCTCGCAACGCCAT

CCGACGGATGATGTTTAAAAGTCCCATGTGGATCACTCCGTTGCCCCGTCGCTCACCGTGTTGGGGGGAA

GGTGCACATGGCTCAGTTCTCAATGGAAATTATCTGCCTAACCGGCTCAGTTCTGCGTAGAAACCAACAT

GCAAGCTCCACCGGGTGCAAAGCGGCAGCGGCGGCAGGATATATTCAATTGTAAATGGCTTCATGTCCGG

GAAATCTACATGGATCAGCAATGAGTATGATGGTCAATATGGAGAAAAAGAAAGAGTAATTACCAATTTT

TTTTCAATTCAAAAATGTAGATGTCCGCAGCGTTATTATAAAATGAAAGTACATTTTGATAAAACGACAA

ATTACGATCCGTCGTATTTATAGGCGAAAGCAATAAACAAATTATTCTAATTCGGAAATCTTTATTTCGA

CGTGTCTACATTCACGTCCAAATGGGGGCTTAGATGAGAAACTTCACGATCGGC

>gi|18766333|gb|AF458479.1| Binary vector pLH9500, complete sequence

TCTAGAACATGGTGGAGCACGACACTCTCGTCTACTCCAAGAATATCAAAGATACAGTCTCAGAAGACCA

GAGGGCTATTGAGACTTTTCAACAAAGGGTAATATCGGGAAACCTCCTCGGATTCCATTGCCCAGCTATC

TGTCACTTCATCGAAAGGACAGTAGAAAAGGAAGATGGCTTCTACAAATGCCATCATTGCGATAAAGGAA

AGGCTATCGTTCAAGATGCCTCTACCGACAGTGGTCCCAAAGATGGACCCCCACCCACGAGGAACATCGT

GGAAAAAGAAGACGTTCCAACCACGTCTTCAAAGCAAGTGGATTGATGTGATATCTCCACTGACGTAAGG

GATGACGCACAATCCCACTATCCTTCGCAAGACCCTTCCTCTATATAAGGAAGTTCATTTCATTTGGAGA

GGACCTCGAGTGGCCACCATGGGAGCTTGGATTGAACAAGATGGATTGCACGCAGGTTCTCCGGCCGCTT

GGGTGGAGAGGCTATTCGGCTATGACTGGGCACAACAGACAATCGGCTGCTCTGATGCCGCCGTGTTCCG

GCTGTCAGCGCAGGGGCGCCCGGTTCTTTTTGTCAAGACCGACCTGTCCGGTGCCCTGAATGAACTGCAG

GACGAGGCAGCGCGGCTATCGTGGCTGGCCACGACGGGCGTTCCTTGCGCAGCTGTGCTCGACGTTGTCA

CTGAAGCGGGAAGGGACTGGCTGCTATTGGGCGAAGTGCCGGGGCAGGATCTCCTGTCATCTCACCTTGC

TCCTGCCGAGAAAGTATCCATCATGGCTGATGCAATGCGGCGGCTGCATACGCTTGATCCGGCTACCTGC

CCATTCGACCACCAAGCGAAACATCGCATCGAGCGAGCACGTACTCGGATGGAAGCCGGTCTTGTCGATC

AGGATGATCTGGACGAAGAGCATCAGGGGCTCGCGCCAGCCGAACTGTTCGCCAGGCTCAAGGCGCGCAT

GCCCGACGGCGAGGATCTCGTCGTGACCCATGGCGATGCCTGCTTGCCGAATATCATGGTGGAAAATGGC

CGCTTTTCTGGATTCATCGACTGTGGCCGGCTGGGTGTGGCGGACCGCTATCAGGACATAGCGTTGGCTA

CCCGTGATATTGCTGAAGAGCTTGGCGGCGAATGGGCTGACCGCTTCCTCGTGCTTTACGGTATCGCCGC

TCCCGATTCGCAGCGCATCGCCTTCTATCGCCTTCTTGACGAGTTCTTCTGAGCGGGACCCGCAAAAATC

ACCAGTCTCTCTCTACAAATCTATCTCTCTCTATTTTTCTCCAGAATAATGTGTGAGTAGTTCCCAGATA

AGGGAATTAGGGTTCTTATAGGGTTTCGCTCATGTGTTGAGCATATAAGAAACCCTTAGTATGTATTTGT

ATTTGTAAAATACTTCTATCAATAAAATTTCTAATTCCTAAAACCAAAATCCAGTGACCTGCAGGCATGC

AAGCTAGAGGCCCTTAAGGCCTTACTAGTGGATCCCCCGGGATGCATGAATTCAAGCTTATCGATGTCGA

CGCGGCCGCCATGGCCAGATCTGGGCCCGGTACCCGATCAGATTGTCGTTTCCCGCCTTCGGTTTAAACT

ATCAGTGTTTGACAGGATATATTGGCGGGTAAACCTAAGAGAAAAGAGCGTTTATTAGAATAATCGGATA

TTTAAAAGGGCGTGAAAAGGTTTATCCGTTCGTCCATTTGTATGTGCATGCCAACCACAGGGTTCCCCTC

GGGAGTGCTTGGCATTCCGTGCGATAATGACTTCTGTTCAACCACCCAAACGTCGGAAAGCCTGACGACG

GAGCAGCATTCCAAAAAGATCCCTTGGCTCGTCTGGGTCGGCTAGAAGGTCGAGTGGGCTGCTGTGGCTT

GATCCCTCAACGCGGTCGCGGACGTAGCGCAGCGCCGAAAAATCCTCGATCGCAAATCCGACGCTGTCGA

AAAGCGTGATCTGCTTGTCGCTCTTTCGGCCGACGTCCTGGCCAGTCATCACGCGCCAAAGTTCCGTCAC

AGGATGATCTGGCGCGAGTTGCTGGATCTCGCCTTCAATCCGGGTCTGTGGCGGGAACTCCACGAAAATA

TCCGAACGCAGCAAGATATCGCGGTGCATCTCGGTCTTGCCTGGGCAGTCGCCGCCGACGCCGTTGATGT

GGACGCCGAAAAGGATCTAGGTGAAGATCCTTTTTGATAATCTCATGACCAAAATCCCTTAACGTGAGTT

TTCGTTCCACTGAGCGTCAGACCCCGTAGAAAAGATCAAAGGATCTTCTTGAGATCCTTTTTTTCTGCGC

GTAATCTGCTGCTTGCAAACAAAAAAACCACCGCTACCAGCGGTGGTTTGTTTGCCGGATCAAGAGCTAC

CAACTCTTTTTCCGAAGGTAACTGGCTTCAGCAGAGCGCAGATACCAAATACTGTTCTTCTAGTGTAGCC

GTAGTTAGGCCACCACTTCAAGAACTCTGTAGCACCGCCTACATACCTCGCTCTGCTAATCCTGTTACCA

GTGGCTGCTGCCAGTGGCGATAAGTCGTGTCTTACCGGGTTGGACTCAAGACGATAGTTACCGGATAAGG

CGCAGCGGTCGGGCTGAACGGGGGGTTCGTGCACACAGCCCAGCTTGGAGCGAACGACCTACACCGAACT

GAGATACCTACAGCGTGAGCTATGAGAAAGCGCCACGCTTCCCGAAGGGAGAAAGGCGGACAGGTATCCG

GTAAGCGGCAGGGTCGGAACAGGAGAGCGCACGAGGGAGCTTCCAGGGGGAAACGCCTGGTATCTTTATA

GTCCTGTCGGGTTTCGCCACCTCTGACTTGAGCGTCGATTTTTGTGATGCTCGTCAGGGGGGCGGAGCCT

ATGGAAAAACGCCAGCAACGCGGCCTTTTTACGGTTCCTGGCCTTTTGCTGGCCTTTTGCTCACATGTTC

TTTCCTGCGTTATCCCCTGATTCTGTGGATAACCGATTACCGCCTTTGAGTGAGCTGATACCGCTCGCCG

CAGCCGAACGACCGAGCGCAGCGAGTCAGTGAGCGAGGAAGCGGAAGAGCGCCTGATGCGGTATTTTCTC

CTTACGCATCTGTGCGGTATTTCACACCGCATATGGTGCACTCTCAGTACAATCTGCTCTGATGCCGCAT

AGTTAAGCCAGTATACACTCCGCTATCGCTACGTGACTGGGTCATGGCTGCGCCCCGACACCCGCCAACA

CCCGCTGACGCGCCCTGACGGGCTTGTCTGCTCCCGGCATCCGCTTACAGACAAGCTGTGACCGTCTCCG

GGAGCTGCATGTGTCAGAGGTTTTCACCGTCATCACCGAAACGCGCGAGGCAGGGGTACGTCGAGGTCGA

TCCAACCCCTCCGCTGCTATAGTGCAGTCGGCTTCTGACGTTCAGTGCAGCCGTCTTCTGAAAACGACAT

GTCGCACAAGTCCTAAGTTACGCGACAGGCTGCCGCCCTGCCCTTTTCCTGGCGTTTTCTTGTCGCGTGT

TTTAGTCGCATAAAGTAGAATACTTGCGACTAGAACCGGAGACATTACGCCATGAACAAGAGCGCCGCCG

CTGGCCTGCTGGGCTATGCCCGCGTCAGCACCGACGACCAGGACTTGACCAACCAACGGGCCGAACTGCA

CGCGGCCGGCTGCACCAAGCTGTTTTCCGAGAAGATCACCGGCACCAGGCGCGACCGCCCGGAGCTGGCC

AGGATGCTTGACCACCTACGCCCTGGCGACGTTGTGACAGTGACCAGGCTAGACCGCCTGGCCCGCAGCA

CCCGCGACCTACTGGACATTGCCGAGCGCATCCAGGAGGCCGGCGCGGGCCTGCGTAGCCTGGCAGAGCC

GTGGGCCGACACCACCACGCCGGCCGGCCGCATGGTGTTGACCGTGTTCGCCGGCATTGCCGAGTTCGAG

CGTTCCCTAATCATCGACCGCACCCGGAGCGGGCGCGAGGCCGCCAAGGCGCGAGGCGTGAAGTTTGGCC

CCCGCCCTACCCTCACCCCGGCACAGATCGCGCACGCCCGCGAGCTGATCGACCAGGAAGGCCGCACCGT

GAAAGAGGCGGCTGCACTGCTTGGCGTGCATCGCTCGACCCTGTACCGCGCACTTGAGCGCAGCGAGGAA

GTGACGCCCACCGAGGCCAGGCGGCGCGGTGCCTTCCGTGAGGACGCATTGACCGAGGCCGACGCCCTGG

CGGCCGCCGAGAATGAACGCCAAGAGGAACAAGCATGAAACCGCACCAGGACGGCCAGGACGAACCGTTT

TTCATTACCGAAGAGATCGAGGCGGAGATGATCGCGGCCGGGTACGTGTTCGAGCCGCCCGCGCACGTCT

CAACCGTGCGGCTGCATGAAATCCTGGCCGGTTTGTCTGATGCCAAGCTCGCGGCCTGGCCGGCGAGCTT

GGCCGCTGAAGAAACCGAGCGCCGCCGTCTAAAAAGGTGATGTGTATTTGAGTAAAACAGCTTGCGTCAT

GCGGTCGCTGCGTATATGATGCGATGAGTAAATAAACAAATACGCAAGGGGAACGCATGAAGGTTATCGC

TGTACTTAACCAGAAAGGCGGGTCAGGCAAGACGACCATCGCAACCCATCTAGCCCGCGCCCTGCAACTC

GCCGGGGCCGATGTTCTGTTAGTCGATTCCGATCCCCAGGGCAGTGCCCGCGATTGGGCGGCCGTGCGGG

AAGATCAACCGCTAACCGTTGTCGGCATCGACCGCCCGACGATTGACCGCGACGTGAAGGCCATCGGCCG

GCGCGACTTCGTAGTGATCGACGGAGCGCCCCAGGCGGCGGACTTGGCTGTGTCCGCGATCAAGGCAGCC

GACTTCGTGCTGATTCCGGTGCAGCCAAGCCCTTACGACATATGGGCCACCGCCGACCTGGTGGAGCTGG

TTAAGCAGCGCATTGAGGTCACGGATGGAAGGCTACAAGCGGCCTTTGTCGTGTCGCGGGCGATCAAAGG

CACGCGCATCGGCGGTGAGGTTGCCGAGGCGCTGGCCGGGTACGAGCTGCCCATTCTTGAGTCCCGTATC

ACGCAGCGCGTGAGCTACCCAGGCACTGCCGCCGCCGGCACAACCGTTCTTGAATCAGAACCCGAGGGCG

ACGCTGCCCGCGAGGTCCAGGCGCTGGCCGCTGAAATTAAATCAAAACTCATTTGAGTTAATGAGGTAAA

GAGAAAATGAGCAAAAGCACAAACACGCTAAGTGCCGGCCGTCCGAGCGCACGCAGCAGCAAGGCTGCAA

CGTTGGCCAGCCTGGCAGACACGCCAGCCATGAAGCGGGTCAACTTTCAGTTGCCGGCGGAGGATCACAC

CAAGCTGAAGATGTACGCGGTACGCCAAGGCAAGACCATTACCGAGCTGCTATCTGAATACATCGCGCAG

CTACCAGAGTAAATGAGCAAATGAATAAATGAGTAGATGAATTTTAGCGGCTAAAGGAGGCGGCATGGAA

AATCAAGAACAACCAGGCACCGACGCCGTGGAATGCCCCATGTGTGGAGGAACGGGCGGTTGGCCAGGCG

TAAGCGGCTGGGTTGTCTGCCGGCCCTGCAATGGCACTGGAACCCCCAAGCCCGAGGAATCGGCGTGAGC

GGTCGCAAACCATCCGGCCCGGTACAAATCGGCGCGGCGCTGGGTGATGACCTGGTGGAGAAGTTGAAGG

CGGCGCAGGCCGCCCAGCGGCAACGCATCGAGGCAGAAGCACGCCCCGGTGAATCGTGGCAAGCGGCCGC

TGATCGAATCCGCAAAGAATCCCGGCAACCGCCGGCAGCCGGTGCGCCGTCGATTAGGAAGCCGCCCAAG

GGCGACGAGCAACCAGATTTTTTCGTTCCGATGCTCTATGACGTGGGCACCCGCGATAGTCGCAGCATCA

TGGACGTGGCCGTTTTCCGTCTGTCGAAGCGTGACCGACGAGCTGGCGAGGTGATCCGCTACGAGCTTCC

AGACGGGCACGTAGAGGTTTCCGCAGGGCCGGCCGGCATGGCGAGTGTGTGGGATTACGACCTGGTACTG

ATGGCGGTTTCCCATCTAACCGAATCCATGAACCGATACCGGGAAGGGAAGGGAGACAAGCCCGGCCGCG

TGTTCCGTCCACACGTTGCGGACGTACTCAAGTTCTGCCGGCGAGCCGATGGCGGAAAGCAGAAAGACGA

CCTGGTAGAAACCTGCATTCGGTTAAACACCACGCACGTTGCCATGCAGCGTACGAAGAAGGCCAAGAAC

GGCCGCCTGGTGACGGTATCCGAGGGTGAAGCCTTGATTAGCCGCTACAAGATCGTAAAGAGCGAAACCG

GGCGGCCGGAGTACATCGAGATCGAGCTAGCTGATTGGATGTACCGCGAGATCACAGAAGGCAAGAACCC

GGACGTGCTGACGGTTCACCCCGATTACTTTTTGATCGATCCCGGCATCGGCCGTTTTCTCTACCGCCTG

GCACGCCGCGCCGCAGGCAAGGCAGAAGCCAGATGGTTGTTCAAGACGATCTACGAACGCAGTGGCAGCG

CCGGAGAGTTCAAGAAGTTCTGTTTCACCGTGCGCAAGCTGATCGGGTCAAATGACCTGCCGGAGTACGA

TTTGAAGGAGGAGGCGGGGCAGGCTGGCCCGATCCTAGTCATGCGCTACCGCAACCTGATCGAGGGCGAA

GCATCCGCCGGTTCCTAATGTACGGAGCAGATGCTAGGGCAAATTGCCCTAGCAGGGGAAAAAGGTCGAA

AAGGTCTCTTTCCTGTGGATAGCACGTACATTGGGAACCCAAAGCCGTACATTGGGAACCGGAACCCGTA

CATTGGGAACCCAAAGCCGTACATTGGGAACCGGTCACACATGTAAGTGACTGATATAAAAGAGAAAAAA

GGCGATTTTTCCGCCTAAAACTCTTTAAAACTTATTAAAACTCTTAAAACCCGCCTGGCCTGTGCATAAC

TGTCTGGCCAGCGCACAGCCGAAGAGCTGCAAAAAGCGCCTACCCTTCGGTCGCTGCGCTCCCTACGCCC

CGCCGCTTCGCGTCGGCCTATCGCGGCCGCTGGCCGCTCAAAAATGGCTGGCCTACGGCCAGGCAATCTA

CCAGGGCGCGGACAAGCCGCGCCGTCGCCACTCGACCGCCGGCGCCCACATCAAGGCACCGGTGGGTATG

CCTGACGATGCGTGGAGACCGAAACCTTGCGCTCGTTCGCCAGCCAGGACAGAAATGCCTCGACTTCGCT

GCTGCCCAAGGTTGCCGGGTGACGCACACCGTGGAAACGGATGAAGGCACGAACCCAGTGGACATAAGCC

TGTTCGGTTCGTAAGCTGTAATGCAAGTAGCGTATGCGCTCACGCAACTGGTCCAGAACCTTGACCGAAC

GCAGCGGTGGTAACGGCGCAGTGGCGGTTTTCATGGCTTGTTATGACTGTTTTTTTGGGGTACAGTCTAT

GCCTCGGGCATCCAAGCAGCAAGCGCGTTACGCCGTGGGTCGATGTTTGATGTTATGGAGCAGCAACGAT

GTTACGCAGCAGGGCAGTCGCCCTAAAACAAAGTTAAACATCATGAGGGAAGCGGTGATCGCCGAAGTAT

CGACTCAACTATCAGAGGTAGTTGGCGTCATCGAGCGCCATCTCGAACCGACGTTGCTGGCCGTACATTT

GTACGGCTCCGCAGTGGATGGCGGCCTGAAGCCACACAGTGATATTGATTTGCTGGTTACGGTGACCGTA

AGGCTTGATGAAACAACGCGGCGAGCTTTGATCAACGACCTTTTGGAAACTTCGGCTTCCCCTGGAGAGA

GCGAGATTCTCCGCGCTGTAGAAGTCACCATTGTTGTGCACGACGACATCATTCCGTGGCGTTATCCAGC

TAAGCGCGAACTGCAATTTGGAGAATGGCAGCGCAATGACATTCTTGCAGGTATCTTCGAGCCAGCCACG

ATCGACATTGATCTGGCTATCTTGCTGACAAAAGCAAGAGAACATAGCGTTGCCTTGGTAGGTCCAGCGG

CGGAGGAACTCTTTGATCCGGTTCCTGAACAGGATCTATTTGAGGCGCTAAATGAAACCTTAACGCTATG

GAACTCGCCGCCCGACTGGGCTGGCGATGAGCGAAATGTAGTGCTTACGTTGTCCCGCATTTGGTACAGC

GCAGTAACCGGCAAAATCGCGCCGAAGGATGTCGCTGCCGACTGGGCAATGGAGCGCCTGCCGGCCCAGT

ATCAGCCCGTCATACTTGAAGCTAGACAGGCTTATCTTGGACAAGAAGAAGATCGCTTGGCCTCGCGCGC

AGATCAGTTGGAAGAATTTGTCCACTACGTGAAAGGCGAGATCACCAAGGTAGTCGGCAAATAATGTCTA

ACAATTCGTTCAAGCCGACGCCGCTTCGCGGCGCGGCTTAACTCAAGCGTTAGATGCACTAAGCACATAA

TTGCTCACAGCCAAACTATCAGGTCAAGTCTGCTTTTATTATTTTTAAGCGTGCATAATAAGCCCTACAC

AAATTGGGAGATATATCATGAAAGGCTGGCTTTTTCTTGTTATCGCAATAGTTGGCGAAGTAATCGCAAC

ATAGCTTGCTTGGTCGTTCCGCGTGAACGTCGGCTCGATTGTACCTGCGTTCAAATACTTTGCGATCGTG

TTGCGCGCCTGCCCGGTGCGTCGGCTGATCTCACGGATCGACTGCTTCTCTCGCAACGCCATCCGACGGA

TGATGTTTAAAAGTCCCATGTGGATCACTCCGTTGCCCCGTCGCTCACCGTGTTGGGGGGAAGGTGCACA

TGGCTCAGTTCTCAATGGAAATTATCTGCCTAACCGGCTCAGTTCTGCGTAGAAACCAACATGCAAGCTC

CACCGGGTGCAAAGCGGCAGCGGCGGCAGGATATATTCAATTGTAAATGGCTTCATGTCCGGGAAATCTA

CATGGATCAGCAATGAGTATGATGGTCAATATGGAGAAAAAGAAAGAGTAATTACCAATTTTTTTTCAAT

TCAAAAATGTAGATGTCCGCAGCGTTATTATAAAATGAAAGTACATTTTGATAAAACGACAAATTACGAT

CCGTCGTATTTATAGGCGAAAGCAATAAACAAATTATTCTAATTCGGAAATCTTTATTTCGACGTGTCTA

CATTCACGTCCAAATGGGGGCTTAGATGAGAAACTTCACGATCGGC

>gi|19569229|gb|AF485783.1| Binary vector pBI121, complete sequence

TGAGCGTCGCAAAGGCGCTCGGTCTTGCCTTGCTCGTCGGTGATGTACTTCACCAGCTCCGCGAAGTCGC

TCTTCTTGATGGAGCGCATGGGGACGTGCTTGGCAATCACGCGCACCCCCCGGCCGTTTTAGCGGCTAAA

AAAGTCATGGCTCTGCCCTCGGGCGGACCACGCCCATCATGACCTTGCCAAGCTCGTCCTGCTTCTCTTC

GATCTTCGCCAGCAGGGCGAGGATCGTGGCATCACCGAACCGCGCCGTGCGCGGGTCGTCGGTGAGCCAG

AGTTTCAGCAGGCCGCCCAGGCGGCCCAGGTCGCCATTGATGCGGGCCAGCTCGCGGACGTGCTCATAGT

CCACGACGCCCGTGATTTTGTAGCCCTGGCCGACGGCCAGCAGGTAGGCCGACAGGCTCATGCCGGCCGC

CGCCGCCTTTTCCTCAATCGCTCTTCGTTCGTCTGGAAGGCAGTACACCTTGATAGGTGGGCTGCCCTTC

CTGGTTGGCTTGGTTTCATCAGCCATCCGCTTGCCCTCATCTGTTACGCCGGCGGTAGCCGGCCAGCCTC

GCAGAGCAGGATTCCCGTTGAGCACCGCCAGGTGCGAATAAGGGACAGTGAAGAAGGAACACCCGCTCGC

GGGTGGGCCTACTTCACCTATCCTGCCCGGCTGACGCCGTTGGATACACCAAGGAAAGTCTACACGAACC

CTTTGGCAAAATCCTGTATATCGTGCGAAAAAGGATGGATATACCGAAAAAATCGCTATAATGACCCCGA

AGCAGGGTTATGCAGCGGAAAAGCGCCACGCTTCCCGAAGGGAGAAAGGCGGACAGGTATCCGGTAAGCG

GCAGGGTCGGAACAGGAGAGCGCACGAGGGAGCTTCCAGGGGGAAACGCCTGGTATCTTTATAGTCCTGT

CGGGTTTCGCCACCTCTGACTTGAGCGTCGATTTTTGTGATGCTCGTCAGGGGGGCGGAGCCTATGGAAA

AACGCCAGCAACGCGGCCTTTTTACGGTTCCTGGCCTTTTGCTGGCCTTTTGCTCACATGTTCTTTCCTG

CGTTATCCCCTGATTCTGTGGATAACCGTATTACCGCCTTTGAGTGAGCTGATACCGCTCGCCGCAGCCG

AACGACCGAGCGCAGCGAGTCAGTGAGCGAGGAAGCGGAAGAGCGCCAGAAGGCCGCCAGAGAGGCCGAG

CGCGGCCGTGAGGCTTGGACGCTAGGGCAGGGCATGAAAAAGCCCGTAGCGGGCTGCTACGGGCGTCTGA

CGCGGTGGAAAGGGGGAGGGGATGTTGTCTACATGGCTCTGCTGTAGTGAGTGGGTTGCGCTCCGGCAGC

GGTCCTGATCAATCGTCACCCTTTCTCGGTCCTTCAACGTTCCTGACAACGAGCCTCCTTTTCGCCAATC

CATCGACAATCACCGCGAGTCCCTGCTCGAACGCTGCGTCCGGACCGGCTTCGTCGAAGGCGTCTATCGC

GGCCCGCAACAGCGGCGAGAGCGGAGCCTGTTCAACGGTGCCGCCGCGCTCGCCGGCATCGCTGTCGCCG

GCCTGCTCCTCAAGCACGGCCCCAACAGTGAAGTAGCTGATTGTCATCAGCGCATTGACGGCGTCCCCGG

CCGAAAAACCCGCCTCGCAGAGGAAGCGAAGCTGCGCGTCGGCCGTTTCCATCTGCGGTGCGCCCGGTCG

CGTGCCGGCATGGATGCGCGCGCCATCGCGGTAGGCGAGCAGCGCCTGCCTGAAGCTGCGGGCATTCCCG

ATCAGAAATGAGCGCCAGTCGTCGTCGGCTCTCGGCACCGAATGCGTATGATTCTCCGCCAGCATGGCTT

CGGCCAGTGCGTCGAGCAGCGCCCGCTTGTTCCTGAAGTGCCAGTAAAGCGCCGGCTGCTGAACCCCCAA

CCGTTCCGCCAGTTTGCGTGTCGTCAGACCGTCTACGCCGACCTCGTTCAACAGGTCCAGGGCGGCACGG

ATCACTGTATTCGGCTGCAACTTTGTCATGCTTGACACTTTATCACTGATAAACATAATATGTCCACCAA

CTTATCAGTGATAAAGAATCCGCGCGTTCAATCGGACCAGCGGAGGCTGGTCCGGAGGCCAGACGTGAAA

CCCAACATACCCCTGATCGTAATTCTGAGCACTGTCGCGCTCGACGCTGTCGGCATCGGCCTGATTATGC

CGGTGCTGCCGGGCCTCCTGCGCGATCTGGTTCACTCGAACGACGTCACCGCCCACTATGGCATTCTGCT

GGCGCTGTATGCGTTGGTGCAATTTGCCTGCGCACCTGTGCTGGGCGCGCTGTCGGATCGTTTCGGGCGG

CGGCCAATCTTGCTCGTCTCGCTGGCCGGCGCCAGATCTGGGGAACCCTGTGGTTGGCATGCACATACAA

ATGGACGAACGGATAAACCTTTTCACGCCCTTTTAAATATCCGATTATTCTAATAAACGCTCTTTTCTCT

TAGGTTTACCCGCCAATATATCCTGTCAAACACTGATAGTTTAAACTGAAGGCGGGAAACGACAATCTGA

TCATGAGCGGAGAATTAAGGGAGTCACGTTATGACCCCCGCCGATGACGCGGGACAAGCCGTTTTACGTT

TGGAACTGACAGAACCGCAACGTTGAAGGAGCCACTCAGCCGCGGGTTTCTGGAGTTTAATGAGCTAAGC

ACATACGTCAGAAACCATTATTGCGCGTTCAAAAGTCGCCTAAGGTCACTATCAGCTAGCAAATATTTCT

TGTCAAAAATGCTCCACTGACGTTCCATAAATTCCCCTCGGTATCCAATTAGAGTCTCATATTCACTCTC

AATCCAAATAATCTGCACCGGATCTGGATCGTTTCGCATGATTGAACAAGATGGATTGCACGCAGGTTCT

CCGGCCGCTTGGGTGGAGAGGCTATTCGGCTATGACTGGGCACAACAGACAATCGGCTGCTCTGATGCCG

CCGTGTTCCGGCTGTCAGCGCAGGGGCGCCCGGTTCTTTTTGTCAAGACCGACCTGTCCGGTGCCCTGAA

TGAACTGCAGGACGAGGCAGCGCGGCTATCGTGGCTGGCCACGACGGGCGTTCCTTGCGCAGCTGTGCTC

GACGTTGTCACTGAAGCGGGAAGGGACTGGCTGCTATTGGGCGAAGTGCCGGGGCAGGATCTCCTGTCAT

CTCACCTTGCTCCTGCCGAGAAAGTATCCATCATGGCTGATGCAATGCGGCGGCTGCATACGCTTGATCC

GGCTACCTGCCCATTCGACCACCAAGCGAAACATCGCATCGAGCGAGCACGTACTCGGATGGAAGCCGGT

CTTGTCGATCAGGATGATCTGGACGAAGAGCATCAGGGGCTCGCGCCAGCCGAACTGTTCGCCAGGCTCA

AGGCGCGCATGCCCGACGGCGATGATCTCGTCGTGACCCATGGCGATGCCTGCTTGCCGAATATCATGGT

GGAAAATGGCCGCTTTTCTGGATTCATCGACTGTGGCCGGCTGGGTGTGGCGGACCGCTATCAGGACATA

GCGTTGGCTACCCGTGATATTGCTGAAGAGCTTGGCGGCGAATGGGCTGACCGCTTCCTCGTGCTTTACG

GTATCGCCGCTCCCGATTCGCAGCGCATCGCCTTCTATCGCCTTCTTGACGAGTTCTTCTGAGCGGGACT

CTGGGGTTCGAAATGACCGACCAAGCGACGCCCAACCTGCCATCACGAGATTTCGATTCCACCGCCGCCT

TCTATGAAAGGTTGGGCTTCGGAATCGTTTTCCGGGACGCCGGCTGGATGATCCTCCAGCGCGGGGATCT

CATGCTGGAGTTCTTCGCCCACGGGATCTCTGCGGAACAGGCGGTCGAAGGTGCCGATATCATTACGACA

GCAACGGCCGACAAGCACAACGCCACGATCCTGAGCGACAATATGATCGGGCCCGGCGTCCACATCAACG

GCGTCGGCGGCGACTGCCCAGGCAAGACCGAGATGCACCGCGATATCTTGCTGCGTTCGGATATTTTCGT

GGAGTTCCCGCCACAGACCCGGATGATCCCCGATCGTTCAAACATTTGGCAATAAAGTTTCTTAAGATTG

AATCCTGTTGCCGGTCTTGCGATGATTATCATATAATTTCTGTTGAATTACGTTAAGCATGTAATAATTA

ACATGTAATGCATGACGTTATTTATGAGATGGGTTTTTATGATTAGAGTCCCGCAATTATACATTTAATA

CGCGATAGAAAACAAAATATAGCGCGCAAACTAGGATAAATTATCGCGCGCGGTGTCATCTATGTTACTA

GATCGGGCCTCCTGTCAATGCTGGCGGCGGCTCTGGTGGTGGTTCTGGTGGCGGCTCTGAGGGTGGTGGC

TCTGAGGGTGGCGGTTCTGAGGGTGGCGGCTCTGAGGGAGGCGGTTCCGGTGGTGGCTCTGGTTCCGGTG

ATTTTGATTATGAAAAGATGGCAAACGCTAATAAGGGGGCTATGACCGAAAATGCCGATGAAAACGCGCT

ACAGTCTGACGCTAAAGGCAAACTTGATTCTGTCGCTACTGATTACGGTGCTGCTATCGATGGTTTCATT

GGTGACGTTTCCGGCCTTGCTAATGGTAATGGTGCTACTGGTGATTTTGCTGGCTCTAATTCCCAAATGG

CTCAAGTCGGTGACGGTGATAATTCACCTTTAATGAATAATTTCCGTCAATATTTACCTTCCCTCCCTCA

ATCGGTTGAATGTCGCCCTTTTGTCTTTGGCCCAATACGCAAACCGCCTCTCCCCGCGCGTTGGCCGATT

CATTAATGCAGCTGGCACGACAGGTTTCCCGACTGGAAAGCGGGCAGTGAGCGCAACGCAATTAATGTGA

GTTAGCTCACTCATTAGGCACCCCAGGCTTTACACTTTATGCTTCCGGCTCGTATGTTGTGTGGAATTGT

GAGCGGATAACAATTTCACACAGGAAACAGCTATGACCATGATTACGCCAAGCTTGCATGCCTGCAGGTC

CCCAGATTAGCCTTTTCAATTTCAGAAAGAATGCTAACCCACAGATGGTTAGAGAGGCTTACGCAGCAGG

TCTCATCAAGACGATCTACCCGAGCAATAATCTCCAGGAAATCAAATACCTTCCCAAGAAGGTTAAAGAT

GCAGTCAAAAGATTCAGGACTAACTGCATCAAGAACACAGAGAAAGATATATTTCTCAAGATCAGAAGTA

CTATTCCAGTATGGACGATTCAAGGCTTGCTTCACAAACCAAGGCAAGTAATAGAGATTGGAGTCTCTAA

AAAGGTAGTTCCCACTGAATCAAAGGCCATGGAGTCAAAGATTCAAATAGAGGACCTAACAGAACTCGCC

GTAAAGACTGGCGAACAGTTCATACAGAGTCTCTTACGACTCAATGACAAGAAGAAAATCTTCGTCAACA

TGGTGGAGCACGACACACTTGTCTACTCCAAAAATATCAAAGATACAGTCTCAGAAGACCAAAGGGCAAT

TGAGACTTTTCAACAAAGGGTAATATCCGGAAACCTCCTCGGATTCCATTGCCCAGCTATCTGTCACTTT

ATTGTGAAGATAGTGGAAAAGGAAGGTGGCTCCTACAAATGCCATCATTGCGATAAAGGAAAGGCCATCG

TTGAAGATGCCTCTGCCGACAGTGGTCCCAAAGATGGACCCCCACCCACGAGGAGCATCGTGGAAAAAGA

AGACGTTCCAACCACGTCTTCAAAGCAAGTGGATTGATGTGATATCTCCACTGACGTAAGGGATGACGCA

CAATCCCACTATCCTTCGCAAGACCCTTCCTCTATATAAGGAAGTTCATTTCATTTGGAGAGAACACGGG

GGACTCTAGAGGATCCCCGGGTGGTCAGTCCCTTATGTTACGTCCTGTAGAAACCCCAACCCGTGAAATC

AAAAAACTCGACGGCCTGTGGGCATTCAGTCTGGATCGCGAAAACTGTGGAATTGATCAGCGTTGGTGGG

AAAGCGCGTTACAAGAAAGCCGGGCAATTGCTGTGCCAGGCAGTTTTAACGATCAGTTCGCCGATGCAGA

TATTCGTAATTATGCGGGCAACGTCTGGTATCAGCGCGAAGTCTTTATACCGAAAGGTTGGGCAGGCCAG

CGTATCGTGCTGCGTTTCGATGCGGTCACTCATTACGGCAAAGTGTGGGTCAATAATCAGGAAGTGATGG

AGCATCAGGGCGGCTATACGCCATTTGAAGCCGATGTCACGCCGTATGTTATTGCCGGGAAAAGTGTACG

TATCACCGTTTGTGTGAACAACGAACTGAACTGGCAGACTATCCCGCCGGGAATGGTGATTACCGACGAA

AACGGCAAGAAAAAGCAGTCTTACTTCCATGATTTCTTTAACTATGCCGGAATCCATCGCAGCGTAATGC

TCTACACCACGCCGAACACCTGGGTGGACGATATCACCGTGGTGACGCATGTCGCGCAAGACTGTAACCA

CGCGTCTGTTGACTGGCAGGTGGTGGCCAATGGTGATGTCAGCGTTGAACTGCGTGATGCGGATCAACAG

GTGGTTGCAACTGGACAAGGCACTAGCGGGACTTTGCAAGTGGTGAATCCGCACCTCTGGCAACCGGGTG

AAGGTTATCTCTATGAACTGTGCGTCACAGCCAAAAGCCAGACAGAGTGTGATATCTACCCGCTTCGCGT

CGGCATCCGGTCAGTGGCAGTGAAGGGCGAACAGTTCCTGATTAACCACAAACCGTTCTACTTTACTGGC

TTTGGTCGTCATGAAGATGCGGACTTGCGTGGCAAAGGATTCGATAACGTGCTGATGGTGCACGACCACG

CATTAATGGACTGGATTGGGGCCAACTCCTACCGTACCTCGCATTACCCTTACGCTGAAGAGATGCTCGA

CTGGGCAGATGAACATGGCATCGTGGTGATTGATGAAACTGCTGCTGTCGGCTTTAACCTCTCTTTAGGC

ATTGGTTTCGAAGCGGGCAACAAGCCGAAAGAACTGTACAGCGAAGAGGCAGTCAACGGGGAAACTCAGC

AAGCGCACTTACAGGCGATTAAAGAGCTGATAGCGCGTGACAAAAACCACCCAAGCGTGGTGATGTGGAG

TATTGCCAACGAACCGGATACCCGTCCGCAAGGTGCACGGGAATATTTCGCGCCACTGGCGGAAGCAACG

CGTAAACTCGACCCGACGCGTCCGATCACCTGCGTCAATGTAATGTTCTGCGACGCTCACACCGATACCA

TCAGCGATCTCTTTGATGTGCTGTGCCTGAACCGTTATTACGGATGGTATGTCCAAAGCGGCGATTTGGA

AACGGCAGAGAAGGTACTGGAAAAAGAACTTCTGGCCTGGCAGGAGAAACTGCATCAGCCGATTATCATC

ACCGAATACGGCGTGGATACGTTAGCCGGGCTGCACTCAATGTACACCGACATGTGGAGTGAAGAGTATC

AGTGTGCATGGCTGGATATGTATCACCGCGTCTTTGATCGCGTCAGCGCCGTCGTCGGTGAACAGGTATG

GAATTTCGCCGATTTTGCGACCTCGCAAGGCATATTGCGCGTTGGCGGTAACAAGAAAGGGATCTTCACT

CGCGACCGCAAACCGAAGTCGGCGGCTTTTCTGCTGCAAAAACGCTGGACTGGCATGAACTTCGGTGAAA

AACCGCAGCAGGGAGGCAAACAATGAATCAACAACTCTCCTGGCGCACCATCGTCGGCTACAGCCTCGGG

AATTGCTACCGAGCTCGAATTTCCCCGATCGTTCAAACATTTGGCAATAAAGTTTCTTAAGATTGAATCC

TGTTGCCGGTCTTGCGATGATTATCATATAATTTCTGTTGAATTACGTTAAGCATGTAATAATTAACATG

TAATGCATGACGTTATTTATGAGATGGGTTTTTATGATTAGAGTCCCGCAATTATACATTTAATACGCGA

TAGAAAACAAAATATAGCGCGCAAACTAGGATAAATTATCGCGCGCGGTGTCATCTATGTTACTAGATCG

GGAATTCACTGGCCGTCGTTTTACAACGTCGTGACTGGGAAAACCCTGGCGTTACCCAACTTAATCGCCT

TGCAGCACATCCCCCTTTCGCCAGCTGGCGTAATAGCGAAGAGGCCCGCACCGATCGCCCTTCCCAACAG

TTGCGCAGCCTGAATGGCGCCCGCTCCTTTCGCTTTCTTCCCTTCCTTTCTCGCCACGTTCGCCGGCTTT

CCCCGTCAAGCTCTAAATCGGGGGCTCCCTTTAGGGTTCCGATTTAGTGCTTTACGGCACCTCGACCCCA

AAAAACTTGATTTGGGTGATGGTTCACGTAGTGGGCCATCGCCCTGATAGACGGTTTTTCGCCCTTTGAC

GTTGGAGTCCACGTTCTTTAATAGTGGACTCTTGTTCCAAACTGGAACAACACTCAACCCTATCTCGGGC

TATTCTTTTGATTTATAAGGGATTTTGCCGATTTCGGAACCACCATCAAACAGGATTTTCGCCTGCTGGG

GCAAACCAGCGTGGACCGCTTGCTGCAACTCTCTCAGGGCCAGGCGGTGAAGGGCAATCAGCTGTTGCCC

GTCTCACTGGTGAAAAGAAAAACCACCCCAGTACATTAAAAACGTCCGCAATGTGTTATTAAGTTGTCTA

AGCGTCAATTTGTTTACACCACAATATATCCTGCCACCAGCCAGCCAACAGCTCCCCGACCGGCAGCTCG

GCACAAAATCACCACTCGATACAGGCAGCCCATCAGTCCGGGACGGCGTCAGCGGGAGAGCCGTTGTAAG

GCGGCAGACTTTGCTCATGTTACCGATGCTATTCGGAAGAACGGCAACTAAGCTGCCGGGTTTGAAACAC

GGATGATCTCGCGGAGGGTAGCATGTTGATTGTAACGATGACAGAGCGTTGCTGCCTGTGATCAAATATC

ATCTCCCTCGCAGAGATCCGAATTATCAGCCTTCTTATTCATTTCTCGCTTAACCGTGACAGGCTGTCGA

TCTTGAGAACTATGCCGACATAATAGGAAATCGCTGGATAAAGCCGCTGAGGAAGCTGAGTGGCGCTATT

TCTTTAGAAGTGAACGTTGACGATATCAACTCCCCTATCCATTGCTCACCGAATGGTACAGGTCGGGGAC

CCGAAGTTCCGACTGTCGGCCTGATGCATCCCCGGCTGATCGACCCCAGATCTGGGGCTGAGAAAGCCCA

GTAAGGAAACAACTGTAGGTTCGAGTCGCGAGATCCCCCGGAACCAAAGGAAGTAGGTTAAACCCGCTCC

GATCAGGCCGAGCCACGCCAGGCCGAGAACATTGGTTCCTGTAGGCATCGGGATTGGCGGATCAAACACT

AAAGCTACTGGAACGAGCAGAAGTCCTCCGGCCGCCAGTTGCCAGGCGGTAAAGGTGAGCAGAGGCACGG

GAGGTTGCCACTTGCGGGTCAGCACGGTTCCGAACGCCATGGAAACCGCCCCCGCCAGGCCCGCTGCGAC

GCCGACAGGATCTAGCGCTGCGTTTGGTGTCAACACCAACAGCGCCACGCCCGCAGTTCCGCAAATAGCC

CCCAGGACCGCCATCAATCGTATCGGGCTACCTAGCAGAGCGGCAGAGATGAACACGACCATCAGCGGCT

GCACAGCGCCTACCGTCGCCGCGACCCCGCCCGGCAGGCGGTAGACCGAAATAAACAACAAGCTCCAGAA

TAGCGAAATATTAAGTGCGCCGAGGATGAAGATGCGCATCCACCAGATTCCCGTTGGAATCTGTCGGACG

ATCATCACGAGCAATAAACCCGCCGGCAACGCCCGCAGCAGCATACCGGCGACCCCTCGGCCTCGCTGTT

CGGGCTCCACGAAAACGCCGGACAGATGCGCCTTGTGAGCGTCCTTGGGGCCGTCCTCCTGTTTGAAGAC

CGACAGCCCAATGATCTCGCCGTCGATGTAGGCGCCGAATGCCACGGCATCTCGCAACCGTTCAGCGAAC

GCCTCCATGGGCTTTTTCTCCTCGTGCTCGTAAACGGACCCGAACATCTCTGGAGCTTTCTTCAGGGCCG

ACAATCGGATCTCGCGGAAATCCTGCACGTCGGCCGCTCCAAGCCGTCGAATCTGAGCCTTAATCACAAT

TGTCAATTTTAATCCTCTGTTTATCGGCAGTTCGTAGAGCGCGCCGTGCGTCCCGAGCGATACTGAGCGA

AGCAAGTGCGTCGAGCAGTGCCCGCTTGTTCCTGAAATGCCAGTAAAGCGCTGGCTGCTGAACCCCCAGC

CGGAACTGACCCCACAAGGCCCTAGCGTTTGCAATGCACCAGGTCATCATTGACCCAGGCGTGTTCCACC

AGGCCGCTGCCTCGCAACTCTTCGCAGGCTTCGCCGACCTGCTCGCGCCACTTCTTCACGCGGGTGGAAT

CCGATCCGCACATGAGGCGGAAGGTTTCCAGCTTGAGCGGGTACGGCTCCCGGTGCGAGCTGAAATAGTC

GAACATCCGTCGGGCCGTCGGCGACAGCTTGCGGTACTTCTCCCATATGAATTTCGTGTAGTGGTCGCCA

GCAAACAGCACGACGATTTCCTCGTCGATCAGGACCTGGCAACGGGACGTTTTCTTGCCACGGTCCAGGA

CGCGGAAGCGGTGCAGCAGCGACACCGATTCCAGGTGCCCAACGCGGTCGGACGTGAAGCCCATCGCCGT

CGCCTGTAGGCGCGACAGGCATTCCTCGGCCTTCGTGTAATACCGGCCATTGATCGACCAGCCCAGGTCC

TGGCAAAGCTCGTAGAACGTGAAGGTGATCGGCTCGCCGATAGGGGTGCGCTTCGCGTACTCCAACACCT

GCTGCCACACCAGTTCGTCATCGTCGGCCCGCAGCTCGACGCCGGTGTAGGTGATCTTCACGTCCTTGTT

GACGTGGAAAATGACCTTGTTTTGCAGCGCCTCGCGCGGGATTTTCTTGTTGCGCGTGGTGAACAGGGCA

GAGCGGGCCGTGTCGTTTGGCATCGCTCGCATCGTGTCCGGCCACGGCGCAATATCGAACAAGGAAAGCT

GCATTTCCTTGATCTGCTGCTTCGTGTGTTTCAGCAACGCGGCCTGCTTGGCCTCGCTGACCTGTTTTGC

CAGGTCCTCGCCGGCGGTTTTTCGCTTCTTGGTCGTCATAGTTCCTCGCGTGTCGATGGTCATCGACTTC

GCCAAACCTGCCGCCTCCTGTTCGAGACGACGCGAACGCTCCACGGCGGCCGATGGCGCGGGCAGGGCAG

GGGGAGCCAGTTGCACGCTGTCGCGCTCGATCTTGGCCGTAGCTTGCTGGACCATCGAGCCGACGGACTG

GAAGGTTTCGCGGGGCGCACGCATGACGGTGCGGCTTGCGATGGTTTCGGCATCCTCGGCGGAAAACCCC

GCGTCGATCAGTTCTTGCCTGTATGCCTTCCGGTCAAACGTCCGATTCATTCACCCTCCTTGCGGGATTG

CCCCGACTCACGCCGGGGCAATGTGCCCTTATTCCTGATTTGACCCGCCTGGTGCCTTGGTGTCCAGATA

ATCCACCTTATCGGCAATGAAGTCGGTCCCGTAGACCGTCTGGCCGTCCTTCTCGTACTTGGTATTCCGA

ATCTTGCCCTGCACGAATACCAGCGACCCCTTGCCCAAATACTTGCCGTGGGCCTCGGCCTGAGAGCCAA

AACACTTGATGCGGAAGAAGTCGGTGCGCTCCTGCTTGTCGCCGGCATCGTTGCGCCACATCTAGGTACT

AAAACAATTCATCCAGTAAAATATAATATTTTATTTTCTCCCAATCAGGCTTGATCCCCAGTAAGTCAAA

AAATAGCTCGACATACTGTTCTTCCCCGATATCCTCCCTGATCGACCGGACGCAGAAGGCAATGTCATAC

CACTTGTCCGCCCTGCCGCTTCTCCCAAGATCAATAAAGCCACTTACTTTGCCATCTTTCACAAAGATGT

TGCTGTCTCCCAGGTCGCCGTGGGAAAAGACAAGTTCCTCTTCGGGCTTTTCCGTCTTTAAAAAATCATA

CAGCTCGCGCGGATCTTTAAATGGAGTGTCTTCTTCCCAGTTTTCGCAATCCACATCGGCCAGATCGTTA

TTCAGTAAGTAATCCAATTCGGCTAAGCGGCTGTCTAAGCTATTCGTATAGGGACAATCCGATATGTCGA

TGGAGTGAAAGAGCCTGATGCACTCCGCATACAGCTCGATAATCTTTTCAGGGCTTTGTTCATCTTCATA

CTCTTCCGAGCAAAGGACGCCATCGGCCTCACTCATGAGCAGATTGCTCCAGCCATCATGCCGTTCAAAG

TGCAGGACCTTTGGAACAGGCAGCTTTCCTTCCAGCCATAGCATCATGTCCTTTTCCCGTTCCACATCAT

AGGTGGTCCCTTTATACCGGCTGTCCGTCATTTTTAAATATAGGTTTTCATTTTCTCCCACCAGCTTATA

TACCTTAGCAGGAGACATTCCTTCCGTATCTTTTACGCAGCGGTATTTTTCGATCAGTTTTTTCAATTCC

GGTGATATTCTCATTTTAGCCATTTATTATTTCCTTCCTCTTTTCTACAGTATTTAAAGATACCCCAAGA

AGCTAATTATAACAAGACGAACTCCAATTCACTGTTCCTTGCATTCTAAAACCTTAAATACCAGAAAACA

GCTTTTTCAAAGTTGTTTTCAAAGTTGGCGTATAACATAGTATCGACGGAGCCGATTTTGAAACCACAAT

TATGGGTGATGCTGCCAACTTACTGATTTAGTGTATGATGGTGTTTTTGAGGTGCTCCAGTGGCTTCTGT

GTCTATCAGCTGTCCCTCCTGTTCAGCTACTGACGGGGTGGTGCGTAACGGCAAAAGCACCGCCGGACAT

CAGCGCTATCTCTGCTCTCACTGCCGTAAAACATGGCAACTGCAGTTCACTTACACCGCTTCTCAACCCG

GTACGCACCAGAAAATCATTGATATGGCCATGAATGGCGTTGGATGCCGGGCAACAGCCCGCATTATGGG

CGTTGGCCTCAACACGATTTTACGTCACTTAAAAAACTCAGGCCGCAGTCGGTAACCTCGCGCATACAGC

CGGGCAGTGACGTCATCGTCTGCGCGGAAATGGACGAACAGTGGGGCTATGTCGGGGCTAAATCGCGCCA

GCGCTGGCTGTTTTACGCGTATGACAGTCTCCGGAAGACGGTTGTTGCGCACGTATTCGGTGAACGCACT

ATGGCGACGCTGGGGCGTCTTATGAGCCTGCTGTCACCCTTTGACGTGGTGATATGGATGACGGATGGCT

GGCCGCTGTATGAATCCCGCCTGAAGGGAAAGCTGCACGTAATCAGCAAGCGATATACGCAGCGAATTGA

GCGGCATAACCTGAATCTGAGGCAGCACCTGGCACGGCTGGGACGGAAGTCGCTGTCGTTCTCAAAATCG

GTGGAGCTGCATGACAAAGTCATCGGGCATTATCTGAACATAAAACACTATCAATAAGTTGGAGTCATTA

CCCAATTATGATAGAATTTACAAGCTATAAGGTTATTGTCCTGGGTTTCAAGCATTAGTCCATGCAAGTT

TTTATGCTTTGCCCATTCTATAGATATATTGATAAGCGCGCTGCCTATGCCTTGCCCCCTGAAATCCTTA

CATACGGCGATATCTTCTATATAAAAGATATATTATCTTATCAGTATTGTCAATATATTCAAGGCAATCT

GCCTCCTCATCCTCTTCATCCTCTTCGTCTTGGTAGCTTTTTAAATATGGCGCTTCATAGAGTAATTCTG

TAAAGGTCCAATTCTCGTTTTCATACCTCGGTATAATCTTACCTATCACCTCAAATGGTTCGCTGGGTTT

ATCGCACCCCCGAACACGAGCACGGCACCCGCGACCACTATGCCAAGAATGCCCAAGGTAAAAATTGCCG

GCCCCGCCATGAAGTCCGTGAATGCCCCGACGGCCGAAGTGAAGGGCAGGCCGCCACCCAGGCCGCCGCC

CTCACTGCCCGGCACCTGGTCGCTGAATGTCGATGCCAGCACCTGCGGCACGTCAATGCTTCCGGGCGTC

GCGCTCGGGCTGATCGCCCATCCCGTTACTGCCCCGATCCCGGCAATGGCAAGGACTGCCAGCGCTGCCA

TTTTTGGGGTGAGGCCGTTCGCGGCCGAGGGGCGCAGCCCCTGGGGGGATGGGAGGCCCGCGTTAGCGGG

CCGGGAGGGTTCGAGAAGGGGGGGCACCCCCCTTCGGCGTGCGCGGTCACGCGCACAGGGCGCAGCCCTG

GTTAAAAACAAGGTTTATAAATATTGGTTTAAAAGCAGGTTAAAAGACAGGTTAGCGGTGGCCGAAAAAC

GGGCGGAAACCCTTGCAAATGCTGGATTTTCTGCCTGTGGACAGCCCCTCAAATGTCAATAGGTGCGCCC

CTCATCTGTCAGCACTCTGCCCCTCAAGTGTCAAGGATCGCGCCCCTCATCTGTCAGTAGTCGCGCCCCT

CAAGTGTCAATACCGCAGGGCACTTATCCCCAGGCTTGTCCACATCATCTGTGGGAAACTCGCGTAAAAT

CAGGCGTTTTCGCCGATTTGCGAGGCTGGCCAGCTCCACGTCGCCGGCCGAAATCGAGCCTGCCCCTCAT

CTGTCAACGCCGCGCCGGGTGAGTCGGCCCCTCAAGTGTCAACGTCCGCCCCTCATCTGTCAGTGAGGGC

CAAGTTTTCCGCGAGGTATCCACAACGCCGGCGGCCGCGGTGTCTCGCACACGGCTTCGACGGCGTTTCT

GGCGCGTTTGCAGGGCCATAGACGGCCGCCAGCCCAGCGGCGAGGGCAACCAGCCCGG

>gi|20454202|gb|AF502128.1| Transient expression vector pBI221, complete sequence

AAGCTTGCATGCCTGCAGGTCCCCAGATTAGCCTTTTCAATTTCAGAAAGAATGCTAACCCACAGATGGT

TAGAGAGGCTTACGCAGCAGGTCTCATCAAGACGATCTACCCGAGCAATAATCTCCAGGAAATCAAATAC

CTTCCCAAGAAGGTTAAAGATGCAGTCAAAAGATTCAGGACTAACTGCATCAAGAACACAGAGAAAGATA

TATTTCTCAAGATCAGAAGTACTATTCCAGTATGGACGATTCAAGGCTTGCTTCACAAACCAAGGCAAGT

AATAGAGATTGGAGTCTCTAAAAAGGTAGTTCCCACTGAATCAAAGGCCATGGAGTCAAAGATTCAAATA

GAGGACCTAACAGAACTCGCCGTAAAGACTGGCGAACAGTTCATACAGAGTCTCTTACGACTCAATGACA

AGAAGAAAATCTTCGTCAACATGGTGGAGCACGACACACTTGTCTACTCCAAAAATATCAAAGATACAGT

CTCAGAAGACCAAAGGGCAATTGAGACTTTTCAACAAAGGGTAATATCCGGAAACCTCCTCGGATTCCAT

TGCCCAGCTATCTGTCACTTTATTGTGAAGATAGTGGAAAAGGAAGGTGGCTCCTACAAATGCCATCATT

GCGATAAAGGAAAGGCCATCGTTGAAGATGCCTCTGCCGACAGTGGTCCCAAAGATGGACCCCCACCCAC

GAGGAGCATCGTGGAAAAAGAAGACGTTCCAACCACGTCTTCAAAGCAAGTGGATTGATGTGATATCTCC

ACTGACGTAAGGGATGACGCACAATCCCACTATCCTTCGCAAGACCCTTCCTCTATATAAGGAAGTTCAT

TTCATTTGGAGAGAACACGGGGGACTCTAGAGGATCCCCGGGTGGTCAGTCCCTTATGTTACGTCCTGTA

GAAACCCCAACCCGTGAAATCAAAAAACTCGACGGCCTGTGGGCATTCAGTCTGGATCGCGAAAACTGTG

GAATTGATCAGCGTTGGTGGGAAAGCGCGTTACAAGAAAGCCGGGCAATTGCTGTGCCAGGCAGTTTTAA

CGATCAGTTCGCCGATGCAGATATTCGTAATTATGCGGGCAACGTCTGGTATCAGCGCGAAGTCTTTATA

CCGAAAGGTTGGGCAGGCCAGCGTATCGTGCTGCGTTTCGATGCGGTCACTCATTACGGCAAAGTGTGGG

TCAATAATCAGGAAGTGATGGAGCATCAGGGCGGCTATACGCCATTTGAAGCCGATGTCACGCCGTATGT

TATTGCCGGGAAAAGTGTACGTATCACCGTTTGTGTGAACAACGAACTGAACTGGCAGACTATCCCGCCG

GGAATGGTGATTACCGACGAAAACGGCAAGAAAAAGCAGTCTTACTTCCATGATTTCTTTAACTATGCCG

GAATCCATCGCAGCGTAATGCTCTACACCACGCCGAACACCTGGGTGGACGATATCACCGTGGTGACGCA

TGTCGCGCAAGACTGTAACCACGCGTCTGTTGACTGGCAGGTGGTGGCCAATGGTGATGTCAGCGTTGAA

CTGCGTGATGCGGATCAACAGGTGGTTGCAACTGGACAAGGCACTAGCGGGACTTTGCAAGTGGTGAATC

CGCACCTCTGGCAACCGGGTGAAGGTTATCTCTATGAACTGTGCGTCACAGCCAAAAGCCAGACAGAGTG

TGATATCTACCCGCTTCGCGTCGGCATCCGGTCAGTGGCAGTGAAGGGCGAACAGTTCCTGATTAACCAC

AAACCGTTCTACTTTACTGGCTTTGGTCGTCATGAAGATGCGGACTTGCGTGGCAAAGGATTCGATAACG

TGCTGATGGTGCACGACCACGCATTAATGGACTGGATTGGGGCCAACTCCTACCGTACCTCGCATTACCC

TTACGCTGAAGAGATGCTCGACTGGGCAGATGAACATGGCATCGTGGTGATTGATGAAACTGCTGCTGTC

GGCTTTAACCTCTCTTTAGGCATTGGTTTCGAAGCGGGCAACAAGCCGAAAGAACTGTACAGCGAAGAGG

CAGTCAACGGGGAAACTCAGCAAGCGCACTTACAGGCGATTAAAGAGCTGATAGCGCGTGACAAAAACCA

CCCAAGCGTGGTGATGTGGAGTATTGCCAACGAACCGGATACCCGTCCGCAAGGTGCACGGGAATATTTC

GCGCCACTGGCGGAAGCAACGCGTAAACTCGACCCGACGCGTCCGATCACCTGCGTCAATGTAATGTTCT

GCGACGCTCACACCGATACCATCAGCGATCTCTTTGATGTGCTGTGCCTGAACCGTTATTACGGATGGTA

TGTCCAAAGCGGCGATTTGGAAACGGCAGAGAAGGTACTGGAAAAAGAACTTCTGGCCTGGCAGGAGAAA

CTGCATCAGCCGATTATCATCACCGAATACGGCGTGGATACGTTAGCCGGGCTGCACTCAATGTACACCG

ACATGTGGAGTGAAGAGTATCAGTGTGCATGGCTGGATATGTATCACCGCGTCTTTGATCGCGTCAGCGC

CGTCGTCGGTGAACAGGTATGGAATTTCGCCGATTTTGCGACCTCGCAAGGCATATTGCGCGTTGGCGGT

AACAAGAAAGGGATCTTCACTCGCGACCGCAAACCGAAGTCGGCGGCTTTTCTGCTGCAAAAACGCTGGA

CTGGCATGAACTTCGGTGAAAAACCGCAGCAGGGAGGCAAACAATGAATCAACAACTCTCCTGGCGCACC

ATCGTCGGCTACAGCCTCGGGAATTGCTACCGAGCTCGAATTTCCCCGATCGTTCAAACATTTGGCAATA

AAGTTTCTTAAGATTGAATCCTGTTGCCGGTCTTGCGATGATTATCATATAATTTCTGTTGAATTACGTT

AAGCATGTAATAATTAACATGTAATGCATGACGTTATTTATGAGATGGGTTTTTATGATTAGAGTCCCGC

AATTATACATTTAATACGCGATAGAAAACAAAATATAGCGCGCAAACTAGGATAAATTATCGCGCGCGGT

GTCATCTATGTTACTAGATCGGGAATTCACTGGCCGTCGTTTTACAACGTCGTGACTGGGAAAACCCTGG

CGTTACCCAACTTAATCGCCTTGCAGCACATCCCCCTTTCGCCAGCTGGCGTAATAGCGAAGAGGCCCGC

ACCGATCGCCCTTCCCAACAGTTGCGCAGCCTGAATGGCGAATGGCGCCTGATGCGGTATTTTCTCCTTA

CGCATCTGTGCGGTATTTCACACCGCATATGGTGCACTCTCAGTACAATCTGCTCTGATGCCGCATAGTT

AAGCCAGCCCCGACACCCGCCAACACCCGCTGACGCGCCCTGACGGGCTTGTCTGCTCCCGGCATCCGCT

TACAGACAAGCTGTGACCGTCTCCGGGAGCTGCATGTGTCAGAGGTTTTCACCGTCATCACCGAAACGCG

CGAGACGAAAGGGCCTCGTGATACGCCTATTTTTATAGGTTAATGTCATGATAATAATGGTTTCTTAGAC

GTCAGGTGGCACTTTTCGGGGAAATGTGCGCGGAACCCCTATTTGTTTATTTTTCTAAATACATTCAAAT

ATGTATCCGCTCATGAGACAATAACCCTGATAAATGCTTCAATAATATTGAAAAAGGAAGAGTATGAGTA

TTCAACATTTCCGTGTCGCCCTTATTCCCTTTTTTGCGGCATTTTGCCTTCCTGTTTTTGCTCACCCAGA

AACGCTGGTGAAAGTAAAAGATGCTGAAGATCAGTTGGGTGCACGAGTGGGTTACATCGAACTGGATCTC

AACAGCGGTAAGATCCTTGAGAGTTTTCGCCCCGAAGAACGTTTTCCAATGATGAGCACTTTTAAAGTTC

TGCTATGTGGCGCGGTATTATCCCGTATTGACGCCGGGCAAGAGCAACTCGGTCGCCGCATACACTATTC

TCAGAATGACTTGGTTGAGTACTCACCAGTCACAGAAAAGCATCTTACGGATGGCATGACAGTAAGAGAA

TTATGCAGTGCTGCCATAACCATGAGTGATAACACTGCGGCCAACTTACTTCTGACAACGATCGGAGGAC

CGAAGGAGCTAACCGCTTTTTTGCACAACATGGGGGATCATGTAACTCGCCTTGATCGTTGGGAACCGGA

GCTGAATGAAGCCATACCAAACGACGAGCGTGACACCACGATGCCTGTAGCAATGGCAACAACGTTGCGC

AAACTATTAACTGGCGAACTACTTACTCTAGCTTCCCGGCAACAATTAATAGACTGGATGGAGGCGGATA

AAGTTGCAGGACCACTTCTGCGCTCGGCCCTTCCGGCTGGCTGGTTTATTGCTGATAAATCTGGAGCCGG

TGAGCGTGGGTCTCGCGGTATCATTGCAGCACTGGGGCCAGATGGTAAGCCCTCCCGTATCGTAGTTATC

TACACGACGGGGAGTCAGGCAACTATGGATGAACGAAATAGACAGATCGCTGAGATAGGTGCCTCACTGA

TTAAGCATTGGTAACTGTCAGACCAAGTTTACTCATATATACTTTAGATTGATTTAAAACTTCATTTTTA

ATTTAAAAGGATCTAGGTGAAGATCCTTTTTGATAATCTCATGACCAAAATCCCTTAACGTGAGTTTTCG

TTCCACTGAGCGTCAGACCCCGTAGAAAAGATCAAAGGATCTTCTTGAGATCCTTTTTTTCTGCGCGTAA

TCTGCTGCTTGCAAACAAAAAAACCACCGCTACCAGCGGTGGTTTGTTTGCCGGATCAAGAGCTACCAAC

TCTTTTTCCGAAGGTAACTGGCTTCAGCAGAGCGCAGATACCAAATACTGTCCTTCTAGTGTAGCCGTAG

TTAGGCCACCACTTCAAGAACTCTGTAGCACCGCCTACATACCTCGCTCTGCTAATCCTGTTACCAGTGG

CTGCTGCCAGTGGCGATAAGTCGTGTCTTACCGGGTTGGACTCAAGACGATAGTTACCGGATAAGGCGCA

GCGGTCGGGCTGAACGGGGGGTTCGTGCACACAGCCCAGCTTGGAGCGAACGACCTACACCGAACTGAGA

TACCTACAGCGTGAGCTATGAGAAAGCGCCACGCTTCCCGAAGGGAGAAAGGCGGACAGGTATCCGGTAA

GCGGCAGGGTCGGAACAGGAGAGCGCACGAGGGAGCTTCCAGGGGGAAACGCCTGGTATCTTTATAGTCC

TGTCGGGTTTCGCCACCTCTGACTTGAGCGTCGATTTTTGTGATGCTCGTCAGGGGGGCGGAGCCTATGG

AAAAACGCCAGCAACGCGGCCTTTTTACGGTTCCTGGCCTTTTGCTGGCCTTTTGCTCACATGTTCTTTC

CTGCGTTATCCCCTGATTCTGTGGATAACCGTATTACCGCCTTTGAGTGAGCTGATACCGCTCGCCGCAG

CCGAACGACCGAGCGCAGCGAGTCAGTGAGCGAGGAAGCGGAAGAGCGCCCAATACGCAAACCGCCTCTC

CCCGCGCGTTGGCCGATTCATTAATGCAGCTGGCACGACAGGTTTCCCGACTGGAAAGCGGGCAGTGAGC

GCAACGCAATTAATGTGAGTTAGCTCACTCATTAGGCACCCCAGGCTTTACACTTTATGCTTCCGGCTCG

TATGTTGTGTGGAATTGTGAGCGGATAACAATTTCACACAGGAAACAGCTATGACCATGATTACGCC

>gi|2071944|gb|U84006.1|EVU84006 Expression vector pBSII-LUCINT firefly luciferase (LUCINT), beta-galactosidase (lacZ) and beta-lactamase (ampR) genes, complete cds and lac operon promoter sequence

GGTACCGGGCCCCCCCTCGACGGTATCGATAAGCTTGCATGCCTGCAGGTCTGCTGACCCACAGATGGTT

AGAGAGGCCTACGCGGCAGGTCTCATCAAGACGATCTACCCGAGTAATAATCTCCAGGAGATCAAATACC

TTCCCAAGAAGGTTAAAGATGCAGTCAAAAGATTCAGGACTAACTGCATCAAGAACACAGAGAAAGATAT

ATTTCTCAAGATCAGAAGTACTATTCCAGTATGGACGATTCAAGGCTTGCTTCATAAACCAAGGCAAGTA

ATAGAGATTGGAGTCTCTAAGAAAGTAGTTCCTACTGAATCAAAGGCCATGGAGTCAAAAATTCAGATCG

AGGATCTAACAGAACTCGCCGTGAAGACTGGCGAACAGTTCATACAGAGTCTTTTACGACTCAATGACAA

GAAGAAAATCTTCGTCAACATGGTGGAGCACGACACTCTCGTCTACTCCAAGAATATCAAAGATACAGTC

TCAGAAGACCAAAGGGCTATTGAGACTTTTCAACAAAGGGTAATATCGGGAAACCTCCTCGGATTCCATT

GCCCAGCTATCTGTCACTTCATCAAAAGGACAGTAGAAAAGGAAGGTGGCACCTACAAATGCCATCATTG

CGATAAAGGAAAGGCTATCGTTCAAGATGCCTCTGCCGACAGTGGTCCCAAAGATGGACCCCCACCCACG

AGGAGCATCGTGGAAAAAGAAGACGTTCCAACCACGTCTTCAAAGCAAGTGGATTGATGTGATATCTCCA

CTGACGTAAGGGATGACGCACAATCCCACTATCCTTCGCAAGACCCTTCCTCTATATAAGGAAGTTCATT

TCATTTGGAGAGACTCTAGTGGATCCCCCGTACCATGGAAGACGCCAAAAACATAAAGAAAGGCCCGGCG

CCATTCTATCCGCTAGAGGATGGAACCGCTGGAGAGCAACTGCATAAGGCTATGAAGAGATACGCCCTGG

TTCCTGGAACAATTGCTTTTACAGATGCACATATCGAGGTGAACATTACGTAAGTTTCTGCTTCTACCTT

TGATATATATATAATAATTATCATTAATTAGTAGTAATATAATATTTCAAATATTTTTTTCAAAATAAAA

GAATGTAGTATATAGCAATTGCTTTTCTGTAGTTTATAAGTGTGTATATTTTAATTTATAACTTTTCTAA

TATATGACCAAAATTTGTTGATGTGCAGGTACGCGGAATACTTCGAAATGTCCGTTCGGTTGGCAGAAGC

TATGAAACGATATGGGCTGAATACAAATCACAGAATCGTCGTATGCAGTGAAAACTCTCTTCAATTCTTT

ATGCCGGTGTTGGGCGCGTTATTTATCGGAGTTGCAGTTGCGCCCGCGAACGACATTTATAATGAACGTG

AATTGCTCAACAGTATGAACATTTCGCAGCCTACCGTAGTGTTTGTTTCCAAAAAGGGGTTGCAAAAAAT

TTTGAACGTGCAAAAAAAATTACCAATAATCCAGAAAATTATTATCATGGATTCTAAAACGGATTACCAG

GGATTTCAGTCGATGTACACGTTCGTCACATCTCATCTACCTCCCGGTTTTAATGAATACGATTTTGTAC

CAGAGTCCTTTGATCGTGACAAAACAATTGCACTGATAATGAACTCCTCTGGATCTACTGGGTTACCTAA

GGGTGTGGCCCTTCCGCATAGAACTGCCTGCGTCAGATTCTCGCATGCCAGAGATCCTATTTTTGGCAAT

CAAATCATTCCGGATACTGCGATTTTAAGTGTTGTTCCATTCCATCACGGTTTTGGAATGTTTACTACAC

TCGGATATTTGATATGTGGATTTCGAGTCGTCTTAATGTATAGATTTGAAGAAGAGCTGTTTTTACGATC

CCTTCAGGATTACAAAATTCAAAGTGCGTTGCTAGTACCAACCCTATTTTCATTCTTCGCCAAAAGCACT

CTGATTGACAAATACGATTTATCTAATTTACACGAAATTGCTTCTGGGGGCGCACCTCTTTCGAAAGAAG

TCGGGGAAGCGGTTGCAAAACGCTTCCATCTTCCAGGGATACGACAAGGATATGGGCTCACTGAGACTAC

ATCAGCTATTCTGATTACACCCGAGGGGGATGATAAACCGGGCGCGGTCGGTAAAGTTGTTCCATTTTTT

GAAGCGAAGGTTGTGGATCTGGATACCGGGAAAACGCTGGGCGTTAATCAGAGAGGCGAATTATGTGTCA

GAGGACCTATGATTATGTCCGGTTATGTAAACAATCCGGAAGCGACCAACGCCTTGATTGACAAGGATGG

ATGGCTACATTCTGGAGACATAGCTTACTGGGACGAAGACGAACACTTCTTCATAGTTGACCGCTTGAAG

TCTTTAATTAAATACAAAGGATACCAGGTGGCCCCCGCTGAATTGGAGTCGATATTGTTACAACACCCCA

ACATCTTCGACGCGGGCGTGGCAGGTCTTCCCGACGATGACGCCGGTGAACTTCCCGCCGCCGTTGTTGT

TTTGGAGCACGGAAAGACGATGACGGAAAAAGAGATCGTGGATTACGTCGCCAGTCAAGTAACAACCGCC

AAAAAGTTGCGCGGAGGAGTTGTGTTTGTGGACGAAGTACCGAAAGGTCTTACCGGAAAACTCGACGCAA

GAAAAATCAGAGAGATCCTCATAAAGGCCAAGAAGGGCGGAAAGTCCAAATTGTAAAATGTAACTGTATT

CAGCGATGACGAAATTCTTAGCTATTGTAATACTCTAGGGGCTGCAAGATCTGCTCGAATTTCCCCGATC

GTTCAAACATTTGGCAATAAAGTTTCTTAAGATTGAATCCTGTTGCCGGTCTTGCGATGATTATCATATA

ATTTCTGTTGAATTACGTTAAGCATGTAATAATTAACATGTAATGCATGACGTTATTTATGAGATGGGTT

TTTATGATTAGAGTCCCGCAATTATACATTTAATACGCGATAGAAAACAAAATATAGCGCGCAAACTAGG

ATAAATTATCGCGCGCGGTGTCATCTATGTTACTAGATCGGGAATTCCTGCAGCCCGGGGGATCCACTAG

TTCTAGAGCGGCCGCCACCGCGGTGGAGCTCCAATTCGCCCTATAGTGAGTCGTATTACAATTCACTGGC

CGTCGTTTTACAACGTCGTGACTGGGAAAACCCTGGCGTTACCCAACTTAATCGCCTTGCAGCACATCCC

CCTTTCGCCAGCTGGCGTAATAGCGAAGAGGCCCGCACCGATCGCCCTTCCCAACAGTTGCGCAGCCTGA

ATGGCGAATGGCGCGAAATTGTAAACGTTAATATTTTGTTAAAATTCGCGTTAAATTTTTGTTAAATCAG

CTCATTTTTTAACCAATAGGCCGAAATCGGCAAAATCCCTTATAAATCAAAAGAATAGACCGAGATAGGG

TTGAGTGTTGTTCCAGTTTGGAACAAGAGTCCACTATTAAAGAACGTGGACTCCAACGTCAAAGGGCGAA

AAACCGTCTATCAGGGCGATGGCCCACTACGTGAACCATCACCCTAATCAAGTTTTTTGGGGTCGAGGTG

CCGTAAAGCACTAAATCGGAACCCTAAAGGGAGCCCCCGATTTAGAGCTTGACGGGGAAAGCCGGCGAAC

GTGGCGAGAAAGGAAGGGAAGAAAGCGAAAGGAGCGGGCGCTAGGGCGCTGGCAAGTGTAGCGGTCACGC

TGCGCGTAACCACCACACCCGCCGCGCTTAATGCGCCGCTACAGGGCGCGTCCCAGGTGGCACTTTTCGG

GGAAATGTGCGCGGAACCCCTATTTGTTTATTTTTCTAAATACATTCAAATATGTATCCGCTCATGAGAC

AATAACCCTGATAAATGCTTCAATAATATTGAAAAAGGAAGAGTATGAGTATTCAACATTTCCGTGTCGC

CCTTATTCCCTTTTTTGCGGCATTTTGCCTTCCTGTTTTTGCTCACCCAGAAACGCTGGTGAAAGTAAAA

GATGCTGAAGATCAGTTGGGTGCACGAGTGGGTTACATCGAACTGGATCTCAACAGCGGTAAGATCCTTG

AGAGTTTTCGCCCCGAAGAACGTTTTCCAATGATGAGCACTTTTAAAGTTCTGCTATGTGGCGCGGTATT

ATCCCGTATTGACGCCGGGCAAGAGCAACTCGGTCGCCGCATACACTATTCTCAGAATGACTTGGTTGAG

TACTCACCAGTCACAGAAAAGCATCTTACGGATGGCATGACAGTAAGAGAATTATGCAGTGCTGCCATAA

CCATGAGTGATAACACTGCGGCCAACTTACTTCTGACAACGATCGGAGGACCGAAGGAGCTAACCGCTTT

TTTGCACAACATGGGGGATCATGTAACTCGCCTTGATCGTTGGGAACCGGAGCTGAATGAAGCCATACCA

AACGACGAGCGTGACACCACGATGCCTGTAGCAATGGCAACAACGTTGCGCAAACTATTAACTGGCGAAC

TACTTACTCTAGCTTCCCGGCAACAATTAATAGACTGGATGGAGGCGGATAAAGTTGCAGGACCACTTCT

GCGCTCGGCCCTTCCGGCTGGCTGGTTTATTGCTGATAAATCTGGAGCCGGTGAGCGTGGGTCTCGCGGT

ATCATTGCAGCACTGGGGCCAGATGGTAAGCCCTCCCGTATCGTAGTTATCTACACGACGGGGAGTCAGG

CAACTATGGATGAACGAAATAGACAGATCGCTGAGATAGGTGCCTCACTGATTAAGCATTGGTAACTGTC

AGACCAAGTTTACTCATATATACTTTAGATTGATTTAAAACTTCATTTTTAATTTAAAAGGATCTAGGTG

AAGATCCTTTTTGATAATCTCATGACCAAAATCCCTTAACGTGAGTTTTCGTTCCACTGAGCGTCAGACC

CCGTAGAAAAGATCAAAGGATCTTCTTGAGATCCTTTTTTTCTGCGCGTAATCTGCTGCTTGCAAACAAA

AAAACCACCGCTACCAGCGGTGGTTTGTTTGCCGGATCAAGAGCTACCAACTCTTTTTCCGAAGGTAACT

GGCTTCAGCAGAGCGCAGATACCAAATACTGTCCTTCTAGTGTAGCCGTAGTTAGGCCACCACTTCAAGA

ACTCTGTAGCACCGCCTACATACCTCGCTCTGCTAATCCTGTTACCAGTGGCTGCTGCCAGTGGCGATAA

GTCGTGTCTTACCGGGTTGGACTCAAGACGATAGTTACCGGATAAGGCGCAGCGGTCGGGCTGAACGGGG

GGTTCGTGCACACAGCCCAGCTTGGAGCGAACGACCTACACCGAACTGAGATACCTACAGCGTGAGCTAT

GAGAAAGCGCCACGCTTCCCGAAGGGAGAAAGGCGGACAGGTATCCGGTAAGCGGCAGGGTCGGAACAGG

AGAGCGCACGAGGGAGCTTCCAGGGGGAAACGCCTGGTATCTTTATAGTCCTGTCGGGTTTCGCCACCTC

TGACTTGAGCGTCGATTTTTGTGATGCTCGTCAGGGGGGCGGAGCCTATGGAAAAACGCCAGCAACGCGG

CCTTTTTACGGTTCCTGGCCTTTTGCTGGCCTTTTGCTCACATGTTCTTTCCTGCGTTATCCCCTGATTC

TGTGGATAACCGTATTACCGCCTTTGAGTGAGCTGATACCGCTCGCCGCAGCCGAACGACCGAGCGCAGC

GAGTCAGTGAGCGAGGAAGCGGAAGAGCGCCCAATACGCAAACCGCCTCTCCCCGCGCGTTGGCCGATTC

ATTAATGCAGCTGGCACGACAGGTTTCCCGACTGGAAAGCGGGCAGTGAGCGCAACGCAATTAATGTGAG

TTAGCTCACTCATTAGGCACCCCAGGCTTTACACTTTATGCTTCCGGCTCGTATGTTGTGTGGAATTGTG

AGCGGATAACAATTTCACACAGGAAACAGCTATGACCATGATTACGCCAAGCTCGGAATTAACCCTCACT

AAAGGGAACAAAAGCTG

>gi|209228|gb|M61152.1|SYNPWB146 Artificial T-DNA conferring expression of a luciferase/kanamycin-resistance fusion to transgenic plants

GAATTCGAGGATTTTTCGGCGCTGCGCTACGTCCGCGACCGCGTTGAGGGATCAAGCCACAGCAGCCCAC

TCGACCTTCTNNCCGACCCAGACGAGCCAAGGGATCTTTTTGGAATGCTGCTCCGTCGTCAGGCTTTCCG

ACGTTTGGGTGGTTGAACAGAAGTCATTATCGCACGGAATGCCAAGCACTCCCGAGGGGAACCCTGTGGT

TGGCNTGCACATACAAATGGACGAACGGATAAACCTTTTCACGCCCTTTTAAATATCCGATTATTCTAAT

AAACGCTCTTTTCTCTTAGGTTTACCCGCCAATATATCCTGTCAAACACTGATAGTTTAAACTGAAGGCG

GGAAACGACAATCTGATCATGAGCGGAGAATTAAGGGAGTCACGTTATGACCCCCGCCGATGACGCGGGA

CAAGCCGTTTTACGTTTGGAACTGACAGAACCGCAACGTTGAAGGAGCCACTCAGCCGCGGGTTTCTGGA

GTTTAATGAGCTAAGGGTACCCTAAGCACATACGTCAGAAACCATTATTGCGCGTTCAAAAGTCGCCTAA

GGTCACTATCAGCTAGCAAATATTTCTTGTCAAAAATGCTCCACTGACGTTCCATAAATTCCCCTCGGTA

TCCAATTAGAGTCTCAGATCTCCTTTGCCCCGGAGATCACCATGGACGACTTTCTCTATCTCTACGATCT

AGGAAGAAAGTTCGACGGAGAAGGTGACGATACCATGTTCACCACCGATAATGAGAAGATTAGCCTCTTC

AATTTCAGAAAGAATGCTGACCCACAGATGGTTAGAGAGGCCTACGCGGCAGGTCTCATCAAGACGATCT

ACCCGAGTAATAATCTCCAGGAGATCAAATACCTTCCCAAGAAGGTTAAAGATGCAGTCAAAAGATTCAG

GACTAACTGCATCAAGAACACAGAGAAAGATATATTTCTCAAGATCAGAAGTACTATTCCAGTATGGACG

ATTCAAGGCTTGCTTCATAAACCAAGGCAAGTAATAGAGATTGGAGTCTCTAAGAAAGTAGTTCCTACTG

AATCAAAGGCCATGGAGTCAAAAATTCAGATCGAGGATCTAACAGAACTCGCCGTGAAGACTGGCGAACA

GTTCATACAGAGTCTTTTACGACTCAATGACAAGAAGAAAATCTTCGTCAACATGGTGGAGCACGACACT

CTCGTCTACTCCAAGAATATCAAAGATACAGTCTCAGAAGACCAAAGGGCTATTGAGACTTTTCAACAAA

GGGTAATATCGGGAAACCTCCTCGGATTCCATTGCCCAGCTATCTGTCACTTCATCAAAAGGACAGTAGA

AAAGGAAGGTGGCACCTACAAATGCCATCATTGCGATAAAGGAAAGGCTATCGTTCAAGATGCCTCTGCC

GACAGTGGTCCCAAAGATGGACCCCCACCCACGAGGAGCATCGTGGAAAAAGAAGACGTTCCAACCACGT

CTTCAAAGCAAGTGGATTGATGTGATATCTCCACTGACGTAAGGGATGACGCACAATCCCACTATCCTTC

GCAAGACCCTTCCTCTATATAAGGAAGTTCATTTCATTTGGAGAGGACACGCTGAAATCACCAGTCTCTC

TCTACAAATCTATCTCTCTCTAGATCTCTCTCAATCCAAATAATCTGCAATGGCAATTACCGGATCTTGG

AGGTTACTTATGGATCCCGCCAAAAACATAAAGAAAGGCCCGGCGCCATTCTATCCTCTAGAGGATGGAA

CCGCTGGAGAGCAACTGCATAAGGCTATGAAGAGATACGCCCTGGTTCCTGGAACAATTGCTTTTACAGA

TGCACATATCGAGGTGAACATCACGTACGCGGAATACTTCGAAATGTCCGTTCGGTTGGCAGAAGCTATG

AAACGATATGGGCTGAATACAAATCACAGAATCGTCGTATGCAGTGAAAACTCTCTTCAATTCTTTATGC

CGGTGTTGGGCGCGTTATTTATCGGAGTTGCAGTTGCGCCCGCGAACGACATTTATAATGAACGTGAATT

GCTCAACAGTATGAACATTTCGCAGCCTACCGTAGTGTTTGTTTCCAAAAAGGGGTTGCAAAAAATTTTG

AACGTGCAAAAAAAATTACCAATAATCCAGAAAATTATTATCATGGATTCTAAAACGGATTACCAGGGAT

TTCAGTCGATGTACACGTTCGTCACATCTCATCTACCTCCCGGTTTTAATGAATACGATTTTGTACCAGA

GTCCTTTGATCGTGACAAAACAATTGCACTGATAATGAATTCCTCTGGATCTACTGGGTTACCTAAGGGT

GTGGCCCTTCCGCATAGAACTGCCTGCGTCAGATTCTCGCATGCCAGAGATCCTATTTTTGGCAATCAAA

TCATTCCGGATACTGCGATTTTAAGTGTTGTTCCATTCCATCACGGTTTTGGAATGTTTACTACACTCGG

ATATTTGATATGTGGATTTCGAGTCGTCTTAATGTATAGATTTGAAGAAGAGCTGTTTTTACGATCCCTT

CAGGATTACAAAATTCAAAGTGCGTTGCTAGTACCAACCCTATTTTCATTCTTCGCCAAAAGCACTCTGA

TTGACAAATACGATTTATCTAATTTACACGAAATTGCTTCTGGGGGCGCACCTCTTTCGAAAGAAGTCGG

GGAAGCGGTTGCAAAACGCTTCCATCTTCCAGGGATACGACAAGGATATGGGCTCACTGAGACTACATCA

GCTATTCTGATTACACCCGAGGGGGATGATAAACCGGGCGCGGTCGGTAAAGTTGTTCCATTTTTTGAAG

CGAAGGTTGTGGATCTGGATACCGGGAAAACGCTGGGCGTTAATCAGAGAGGCGAATTATGTGTCAGAGG

ACCTATGATTATGTCCGGTTATGTAAACAATCCGGAAGCGACCAACGCCTTGATTGACAAGGATGGATGG

CTACATTCTGGAGACATAGCTTACTGGGACGAAGACGAACACTTCTTCATAGTTGACCGCTTGAAGTCTT

TAATTAAATACAAAGGATATCAGGTGGCCCCCGCTGAATTGGAATCGATATTGTTACAACACCCCAACAT

CTTCGACGCGGGCGTGGCAGGTCTTCCCGACGATGACGCCGGTGAACTTCCCGCCGCCGTTGTTGTTTTG

GAGCACGGAAAGACGATGACGGAAAAAGAGATCGTGGATTACGTCGCCAGTCAAGTAACAACCGCGAAAA

AGTTGCGCGGAGGAGTTGTGTTTGTGGACGAAGTACCGAAAGGTCTTACCGGAAAACTCGACGCAAGAAA

AATCAGAGAGATCCTCATAAAGGCCAAGAAGGGCGGAAAGTCCAAATTCTCGAGGGGATTGCACGCAGGT

TCTCCGGCCGCTTGGGTGGAGAGGCTATTCGGCTATGACTGGGCACAACAGACAATCGGCTGCTCTGATG

CCGCCGTGTTCCGGCTGTCAGCGCAGGGGCGCCCGGTTCTTTTTGTCAAGACCGACCTGTCCGGTGCCCT

GAATGAACTGCAGGACGAGGCAGCGCGGCTATCGTGGCTGGCCACGACGGGCGTTCCTTGCGCAGCTGTG

CTCGACGTTGTCACTGAAGCGGGAAGGGACTGGCTGCTATTGGGCGAAGTGCCGGGGCAGGATCTCCTGT

CATCTCACCTTGCTCCTGCCGAGAAAGTATCCATCATGGCTGATGCAATGCGGCGGCTGCATACGCTTGA

TCCGGCTACCTGCCCATTCGACCACCAAGCGAAACATCGCATCGAGCGAGCACGTACTCGGATGGAAGCC

GGTCTTGTCGATCAGGATGATCTGGACGAAGAGCATCAGGGGCTCGCGCCAGCCGAACTGTTCGCCAGGC

TCAAGGCGCGCATGCCCGACGGCGAGGATCTCGTCGTGACCCATGGCGATGCCTGCTTGCCGAATATCAT

GGTGGAAAATGGCCGCTTTTCTGGATTCATCGACTGTGGCCGGCTGGGTGTGGCGGACCGCTATCAGGAC

ATAGCGTTGGCTACCCGTGATATTGCTGAAGAGCTTGGCGGCGAATGGGCTGACCGCTTCCTCGTGCTTT

ACGGTATCGCCGCTCCCGATTCGCAGCGCATCGCCTTCTATCGCCTTCTTGACGAGTTCTTCTGAAGCTT

GCTAAGGAAATAATTGAGAAGGAAAATCCATCCATAACTAATATTCCAATATTATTGAGTGGTTCTCCAA

TTACATTAGATTATCTATGTGATCGAGTTCGTCTTTTTGATAACATCTTGGGCTTTGTTGTACAAATGCC

TGTGGTGACTTAATTAATGGACCTTATTATTGAAGTAATTAAGCAGCCACAATATGTCAAAAAATAATAA

TTAGGGTTCATGTATGTTCATTATAATGCATCTCTTCATGTACTCTTACTATATATAATGGAAAAAATAA

GTGTGGCTTAATTACATGTACTCCTCTCCTTAATTAATTCCTTTTTACATGTGGTACTACGTTATTTTGA

TTATTTTAATTTATTTGTCTGATTTTGACTATAACATCTTTGGACATATATTTAATTAATGTGCGAGAGC

TAAGATTTGAATTGTAATTGACAAATTAGTCCCCCAACCCCACTACACCCCAAAAATAAAAATAAAACGT

GACAATGTGTTATAATTTTTTGGAATAAATTAAGAAGGTTAAGGGATCAAGTAAACAAAAAATATGTGTG

GAAAGATAAATATGTATTTATCTTTAATTAGAAGTTTTGAATTTGAACATAAATATATATAAAAAAAGGT

TATCTATTAATTGATCTACTCTAAAGTGGATATTGAATATCAAATAAAAAACTAAAAAAAAGTAAAGCAT

AACAAGATCTCTAGAAGCTTCCGTCGTCGAAGCAGATCGTTCAAACATTTGGCAATAAAGTTTCTTAAGA

TTGAATCCTGTTGCCGGTCTTGCGATGATTATCATATAATTTCTGTTGAATTACGTTAAGCATGTAATAA

TTAACATGTAATGCATGACGTTATTTATGAGATGGGTTTTTATGATTAGAGTCCCGCAATTATACATTTA

ATACGCGATAGAAAACAAAATATAGCGCGCAAACTAGGATAAATTATCGCGCGCGGTGTCATCTATGTTA

CTAGATCGATCAAACTTCGGTACTGTGTAATGACGATGAGCAATCGAGAGGCTGACTAACAAAAGGTATG

CCCAAAAACAACCTCTCCAAACTGTTTCGAATTGGAAGTTTCTGCTCATGCCGACAGGCATAACTTAGAT

ATTCGCGGGCTATTCCCACTAATTCGTCCTGCTGGTTTGCGCCAAGATAAATCAGTGCATCTCCTTACAA

GTTCCTCTGTCTTGTGAAATGAACTGCTGACTGCCCCCCAAGAAAGCCTCCTCATCTCCCAGTTGGCGGC

GGCTGATACACCATCGAAAACCCACGTCCGAACACTTGATACATGTGCCTGAGAAATAGGCCTACGTCCA

AGAGCAAGTCCTTTCTGTGCTCGTCGGAAATTCCTCTCCTGTCAGACGGTCGTGCGCATGTCTTGCGTTG

ATGAAGCTAGCTTGCTGGCGTAATAGCGAAGAGGCCCGCACCGGAATTCNNNNNNNNNNNNNNNNNNNNN

NNNNNNNNNNNNNTTTAAANNNNNNNNNNTGATCANNNNNNNNNNTGATCANNNNNNNNNNCGATCGNNN

NNNNNNNCAGCTGNNNNNNNNNNCCTNNNNNAGGNNNNNNNNNNGTCGACNNNNNNNNNNCCCGGGNNNN

NNNCCCGGGNNNNNNNNNNAGGCCTNNNNNNNNNNGAANNNNTTCNNNNNNNNNNGCGGCCGCNNNNNNN

NNNGCGGCCGCNNNNNNNNNNGCGGCCGCNNNNNNNNNNGCCNNNNNGGCNNNNNNNNNNGCCNNNNNGG

CNNNNNNNNNNGCCNNNNNGGCNNNNNNNNNNGCCNNNNNGGCNNNNNNNNNNGCCNNNNNGGCNNNNNN

NNNNCCGCGGNNNNNNNNNNCCGCGGNNNNNNNNNNCCGCGGNNNNNNNNNNAATATTNNNNNNNNNNAA

TATTNNNNNNNNNNAATATTNNNNNNNNNNAATATTNNNNNNNNNNAATATTNNNNNNNNNN

>gi|21389151|gb|AF406991.1| Tobacco rattle virus RNA2-based VIGS vector pTRV2, complete sequence

ATAAAACATTGCACCTATGGTGTTGCCCTGGCTGGGGTATGTCAGTGATCGCAGTAGAATGTACTAATTG

ACAAGTTGGAGAATACGGTAGAACGTCCTTATCCAACACAGCCTTTATCCCTCTCCCTGACGAGGTTTTT

GTCAGTGTAATATTTCTTTTTGAACTATCCAGCTTAGTACCGTACGGGAAAGTGACTGGTGTGCTTATCT

TTGAAATGTTACTTTGGGTTTCGGTTCTTTAGGTTAGTAAGAAAGCACTTGTCTTCTCATACAAAGGAAA

ACCTGAGACGTATCGCTTACGAAAGTAGCAATGAAAGAAAGGTGGTGGTTTTAATCGCTACCGCAAAAAC

GATGGGGTCGTTTTAATTAACTTCTCCTACGCAAGCGTCTAAACGGACGTTGGGGTTTTGCTAGTTTCTT

TAGAGAAAACTAGCTAAGTCTTTAATGTTATCATTAGAGATGGCATAAATATAATACTTGTGTCTGCTGA

TAAGATCATTTTAATTTGGACGATTAGACTTGTTGAACTACAGGTTACTGAATCACTTGCGCTAATCAAC

ATGGGAGATATGTACGATGAATCATTTGACAAGTCGGGCGGTCCTGCTGACTTGATGGACGATTCTTGGG

TGGAATCAGTTTCGTGGAAAGATCTGTTGAAGAAGTTACACAGCATAAAATTTGCACTACAGTCTGGTAG

AGATGAGATCACTGGGTTACTAGCGGCACTGAATAGACAGTGTCCTTATTCACCATATGAGCAGTTTCCA

GATAAGAAGGTGTATTTCCTTTTAGACTCACGGGCTAACAGTGCTCTTGGTGTGATTCAGAACGCTTCAG

CGTTCAAGAGACGAGCTGATGAGAAGAATGCAGTGGCGGGTGTTACAAATATTCCTGCGAATCCAAACAC

AACGGTTACGACGAACCAAGGGAGTACTACTACTACCAAGGCGAACACTGGCTCGACTTTGGAAGAAGAC

TTGTACACTTATTACAAATTCGATGATGCCTCTACAGCTTTCCACAAATCTCTAACTTCGTTAGAGAACA

TGGAGTTGAAGAGTTATTACCGAAGGAACTTTGAGAAAGTATTCGGGATTAAGTTTGGTGGAGCAGCTGC

TAGTTCATCTGCACCGCCTCCAGCGAGTGGAGGTCCGATACGTCCTAATCCCTAGGGATTTAAGGACGTG

AACTCTGTTGAGATCTCTGTGAAATTCAGAGGGTGGGTGATACCATATTCACTGATGCCATTAGCGACAT

CTAAATAGGGCTAATTGTGACTAATTTGAGGGAATTTCCTTTACCATTGACGTCAGTGTCGTTGGTAGCA

TTTGAGTTTCGCAATGCACGAATTACTTAGGAAGTGGCTTGACGACACTAATGTGTTATTGTTAGATAAT

GGTTTGGTGGTCAAGGTACGTAGTAGAGTCCCACATATTCGCACGTATGAAGTAATTGGAAAGTTGTCAG

TTTTTGATAATTCACTGGGAGATGATACGCTGTTTGAGGGAAAAGTAGAGAACGTATTTGTTTTTATGTT

CAGGCGGTTCTTGTGTGTCAACAAAGATGGACATTGTTACTCAAGGAAGCACGATGAGCTTTATTATTAC

GGACGAGTGGACTTAGATTCTGTGAGTAAGGTTACCGAATTCTCTAGAAGGCCTCCATGGGGATCCGGTA

CCGAGCTCACGCGTCTCGAGGCCCGGGCATGTCCCGAAGACATTAAACTACGGTTCTTTAAGTAGATCCG

TGTCTGAAGTTTTAGGTTCAATTTAAACCTACGAGATTGACATTCTCGACTGATCTTGATTGATCGGTAA

GTCTTTTGTAATTTAATTTTCTTTTTGATTTTATTTTAAATTGTTATCTGTTTCTGTGTATAGACTGTTT

GAGATCGGCGTTTGGCCGACTCATTGTCTTACCATAGGGGAACGGACTTTGTTTGTGTTGTTATTTTATT

TGTATTTTATTAAAATTCTCAACGATCTGAAAAAGCCTCGCGGCTAAGAGATTGTTGGGGGGTGAGTAAG

TACTTTTAAAGTGATGATGGTTACAAAGGCAAAAGGGGTAAAACCCCTCGCCTACGTAAGCGTTATTACG

CCCGTCTGTACTTATATCAGTACACTGACGAGTCCCTAAAGGACGAAACGGGAGAACGCTAGCCACCACC

ACCACCACCACGTGTGAATTACAGGTGACCAGCTCGAATTTCCCCGATCGTTCAAACATTTGGCAATAAA

GTTTCTTAAGATTGAATCCTGTTGCCGGTCTTGCGATGATTATCATATAATTTCTGTTGAATTACGTTAA

GCATGTAATAATTAACATGTAATGCATGACGTTATTTATGAGATGGGTTTTTATGATTAGAGTCCCGCAA

TTATACATTTAATACGCGATAGAAAACAAAATATAGCGCGCAAACTAGGATAAATTATCGCGCGCGGTGT

CATCTATGTTACTAGATCGGGAATTAAACTATCAGTGTTTGACAGGATATATTGGCGGGTAAACCTAAGA

GAAAAGAGCGTTTATTAGAATAACGGATATTTAAAAGGGCGTGAAAAGGTTTATCCGTTCGTCCATTTGT

ATGTGCATGCCAACCACAGGGTTCCCCTCGGGATCAAAGTACTTTGATCCAACCCCTCCGCTGCTATAGT

GCAGTCGGCTTCTGACGTTCAGTGCAGCCGTCTTCTGAAAACGACATGTCGCACAAGTCCTAAGTTACGC

GACAGGCTGCCGCCCTGCCCTTTTCCTGGCGTTTTCTTGTCGCGTGTTTTAGTCGCATAAAGTAGAATAC

TTGCGACTAGAACCGGAGACATTACGCCATGAACAAGAGCGCCGCCGCTGGCCTGCTGGGCTATGCCCGC

GTCAGCACCGACGACCAGGACTTGACCAACCAACGGGCCGAACTGCACGCGGCCGGCTGCACCAAGCTGT

TTTCCGAGAAGATCACCGGCACCAGGCGCGACCGCCCGGAGCTGGCCAGGATGCTTGACCACCTACGCCC

TGGCGACGTTGTGACAGTGACCAGGCTAGACCGCCTGGCCCGCAGCACCCGCGACCTACTGGACATTGCC

GAGCGCATCCAGGAGGCCGGCGCGGGCCTGCGTAGCCTGGCAGAGCCGTGGGCCGACACCACCACGCCGG

CCGGCCGCATGGTGTTGACCGTGTTCGCCGGCATTGCCGAGTTCGAGCGTTCCCTAATCATCGACCGCAC

CCGGAGCGGGCGCGAGGCCGCCAAGGCCCGAGGCGTGAAGTTTGGCCCCCGCCCTACCCTCACCCCGGCA

CAGATCGCGCACGCCCGCGAGCTGATCGACCAGGAAGGCCGCACCGTGAAAGAGGCGGCTGCACTGCTTG

GCGTGCATCGCTCGACCCTGTACCGCGCACTTGAGCGCAGCGAGGAAGTGACGCCCACCGAGGCCAGGCG

GCGCGGTGCCTTCCGTGAGGACGCATTGACCGAGGCCGACGCCCTGGCGGCCGCCGAGAATGAACGCCAA

GAGGAACAAGCATGAAACCGCACCAGGACGGCCAGGACGAACCGTTTTTCATTACCGAAGAGATCGAGGC

GGAGATGATCGCGGCCGGGTACGTGTTCGAGCCGCCCGCGCACGTCTCAACCGTGCGGCTGCATGAAATC

CTGGCCGGTTTGTCTGATGCCAAGCTGGCGGCCTGGCCGGCCAGCTTGGCCGCTGAAGAAACCGAGCGCC

GCCGTCTAAAAAGGTGATGTGTATTTGAGTAAAACAGCTTGCGTCATGCGGTCGCTGCGTATATGATGCG

ATGAGTAAATAAACAAATACGCAAGGGGAACGCATGAAGGTTATCGCTGTACTTAACCAGAAAGGCGGGT

CAGGCAAGACGACCATCGCAACCCATCTAGCCCGCGCCCTGCAACTCGCCGGGGCCGATGTTCTGTTAGT

CGATTCCGATCCCCAGGGCAGTGCCCGCGATTGGGCGGCCGTGCGGGAAGATCAACCGCTAACCGTTGTC

GGCATCGACCGCCCGACGATTGACCGCGACGTGAAGGCCATCGGCCGGCGCGACTTCGTAGTGATCGACG

GAGCGCCCCAGGCGGCGGACTTGGCTGTGTCCGCGATCAAGGCAGCCGACTTCGTGCTGATTCCGGTGCA

GCCAAGCCCTTACGACATATGGGCCACCGCCGACCTGGTGGAGCTGGTTAAGCAGCGCATTGAGGTCACG

GATGGAAGGCTACAAGCGGCCTTTGTCGTGTCGCGGGCGATCAAAGGCACGCGCATCGGCGGTGAGGTTG

CCGAGGCGCTGGCCGGGTACGAGCTGCCCATTCTTGAGTCCCGTATCACGCAGCGCGTGAGCTACCCAGG

CACTGCCGCCGCCGGCACAACCGTTCTTGAATCAGAACCCGAGGGCGACGCTGCCCGCGAGGTCCAGGCG

CTGGCCGCTGAAATTAAATCAAAACTCATTTGAGTTAATGAGGTAAAGAGAAAATGAGCAAAAGCACAAA

CACGCTAAGTGCCGGCCGTCCGAGCGCACGCAGCAGCAAGGCTGCAACGTTGGCCAGCCTGGCAGACACG

CCAGCCATGAAGCGGGTCAACTTTCAGTTGCCGGCGGAGGATCACACCAAGCTGAAGATGTACGCGGTAC

GCCAAGGCAAGACCATTACCGAGCTGCTATCTGAATACATCGCGCAGCTACCAGAGTAAATGAGCAAATG

AATAAATGAGTAGATGAATTTTAGCGGCTAAAGGAGGCGGCATGGAAAATCAAGAACAACCAGGCACCGA

CGCCGTGGAATGCCCCATGTGTGGAGGAACGGGCGGTTGGCCAGGCGTAAGCGGCTGGGTTGTCTGCCGG

CCCTGCAATGGCACTGGAACCCCCAAGCCCGAGGAATCGGCGTGACGGTCGCAAACCATCCGGCCCGGTA

CAAATCGGCGCGGCGCTGGGTGATGACCTGGTGGAGAAGTTGAAGGCCGCGCAGGCCGCCCAGCGGCAAC

GCATCGAGGCAGAAGCACGCCCCGGTGAATCGTGGCAAGCGGCCGCTGATCGAATCCGCAAAGAATCCCG

GCAACCGCCGGCAGCCGGTGCGCCGTCGATTAGGAAGCCGCCCAAGGGCGACGAGCAACCAGATTTTTTC

GTTCCGATGCTCTATGACGTGGGCACCCGCGATAGTCGCAGCATCATGGACGTGGCCGTTTTCCGTCTGT

CGAAGCGTGACCGACGAGCTGGCGAGGTGATCCGCTACGAGCTTCCAGACGGGCACGTAGAGGTTTCCGC

AGGGCCGGCCGGCATGGCCAGTGTGTGGGATTACGACCTGGTACTGATGGCGGTTTCCCATCTAACCGAA

TCCATGAACCGATACCGGGAAGGGAAGGGAGACAAGCCCGGCCGCGTGTTCCGTCCACACGTTGCGGACG

TACTCAAGTTCTGCCGGCGAGCCGATGGCGGAAAGCAGAAAGACGACCTGGTAGAAACCTGCATTCGGTT

AAACACCACGCACGTTGCCATGCAGCGTACGAAGAAGGCCAAGAACGGCCGCCTGGTGACGGTATCCGAG

GGTGAAGCCTTGATTAGCCGCTACAAGATCGTAAAGAGCGAAACCGGGCGGCCGGAGTACATCGAGATCG

AGCTAGCTGATTGGATGTACCGCGAGATCACAGAAGGCAAGAACCCGGACGTGCTGACGGTTCACCCCGA

TTACTTTTTGATCGATCCCGGCATCGGCCGTTTTCTCTACCGCCTGGCACGCCGCGCCGCAGGCAAGGCA

GAAGCCAGATGGTTGTTCAAGACGATCTACGAACGCAGTGGCAGCGCCGGAGAGTTCAAGAAGTTCTGTT

TCACCGTGCGCAAGCTGATCGGGTCAAATGACCTGCCGGAGTACGATTTGAAGGAGGAGGCGGGGCAGGC

TGGCCCGATCCTAGTCATGCGCTACCGCAACCTGATCGAGGGCGAAGCATCCGCCGGTTCCTAATGTACG

GAGCAGATGCTAGGGCAAATTGCCCTAGCAGGGGAAAAAGGTCGAAAAGGTCTCTTTCCTGTGGATAGCA

CGTACATTGGGAACCCAAAGCCGTACATTGGGAACCGGAACCCGTACATTGGGAACCCAAAGCCGTACAT

TGGGAACCGGTCACACATGTAAGTGACTGATATAAAAGAGAAAAAAGGCGATTTTTCCGCCTAAAACTCT

TTAAAACTTATTAAAACTCTTAAAACCCGCCTGGCCTGTGCATAACTGTCTGGCCAGCGCACAGCCGAAG

AGCTGCAAAAAGCGCCTACCCTTCGGTCGCTGCGCTCCCTACGCCCCGCCGCTTCGCGTCGGCCTATCGC

GGCCGCTGGCCGCTCAAAAATGGCTGGCCTACGGCCAGGCAATCTACCAGGGCGCGGACAAGCCGCGCCG

TCGCCACTCGACCGCCGGCGCCCACATCAAGGCACCCTGCCTCGCGCGTTTCGGTGATGACGGTGAAAAC

CTCTGACACATGCAGCTCCCGGAGACGGTCACAGCTTGTCTGTAAGCGGATGCCGGGAGCAGACAAGCCC

GTCAGGGCGCGTCAGCGGGTGTTGGCGGGTGTCGGGGCGCAGCCATGACCCAGTCACGTAGCGATAGCGG

AGTGTATACTGGCTTAACTATGCGGCATCAGAGCAGATTGTACTGAGAGTGCACCATATGCGGTGTGAAA

TACCGCACAGATGCGTAAGGAGAAAATACCGCATCAGGCGCTCTTCCGCTTCCTCGCTCACTGACTCGCT

GCGCTCGGTCGTTCGGCTGCGGCGAGCGGTATCAGCTCACTCAAAGGCGGTAATACGGTTATCCACAGAA

TCAGGGGATAACGCAGGAAAGAACATGTGAGCAAAAGGCCAGCAAAAGGCCAGGAACCGTAAAAAGGCCG

CGTTGCTGGCGTTTTTCCATAGGCTCCGCCCCCCTGACGAGCATCACAAAAATCGACGCTCAAGTCAGAG

GTGGCGAAACCCGACAGGACTATAAAGATACCAGGCGTTTCCCCCTGGAAGCTCCCTCGTGCGCTCTCCT

GTTCCGACCCTGCCGCTTACCGGATACCTGTCCGCCTTTCTCCCTTCGGGAAGCGTGGCGCTTTCTCATA

GCTCACGCTGTAGGTATCTCAGTTCGGTGTAGGTCGTTCGCTCCAAGCTGGGCTGTGTGCACGAACCCCC

CGTTCAGCCCGACCGCTGCGCCTTATCCGGTAACTATCGTCTTGAGTCCAACCCGGTAAGACACGACTTA

TCGCCACTGGCAGCAGCCACTGGTAACAGGATTAGCAGAGCGAGGTATGTAGGCGGTGCTACAGAGTTCT

TGAAGTGGTGGCCTAACTACGGCTACACTAGAAGGACAGTATTTGGTATCTGCGCTCTGCTGAAGCCAGT

TACCTTCGGAAAAAGAGTTGGTAGCTCTTGATCCGGCAAACAAACCACCGCTGGTAGCGGTGGTTTTTTT

GTTTGCAAGCAGCAGATTACGCGCAGAAAAAAAGGATCTCAAGAAGATCCTTTGATCTTTTCTACGGGGT

CTGACGCTCAGTGGAACGAAAACTCACGTTAAGGGATTTTGGTCATGCATTCTAGGTACTAAAACAATTC

ATCCAGTAAAATATAATATTTTATTTTCTCCCAATCAGGCTTGATCCCCAGTAAGTCAAAAAATAGCTCG

ACATACTGTTCTTCCCCGATATCCTCCCTGATCGACCGGACGCAGAAGGCAATGTCATACCACTTGTCCG

CCCTGCCGCTTCTCCCAAGATCAATAAAGCCACTTACTTTGCCATCTTTCACAAAGATGTTGCTGTCTCC

CAGGTCGCCGTGGGAAAAGACAAGTTCCTCTTCGGGCTTTTCCGTCTTTAAAAAATCATACAGCTCGCGC

GGATCTTTAAATGGAGTGTCTTCTTCCCAGTTTTCGCAATCCACATCGGCCAGATCGTTATTCAGTAAGT

AATCCAATTCGGCTAAGCGGCTGTCTAAGCTATTCGTATAGGGACAATCCGATATGTCGATGGAGTGAAA

GAGCCTGATGCACTCCGCATACAGCTCGATAATCTTTTCAGGGCTTTGTTCATCTTCATACTCTTCCGAG

CAAAGGACGCCATCGGCCTCACTCATGAGCAGATTGCTCCAGCCATCATGCCGTTCAAAGTGCAGGACCT

TTGGAACAGGCAGCTTTCCTTCCAGCCATAGCATCATGTCCTTTTCCCGTTCCACATCATAGGTGGTCCC

TTTATACCGGCTGTCCGTCATTTTTAAATATAGGTTTTCATTTTCTCCCACCAGCTTATATACCTTAGCA

GGAGACATTCCTTCCGTATCTTTTACGCAGCGGTATTTTTCGATCAGTTTTTTCAATTCCGGTGATATTC

TCATTTTAGCCATTTATTATTTCCTTCCTCTTTTCTACAGTATTTAAAGATACCCCAAGAAGCTAATTAT

AACAAGACGAACTCCAATTCACTGTTCCTTGCATTCTAAAACCTTAAATACCAGAAAACAGCTTTTTCAA

AGTTGTTTTCAAAGTTGGCGTATAACATAGTATCGACGGAGCCGATTTTGAAACCGCGGTGATCACAGGC

AGCAACGCTCTGTCATCGTTACAATCAACATGCTACCCTCCGCGAGATCATCCGTGTTTCAAACCCGGCA

GCTTAGTTGCCGTTCTTCCGAATAGCATCGGTAACATGAGCAAAGTCTGCCGCCTTACAACGGCTCTCCC

GCTGACGCCGTCCCGGACTGATGGGCTGCCTGTATCGAGTGGTGATTTTGTGCCGAGCTGCCGGTCGGGG

AGCTGTTGGCTGGCTGGTGGCAGGATATATTGTGGTGTAAACAAATTGACGCTTAGACAACTTAATAACA

CATTGCGGACGTTTTTAATGTACTGAATTAACGCCGAATTAATTCCTAGGCCACCATGTTGGGCCCGGCG

CGCCAAGCTTGCATGCCTGCAGGTCAACATGGTGGAGCACGACACTCTCGTCTACTCCAAGAATATCAAA

GATACAGTCTCAGAAGACCAGAGGGCTATTGAGACTTTTCAACAAAGGGTAATATCGGGAAACCTCCTCG

GATTCCATTGCCCAGCTATCTGTCACTTCATCGAAAGGACAGTAGAAAAGGAAGATGGCTTCTACAAATG

CCATCATTGCGATAAAGGAAAGGCTATCGTTCAAGATGCCTCTACCGACAGTGGTCCCAAAGATGGACCC

CCACCCACGAGGAACATCGTGGAAAAAGAAGACGTTCCAACCACGTCTTCAAAGCAAGTGGATTGATGTG

ATGGTCAACATGGTGGAGCACGACACTCTCGTCTACTCCAAGAATATCAAAGATACAGTCTCAGAAGACC

AGAGGGCTATTGAGACTTTTCAACAAAGGGTAATATCGGGAAACCTCCTCGGATTCCATTGCCCAGCTAT

CTGTCACTTCATCGAAAGGACAGTAGAAAAGGAAGATGGCTTCTACAAATGCCATCATTGCGATAAAGGA

AAGGCTATCGTTCAAGATGCCTCTACCGACAGTGGTCCCAAAGATGGACCCCCACCCACGAGGAACATCG

TGGAAAAAGAAGACGTTCCAACCACGTCTTCAAAGCAAGTGGATTGATGTGATATCTCCACTGACGTAAG

GGATGACGCACAATCCCACTATCCTTCGCAAGACCCTTCCTCTATATAAGGAAGTTCATTTCATTTGGAG

AGG

>gi|21552736|gb|AF408413.1| Binary vector pJawohl8-RNAi, complete sequence

CTCGAGAAAGAGGATCCACCTGAGGATCACAAGTTTGTACAAAAAAGCTGAACGAGAAACGTAAAATGAT

ATAAATATCAATATATTAAATTAGATTTTGCATAAAAAACAGACTACATAATACTGTAAAACACAACATA

TCCAGTCACTATGGCGGCCGCATTAGGCACCCCAGGCTTTACACTTTATGCTTCCGGCTCGTATAATGTG

TGGATTTTGAGTTAGGATCCGTCGAGATTTTCAGGAGCTAAGGAAGCTAAAATGGAGAAAAAAATCACTG

GATATACCACCGTTGATATATCCCAATGGCATCGTAAAGAACATTTTGAGGCATTTCAGTCAGTTGCTCA

ATGTACCTATAACCAGACCGTTCAGCTGGATATTACGGCCTTTTTAAAGACCGTAAAGAAAAATAAGCAC

AAGTTTTATCCGGCCTTTATTCACATTCTTGCCCGCCTGATGAATGCTCATCCGGAATTCCGTATGGCAA

TGAAAGACGGTGAGCTGGTGATATGGGATAGTGTTCACCCTTGTTACACCGTTTTCCATGAGCAAACTGA

AACGTTTTCATCGCTCTGGAGTGAATACCACGACGATTTCCGGCAGTTTCTACACATATATTCGCAAGAT

GTGGCGTGTTACGGTGAAAACCTGGCCTATTTCCCTAAAGGGTTTATTGAGAATATGTTTTTCGTCTCAG

CCAATCCCTGGGTGAGTTTCACCAGTTTTGATTTAAACGTGGCCAATATGGACAACTTCTTCGCCCCCGT

TTTCACCATGGGCAAATATTATACGCAAGGCGACAAGGTGCTGATGCCGCTGGCGATTCAGGTTCATCAT

GCCGTCTGTGATGGCTTCCATGTCGGCAGAATGCTTAATGAATTACAACAGTACTGCGATGAGTGGCAGG

GCGGGGCGTAAACGCGTGGATCCGGCTTACTAAAAGCCAGATAACAGTATGCGTATTTGCGCGCTGATTT

TTGCGGTATAAGAATATATACTGATATGTATACCCGAAGTATGTCAAAAAGAGGTGTGCTATGAAGCAGC

GTATTACAGTGACAGTTGACAGCGACAGCTATCAGTTGCTCAAGGCATATATGATGTCAATATCTCCGGT

CTGGTAAGCACAACCATGCAGAATGAAGCCCGTCGTCTGCGTGCCGAACGCTGGAAAGCGGAAAATCAGG

AAGGGATGGCTGAGGTCGCCCGGTTTATTGAAATGAACGGCTCTTTTGCTGACGAGAACAGGGACTGGTG

AAATGCAGTTTAAGGTTTACACCTATAAAAGAGAGAGCCGTTATCGTCTGTTTGTGGATGTACAGAGTGA

TATTATTGACACGCCCGGGCGACGGATGGTGATCCCCCTGGCCAGTGCACGTCTGCTGTCAGATAAAGTC

TCCCGTGAACTTTACCCGGTGGTGCATATCGGGGATGAAAGCTGGCGCATGATGACCACCGATATGGCCA

GTGTGCCGGTCTCCGTTATCGGGGAAGAAGTGGCTGATCTCAGCCACCGCGAAAATGACATCAAAAACGC

CATTAACCTGATGTTCTGGGGAATATAAATGTCAGGCTCCCTTATACACAGCCAGTCTGCAGGTCGACCA

TAGTGACTGGATATGTTGTGTTTTACAGTATTATGTAGTCTGTTTTTTATGCAAAATCTAATTTAATATA

TTGATATTTATATCATTTTACGTTTCTCGTTCAGCTTTCTTGTACAAAGTGGTGATCCTCCGTTCCATGG

GCTAGAAGCTTCTCCTCCTCTGCTAACGTAAGCCTCTCTGTTTTTTTTCTCTGTTTCTTTTGAAATGAAT

CCAATTAGTGATGATAATCTGTGTTTGATGTATCATTGATTTAACATCTTGACAATGAATCGTGATCGGA

AGTGATAAAGTTATGGGTCAACGGTTTCAAAGAGAGAGAAAGACTTTTAGAGTCAACTCTCGACTCTTTC

TTAATTATGTTATTGCTATTTGTCTCTTTTCTTGAAGTCTGAACAATTCTTGGGATTGTTTTGCAGGTTC

TAGCTTCTCCAACCACAGGAATTCATCGCCCGGGACTGGGTTATCACCACTTTGTACAAGAAAGCTGAAC

GAGAAACGTAAAATGATATAAATATCAATATATTAAATTAGATTTTGCATAAAAAACAGACTACATAATA

CTGTAAAACACAACATATCCAGTCACTATGGTCGACCTGCAGACTGGCTGTGTATAAGGGAGCCTGACAT

TTATATTCCCCAGAACATCAGGTTAATGGCGTTTTTGATGTCATTTTCGCGGTGGCTGAGATCAGCCACT

TCTTCCCCGATAACGGAGACCGGCACACTGGCCATATCGGTGGTCATCATGCGCCAGCTTTCATCCCCGA

TATGCACCACCGGGTAAAGTTCACGGGAGACTTTATCTGACAGCAGACGTGCACTGGCCAGGGGGATCAC

CATCCGTCGCCCGGGCGTGTCAATAATATCACTCTGTACATCCACAAACAGACGATAACGGCTCTCTCTT

TTATAGGTGTAAACCTTAAACTGCATTTCACCAGTCCCTGTTCTCGTCAGCAAAAGAGCCGTTCATTTCA

ATAAACCGGGCGACCTCAGCCATCCCTTCCTGATTTTCCGCTTTCCAGCGTTCGGCACGCAGACGACGGG

CTTCATTCTGCATGGTTGTGCTTACCAGACCGGAGATATTGACATCATATATGCCTTGAGCAACTGATAG

CTGTCGCTGTCAACTGTCACTGTAATACGCTGCTTCATAGCACACCTCTTTTTGACATACTTCGGGTATA

CATATCAGTATATATTCTTATACCGCAAAAATCAGCGCGCAAATACGCATACTGTTATCTGGCTTTTAGT

AAGCCGGATCCACGCGTTTACGCCCCGCCCTGCCACTCATCGCAGTACTGTTGTAATTCATTAAGCATTC

TGCCGACATGGAAGCCATCACAGACGGCATGATGAACCTGAATCGCCAGCGGCATCAGCACCTTGTCGCC

TTGCGTATAATATTTGCCCATGGTGAAAACGGGGGCGAAGAAGTTGTCCATATTGGCCACGTTTAAATCA

AAACTGGTGAAACTCACCCAGGGATTGGCTGAGACGAAAAACATATTCTCAATAAACCCTTTAGGGAAAT

AGGCCAGGTTTTCACCGTAACACGCCACATCTTGCGAATATATGTGTAGAAACTGCCGGAAATCGTCGTG

GTATTCACTCCAGAGCGATGAAAACGTTTCAGTTTGCTCATGGAAAACGGTGTAACAAGGGTGAACACTA

TCCCATATCACCAGCTCACCGTCTTTCATTGCCATACGGAATTCCGGATGAGCATTCATCAGGCGGGCAA

GAATGTGAATAAAGGCCGGATAAAACTTGTGCTTATTTTTCTTTACGGTCTTTAAAAAGGCCGTAATATC

CAGCTGAACGGTCTGGTTATAGGTACATTGAGCAACTGACTGAAATGCCTCAAAATGTTCTTTACGATGC

CATTGGGATATATCAACGGTGGTATATCCAGTGATTTTTTTCTCCATTTTAGCTTCCTTAGCTCCTGAAA

ATCTCGACGGATCCTAACTCAAAATCCACACATTATACGAGCCGGAAGCATAAAGTGTAAAGCCTGGGGT

GCCTAATGCGGCCGCCATAGTGACTGGATATGTTGTGTTTTACAGTATTATGTAGTCTGTTTTTTATGCA

AAATCTAATTTAATATATTGATATTTATATCATTTTACGTTTCTCGTTCAGCTTTTTTGTACAAACTTGT

GATAACTTCCGATCGATTCTAGACTAGTTCTAGAGTCCGCAAAAATCACCAGTCTCTCTCTACAAATCTA

TCTCTCTCTATTTTTCTCCAGAATAATGTGTGAGTAGTTCCCAGATAAGGGAATTAGGGTTCTTATAGGG

TTTCGCTCATGTGTTGAGCATATAAGAAACCCTTAGTATGTATTTGTATTTGTAAAATACTTCTATCAAT

AAAATTTCTAATTCCTAAAACCAAAATCCAGTGACCGGGCGGCCGCCACCGCGGTGGAGGGGGATCAGAT

TGTCGTTTCCCGCCTTCAGTTTAAACTATCAGTGTTTGACAGGATATATTGGCGGGTAAACCTAAGAGAA

AAGAGCGTTTATTAGAATAATCGGATATTTAAAAGGGCGTGAAAAGGTTTATCCGTTCGTCCATTTGTAT

GTGTACATCACCGACGAGCAAGGCAAGACCGAGCGCCTTTCCGACGCTCACCGGGCTGGTTGCCCTCGCC

GCTGGGCTGGCGGCCGTCTATGGCCCTGCAAACGCGCCAGAAACGCCGTCGAAGCCGTGTGCGAGACACC

GGCCGCCGGCGTTGTGGATACCTCGCGGAAAACTTGGCCCTCACTGACAGATGAGGGGCGGACGTTGACA

CTTGAGGGGCCGACTCACCCGGCGCGGCGTTGACAGATGAGGGGCAGGCTCGATTTCGGCCGGCGACGTG

GAGCTGGCCAGCCTCGCAAATCGGCGAAAACGCCTGATTTTACGCGAGTTTCCCACAGATGATGTGGACA

AGCCTGGGGATAAGTGCCCTGCGGTATTGACACTTGAGGGGCGCGACTACTGACAGATGAGGGGCGCGAT

CCTTGACACTTGAGGGGCAGAGTGCTGACAGATGAGGGGCGCACCTATTGACATTTGAGGGGCTGTCCAC

AGGCAGAAAATCCAGCATTTGCAAGGGTTTCCGCCCGTTTTTCGGCCACCGCTAACCTGTCTTTTAACCT

GCTTTTAAACCAATATTTATAAACCTTGTTTTTAACCAGGGCTGCGCCCTGTGCGCGTGACCGCGCACGC

CGAAGGGGGGTGCCCCCCCTTCTCGAACCCTCCCGGCCCGCTAACGCGGGCCTCCCATCCCCCCAGGGGC

TGCGCCCCTCGGCCGCGAACGGCCTCACCCCAAAAATGGCAGCGCTGGCAGTCCTTGCCATTGCCGGGAT

CGGGGCAGTAACGGGATGGGCGATCAGCCCGACAAGCTACCCCTATTTGTTTATTTTTCTAAATACATTC

AAATATGTATCCGCTCATGAGACAATAACCCTGATAAATGCTTCAATAATATTGAAAAAGGAAGAGTATG

AGTATTCAACATTTCCGTGTCGCCCTTATTCCCTTTTTTGCGGCATTTTGCCTTCCTGTTTTTGCTCACC

CAGAAACGCTGGTGAAAGTAAAAGATGCTGAAGATCAGTTGGGTGCACGAGTGGGTTACATCGAACTGGA

TCTCAACAGCGGTAAGATCCTTGAGAGTTTTCGCCCCGAAGAACGTTTTCCAATGATGAGCACTTTTAAA

GTTCTGCTATGTGGCGCGGTATTATCCCGTATTGACGCCGGGCAAGAGCAACTCGGTCGCCGCATACACT

ATTCTCAGAATGACTTGGTTGAGTACTCACCAGTCACAGAAAAGCATCTTACGGATGGCATGACAGTAAG

AGAATTATGCAGTGCTGCCATAACCATGAGTGATAACACTGCGGCCAACTTACTTCTGACAACGATCGGA

GGACCGAAGGAGCTAACCGCTTTTTTGCACAACATGGGGGATCATGTAACTCGCCTTGATCGTTGGGAAC

CGGAGCTGAATGAAGCCATACCAAACGACGAGCGTGACACCACGATGCCTGTAGCAATGGCAACAACGTT

GCGCAAACTATTAACTGGCGAACTACTTACTCTAGCTTCCCGGCAACAATTAATAGACTGGATGGAGGCG

GATAAAGTTGCAGGACCACTTCTGCGCTCGGCCCTTCCGGCTGGCTGGTTTATTGCTGATAAATCTGGAG

CCGGTGAGCGTGGGTCTCGCGGTATCATTGCAGCACTGGGGCCAGATGGTAAGCCCTCCCGTATCGTAGT

TATCTACACGACGGGGAGTCAGGCAACTATGGATGAACGAAATAGACAGATCGCTGAGATAGGTGCCTCA

CTGATTAAGCATTGGTAACTGTCAGACCAAGTTTACTCATATATACTTTAGATTGATTTAAAACTTCATT

TTTAATTTAAAAGGATCTAGGTGAAGATCCTTTTTGATAATCTCATGACCAAAATCCCTTAACGTGAGTT

TTCGTTCCACTGAGCGTCAGACCCCGTAGAAAAGATCAAAGGATCTTCTTGAGATCCTTTTTTTCTGCGC

GTAATCTGCTGCTTGCAAACAAAAAAACCACCGCTACCAGCGGTGGTTTGTTTGCCGGATCAAGAGCTAC

CAACTCTTTTTCCGAAGGTAACTGGCTTCAGCAGAGCGCAGATACCAAATACTGTCCTTCTAGTGTAGCC

GTAGTTAGGCCACCACTTCAAGAACTCTGTAGCACCGCCTACATACCTCGCTCTGCTAATCCTGTTACCA

GTGGCTGCTGCCAGTGGCGATAAGTCGTGTCTTACCGGGTTGGACTCAAGACGATAGTTACCGGATAAGG

CGCAGCGGTCGGGCTGAACGGGGGGTTCGTGCACACAGCCCAGCTTGGAGCGAACGACCTACACCGAACT

GAGATACCTACAGCGTGAGCTATGAGAAAGCGCCACGCTTCCCGAAGGGAGAAAGGCGGACAGGTATCCG

GTAAGCGGCAGGGTCGGAACAGGAGAGCGCACGAGGGAGCTTCCAGGGGGAAACGCCTGGTATCTTTATA

GTCCTGTCGGGTTTCGCCACCTCTGACTTGAGCGTCGATTTTTGTGATGCTCGTCAGGGGGGCGGAGCCT

ATGGAAAAACGCCAGCAACGCGGCCTTTTTACGGTTCCTGGCCTTTTGCTGGCCTTTTGCTCACATGGAC

TCTAGCTAGAGGATCACAGGCAGCAACGCTCTGTCATCGTTACAATCAACATGCTACCCTCCGCGAGATC

ATCCGTGTTTCAAACCCGGCAGCTTAGTTGCCGTTCTTCCGAATAGCATCGGTAACATGAGCAAAGTCTG

CCGCCTTACAACGGCTCTCCCGCTGACGCCGTCCCGGACTGATGGGCTGCCTGTATCGAGTGGTGATTTT

GTGCCGAGCTGCCGGTCGGGGAGCTGTTGGCTGGCTGGTGGCAGGATATATTGTGGTGTAAACAAATTGA

CGCTTAGACAACTTAATAACACATTGCGGACGTTTTTAATGATCGAATACTAACGTCTCTACCAGATATC

AGCTTGCATGCCGGTCGATCTAGTAACATAGATGACACCGCGCGCGATAATTTATCCTAGTTTGCGCGCT

ATATTTTGTTTTCTATCGCGTATTAAATGTATAATTGCGGGACTCTAATCATAAAAACCCATCTCATAAA

TAACGTCATGCATTACATGTTAATTATTACATGCTTAACGTAATTCAACAGAAATTATATGATAATCATC

GCAAGACCGGCAACAGGATTCAATCTTAAGAAACTTTATTGCCAAATGTTTGAACGATCTGCTTGACTCT

AGGGGTCATCAGATTTCGGTGACGGGCAGGACCGGACGGGGCGGCACCGGCAGGCTGAAGTCCAGCTGCC

AGAAACCCACGTCATGCCAGTTCCCGTGCTTGAAGCCGGCCGCCCGCAGCATGCCGCGGGGGGCATATCC

GAGCGCCTCGTGCATGCGCACGCTCGGGTCGTTGGGCAGCCCGATGACAGCGACCACGCTCTTGAAGCCC

TGTGCCTCCAGGGACTTCAGCAGGTGGGTGTAGAGCGTGGAGCCCAGTCCCGTCCGCTGGTGGCGGGGGG

AGACGTACACGGTCGACTCGGCCGTCCAGTCGTAGGCGTTGCGTGCCTTCCAGGGGCCCGCGTAGGCGAT

GCCGGCGACCTCGCCGTCCACCTCGGCGACGAGCCAGGGATAGCGCTCCCGCAGACGGACGAGGTCGTCC

GTCCACTCCTGCGGTTCCTGCGGCTCGGTACGGAAGTTGACCGTGCTTGTCTCGATGTAGTGGTTGACGA

TGGTGCAGACCGCCGGCATGTCCGCCTCGGTGGCACGGCGGATGTCGGCCGGGCGTCGTTCTGGGCTCAT

GGTAGATCCCCCTCGATCGAGTTGAGAGTGAATATGAGACTCTAATTGGATACCGAGGGGAATTTATGGA

ACGTCAGTGGAGCATTTTTGACAAGAAATATTTGCTAGCTGATAGTGACCTTAGGCGACTTTTGAACGCG

CAATAATGGTTTCTGACGTATGTGCTTAGCTCATTAAACTCCAGAAACCCGCGGCTCAGTGGCTCCTTCA

ACGTTGCGGTTCTGTCAGTTCCAAACGTAAAACGGCTTGTCCCGCGTCATCGGCGGGGGTCATAACGTGA

CTCCCTTAATTCTCCGCTCATGATCAGATTGTCGTTTCCCGCCTTCGGTTTGGGCGCGCCCGGTCTCAGA

AGACCAGAGGGCTATTGAGACTTTTCAACAAAGGGTAATATCGGGAAACCTCCTCGGATTCCATTGCCCA

GCTATCTGTCACTTCATCGAAAGGACAGTAGAAAAGGAAGATGGCTTCTACAAATGCCATCATTGCGATA

AAGGAAAGGCTATCGTTCAAGAATGCCTCTACCGACAGTGGTCCCAAAGATGGACCCCCACCCACGAGGA

ACATCGTGGAAAAAGAAGACGTTCCAACCACGTCTTCAAAGCAAGTGGATTGATGTGATAACATGGTGGA

GCACGACACTCTCGTCTACTCCAAGAATATCAAAGATACAGTCTCAGAAGACCAGAGGGCTATTGAGACT

TTTCAACAAAGGGTAATATCGGGAAACCTCCTCGGATTCCATTGCCCAGCTATCTGTCACTTCATCGAAA

GGACAGTAGAAAAGGAAGATGGCTTCTACAAATGCCATCATTGCGATAAAGGAAAGGCTATCGTTCAAGA

ATGCCTCTACCGACAGTGGTCCCAAAGATGGACCCCCACCCACGAGGAACATCGTGGAAAAAGAAGACGT

TCCAACCACGTCTTCAAAGCAAGTGGATTGATGTGATATCTCCACTGACGTAAGGGATGACGCACAATCC

CACTATCCTTCGCAAGACCCTTCCTCTATATAAGGAAGTTCATTTCATTTGGAGAGGAC

>gi|22773774|gb|AY141042.1| Binary vector pGV4945 kanamycin resistance and ribosome-inactivating protein genes, complete cds

GGCAGGATATATTCAATTGTAAATGGCTTCATGTCCGGGAAATCTACATGGATCAGCAATGAGTATGATG

GTCAATATGGAGAAAAAGAAAGAGTAATTACCAATTTTTTTTCAATTCAAAAATGTAGATGTCCGCAGCG

TTATTATAAAATGAAAGTACATTTTGATAAAACGACAAATTACGATCCGTCGTATTTATAGGCGAAAGCA

ATAAACAAATTATTCTAATTCGGAAATCTTTATTTCGACGTGTCTACATTCACGTCCAAATGGGGGCTTA

GATGAGAAACTTCACGATCGATGCCTTGATTTCGCCATTCCCAGATACCCATTTCATCTTCAGATTGGTC

TGAGATTATGCGAAAATATACACTCATATACATAAATACTGACAGTTTGAGCTACCAATTCAGTGTAGCC

CATTACCTCACATAATTCACTCAAATGCTAGGCAGTCTGTCAACTCGGCGTCAATTTGTCGGCCACTATA

CGATAGTTGCGCAAATTTTCAAAGTCCTGGCCTAACATCACACCTCTGTCGGCGGCGGGTCCCATTTGTG

ATAAATCCACCATCACAATAGATAGTCTAATGGACGAAAAAGGCGAATATTTCGATGCTGAGATTCGACG

CAATTAATTCGAGAAAAATCCCGTGATTGATGCTGTTGAGTTACCAATAATATGGGCAGCGAAGGCCATT

TAATTATAAGATCCGTCTCGACCAAAGCGGCCATCGTGCCTCCCCACTCCTGCTGTTCGGGGGCATGGAT

GCGCGGATAGCCGCTGCTGGTTTCCTGGATGCCGACCGGATTTGCACTGCCGGTAGAACTCCGCGAGGTC

GTCCAGCCTCAGGCAGCAGCTGAACCAACTCGCGAGGGGATCGAGCCCCTGCTGAGCCTCGACATGTTGT

CGCAAAATTCGCCCTGGACCCGCCCAACGATTTGTCGTCACTGTCAAGGTTTGACCTGCACTTCATTTGG

GGCCCACATACACCAAAAAAATGCTGCATAATTCTCGGGGCAGCAAGTCGGTTACCCGGCCGCCGTGCTG

GACCGGGTTGAATGGTGCCCGTAACTTTCGGTAGAGCGGACGGCCAATACTCAACTTCAAGGAATCTCAC

CCATGCGCGCCGGCGGGGAACCGGAGTTCCCTTCAGTGAACGTTATTAGTTCGCCGCTCGGTGTGTCGTA

GATACTAGCCCCTGGGGCCTTTTGAAATTTGAATAAGATTTATGTAATCAGTCTTTTAGGTTTGACCGGT

TCTGCCGCTTTTTTTAAAATTGGATTTGTAATAATAAAACGCAATTGTTTGTTATTGTGGCGCTCTATCA

TAGATGTCGCTATAAACCTATTCAGCACAATATATTGTTTTCATTTTAATATTGTACATATAAGTAGTAG

GGTACAATCAGTAAATTGAACGGAGAATATTATTCATAAAAATACGATAGTAACGGGTGATATATTCATT

CATTAGAATGAACCGAAACCGGCGGTAAGGATCTGAGCTACACATGCTCAGGTTTTTTACAACGTGCACA

ACAGAATTGAAAGCAAATATCATGCGATCATAGGCGTCTCGCATATCTCATTAAAGCAGGGGGTGGGCGA

AGAACTCCAGCATGAGATCCCCGCGCTGGAGGATCATCCAGCCGGCGTCCCGGAAAACGATTCCGAAGCC

CAACCTTTCATAGAAGGCGGCGGTGGAATCGAAATCTCGTGATGGCAGGTTGGGCGTCGCTTGGTCGGCA

TTTCGAACCCCAGAGTCCCGCTCAGAAGAACTCGTCAAGAAGGCGATAGAAGGCGATGCGCTGCGAATCG

GGAGCGGCGATACCGTAAAGCACGAGGAAGCGGTCAGCCCATTCGCCGCCAAGCTCTTCAGCAATATCAC

GGGTAGCCAACGCTATGTCCTGATAGCGGTCCGCCACACCCAGCCGGCCACAGTCGATGAATCCAGAAAA

GCGGCCATTTTCCACCATGATATTCGGCAAGCACGCATCGCCATGGGTCACGACGAGATCCTCGCCGTCG

GGCATGCGCGCCTTGAGCCTGGCGAACAGTTCGGCTGGCGCGAGCCCCTGATGCTCTTCGTCCAGATCAT

CCTGATCGACAAGACCGGCTTCCATCCGAGTACGTGCTCGCTCGATGCGATGTTTCGCTTGGTGGTCGAA

TGGGCAGGTAGCCGGATCAAGCGTATGCAGCCGCCGCATTGCATCAGCCATGATGGATACTTTCTCGGCA

GGAGCAAGGTGAGATGACAGGAGATCCTGCCCCGGCACTTCGCCCAATAGCAGCCAGTCCCTTCCCGCTT

CAGTGACAACGTCGAGCACAGCTGCGCAAGGAACGCCCGTCGTGGCCAGCCACGATAGCCGCGCTGCCTC

GTCCTGCAGTTCATTCAGGGCACCGGACAGGTCGGTCTTGACAAAAAGAACCGGGCGCCCCTGCGCTGAC

AGCCGGAACACGGCGGCATCAGAGCAGCCGATTGTCTGTTGTGCCCAGTCATAGCCGAATAGCCTCTCCA

CCCAAGCGGCCGGAGAACCTGCGTGCAATCCATCTTGTTCAATCCACATGATCATGGGCCGGATCTGGAT

TGAGAGTGAATATGAGACTCTAATTGGATACCGAGGGGAATTTATGGAACGTCAGTGGAGCATTTTTGAC

AAGAAATATTTGCTAGCTGATAGTGACCTTAGGCGACTTTTGAACGCGCAATAATGGTTTCTGACGTATG

TGCTTAGCTCATTAAACTCCAGAAACCCGCGGCTGAGTGGCTCCTTCAACGTTGCGGTTCTGTCAGTTCC

AAACGTAAAACGGCTTGTCCCGCGTCATCGGCGGGGGTCATAACGTGACTCCCTTAATTCTCCGCTCATG

ATCCTGTTTCCTGTGTGAAATTGTTATCCGCTCACAATTCCACACATTATACGAGCCGGAAGCATAAAGT

GTAAAGCCTGGGGTGCCTAATGAGTGAGAATTGACGAGATCTTTCCCGGCGTTTGCCCAGCACCTATTTC

CTGCCTGCGCTGATTACGACCAAAATGTTATTCAACATGATTGGCTTACAGACGAGAATGCCGGGGGAGC

TTTCAAACTCAACCGGCGTGGTGAGGATTTTTATTCTGAAGAACTTTTCTTTCAAGCACTGGACACGGCT

AATGATACCGGAGTTTACTTGGCGGGTTGCAGTTGTTCCTTCACAGGTGGATGGGTGGAGGGTGCTATTC

AGACCGCGTGTAACGCCGTCTGTGCAATTATCCACAATTGTGGAGGCATTTTGGCAAAGGGCAATCCTCT

CGAACACTCTTGGAAGAGATATAACTACCGCACTAGAAATTAGTCTATGGATCCGATATCAAGCTTGCAT

GCCTGCAGGTCCCCAGATTAGCCTTTTCAATTTCAGAAAGAATGCTAACCCACAGATGGTTAGAGAGGCT

TACGCAGCAGGTCTCATCAAGACGATCTACCCGAGCAATAATCTCCAGGAAATCAAATACCTTCCCAAGA

AGGTTAAAGATGCAGTCAAAAGATTCAGGACTAACTGCATCAAGAACACAGAGAAAGATATATTTCTCAA

GATCAGAAGTACTATTCCAGTATGGACGATTCAAGGCTTGCTTCACAAACCAAGGCAAGTAATAGAGATT

GGAGTCTCTAAAAAGGTAGTTCCCACTGAATCAAAGGCCATGGAGTCAAAGATTCAAATAGAGGACCTAA

CAGAACTCGCCGTAAAGACTGGCGAACAGTTCATACAGAGTCTCTTACGACTCAATGACAAGAAGAAAAT

CTTCGTCAACATGGTGGAGCACGACACACTTGTCTACTCCAAAAATATCAAAGATACAGTCTCAGAAGAC

CAAAGGGCAATTGAGACTTTTCAACAAAGGGTAATATCGGGAAACCTCCTCGGATTCCATTGCCCAGCTA

TCTGTCACTTTATTGTGAAGATAGTGGAAAAGGAAGGTGGCTCCTACAAATGCCATCATTGCGATAAAGG

AAAGGCCATCGTTGAAGATGCCTCTGCCGACAGTGGTCCCAAAGATGGACCCCCACCCACGAGGAGCATC

GTGGAAAAAGAAGACGTTCCAACCACGTCTTCAAAGCAAGTGGATTGATGTGATATCTCCACTGACGTAA

GGGATGACGCACAATCCCACTATCCTTCGCAAGACCCTTCCTCTATATAAGGAAGTTCATTTCATTTGGA

GAGAACACGGGGGACTCTAGAGGATCCCCAATTCGCCCTTATGAGAGTGGTGGCAGGAATATTGTACATT

GTGGTTGTGGCAATTTGCGGCCTTGGAATCCAAGGCGGCACCCTGCAGCAGGACTATCCCTCCGTCTCTT

TCCAAGGCAGCACCCTGCAGCAGCAGGACTTTCCCACCATTCTCTTCAATATTTTGGCGGGCGAGACATA

CGGAGGCTTCATAGCGGAACTGCGAGAGGTTGTGACCCGGACAGCCGATACCAAAAATGGTATACCAGTA

CTGCTGAACCCAGCAAATCCAGTGCCGGTTCGGGAGCGGTTCGTTATGGTCCATCTCACTGGTCGCAACA

ACAAAACCGTTATCTTGGCACTAGATGTGACGAACCTTTATGTGGCGGCTTTTAGCGCAAATAACGTTGC

CTACTTTTTCAGTGACTTTACGGCCCTTGAACGTGAAAATTTATTCTCCGGCATGCTCAGTATCAGGTTG

AGTTTCACCAGCAAGTATGTCAGCCTTGAGCACAAGGCTGGAGTTGGCGGCGGCAGGGAAAATATCTCTC

TGGGGCCCACTCCACTAGATGAAGCCACCAATAGCTTGTGGTCCGGTAGGAGCGTAACTGAAGCGAGCAT

AGCCGAAGCGCTCCTTGTGGTCATTCAGATGGTGTCGGAAGCGGCAAGGTTCAGGCACATTGAGGAACGA

GTCCGCAGAAGCTTTACGGCCGCTGATCATCAGCTGACTTTTAGACCGGACGGTTTGATGCTGAGCATGG

AGAACGAGTGGTCGTCCATGTCCTTGGAGGTCCAGCGGCCCATAGAGGGAGGGATCTTCATTGGGGTCGT

TCAACTTGAAGACGAGAGTTTCAAGCCCCTCAGAGTAGACAACTTTAATACACTCAGTCGCTATACCATG

GTAGCGCTTCTTCTCTTCCGATGCGGCCACCCTCCCGCTGCAGCTCAGATCATAAGAATGCCTGTTGTTC

TTGCAGGCAGGGAGTACGACGACGACGACGAGACGTGTACGGTTGGAGCGCCCACAAGACGCATCAGCGG

TCGGGATGGATTGTGCGTGGACGTGAGGGATGGGCGTGACAACGATGGGAATCCCATCCAGCTGTGGCCA

TGTGGTGCTCAGAGAAACCAACAATGGACGTTCCACACAGACGGAACTATCCGGTCGATGGGTAAATGCA

TGACTACCTACGGGTACAGCCCAGGAAACTATGTAATGATATTCAACTGTCGTACCGCTGTTCCAGACGC

CACCAAGTGGGTAGTATCAATTGACGGCAGCATAACCAATCCTCGCTCTGGACTCGTCCTCACCGCTCTT

CAGGCTGCACACGGCACCACCCTGACGGTAAATACAAATACCCACTCCGCAAGGCAGGGTTGGAGCGTGG

GAGATGATGTACAGCCCATCGTTACTTACATTGTGGGGTTTAAAGATATGTGCTTGCAGCGCAACAGCAC

TCGTGTATGGCTGGAGGATTGTGCGGTCGACAGGCAGCAGCAACGGTGGGCACTCTACAGTGACGGCACC

ATCCGAGTAGATAGCGATCGTAGCCTATGCGTGACCTCCAATGGCCACAGCTCCAGGGATGTCATCATCA

TCCTTACATGCGACGGCCGGGGCAACCAGCGCTGGGTATTCAACACCGACGGCACCATCTTAAACCCAAA

TGCTCAACTGGTTATGGACGTGAGAGGATCCGATGTCGCTCTTCGCCAGATCATTCTCTATCAACCCACT

GGGAATCCTAACCAGCAATGGATGACAATGACACGCACCCGCCCAAGTTTGACAAGTTAAAGGGCGAATT

GGGTACCGAGCTCGAATTTCCCCGATCGTTCAAACATTTGGCAATAAAGTTTCTTAAGATTGAATCCTGT

TGCCGGTCTTGCGATGATTATCATATAATTTCTGTTGAATTACGTTAAGCATGTAATAATTAACATGTAA

TGCATGACGTTATTTATGAGATGGGTTTTTATGATTAGAGTCCCGCAATTATACATTTAATACGCGATAG

AAAACAAAATATAGCGCGCAAACTAGGATAAATTATCGCGCGCGGTGTCATCTATGTTACTAGATCGGGA

ATTCGTTGATCTAACATCTACAAATTGCCTTTTCTTATCGACCATGTACGTAAGCGCTTACGTTTTTGGT

GGACCCTTGAGGAAACTGGTAGCTGTTGTGGGCCTGTGGTCTCAAGATGGATCATTAATTTCCACCTTCA

CCTACGATGGGGGGCATCGCACCGGTGAGTAATATTGTACGGCTAAGAGCGAATTTGGCCTGTAGACCTC

AATTGCGAGCTTTCTAATTTCAAACTATTCGGGCCTAACTTTTGGTGTGATGATGCTGACTGGCAGGATA

TATACCGTTGTAATT

>gi|23381124|emb|AJ414109.1|ATU414109 Binary vector pGV4126 DNA, region between right and left T-DNA border and LECASAII gene

AATTACAACGGTATATATCCTGCCAGTCAGCATCATCACACCAAAAGTTAGGCCCGAATAGTTTGAAATT

AGAAAGCTCGCAATTGAGGTCTACAGGCCAAATTCGCTCTTAGCCGTACAATATTACTCACCGGTGCGAT

GCCCCCCATCGTAGGTGAAGGTGGAAATTAATGATCCATCTTGAGACCACAGGCCCACAACAGCTACCAG

TTTCCTCAAGGGTCCACCAAAAACGTAAGCGCTTACGTACATGGTCGATAAGAAAAGGCAATTTGTAGAT

GTTAGATCTTCTAGAGTTAACGAATTACGTTAAGCATGTAATAATTAACATGTAATGCATGACGTTATTT

ATGAGATGGGTTTTTATGATTAGAGTCCCGCAATTATACATTTAATACGCGATAGAAAACAAAATATAGC

GCGCAAACTAGGATAAATTATCGCGCGCGGTGTCATCTATGTTACTAGATCGGGAATTCGTCGACAAGCT

TGTCGACGAATTCGCGGCCGCTAACCAGCAAACTGTGCTTAATATATATAATGCAGCATAACAGTTGACA

TAAGTACAGTGCATCACCATCACGCACATTATTCATTATTCATTATTCAAACTTAAAACATGGAACACAC

ATCCTAAAACTCAAGCAGCACCGGCCAGAGTGCCAACCTTTCGGATGACAGGTCCACGAGCACTTCCTTT

GTAGGTATTAGTAGACCAAATATCAGATCCGTAAATGACAACGTTACCGTCCTCTTGAAGCACCAGGACA

TAGTTTCCATTCCCTCTTACACTGTGACTGGCCCAGAGGGAACGTCCTTCGGCATCGTAGACCACAAAGT

TGCCATCAGACTGCAGCACCGCCTTGCATCCTTTTTTTCCGGGGATGTCAGTGTTTGAGGTCCAGACGGC

AGTGCTGTGGTCGTACAGCACGAGGTTGCAGTCTTCCTGCATTATAAGATGGTACGGTTCCACATCTAGG

GATTGGCCTGCGTACAGACCTTCGTCGTTCATCAGTATGTTCCTGGCCATGCATGTGGATGCCCATATGG

TTAGAATAGCGGCTACAGTGGCTATGCTCATTACTTTAGGAGATGGAGTAGTCATTCTTTGCGGCCGCGA

ATTAGAGTCCCCCGTGTTCTCTCCAAATGAAATGAACTTCCTTATATAGAGGAAGGGTCTTGCGAAGGAT

AGTGGGATTGTGCGTCATCCCTTACGTCAGTGGAGATATCACATCAATCCACTTGCTTTGAAGACGTGGT

TGGAACGTCTTCTTTTTCCACGATGCTCCTCGTGGGTGGGGGTCCATCTTTGGGACCACTGTCGGCAGAG

GCATCTTCAACGATGGCCTTTCCTTTATCGCAATGATGGCATTTGTAGGAGCCACCTTCCTTTTCCACTA

TCTTCACAATAAAGTGACAGATAGCTGGGCAATGGAATCCGAGGAGGTTTCCCGATATTACCCTTTGTTG

AAAAGTCTCAATTGCCCTTTGGTCTTCTGAGACTGTATCTTTGATATTTTTGGAGTAGACAAGTGTGTCG

TGCTCCACCATGTTGACGAAGATTTTCTTCTTGTCATTGAGTCGTAAGAGACTCTGTATGAACTGTTCGC

CAGTCTTTACGGCGAGTTCTGTTAGGTCCTCTATTTGAATCTTTGACTCCATGGCCTTTGATTCAGTGGG

AACTACCTTTTTAGAGACTCCAATCTCTATTACTTGCCTTGGTTTGTGAAGCAAGCCTTGAATCGTCCAT

ACTGGAATAGTACTTCTGATCTTGAGAAATATATCTTTCTCTGTGTTCTTGATGCAGTTAGTCCTGAATC

TTTTGACTGCATCTTTAACCTTCTTGGGAAGGTATTTGATTTCCTGGAGATTATTGCTCGGGTAGATCGT

CTTGATGAGACCTGCTGCGTAAGCCTCTCTAACCATCTGTGGGTTAGCATTCTTTCTGAAATTGAAAAGG

CTAATCTGGGGACCTGCAGGCATGCAAGCTTGATATCGGATCCATAGACTAATTTCTAGTGCGGTAGTTA

TATCTCTTCCAAGAGTGTTCGAGAGGATTGCCCTTTGCCAAAATGCCTCCACAATTGTGGATAATTGCAC

AGACGGCGTTACACGCGGTCTGAATAGCACCCTCCACCCATCCACCTGTGAAGGAACAACTGCAACCCGC

CAAGTAAACTCCGGTATCATTAGCCGTGTCCAGTGCTTGAAAGAAAAGTTCTTCAGAATAAAAATCCTCA

CCACGCCGGTTGAGTTTGAAAGCTCCCCCGGCATTCTCGTCTGTAAGCCAATCATGTTGAATAACATTTT

GGTCGTAATCAGCGCAGGCAGGAAATAGGTGCTGGGCAAACGCCGGGAAAGATCTCGTCAATTCTCACTC

ATTAGGCACCCCAGGCTTTACACTTTATGCTTCCGGCTCGTATAATGTGTGGAATTGTGAGCGGATAACA

ATTTCACACAGGAAACAGGATCATGAGCGGAGAATTAAGGGAGTCACGTTATGACCCCCGCCGATGACGC

GGGACAAGCCGTTTTACGTTTGGAACTGACAGAACCGCAACGTTGAAGGAGCCACTCAGCCGCGGGTTTC

TGGAGTTTAATGAGCTAAGCACATACGTCAGAAACCATTATTGCGCGTTCAAAAGTCGCCTAAGGTCACT

ATCAGCTAGCAAATATTTCTTGTCAAAAATGCTCCACTGACGTTCCATAAATTCCCCTCGGTATCCAATT

AGAGTCTCATATTCACTCTCAATCCAGATCCGGCCCATGATCATGTGGATTGAACAAGATGGATTGCACG

CAGGTTCTCCGGCCGCTTGGGTGGAGAGGCTATTCGGCTATGACTGGGCACAACAGACAATCGGCTGCTC

TGATGCCGCCGTGTTCCGGCTGTCAGCGCAGGGGCGCCCGGTTCTTTTTGTCAAGACCGACCTGTCCGGT

GCCCTGAATGAACTGCAGGACGAGGCAGCGCGGCTATCGTGGCTGGCCACGACGGGCGTTCCTTGCGCAG

CTGTGCTCGACGTTGTCACTGAAGCGGGAAGGGACTGGCTGCTATTGGGCGAAGTGCCGGGGCAGGATCT

CCTGTCATCTCACCTTGCTCCTGCCGAGAAAGTATCCATCATGGCTGATGCAATGCGGCGGCTGCATACG

CTTGATCCGGCTACCTGCCCATTCGACCACCAAGCGAAACATCGCATCGAGCGAGCACGTACTCGGATGG

AAGCCGGTCTTGTCGATCAGGATGATCTGGACGAAGAGCATCAGGGGCTCGCGCCAGCCGAACTGTTCGC

CAGGCTCAAGGCGCGCATGCCCGACGGCGAGGATCTCGTCGTGACCCATGGCGATGCGTGCTTGCCGAAT

ATCATGGTGGAAAATGGCCGCTTTTCTGGATTCATCGACTGTGGCCGGCTGGGTGTGGCGGACCGCTATC

AGGACATAGCGTTGGCTACCCGTGATATTGCTGAAGAGCTTGGCGGCGAATGGGCTGACCGCTTCCTCGT

GCTTTACGGTATCGCCGCTCCCGATTCGCAGCGCATCGCCTTCTATCGCCTTCTTGACGAGTTCTTCTGA

GCGGGACTCTGGGGTTCGAAATGCCGACCAAGCGACGCCCAACCTGCCATCACGAGATTTCGATTCCACC

GCCGCCTTCTATGAAAGGTTGGGCTTCGGAATCGTTTTCCGGGACGCCGGCTGGATGATCCTCCAGCGCG

GGGATCTCATGCTGGAGTTCTTCGCCCACCCCCTGCTTTAATGAGATATGCGAGACGCCTATGATCGCAT

GATATTTGCTTTCAATTCTGTTGTGCACGTTGTAAAAAACCTGAGCATGTGTAGCTCAGATCCTTACCGC

CGGTTTCGGTTCATTCTAATGAATGAATATATCACCCGTTACTATCGTATTTTTATGAATAATATTCTCC

GTTCAATTTACTGATTGTACCCTACTACTTATATGTACAATATTAAAATGAAAACAATATATTGTGCTGA

ATAGGTTTATAGCGACATCTATGATAGAGCGCCACAATAACAAACAATTGCGTTTTATTATTACAAATCC

AATTTTAAAAAAAGCGGCAGAACCGGTCAAACCTAAAAGACTGATTACATAAATCTTATTCAAATTTCAA

AAGGCCCCAGGGGCTAGTATCTACGACACACCGAGCGGCGAACTAATAACGTTCACTGAAGGGAACTCCG

GTTCCCCGCCGGCGCGCATGGGTGAGATTCCTTGAAGTTGAGTATTGGCCGTCCGCTCTACCGAAAGTTA

CGGGCACCATTCAACCCGGTCCAGCACGGCGGCCGGGTAACCGACTTGCTGCCCCGAGAATTATGCAGCA

TTTTTTTGGTGTATGTGGGCCCCAAATGAAGTGCAGGTCAAACCTTGACAGTGACGACAAATCGTTGGGC

GGGTCCAGGGCGAATTTTGCGACAACATGTCGAGGCTCAGCAGGGGCTCGATCCCCTCGCGAGTTGGTTC

AGCTGCTGCCTGAGGCTGGACGACCTCGCGGAGTTCTACCGGCAGTGCAAATCCGGTCGGCATCCAGGAA

ACCAGCAGCGGCTATCCGCGCATCCATGCCCCCGAACAGCAGGAGTGGGGAGGCACGATGGCCGCTTTGG

TCGAGACGGATCTTATAATTAAATGGCCTTCGCTGCCCATATTATTGGTAACTCAACAGCATCAATCACG

GGATTTTTCTCGAATTAATTGCGTCGAATCTCAGCATCGAAATATTCGCCTTTTTCGTCCATTAGACTAT

CTATTGTGATGGTGGATTTATCACAAATGGGACCCGCCGCCGACAGAGGTGTGATGTTAGGCCAGGACTT

TGAAAATTTGCGCAACTATCGTATAGTGGCCGACAAATTGACGCCGAGTTGACAGACTGCCTAGCATTTG

AGTGAATTATGTGAGGTAATGGGCTACACTGAATTGGTAGCTCAAACTGTCAGTATTTATGTATATGAGT

GTATATTTTCGCATAATCTCAGACCAATCTGAAGATGAAATGGGTATCTGGGAATGGCGAAATCAAGGCA

TCGATCGTGAAGTTTCTCATCTAAGCCCCCATTTGGACGTGAATGTAGACACGTCGAAATAAAGATTTCC

GAATTAGAATAATTTGTTTATTGCTTTCGCCTATAAATACGACGGATCGTAATTTGTCGTTTTATCAAAA

TGTACTTTCATTTTATAATAACGCTGCGGACATCTACATTTTTGAATTGAAAAAAAATTGGTAATTACTC

TTTCTTTTTCTCCATATTGACCATCATACTCATTGCTGATCCATGTAGATTTCCCGGACATGAAGCCATT

TACAATTGAATATATCCTGCC

>gi|23381127|emb|AJ414110.1|ATU414110 Binary vector pGV4128 DNA, region between right and left T-DNA border and LECASAII gene

AATTACAACGGTATATATCCTGCCAGTCAGCATCATCACACCAAAAGTTAGGCCCGAATAGTTTGAAATT

AGAAAGCTCGCAATTGAGGTCTACAGGCCAAATTCGCTCTTAGCCGTACAATATTACTCACCGGTGCGAT

GCCCCCCATCGTAGGTGAAGGTGGAAATTAATGATCCATCTTGAGACCACAGGCCCACAACAGCTACCAG

TTTCCTCAAGGGTCCACCAAAAACGTAAGCGCTTACGTACATGGTCGATAAGAAAAGGCAATTTGTAGAT

GTTAGATCTTCTAGAGTTAACGAATTCCCGATCTAGTAACATAGATGACACCGCGCGCGATAATTTATCC

TAGTTTGCGCGCTATATTTTGTTTTCTATCGCGTATTAAATGTATAATTGCGGGACTCTAATCATAAAAA

CCCATCTCATAAATAACGTCATGCATTACATGTTAATTATTACATGCTTAACGTAATTCAACAGAAATTA

TATGATAATCATCGCAAGACCGGCAACAGGATTCAATCTTAAGAAACTTTATTGCCAAATGTTTGAACGA

TCGGGGAAATTCGAGCTCGGTACCCAATTCGCGGCCGCTAACCAGCAAACTGTGCTTAATATATATAATG

CAGCATAACAGTTGACATAAGTACAGTGCATCACCATCACGCACATTATTCATTATTCATTATTCAAACT

TAAAACATGGAACACACATCCTAAAACTCAAGCAGCACCGGCCAGAGTGCCAACCTTTCGGATGACAGGT

CCACGAGCACTTCCTTTGTAGGTATTAGTAGACCAAATATCAGATCCGTAAATGACAACGTTACCGTCCT

CTTGAAGCACCAGGACATAGTTTCCATTCCCTCTTACACTGTGACTGGCCCAGAGGGAACGTCCTTCGGC

ATCGTAGACCACAAAGTTGCCATCAGACTGCAGCACCGCCTTGCATCCTTTTTTTCCGGGGATGTCAGTG

TTTGAGGTCCAGACGGCAGTGCTGTGGTCGTACAGCACGAGGTTGCAGTCTTCCTGCATTATAAGATGGT

ACGGTTCCACATCTAGGGATTGGCCTGCGTACAGACCTTCGTCGTTCATCAGTATGTTCCTGGCCATGCA

TGTGGATGCCCATATGGTTAGAATAGCGGCTACAGTGGCTATGCTCATTACTTTAGGAGATGGAGTAGTC

ATTCTTTGCGGCCGCGAATTGGGGATCCTCTAGAGTCCCCCGTGTTCTCTCCAAATGAAATGAACTTCCT

TATATAGAGGAAGGGTCTTGCGAAGGATAGTGGGATTGTGCGTCATCCCTTACGTCAGTGGAGATATCAC

ATCAATCCACTTGCTTTGAAGACGTGGTTGGAACGTCTTCTTTTTCCACGATGCTCCTCGTGGGTGGGGG

TCCATCTTTGGGACCACTGTCGGCAGAGGCATCTTCAACGATGGCCTTTCCTTTATCGCAATGATGGCAT

TTGTAGGAGCCACCTTCCTTTTCCACTATCTTCACAATAAAGTGACAGATAGCTGGGCAATGGAATCCGA

GGAGGTTTCCCGATATTACCCTTTGTTGAAAAGTCTCAATTGCCCTTTGGTCTTCTGAGACTGTATCTTT

GATATTTTTGGAGTAGACAAGTGTGTCGTGCTCCACCATGTTGACGAAGATTTTCTTCTTGTCATTGAGT

CGTAAGAGACTCTGTATGAACTGTTCGCCAGTCTTTACGGCGAGTTCTGTTAGGTCCTCTATTTGAATCT

TTGACTCCATGGCCTTTGATTCAGTGGGAACTACCTTTTTAGAGACTCCAATCTCTATTACTTGCCTTGG

TTTGTGAAGCAAGCCTTGAATCGTCCATACTGGAATAGTACTTCTGATCTTGAGAAATATATCTTTCTCT

GTGTTCTTGATGCAGTTAGTCCTGAATCTTTTGACTGCATCTTTAACCTTCTTGGGAAGGTATTTGATTT

CCTGGAGATTATTGCTCGGGTAGATCGTCTTGATGAGACCTGCTGCGTAAGCCTCTCTAACCATCTGTGG

GTTAGCATTCTTTCTGAAATTGAAAAGGCTAATCTGGGGACCTGCAGGCATGCAAGCTTGATATCGGATC

CATAGACTAATTTCTAGTGCGGTAGTTATATCTCTTCCAAGAGTGTTCGAGAGGATTGCCCTTTGCCAAA

ATGCCTCCACAATTGTGGATAATTGCACAGACGGCGTTACACGCGGTCTGAATAGCACCCTCCACCCATC

CACCTGTGAAGGAACAACTGCAACCCGCCAAGTAAACTCCGGTATCATTAGCCGTGTCCAGTGCTTGAAA

GAAAAGTTCTTCAGAATAAAAATCCTCACCACGCCGGTTGAGTTTGAAAGCTCCCCCGGCATTCTCGTCT

GTAAGCCAATCATGTTGAATAACATTTTGGTCGTAATCAGCGCAGGCAGGAAATAGGTGCTGGGCAAACG

CCGGGAAAGATCTCGTCAATTCTCACTCATTAGGCACCCCAGGCTTTACACTTTATGCTTCCGGCTCGTA

TAATGTGTGGAATTGTGAGCGGATAACAATTTCACACAGGAAACAGGATCATGAGCGGAGAATTAAGGGA

GTCACGTTATGACCCCCGCCGATGACGCGGGACAAGCCGTTTTACGTTTGGAACTGACAGAACCGCAACG

TTGAAGGAGCCACTCAGCCGCGGGTTTCTGGAGTTTAATGAGCTAAGCACATACGTCAGAAACCATTATT

GCGCGTTCAAAAGTCGCCTAAGGTCACTATCAGCTAGCAAATATTTCTTGTCAAAAATGCTCCACTGACG

TTCCATAAATTCCCCTCGGTATCCAATTAGAGTCTCATATTCACTCTCAATCCAGATCCGGCCCATGATC

ATGTGGATTGAACAAGATGGATTGCACGCAGGTTCTCCGGCCGCTTGGGTGGAGAGGCTATTCGGCTATG

ACTGGGCACAACAGACAATCGGCTGCTCTGATGCCGCCGTGTTCCGGCTGTCAGCGCAGGGGCGCCCGGT

TCTTTTTGTCAAGACCGACCTGTCCGGTGCCCTGAATGAACTGCAGGACGAGGCAGCGCGGCTATCGTGG

CTGGCCACGACGGGCGTTCCTTGCGCAGCTGTGCTCGACGTTGTCACTGAAGCGGGAAGGGACTGGCTGC

TATTGGGCGAAGTGCCGGGGCAGGATCTCCTGTCATCTCACCTTGCTCCTGCCGAGAAAGTATCCATCAT

GGCTGATGCAATGCGGCGGCTGCATACGCTTGATCCGGCTACCTGCCCATTCGACCACCAAGCGAAACAT

CGCATCGAGCGAGCACGTACTCGGATGGAAGCCGGTCTTGTCGATCAGGATGATCTGGACGAAGAGCATC

AGGGGCTCGCGCCAGCCGAACTGTTCGCCAGGCTCAAGGCGCGCATGCCCGACGGCGAGGATCTCGTCGT

GACCCATGGCGATGCGTGCTTGCCGAATATCATGGTGGAAAATGGCCGCTTTTCTGGATTCATCGACTGT

GGCCGGCTGGGTGTGGCGGACCGCTATCAGGACATAGCGTTGGCTACCCGTGATATTGCTGAAGAGCTTG

GCGGCGAATGGGCTGACCGCTTCCTCGTGCTTTACGGTATCGCCGCTCCCGATTCGCAGCGCATCGCCTT

CTATCGCCTTCTTGACGAGTTCTTCTGAGCGGGACTCTGGGGTTCGAAATGCCGACCAAGCGACGCCCAA

CCTGCCATCACGAGATTTCGATTCCACCGCCGCCTTCTATGAAAGGTTGGGCTTCGGAATCGTTTTCCGG

GACGCCGGCTGGATGATCCTCCAGCGCGGGGATCTCATGCTGGAGTTCTTCGCCCACCCCCTGCTTTAAT

GAGATATGCGAGACGCCTATGATCGCATGATATTTGCTTTCAATTCTGTTGTGCACGTTGTAAAAAACCT

GAGCATGTGTAGCTCAGATCCTTACCGCCGGTTTCGGTTCATTCTAATGAATGAATATATCACCCGTTAC

TATCGTATTTTTATGAATAATATTCTCCGTTCAATTTACTGATTGTACCCTACTACTTATATGTACAATA

TTAAAATGAAAACAATATATTGTGCTGAATAGGTTTATAGCGACATCTATGATAGAGCGCCACAATAACA

AACAATTGCGTTTTATTATTACAAATCCAATTTTAAAAAAAGCGGCAGAACCGGTCAAACCTAAAAGACT

GATTACATAAATCTTATTCAAATTTCAAAAGGCCCCAGGGGCTAGTATCTACGACACACCGAGCGGCGAA

CTAATAACGTTCACTGAAGGGAACTCCGGTTCCCCGCCGGCGCGCATGGGTGAGATTCCTTGAAGTTGAG

TATTGGCCGTCCGCTCTACCGAAAGTTACGGGCACCATTCAACCCGGTCCAGCACGGCGGCCGGGTAACC

GACTTGCTGCCCCGAGAATTATGCAGCATTTTTTTGGTGTATGTGGGCCCCAAATGAAGTGCAGGTCAAA

CCTTGACAGTGACGACAAATCGTTGGGCGGGTCCAGGGCGAATTTTGCGACAACATGTCGAGGCTCAGCA

GGGGCTCGATCCCCTCGCGAGTTGGTTCAGCTGCTGCCTGAGGCTGGACGACCTCGCGGAGTTCTACCGG

CAGTGCAAATCCGGTCGGCATCCAGGAAACCAGCAGCGGCTATCCGCGCATCCATGCCCCCGAACAGCAG

GAGTGGGGAGGCACGATGGCCGCTTTGGTCGAGACGGATCTTATAATTAAATGGCCTTCGCTGCCCATAT

TATTGGTAACTCAACAGCATCAATCACGGGATTTTTCTCGAATTAATTGCGTCGAATCTCAGCATCGAAA

TATTCGCCTTTTTCGTCCATTAGACTATCTATTGTGATGGTGGATTTATCACAAATGGGACCCGCCGCCG

ACAGAGGTGTGATGTTAGGCCAGGACTTTGAAAATTTGCGCAACTATCGTATAGTGGCCGACAAATTGAC

GCCGAGTTGACAGACTGCCTAGCATTTGAGTGAATTATGTGAGGTAATGGGCTACACTGAATTGGTAGCT

CAAACTGTCAGTATTTATGTATATGAGTGTATATTTTCGCATAATCTCAGACCAATCTGAAGATGAAATG

GGTATCTGGGAATGGCGAAATCAAGGCATCGATCGTGAAGTTTCTCATCTAAGCCCCCATTTGGACGTGA

ATGTAGACACGTCGAAATAAAGATTTCCGAATTAGAATAATTTGTTTATTGCTTTCGCCTATAAATACGA

CGGATCGTAATTTGTCGTTTTATCAAAATGTACTTTCATTTTATAATAACGCTGCGGACATCTACATTTT

TGAATTGAAAAAAAATTGGTAATTACTCTTTCTTTTTCTCCATATTGACCATCATACTCATTGCTGATCC

ATGTAGATTTCCCGGACATGAAGCCATTTACAATTGAATATATCCTGCC

>gi|23381130|emb|AJ414111.1|ATU414111 Binary vector pGV4223 DNA, region between right and left T-DNA border and LECASAL gene

AATTACAACGGTATATATCCTGCCAGTCAGCATCATCACACCAAAAGTTAGGCCCGAATAGTTTGAAATT

AGAAAGCTCGCAATTGAGGTCTACAGGCCAAATTCGCTCTTAGCCGTACAATATTACTCACCGGTGCGAT

GCCCCCCATCGTAGGTGAAGGTGGAAATTAATGATCCATCTTGAGACCACAGGCCCACAACAGCTACCAG

TTTCCTCAAGGGTCCACCAAAAACGTAAGCGCTTACGTACATGGTCGATAAGAAAAGGCAATTTGTAGAT

GTTAGATCTTCTAGAGTTAACGAATTCCCGATCTAGTAACATAGATGACACCGCGCGCGATAATTTATCC

TAGTTTGCGCGCTATATTTTGTTTTCTATCGCGTATTAAATGTATAATTGCGGGACTCTAATCATAAAAA

CCCATCTCATAAATAACGTCATGCATTACATGTTAATTATTACATGCTTAACGTAATTCAACAGAAATTA

TATGATAATCATCGCAAGACCGGCAACAGGATTCAATCTTAAGAAACTTTATTGCCAAATGTTTGAACGA

TCGGGGAAATTCGAGCTCGGTACCCAATTCGCGGCCGCTTTTTTTTTAACCTTAAAAGAAGGACCATACA

ATATGATTATTGCAGATGCAACCAGCAAGCACATGCAGTGCCAAATATACATAATTAATACAGGATAACA

ACTTACATACATACATAGAGATAGAGTGCATCACTGTCGCACACACAATTCATTATTTAAACCTAAAACA

CATGGAACTTATCTCAAAACTCAAGCAGCACCGCTGCCAACCTTTCGGATAGCTGCAGTGACATTCTGAT

TCACGACTACGGGTCCAATCACGGAGCCTCCATCAACAGTACCATTCATAGCCATGACAACAGCTCCACC

CACACTTCTTCTGTAGGTACCAGTAGACCAAATATCAGATCCGTAAATGACAACGTTTCTGTCCTCCTGA

AGCAGCAGGATATAGTTCCCGTTCCCTCTTACACTGTTACTGGCCCAGACGGGACGCCCGTTAACATCGT

AGACCACAAAGTTGCCATCCCTCTGCATGACAGCCCTGCACCCGTTTTTGCCGGTGACACCGGTGTTAGA

GGCCCAGATGGGGGTGCTGTATTCGTACAGCACGAGGTTGCAGTCATCCTGCATTATAAACTTGTATTGT

TCTACATCCAGGGATTGGCCTGCGTAGAGTCCTTCACCGTTCGTCAGTAGGTTCCTGGCCATGCATGTGG

ATGCCAGTATGGTTAGAATAGCGGCTACAGTGGCTATTCTCATCATTGCTTTAGGAGATGAAGTAGTACG

ACCCATTCTAGCGGCCGCGAATTGGGGATCCTCTAGAGTCCCCCGTGTTCTCTCCAAATGAAATGAACTT

CCTTATATAGAGGAAGGGTCTTGCGAAGGATAGTGGGATTGTGCGTCATCCCTTACGTCAGTGGAGATAT

CACATCAATCCACTTGCTTTGAAGACGTGGTTGGAACGTCTTCTTTTTCCACGATGCTCCTCGTGGGTGG

GGGTCCATCTTTGGGACCACTGTCGGCAGAGGCATCTTCAACGATGGCCTTTCCTTTATCGCAATGATGG

CATTTGTAGGAGCCACCTTCCTTTTCCACTATCTTCACAATAAAGTGACAGATAGCTGGGCAATGGAATC

CGAGGAGGTTTCCCGATATTACCCTTTGTTGAAAAGTCTCAATTGCCCTTTGGTCTTCTGAGACTGTATC

TTTGATATTTTTGGAGTAGACAAGTGTGTCGTGCTCCACCATGTTGACGAAGATTTTCTTCTTGTCATTG

AGTCGTAAGAGACTCTGTATGAACTGTTCGCCAGTCTTTACGGCGAGTTCTGTTAGGTCCTCTATTTGAA

TCTTTGACTCCATGGCCTTTGATTCAGTGGGAACTACCTTTTTAGAGACTCCAATCTCTATTACTTGCCT

TGGTTTGTGAAGCAAGCCTTGAATCGTCCATACTGGAATAGTACTTCTGATCTTGAGAAATATATCTTTC

TCTGTGTTCTTGATGCAGTTAGTCCTGAATCTTTTGACTGCATCTTTAACCTTCTTGGGAAGGTATTTGA

TTTCCTGGAGATTATTGCTCGGGTAGATCGTCTTGATGAGACCTGCTGCGTAAGCCTCTCTAACCATCTG

TGGGTTAGCATTCTTTCTGAAATTGAAAAGGCTAATCTGGGGACCTGCAGGCATGCAAGCTTGATATCGG

ATCCATAGACTAATTTCTAGTGCGGTAGTTATATCTCTTCCAAGAGTGTTCGAGAGGATTGCCCTTTGCC

AAAATGCCTCCACAATTGTGGATAATTGCACAGACGGCGTTACACGCGGTCTGAATAGCACCCTCCACCC

ATCCACCTGTGAAGGAACAACTGCAACCCGCCAAGTAAACTCCGGTATCATTAGCCGTGTCCAGTGCTTG

AAAGAAAAGTTCTTCAGAATAAAAATCCTCACCACGCCGGTTGAGTTTGAAAGCTCCCCCGGCATTCTCG

TCTGTAAGCCAATCATGTTGAATAACATTTTGGTCGTAATCAGCGCAGGCAGGAAATAGGTGCTGGGCAA

ACGCCGGGAAAGATCTCGTCAATTCTCACTCATTAGGCACCCCAGGCTTTACACTTTATGCTTCCGGCTC

GTATAATGTGTGGAATTGTGAGCGGATAACAATTTCACACAGGAAACAGGATCATGAGCGGAGAATTAAG

GGAGTCACGTTATGACCCCCGCCGATGACGCGGGACAAGCCGTTTTACGTTTGGAACTGACAGAACCGCA

ACGTTGAAGGAGCCACTCAGCCGCGGGTTTCTGGAGTTTAATGAGCTAAGCACATACGTCAGAAACCATT

ATTGCGCGTTCAAAAGTCGCCTAAGGTCACTATCAGCTAGCAAATATTTCTTGTCAAAAATGCTCCACTG

ACGTTCCATAAATTCCCCTCGGTATCCAATTAGAGTCTCATATTCACTCTCAATCCAGATCCGGCCCATG

ATCATGTGGATTGAACAAGATGGATTGCACGCAGGTTCTCCGGCCGCTTGGGTGGAGAGGCTATTCGGCT

ATGACTGGGCACAACAGACAATCGGCTGCTCTGATGCCGCCGTGTTCCGGCTGTCAGCGCAGGGGCGCCC

GGTTCTTTTTGTCAAGACCGACCTGTCCGGTGCCCTGAATGAACTGCAGGACGAGGCAGCGCGGCTATCG

TGGCTGGCCACGACGGGCGTTCCTTGCGCAGCTGTGCTCGACGTTGTCACTGAAGCGGGAAGGGACTGGC

TGCTATTGGGCGAAGTGCCGGGGCAGGATCTCCTGTCATCTCACCTTGCTCCTGCCGAGAAAGTATCCAT

CATGGCTGATGCAATGCGGCGGCTGCATACGCTTGATCCGGCTACCTGCCCATTCGACCACCAAGCGAAA

CATCGCATCGAGCGAGCACGTACTCGGATGGAAGCCGGTCTTGTCGATCAGGATGATCTGGACGAAGAGC

ATCAGGGGCTCGCGCCAGCCGAACTGTTCGCCAGGCTCAAGGCGCGCATGCCCGACGGCGAGGATCTCGT

CGTGACCCATGGCGATGCGTGCTTGCCGAATATCATGGTGGAAAATGGCCGCTTTTCTGGATTCATCGAC

TGTGGCCGGCTGGGTGTGGCGGACCGCTATCAGGACATAGCGTTGGCTACCCGTGATATTGCTGAAGAGC

TTGGCGGCGAATGGGCTGACCGCTTCCTCGTGCTTTACGGTATCGCCGCTCCCGATTCGCAGCGCATCGC

CTTCTATCGCCTTCTTGACGAGTTCTTCTGAGCGGGACTCTGGGGTTCGAAATGCCGACCAAGCGACGCC

CAACCTGCCATCACGAGATTTCGATTCCACCGCCGCCTTCTATGAAAGGTTGGGCTTCGGAATCGTTTTC

CGGGACGCCGGCTGGATGATCCTCCAGCGCGGGGATCTCATGCTGGAGTTCTTCGCCCACCCCCTGCTTT

AATGAGATATGCGAGACGCCTATGATCGCATGATATTTGCTTTCAATTCTGTTGTGCACGTTGTAAAAAA

CCTGAGCATGTGTAGCTCAGATCCTTACCGCCGGTTTCGGTTCATTCTAATGAATGAATATATCACCCGT

TACTATCGTATTTTTATGAATAATATTCTCCGTTCAATTTACTGATTGTACCCTACTACTTATATGTACA

ATATTAAAATGAAAACAATATATTGTGCTGAATAGGTTTATAGCGACATCTATGATAGAGCGCCACAATA

ACAAACAATTGCGTTTTATTATTACAAATCCAATTTTAAAAAAAGCGGCAGAACCGGTCAAACCTAAAAG

ACTGATTACATAAATCTTATTCAAATTTCAAAAGGCCCCAGGGGCTAGTATCTACGACACACCGAGCGGC

GAACTAATAACGTTCACTGAAGGGAACTCCGGTTCCCCGCCGGCGCGCATGGGTGAGATTCCTTGAAGTT

GAGTATTGGCCGTCCGCTCTACCGAAAGTTACGGGCACCATTCAACCCGGTCCAGCACGGCGGCCGGGTA

ACCGACTTGCTGCCCCGAGAATTATGCAGCATTTTTTTGGTGTATGTGGGCCCCAAATGAAGTGCAGGTC

AAACCTTGACAGTGACGACAAATCGTTGGGCGGGTCCAGGGCGAATTTTGCGACAACATGTCGAGGCTCA

GCAGGGGCTCGATCCCCTCGCGAGTTGGTTCAGCTGCTGCCTGAGGCTGGACGACCTCGCGGAGTTCTAC

CGGCAGTGCAAATCCGGTCGGCATCCAGGAAACCAGCAGCGGCTATCCGCGCATCCATGCCCCCGAACAG

CAGGAGTGGGGAGGCACGATGGCCGCTTTGGTCGAGACGGATCTTATAATTAAATGGCCTTCGCTGCCCA

TATTATTGGTAACTCAACAGCATCAATCACGGGATTTTTCTCGAATTAATTGCGTCGAATCTCAGCATCG

AAATATTCGCCTTTTTCGTCCATTAGACTATCTATTGTGATGGTGGATTTATCACAAATGGGACCCGCCG

CCGACAGAGGTGTGATGTTAGGCCAGGACTTTGAAAATTTGCGCAACTATCGTATAGTGGCCGACAAATT

GACGCCGAGTTGACAGACTGCCTAGCATTTGAGTGAATTATGTGAGGTAATGGGCTACACTGAATTGGTA

GCTCAAACTGTCAGTATTTATGTATATGAGTGTATATTTTCGCATAATCTCAGACCAATCTGAAGATGAA

ATGGGTATCTGGGAATGGCGAAATCAAGGCATCGATCGTGAAGTTTCTCATCTAAGCCCCCATTTGGACG

TGAATGTAGACACGTCGAAATAAAGATTTCCGAATTAGAATAATTTGTTTATTGCTTTCGCCTATAAATA

CGACGGATCGTAATTTGTCGTTTTATCAAAATGTACTTTCATTTTATAATAACGCTGCGGACATCTACAT

TTTTGAATTGAAAAAAAATTGGTAATTACTCTTTCTTTTTCTCCATATTGACCATCATACTCATTGCTGA

TCCATGTAGATTTCCCGGACATGAAGCCATTTACAATTGAATATATCCTGCC

>gi|23392741|emb|AJ414112.1|ECO414112 Cloning vector pGV1025 DNA, complete sequence

TCGCGCGTTTCGGTGATGACGGTGAAAACCTCTGACACATGCAGCTCCCGGAGACGGTCACAGCTTGTCT

GTAAGCGGATGCCGGGAGCAGACAAGCCCGTCAGGGCGCGTCAGCGGGTGTTGGCGGGTGTCGGGGCTGG

CTTAACTATGCGGCATCAGAGCAGATTGTACTGAGAGTGCACCATATGCGGTGTGAAATACCGCACAGAT

GCGTAAGGAGAAAATACCGCATCAGGCGCCATTCGCCATTCAGGCTGCGCAACTGTTGGGAAGGGCGATC

GGTGCGGGCCTCTTCGCTATTACGCCAGCTGGCGAAAGGGGGATGTGCTGCAAGGCGATTAAGTTGGGTA

ACGCCAGGGTTTTCCCAGTCACGACGTTGTAAAACGACGGCCAGTGCCAAGCTTGCATGCCTGCAGGTCC

CCAGATTAGCCTTTTCAATTTCAGAAAGAATGCTAACCCACAGATGGTTAGAGAGGCTTACGCAGCAGGT

CTCATCAAGACGATCTACCCGAGCAATAATCTCCAGGAAATCAAATACCTTCCCAAGAAGGTTAAAGATG

CAGTCAAAAGATTCAGGACTAACTGCATCAAGAACACAGAGAAAGATATATTTCTCAAGATCAGAAGTAC

TATTCCAGTATGGACGATTCAAGGCTTGCTTCACAAACCAAGGCAAGTAATAGAGATTGGAGTCTCTAAA

AAGGTAGTTCCCACTGAATCAAAGGCCATGGAGTCAAAGATTCAAATAGAGGACCTAACAGAACTCGCCG

TAAAGACTGGCGAACAGTTCATACAGAGTCTCTTACGACTCAATGACAAGAAGAAAATCTTCGTCAACAT

GGTGGAGCACGACACACTTGTCTACTCCAAAAATATCAAAGATACAGTCTCAGAAGACCAAAGGGCAATT

GAGACTTTTCAACAAAGGGTAATATCGGGAAACCTCCTCGGATTCCATTGCCCAGCTATCTGTCACTTTA

TTGTGAAGATAGTGGAAAAGGAAGGTGGCTCCTACAAATGCCATCATTGCGATAAAGGAAAGGCCATCGT

TGAAGATGCCTCTGCCGACAGTGGTCCCAAAGATGGACCCCCACCCACGAGGAGCATCGTGGAAAAAGAA

GACGTTCCAACCACGTCTTCAAAGCAAGTGGATTGATGTGATATCTCCACTGACGTAAGGGATGACGCAC

AATCCCACTATCCTTCGCAAGACCCTTCCTCTATATAAGGAAGTTCATTTCATTTGGAGAGAACACGGGG

GACTCTAGAGGATCCCCGGGTGGTCAGTCCCTTATGTTACGTCCTGTAGAAACCCCAACCCGTGAAATCA

AAAAACTCGACGGCCTGTGGGCATTCAGTCTGGATCGCGAAAACTGTGGAATTGATCAGCGTTGGTGGGA

AAGCGCGTTACAAGAAAGCCGGGCAATTGCTGTGCCAGGCAGTTTTAACGATCAGTTCGCCGATGCAGAT

ATTCGTAATTATGCGGGCAACGTCTGGTATCAGCGCGAAGTCTTTATACCGAAAGGTTGGGCAGGCCAGC

GTATCGTGCTGCGTTTCGATGCGGTCACTCATTACGGCAAAGTGTGGGTCAATAATCAGGAAGTGATGGA

GCATCAGGGCGGCTATACGCCATTTGAAGCCGATGTCACGCCGTATGTTATTGCCGGGAAAAGTGTACGT

ATCACCGTTTGTGTGAACAACGAACTGAACTGGCAGACTATCCCGCCGGGAATGGTGATTACCGACGAAA

ACGGCAAGAAAAAGCAGTCTTACTTCCATGATTTCTTTAACTATGCCGGAATCCATCGCAGCGTAATGCT

CTACACCACGCCGAACACCTGGGTGGACGATATCACCGTGGTGACGCATGTCGCGCAAGACTGTAACCAC

GCGTCTGTTGACTGGCAGGTGGTGGCCAATGGTGATGTCAGCGTTGAACTGCGTGATGCGGATCAACAGG

TGGTTGCAACTGGACAAGGCACTAGCGGGACTTTGCAAGTGGTGAATCCGCACCTCTGGCAACCGGGTGA

AGGTTATCTCTATGAACTGTGCGTCACAGCCAAAAGCCAGACAGAGTGTGATATCTACCCGCTTCGCGTC

GGCATCCGGTCAGTGGCAGTGAAGGGCGAACAGTTCCTGATTAACCACAAACCGTTCTACTTTACTGGCT

TTGGTCGTCATGAAGATGCGGACTTACGTGGCAAAGGATTCGATAACGTGCTGATGGTGCACGACCACGC

ATTAATGGACTGGATTGGGGCCAACTCCTACCGTACCTCGCATTACCCTTACGCTGAAGAGATGCTCGAC

TGGGCAGATGAACATGGCATCGTGGTGATTGATGAAACTGCTGCTGTCGGCTTTAACCTCTCTTTAGGCA

TTGGTTTCGAAGCGGGCAACAAGCCGAAAGAACTGTACAGCGAAGAGGCAGTCAACGGGGAAACTCAGCA

AGCGCACTTACAGGCGATTAAAGAGCTGATAGCGCGTGACAAAAACCACCCAAGCGTGGTGATGTGGAGT

ATTGCCAACGAACCGGATACCCGTCCGCAAGGTGCACGGGAATATTTCGCGCCACTGGCGGAAGCAACGC

GTAAACTCGACCCGACGCGTCCGATCACCTGCGTCAATGTAATGTTCTGCGACGCTCACACCGATACCAT

CAGCGATCTCTTTGATGTGCTGTGCCTGAACCGTTATTACGGATGGTATGTCCAAAGCGGCGATTTGGAA

ACGGCAGAGAAGGTACTGGAAAAAGAACTTCTGGCCTGGCAGGAGAAACTGCATCAGCCGATTATCATCA

CCGAATACGGCGTGGATACGTTAGCCGGGCTGCACTCAATGTACACCGACATGTGGAGTGAAGAGTATCA

GTGTGCATGGCTGGATATGTATCACCGCGTCTTTGATCGCGTCAGCGCCGTCGTCGGTGAACAGGTATGG

AATTTCGCCGATTTTGCGACCTCGCAAGGCATATTGCGCGTTGGCGGTAACAAGAAAGGGATCTTCACTC

GCGACCGCAAACCGAAGTCGGCGGCTTTTCTGCTGCAAAAACGCTGGACTGGCATGAACTTCGGTGAAAA

ACCGCAGCAGGGAGGCAAACAATGAATCAACAACTCTCCTGGCGCACCATCGTCGGCTACAGCCTCGGGA

ATTGCTACCGAGCTCGAATTTCCCCGATCGTTCAAACATTTGGCAATAAAGTTTCTTAAGATTGAATCCT

GTTGCCGGTCTTGCGATGATTATCATATAATTTCTGTTGAATTACGTTAAGCATGTAATAATTAACATGT

AATGCATGACGTTATTTATGAGATGGGTTTTTATGATTAGAGTCCCGCAATTATACATTTAATACGCGAT

AGAAAACAAAATATAGCGCGCAAACTAGGATAAATTATCGCGCGCGGTGTCATCTATGTTACTAGATCGG

GAATTCGTAATCATGGTCATAGCTGTTTCCTGTGTGAAATTGTTATCCGCTCACAATTCCACACAACATA

CGAGCCGGAAGCATAAAGTGTAAAGCCTGGGGTGCCTAATGAGTGAGCTAACTCACATTAATTGCGTTGC

GCTCACTGCCCGCTTTCCAGTCGGGAAACCTGTCGTGCCAGCTGCATTAATGAATCGGCCAACGCGCGGG

GAGAGGCGGTTTGCGTATTGGGCGCTCTTCCGCTTCCTCGCTCACTGACTCGCTGCGCTCGGTCGTTCGG

CTGCGGCGAGCGGTATCAGCTCACTCAAAGGCGGTAATACGGTTATCCACAGAATCAGGGGATAACGCAG

GAAAGAACATGTGAGCAAAAGGCCAGCAAAAGGCCAGGAACCGTAAAAAGGCCGCGTTGCTGGCGTTTTT

CCATAGGCTCCGCCCCCCTGACGAGCATCACAAAAATCGACGCTCAAGTCAGAGGTGGCGAAACCCGACA

GGACTATAAAGATACCAGGCGTTTCCCCCTGGAAGCTCCCTCGTGCGCTCTCCTGTTCCGACCCTGCCGC

TTACCGGATACCTGTCCGCCTTTCTCCCTTCGGGAAGCGTGGCGCTTTCTCAATGCTCACGCTGTAGGTA

TCTCAGTTCGGTGTAGGTCGTTCGCTCCAAGCTGGGCTGTGTGCACGAACCCCCCGTTCAGCCCGACCGC

TGCGCCTTATCCGGTAACTATCGTCTTGAGTCCAACCCGGTAAGACACGACTTATCGCCACTGGCAGCAG

CCACTGGTAACAGGATTAGCAGAGCGAGGTATGTAGGCGGTGCTACAGAGTTCTTGAAGTGGTGGCCTAA

CTACGGCTACACTAGAAGGACAGTATTTGGTATCTGCGCTCTGCTGAAGCCAGTTACCTTCGGAAAAAGA

GTTGGTAGCTCTTGATCCGGCAAACAAACCACCGCTGGTAGCGGTGGTTTTTTTGTTTGCAAGCAGCAGA

TTACGCGCAGAAAAAAAGGATCTCAAGAAGATCCTTTGATCTTTTCTACGGGGTCTGACGCTCAGTGGAA

CGAAAACTCACGTTAAGGGATTTTGGTCATGAGATTATCAAAAAGGATCTTCACCTAGATCCTTTTAAAT

TAAAAATGAAGTTTTAAATCAATCTAAAGTATATATGAGTAAACTTGGTCTGACAGTTACCAATGCTTAA

TCAGTGAGGCACCTATCTCAGCGATCTGTCTATTTCGTTCATCCATAGTTGCCTGACTCCCCGTCGTGTA

GATAACTACGATACGGGAGGGCTTACCATCTGGCCCCAGTGCTGCAATGATACCGCGAGACCCACGCTCA

CCGGCTCCAGATTTATCAGCAATAAACCAGCCAGCCGGAAGGGCCGAGCGCAGAAGTGGTCCTGCAACTT

TATCCGCCTCCATCCAGTCTATTAATTGTTGCCGGGAAGCTAGAGTAAGTAGTTCGCCAGTTAATAGTTT

GCGCAACGTTGTTGCCATTGCTACAGGCATCGTGGTGTCACGCTCGTCGTTTGGTATGGCTTCATTCAGC

TCCGGTTCCCAACGATCAAGGCGAGTTACATGATCCCCCATGTTGTGCAAAAAAGCGGTTAGCTCCTTCG

GTCCTCCGATCGTTGTCAGAAGTAAGTTGGCCGCAGTGTTATCACTCATGGTTATGGCAGCACTGCATAA

TTCTCTTACTGTCATGCCATCCGTAAGATGCTTTTCTGTGACTGGTGAGTACTCAACCAAGTCATTCTGA

GAATAGTGTATGCGGCGACCGAGTTGCTCTTGCCCGGCGTCAACACGGGATAATACCGCGCCACATAGCA

GAACTTTAAAAGTGCTCATCATTGGAAAACGTTCTTCGGGGCGAAAACTCTCAAGGATCTTACCGCTGTT

GAGATCCAGTTCGATGTAACCCACTCGTGCACCCAACTGATCTTCAGCATCTTTTACTTTCACCAGCGTT

TCTGGGTGAGCAAAAACAGGAAGGCAAAATGCCGCAAAAAAGGGAATAAGGGCGACACGGAAATGTTGAA

TACTCATACTCTTCCTTTTTCAATATTATTGAAGCATTTATCAGGGTTATTGTCTCATGAGCGGATACAT

ATTTGAATGTATTTAGAAAAATAAACAAATAGGGGTTCCGCGCACATTTCCCCGAAAAGTGCCACCTGAC

GTCTAAGAAACCATTATTATCATGACATTAACCTATAAAAATAGGCGTATCACGAGGCCCTTTCGTC

>gi|23392743|emb|AJ414113.1|ECO414113 Cloning vector pGV4112 DNA, complete sequence

TCGCGCGTTTCGGTGATGACGGTGAAAACCTCTGACACATGCAGCTCCCGGAGACGGTCACAGCTTGTCT

GTAAGCGGATGCCGGGAGCAGACAAGCCCGTCAGGGCGCGTCAGCGGGTGTTGGCGGGTGTCGGGGCTGG

CTTAACTATGCGGCATCAGAGCAGATTGTACTGAGAGTGCACCATATGCGGTGTGAAATACCGCACAGAT

GCGTAAGGAGAAAATACCGCATCAGGCGCCATTCGCCATTCAGGCTGCGCAACTGTTGGGAAGGGCGATC

GGTGCGGGCCTCTTCGCTATTACGCCAGCTGGCGAAAGGGGGATGTGCTGCAAGGCGATTAAGTTGGGTA

ACGCCAGGGTTTTCCCAGTCACGACGTTGTAAAACGACGGCCAGTGCCAAGCTTGCATGCCTGCAGGTCC

CCAGATTAGCCTTTTCAATTTCAGAAAGAATGCTAACCCACAGATGGTTAGAGAGGCTTACGCAGCAGGT

CTCATCAAGACGATCTACCCGAGCAATAATCTCCAGGAAATCAAATACCTTCCCAAGAAGGTTAAAGATG

CAGTCAAAAGATTCAGGACTAACTGCATCAAGAACACAGAGAAAGATATATTTCTCAAGATCAGAAGTAC

TATTCCAGTATGGACGATTCAAGGCTTGCTTCACAAACCAAGGCAAGTAATAGAGATTGGAGTCTCTAAA

AAGGTAGTTCCCACTGAATCAAAGGCCATGGAGTCAAAGATTCAAATAGAGGACCTAACAGAACTCGCCG

TAAAGACTGGCGAACAGTTCATACAGAGTCTCTTACGACTCAATGACAAGAAGAAAATCTTCGTCAACAT

GGTGGAGCACGACACACTTGTCTACTCCAAAAATATCAAAGATACAGTCTCAGAAGACCAAAGGGCAATT

GAGACTTTTCAACAAAGGGTAATATCGGGAAACCTCCTCGGATTCCATTGCCCAGCTATCTGTCACTTTA

TTGTGAAGATAGTGGAAAAGGAAGGTGGCTCCTACAAATGCCATCATTGCGATAAAGGAAAGGCCATCGT

TGAAGATGCCTCTGCCGACAGTGGTCCCAAAGATGGACCCCCACCCACGAGGAGCATCGTGGAAAAAGAA

GACGTTCCAACCACGTCTTCAAAGCAAGTGGATTGATGTGATATCTCCACTGACGTAAGGGATGACGCAC

AATCCCACTATCCTTCGCAAGACCCTTCCTCTATATAAGGAAGTTCATTTCATTTGGAGAGAACACGGGG

GACTCTAGAGGATCCCCGGGTACCGAGCTCGAATTTCCCCGATCGTTCAAACATTTGGCAATAAAGTTTC

TTAAGATTGAATCCTGTTGCCGGTCTTGCGATGATTATCATATAATTTCTGTTGAATTACGTTAAGCATG

TAATAATTAACATGTAATGCATGACGTTATTTATGAGATGGGTTTTTATGATTAGAGTCCCGCAATTATA

CATTTAATACGCGATAGAAAACAAAATATAGCGCGCAAACTAGGATAAATTATCGCGCGCGGTGTCATCT

ATGTTACTAGATCGGGAATTCGTAATCATGGTCATAGCTGTTTCCTGTGTGAAATTGTTATCCGCTCACA

ATTCCACACAACATACGAGCCGGAAGCATAAAGTGTAAAGCCTGGGGTGCCTAATGAGTGAGCTAACTCA

CATTAATTGCGTTGCGCTCACTGCCCGCTTTCCAGTCGGGAAACCTGTCGTGCCAGCTGCATTAATGAAT

CGGCCAACGCGCGGGGAGAGGCGGTTTGCGTATTGGGCGCTCTTCCGCTTCCTCGCTCACTGACTCGCTG

CGCTCGGTCGTTCGGCTGCGGCGAGCGGTATCAGCTCACTCAAAGGCGGTAATACGGTTATCCACAGAAT

CAGGGGATAACGCAGGAAAGAACATGTGAGCAAAAGGCCAGCAAAAGGCCAGGAACCGTAAAAAGGCCGC

GTTGCTGGCGTTTTTCCATAGGCTCCGCCCCCCTGACGAGCATCACAAAAATCGACGCTCAAGTCAGAGG

TGGCGAAACCCGACAGGACTATAAAGATACCAGGCGTTTCCCCCTGGAAGCTCCCTCGTGCGCTCTCCTG

TTCCGACCCTGCCGCTTACCGGATACCTGTCCGCCTTTCTCCCTTCGGGAAGCGTGGCGCTTTCTCAATG

CTCACGCTGTAGGTATCTCAGTTCGGTGTAGGTCGTTCGCTCCAAGCTGGGCTGTGTGCACGAACCCCCC

GTTCAGCCCGACCGCTGCGCCTTATCCGGTAACTATCGTCTTGAGTCCAACCCGGTAAGACACGACTTAT

CGCCACTGGCAGCAGCCACTGGTAACAGGATTAGCAGAGCGAGGTATGTAGGCGGTGCTACAGAGTTCTT

GAAGTGGTGGCCTAACTACGGCTACACTAGAAGGACAGTATTTGGTATCTGCGCTCTGCTGAAGCCAGTT

ACCTTCGGAAAAAGAGTTGGTAGCTCTTGATCCGGCAAACAAACCACCGCTGGTAGCGGTGGTTTTTTTG

TTTGCAAGCAGCAGATTACGCGCAGAAAAAAAGGATCTCAAGAAGATCCTTTGATCTTTTCTACGGGGTC

TGACGCTCAGTGGAACGAAAACTCACGTTAAGGGATTTTGGTCATGAGATTATCAAAAAGGATCTTCACC

TAGATCCTTTTAAATTAAAAATGAAGTTTTAAATCAATCTAAAGTATATATGAGTAAACTTGGTCTGACA

GTTACCAATGCTTAATCAGTGAGGCACCTATCTCAGCGATCTGTCTATTTCGTTCATCCATAGTTGCCTG

ACTCCCCGTCGTGTAGATAACTACGATACGGGAGGGCTTACCATCTGGCCCCAGTGCTGCAATGATACCG

CGAGACCCACGCTCACCGGCTCCAGATTTATCAGCAATAAACCAGCCAGCCGGAAGGGCCGAGCGCAGAA

GTGGTCCTGCAACTTTATCCGCCTCCATCCAGTCTATTAATTGTTGCCGGGAAGCTAGAGTAAGTAGTTC

GCCAGTTAATAGTTTGCGCAACGTTGTTGCCATTGCTACAGGCATCGTGGTGTCACGCTCGTCGTTTGGT

ATGGCTTCATTCAGCTCCGGTTCCCAACGATCAAGGCGAGTTACATGATCCCCCATGTTGTGCAAAAAAG

CGGTTAGCTCCTTCGGTCCTCCGATCGTTGTCAGAAGTAAGTTGGCCGCAGTGTTATCACTCATGGTTAT

GGCAGCACTGCATAATTCTCTTACTGTCATGCCATCCGTAAGATGCTTTTCTGTGACTGGTGAGTACTCA

ACCAAGTCATTCTGAGAATAGTGTATGCGGCGACCGAGTTGCTCTTGCCCGGCGTCAACACGGGATAATA

CCGCGCCACATAGCAGAACTTTAAAAGTGCTCATCATTGGAAAACGTTCTTCGGGGCGAAAACTCTCAAG

GATCTTACCGCTGTTGAGATCCAGTTCGATGTAACCCACTCGTGCACCCAACTGATCTTCAGCATCTTTT

ACTTTCACCAGCGTTTCTGGGTGAGCAAAAACAGGAAGGCAAAATGCCGCAAAAAAGGGAATAAGGGCGA

CACGGAAATGTTGAATACTCATACTCTTCCTTTTTCAATATTATTGAAGCATTTATCAGGGTTATTGTCT

CATGAGCGGATACATATTTGAATGTATTTAGAAAAATAAACAAATAGGGGTTCCGCGCACATTTCCCCGA

AAAGTGCCACCTGACGTCTAAGAAACCATTATTATCATGACATTAACCTATAAAAATAGGCGTATCACGA

GGCCCTTTCGTC

>gi|24850291|gb|AY147202.1| Binary vector pGV4939 kanamycin resistance protein and ribosome-inactivating protein genes, complete cds

GGCAGGATATATTCAATTGTAAATGGCTTCATGTCCGGGAAATCTACATGGATCAGCAATGAGTATGATG

GTCAATATGGAGAAAAAGAAAGAGTAATTACCAATTTTTTTTCAATTCAAAAATGTAGATGTCCGCAGCG

TTATTATAAAATGAAAGTACATTTTGATAAAACGACAAATTACGATCCGTCGTATTTATAGGCGAAAGCA

ATAAACAAATTATTCTAATTCGGAAATCTTTATTTCGACGTGTCTACATTCACGTCCAAATGGGGGCTTA

GATGAGAAACTTCACGATCGATGCCTTGATTTCGCCATTCCCAGATACCCATTTCATCTTCAGATTGGTC

TGAGATTATGCGAAAATATACACTCATATACATAAATACTGACAGTTTGAGCTACCAATTCAGTGTAGCC

CATTACCTCACATAATTCACTCAAATGCTAGGCAGTCTGTCAACTCGGCGTCAATTTGTCGGCCACTATA

CGATAGTTGCGCAAATTTTCAAAGTCCTGGCCTAACATCACACCTCTGTCGGCGGCGGGTCCCATTTGTG

ATAAATCCACCATCACAATAGATAGTCTAATGGACGAAAAAGGCGAATATTTCGATGCTGAGATTCGACG

CAATTAATTCGAGAAAAATCCCGTGATTGATGCTGTTGAGTTACCAATAATATGGGCAGCGAAGGCCATT

TAATTATAAGATCCGTCTCGACCAAAGCGGCCATCGTGCCTCCCCACTCCTGCTGTTCGGGGGCATGGAT

GCGCGGATAGCCGCTGCTGGTTTCCTGGATGCCGACCGGATTTGCACTGCCGGTAGAACTCCGCGAGGTC

GTCCAGCCTCAGGCAGCAGCTGAACCAACTCGCGAGGGGATCGAGCCCCTGCTGAGCCTCGACATGTTGT

CGCAAAATTCGCCCTGGACCCGCCCAACGATTTGTCGTCACTGTCAAGGTTTGACCTGCACTTCATTTGG

GGCCCACATACACCAAAAAAATGCTGCATAATTCTCGGGGCAGCAAGTCGGTTACCCGGCCGCCGTGCTG

GACCGGGTTGAATGGTGCCCGTAACTTTCGGTAGAGCGGACGGCCAATACTCAACTTCAAGGAATCTCAC

CCATGCGCGCCGGCGGGGAACCGGAGTTCCCTTCAGTGAACGTTATTAGTTCGCCGCTCGGTGTGTCGTA

GATACTAGCCCCTGGGGCCTTTTGAAATTTGAATAAGATTTATGTAATCAGTCTTTTAGGTTTGACCGGT

TCTGCCGCTTTTTTTAAAATTGGATTTGTAATAATAAAACGCAATTGTTTGTTATTGTGGCGCTCTATCA

TAGATGTCGCTATAAACCTATTCAGCACAATATATTGTTTTCATTTTAATATTGTACATATAAGTAGTAG

GGTACAATCAGTAAATTGAACGGAGAATATTATTCATAAAAATACGATAGTAACGGGTGATATATTCATT

CATTAGAATGAACCGAAACCGGCGGTAAGGATCTGAGCTACACATGCTCAGGTTTTTTACAACGTGCACA

ACAGAATTGAAAGCAAATATCATGCGATCATAGGCGTCTCGCATATCTCATTAAAGCAGGGGGTGGGCGA

AGAACTCCAGCATGAGATCCCCGCGCTGGAGGATCATCCAGCCGGCGTCCCGGAAAACGATTCCGAAGCC

CAACCTTTCATAGAAGGCGGCGGTGGAATCGAAATCTCGTGATGGCAGGTTGGGCGTCGCTTGGTCGGCA

TTTCGAACCCCAGAGTCCCGCTCAGAAGAACTCGTCAAGAAGGCGATAGAAGGCGATGCGCTGCGAATCG

GGAGCGGCGATACCGTAAAGCACGAGGAAGCGGTCAGCCCATTCGCCGCCAAGCTCTTCAGCAATATCAC

GGGTAGCCAACGCTATGTCCTGATAGCGGTCCGCCACACCCAGCCGGCCACAGTCGATGAATCCAGAAAA

GCGGCCATTTTCCACCATGATATTCGGCAAGCAGGCATCGCCATGGGTCACGACGAGATCCTCGCCGTCG

GGCATGCGCGCCTTGAGCCTGGCGAACAGTTCGGCTGGCGCGAGCCCCTGATGCTCTTCGTCCAGATCAT

CCTGATCGACAAGACCGGCTTCCATCCGAGTACGTGCTCGCTCGATGCGATGTTTCGCTTGGTGGTCGAA

TGGGCAGGTAGCCGGATCAAGCGTATGCAGCCGCCGCATTGCATCAGCCATGATGGATACTTTCTCGGCA

GGAGCAAGGTGAGATGACAGGAGATCCTGCCCCGGCACTTCGCCCAATAGCAGCCAGTCCCTTCCCGCTT

CAGTGACAACGTCGAGCACAGCTGCGCAAGGAACGCCCGTCGTGGCCAGCCACGATAGCCGCGCTGCCTC

GTCCTGCAGTTCATTCAGGGCACCGGACAGGTCGGTCTTGACAAAAAGAACCGGGCGCCCCTGCGCTGAC

AGCCGGAACACGGCGGCATCAGAGCAGCCGATTGTCTGTTGTGCCCAGTCATAGCCGAATAGCCTCTCCA

CCCAAGCGGCCGGAGAACCTGCGTGCAATCCATCTTGTTCAATCCACATGATCATGGGCCGGATCTGGAT

TGAGAGTGAATATGAGACTCTAATTGGATACCGAGGGGAATTTATGGAACGTCAGTGGAGCATTTTTGAC

AAGAAATATTTGCTAGCTGATAGTGACCTTAGGCGACTTTTGAACGCGCAATAATGGTTTCTGACGTATG

TGCTTAGCTCATTAAACTCCAGAAACCCGCGGCTGAGTGGCTCCTTCAACGTTGCGGTTCTGTCAGTTCC

AAACGTAAAACGGCTTGTCCCGCGTCATCGGCGGGGGTCATAACGTGACTCCCTTAATTCTCCGCTCATG

ATCCTGTTTCCTGTGTGAAATTGTTATCCGCTCACAATTCCACACATTATACGAGCCGGAAGCATAAAGT

GTAAAGCCTGGGGTGCCTAATGAGTGAGAATTGACGAGATCTTTCCCGGCGTTTGCCCAGCACCTATTTC

CTGCCTGCGCTGATTACGACCAAAATGTTATTCAACATGATTGGCTTACAGACGAGAATGCCGGGGGAGC

TTTCAAACTCAACCGGCGTGGTGAGGATTTTTATTCTGAAGAACTTTTCTTTCAAGCACTGGACACGGCT

AATGATACCGGAGTTTACTTGGCGGGTTGCAGTTGTTCCTTCACAGGTGGATGGGTGGAGGGTGCTATTC

AGACCGCGTGTAACGCCGTCTGTGCAATTATCCACAATTGTGGAGGCATTTTGGCAAAGGGCAATCCTCT

CGAACACTCTTGGAAGAGATATAACTACCGCACTAGAAATTAGTCTATGGATCCGATATCAAGCTTGCAT

GCCTGCAGGTCCCCAGATTAGCCTTTTCAATTTCAGAAAGAATGCTAACCCACAGATGGTTAGAGAGGCT

TACGCAGCAGGTCTCATCAAGACGATCTACCCGAGCAATAATCTCCAGGAAATCAAATACCTTCCCAAGA

AGGTTAAAGATGCAGTCAAAAGATTCAGGACTAACTGCATCAAGAACACAGAGAAAGATATATTTCTCAA

GATCAGAAGTACTATTCCAGTATGGACGATTCAAGGCTTGCTTCACAAACCAAGGCAAGTAATAGAGATT

GGAGTCTCTAAAAAGGTAGTTCCCACTGAATCAAAGGCCATGGAGTCAAAGATTCAAATAGAGGACCTAA

CAGAACTCGCCGTAAAGACTGGCGAACAGTTCATACAGAGTCTCTTACGACTCAATGACAAGAAGAAAAT

CTTCGTCAACATGGTGGAGCACGACACACTTGTCTACTCCAAAAATATCAAAGATACAGTCTCAGAAGAC

CAAAGGGCAATTGAGACTTTTCAACAAAGGGTAATATCGGGAAACCTCCTCGGATTCCATTGCCCAGCTA

TCTGTCACTTTATTGTGAAGATAGTGGAAAAGGAAGGTGGCTCCTACAAATGCCATCATTGCGATAAAGG

AAAGGCCATCGTTGAAGATGCCTCTGCCGACAGTGGTCCCAAAGATGGACCCCCACCCACGAGGAGCATC

GTGGAAAAAGAAGACGTTCCAACCACGTCTTCAAAGCAAGTGGATTGATGTGATATCTCCACTGACGTAA

GGGATGACGCACAATCCCACTATCCTTCGCAAGACCCTTCCTCTATATAAGGAAGTTCATTTCATTTGGA

GAGAACACGGGGGACTCTAGAGGATCCCCAATTCGCCCTTATGAGAGTGGTAGCTGCAATATTTTACATT

AATGTAGTTGTGGCATTAATATGCGGCCTTGGAATCCAAGGCGGCGCCCTGGACCTGCAGGACTATCCCT

CCGTCTCCTTCCAAGGCGACGCCATGCAACTGCAGGACTATCCCTCCATCACCTTCAATTTGAAGGGTGC

CACCTGGCAGACATACAGAGACTTCATAGAGAAACTGCGAGAGATTGTGACCCGGGGAGCCACTACCATT

GCTGGTACTTCTATACCAGTGCTGAACCGTGTAGTGCCCGATTCGAGGCGGTTCGTTTATGTCCGTCTCA

TCAATCTCGACGGCAACGTCGTGACCATTGCAGTAGATGTGACGAGCCTTTATGTGGTTGCTTTTAGCGC

AAATAACGTAGGCTACTTTTTCAGTGACTCTACGGAAACTGAACGCACAAATTTATTCGTCGGCATTACA

CATGACAAACCTTTGGGTTTCACCAGCAACTATAACAGCCTTGAGAACTGGGCAGGAGCTGACAGGGGAT

CTATCCCTCTGGGACCCGCTCTCCTAAATAAGGCCATCAGAGATTTGAGAAGCAATGGAAGAGATAACGA

TGCAGCCAAATCGCTCATTGTGGTCATTCAGATGGTGTCGGAAGCGGCAAGGTTCAGACGCATTGAGGAA

CAAGTCCGCAGAAGCATAGCCGATCAAGACACTTTTACACCAGGCAGTTTGATGATTACCATGAAAAAAA

AATGGTCGAAAATGTCCCAGCAGGTCGAGAGGTCCGTAAACGATCAAGGGATCTTCACCGGGATTTTCAC

CAGGACCGTTCAACTTATAGACGACAATCTCCAGACCCTCAACATAGACAACTTTAATGCACTCAGTTTA

CATACCATGCTAGCGATTCTTCTCTTCCGATGCCGTACGACAACTAGTAGCCACAATACCTTGCCCGCTG

CATCTAACATTGTTCTTATGGGCGAGGACTATGTCGACAAAGATGACGAAAAGTGTACGGTAGGAGAGCC

CACAAGACGCATCAGCGGTCGGGCCGGATGGTGTGTGGACGTGAAGGACGGGAGAGACAACGATGGTAAT

CCAATCCAGGTGTTGTCATGTGGTGATGGACAAGAAAGGAAGCAGCAGTGGACGTTCCACAGAGACGGAA

CAATCCGGTCGAAGCTGGGTAAATGCATGACTGCCTACGGGTTTAAGCATGGAGAGTATGTGATGATATA

CGACTGCGATACGGCTATTGCAGGCGCCAACAAGTGGGTAGTATCGATTGACGGCACCATCACCAATCCT

ATATCTGGACTGGTCCTCACAGCTCCTCGAGGTGCAACAGGCACCACCCTGTTGGTGGAGAAGAATGTCC

ACGCCGCTAGACAGTGTTGGCGTGTGGGAGATGATGTAGAGCCCATCGTTACTAAAATTGTGGGGTTTCA

AGAGAAGTGCTTGGAGGCTAACTATCTTGAAAACACTAATGTATCAAGATACACTAAGGTATTTCTGGAC

GATTGTGTGCTCGACAGGCAGCAGCAACGGTGGGCACTCTATAGTGACGGCACCATCCGAGCAGATAGTG

ATCGTAGCCTATGCGTGGCCGACGATGGCCACAGATCCCTTGATTTCATCATCATTCTTGCGTGCAAAGG

ATGGGGCAACCAGCGCTGGGTGTTCAACACCGACGGCACCATCTTAAACCCAAATGCTAAACTAGTTATG

GACGTCAAGGACAGCGATGTCTCTCTTCTGCAGATCATTCTCCACCAATCAACTGGGAAGCCTAACCAGA

AGTGGTTGACAGTGACACTCCCCCGCACAATAAGTTAGAGGGCGAATTGGGTACCGAGCTCGAATTTCCC

CGATCGTTCAAACATTTGGCAATAAAGTTTCTTAAGATTGAATCCTGTTGCCGGTCTTGCGATGATTATC

ATATAATTTCTGTTGAATTACGTTAAGCATGTAATAATTAACATGTAATGCATGACGTTATTTATGAGAT

GGGTTTTTATGATTAGAGTCCCGCAATTATACATTTAATACGCGATAGAAAACAAAATATAGCGCGCAAA

CTAGGATAAATTATCGCGCGCGGTGTCATCTATGTTACTAGATCGGGAATTCGTTGATCTAACATCTACA

AATTGCCTTTTCTTATCGACCATGTACGTAAGCGCTTACGTTTTTGGTGGACCCTTGAGGAAACTGGTAG

CTGTTGTGGGCCTGTGGTCTCAAGATGGATCATTAATTTCCACCTTCACCTACGATGGGGGGCATCGCAC

CGGTGAGTAATATTGTACGGCTAAGAGCGAATTTGGCCTGTAGACCTCAATTGCGAGCTTTCTAATTTCA

AACTATTCGGGCCTAACTTTTGGTGTGATGATGCTGACTGGCAGGATATATACCGTTGTAATT

>gi|27524897|emb|AJ315956.1|BCL315956 Binary Cloning vector pAC161

GAATTCGTAATCATGTCATAGCTGTTTCCTGTGTGAAATTGTTATCCGCTCACAATTCCACACAACATAC

GAGCCGGAAGCATAAAGTGTAAAGCCTGGGGTGCCTAATGAGTGAGCTAACTCACATTAATTGCGTTGCG

CTCACTGCCCGCTTTCCAGTCGGGAAACCTGTCGTGCCAGCTGCATTAATGAATCGGCCAACGCGCGGGG

AGAGGCGGTTTGCGTATTGGGCGCTCTTCCGCTTCCTCGCTCACTGACTCGCTGCGCTCGGTCGTTCGGC

TGCGGCGAGCGGTATCAGCTCACTCAAAGGCGGTAATACGGTTATCCACAGAATCAGGGGATAACGCAGG

AAAGAACATGTGAGCAAAAGGCCAGCAAAAGGCCAGGAACCGTAAAAAGGCCGCGTTGCTGGCGTTTTTC

CATAGGCTCCGCCCCCCTGACGAGCATCACAAAAATCGACGCTCAAGTCAGAGGTGGCGAAACCCGACAG

GACTATAAAGATACCAGGCGTTTCCCCCTGGAAGCTCCCTCGTGCGCTCTCCTGTTCCGACCCTGCCGCT

TACCGGATACCTGTCCGCCTTTCTCCCTTCGGGAAGCGTGGCGCTTTCTCATAGCTCACGCTGTAGGTAT

CTCAGTTCGGTGTAGGTCGTTCGCTCCAAGCTGGGCTGTGTGCACGAACCCCCCGTTCAGCCCGACCGCT

GCGCCTTATCCGGTAACTATCGTCTTGAGTCCAACCCGGTAAGACACGACTTATCGCCACTGGCAGCAGC

CACTGGTAACAGGATTAGCAGAGCGAGGTATGTAGGCGGTGCTACAGAGTTCTTGAAGTGGTGGCCTAAC

TACGGCTACACTAGAAGAACAGTATTTGGTATCTGCGCTCTGCTGAAGCCAGTTACCTTCGGAAAAAGAG

TTGGTAGCTCTTGATCCGGCAAACAAACCACCGCTGGTAGCGGTGGTTTTTTTGTTTGCAAGCAGCAGAT

TACGCGCAGAAAAAAAGGATCTCAAGAAGATCCTTTGATCTTTTCTACGGGGTCTGACGCTCAGTGGAAC

GAAAACTCACGTTAAGGGATTTTGGTCATGAGATTATCAAAAAGGATCTTCACCTAGATCCTTTTAAATT

AAAAATGAAGTTTTAAATCAATCTAAAGTATATATGAGTAAACTTGGTCTGACAGTTACCAATGCTTAAT

CAGTGAGGCACCTATCTCAGCGATCTGTCTATTTCGTTCATCCATAGTTGCCTGACTCCCCGTCGTGTAG

ATAACTACGATACGGGAGGGCTTACCATCTGGCCCCAGTGCTGCAATGATACCGCGAGACCCACGCTCAC

CGGCTCCAGATTTATCAGCAATAAACCAGCCAGCCGGAAGGGCCGAGCGCAGAAGTGGTCCTGCAACTTT

ATCCGCCTCCATCCAGTCTATTAATTGTTGCCGGGAAGCTAGAGTAAGTAGTTCGCCAGTTAATAGTTTG

CGCAACGTTGTTGCCATTGCTACAGGCATCGTGGTGTCACGCTCGTCGTTTGGTATGGCTTCATTCAGCT

CCGGTTCCCAACGATCAAGGCGAGTTACATGATCCCCCATGTTGTGCAAAAAAGCGGTTAGCTCCTTCGG

TCCTCCGATCGTTGTCAGAAGTAAGTTGGCCGCAGTGTTATCACTCATGGTTATGGCAGCACTGCATAAT

TCTCTTACTGTCATGCCATCCGTAAGATGCTTTTCTGTGACTGGTGAGTACTCAACCAAGTCATTCTGAG

AATAGTGTATGCGGCGACCGAGTTGCTCTTGCCCGGCGTCAATACGGGATAATACCGCGCCACATAGCAG

AACTTTAAAAGTGCTCATCATTGGAAAACGTTCTTCGGGGCGAAAACTCTCAAGGATCTTACCGCTGTTG

AGATCCAGTTCGATGTAACCCACTCGTGCACCCAACTGATCTTCAGCATCTTTTACTTTCACCAGCGTTT

CTGGGTGAGCAAAAACAGGAAGGCAAAATGCCGCAAAAAAGGGAATAAGGGCGACACGGAAATGTTGAAT

ACTCATACTCTTCCTTTTTCAATATTATTGAAGCATTTATCAGGGTTATTGTCTCATGAGCGGATACATA

TTTGAATGTATTTAGAAAAATAAACAAATAGGGGTTCCGCGCACATTTCCCCGAAAAGTGCCACCTGACG

TCTAAGAAACCATTATTATCATGACATTAACCTATAAAAATAGGCGTATCACGAGGCCCTTTCGTCTCGC

GCGTTTCGGTGATGACGGTGAAAACCTCTGACACATGCAGCTCCCGGAGACGGTCACAGCTTGTCTGTAA

GCGGATGCCGGGAGCAGACAAGCCCGTCAGGGCGCGTCAGCGGGTGTTGGCGGGTGTCGGGGCTGGCTTA

ACTATGCGGCATCAGAGCAGATTGTACTGAGAGTGCACCATATGCGGTGTGAAATACCGCACAGATGCGT

AAGGAGAAAATACCGCATCAGGCGCCATTCGCCATTCAGGCTGCGCAACTGTTGGGAAGGGCGATCGGTG

CGGGCCTCTTCGCTATTACGCCAGCTGGCGAAAGGGGGATGTGCTGCAAGGCGATTAAGTTGGGTAACGC

CAGGGTTTTCCCAGTCACGACGTTGTAAAACGACGGCCAGTGCCAAGCTTGCATGTCGACATGCATGCAT

GTCGACATGCATGCATGTCGACTCTAGAGGATCCCCAATTCCCATGGAGTCAAAGATTCAAATAGAGGAC

CTAACAGAACTCGCCGTAAAGACTGGCGAACAGTTCATACAGAGTCTCTTACGACTCAATGACAAGAAGA

AAATCTTCGTCAACATGGTGGAGCACGACACGCTTGTCTACTCCAAAAATATCAAAGATACAGTCTCAGA

AGACCAAAGGGCAATTGAGACTTTTCAACAAAGGGTAATATCCGGAAACCTCCTCGGATTCCATTGCCCA

GCTATCTGTCACTTTATTGTGAAGATAGTGGAAAAGGAAGGTGGCTCCTACAAATGCCATCATTGCGATA

AAGGAAAGGCCATCGTTGAAGATGCCTCTGCCGACAGTGGTCCCAAAGATGGACCCCCACCCACGAGGAG

CATCGTGGAAAAAGAAGACGTTCCAACCACGTCTTCAAAGCAAGTGGATTGATGTGATATCTCCACTGAC

GTAAGGGATGACGCACAATCCCACTATCCTTCGCAAGACCCTTCCTCTATATAAGGAAGTTCATTTCATT

TGGAGAGGACAGGGTACCCGGGGATCAGATTGTCGTTTCCCGCCTTCGGTTTAAACTATCAGTGTTTGAC

AGGATATATTGGCGGGTAAACCTAAGAGAAAAGAGCGTTTATTAGAATAATCGGATATTTAAAAGGGCGT

GAAAAGGTTTATCCGTTCGTCCATTTGTATGTGCATGCCAACCACAGGGTTCCCCTCGGGAGTGCTTGGC

ATTCCGTGCGATAATGACTTCTGTTCAACCACCCAAACGTCGGAAAGCCTGACGACGGAGCAGCATTCCA

AAAAGATCCCTTGGCTCGTCTGGGTCGGCTAGAAGGTCGAGTGGGCTGCTGTGGCTTGATCCCTCAACGC

GGTCGCGGACGTAGCGCAGCGCCGAAAAATCCTCGATCGCAAATCCGACGCTGTCGAAAAGCGTGATCTG

CTTGTCGCTCTTTCGGCCGACGTCCTGGCCAGTCATCACGCGCCAAAGTTCCGTCACAGGATGATCTGGC

GCGAGTTGCTGGATCTCGCCTTCAATCCGGGTCTGTGGCGGGAACTCCACGAAAATATCCGAACGCAGCA

AGATATCGCGGTGCATCTCGGTCTTGCCTGGGCAGTCGCCGCCGACGCCGTTGATGTGGACGCCGGGCCC

GATCATATTGTCGCTCAGGATCGTGGCGTTGTGCTTGTCGGCCGTTGCTGTCGTAATGATATCGGCACCT

TCGACCGCCTGTTCCGCAGAGGTGCAGGCCTCGATCTGAAACCCGAACCGCTGGAGATTGCGGGAGCAGC

GAGCAGTAGCCTCGGGGTCGATGTCGTAAAGTCGTATCCGATCGACGCCGATCAGCGCCTTGAAGGCCAA

AGCCTGGAACTCACTTTGGGCACCGTTGCCGATCAGCGCCATCGTGCGCGAATCTTTACGGGCCAGATAC

TTTGCCGCGATCGCGGAGGTCGCGGCCGTTCGCAAGGCCGTCAGGATTGTCATTTCCGACAGCAGCAGCG

GATAGCCGCTATCGACATCGGAGAGCACGCCGAACGCGGTTACCTAGAGCGGCCGCCACCGCGGTGCCTT

GATGTGGGCGCCGGCGGTCGAGTGGCGACGGCGCGGCTTGTCCGCGCCCTGGTAGATTGCCTGGCCGTAG

GCCAGCCATTTTTGAGCGGCCAGCGGCCGCGATAGGCCGACGCGAAGCGGCGGGGCGTAGGGAGCGCAGC

GACCGAAGGGTAGGCGCTTTTTGCAGCTCTTCGGCTGTGCGCTGGCCAGACAGTTATGCACAGGCCAGGC

GGGTTTTAAGAGTTTTAATAAGTTTTAAAGAGTTTTAGGCGGAAAAATCGCCTTTTTTCTCTTTTATATC

AGTCACTTACATGTGTGACCGGTTCCCAATGTACGGCTTTGGGTTCCCAATGTACGGGTTCCGGTTCCCA

ATGTACGGCTTTGGGTTCCCAATGTACGTGCTATCCACAGGAAAGAGACCTTTTCGACCTTTTTCCCCTG

CTAGGGCAATTTGCCCTAGCATCTGCTCCGTACATTAGGAACCGGCGGATGCTTCGCCCTCGATCAGGTT

GCGGTAGCGCATGACTAGGATCGGGCCAGCCTGCCCCGCCTCCTCCTTCAAATCGTACTCCGGCAGGTCA

TTTGACCCGATCAGCTTGCGCACGGTGAAACAGAACTTCTTGAACTCTCCGGCGCTGCCACTGCGTTCGT

AGATCGTCTTGAACAACCATCTGGCTTCTGCCTTGCCTGCGGCGCGGCGTGCCAGGCGGTAGAGAAAACG

GCCGATGCCGGGATCGATCAAAAAGTAATCGGGGTGAACCGTCAGCACGTCCGGGTTCTTGCCTTCTGTG

ATCTCGCGGTACATCCAATCAGCTAGCTCGATCTCGATGTACTCCGGCCGCCCGGTTTCGCTCTTTACGA

TCTTGTAGCGGCTAATCAAGGCTTCACCCTCGGATACCGTCACCAGGCGGCCGTTCTTGGCCTTCTTCGT

ACGCTGCATGGCAACGTGCGTGGTGTTTAACCGAATGCAGGTTTCTACCAGGTCGTCTTTCTGCTTTCCG

CCATCGGCTCGCCGGCAGAACTTGAGTACGTCCGCAACGTGTGGACGGAACACGCGGCCGGGCTTGTCTC

CCTTCCCTTCCCGGTATCGGTTCATGGATTCGGTTAGATGGGAAACCGCCATCAGTACCAGGTCGTAATC

CCACACACTGGCCATGCCGGCCGGCCCTGCGGAAACCTCTACGTGCCCGTCTGGAAGCTCGTAGCGGATC

ACCTCGCCAGCTCGTCGGTCACGCTTCGACAGACGGAAAACGGCCACGTCCATGATGCTGCGACTATCGC

GGGTGCCCACGTCATAGAGCATCGGAACGAAAAAATCTGGTTGCTCGTCGCCCTTGGGCGGCTTCCTAAT

CGACGGCGCACCGGCTGCCGGCGGTTGCCGGGATTCTTTGCGGATTCGATCAGCGGCCGCTTGCCACGAT

TCACCGGGGCGTGCTTCTGCCTCGATGCGTTGCCGCTGGGCGGCCTGCGCGGCCTTCAACTTCTCCACCA

GGTCATCACCCAGCGCCGCGCCGATTTGTACCGGGCCGGATGGTTTGCGACCGCTCACGCCGATTCCTCG

GGCTTGGGGGTTCCAGTGCCATTGCAGGGCCGGCAGACAACCCAGCCGCTTACGCCTGGCCAACCGCCCG

TTCCTCCACACATGGGGCATTCCACGGCGTCGGTGCCTGGTTGTTCTTGATTTTCCATGCCGCCTCCTTT

AGCCGCTAAAATTCATCTACTCATTTATTCATTTGCTCATTTACTCTGGTAGCTGCGCGATGTATTCAGA

TAGCAGCTCGGTAATGGTCTTGCCTTGGCGTACCGCGTACATCTTCAGCTTGGTGTGATCCTCCGCCGGC

AACTGAAAGTTGACCCGCTTCATGGCTGGCGTGTCTGCCAGGCTGGCCAACGTTGCAGCCTTGCTGCTGC

GTGCGCTCGGACGGCCGGCACTTAGCGTGTTTGTGCTTTTGCTCATTTTCTCTTTACCTCATTAACTCAA

ATGAGTTTTGATTTAATTTCAGCGGCCAGCGCCTGGACCTCGCGGGCAGCGTCGCCCTCGGGTTCTGATT

CAAGAACGGTTGTGCCGGCGGCGGCAGTGCCTGGGTAGCTCACGCGCTGCGTGATACGGGACTCAAGAAT

GGGCAGCTCGTACCCGGCCAGCGCCTCGGCAACCTCACCGCCGATGCGCGTGCCTTTGATCGCCCGCGAC

ACGACAAAGGCCGCTTGTAGCCTTCCATCCGTGACCTCAATGCGCTGCTTAACCAGCTCCACCAGGTCGG

CGGTGGCCCATATGTCGTAAGGGCTTGGCTGCACCGGAATCAGCACGAAGTCGGCTGCCTTGATCGCGGA

CACAGCCAAGTCCGCCGCCTGGGGCGCTCCGTCGATCACTACGAAGTCGCGCCGGCCGATGGCCTTCACG

TCGCGGTCAATCGTCGGGCGGTCGATGCCGACAACGGTTAGCGGTTGATCTTCCCGCACGGCCGCCCAAT

CGCGGGCACTGCCCTGGGGATCGGAATCGACTAACAGAACATCGGCCCCGGCGAGTTGCAGGGCGCGGGC

TAGATGGGTTGCGATGGTCGTCTTGCCTGACCCGCCTTTCTGGTTAAGTACAGCGATAACCTTCATGCGT

TCCCCTTGCGTATTTGTTTATTTACTCATCGCATCATATACGCAGCGACCGCATGACGCAAGCTGTTTTA

CTCAAATACACATCACCTTTTTAGACGGCGGCGCTCGGTTTCTTCAGCGGCCAAGCTGGCCGGCCAGGCC

GCCAGCTTGGCATCAGACAAACCGGCCAGGATTTCATGCAGCCGCACGGTTGAGACGTGCGCGGGCGGCT

CGAACACGTACCCGGCCGCGATCATCTCCGCCTCGATCTCTTCGGTAATGAAAAACGGTTCGTCCTGGCC

GTCCTGGTGCGGTTTCATGCTTGTTCCTCTTGGCGTTCATTCTCGGCGGCCGCCAGGGCGTCGGCCTCGG

TCAATGCGTCCTCACGGAAGGCACCGCGCCGCCTGGCCTCGGTGGGCGTCACTTCCTCGCTGCGCTCAAG

TGCGCGGTACAGGGTCGAGCGATGCACGCCAAGCAGTGCAGCCGCCTCTTTCACGGTGCGGCCTTCCTGG

TCGATCAGCTCGCGGGCGTGCGCGATCTGTGCCGGGGTGAGGGTAGGGCGGGGGCCAAACTTCACGCCTC

GGGCCTTGGCGGCCTCGCGCCCGCTCCGGGTGCGGTCGATGATTAGGGAACGCTCGAACTCGGCAATGCC

GGCGAACACGGTCAACACCATGCGGCCGGCCGGCGTGGTGGTGTCGGCCCACGGCTCTGCCAGGCTACGC

AGGCCCGCGCCGGCCTCCTGGATGCGCTCGGCAATGTCCAGTAGGTCGCGGGTGCTGCGGGCCAGGCGGT

CTAGCCTGGTCACTGTCACAACGTCGCCAGGGCGTAGGTGGTCAAGCATCCTGGCCAGCTCCGGGCGGTC

GCGCCTGGTGCCGGTGATCTTCTCGGAAAACAGCTTGGTGCAGCCGGCCGCGTGCAGTTCGGCCCGTTGG

TTGGTCAAGTCCTGGTCGTCGGTGCTGACGCGGGCATAGCCCAGCAGGCCAGCGGCGGCGCTCTTGTTCA

TGGCGTAATGTCTCCGGTTCTAGTCGCAAGTATTCTACTTTATGCGACTAAAACACGCGACAAGAAAACG

CCAGGAAAAGGGCAGGGCGGCAGCCTGTCGCGTAACTTAGGACTTGTGCGACATGTCGTTTTCAGAAGAC

GGCTGCACTGAACGTCAGAAGCCGACTGCACTATAGCAGCGGAGGGGTTGGATCGACCTCGAGGGGGGGC

CCGGTACCGTGAACGTCGGCTCGATTGTACCTGCGTTCAAATACTTTGCGATCGTGTTGCGCGCCTGCCC

GGTGCGTCGGCTGATCTCACGGATCGACTGCTTCTCTCGCAACGCCATCCGACGGATGATGTTTAAAAGT

CCCATGTGGATCACTCCGTTGCCCCGTCGCTCACCGTGTTGGGGGGAAGGTGCACATGGCTCAGTTCTCA

ATGGAAATTATCTGCCTAACCGGCTCAGTTCTGCGTAGAAACCAACATGCAAGCTCCACCGGGTGCAAAG

CGGCAGCGGCGGCAGGATATATTCAATTGTAAATGGCTTCATGTCCGGGAAATCTACATGGATCAGCAAT

GAGTATGATGGTCAATATGGAGAAAAAGAAAGAGTAATTACCAATTTTTTTTCAATTCAAAAATGTAGAT

GTCCGCAGCGTTATTATAAAATGAAAGTACATTTTGATAAAACGACAAATTACGATCCGTCGTATTTATA

GGCGAAAGCAATAAACAAATTATTCTAATTCGGAAATCTTTATTTCGACGTGTCTACATTCACGTCCAAA

TGGGGGCTTAGATGAGAAACTTCACGATCGATGCCTTGATTTCGCCATTCCCAGATACCCATTTCATCTT

CAGATTGGTCTGAGATTATGCGAAAATATACACTCATATACATAAATACTGACAGTTTGAGCTACCAATT

CAGTGTAGCCCATTACCTTACATAATTCACTCAAATGCTAGGCAGTCTGTCAACTCGGCGTCAATTTGTC

GGCCACTATACGATAGTTGCGCAAATTTTCAAAGTCCTGGCCTAACATCACACCTCTGTCGGCGGCGGGT

CCCATTTGTGATAAATCCACCATCACAATAGATAGTCTAATGGACGAAAAAGGCGAATATTTCGATGCTG

AGATTCGACGCAATTAATTCGAGAAAAATCCCGTGATTGATGCTGTTGAGTTACCAATAATATGGGCAGC

GAAGGCCATTTAATTATAAGATCCGATTTGGTGTATCGAGATTGGTTATGAAATTCAGATGCTAGTGTAA

TGTATTGGTAATTTGGGAAGATATAATAGGAAGCAAGGCTATTTATCCATTTCTGAAAAGGCGAAATGGC

GTCACCGCGAGCGTCACGCGCATTCCGTTCTTGCTGTAAAGCGTTGTTTGGTACACTTTTGACTAGCGAG

GCTTGGCGTGTCAGCGTATCTATTCAAAAGTCGTTAATGGCTGCGGATCAAGAAAAAGTTGGAATAGAAA

CAGAATACCCGCGAAATTCAGGCCCGGTTGCCATGTCCTACACGCCGAAATAAACGACCAAATTAGTAGA

AAAATAAAAACTGACTCGGATACTTACGTCACGTCTTGCGCACTGATTTGAAAAATCTCAATATAAACAA

AGACGGCCACAAGAAAAAACCAAAACACCGATATTCATTAATCTTATCTAGTTTCTCAAAAAAATTCATA

TCTTCCACACGTGGATCGATCCGTCGTACCAGCTTTGCAATTCATACAGAAGTGAGAAAAATGGCTTCTA

TGATATCCTCTTCAGCTGTGACTACAGTCAGCCGTGCTTCTACGGTGCAATCGGCCGCGGTGGCTCCATT

CGGCGGCCTCAAATCCATGACTGGATTCCCAGTTAAGAAGGTCAACACTGACATTACTTCCATTACAAGC

AATGGTGGAAGAGTAAAGTGCATGGTGACGGTGTTCGGCATTCTGAATCTCACCGAGGACTCCTTCTTCG

ATGAGAGCCGGCGGCTAGACCCCGCCGGCGCTGTCACCGCGGCGATCGAAATGCTGCGAGTCGGATCAGA

CGTCGTGGATGTCGGACCGGCCGCCAGCCATCCGGACGCGAGGCCTGTATCGCCGGCCGATGAGATCAGA

CGTATTGCGCCGCTCTTAGACGCCCTGTCCGATCAGATGCACCGTGTTTCAATCGACAGCTTCCAACCGG

AAACCCAGCGCTATGCGCTCAAGCGCGGCGTGGGCTACCTGAACGATATCCAAGGATTTCCTGACCCTGC

GCTCTATCCCGATATTGCTGAGGCGGACTGCAGGCTGGTGGTTATGCACTCAGCGCAGCGGGATGGCATC

GCCACCCGCACCGGTCACCTTCGACCCGAAGACGCGCTCGACGAGATTGTGCGGTTCTTCGAGGCGCGGG

TTTCCGCCTTGCGACGGAGCGGGGTCGCTGCCGACCGGCTCATCCTCGATCCGGGGATGGGATTTTTCTT

GAGCCCCGCACCGGAAACATCGCTGCACGTGCTGTCGAACCTTCAAAAGCTGAAGTCGGCGTTGGGGCTT

CCGCTATTGGTCTCGGTGTCGCGGAAATCCTTCTTGGGCGCCACCGTTGGCCTTCCTGTAAAGGATCTGG

GTCCAGCGAGCCTTGCGGCGGAACTTCACGCGATCGGCAATGGCGCTGACTACGTCCGCACCCACGCGCC

TGGAGATCTGCGAAGCGCAATCACCATCTCGGAAACCCTCGCGAAATTTCGCAGTCGCGACGCCAGAGAC

CGAGGGTTAGATCATGCCTAGCTTGGCTGCAGGTCGACCTGCAGGCATGCCCGCTGAAATCACCAGTCTC

TCTCTACAAATCTATCTCTCTCTATAATAATGTGTGAGTAGTTCCCAGATAAGGGAATTAGGGTTCTTAT

AGGGTTTCGCTCATGTGTTGAGCATATAAGAAACCCTTAGTATGTATTTGTATTTGTAAAATACTTCTAT

CAATAAAATTTCTAATTCCTAAAACCAAAATCCAGGGGTACCGAGCTC

>gi|28170640|emb|AJ537513.1|SYN537513 Synthetic construct plasmid pAC106

GAATTCGAGCTCGGTACCCGGGGATCCTCTAGAGTCGACCTGCAGGCATGCAAGCTTGGCGTAATCATGG

TCATAGCTGTTTCCTGTGTGAAATTGTTATCCGCTCACAATTCCACACAACATACGAGCCGGAAGCATAA

AGTGTAAAGCCTGGGGTGCCTAATGAGTGAGCTAACTCACATTAATTGCGTTGCGCTCACTGCCCGCTTT

CCAGTCGGGAAACCTGTCGTGCCAGCTGCATTAATGAATCGGCCAACGCGCGGGGAGAGGCGGTTTGCGT

ATTGGGCGCTCTTCCGCTTCCTCGCTCACTGACTCGCTGCGCTCGGTCGTTCGGCTGCGGCGAGCGGTAT

CAGCTCACTCAAAGGCGGTAATACGGTTATCCACAGAATCAGGGGATAACGCAGGAAAGAACATGTGAGC

AAAAGGCCAGCAAAAGGCCAGGAACCGTAAAAAGGCCGCGTTGCTGGCGTTTTTCCATAGGCTCCGCCCC

CCTGACGAGCATCACAAAAATCGACGCTCAAGTCAGAGGTGGCGAAACCCGACAGGACTATAAAGATACC

AGGCGTTTCCCCCTGGAAGCTCCCTCGTGCGCTCTCCTGTTCCGACCCTGCCGCTTACCGGATACCTGTC

CGCCTTTCTCCCTTCGGGAAGCGTGGCGCTTTCTCATAGCTCACGCTGTAGGTATCTCAGTTCGGTGTAG

GTCGTTCGCTCCAAGCTGGGCTGTGTGCACGAACCCCCCGTTCAGCCCGACCGCTGCGCCTTATCCGGTA

ACTATCGTCTTGAGTCCAACCCGGTAAGACACGACTTATCGCCACTGGCAGCAGCCACTGGTAACAGGAT

TAGCAGAGCGAGGTATGTAGGCGGTGCTACAGAGTTCTTGAAGTGGTGGCCTAACTACGGCTACACTAGA

AGAACAGTATTTGGTATCTGCGCTCTGCTGAAGCCAGTTACCTTCGGAAAAAGAGTTGGTAGCTCTTGAT

CCGGCAAACAAACCACCGCTGGTAGCGGTGGTTTTTTTGTTTGCAAGCAGCAGATTACGCGCAGAAAAAA

AGGATCTCAAGAAGATCCTTTGATCTTTTCTACGGGGTCTGACGCTCAGTGGAACGAAAACTCACGTTAA

GGGATTTTGGTCATGAGATTATCAAAAAGGATCTTCACCTAGATCCTTTTAAATTAAAAATGAAGTTTTA

AATCAATCTAAAGTATATATGAGTAAACTTGGTCTGACAGTTACCAATGCTTAATCAGTGAGGCACCTAT

CTCAGCGATCTGTCTATTTCGTTCATCCATAGTTGCCTGACTCCCCGTCGTGTAGATAACTACGATACGG

GAGGGCTTACCATCTGGCCCCAGTGCTGCAATGATACCGCGAGACCCACGCTCACCGGCTCCAGATTTAT

CAGCAATAAACCAGCCAGCCGGAAGGGCCGAGCGCAGAAGTGGTCCTGCAACTTTATCCGCCTCCATCCA

GTCTATTAATTGTTGCCGGGAAGCTAGAGTAAGTAGTTCGCCAGTTAATAGTTTGCGCAACGTTGTTGCC

ATTGCTACAGGCATCGTGGTGTCACGCTCGTCGTTTGGTATGGCTTCATTCAGCTCCGGTTCCCAACGAT

CAAGGCGAGTTACATGATCCCCCATGTTGTGCAAAAAAGCGGTTAGCTCCTTCGGTCCTCCGATCGTTGT

CAGAAGTAAGTTGGCCGCAGTGTTATCACTCATGGTTATGGCAGCACTGCATAATTCTCTTACTGTCATG

CCATCCGTAAGATGCTTTTCTGTGACTGGTGAGTACTCAACCAAGTCATTCTGAGAATAGTGTATGCGGC

GACCGAGTTGCTCTTGCCCGGCGTCAATACGGGATAATACCGCGCCACATAGCAGAACTTTAAAAGTGCT

CATCATTGGAAAACGTTCTTCGGGGCGAAAACTCTCAAGGATCTTACCGCTGTTGAGATCCAGTTCGATG

TAACCCACTCGTGCACCCAACTGATCTTCAGCATCTTTTACTTTCACCAGCGTTTCTGGGTGAGCAAAAA

CAGGAAGGCAAAATGCCGCAAAAAAGGGAATAAGGGCGACACGGAAATGTTGAATACTCATACTCTTCCT

TTTTCAATATTATTGAAGCATTTATCAGGGTTATTGTCTCATGAGCGGATACATATTTGAATGTATTTAG

AAAAATAAACAAATAGGGGTTCCGCGCACATTTCCCCGAAAAGTGCCACCTGACGTCTAAGAAACCATTA

TTATCATGACATTAACCTATAAAAATAGGCGTATCACGAGGCCCTTTCGTCTCGCGCGTTTCGGTGATGA

CGGTGAAAACCTCTGACACATGCAGCTCCCGGAGACGGTCACAGCTTGTCTGTAAGCGGATGCCGGGAGC

AGACAAGCCCGTCAGGGCGCGTCAGCGGGTGTTGGCGGGTGTCGGGGCTGGCTTAACTATGCGGCATCAG

AGCAGATTGTACTGAGAGTGCACCATATGCGGTGTGAAATACCGCACAGATGCGTAAGGAGAAAATACCG

CATCAGGCGCCATTCGCCATTCAGGCTGCGCAACTGTTGGGAAGGGCGATCGGTGCGGGCCTCTTCGCTA

TTACGCCAGCTGGCGAAAGGGGGATGTGCTGCAAGGCGATTAAGTTGGGTAACGCCAGGGTTTTCCCAGT

CACGACGTACTCCATGGGACGGCCAGTGAATTGATCCCCAATTCCCATGGAGTCAAAGATTCAAATAGAG

GACCTAACAGAACTCGCCGTAAAGACTGGCGAACAGTTCATACAGAGTCTCTTACGACTCAATGACAAGA

AGAAAATCTTCGTCAACATGGTGGAGCACGACACGCTTGTCTACTCCAAAAATATCAAAGATACAGTCTC

AGAAGACCAAAGGGCAATTGAGACTTTTCAACAAAGGGTAATATCCGGAAACCTCCTCGGATTCCATTGC

CCAGCTATCTGTCACTTTATTGTGAAGATAGTGGAAAAGGAAGGTGGCTCCTACAAATGCCATCATTGCG

ATAAAGGAAAGGCCATCGTTGAAGATGCCTCTGCCGACAGTGGTCCCAAAGATGGACCCCCACCCACGAG

GAGCATCGTGGAAAAAGAAGACGTTCCAACCACGTCTTCAAAGCAAGTGGATTGATGTGATATCTCCACT

GACGTAAGGGATGACGCACAATCCCACTATCCTTCGCAAGACCCTTCCTCTATATAAGGAAGTTCATTTC

ATTTGGAGAGGACAGGGTACCCGGGGATCAGATTGTCGTTTCCCGCCTTCGGTTTAAACTATCAGTGTTT

GACAGGATATATTGGCGGGTAAACCTAAGAGAAAAGAGCGTTTATTAGAATAATCGGATATTTAAAAGGG

CGTGAAAAGGTTTATCCGTTCGTCCATTTGTATGTGCATGCCAACCACAGGGTTCCCCTCGGGAGTGCTT

GGCATTCCGTGCGATAATGACTTCTGTTCAACCACCCAAACGTCGGAAAGCCTGACGACGGAGCAGCATT

CCAAAAAGATCCCTTGGCTCGTCTGGGTCGGCTAGAAGGTCGAGTGGGCTGCTGTGGCTTGATCCCTCAA

CGCGGTCGCGGACGTAGCGCAGCGCCGAAAAATCCTCGATCGCAAATCCGACGCTGTCGAAAAGCGTGAT

CTGCTTGTCGCTCTTTCGGCCGACGTCCTGGCCAGTCATCACGCGCCAAAGTTCCGTCACAGGATGATCT

GGCGCGAGTTGCTGGATCTCGCCTTCAATCCGGGTCTGTGGCGGGAACTCCACGAAAATATCCGAACGCA

GCAAGATATCGCGGTGCATCTCGGTCTTGCCTGGGCAGTCGCCGCCGACGCCGTTGATGTGGACGCCGGG

CCCGATCATATTGTCGCTCAGGATCGTGGCGTTGTGCTTGTCGGCCGTTGCTGTCGTAATGATATCGGCA

CCTTCGACCGCCTGTTCCGCAGAGGTGCAGGCCTCGATCTGAAACCCGAACCGCTGGAGATTGCGGGAGC

AGCGAGCAGTAGCCTCGGGGTCGATGTCGTAAAGTCGTATCCGATCGACGCCGATCAGCGCCTTGAAGGC

CAAAGCCTGGAACTCACTTTGGGCACCGTTGCCGATCAGCGCCATCGTGCGCGAATCTTTACGGGCCAGA

TACTTTGCCGCGATCGCGGAGGTCGCGGCCGTTCGCAAGGCCGTCAGGATTGTCATTTCCGACAGCAGCA

GCGGATAGCCGCTATCGACATCGGAGAGCACGCCGAACGCGGTTACCTAGAGCGGCCGCCACCGCGGTGC

CTTGATGTGGGCGCCGGCGGTCGAGTGGCGACGGCGCGGCTTGTCCGCGCCCTGGTAGATTGCCTGGCCG

TAGGCCAGCCATTTTTGAGCGGCCAGCGGCCGCGATAGGCCGACGCGAAGCGGCGGGGCGTAGGGAGCGC

AGCGACCGAAGGGTAGGCGCTTTTTGCAGCTCTTCGGCTGTGCGCTGGCCAGACAGTTATGCACAGGCCA

GGCGGGTTTTAAGAGTTTTAATAAGTTTTAAAGAGTTTTAGGCGGAAAAATCGCCTTTTTTCTCTTTTAT

ATCAGTCACTTACATGTGTGACCGGTTCCCAATGTACGGCTTTGGGTTCCCAATGTACGGGTTCCGGTTC

CCAATGTACGGCTTTGGGTTCCCAATGTACGTGCTATCCACAGGAAAGAGACCTTTTCGACCTTTTTCCC

CTGCTAGGGCAATTTGCCCTAGCATCTGCTCCGTACATTAGGAACCGGCGGATGCTTCGCCCTCGATCAG

GTTGCGGTAGCGCATGACTAGGATCGGGCCAGCCTGCCCCGCCTCCTCCTTCAAATCGTACTCCGGCAGG

TCATTTGACCCGATCAGCTTGCGCACGGTGAAACAGAACTTCTTGAACTCTCCGGCGCTGCCACTGCGTT

CGTAGATCGTCTTGAACAACCATCTGGCTTCTGCCTTGCCTGCGGCGCGGCGTGCCAGGCGGTAGAGAAA

ACGGCCGATGCCGGGATCGATCAAAAAGTAATCGGGGTGAACCGTCAGCACGTCCGGGTTCTTGCCTTCT

GTGATCTCGCGGTACATCCAATCAGCTAGCTCGATCTCGATGTACTCCGGCCGCCCGGTTTCGCTCTTTA

CGATCTTGTAGCGGCTAATCAAGGCTTCACCCTCGGATACCGTCACCAGGCGGCCGTTCTTGGCCTTCTT

CGTACGCTGCATGGCAACGTGCGTGGTGTTTAACCGAATGCAGGTTTCTACCAGGTCGTCTTTCTGCTTT

CCGCCATCGGCTCGCCGGCAGAACTTGAGTACGTCCGCAACGTGTGGACGGAACACGCGGCCGGGCTTGT

CTCCCTTCCCTTCCCGGTATCGGTTCATGGATTCGGTTAGATGGGAAACCGCCATCAGTACCAGGTCGTA

ATCCCACACACTGGCCATGCCGGCCGGCCCTGCGGAAACCTCTACGTGCCCGTCTGGAAGCTCGTAGCGG

ATCACCTCGCCAGCTCGTCGGTCACGCTTCGACAGACGGAAAACGGCCACGTCCATGATGCTGCGACTAT

CGCGGGTGCCCACGTCATAGAGCATCGGAACGAAAAAATCTGGTTGCTCGTCGCCCTTGGGCGGCTTCCT

AATCGACGGCGCACCGGCTGCCGGCGGTTGCCGGGATTCTTTGCGGATTCGATCAGCGGCCGCTTGCCAC

GATTCACCGGGGCGTGCTTCTGCCTCGATGCGTTGCCGCTGGGCGGCCTGCGCGGCCTTCAACTTCTCCA

CCAGGTCATCACCCAGCGCCGCGCCGATTTGTACCGGGCCGGATGGTTTGCGACCGCTCACGCCGATTCC

TCGGGCTTGGGGGTTCCAGTGCCATTGCAGGGCCGGCAGACAACCCAGCCGCTTACGCCTGGCCAACCGC

CCGTTCCTCCACACATGGGGCATTCCACGGCGTCGGTGCCTGGTTGTTCTTGATTTTCCATGCCGCCTCC

TTTAGCCGCTAAAATTCATCTACTCATTTATTCATTTGCTCATTTACTCTGGTAGCTGCGCGATGTATTC

AGATAGCAGCTCGGTAATGGTCTTGCCTTGGCGTACCGCGTACATCTTCAGCTTGGTGTGATCCTCCGCC

GGCAACTGAAAGTTGACCCGCTTCATGGCTGGCGTGTCTGCCAGGCTGGCCAACGTTGCAGCCTTGCTGC

TGCGTGCGCTCGGACGGCCGGCACTTAGCGTGTTTGTGCTTTTGCTCATTTTCTCTTTACCTCATTAACT

CAAATGAGTTTTGATTTAATTTCAGCGGCCAGCGCCTGGACCTCGCGGGCAGCGTCGCCCTCGGGTTCTG

ATTCAAGAACGGTTGTGCCGGCGGCGGCAGTGCCTGGGTAGCTCACGCGCTGCGTGATACGGGACTCAAG

AATGGGCAGCTCGTACCCGGCCAGCGCCTCGGCAACCTCACCGCCGATGCGCGTGCCTTTGATCGCCCGC

GACACGACAAAGGCCGCTTGTAGCCTTCCATCCGTGACCTCAATGCGCTGCTTAACCAGCTCCACCAGGT

CGGCGGTGGCCCATATGTCGTAAGGGCTTGGCTGCACCGGAATCAGCACGAAGTCGGCTGCCTTGATCGC

GGACACAGCCAAGTCCGCCGCCTGGGGCGCTCCGTCGATCACTACGAAGTCGCGCCGGCCGATGGCCTTC

ACGTCGCGGTCAATCGTCGGGCGGTCGATGCCGACAACGGTTAGCGGTTGATCTTCCCGCACGGCCGCCC

AATCGCGGGCACTGCCCTGGGGATCGGAATCGACTAACAGAACATCGGCCCCGGCGAGTTGCAGGGCGCG

GGCTAGATGGGTTGCGATGGTCGTCTTGCCTGACCCGCCTTTCTGGTTAAGTACAGCGATAACCTTCATG

CGTTCCCCTTGCGTATTTGTTTATTTACTCATCGCATCATATACGCAGCGACCGCATGACGCAAGCTGTT

TTACTCAAATACACATCACCTTTTTAGACGGCGGCGCTCGGTTTCTTCAGCGGCCAAGCTGGCCGGCCAG

GCCGCCAGCTTGGCATCAGACAAACCGGCCAGGATTTCATGCAGCCGCACGGTTGAGACGTGCGCGGGCG

GCTCGAACACGTACCCGGCCGCGATCATCTCCGCCTCGATCTCTTCGGTAATGAAAAACGGTTCGTCCTG

GCCGTCCTGGTGCGGTTTCATGCTTGTTCCTCTTGGCGTTCATTCTCGGCGGCCGCCAGGGCGTCGGCCT

CGGTCAATGCGTCCTCACGGAAGGCACCGCGCCGCCTGGCCTCGGTGGGCGTCACTTCCTCGCTGCGCTC

AAGTGCGCGGTACAGGGTCGAGCGATGCACGCCAAGCAGTGCAGCCGCCTCTTTCACGGTGCGGCCTTCC

TGGTCGATCAGCTCGCGGGCGTGCGCGATCTGTGCCGGGGTGAGGGTAGGGCGGGGGCCAAACTTCACGC

CTCGGGCCTTGGCGGCCTCGCGCCCGCTCCGGGTGCGGTCGATGATTAGGGAACGCTCGAACTCGGCAAT

GCCGGCGAACACGGTCAACACCATGCGGCCGGCCGGCGTGGTGGTGTCGGCCCACGGCTCTGCCAGGCTA

CGCAGGCCCGCGCCGGCCTCCTGGATGCGCTCGGCAATGTCCAGTAGGTCGCGGGTGCTGCGGGCCAGGC

GGTCTAGCCTGGTCACTGTCACAACGTCGCCAGGGCGTAGGTGGTCAAGCATCCTGGCCAGCTCCGGGCG

GTCGCGCCTGGTGCCGGTGATCTTCTCGGAAAACAGCTTGGTGCAGCCGGCCGCGTGCAGTTCGGCCCGT

TGGTTGGTCAAGTCCTGGTCGTCGGTGCTGACGCGGGCATAGCCCAGCAGGCCAGCGGCGGCGCTCTTGT

TCATGGCGTAATGTCTCCGGTTCTAGTCGCAAGTATTCTACTTTATGCGACTAAAACACGCGACAAGAAA

ACGCCAGGAAAAGGGCAGGGCGGCAGCCTGTCGCGTAACTTAGGACTTGTGCGACATGTCGTTTTCAGAA

GACGGCTGCACTGAACGTCAGAAGCCGACTGCACTATAGCAGCGGAGGGGTTGGATCGACCTCGAGGGGG

GGCCCGGTACCGTGAACGTCGGCTCGATTGTACCTGCGTTCAAATACTTTGCGATCGTGTTGCGCGCCTG

CCCGGTGCGTCGGCTGATCTCACGGATCGACTGCTTCTCTCGCAACGCCATCCGACGGATGATGTTTAAA

AGTCCCATGTGGATCACTCCGTTGCCCCGTCGCTCACCGTGTTGGGGGGAAGGTGCACATGGCTCAGTTC

TCAATGGAAATTATCTGCCTAACCGGCTCAGTTCTGCGTAGAAACCAACATGCAAGCTCCACCGGGTGCA

AAGCGGCAGCGGCGGCAGGATATATTCAATTGTAAATGGCTTCATGTCCGGGAAATCTACATGGATCAGC

AATGAGTATGATGGTCAATATGGAGAAAAAGAAAGAGTAATTACCAATTTTTTTTCAATTCAAAAATGTA

GATGTCCGCAGCGTTATTATAAAATGAAAGTACATTTTGATAAAACGACAAATTACGATCCGTCGTATTT

ATAGGCGAAAGCAATAAACAAATTATTCTAATTCGGAAATCTTTATTTCGACGTGTCTACATTCACGTCC

AAATGGGGGCTTAGATGAGAAACTTCACGATCGATGCCTTGATTTCGCCATTCCCAGATACCCATTTCAT

CTTCAGATTGGTCTGAGATTATGCGAAAATATACACTCATATACATAAATACTGACAGTTTGAGCTACCA

ATTCAGTGTAGCCCATTACCTTACATAATTCACTCAAATGCTAGGCAGTCTGTCAACTCGGCGTCAATTT

GTCGGCCACTATACGATAGTTGCGCAAATTTTCAAAGTCCTGGCCTAACATCACACCTCTGTCGGCGGCG

GGTCCCATTTGTGATAAATCCACCATCACAATAGATAGTCTAATGGACGAAAAAGGCGAATATTTCGATG

CTGAGATTCGACGCAATTAATTCGAGAAAAATCCCGTGATTGATGCTGTTGAGTTACCAATAATATGGGC

AGCGAAGGCCATTTAATTATAAGATCTGATCCCCGGGTACCGAGCTCGGTACCCCTGGATTTTGGTTTTA

GGAATTAGAAATTTTATTGATAGAAGTATTTTACAAATACAAATACATACTAAGGGTTTCTTATATGCTC

AACACATGAGCGAAACCCTATAAGAACCCTAATTCCCTTATCTGGGAACTACTCACACATTATTATAGAG

AGAGATAGATTTGTAGAGAGAGACTGGTGATTTCAGCGGGCATGCCTGCAGGTCGACCTGCAGCCAAGCT

AGGCATGATCTAACCCTCGGTCTCTGGCGTCGCGACTGCGAAATTTCGCGAGGGTTTCCGAGATGGTGAT

TGCGCTTCGCAGATCTCCAGGCGCGTGGGTGCGGACGTAGTCAGCGCCATTGCCGATCGCGTGAAGTTCC

GCCGCAAGGCTCGCTGGACCCAGATCCTTTACAGGAAGGCCAACGGTGGCGCCCAAGAAGGATTTCCGCG

ACACCGAGACCAATAGCGGAAGCCCCAACGCCGACTTCAGCTTTTGAAGGTTCGACAGCACGTGCAGCGA

TGTTTCCGGTGCGGGGCTCAAGAAAAATCCCATCCCCGGATCGAGGATGAGCCGGTCGGCAGCGACCCCG

CTCCGTCGCAAGGCGGAAACCCGCGCCTCGAAGAACCGCACAATCTCGTCGAGCGCGTCTTCGGGTCGAA

GGTGACCGGTGCGGGTGGCGATGCCATCCCGCTGCGCTGAGTGCATAACCACCAGCCTGCAGTCCGCCTC

AGCAATATCGGGATAGAGCGCAGGGTCAGGAAATCCTTGGATATCGTTCAGGTAGCCCACGCCGCGCTTG

AGCGCATAGCGCTGGGTTTCCGGTTGGAAGCTGTCGATTGAAACACGGTGCATCTGATCGGACAGGGCGT

CTAAGAGCGGCGCAATACGTCTGATCTCATCGGCCGGCGATACAGGCCTCGCGTCCGGATGGCTGGCGGC

CGGTCCGACATCCACGACGTCTGATCCGACTCGCAGCATTTCGATCGCCGCGGTGACAGCGCCGGCGGGG

TCTAGCCGCCGGCTCTCATCGAAGAAGGAGTCCTCGGTGAGATTCAGAATGCCGAACACCGTCACCATGC

ACTTTACTCTTCCACCATTGCTTGTAATGGAAGTAATGTCAGTGTTGACCTTCTTAACTGGGAATCCAGT

CATGGATTTGAGGCCGCCGAATGGAGCCACCGCGGCCGATTGCACCGTAGAAGCACGGCTGACTGTAGTC

ACAGCTGAAGAGGATATCATAGAAGCCATTTTTCTCACTTCTGTATGAATTGCAAAGCTGGTACCCTGTC

CTCTCCAAATGAAATGAACTTCCTTATATAGAGGAAGGGTCTTGCGAAGGATAGTGGGATTGTGCGTCAT

CCCTTACGTCAGTGGAGATATCACATCAATCCACTTGCTTTGAAGACGTGGTTGGAACGTCTTCTTTTTC

CACGATGCTCCTCGTGGGTGGGGGTCCATCTTTGGGACCACTGTCGGCAGAGGCATCTTCAACGATGGCC

TTTCCTTTATCGCAATGATGGCATTTGTAGGAGCCACCTTCCTTTTCCACTATCTTCACAATAAAGTGAC

AGATAGCTGGGCAATGGAATCCGAGGAGGTTTCCGGATATTACCCTTTGTTGAAAAGTCTCAATTGCCCT

TTGGTCTTCTGAGACTGTATCTTTGATATTTTTGGAGTAGACAAGCGTGTCGTGCTCCACCATGTTGACG

AAGATTTTCTTCTTGTCATTGAGTCGTAAGAGACTCTGTATGAACTGTTCGCCAGTCTTTACGGCGAGTT

CTGTTAGGTCCTCTATTTGAATCTTTG

>gi|28170643|emb|AJ537514.1|SYN537514 Synthetic construct plasmid pAC161

GAATTCGTAATCATGTCATAGCTGTTTCCTGTGTGAAATTGTTATCCGCTCACAATTCCACACAACATAC

GAGCCGGAAGCATAAAGTGTAAAGCCTGGGGTGCCTAATGAGTGAGCTAACTCACATTAATTGCGTTGCG

CTCACTGCCCGCTTTCCAGTCGGGAAACCTGTCGTGCCAGCTGCATTAATGAATCGGCCAACGCGCGGGG

AGAGGCGGTTTGCGTATTGGGCGCTCTTCCGCTTCCTCGCTCACTGACTCGCTGCGCTCGGTCGTTCGGC

TGCGGCGAGCGGTATCAGCTCACTCAAAGGCGGTAATACGGTTATCCACAGAATCAGGGGATAACGCAGG

AAAGAACATGTGAGCAAAAGGCCAGCAAAAGGCCAGGAACCGTAAAAAGGCCGCGTTGCTGGCGTTTTTC

CATAGGCTCCGCCCCCCTGACGAGCATCACAAAAATCGACGCTCAAGTCAGAGGTGGCGAAACCCGACAG

GACTATAAAGATACCAGGCGTTTCCCCCTGGAAGCTCCCTCGTGCGCTCTCCTGTTCCGACCCTGCCGCT

TACCGGATACCTGTCCGCCTTTCTCCCTTCGGGAAGCGTGGCGCTTTCTCATAGCTCACGCTGTAGGTAT

CTCAGTTCGGTGTAGGTCGTTCGCTCCAAGCTGGGCTGTGTGCACGAACCCCCCGTTCAGCCCGACCGCT

GCGCCTTATCCGGTAACTATCGTCTTGAGTCCAACCCGGTAAGACACGACTTATCGCCACTGGCAGCAGC

CACTGGTAACAGGATTAGCAGAGCGAGGTATGTAGGCGGTGCTACAGAGTTCTTGAAGTGGTGGCCTAAC

TACGGCTACACTAGAAGAACAGTATTTGGTATCTGCGCTCTGCTGAAGCCAGTTACCTTCGGAAAAAGAG

TTGGTAGCTCTTGATCCGGCAAACAAACCACCGCTGGTAGCGGTGGTTTTTTTGTTTGCAAGCAGCAGAT

TACGCGCAGAAAAAAAGGATCTCAAGAAGATCCTTTGATCTTTTCTACGGGGTCTGACGCTCAGTGGAAC

GAAAACTCACGTTAAGGGATTTTGGTCATGAGATTATCAAAAAGGATCTTCACCTAGATCCTTTTAAATT

AAAAATGAAGTTTTAAATCAATCTAAAGTATATATGAGTAAACTTGGTCTGACAGTTACCAATGCTTAAT

CAGTGAGGCACCTATCTCAGCGATCTGTCTATTTCGTTCATCCATAGTTGCCTGACTCCCCGTCGTGTAG

ATAACTACGATACGGGAGGGCTTACCATCTGGCCCCAGTGCTGCAATGATACCGCGAGACCCACGCTCAC

CGGCTCCAGATTTATCAGCAATAAACCAGCCAGCCGGAAGGGCCGAGCGCAGAAGTGGTCCTGCAACTTT

ATCCGCCTCCATCCAGTCTATTAATTGTTGCCGGGAAGCTAGAGTAAGTAGTTCGCCAGTTAATAGTTTG

CGCAACGTTGTTGCCATTGCTACAGGCATCGTGGTGTCACGCTCGTCGTTTGGTATGGCTTCATTCAGCT

CCGGTTCCCAACGATCAAGGCGAGTTACATGATCCCCCATGTTGTGCAAAAAAGCGGTTAGCTCCTTCGG

TCCTCCGATCGTTGTCAGAAGTAAGTTGGCCGCAGTGTTATCACTCATGGTTATGGCAGCACTGCATAAT

TCTCTTACTGTCATGCCATCCGTAAGATGCTTTTCTGTGACTGGTGAGTACTCAACCAAGTCATTCTGAG

AATAGTGTATGCGGCGACCGAGTTGCTCTTGCCCGGCGTCAATACGGGATAATACCGCGCCACATAGCAG

AACTTTAAAAGTGCTCATCATTGGAAAACGTTCTTCGGGGCGAAAACTCTCAAGGATCTTACCGCTGTTG

AGATCCAGTTCGATGTAACCCACTCGTGCACCCAACTGATCTTCAGCATCTTTTACTTTCACCAGCGTTT

CTGGGTGAGCAAAAACAGGAAGGCAAAATGCCGCAAAAAAGGGAATAAGGGCGACACGGAAATGTTGAAT

ACTCATACTCTTCCTTTTTCAATATTATTGAAGCATTTATCAGGGTTATTGTCTCATGAGCGGATACATA

TTTGAATGTATTTAGAAAAATAAACAAATAGGGGTTCCGCGCACATTTCCCCGAAAAGTGCCACCTGACG

TCTAAGAAACCATTATTATCATGACATTAACCTATAAAAATAGGCGTATCACGAGGCCCTTTCGTCTCGC

GCGTTTCGGTGATGACGGTGAAAACCTCTGACACATGCAGCTCCCGGAGACGGTCACAGCTTGTCTGTAA

GCGGATGCCGGGAGCAGACAAGCCCGTCAGGGCGCGTCAGCGGGTGTTGGCGGGTGTCGGGGCTGGCTTA

ACTATGCGGCATCAGAGCAGATTGTACTGAGAGTGCACCATATGCGGTGTGAAATACCGCACAGATGCGT

AAGGAGAAAATACCGCATCAGGCGCCATTCGCCATTCAGGCTGCGCAACTGTTGGGAAGGGCGATCGGTG

CGGGCCTCTTCGCTATTACGCCAGCTGGCGAAAGGGGGATGTGCTGCAAGGCGATTAAGTTGGGTAACGC

CAGGGTTTTCCCAGTCACGACGTTGTAAAACGACGGCCAGTGCCAAGCTTGCATGTCGACATGCATGCAT

GTCGACATGCATGCATGTCGACTCTAGAGGATCCCCAATTCCCATGGAGTCAAAGATTCAAATAGAGGAC

CTAACAGAACTCGCCGTAAAGACTGGCGAACAGTTCATACAGAGTCTCTTACGACTCAATGACAAGAAGA

AAATCTTCGTCAACATGGTGGAGCACGACACGCTTGTCTACTCCAAAAATATCAAAGATACAGTCTCAGA

AGACCAAAGGGCAATTGAGACTTTTCAACAAAGGGTAATATCCGGAAACCTCCTCGGATTCCATTGCCCA

GCTATCTGTCACTTTATTGTGAAGATAGTGGAAAAGGAAGGTGGCTCCTACAAATGCCATCATTGCGATA

AAGGAAAGGCCATCGTTGAAGATGCCTCTGCCGACAGTGGTCCCAAAGATGGACCCCCACCCACGAGGAG

CATCGTGGAAAAAGAAGACGTTCCAACCACGTCTTCAAAGCAAGTGGATTGATGTGATATCTCCACTGAC

GTAAGGGATGACGCACAATCCCACTATCCTTCGCAAGACCCTTCCTCTATATAAGGAAGTTCATTTCATT

TGGAGAGGACAGGGTACCCGGGGATCAGATTGTCGTTTCCCGCCTTCGGTTTAAACTATCAGTGTTTGAC

AGGATATATTGGCGGGTAAACCTAAGAGAAAAGAGCGTTTATTAGAATAATCGGATATTTAAAAGGGCGT

GAAAAGGTTTATCCGTTCGTCCATTTGTATGTGCATGCCAACCACAGGGTTCCCCTCGGGAGTGCTTGGC

ATTCCGTGCGATAATGACTTCTGTTCAACCACCCAAACGTCGGAAAGCCTGACGACGGAGCAGCATTCCA

AAAAGATCCCTTGGCTCGTCTGGGTCGGCTAGAAGGTCGAGTGGGCTGCTGTGGCTTGATCCCTCAACGC

GGTCGCGGACGTAGCGCAGCGCCGAAAAATCCTCGATCGCAAATCCGACGCTGTCGAAAAGCGTGATCTG

CTTGTCGCTCTTTCGGCCGACGTCCTGGCCAGTCATCACGCGCCAAAGTTCCGTCACAGGATGATCTGGC

GCGAGTTGCTGGATCTCGCCTTCAATCCGGGTCTGTGGCGGGAACTCCACGAAAATATCCGAACGCAGCA

AGATATCGCGGTGCATCTCGGTCTTGCCTGGGCAGTCGCCGCCGACGCCGTTGATGTGGACGCCGGGCCC

GATCATATTGTCGCTCAGGATCGTGGCGTTGTGCTTGTCGGCCGTTGCTGTCGTAATGATATCGGCACCT

TCGACCGCCTGTTCCGCAGAGGTGCAGGCCTCGATCTGAAACCCGAACCGCTGGAGATTGCGGGAGCAGC

GAGCAGTAGCCTCGGGGTCGATGTCGTAAAGTCGTATCCGATCGACGCCGATCAGCGCCTTGAAGGCCAA

AGCCTGGAACTCACTTTGGGCACCGTTGCCGATCAGCGCCATCGTGCGCGAATCTTTACGGGCCAGATAC

TTTGCCGCGATCGCGGAGGTCGCGGCCGTTCGCAAGGCCGTCAGGATTGTCATTTCCGACAGCAGCAGCG

GATAGCCGCTATCGACATCGGAGAGCACGCCGAACGCGGTTACCTAGAGCGGCCGCCACCGCGGTGCCTT

GATGTGGGCGCCGGCGGTCGAGTGGCGACGGCGCGGCTTGTCCGCGCCCTGGTAGATTGCCTGGCCGTAG

GCCAGCCATTTTTGAGCGGCCAGCGGCCGCGATAGGCCGACGCGAAGCGGCGGGGCGTAGGGAGCGCAGC

GACCGAAGGGTAGGCGCTTTTTGCAGCTCTTCGGCTGTGCGCTGGCCAGACAGTTATGCACAGGCCAGGC

GGGTTTTAAGAGTTTTAATAAGTTTTAAAGAGTTTTAGGCGGAAAAATCGCCTTTTTTCTCTTTTATATC

AGTCACTTACATGTGTGACCGGTTCCCAATGTACGGCTTTGGGTTCCCAATGTACGGGTTCCGGTTCCCA

ATGTACGGCTTTGGGTTCCCAATGTACGTGCTATCCACAGGAAAGAGACCTTTTCGACCTTTTTCCCCTG

CTAGGGCAATTTGCCCTAGCATCTGCTCCGTACATTAGGAACCGGCGGATGCTTCGCCCTCGATCAGGTT

GCGGTAGCGCATGACTAGGATCGGGCCAGCCTGCCCCGCCTCCTCCTTCAAATCGTACTCCGGCAGGTCA

TTTGACCCGATCAGCTTGCGCACGGTGAAACAGAACTTCTTGAACTCTCCGGCGCTGCCACTGCGTTCGT

AGATCGTCTTGAACAACCATCTGGCTTCTGCCTTGCCTGCGGCGCGGCGTGCCAGGCGGTAGAGAAAACG

GCCGATGCCGGGATCGATCAAAAAGTAATCGGGGTGAACCGTCAGCACGTCCGGGTTCTTGCCTTCTGTG

ATCTCGCGGTACATCCAATCAGCTAGCTCGATCTCGATGTACTCCGGCCGCCCGGTTTCGCTCTTTACGA

TCTTGTAGCGGCTAATCAAGGCTTCACCCTCGGATACCGTCACCAGGCGGCCGTTCTTGGCCTTCTTCGT

ACGCTGCATGGCAACGTGCGTGGTGTTTAACCGAATGCAGGTTTCTACCAGGTCGTCTTTCTGCTTTCCG

CCATCGGCTCGCCGGCAGAACTTGAGTACGTCCGCAACGTGTGGACGGAACACGCGGCCGGGCTTGTCTC

CCTTCCCTTCCCGGTATCGGTTCATGGATTCGGTTAGATGGGAAACCGCCATCAGTACCAGGTCGTAATC

CCACACACTGGCCATGCCGGCCGGCCCTGCGGAAACCTCTACGTGCCCGTCTGGAAGCTCGTAGCGGATC

ACCTCGCCAGCTCGTCGGTCACGCTTCGACAGACGGAAAACGGCCACGTCCATGATGCTGCGACTATCGC

GGGTGCCCACGTCATAGAGCATCGGAACGAAAAAATCTGGTTGCTCGTCGCCCTTGGGCGGCTTCCTAAT

CGACGGCGCACCGGCTGCCGGCGGTTGCCGGGATTCTTTGCGGATTCGATCAGCGGCCGCTTGCCACGAT

TCACCGGGGCGTGCTTCTGCCTCGATGCGTTGCCGCTGGGCGGCCTGCGCGGCCTTCAACTTCTCCACCA

GGTCATCACCCAGCGCCGCGCCGATTTGTACCGGGCCGGATGGTTTGCGACCGCTCACGCCGATTCCTCG

GGCTTGGGGGTTCCAGTGCCATTGCAGGGCCGGCAGACAACCCAGCCGCTTACGCCTGGCCAACCGCCCG

TTCCTCCACACATGGGGCATTCCACGGCGTCGGTGCCTGGTTGTTCTTGATTTTCCATGCCGCCTCCTTT

AGCCGCTAAAATTCATCTACTCATTTATTCATTTGCTCATTTACTCTGGTAGCTGCGCGATGTATTCAGA

TAGCAGCTCGGTAATGGTCTTGCCTTGGCGTACCGCGTACATCTTCAGCTTGGTGTGATCCTCCGCCGGC

AACTGAAAGTTGACCCGCTTCATGGCTGGCGTGTCTGCCAGGCTGGCCAACGTTGCAGCCTTGCTGCTGC

GTGCGCTCGGACGGCCGGCACTTAGCGTGTTTGTGCTTTTGCTCATTTTCTCTTTACCTCATTAACTCAA

ATGAGTTTTGATTTAATTTCAGCGGCCAGCGCCTGGACCTCGCGGGCAGCGTCGCCCTCGGGTTCTGATT

CAAGAACGGTTGTGCCGGCGGCGGCAGTGCCTGGGTAGCTCACGCGCTGCGTGATACGGGACTCAAGAAT

GGGCAGCTCGTACCCGGCCAGCGCCTCGGCAACCTCACCGCCGATGCGCGTGCCTTTGATCGCCCGCGAC

ACGACAAAGGCCGCTTGTAGCCTTCCATCCGTGACCTCAATGCGCTGCTTAACCAGCTCCACCAGGTCGG

CGGTGGCCCATATGTCGTAAGGGCTTGGCTGCACCGGAATCAGCACGAAGTCGGCTGCCTTGATCGCGGA

CACAGCCAAGTCCGCCGCCTGGGGCGCTCCGTCGATCACTACGAAGTCGCGCCGGCCGATGGCCTTCACG

TCGCGGTCAATCGTCGGGCGGTCGATGCCGACAACGGTTAGCGGTTGATCTTCCCGCACGGCCGCCCAAT

CGCGGGCACTGCCCTGGGGATCGGAATCGACTAACAGAACATCGGCCCCGGCGAGTTGCAGGGCGCGGGC

TAGATGGGTTGCGATGGTCGTCTTGCCTGACCCGCCTTTCTGGTTAAGTACAGCGATAACCTTCATGCGT

TCCCCTTGCGTATTTGTTTATTTACTCATCGCATCATATACGCAGCGACCGCATGACGCAAGCTGTTTTA

CTCAAATACACATCACCTTTTTAGACGGCGGCGCTCGGTTTCTTCAGCGGCCAAGCTGGCCGGCCAGGCC

GCCAGCTTGGCATCAGACAAACCGGCCAGGATTTCATGCAGCCGCACGGTTGAGACGTGCGCGGGCGGCT

CGAACACGTACCCGGCCGCGATCATCTCCGCCTCGATCTCTTCGGTAATGAAAAACGGTTCGTCCTGGCC

GTCCTGGTGCGGTTTCATGCTTGTTCCTCTTGGCGTTCATTCTCGGCGGCCGCCAGGGCGTCGGCCTCGG

TCAATGCGTCCTCACGGAAGGCACCGCGCCGCCTGGCCTCGGTGGGCGTCACTTCCTCGCTGCGCTCAAG

TGCGCGGTACAGGGTCGAGCGATGCACGCCAAGCAGTGCAGCCGCCTCTTTCACGGTGCGGCCTTCCTGG

TCGATCAGCTCGCGGGCGTGCGCGATCTGTGCCGGGGTGAGGGTAGGGCGGGGGCCAAACTTCACGCCTC

GGGCCTTGGCGGCCTCGCGCCCGCTCCGGGTGCGGTCGATGATTAGGGAACGCTCGAACTCGGCAATGCC

GGCGAACACGGTCAACACCATGCGGCCGGCCGGCGTGGTGGTGTCGGCCCACGGCTCTGCCAGGCTACGC

AGGCCCGCGCCGGCCTCCTGGATGCGCTCGGCAATGTCCAGTAGGTCGCGGGTGCTGCGGGCCAGGCGGT

CTAGCCTGGTCACTGTCACAACGTCGCCAGGGCGTAGGTGGTCAAGCATCCTGGCCAGCTCCGGGCGGTC

GCGCCTGGTGCCGGTGATCTTCTCGGAAAACAGCTTGGTGCAGCCGGCCGCGTGCAGTTCGGCCCGTTGG

TTGGTCAAGTCCTGGTCGTCGGTGCTGACGCGGGCATAGCCCAGCAGGCCAGCGGCGGCGCTCTTGTTCA

TGGCGTAATGTCTCCGGTTCTAGTCGCAAGTATTCTACTTTATGCGACTAAAACACGCGACAAGAAAACG

CCAGGAAAAGGGCAGGGCGGCAGCCTGTCGCGTAACTTAGGACTTGTGCGACATGTCGTTTTCAGAAGAC

GGCTGCACTGAACGTCAGAAGCCGACTGCACTATAGCAGCGGAGGGGTTGGATCGACCTCGAGGGGGGGC

CCGGTACCGTGAACGTCGGCTCGATTGTACCTGCGTTCAAATACTTTGCGATCGTGTTGCGCGCCTGCCC

GGTGCGTCGGCTGATCTCACGGATCGACTGCTTCTCTCGCAACGCCATCCGACGGATGATGTTTAAAAGT

CCCATGTGGATCACTCCGTTGCCCCGTCGCTCACCGTGTTGGGGGGAAGGTGCACATGGCTCAGTTCTCA

ATGGAAATTATCTGCCTAACCGGCTCAGTTCTGCGTAGAAACCAACATGCAAGCTCCACCGGGTGCAAAG

CGGCAGCGGCGGCAGGATATATTCAATTGTAAATGGCTTCATGTCCGGGAAATCTACATGGATCAGCAAT

GAGTATGATGGTCAATATGGAGAAAAAGAAAGAGTAATTACCAATTTTTTTTCAATTCAAAAATGTAGAT

GTCCGCAGCGTTATTATAAAATGAAAGTACATTTTGATAAAACGACAAATTACGATCCGTCGTATTTATA

GGCGAAAGCAATAAACAAATTATTCTAATTCGGAAATCTTTATTTCGACGTGTCTACATTCACGTCCAAA

TGGGGGCTTAGATGAGAAACTTCACGATCGATGCCTTGATTTCGCCATTCCCAGATACCCATTTCATCTT

CAGATTGGTCTGAGATTATGCGAAAATATACACTCATATACATAAATACTGACAGTTTGAGCTACCAATT

CAGTGTAGCCCATTACCTTACATAATTCACTCAAATGCTAGGCAGTCTGTCAACTCGGCGTCAATTTGTC

GGCCACTATACGATAGTTGCGCAAATTTTCAAAGTCCTGGCCTAACATCACACCTCTGTCGGCGGCGGGT

CCCATTTGTGATAAATCCACCATCACAATAGATAGTCTAATGGACGAAAAAGGCGAATATTTCGATGCTG

AGATTCGACGCAATTAATTCGAGAAAAATCCCGTGATTGATGCTGTTGAGTTACCAATAATATGGGCAGC

GAAGGCCATTTAATTATAAGATCCGATTTGGTGTATCGAGATTGGTTATGAAATTCAGATGCTAGTGTAA

TGTATTGGTAATTTGGGAAGATATAATAGGAAGCAAGGCTATTTATCCATTTCTGAAAAGGCGAAATGGC

GTCACCGCGAGCGTCACGCGCATTCCGTTCTTGCTGTAAAGCGTTGTTTGGTACACTTTTGACTAGCGAG

GCTTGGCGTGTCAGCGTATCTATTCAAAAGTCGTTAATGGCTGCGGATCAAGAAAAAGTTGGAATAGAAA

CAGAATACCCGCGAAATTCAGGCCCGGTTGCCATGTCCTACACGCCGAAATAAACGACCAAATTAGTAGA

AAAATAAAAACTGACTCGGATACTTACGTCACGTCTTGCGCACTGATTTGAAAAATCTCAATATAAACAA

AGACGGCCACAAGAAAAAACCAAAACACCGATATTCATTAATCTTATCTAGTTTCTCAAAAAAATTCATA

TCTTCCACACGTGGATCGATCCGTCGTACCAGCTTTGCAATTCATACAGAAGTGAGAAAAATGGCTTCTA

TGATATCCTCTTCAGCTGTGACTACAGTCAGCCGTGCTTCTACGGTGCAATCGGCCGCGGTGGCTCCATT

CGGCGGCCTCAAATCCATGACTGGATTCCCAGTTAAGAAGGTCAACACTGACATTACTTCCATTACAAGC

AATGGTGGAAGAGTAAAGTGCATGGTGACGGTGTTCGGCATTCTGAATCTCACCGAGGACTCCTTCTTCG

ATGAGAGCCGGCGGCTAGACCCCGCCGGCGCTGTCACCGCGGCGATCGAAATGCTGCGAGTCGGATCAGA

CGTCGTGGATGTCGGACCGGCCGCCAGCCATCCGGACGCGAGGCCTGTATCGCCGGCCGATGAGATCAGA

CGTATTGCGCCGCTCTTAGACGCCCTGTCCGATCAGATGCACCGTGTTTCAATCGACAGCTTCCAACCGG

AAACCCAGCGCTATGCGCTCAAGCGCGGCGTGGGCTACCTGAACGATATCCAAGGATTTCCTGACCCTGC

GCTCTATCCCGATATTGCTGAGGCGGACTGCAGGCTGGTGGTTATGCACTCAGCGCAGCGGGATGGCATC

GCCACCCGCACCGGTCACCTTCGACCCGAAGACGCGCTCGACGAGATTGTGCGGTTCTTCGAGGCGCGGG

TTTCCGCCTTGCGACGGAGCGGGGTCGCTGCCGACCGGCTCATCCTCGATCCGGGGATGGGATTTTTCTT

GAGCCCCGCACCGGAAACATCGCTGCACGTGCTGTCGAACCTTCAAAAGCTGAAGTCGGCGTTGGGGCTT

CCGCTATTGGTCTCGGTGTCGCGGAAATCCTTCTTGGGCGCCACCGTTGGCCTTCCTGTAAAGGATCTGG

GTCCAGCGAGCCTTGCGGCGGAACTTCACGCGATCGGCAATGGCGCTGACTACGTCCGCACCCACGCGCC

TGGAGATCTGCGAAGCGCAATCACCATCTCGGAAACCCTCGCGAAATTTCGCAGTCGCGACGCCAGAGAC

CGAGGGTTAGATCATGCCTAGCTTGGCTGCAGGTCGACCTGCAGGCATGCCCGCTGAAATCACCAGTCTC

TCTCTACAAATCTATCTCTCTCTATAATAATGTGTGAGTAGTTCCCAGATAAGGGAATTAGGGTTCTTAT

AGGGTTTCGCTCATGTGTTGAGCATATAAGAAACCCTTAGTATGTATTTGTATTTGTAAAATACTTCTAT

CAATAAAATTTCTAATTCCTAAAACCAAAATCCAGGGGTACCGAGCTC

>gi|28864234|gb|AY189980.1| Expression vector pBS-35S-Rluc, complete sequence

CACCTAAATTGTAAGCGTTAATATTTTGTTAAAATTCGCGTTAAATTTTTGTTAAATCAGCTCATTTTTT

AACCAATAGGCCGAAATCGGCAAAATCCCTTATAAATCAAAAGAATAGACCGAGATAGGGTTGAGTGTTG

TTCCAGTTTGGAACAAGAGTCCACTATTAAAGAACGTGGACTCCAACGTCAAAGGGCGAAAAACCGTCTA

TCAGGGCGATGGCCCACTACGTGAACCATCACCCTAATCAAGTTTTTTGGGGTCGAGGTGCCGTAAAGCA

CTAAATCGGAACCCTAAAGGGAGCCCCCGATTTAGAGCTTGACGGGGAAAGCCGGCGAACGTGGCGAGAA

AGGAAGGGAAGAAAGCGAAAGGAGCGGGCGCTAGGGCGCTGGCAAGTGTAGCGGTCACGCTGCGCGTAAC

CACCACACCCGCCGCGCTTAATGCGCCGCTACAGGGCGCGTCCCATTCGCCATTCAGGCTGCGCAACTGT

TGGGAAGGGCGATCGGTGCGGGCCTCTTCGCTATTACGCCAGCTGGCGAAAGGGGGATGTGCTGCAAGGC

GATTAAGTTGGGTAACGCCAGGGTTTTCCCAGTCACGACGTTGTAAAACGACGGCCAGTGAATTGTAATA

CGACTCACTATAGGGCGAATTGGGTACCGGGCCCCCCTCGAGGTCGACGGTATCGATAAGCTTGATATCG

AATTCCTGCAGGTCAACATGGTGGAGCACGACACACTTGTCTACTCCAAAAATATCAAAGATACAGTCTC

AGAAGACCAAAGGGCAATTGAGACTTTTCAACAAAGGGTAATATCCGGAAACCTCCTCGGATTCCATTGC

CCAGCTATCTGTCACTTTATTGTGAAGATAGTGGAAAAGGAAGGTGGCTCCTACAAATGCCATCATTGCG

ATAAAGGAAAGGCCATCGTTGAAGATGCCTCTGCCGACAGTGGTCCCAAAGATGGACCCCCACCCACGAG

GAGCATCGTGGAAAAAGAAGACGTTCCAACCACGTCTTCAAAGCAAGTGGATTGATGTGATAACATGGTG

GAGCACGACACACTTGTCTACTCCAAAAATATCAAAGATACAGTCTCAGAAGACCAAAGGGCAATTGAGA

CTTTTCAACAAAGGGTAATATCCGGAAACCTCCTCGGATTCCATTGCCCAGCTATCTGTCACTTTATTGT

GAAGATAGTGGAAAAGGAAGGTGGCTCCTACAAATGCCATCATTGCGATAAAGGAAAGGCCATCGTTGAA

GATGCCTCTGCCGACAGTGGTCCCAAAGATGGACCCCCACCCACGAGGAGCATCGTGGAAAAAGAAGACG

TTCCAACCACGTCTTCAAAGCAAGTGGATTGATGTGATATCTCCACTGACGTAAGGGATGACGCACAATC

CCACTATCCTTCGCAAGACCCTTCCTCTATATAAGGAAGTTCATTTCATTTGGAGAGGACCTCGAGAATT

CTCAACACAACATATACAAAACAAACGAATCTCAAGCAATCAAGCATTCTACTTCTATTGCAGCAATTTA

AATCATTTCTTTTAAAGCAAAAGCAATTTTCTGAAAATTTTCACCATTTACGAACGATAGCCATGGCTTC

GAAAGTTTATGATCCAGAACAAAGGAAACGGATGATAACTGGTCCGCAGTGGTGGGCCAGATGTAAACAA

ATGAATGTTCTTGATTCATTTATTAATTATTATGATTCAGAAAAACATGCAGAAAATGCTGTTATTTTTT

TACATGGTAACGCGGCCTCTTCTTATTTATGGCGACATGTTGTGCCACATATTGAGCCAGTAGCGCGGTG

TATTATACCAGACCTTATTGGTATGGGCAAATCAGGCAAATCTGGTAATGGTTCTTATAGGTTACTTGAT

CATTACAAATATCTTACTGCATGGTTTGAACTTCTTAATTTACCAAAGAAGATCATTTTTGTCGGCCATG

ATTGGGGTGCTTGTTTGGCATTTCATTATAGCTATGAGCATCAAGATAAGATCAAAGCAATAGTTCACGC

TGAAAGTGTAGTAGATGTGATTGAATCATGGGATGAATGGCCTGATATTGAAGAAGATATTGCGTTGATC

AAATCTGAAGAAGGAGAAAAAATGGTTTTGGAGAATAACTTCTTCGTGGAAACCATGTTGCCATCAAAAA

TCATGAGAAAGTTAGAACCAGAAGAATTTGCAGCATATCTTGAACCATTCAAAGAGAAAGGTGAAGTTCG

TCGTCCAACATTATCATGGCCTCGTGAAATCCCGTTAGTAAAAGGTGGTAAACCTGACGTTGTACAAATT

GTTAGGAATTATAATGCTTATCTACGTGCAAGTGATGATTTACCAAAAATGTTTATTGAATCGGACCCAG

GATTCTTTTCCAATGCTATTGTTGAAGGTGCCAAGAAGTTTCCTAATACTGAATTTGTCAAAGTAAAAGG

TCTTCATTTTTCGCAAGAAGATGCACCTGATGAAATGGGAAAATATATCAAATCGTTCGTTGAGCGAGTT

CTCAAAAATGAACAAAGATCTATCTAGAGTCCGCAAAAATCACCAGTCTCTCTCTACAAATCTATCTCTC

TCTATTTTTCTCCAGAATAATGTGTGAGTAGTTCCCAGATAAGGGAATTAGGGTTCTTATAGGGTTTCGC

TCATGTGTTGAGCATATAAGAAACCCTTAGTATGTATTTGTATTTGTAAAATACTTCTATCAATAAAATT

TCTAATTCCTAAAACCAAAATCCAGTGACCTGCAGCCCGGGGGATCCACTAGAGCGGCCGCCACCGCGGT

GGAGCTCCAGCTTTTGTTCCCTTTAGTGAGGGTTAATTTCGAGCTTGGCGTAATCATGGTCATAGCTGTT

TCCTGTGTGAAATTGTTATCCGCTCACAATTCCACACAACATACGAGCCGGAAGCATAAAGTGTAAAGCC

TGGGGTGCCTAATGAGTGAGCTAACTCACATTAATTGCGTTGCGCTCACTGCCCGCTTTCCAGTCGGGAA

ACCTGTCGTGCCAGCTGCATTAATGAATCGGCCAACGCGCGGGGAGAGGCGGTTTGCGTATTGGGCGCTC

TTCCGCTTCCTCGCTCACTGACTCGCTGCGCTCGGTCGTTCGGCTGCGGCGAGCGGTATCAGCTCACTCA

AAGGCGGTAATACGGTTATCCACAGAATCAGGGGATAACGCAGGAAAGAACATGTGAGCAAAAGGCCAGC

AAAAGGCCAGGAACCGTAAAAAGGCCGCGTTGCTGGCGTTTTTCCATAGGCTCCGCCCCCCTGACGAGCA

TCACAAAAATCGACGCTCAAGTCAGAGGTGGCGAAACCCGACAGGACTATAAAGATACCAGGCGTTTCCC

CCTGGAAGCTCCCTCGTGCGCTCTCCTGTTCCGACCCTGCCGCTTACCGGATACCTGTCCGCCTTTCTCC

CTTCGGGAAGCGTGGCGCTTTCTCATAGCTCACGCTGTAGGTATCTCAGTTCGGTGTAGGTCGTTCGCTC

CAAGCTGGGCTGTGTGCACGAACCCCCCGTTCAGCCCGACCGCTGCGCCTTATCCGGTAACTATCGTCTT

GAGTCCAACCCGGTAAGACACGACTTATCGCCACTGGCAGCAGCCACTGGTAACAGGATTAGCAGAGCGA

GGTATGTAGGCGGTGCTACAGAGTTCTTGAAGTGGTGGCCTAACTACGGCTACACTAGAAGGACAGTATT

TGGTATCTGCGCTCTGCTGAAGCCAGTTACCTTCGGAAAAAGAGTTGGTAGCTCTTGATCCGGCAAACAA

ACCACCGCTGGTAGCGGTGGTTTTTTTGTTTGCAAGCAGCAGATTACGCGCAGAAAAAAAGGATCTCAAG

AAGATCCTTTGATCTTTTCTACGGGGTCTGACGCTCAGTGGAACGAAAACTCACGTTAAGGGATTTTGGT

CATGAGATTATCAAAAAGGATCTTCACCTAGATCCTTTTAAATTAAAAATGAAGTTTTAAATCAATCTAA

AGTATATATGAGTAAACTTGGTCTGACAGTTACCAATGCTTAATCAGTGAGGCACCTATCTCAGCGATCT

GTCTATTTCGTTCATCCATAGTTGCCTGACTCCCCGTCGTGTAGATAACTACGATACGGGAGGGCTTACC

ATCTGGCCCCAGTGCTGCAATGATACCGCGAGACCCACGCTCACCGGCTCCAGATTTATCAGCAATAAAC

CAGCCAGCCGGAAGGGCCGAGCGCAGAAGTGGTCCTGCAACTTTATCCGCCTCCATCCAGTCTATTAATT

GTTGCCGGGAAGCTAGAGTAAGTAGTTCGCCAGTTAATAGTTTGCGCAACGTTGTTGCCATTGCTACAGG

CATCGTGGTGTCACGCTCGTCGTTTGGTATGGCTTCATTCAGCTCCGGTTCCCAACGATCAAGGCGAGTT

ACATGATCCCCCATGTTGTGCAAAAAAGCGGTTAGCTCCTTCGGTCCTCCGATCGTTGTCAGAAGTAAGT

TGGCCGCAGTGTTATCACTCATGGTTATGGCAGCACTGCATAATTCTCTTACTGTCATGCCATCCGTAAG

ATGCTTTTCTGTGACTGGTGAGTACTCAACCAAGTCATTCTGAGAATAGTGTATGCGGCGACCGAGTTGC

TCTTGCCCGGCGTCAATACGGGATAATACCGCGCCACATAGCAGAACTTTAAAAGTGCTCATCATTGGAA

AACGTTCTTCGGGGCGAAAACTCTCAAGGATCTTACCGCTGTTGAGATCCAGTTCGATGTAACCCACTCG

TGCACCCAACTGATCTTCAGCATCTTTTACTTTCACCAGCGTTTCTGGGTGAGCAAAAACAGGAAGGCAA

AATGCCGCAAAAAAGGGAATAAGGGCGACACGGAAATGTTGAATACTCATACTCTTCCTTTTTCAATATT

ATTGAAGCATTTATCAGGGTTATTGTCTCATGAGCGGATACATATTTGAATGTATTTAGAAAAATAAACA

AATAGGGGTTCCGCGCACATTTCCCCGAAAAGTGC

>gi|28864237|gb|AY189981.1| Expression vector pBS-35S-YFP, complete sequence

CACCTAAATTGTAAGCGTTAATATTTTGTTAAAATTCGCGTTAAATTTTTGTTAAATCAGCTCATTTTTT

AACCAATAGGCCGAAATCGGCAAAATCCCTTATAAATCAAAAGAATAGACCGAGATAGGGTTGAGTGTTG

TTCCAGTTTGGAACAAGAGTCCACTATTAAAGAACGTGGACTCCAACGTCAAAGGGCGAAAAACCGTCTA

TCAGGGCGATGGCCCACTACGTGAACCATCACCCTAATCAAGTTTTTTGGGGTCGAGGTGCCGTAAAGCA

CTAAATCGGAACCCTAAAGGGAGCCCCCGATTTAGAGCTTGACGGGGAAAGCCGGCGAACGTGGCGAGAA

AGGAAGGGAAGAAAGCGAAAGGAGCGGGCGCTAGGGCGCTGGCAAGTGTAGCGGTCACGCTGCGCGTAAC

CACCACACCCGCCGCGCTTAATGCGCCGCTACAGGGCGCGTCCCATTCGCCATTCAGGCTGCGCAACTGT

TGGGAAGGGCGATCGGTGCGGGCCTCTTCGCTATTACGCCAGCTGGCGAAAGGGGGATGTGCTGCAAGGC

GATTAAGTTGGGTAACGCCAGGGTTTTCCCAGTCACGACGTTGTAAAACGACGGCCAGTGAATTGTAATA

CGACTCACTATAGGGCGAATTGGGTACCGGGCCCCCCTCGAGGTCGACGGTATCGATAAGCTTGATATCG

AATTCCTGCAGGTCAACATGGTGGAGCACGACACACTTGTCTACTCCAAAAATATCAAAGATACAGTCTC

AGAAGACCAAAGGGCAATTGAGACTTTTCAACAAAGGGTAATATCCGGAAACCTCCTCGGATTCCATTGC

CCAGCTATCTGTCACTTTATTGTGAAGATAGTGGAAAAGGAAGGTGGCTCCTACAAATGCCATCATTGCG

ATAAAGGAAAGGCCATCGTTGAAGATGCCTCTGCCGACAGTGGTCCCAAAGATGGACCCCCACCCACGAG

GAGCATCGTGGAAAAAGAAGACGTTCCAACCACGTCTTCAAAGCAAGTGGATTGATGTGATAACATGGTG

GAGCACGACACACTTGTCTACTCCAAAAATATCAAAGATACAGTCTCAGAAGACCAAAGGGCAATTGAGA

CTTTTCAACAAAGGGTAATATCCGGAAACCTCCTCGGATTCCATTGCCCAGCTATCTGTCACTTTATTGT

GAAGATAGTGGAAAAGGAAGGTGGCTCCTACAAATGCCATCATTGCGATAAAGGAAAGGCCATCGTTGAA

GATGCCTCTGCCGACAGTGGTCCCAAAGATGGACCCCCACCCACGAGGAGCATCGTGGAAAAAGAAGACG

TTCCAACCACGTCTTCAAAGCAAGTGGATTGATGTGATATCTCCACTGACGTAAGGGATGACGCACAATC

CCACTATCCTTCGCAAGACCCTTCCTCTATATAAGGAAGTTCATTTCATTTGGAGAGGACCTCGAGAATT

CTCAACACAACATATACAAAACAAACGAATCTCAAGCAATCAAGCATTCTACTTCTATTGCAGCAATTTA

AATCATTTCTTTTAAAGCAAAAGCAATTTTCTGAAAATTTTCACCATTTACGAACGATAGCCATGGTGAG

CAAGGGCGAGGAGCTGTTCACCGGGGTGGTGCCCATCCTGGTCGAGCTGGACGGCGACGTAAACGGCCAC

AAGTTCAGCGTGTCCGGCGAGGGCGAGGGCGATGCCACCTACGGCAAGCTGACCCTGAAGTTCATCTGCA

CCACCGGCAAGCTGCCCGTGCCCTGGCCCACCCTCGTGACCACCTTCGGCTACGGCCTGCAGTGCTTCGC

CCGCTACCCCGACCACATGAAGCAGCACGACTTCTTCAAGTCCGCCATGCCCGAAGGCTACGTCCAGGAG

CGCACCATCTTCTTCAAGGACGACGGCAACTACAAGACCCGCGCCGAGGTGAAGTTCGAGGGCGACACCC

TGGTGAACCGCATCGAGCTGAAGGGCATCGACTTCAAGGAGGACGGCAACATCCTGGGGCACAAGCTGGA

GTACAACTACAACAGCCACAACGTCTATATCATGGCCGACAAGCAGAAGAACGGCATCAAGGTGAACTTC

AAGATCCGCCACAACATCGAGGACGGCAGCGTGCAGCTCGCCGACCACTACCAGCAGAACACCCCCATCG

GCGACGGCCCCGTGCTGCTGCCCGACAACCACTACCTGAGCTACCAGTCCGCCCTGAGCAAAGACCCCAA

CGAGAAGCGCGATCACATGGTCCTGCTGGAGTTCGTGACCGCCGCCGGGATCACTCTCGGCATGGACGAG

CTGTACAAGAGATCTATCTAGAGTCCGCAAAAATCACCAGTCTCTCTCTACAAATCTATCTCTCTCTATT

TTTCTCCAGAATAATGTGTGAGTAGTTCCCAGATAAGGGAATTAGGGTTCTTATAGGGTTTCGCTCATGT

GTTGAGCATATAAGAAACCCTTAGTATGTATTTGTATTTGTAAAATACTTCTATCAATAAAATTTCTAAT

TCCTAAAACCAAAATCCAGTGACCTGCAGCCCGGGGGATCCACTAGAGCGGCCGCCACCGCGGTGGAGCT

CCAGCTTTTGTTCCCTTTAGTGAGGGTTAATTTCGAGCTTGGCGTAATCATGGTCATAGCTGTTTCCTGT

GTGAAATTGTTATCCGCTCACAATTCCACACAACATACGAGCCGGAAGCATAAAGTGTAAAGCCTGGGGT

GCCTAATGAGTGAGCTAACTCACATTAATTGCGTTGCGCTCACTGCCCGCTTTCCAGTCGGGAAACCTGT

CGTGCCAGCTGCATTAATGAATCGGCCAACGCGCGGGGAGAGGCGGTTTGCGTATTGGGCGCTCTTCCGC

TTCCTCGCTCACTGACTCGCTGCGCTCGGTCGTTCGGCTGCGGCGAGCGGTATCAGCTCACTCAAAGGCG

GTAATACGGTTATCCACAGAATCAGGGGATAACGCAGGAAAGAACATGTGAGCAAAAGGCCAGCAAAAGG

CCAGGAACCGTAAAAAGGCCGCGTTGCTGGCGTTTTTCCATAGGCTCCGCCCCCCTGACGAGCATCACAA

AAATCGACGCTCAAGTCAGAGGTGGCGAAACCCGACAGGACTATAAAGATACCAGGCGTTTCCCCCTGGA

AGCTCCCTCGTGCGCTCTCCTGTTCCGACCCTGCCGCTTACCGGATACCTGTCCGCCTTTCTCCCTTCGG

GAAGCGTGGCGCTTTCTCATAGCTCACGCTGTAGGTATCTCAGTTCGGTGTAGGTCGTTCGCTCCAAGCT

GGGCTGTGTGCACGAACCCCCCGTTCAGCCCGACCGCTGCGCCTTATCCGGTAACTATCGTCTTGAGTCC

AACCCGGTAAGACACGACTTATCGCCACTGGCAGCAGCCACTGGTAACAGGATTAGCAGAGCGAGGTATG

TAGGCGGTGCTACAGAGTTCTTGAAGTGGTGGCCTAACTACGGCTACACTAGAAGGACAGTATTTGGTAT

CTGCGCTCTGCTGAAGCCAGTTACCTTCGGAAAAAGAGTTGGTAGCTCTTGATCCGGCAAACAAACCACC

GCTGGTAGCGGTGGTTTTTTTGTTTGCAAGCAGCAGATTACGCGCAGAAAAAAAGGATCTCAAGAAGATC

CTTTGATCTTTTCTACGGGGTCTGACGCTCAGTGGAACGAAAACTCACGTTAAGGGATTTTGGTCATGAG

ATTATCAAAAAGGATCTTCACCTAGATCCTTTTAAATTAAAAATGAAGTTTTAAATCAATCTAAAGTATA

TATGAGTAAACTTGGTCTGACAGTTACCAATGCTTAATCAGTGAGGCACCTATCTCAGCGATCTGTCTAT

TTCGTTCATCCATAGTTGCCTGACTCCCCGTCGTGTAGATAACTACGATACGGGAGGGCTTACCATCTGG

CCCCAGTGCTGCAATGATACCGCGAGACCCACGCTCACCGGCTCCAGATTTATCAGCAATAAACCAGCCA

GCCGGAAGGGCCGAGCGCAGAAGTGGTCCTGCAACTTTATCCGCCTCCATCCAGTCTATTAATTGTTGCC

GGGAAGCTAGAGTAAGTAGTTCGCCAGTTAATAGTTTGCGCAACGTTGTTGCCATTGCTACAGGCATCGT

GGTGTCACGCTCGTCGTTTGGTATGGCTTCATTCAGCTCCGGTTCCCAACGATCAAGGCGAGTTACATGA

TCCCCCATGTTGTGCAAAAAAGCGGTTAGCTCCTTCGGTCCTCCGATCGTTGTCAGAAGTAAGTTGGCCG

CAGTGTTATCACTCATGGTTATGGCAGCACTGCATAATTCTCTTACTGTCATGCCATCCGTAAGATGCTT

TTCTGTGACTGGTGAGTACTCAACCAAGTCATTCTGAGAATAGTGTATGCGGCGACCGAGTTGCTCTTGC

CCGGCGTCAATACGGGATAATACCGCGCCACATAGCAGAACTTTAAAAGTGCTCATCATTGGAAAACGTT

CTTCGGGGCGAAAACTCTCAAGGATCTTACCGCTGTTGAGATCCAGTTCGATGTAACCCACTCGTGCACC

CAACTGATCTTCAGCATCTTTTACTTTCACCAGCGTTTCTGGGTGAGCAAAAACAGGAAGGCAAAATGCC

GCAAAAAAGGGAATAAGGGCGACACGGAAATGTTGAATACTCATACTCTTCCTTTTTCAATATTATTGAA

GCATTTATCAGGGTTATTGTCTCATGAGCGGATACATATTTGAATGTATTTAGAAAAATAAACAAATAGG

GGTTCCGCGCACATTTCCCCGAAAAGTGC

>gi|28864240|gb|AY189982.1| Expression vector pBS-35S-Ala-Rluc, complete sequence

CACCTAAATTGTAAGCGTTAATATTTTGTTAAAATTCGCGTTAAATTTTTGTTAAATCAGCTCATTTTTT

AACCAATAGGCCGAAATCGGCAAAATCCCTTATAAATCAAAAGAATAGACCGAGATAGGGTTGAGTGTTG

TTCCAGTTTGGAACAAGAGTCCACTATTAAAGAACGTGGACTCCAACGTCAAAGGGCGAAAAACCGTCTA

TCAGGGCGATGGCCCACTACGTGAACCATCACCCTAATCAAGTTTTTTGGGGTCGAGGTGCCGTAAAGCA

CTAAATCGGAACCCTAAAGGGAGCCCCCGATTTAGAGCTTGACGGGGAAAGCCGGCGAACGTGGCGAGAA

AGGAAGGGAAGAAAGCGAAAGGAGCGGGCGCTAGGGCGCTGGCAAGTGTAGCGGTCACGCTGCGCGTAAC

CACCACACCCGCCGCGCTTAATGCGCCGCTACAGGGCGCGTCCCATTCGCCATTCAGGCTGCGCAACTGT

TGGGAAGGGCGATCGGTGCGGGCCTCTTCGCTATTACGCCAGCTGGCGAAAGGGGGATGTGCTGCAAGGC

GATTAAGTTGGGTAACGCCAGGGTTTTCCCAGTCACGACGTTGTAAAACGACGGCCAGTGAATTGTAATA

CGACTCACTATAGGGCGAATTGGGTACCGGGCCCCCCTCGAGGTCGACGGTATCGATAAGCTTGATATCG

AATTCCTGCAGGTCAACATGGTGGAGCACGACACACTTGTCTACTCCAAAAATATCAAAGATACAGTCTC

AGAAGACCAAAGGGCAATTGAGACTTTTCAACAAAGGGTAATATCCGGAAACCTCCTCGGATTCCATTGC

CCAGCTATCTGTCACTTTATTGTGAAGATAGTGGAAAAGGAAGGTGGCTCCTACAAATGCCATCATTGCG

ATAAAGGAAAGGCCATCGTTGAAGATGCCTCTGCCGACAGTGGTCCCAAAGATGGACCCCCACCCACGAG

GAGCATCGTGGAAAAAGAAGACGTTCCAACCACGTCTTCAAAGCAAGTGGATTGATGTGATAACATGGTG

GAGCACGACACACTTGTCTACTCCAAAAATATCAAAGATACAGTCTCAGAAGACCAAAGGGCAATTGAGA

CTTTTCAACAAAGGGTAATATCCGGAAACCTCCTCGGATTCCATTGCCCAGCTATCTGTCACTTTATTGT

GAAGATAGTGGAAAAGGAAGGTGGCTCCTACAAATGCCATCATTGCGATAAAGGAAAGGCCATCGTTGAA

GATGCCTCTGCCGACAGTGGTCCCAAAGATGGACCCCCACCCACGAGGAGCATCGTGGAAAAAGAAGACG

TTCCAACCACGTCTTCAAAGCAAGTGGATTGATGTGATATCTCCACTGACGTAAGGGATGACGCACAATC

CCACTATCCTTCGCAAGACCCTTCCTCTATATAAGGAAGTTCATTTCATTTGGAGAGGACCTCGAGAATT

CTCAACACAACATATACAAAACAAACGAATCTCAAGCAATCAAGCATTCTACTTCTATTGCAGCAATTTA

AATCATTTCTTTTAAAGCAAAAGCAATTTTCTGAAAATTTTCACCATTTACGAACGATAGCCATGGCGGC

GGCCGCACCGGTTGCTGCTGCTGCTGCTGCTCTCATGGCTTCGAAAGTTTATGATCCAGAACAAAGGAAA

CGGATGATAACTGGTCCGCAGTGGTGGGCCAGATGTAAACAAATGAATGTTCTTGATTCATTTATTAATT

ATTATGATTCAGAAAAACATGCAGAAAATGCTGTTATTTTTTTACATGGTAACGCGGCCTCTTCTTATTT

ATGGCGACATGTTGTGCCACATATTGAGCCAGTAGCGCGGTGTATTATACCAGACCTTATTGGTATGGGC

AAATCAGGCAAATCTGGTAATGGTTCTTATAGGTTACTTGATCATTACAAATATCTTACTGCATGGTTTG

AACTTCTTAATTTACCAAAGAAGATCATTTTTGTCGGCCATGATTGGGGTGCTTGTTTGGCATTTCATTA

TAGCTATGAGCATCAAGATAAGATCAAAGCAATAGTTCACGCTGAAAGTGTAGTAGATGTGATTGAATCA

TGGGATGAATGGCCTGATATTGAAGAAGATATTGCGTTGATCAAATCTGAAGAAGGAGAAAAAATGGTTT

TGGAGAATAACTTCTTCGTGGAAACCATGTTGCCATCAAAAATCATGAGAAAGTTAGAACCAGAAGAATT

TGCAGCATATCTTGAACCATTCAAAGAGAAAGGTGAAGTTCGTCGTCCAACATTATCATGGCCTCGTGAA

ATCCCGTTAGTAAAAGGTGGTAAACCTGACGTTGTACAAATTGTTAGGAATTATAATGCTTATCTACGTG

CAAGTGATGATTTACCAAAAATGTTTATTGAATCGGACCCAGGATTCTTTTCCAATGCTATTGTTGAAGG

TGCCAAGAAGTTTCCTAATACTGAATTTGTCAAAGTAAAAGGTCTTCATTTTTCGCAAGAAGATGCACCT

GATGAAATGGGAAAATATATCAAATCGTTCGTTGAGCGAGTTCTCAAAAATGAACAAAGATCTATCTAGA

GTCCGCAAAAATCACCAGTCTCTCTCTACAAATCTATCTCTCTCTATTTTTCTCCAGAATAATGTGTGAG

TAGTTCCCAGATAAGGGAATTAGGGTTCTTATAGGGTTTCGCTCATGTGTTGAGCATATAAGAAACCCTT

AGTATGTATTTGTATTTGTAAAATACTTCTATCAATAAAATTTCTAATTCCTAAAACCAAAATCCAGTGA

CCTGCAGCCCGGGGGATCCACTAGAGCGGCCGGCCGCCACCGCGGTGGAGCTCCAGCTTTTGTTCCCTTT

AGTGAGGGTTAATTTCGAGCTTGGCGTAATCATGGTCATAGCTGTTTCCTGTGTGAAATTGTTATCCGCT

CACAATTCCACACAACATACGAGCCGGAAGCATAAAGTGTAAAGCCTGGGGTGCCTAATGAGTGAGCTAA

CTCACATTAATTGCGTTGCGCTCACTGCCCGCTTTCCAGTCGGGAAACCTGTCGTGCCAGCTGCATTAAT

GAATCGGCCAACGCGCGGGGAGAGGCGGTTTGCGTATTGGGCGCTCTTCCGCTTCCTCGCTCACTGACTC

GCTGCGCTCGGTCGTTCGGCTGCGGCGAGCGGTATCAGCTCACTCAAAGGCGGTAATACGGTTATCCACA

GAATCAGGGGATAACGCAGGAAAGAACATGTGAGCAAAAGGCCAGCAAAAGGCCAGGAACCGTAAAAAGG

CCGCGTTGCTGGCGTTTTTCCATAGGCTCCGCCCCCCTGACGAGCATCACAAAAATCGACGCTCAAGTCA

GAGGTGGCGAAACCCGACAGGACTATAAAGATACCAGGCGTTTCCCCCTGGAAGCTCCCTCGTGCGCTCT

CCTGTTCCGACCCTGCCGCTTACCGGATACCTGTCCGCCTTTCTCCCTTCGGGAAGCGTGGCGCTTTCTC

ATAGCTCACGCTGTAGGTATCTCAGTTCGGTGTAGGTCGTTCGCTCCAAGCTGGGCTGTGTGCACGAACC

CCCCGTTCAGCCCGACCGCTGCGCCTTATCCGGTAACTATCGTCTTGAGTCCAACCCGGTAAGACACGAC

TTATCGCCACTGGCAGCAGCCACTGGTAACAGGATTAGCAGAGCGAGGTATGTAGGCGGTGCTACAGAGT

TCTTGAAGTGGTGGCCTAACTACGGCTACACTAGAAGGACAGTATTTGGTATCTGCGCTCTGCTGAAGCC

AGTTACCTTCGGAAAAAGAGTTGGTAGCTCTTGATCCGGCAAACAAACCACCGCTGGTAGCGGTGGTTTT

TTTGTTTGCAAGCAGCAGATTACGCGCAGAAAAAAAGGATCTCAAGAAGATCCTTTGATCTTTTCTACGG

GGTCTGACGCTCAGTGGAACGAAAACTCACGTTAAGGGATTTTGGTCATGAGATTATCAAAAAGGATCTT

CACCTAGATCCTTTTAAATTAAAAATGAAGTTTTAAATCAATCTAAAGTATATATGAGTAAACTTGGTCT

GACAGTTACCAATGCTTAATCAGTGAGGCACCTATCTCAGCGATCTGTCTATTTCGTTCATCCATAGTTG

CCTGACTCCCCGTCGTGTAGATAACTACGATACGGGAGGGCTTACCATCTGGCCCCAGTGCTGCAATGAT

ACCGCGAGACCCACGCTCACCGGCTCCAGATTTATCAGCAATAAACCAGCCAGCCGGAAGGGCCGAGCGC

AGAAGTGGTCCTGCAACTTTATCCGCCTCCATCCAGTCTATTAATTGTTGCCGGGAAGCTAGAGTAAGTA

GTTCGCCAGTTAATAGTTTGCGCAACGTTGTTGCCATTGCTACAGGCATCGTGGTGTCACGCTCGTCGTT

TGGTATGGCTTCATTCAGCTCCGGTTCCCAACGATCAAGGCGAGTTACATGATCCCCCATGTTGTGCAAA

AAAGCGGTTAGCTCCTTCGGTCCTCCGATCGTTGTCAGAAGTAAGTTGGCCGCAGTGTTATCACTCATGG

TTATGGCAGCACTGCATAATTCTCTTACTGTCATGCCATCCGTAAGATGCTTTTCTGTGACTGGTGAGTA

CTCAACCAAGTCATTCTGAGAATAGTGTATGCGGCGACCGAGTTGCTCTTGCCCGGCGTCAATACGGGAT

AATACCGCGCCACATAGCAGAACTTTAAAAGTGCTCATCATTGGAAAACGTTCTTCGGGGCGAAAACTCT

CAAGGATCTTACCGCTGTTGAGATCCAGTTCGATGTAACCCACTCGTGCACCCAACTGATCTTCAGCATC

TTTTACTTTCACCAGCGTTTCTGGGTGAGCAAAAACAGGAAGGCAAAATGCCGCAAAAAAGGGAATAAGG

GCGACACGGAAATGTTGAATACTCATACTCTTCCTTTTTCAATATTATTGAAGCATTTATCAGGGTTATT

GTCTCATGAGCGGATACATATTTGAATGTATTTAGAAAAATAAACAAATAGGGGTTCCGCGCACATTTCC

CCGAAAAGTGC

>gi|28864243|gb|AY189983.1| Expression vector pBS-35S-Rluc-Ala, complete sequence

CACCTAAATTGTAAGCGTTAATATTTTGTTAAAATTCGCGTTAAATTTTTGTTAAATCAGCTCATTTTTT

AACCAATAGGCCGAAATCGGCAAAATCCCTTATAAATCAAAAGAATAGACCGAGATAGGGTTGAGTGTTG

TTCCAGTTTGGAACAAGAGTCCACTATTAAAGAACGTGGACTCCAACGTCAAAGGGCGAAAAACCGTCTA

TCAGGGCGATGGCCCACTACGTGAACCATCACCCTAATCAAGTTTTTTGGGGTCGAGGTGCCGTAAAGCA

CTAAATCGGAACCCTAAAGGGAGCCCCCGATTTAGAGCTTGACGGGGAAAGCCGGCGAACGTGGCGAGAA

AGGAAGGGAAGAAAGCGAAAGGAGCGGGCGCTAGGGCGCTGGCAAGTGTAGCGGTCACGCTGCGCGTAAC

CACCACACCCGCCGCGCTTAATGCGCCGCTACAGGGCGCGTCCCATTCGCCATTCAGGCTGCGCAACTGT

TGGGAAGGGCGATCGGTGCGGGCCTCTTCGCTATTACGCCAGCTGGCGAAAGGGGGATGTGCTGCAAGGC

GATTAAGTTGGGTAACGCCAGGGTTTTCCCAGTCACGACGTTGTAAAACGACGGCCAGTGAATTGTAATA

CGACTCACTATAGGGCGAATTGGGTACCGGGCCCCCCTCGAGGTCGACGGTATCGATAAGCTTGATATCG

AATTCCTGCAGGTCAACATGGTGGAGCACGACACACTTGTCTACTCCAAAAATATCAAAGATACAGTCTC

AGAAGACCAAAGGGCAATTGAGACTTTTCAACAAAGGGTAATATCCGGAAACCTCCTCGGATTCCATTGC

CCAGCTATCTGTCACTTTATTGTGAAGATAGTGGAAAAGGAAGGTGGCTCCTACAAATGCCATCATTGCG

ATAAAGGAAAGGCCATCGTTGAAGATGCCTCTGCCGACAGTGGTCCCAAAGATGGACCCCCACCCACGAG

GAGCATCGTGGAAAAAGAAGACGTTCCAACCACGTCTTCAAAGCAAGTGGATTGATGTGATAACATGGTG

GAGCACGACACACTTGTCTACTCCAAAAATATCAAAGATACAGTCTCAGAAGACCAAAGGGCAATTGAGA

CTTTTCAACAAAGGGTAATATCCGGAAACCTCCTCGGATTCCATTGCCCAGCTATCTGTCACTTTATTGT

GAAGATAGTGGAAAAGGAAGGTGGCTCCTACAAATGCCATCATTGCGATAAAGGAAAGGCCATCGTTGAA

GATGCCTCTGCCGACAGTGGTCCCAAAGATGGACCCCCACCCACGAGGAGCATCGTGGAAAAAGAAGACG

TTCCAACCACGTCTTCAAAGCAAGTGGATTGATGTGATATCTCCACTGACGTAAGGGATGACGCACAATC

CCACTATCCTTCGCAAGACCCTTCCTCTATATAAGGAAGTTCATTTCATTTGGAGAGGACCTCGAGAATT

CTCAACACAACATATACAAAACAAACGAATCTCAAGCAATCAAGCATTCTACTTCTATTGCAGCAATTTA

AATCATTTCTTTTAAAGCAAAAGCAATTTTCTGAAAATTTTCACCATTTACGAACGATAGCCATGGCTTC

GAAAGTTTATGATCCAGAACAAAGGAAACGGATGATAACTGGTCCGCAGTGGTGGGCCAGATGTAAACAA

ATGAATGTTCTTGATTCATTTATTAATTATTATGATTCAGAAAAACATGCAGAAAATGCTGTTATTTTTT

TACATGGTAACGCGGCCTCTTCTTATTTATGGCGACATGTTGTGCCACATATTGAGCCAGTAGCGCGGTG

TATTATACCAGACCTTATTGGTATGGGCAAATCAGGCAAATCTGGTAATGGTTCTTATAGGTTACTTGAT

CATTACAAATATCTTACTGCATGGTTTGAACTTCTTAATTTACCAAAGAAGATCATTTTTGTCGGCCATG

ATTGGGGTGCTTGTTTGGCATTTCATTATAGCTATGAGCATCAAGATAAGATCAAAGCAATAGTTCACGC

TGAAAGTGTAGTAGATGTGATTGAATCATGGGATGAATGGCCTGATATTGAAGAAGATATTGCGTTGATC

AAATCTGAAGAAGGAGAAAAAATGGTTTTGGAGAATAACTTCTTCGTGGAAACCATGTTGCCATCAAAAA

TCATGAGAAAGTTAGAACCAGAAGAATTTGCAGCATATCTTGAACCATTCAAAGAGAAAGGTGAAGTTCG

TCGTCCAACATTATCATGGCCTCGTGAAATCCCGTTAGTAAAAGGTGGTAAACCTGACGTTGTACAAATT

GTTAGGAATTATAATGCTTATCTACGTGCAAGTGATGATTTACCAAAAATGTTTATTGAATCGGACCCAG

GATTCTTTTCCAATGCTATTGTTGAAGGTGCCAAGAAGTTTCCTAATACTGAATTTGTCAAAGTAAAAGG

TCTTCATTTTTCGCAAGAAGATGCACCTGATGAAATGGGAAAATATATCAAATCGTTCGTTGAGCGAGTT

CTCAAAAATGAACAAAGATCAGCTGCTGCTGCTAGATCTGCGGCCGCACCGGTCTAGAGTCCGCAAAAAT

CACCAGTCTCTCTCTACAAATCTATCTCTCTCTATTTTTCTCCAGAATAATGTGTGAGTAGTTCCCAGAT

AAGGGAATTAGGGTTCTTATAGGGTTTCGCTCATGTGTTGAGCATATAAGAAACCCTTAGTATGTATTTG

TATTTGTAAAATACTTCTATCAATAAAATTTCTAATTCCTAAAACCAAAATCCAGTGACCTGCAGCCCGG

GGGATCCACTAGAGCGGCCGGCCGCCACCGCGGTGGAGCTCCAGCTTTTGTTCCCTTTAGTGAGGGTTAA

TTTCGAGCTTGGCGTAATCATGGTCATAGCTGTTTCCTGTGTGAAATTGTTATCCGCTCACAATTCCACA

CAACATACGAGCCGGAAGCATAAAGTGTAAAGCCTGGGGTGCCTAATGAGTGAGCTAACTCACATTAATT

GCGTTGCGCTCACTGCCCGCTTTCCAGTCGGGAAACCTGTCGTGCCAGCTGCATTAATGAATCGGCCAAC

GCGCGGGGAGAGGCGGTTTGCGTATTGGGCGCTCTTCCGCTTCCTCGCTCACTGACTCGCTGCGCTCGGT

CGTTCGGCTGCGGCGAGCGGTATCAGCTCACTCAAAGGCGGTAATACGGTTATCCACAGAATCAGGGGAT

AACGCAGGAAAGAACATGTGAGCAAAAGGCCAGCAAAAGGCCAGGAACCGTAAAAAGGCCGCGTTGCTGG

CGTTTTTCCATAGGCTCCGCCCCCCTGACGAGCATCACAAAAATCGACGCTCAAGTCAGAGGTGGCGAAA

CCCGACAGGACTATAAAGATACCAGGCGTTTCCCCCTGGAAGCTCCCTCGTGCGCTCTCCTGTTCCGACC

CTGCCGCTTACCGGATACCTGTCCGCCTTTCTCCCTTCGGGAAGCGTGGCGCTTTCTCATAGCTCACGCT

GTAGGTATCTCAGTTCGGTGTAGGTCGTTCGCTCCAAGCTGGGCTGTGTGCACGAACCCCCCGTTCAGCC

CGACCGCTGCGCCTTATCCGGTAACTATCGTCTTGAGTCCAACCCGGTAAGACACGACTTATCGCCACTG

GCAGCAGCCACTGGTAACAGGATTAGCAGAGCGAGGTATGTAGGCGGTGCTACAGAGTTCTTGAAGTGGT

GGCCTAACTACGGCTACACTAGAAGGACAGTATTTGGTATCTGCGCTCTGCTGAAGCCAGTTACCTTCGG

AAAAAGAGTTGGTAGCTCTTGATCCGGCAAACAAACCACCGCTGGTAGCGGTGGTTTTTTTGTTTGCAAG

CAGCAGATTACGCGCAGAAAAAAAGGATCTCAAGAAGATCCTTTGATCTTTTCTACGGGGTCTGACGCTC

AGTGGAACGAAAACTCACGTTAAGGGATTTTGGTCATGAGATTATCAAAAAGGATCTTCACCTAGATCCT

TTTAAATTAAAAATGAAGTTTTAAATCAATCTAAAGTATATATGAGTAAACTTGGTCTGACAGTTACCAA

TGCTTAATCAGTGAGGCACCTATCTCAGCGATCTGTCTATTTCGTTCATCCATAGTTGCCTGACTCCCCG

TCGTGTAGATAACTACGATACGGGAGGGCTTACCATCTGGCCCCAGTGCTGCAATGATACCGCGAGACCC

ACGCTCACCGGCTCCAGATTTATCAGCAATAAACCAGCCAGCCGGAAGGGCCGAGCGCAGAAGTGGTCCT

GCAACTTTATCCGCCTCCATCCAGTCTATTAATTGTTGCCGGGAAGCTAGAGTAAGTAGTTCGCCAGTTA

ATAGTTTGCGCAACGTTGTTGCCATTGCTACAGGCATCGTGGTGTCACGCTCGTCGTTTGGTATGGCTTC

ATTCAGCTCCGGTTCCCAACGATCAAGGCGAGTTACATGATCCCCCATGTTGTGCAAAAAAGCGGTTAGC

TCCTTCGGTCCTCCGATCGTTGTCAGAAGTAAGTTGGCCGCAGTGTTATCACTCATGGTTATGGCAGCAC

TGCATAATTCTCTTACTGTCATGCCATCCGTAAGATGCTTTTCTGTGACTGGTGAGTACTCAACCAAGTC

ATTCTGAGAATAGTGTATGCGGCGACCGAGTTGCTCTTGCCCGGCGTCAATACGGGATAATACCGCGCCA

CATAGCAGAACTTTAAAAGTGCTCATCATTGGAAAACGTTCTTCGGGGCGAAAACTCTCAAGGATCTTAC

CGCTGTTGAGATCCAGTTCGATGTAACCCACTCGTGCACCCAACTGATCTTCAGCATCTTTTACTTTCAC

CAGCGTTTCTGGGTGAGCAAAAACAGGAAGGCAAAATGCCGCAAAAAAGGGAATAAGGGCGACACGGAAA

TGTTGAATACTCATACTCTTCCTTTTTCAATATTATTGAAGCATTTATCAGGGTTATTGTCTCATGAGCG

GATACATATTTGAATGTATTTAGAAAAATAAACAAATAGGGGTTCCGCGCACATTTCCCCGAAAAGTGC

>gi|28864246|gb|AY189984.1| Expression vector pBS-35S-Ala-YFP, complete sequence

CACCTAAATTGTAAGCGTTAATATTTTGTTAAAATTCGCGTTAAATTTTTGTTAAATCAGCTCATTTTTT

AACCAATAGGCCGAAATCGGCAAAATCCCTTATAAATCAAAAGAATAGACCGAGATAGGGTTGAGTGTTG

TTCCAGTTTGGAACAAGAGTCCACTATTAAAGAACGTGGACTCCAACGTCAAAGGGCGAAAAACCGTCTA

TCAGGGCGATGGCCCACTACGTGAACCATCACCCTAATCAAGTTTTTTGGGGTCGAGGTGCCGTAAAGCA

CTAAATCGGAACCCTAAAGGGAGCCCCCGATTTAGAGCTTGACGGGGAAAGCCGGCGAACGTGGCGAGAA

AGGAAGGGAAGAAAGCGAAAGGAGCGGGCGCTAGGGCGCTGGCAAGTGTAGCGGTCACGCTGCGCGTAAC

CACCACACCCGCCGCGCTTAATGCGCCGCTACAGGGCGCGTCCCATTCGCCATTCAGGCTGCGCAACTGT

TGGGAAGGGCGATCGGTGCGGGCCTCTTCGCTATTACGCCAGCTGGCGAAAGGGGGATGTGCTGCAAGGC

GATTAAGTTGGGTAACGCCAGGGTTTTCCCAGTCACGACGTTGTAAAACGACGGCCAGTGAATTGTAATA

CGACTCACTATAGGGCGAATTGGGTACCGGGCCCCCCTCGAGGTCGACGGTATCGATAAGCTTGATATCG

AATTCCTGCAGGTCAACATGGTGGAGCACGACACACTTGTCTACTCCAAAAATATCAAAGATACAGTCTC

AGAAGACCAAAGGGCAATTGAGACTTTTCAACAAAGGGTAATATCCGGAAACCTCCTCGGATTCCATTGC

CCAGCTATCTGTCACTTTATTGTGAAGATAGTGGAAAAGGAAGGTGGCTCCTACAAATGCCATCATTGCG

ATAAAGGAAAGGCCATCGTTGAAGATGCCTCTGCCGACAGTGGTCCCAAAGATGGACCCCCACCCACGAG

GAGCATCGTGGAAAAAGAAGACGTTCCAACCACGTCTTCAAAGCAAGTGGATTGATGTGATAACATGGTG

GAGCACGACACACTTGTCTACTCCAAAAATATCAAAGATACAGTCTCAGAAGACCAAAGGGCAATTGAGA

CTTTTCAACAAAGGGTAATATCCGGAAACCTCCTCGGATTCCATTGCCCAGCTATCTGTCACTTTATTGT

GAAGATAGTGGAAAAGGAAGGTGGCTCCTACAAATGCCATCATTGCGATAAAGGAAAGGCCATCGTTGAA

GATGCCTCTGCCGACAGTGGTCCCAAAGATGGACCCCCACCCACGAGGAGCATCGTGGAAAAAGAAGACG

TTCCAACCACGTCTTCAAAGCAAGTGGATTGATGTGATATCTCCACTGACGTAAGGGATGACGCACAATC

CCACTATCCTTCGCAAGACCCTTCCTCTATATAAGGAAGTTCATTTCATTTGGAGAGGACCTCGAGAATT

CTCAACACAACATATACAAAACAAACGAATCTCAAGCAATCAAGCATTCTACTTCTATTGCAGCAATTTA

AATCATTTCTTTTAAAGCAAAAGCAATTTTCTGAAAATTTTCACCATTTACGAACGATAGCCATGGCGGC

GGCCGCACCGGTTGCTGCTGCTGCTGCTGCTCTCATGGTGAGCAAGGGCGAGGAGCTGTTCACCGGGGTG

GTGCCCATCCTGGTCGAGCTGGACGGCGACGTAAACGGCCACAAGTTCAGCGTGTCCGGCGAGGGCGAGG

GCGATGCCACCTACGGCAAGCTGACCCTGAAGTTCATCTGCACCACCGGCAAGCTGCCCGTGCCCTGGCC

CACCCTCGTGACCACCTTCGGCTACGGCCTGCAGTGCTTCGCCCGCTACCCCGACCACATGAAGCAGCAC

GACTTCTTCAAGTCCGCCATGCCCGAAGGCTACGTCCAGGAGCGCACCATCTTCTTCAAGGACGACGGCA

ACTACAAGACCCGCGCCGAGGTGAAGTTCGAGGGCGACACCCTGGTGAACCGCATCGAGCTGAAGGGCAT

CGACTTCAAGGAGGACGGCAACATCCTGGGGCACAAGCTGGAGTACAACTACAACAGCCACAACGTCTAT

ATCATGGCCGACAAGCAGAAGAACGGCATCAAGGTGAACTTCAAGATCCGCCACAACATCGAGGACGGCA

GCGTGCAGCTCGCCGACCACTACCAGCAGAACACCCCCATCGGCGACGGCCCCGTGCTGCTGCCCGACAA

CCACTACCTGAGCTACCAGTCCGCCCTGAGCAAAGACCCCAACGAGAAGCGCGATCACATGGTCCTGCTG

GAGTTCGTGACCGCCGCCGGGATCACTCTCGGCATGGACGAGCTGTACAAGAGATCTATCTAGAGTCCGC

AAAAATCACCAGTCTCTCTCTACAAATCTATCTCTCTCTATTTTTCTCCAGAATAATGTGTGAGTAGTTC

CCAGATAAGGGAATTAGGGTTCTTATAGGGTTTCGCTCATGTGTTGAGCATATAAGAAACCCTTAGTATG

TATTTGTATTTGTAAAATACTTCTATCAATAAAATTTCTAATTCCTAAAACCAAAATCCAGTGACCTGCA

GCCCGGGGGATCCACTAGAGCGGCCGGCCGCCACCGCGGTGGAGCTCCAGCTTTTGTTCCCTTTAGTGAG

GGTTAATTTCGAGCTTGGCGTAATCATGGTCATAGCTGTTTCCTGTGTGAAATTGTTATCCGCTCACAAT

TCCACACAACATACGAGCCGGAAGCATAAAGTGTAAAGCCTGGGGTGCCTAATGAGTGAGCTAACTCACA

TTAATTGCGTTGCGCTCACTGCCCGCTTTCCAGTCGGGAAACCTGTCGTGCCAGCTGCATTAATGAATCG

GCCAACGCGCGGGGAGAGGCGGTTTGCGTATTGGGCGCTCTTCCGCTTCCTCGCTCACTGACTCGCTGCG

CTCGGTCGTTCGGCTGCGGCGAGCGGTATCAGCTCACTCAAAGGCGGTAATACGGTTATCCACAGAATCA

GGGGATAACGCAGGAAAGAACATGTGAGCAAAAGGCCAGCAAAAGGCCAGGAACCGTAAAAAGGCCGCGT

TGCTGGCGTTTTTCCATAGGCTCCGCCCCCCTGACGAGCATCACAAAAATCGACGCTCAAGTCAGAGGTG

GCGAAACCCGACAGGACTATAAAGATACCAGGCGTTTCCCCCTGGAAGCTCCCTCGTGCGCTCTCCTGTT

CCGACCCTGCCGCTTACCGGATACCTGTCCGCCTTTCTCCCTTCGGGAAGCGTGGCGCTTTCTCATAGCT

CACGCTGTAGGTATCTCAGTTCGGTGTAGGTCGTTCGCTCCAAGCTGGGCTGTGTGCACGAACCCCCCGT

TCAGCCCGACCGCTGCGCCTTATCCGGTAACTATCGTCTTGAGTCCAACCCGGTAAGACACGACTTATCG

CCACTGGCAGCAGCCACTGGTAACAGGATTAGCAGAGCGAGGTATGTAGGCGGTGCTACAGAGTTCTTGA

AGTGGTGGCCTAACTACGGCTACACTAGAAGGACAGTATTTGGTATCTGCGCTCTGCTGAAGCCAGTTAC

CTTCGGAAAAAGAGTTGGTAGCTCTTGATCCGGCAAACAAACCACCGCTGGTAGCGGTGGTTTTTTTGTT

TGCAAGCAGCAGATTACGCGCAGAAAAAAAGGATCTCAAGAAGATCCTTTGATCTTTTCTACGGGGTCTG

ACGCTCAGTGGAACGAAAACTCACGTTAAGGGATTTTGGTCATGAGATTATCAAAAAGGATCTTCACCTA

GATCCTTTTAAATTAAAAATGAAGTTTTAAATCAATCTAAAGTATATATGAGTAAACTTGGTCTGACAGT

TACCAATGCTTAATCAGTGAGGCACCTATCTCAGCGATCTGTCTATTTCGTTCATCCATAGTTGCCTGAC

TCCCCGTCGTGTAGATAACTACGATACGGGAGGGCTTACCATCTGGCCCCAGTGCTGCAATGATACCGCG

AGACCCACGCTCACCGGCTCCAGATTTATCAGCAATAAACCAGCCAGCCGGAAGGGCCGAGCGCAGAAGT

GGTCCTGCAACTTTATCCGCCTCCATCCAGTCTATTAATTGTTGCCGGGAAGCTAGAGTAAGTAGTTCGC

CAGTTAATAGTTTGCGCAACGTTGTTGCCATTGCTACAGGCATCGTGGTGTCACGCTCGTCGTTTGGTAT

GGCTTCATTCAGCTCCGGTTCCCAACGATCAAGGCGAGTTACATGATCCCCCATGTTGTGCAAAAAAGCG

GTTAGCTCCTTCGGTCCTCCGATCGTTGTCAGAAGTAAGTTGGCCGCAGTGTTATCACTCATGGTTATGG

CAGCACTGCATAATTCTCTTACTGTCATGCCATCCGTAAGATGCTTTTCTGTGACTGGTGAGTACTCAAC

CAAGTCATTCTGAGAATAGTGTATGCGGCGACCGAGTTGCTCTTGCCCGGCGTCAATACGGGATAATACC

GCGCCACATAGCAGAACTTTAAAAGTGCTCATCATTGGAAAACGTTCTTCGGGGCGAAAACTCTCAAGGA

TCTTACCGCTGTTGAGATCCAGTTCGATGTAACCCACTCGTGCACCCAACTGATCTTCAGCATCTTTTAC

TTTCACCAGCGTTTCTGGGTGAGCAAAAACAGGAAGGCAAAATGCCGCAAAAAAGGGAATAAGGGCGACA

CGGAAATGTTGAATACTCATACTCTTCCTTTTTCAATATTATTGAAGCATTTATCAGGGTTATTGTCTCA

TGAGCGGATACATATTTGAATGTATTTAGAAAAATAAACAAATAGGGGTTCCGCGCACATTTCCCCGAAA

AGTGC

>gi|28864249|gb|AY189985.1| Expression vector pBS-35S-YFP-Ala, complete sequence

CACCTAAATTGTAAGCGTTAATATTTTGTTAAAATTCGCGTTAAATTTTTGTTAAATCAGCTCATTTTTT

AACCAATAGGCCGAAATCGGCAAAATCCCTTATAAATCAAAAGAATAGACCGAGATAGGGTTGAGTGTTG

TTCCAGTTTGGAACAAGAGTCCACTATTAAAGAACGTGGACTCCAACGTCAAAGGGCGAAAAACCGTCTA

TCAGGGCGATGGCCCACTACGTGAACCATCACCCTAATCAAGTTTTTTGGGGTCGAGGTGCCGTAAAGCA

CTAAATCGGAACCCTAAAGGGAGCCCCCGATTTAGAGCTTGACGGGGAAAGCCGGCGAACGTGGCGAGAA

AGGAAGGGAAGAAAGCGAAAGGAGCGGGCGCTAGGGCGCTGGCAAGTGTAGCGGTCACGCTGCGCGTAAC

CACCACACCCGCCGCGCTTAATGCGCCGCTACAGGGCGCGTCCCATTCGCCATTCAGGCTGCGCAACTGT

TGGGAAGGGCGATCGGTGCGGGCCTCTTCGCTATTACGCCAGCTGGCGAAAGGGGGATGTGCTGCAAGGC

GATTAAGTTGGGTAACGCCAGGGTTTTCCCAGTCACGACGTTGTAAAACGACGGCCAGTGAATTGTAATA

CGACTCACTATAGGGCGAATTGGGTACCGGGCCCCCCTCGAGGTCGACGGTATCGATAAGCTTGATATCG

AATTCCTGCAGGTCAACATGGTGGAGCACGACACACTTGTCTACTCCAAAAATATCAAAGATACAGTCTC

AGAAGACCAAAGGGCAATTGAGACTTTTCAACAAAGGGTAATATCCGGAAACCTCCTCGGATTCCATTGC

CCAGCTATCTGTCACTTTATTGTGAAGATAGTGGAAAAGGAAGGTGGCTCCTACAAATGCCATCATTGCG

ATAAAGGAAAGGCCATCGTTGAAGATGCCTCTGCCGACAGTGGTCCCAAAGATGGACCCCCACCCACGAG

GAGCATCGTGGAAAAAGAAGACGTTCCAACCACGTCTTCAAAGCAAGTGGATTGATGTGATAACATGGTG

GAGCACGACACACTTGTCTACTCCAAAAATATCAAAGATACAGTCTCAGAAGACCAAAGGGCAATTGAGA

CTTTTCAACAAAGGGTAATATCCGGAAACCTCCTCGGATTCCATTGCCCAGCTATCTGTCACTTTATTGT

GAAGATAGTGGAAAAGGAAGGTGGCTCCTACAAATGCCATCATTGCGATAAAGGAAAGGCCATCGTTGAA

GATGCCTCTGCCGACAGTGGTCCCAAAGATGGACCCCCACCCACGAGGAGCATCGTGGAAAAAGAAGACG

TTCCAACCACGTCTTCAAAGCAAGTGGATTGATGTGATATCTCCACTGACGTAAGGGATGACGCACAATC

CCACTATCCTTCGCAAGACCCTTCCTCTATATAAGGAAGTTCATTTCATTTGGAGAGGACCTCGAGAATT

CTCAACACAACATATACAAAACAAACGAATCTCAAGCAATCAAGCATTCTACTTCTATTGCAGCAATTTA

AATCATTTCTTTTAAAGCAAAAGCAATTTTCTGAAAATTTTCACCATTTACGAACGATAGCCATGGTGAG

CAAGGGCGAGGAGCTGTTCACCGGGGTGGTGCCCATCCTGGTCGAGCTGGACGGCGACGTAAACGGCCAC

AAGTTCAGCGTGTCCGGCGAGGGCGAGGGCGATGCCACCTACGGCAAGCTGACCCTGAAGTTCATCTGCA

CCACCGGCAAGCTGCCCGTGCCCTGGCCCACCCTCGTGACCACCTTCGGCTACGGCCTGCAGTGCTTCGC

CCGCTACCCCGACCACATGAAGCAGCACGACTTCTTCAAGTCCGCCATGCCCGAAGGCTACGTCCAGGAG

CGCACCATCTTCTTCAAGGACGACGGCAACTACAAGACCCGCGCCGAGGTGAAGTTCGAGGGCGACACCC

TGGTGAACCGCATCGAGCTGAAGGGCATCGACTTCAAGGAGGACGGCAACATCCTGGGGCACAAGCTGGA

GTACAACTACAACAGCCACAACGTCTATATCATGGCCGACAAGCAGAAGAACGGCATCAAGGTGAACTTC

AAGATCCGCCACAACATCGAGGACGGCAGCGTGCAGCTCGCCGACCACTACCAGCAGAACACCCCCATCG

GCGACGGCCCCGTGCTGCTGCCCGACAACCACTACCTGAGCTACCAGTCCGCCCTGAGCAAAGACCCCAA

CGAGAAGCGCGATCACATGGTCCTGCTGGAGTTCGTGACCGCCGCCGGGATCACTCTCGGCATGGACGAG

CTGTACAAGAGATCAGCTGCTGCTGCTAGATCTGCGGCCGCACCGGTCTAGAGTCCGCAAAAATCACCAG

TCTCTCTCTACAAATCTATCTCTCTCTATTTTTCTCCAGAATAATGTGTGAGTAGTTCCCAGATAAGGGA

ATTAGGGTTCTTATAGGGTTTCGCTCATGTGTTGAGCATATAAGAAACCCTTAGTATGTATTTGTATTTG

TAAAATACTTCTATCAATAAAATTTCTAATTCCTAAAACCAAAATCCAGTGACCTGCAGCCCGGGGGATC

CACTAGAGCGGCCGGCCGCCACCGCGGTGGAGCTCCAGCTTTTGTTCCCTTTAGTGAGGGTTAATTTCGA

GCTTGGCGTAATCATGGTCATAGCTGTTTCCTGTGTGAAATTGTTATCCGCTCACAATTCCACACAACAT

ACGAGCCGGAAGCATAAAGTGTAAAGCCTGGGGTGCCTAATGAGTGAGCTAACTCACATTAATTGCGTTG

CGCTCACTGCCCGCTTTCCAGTCGGGAAACCTGTCGTGCCAGCTGCATTAATGAATCGGCCAACGCGCGG

GGAGAGGCGGTTTGCGTATTGGGCGCTCTTCCGCTTCCTCGCTCACTGACTCGCTGCGCTCGGTCGTTCG

GCTGCGGCGAGCGGTATCAGCTCACTCAAAGGCGGTAATACGGTTATCCACAGAATCAGGGGATAACGCA

GGAAAGAACATGTGAGCAAAAGGCCAGCAAAAGGCCAGGAACCGTAAAAAGGCCGCGTTGCTGGCGTTTT

TCCATAGGCTCCGCCCCCCTGACGAGCATCACAAAAATCGACGCTCAAGTCAGAGGTGGCGAAACCCGAC

AGGACTATAAAGATACCAGGCGTTTCCCCCTGGAAGCTCCCTCGTGCGCTCTCCTGTTCCGACCCTGCCG

CTTACCGGATACCTGTCCGCCTTTCTCCCTTCGGGAAGCGTGGCGCTTTCTCATAGCTCACGCTGTAGGT

ATCTCAGTTCGGTGTAGGTCGTTCGCTCCAAGCTGGGCTGTGTGCACGAACCCCCCGTTCAGCCCGACCG

CTGCGCCTTATCCGGTAACTATCGTCTTGAGTCCAACCCGGTAAGACACGACTTATCGCCACTGGCAGCA

GCCACTGGTAACAGGATTAGCAGAGCGAGGTATGTAGGCGGTGCTACAGAGTTCTTGAAGTGGTGGCCTA

ACTACGGCTACACTAGAAGGACAGTATTTGGTATCTGCGCTCTGCTGAAGCCAGTTACCTTCGGAAAAAG

AGTTGGTAGCTCTTGATCCGGCAAACAAACCACCGCTGGTAGCGGTGGTTTTTTTGTTTGCAAGCAGCAG

ATTACGCGCAGAAAAAAAGGATCTCAAGAAGATCCTTTGATCTTTTCTACGGGGTCTGACGCTCAGTGGA

ACGAAAACTCACGTTAAGGGATTTTGGTCATGAGATTATCAAAAAGGATCTTCACCTAGATCCTTTTAAA

TTAAAAATGAAGTTTTAAATCAATCTAAAGTATATATGAGTAAACTTGGTCTGACAGTTACCAATGCTTA

ATCAGTGAGGCACCTATCTCAGCGATCTGTCTATTTCGTTCATCCATAGTTGCCTGACTCCCCGTCGTGT

AGATAACTACGATACGGGAGGGCTTACCATCTGGCCCCAGTGCTGCAATGATACCGCGAGACCCACGCTC

ACCGGCTCCAGATTTATCAGCAATAAACCAGCCAGCCGGAAGGGCCGAGCGCAGAAGTGGTCCTGCAACT

TTATCCGCCTCCATCCAGTCTATTAATTGTTGCCGGGAAGCTAGAGTAAGTAGTTCGCCAGTTAATAGTT

TGCGCAACGTTGTTGCCATTGCTACAGGCATCGTGGTGTCACGCTCGTCGTTTGGTATGGCTTCATTCAG

CTCCGGTTCCCAACGATCAAGGCGAGTTACATGATCCCCCATGTTGTGCAAAAAAGCGGTTAGCTCCTTC

GGTCCTCCGATCGTTGTCAGAAGTAAGTTGGCCGCAGTGTTATCACTCATGGTTATGGCAGCACTGCATA

ATTCTCTTACTGTCATGCCATCCGTAAGATGCTTTTCTGTGACTGGTGAGTACTCAACCAAGTCATTCTG

AGAATAGTGTATGCGGCGACCGAGTTGCTCTTGCCCGGCGTCAATACGGGATAATACCGCGCCACATAGC

AGAACTTTAAAAGTGCTCATCATTGGAAAACGTTCTTCGGGGCGAAAACTCTCAAGGATCTTACCGCTGT

TGAGATCCAGTTCGATGTAACCCACTCGTGCACCCAACTGATCTTCAGCATCTTTTACTTTCACCAGCGT

TTCTGGGTGAGCAAAAACAGGAAGGCAAAATGCCGCAAAAAAGGGAATAAGGGCGACACGGAAATGTTGA

ATACTCATACTCTTCCTTTTTCAATATTATTGAAGCATTTATCAGGGTTATTGTCTCATGAGCGGATACA

TATTTGAATGTATTTAGAAAAATAAACAAATAGGGGTTCCGCGCACATTTCCCCGAAAAGTGC

>gi|29335742|emb|AJ551314.1|VFO551314 Transfection vector pBTdest

GGATCCATCGATGAATTCCTGCAGGTCAACATGGTGGAGCACGACACTCTCGTCTACTCCAAGAATATCA

AAGATACAGTCTCAGAAGACCAGAGGGCTATTGAGACTTTTCAACAAAGGGTAATATCGGGAAACCTCCT

CGGATTCCATTGCCCAGCTATCTGTCACTTCATCGAAAGGACAGTAGAAAAGGAAGATGGCTTCTACAAA

TGCCATCATTGCGATAAAGGAAAGGCTATCGTTCAAGATGCCTCTACCGACAGTGGTCCCAAAGATGGAC

CCCCACCCACGAGGAACATCGTGGAAAAAGAAGACGTTCCAACCACGTCTTCAAAGCAAGTGGATTGATG

TGATATCTCCACTGACGTAAGGGATGACGCACAATCCCACTATCCTTCGCAAGACCCTTCCTCTATATAA

GGAAGTTCATTTCATTTGGAGAGGACCTCGAATCAACAAGTTTGTACAAAAAAGCTGAACGAGAAACGTA

AAATGATATAAATATCAATATATTAAATTAGATTTTGCATAAAAAACAGACTACATAATACTGTAAAACA

CAACATATCCAGTCACTATGGCGGCCGCATTAGGCACCCCAGGCTTTACACTTTATGCTTCCGGCTCGTA

TAATGTGTGGATTTTGAGTTAGGATCCGGCGAGATTTTCAGGAGCTAAGGAAGCTAAAATGGAGAAAAAA

ATCACTGGATATACCACCGTTGATATATCCCAATGGCATCGTAAAGAACATTTTGAGGCATTTCAGTCAG

TTGCTCAATGTACCTATAACCAGACCGTTCAGCTGGATATTACGGCCTTTTTAAAGACCGTAAAGAAAAA

TAAGCACAAGTTTTATCCGGCCTTTATTCACATTCTTGCCCGCCTGATGAATGCTCATCCGGAATTCCGT

ATGGCAATGAAAGACGGTGAGCTGGTGATATGGGATAGTGTTCACCCTTGTTACACCGTTTTCCATGAGC

AAACTGAAACGTTTTCATCGCTCTGGAGTGAATACCACGACGATTTCCGGCAGTTTCTACACATATATTC

GCAAGATGTGGCGTGTTACGGTGAAAACCTGGCCTATTTCCCTAAAGGGTTTATTGAGAATATGTTTTTC

GTCTCAGCCAATCCCTGGGTGAGTTTCACCAGTTTTGATTTAAACGTGGCCAATATGGACAACTTCTTCG

CCCCCGTTTTCACCATGGGCAAATATTATACGCAAGGCGACAAGGTGCTGATGCCGCTGGCGATTCAGGT

TCATCATGCCGTCTGTGATGGCTTCCATGTCGGCAGAATGCTTAATGAATTACAACAGTACTGCGATGAG

TGGCAGGGCGGGGCGTAAAGATCTGGATCCGGCTTACTAAAAGCCAGATAACAGTATGCGTATTTGCGCG

CTGATTTTTGCGGTATAAGAATATATACTGATATGTATACCCGAAGTATGTCAAAAAGAGGTGTGCTATG

AAGCAGCGTATTACAGTGACAGTTGACAGCGACAGCTATCAGTTGCTCAAGGCATATATGATGTCAATAT

CTCCGGTCTGGTAAGCACAACCATGCAGAATGAAGCCCGTCGTCTGCGTGCCGAACGCTGGAAAGCGGAA

AATCAGGAAGGGATGGCTGAGGTCGCCCGGTTTATTGAAATGAACGGCTCTTTTGCTGACGAGAACAGGG

ACTGGTGAAATGCAGTTTAAGGTTTACACCTATAAAAGAGAGAGCCGTTATCGTCTGTTTGTGGATGTAC

AGAGTGATATTATTGACACGCCCGGGCGACGGATGGTGATCCCCCTGGCCAGTGCACGTCTGCTGTCAGA

TAAAGTCTCCCGTGAACTTTACCCGGTGGTGCATATCGGGGATGAAAGCTGGCGCATGATGACCACCGAT

ATGGCCAGTGTGCCGGTCTCCGTTATCGGGGAAGAAGTGGCTGATCTCAGCCACCGCGAAAATGACATCA

AAAACGCCATTAACCTGATGTTCTGGGGAATATAAATGTCAGGCTCCCTTATACACAGCCAGTCTGCAGG

TCGACCATAGTGACTGGATATGTTGTGTTTTACAGTATTATGTAGTCTGTTTTTTATGCAAAATCTAATT

TAATATATTGATATTTATATCATTTTACGTTTCTCGTTCAGCTTTCTTGTACAAAGTGGTTGATCCGGGT

ACCTGATCATGAGTAATTAGCTCGAATTTCCCCGATCGTTCAAACATTTGGCAATAAAGTTTCTTAAGAT

TGAATCCTGTTGCCGGTCTTGCGATGATTATCATATAATTTCTGTTGAATTACGTTAAGCATGTAATAAT

TAACATGTAATGCATGACGTTATTTATGAGATGGGTTTTTATGATTAGAGTCCCGCAATTATACATTTAA

TACGCGATAGAAAACAAAATATAGCGCGCAAACTAGGATAAATTATCGCGCGCGGTGTCATCTATGTTAC

TAGATCGGGAATTAGATCTGCTAGCAGGTGGCACTTTTCGGGGAAATGTGCGCGGAACCCCTATTTGTTT

ATTTTTCTAAATACATTCAAATATGTATCCGCTCATGAGACAATAACCCTGATAAATGCTTCAATAATAT

TGAAAAAGGAAGAGTATGAGTATTCAACATTTCCGTGTCGCCCTTATTCCCTTTTTTGCGGCATTTTGCC

TTCCTGTTTTTGCTCACCCAGAAACGCTGGTGAAAGTAAAAGATGCTGAAGATCAGTTGGGTGCACGAGT

GGGTTACATCGAACTGGATCTCAACAGCGGTAAGATCCTTGAGAGTTTTCGCCCCGAAGAACGTTTTCCA

ATGATGAGCACTTTTAAAGTTCTGCTATGTGGCGCGGTATTATCCCGTATTGACGCCGGGCAAGAGCAAC

TCGGTCGCCGCATACACTATTCTCAGAATGACTTGGTTGAGTACTCACCAGTCACAGAAAAGCATCTTAC

GGATGGCATGACAGTAAGAGAATTATGCAGTGCTGCCATAACCATGAGTGATAACACTGCGGCCAACTTA

CTTCTGACAACGATCGGAGGACCGAAGGAGCTAACCGCTTTTTTGCACAACATGGGGGATCATGTAACTC

GCCTTGATCGTTGGGAACCGGAGCTGAATGAAGCCATACCAAACGACGAGCGTGACACCACGATGCCTGT

AGCAATGGCAACAACGTTGCGCAAACTATTAACTGGCGAACTACTTACTCTAGCTTCCCGGCAACAATTA

ATAGACTGGATGGAGGCGGATAAAGTTGCAGGACCACTTCTGCGCTCGGCCCTTCCGGCTGGCTGGTTTA

TTGCTGATAAATCTGGAGCCGGTGAGCGTGGGTCTCGCGGTATCATTGCAGCACTGGGGCCAGATGGTAA

GCCCTCCCGTATCGTAGTTATCTACACGACGGGGAGTCAGGCAACTATGGATGAACGAAATAGACAGATC

GCTGAGATAGGTGCCTCACTGATTAAGCATTGGTAACTGTCAGACCAAGTTTACTCATATATACTTTAGA

TTGATTTAAAACTTCATTTTTAATTTAAAAGGATCTAGGTGAAGATCCTTTTTGATAATCTCATGACCAA

AATCCCTTAACGTGAGTTTTCGTTCCACTGAGCGTCAGACCCCGTAGAAAAGATCAAAGGATCTTCTTGA

GATCCTTTTTTTCTGCGCGTAATCTGCTGCTTGCAAACAAAAAAACCACCGCTACCAGCGGTGGTTTGTT

TGCCGGATCAAGAGCTACCAACTCTTTTTCCGAAGGTAACTGGCTTCAGCAGAGCGCAGATACCAAATAC

TGTCCTTCTAGTGTAGCCGTAGTTAGGCCACCACTTCAAGAACTCTGTAGCACCGCCTACATACCTCGCT

CTGCTAATCCTGTTACCAGTGGCTGCTGCCAGTGGCGATAAGTCGTGTCTTACCGGGTTGGACTCAAGAC

GATAGTTACCGGATAAGGCGCAGCGGTCGGGCTGAACGGGGGGTTCGTGCACACAGCCCAGCTTGGAGCG

AACGACCTACACCGAACTGAGATACCTACAGCGTGAGCATTGAGAAAGCGCCACGCTTCCCGAAGGGAGA

AAGGCGGACAGGTATCCGGTAAGCGGCAGGGTCGGAACAGGAGAGCGCACGAGGGAGCTTCCAGGGGGAA

ACGCCTGGTATCTTTATAGTCCTGTCGGGTTTCGCCACCTCTGACTTGAGCGTCGATTTTTGTGATGCTC

GTCAGGGGGGCGGAGCCTATGGAAAAACGCCAGCAACGCGGCCTTTTTACGGTTCCTGGCCTTTTGCTGG

CCTTTTGCTCACATGTTCTTTCCTGCGTTATCCCCTGATTCTGTGGATAACCGTATTACCGCCTTTGAGT

GAGCTGATACCGCTCGCCGCAGCCGAACGACCGAGCGCAGCGAGTCAGTGAGCGAGGAAGCGGAAGAGCG

CCCAATACGCAAACCGCCTCTCCCCGCGCGTTGGCCGATTCATTAATGCAGC

>gi|29568870|gb|AY178047.1| Expression vector pYPX143, complete sequence

GCATGCCAACCACAGGGTTCCCCTCGGGATCAAAGTACTTTGATCCAACCCCTCCGCTGCTATAGTGCAG

TCGGCTTCTGACGTTCAGTGCAGCCGTCTTCTGAAAACGACATGTCGCACAAGTCCTAAGTTACGCGACA

GGCTGCCGCCCTGCCCTTTTCCTGGCGTTTTCTTGTCGCGTGTTTTAGTCGCATAAAGTAGAATACTTGC

GACTAGAACCGGAGACATTACGCCATGAACAAGAGCGCCGCCGCTGGCCTGCTGGGCTATGCCCGCGTCA

GCACCGACGACCAGGACTTGACCAACCAACGGGCCGAACTGCACGCGGCCGGCTGCACCAAGCTGTTTTC

CGAGAAGATCACCGGCACCAGGCGCGACCGCCCGGAGCTGGCCAGGATGCTTGACCACCTACGCCCTGGC

GACGTTGTGACAGTGACCAGGCTAGACCGCCTGGCCCGCAGCACCCGCGACCTACTGGACATTGCCGAGC

GCATCCAGGAGGCCGGCGCGGGCCTGCGTAGCCTGGCAGAGCCGTGGGCCGACACCACCACGCCGGCCGG

CCGCATGGTGTTGACCGTGTTCGCCGGCATTGCCGAGTTCGAGCGTTCCCTAATCATCGACCGCACCCGG

AGCGGGCGCGAGGCCGCCAAGGCCCGAGGCGTGAAGTTTGGCCCCCGCCCTACCCTCACCCCGGCACAGA

TCGCGCACGCCCGCGAGCTGATCGACCAGGAAGGCCGCACCGTGAAAGAGGCGGCTGCACTGCTTGGCGT

GCATCGCTCGACCCTGTACCGCGCACTTGAGCGCAGCGAGGAAGTGACGCCCACCGAGGCCAGGCGGCGC

GGTGCCTTCCGTGAGGACGCATTGACCGAGGCCGACGCCCTGGCGGCCGCCGAGAATGAACGCCAAGAGG

AACAAGCATGAAACCGCACCAGGACGGCCAGGACGAACCGTTTTTCATTACCGAAGAGATCGAGGCGGAG

ATGATCGCGGCCGGGTACGTGTTCGAGCCGCCCGCGCACGTCTCAACCGTGCGGCTGCATGAAATCCTGG

CCGGTTTGTCTGATGCCAAGCTGGCGGCCTGGCCGGCCAGCTTGGCCGCTGAAGAAACCGAGCGCCGCCG

TCTAAAAAGGTGATGTGTATTTGAGTAAAACAGCTTGCGTCATGCGGTCGCTGCGTATATGATGCGATGA

GTAAATAAACAAATACGCAAGGGGAACGCATGAAGGTTATCGCTGTACTTAACCAGAAAGGCGGGTCAGG

CAAGACGACCATCGCAACCCATCTAGCCCGCGCCCTGCAACTCGCCGGGGCCGATGTTCTGTTAGTCGAT

TCCGATCCCCAGGGCAGTGCCCGCGATTGGGCGGCCGTGCGGGAAGATCAACCGCTAACCGTTGTCGGCA

TCGACCGCCCGACGATTGACCGCGACGTGAAGGCCATCGGCCGGCGCGACTTCGTAGTGATCGACGGAGC

GCCCCAGGCGGCGGACTTGGCTGTGTCCGCGATCAAGGCAGCCGACTTCGTGCTGATTCCGGTGCAGCCA

AGCCCTTACGACATATGGGCCACCGCCGACCTGGTGGAGCTGGTTAAGCAGCGCATTGAGGTCACGGATG

GAAGGCTACAAGCGGCCTTTGTCGTGTCGCGGGCGATCAAAGGCACGCGCATCGGCGGTGAGGTTGCCGA

GGCGCTGGCCGGGTACGAGCTGCCCATTCTTGAGTCCCGTATCACGCAGCGCGTGAGCTACCCAGGCACT

GCCGCCGCCGGCACAACCGTTCTTGAATCAGAACCCGAGGGCGACGCTGCCCGCGAGGTCCAGGCGCTGG

CCGCTGAAATTAAATCAAAACTCATTTGAGTTAATGAGGTAAAGAGAAAATGAGCAAAAGCACAAACACG

CTAAGTGCCGGCCGTCCGAGCGCACGCAGCAGCAAGGCTGCAACGTTGGCCAGCCTGGCAGACACGCCAG

CCATGAAGCGGGTCAACTTTCAGTTGCCGGCGGAGGATCACACCAAGCTGAAGATGTACGCGGTACGCCA

AGGCAAGACCATTACCGAGCTGCTATCTGAATACATCGCGCAGCTACCAGAGTAAATGAGCAAATGAATA

AATGAGTAGATGAATTTTAGCGGCTAAAGGAGGCGGCATGGAAAATCAAGAACAACCAGGCACCGACGCC

GTGGAATGCCCCATGTGTGGAGGAACGGGCGGTTGGCCAGGCGTAAGCGGCTGGGTTGTCTGCCGGCCCT

GCAATGGCACTGGAACCCCCAAGCCCGAGGAATCGGCGTGACGGTCGCAAACCATCCGGCCCGGTACAAA

TCGGCGCGGCGCTGGGTGATGACCTGGTGGAGAAGTTGAAGGCCGCGCAGGCCGCCCAGCGGCAACGCAT

CGAGGCAGAAGCACGCCCCGGTGAATCGTGGCAAGCGGCCGCTGATCGAATCCGCAAAGAATCCCGGCAA

CCGCCGGCAGCCGGTGCGCCGTCGATTAGGAAGCCGCCCAAGGGCGACGAGCAACCAGATTTTTTCGTTC

CGATGCTCTATGACGTGGGCACCCGCGATAGTCGCAGCATCATGGACGTGGCCGTTTTCCGTCTGTCGAA

GCGTGACCGACGAGCTGGCGAGGTGATCCGCTACGAGCTTCCAGACGGGCACGTAGAGGTTTCCGCAGGG

CCGGCCGGCATGGCCAGTGTGTGGGATTACGACCTGGTACTGATGGCGGTTTCCCATCTAACCGAATCCA

TGAACCGATACCGGGAAGGGAAGGGAGACAAGCCCGGCCGCGTGTTCCGTCCACACGTTGCGGACGTACT

CAAGTTCTGCCGGCGAGCCGATGGCGGAAAGCAGAAAGACGACCTGGTAGAAACCTGCATTCGGTTAAAC

ACCACGCACGTTGCCATGCAGCGTACGAAGAAGGCCAAGAACGGCCGCCTGGTGACGGTATCCGAGGGTG

AAGCCTTGATTAGCCGCTACAAGATCGTAAAGAGCGAAACCGGGCGGCCGGAGTACATCGAGATCGAGCT

AGCTGATTGGATGTACCGCGAGATCACAGAAGGCAAGAACCCGGACGTGCTGACGGTTCACCCCGATTAC

TTTTTGATCGATCCCGGCATCGGCCGTTTTCTCTACCGCCTGGCACGCCGCGCCGCAGGCAAGGCAGAAG

CCAGATGGTTGTTCAAGACGATCTACGAACGCAGTGGCAGCGCCGGAGAGTTCAAGAAGTTCTGTTTCAC

CGTGCGCAAGCTGATCGGGTCAAATGACCTGCCGGAGTACGATTTGAAGGAGGAGGCGGGGCAGGCTGGC

CCGATCCTAGTCATGCGCTACCGCAACCTGATCGAGGGCGAAGCATCCGCCGGTTCCTAATGTACGGAGC

AGATGCTAGGGCAAATTGCCCTAGCAGGGGAAAAAGGTCGAAAAGGTCTCTTTCCTGTGGATAGCACGTA

CATTGGGAACCCAAAGCCGTACATTGGGAACCGGAACCCGTACATTGGGAACCCAAAGCCGTACATTGGG

AACCGGTCACACATGTAAGTGACTGATATAAAAGAGAAAAAAGGCGATTTTTCCGCCTAAAACTCTTTAA

AACTTATTAAAACTCTTAAAACCCGCCTGGCCTGTGCATAACTGTCTGGCCAGCGCACAGCCGAAGAGCT

GCAAAAAGCGCCTACCCTTCGGTCGCTGCGCTCCCTACGCCCCGCCGCTTCGCGTCGGCCTATCGCGGCC

GCTGGCCGCTCAAAAATGGCTGGCCTACGGCCAGGCAATCTACCAGGGCGCGGACAAGCCGCGCCGTCGC

CACTCGACCGCCGGCGCCCACATCAAGGCACCCTGCCTCGCGCGTTTCGGTGATGACGGTGAAAACCTCT

GACACATGCAGCTCCCGGAGACGGTCACAGCTTGTCTGTAAGCGGATGCCGGGAGCAGACAAGCCCGTCA

GGGCGCGTCAGCGGGTGTTGGCGGGTGTCGGGGCGCAGCCATGACCCAGTCACGTAGCGATAGCGGAGTG

TATACTGGCTTAACTATGCGGCATCAGAGCAGATTGTACTGAGAGTGCACCATATGCGGTGTGAAATACC

GCACAGATGCGTAAGGAGAAAATACCGCATCAGGCGCTCTTCCGCTTCCTCGCTCACTGACTCGCTGCGC

TCGGTCGTTCGGCTGCGGCGAGCGGTATCAGCTCACTCAAAGGCGGTAATACGGTTATCCACAGAATCAG

GGGATAACGCAGGAAAGAACATGTGAGCAAAAGGCCAGCAAAAGGCCAGGAACCGTAAAAAGGCCGCGTT

GCTGGCGTTTTTCCATAGGCTCCGCCCCCCTGACGAGCATCACAAAAATCGACGCTCAAGTCAGAGGTGG

CGAAACCCGACAGGACTATAAAGATACCAGGCGTTTCCCCCTGGAAGCTCCCTCGTGCGCTCTCCTGTTC

CGACCCTGCCGCTTACCGGATACCTGTCCGCCTTTCTCCCTTCGGGAAGCGTGGCGCTTTCTCATAGCTC

ACGCTGTAGGTATCTCAGTTCGGTGTAGGTCGTTCGCTCCAAGCTGGGCTGTGTGCACGAACCCCCCGTT

CAGCCCGACCGCTGCGCCTTATCCGGTAACTATCGTCTTGAGTCCAACCCGGTAAGACACGACTTATCGC

CACTGGCAGCAGCCACTGGTAACAGGATTAGCAGAGCGAGGTATGTAGGCGGTGCTACAGAGTTCTTGAA

GTGGTGGCCTAACTACGGCTACACTAGAAGGACAGTATTTGGTATCTGCGCTCTGCTGAAGCCAGTTACC

TTCGGAAAAAGAGTTGGTAGCTCTTGATCCGGCAAACAAACCACCGCTGGTAGCGGTGGTTTTTTTGTTT

GCAAGCAGCAGATTACGCGCAGAAAAAAAGGATCTCAAGAAGATCCTTTGATCTTTTCTACGGGGTCTGA

CGCTCAGTGGAACGAAAACTCACGTTAAGGGATTTTGGTCATGCATGATATATCTCCCAATTTGTGTAGG

GCTTATTATGCACGCTTAAAAATAATAAAAGCAGACTTGACCTGATAGTTTGGCTGTGAGCAATTATGTG

CTTAGTGCATCTAATCGCTTGAGTTAACGCCGGCGAAGCGGCGTCGGCTTGAACGAATTTCTAGCTAGAG

GATCGCACCAATAACTGCCTTAAAAAAATTACGCCCCGCCCTGCCACTCATCGCAGTACTGTTGTAATTC

ATTAAGCATTCTGCCGACATGGAAGCCATCACAAACGGCATGATGAACCTGAATCGCCAGCGGCATCAGC

ACCTTGTCGCCTTGCGTATAATATTTGCCCATTGTGAAAACGGGGGCGAAGAAGTTGTCCATATTGGCCA

CGTTTAAATCAAAACTGGTGAAACTCACCCAGGGATTGGCTGAGACGAAAAACATATTCTCAATAAACCC

TTTAGGGAAATAGGCCAGGTTTTCACCGTAACACGCCACATCTTGCGAATATATGTGTAGAAACTGCCGG

AAATCGTCGTGGTATTCACTCCAGAGCGATGAAAACGTTTCAGTTTGCTCATGGAAAACGGTGTAACAAG

GGTGAACACTATCCCATATCACCAGCTCACCGTCTTTCATTGCCATACGGAACTCCGGATGAGCATTCAT

CAGGCGGGCAAGAATGTGAATAAAGGCCGGATAAAACTTGTGCTTATTTTTCTTTACGGTCTTTAAAAAG

GCCGTAATATCCAGCTGAACGGTCTGGTTATAGGTACATTGAGCAACTGACTGAAATGCCTCAAAATGTT

CTTTACGATGCCATTGGGATATATCAACGGTGGTATATCCAGTGATTTTTTTCTCCATGATGTTTAACTT

TGTTTTAGGGCGACTGCCCTGCTGCGTAACATCGTTGCTGCTCCATAACATCAAACATCGACCCACGGCG

TAACGCGCTTGCTGCTTGGATGCCCGAGGCATAGACTGTACCCCAAAAAAACATGTCATAACAAGAAGCC

ATGAAAACCGCCACTGCGCCGTTACCACCGCTGCGTTCGGTCAAGGTTCTGGACCAGTTGCGTGACGGCA

GTTACGCTACTTGCATTACAGCTTACGAACCGAACGAGGCTTATGTCCACTGGGTTCGTGCCCGAATTGA

TCACAGGCAGCAACGCTCTGTCATCGTTACAATCAACATGCTACCCTCCGCGAGATCATCCGTGTTTCAA

ACCCGGCAGCTTAGTTGCCGTTCTTCCGAATAGCATCGGTAACATGAGCAAAGTCTGCCGCCTTACAACG

GCTCTCCCGCGCTGGTGGCAGGATATATTGTGGTGTAAACAAATTGACGCTTAGACAACTTAATAACACA

TTGCGGACGTTTTTAATGTACTGAATTAACGCCGAATTAATTCGGGGGATCTGGATTTTAGTACTGGATT

TTGGTTTTAGGAATTAGAAATTTTATTGATAGAAGTATTTTACAAATACAAATACATACTAAGGGTTTCT

TATATGCTCAACACATGAGCGAAACCCTATAGGAACCCTAATTCCCTTATCTGGGAACTACTCACACATT

ATTATGGAGAAACTCGAGTCAGAAGAACTCGTCAAGAAGGCGATAGAAGGCGATCCGCTGCGAATCGGGA

GCGGCGATACCGTAAAGCACGAGGAAGCGGTCAGCCCATTCGCCGCCAAGCTCTTCAGCAATATCACGGG

TAGCCAACGCTATGTCCTGATAGCGGTCCGCCACACCCAGCCGGCCACAGTCGATGAATCCAGAAAAGCG

TATAGTGCCCATCGGTTGCGATACAGGACTATCGCCAGGCGGTGTGGGTCGGCCGGTGTCAGCTACTTAG

GTCTTTTCGCGCCATTTTCCACCATGATATTCGGCAAGCAGGCATCGCCATGGGTCACGACGAGATCCTC

GCCGTCGGGCATGCGCGCCTTGAGCCTGGCGAACAGTTCGGCTGGCGCGAGCCCCTGATGCTCTTCGTCC

AGATCATCCTGATCGACAAGACCGGCTTCCACTCGGACCGCTTGTCAAGCCGACCGCGCTCGGGGACTAC

GAGAAGCAGGTCTAGTAGGACTAGCTGTTCTGGCCGAAGGATCCGAGTACGTGCTCGCTCGATGCGATGT

TTCGCTTGGTGGTCGTATGGGCAGGTAGCCGGATCAAGCGTATGCAGCCGCCGCATTGCATCAGCCATGA

TGGATACTTTCTCGGCAGGAGCAAGGTGAGATGACAGGAGATCCTGCCCCGGCACTTCGCCCAATAGCAG

CCAGTCCCTTCCCGCTTCAGTGACAACGTCGAGCACAGCTGCGCAAGGAACGCCCGTCGTGGCCAGCCAC

GATAGCCGCGCTGCCTCGTCTTGCAGTTCATTCAGGGCACCGGACAGGTCGGTCTTGACAAAAAGAACCG

GGCGCCCCTGCGCTGACAGCCGGAACACGGCGGCATCAGAGCAGCCGATTGTCTGTTGTGCCCAGTCATA

GCCGAATAGCCTCTCCACCCAAGCGGCCGGAGAACCTGCGTGCAATCCATCTTGTTCAATTCGACGGTTC

TGTAACTATCATCATCATCATAGACACACGAAATAAAGTAATCAGATTATCAGTTAAAGCTATGTAATAT

TTACACCATAACCAATCAATTAAAAAATAGATCAGTTTAAAGAAAGATCAAAGCTCAAAAAAATAAAAAG

AGAAAAGGGTCCTAACCAAGAAAATGAAGGAGAAAAACTAGAAATTTACCGTCAGATCCATTGTTGGGCC

CTGTAATTGTAAATAGTAATTGTAATGTTGTTTGTTGTTTGTTGTTGTTGGTAATTGTTGTAAAAATACG

TGTCCTCTCCAAATGAAATGAACTTCCTTATATAGAGGAAGGGTCTTGCGAAGGATAGTGGGATTGTGCG

TCATCCCTTACGTCAGTGGAGATATCACATCAATCCACTTGCTTTGAAGACGTGGTTGGAACGTCTTCTT

TTTCCACGATGCTCCTCGTGGGTGGGGGTCCATCTTTGGGACCACTGTCGGCAGAGGCATCTTCAACGAT

GGCCTTTCCTTTATCGCAATGATGGCATTTGTAGGAGCCACCTTCCTTTTCCACTATCTTCACAATAAAG

TGACAGATAGCTGGGCAATGGAATCCGAGGAGGTTTCCGGATATTACCCTTTGTTGAAAAGTCTCAATGA

ATTCCCGGGAGGCCTACTAGTCGACCGCGGCGATCTAGTAACATAGATGACACCGCGCGCGATAATTTAT

CCTAGTTTGCGCGCTATATTTTGTTTTCTATCGCGTATTAAATGTATAATTGCGGGACTCTAATCATAAA

AACCCATCTCATAAATAACGTCATGCATTACATGTTAATTATTACATGCTTAACGTAATTCAACAGAAAT

TATATGATAATCATCGCAAGACCGGCAACAGGATTCAATCTTAAGAAACTTTATTGCCAAATGTTTGAAC

GATCGGGGAAATTCGAGCTCTCATTGTTTGCCTCCCTGCTGCGGTTTTTCACCGAAGTTCATGCCAGTCC

AGCGTTTTTGCAGCAGAAAAGCCGCCGACTTCGGTTTGCGGTCGCGAGTGAAGATCCCTTTCTTGTTACC

GCCAACGCGCAATATGCCTTGCGAGGTCGCAAAATCGGCGAAATTCCATACCTGTTCACCGACGACGGCG

CTGACGCGATCAAAGACGCGGTGATACATATCCAGCCATGCACACTGATACTCTTCACTCCACATGTCGG

TGTACATTGAGTGCAGCCCGGCTAACGTATCCACGCCGTATTCGGTGATGATAATCGGCTGATGCAGTTT

CTCCTGCCAGGCCAGAAGTTCTTTTTCCAGTACCTTCTCTGCCGTTTCCAAATCGCCGCTTTGGACATAC

CATCCGTAATAACGGTTCAGGCACAGCACATCAAAGAGATCGCTGATGGTATCGGTGTGAGCGTCGCAGA

ACATTACATTGACGCAGGTGATCGGACGCGTCGGGTCGAGTTTACGCGTTGCTTCCGCCAGTGGCGCGAA

ATATTCCCGTGCACCTTGCGGACGGGTATCCGGTTCGTTGGCAATACTCCACATCACCACGCTTGGGTGG

TTTTTGTCACGCGCTATCAGCTCTTTAATCGCCTGTAAGTGCGCTTGCTGAGTTTCCCCGTTGACTGCCT

CTTCGCTGTACAGTTCTTTCGGCTTGTTGCCCGCTTCGAAACCAATGCCTAAAGAGAGGTTAAAGCCGAC

AGCAGCAGTTTCATCAATCACCACGATGCCATGTTCATCTGCCCAGTCGAGCATCTCTTCAGCGTAAGGG

TAATGCGAGGTACGGTAGGAGTTGGCCCCAATCCAGTCCATTAATGCGTGGTCGTGCACCATCAGCACGT

TATCGAATCCTTTGCCACGCAAGTCCGCATCTTCATGACGACCAAAGCCAGTAAAGTAGAACGGTTTGTG

GTTAATCAGGAACTGTTCGCCCTTCACTGCCACTGACCGGATGCCGACGCGAAGCGGGTAGATATCACAC

TCTGTCTGGCTTTTGGCTGTGACGCACAGTTCATAGAGATAACCTTCACCCGGTTGCCAGAGGTGCGGAT

TCACCACTTGCAAAGTCCCGCTAGTGCCTTGTCCAGTTGCAACCACCTGTTGATCCGCATCACGCAGTTC

AACGCTGACATCACCATTGGCCACCACCTGCCAGTCAACAGACGCGTGGTTACAGTCTTGCGCGACATGC

GTCACCACGGTGATATCGTCCACCCAGGTGTTCGGCGTGGTGTAGAGCATTACGCTGCGATGGATTCCGG

CATAGTTAAAGAAATCATGGAAGTAAGACTGCTTTTTCTTGCCGTTTTCGTCGGTAATCACCATTCCCGG

CGGGATAGTCTGCCAGTTCAGTTCGTTGTTCACACAAACGGTGATACGTACACTTTTCCCGGCAATAACA

TACGGCGTGACATCGGCTTCAAATGGCGTATAGCCGCCCTGATGCTCCATCACTTCCTGATTATTGACCC

ACACTTTGCCGTAATGAGTGACCGCATCGAAACGCAGCACGATACGCTGGCCTGCCCAACCTTTCGGTAT

AAAGACTTCGCGCTGATACCAGACGTTGCCCGCATAATTACGAATATCTGCATCGGCGAACTGATCGTTA

AAACTGCCTGGCACAGCAATTGCCCGGCTTTCTTGTAACGCGCTTTCCCACCAACGCTGATCAATTCCAC

AGTTTTCGCGATCCAGACTGAATGCCCACAGGCCGTCGAGTTTTTTGATTTCACGGGTTGGGGTTTCTAC

AGGACGTAACATGGATCCTGTAATTGTAAATAGTAATTGTAATGTTGTTTGTTGTTTGTTGTTGTTGGTA

ATTGTTGTAAAAATACGTGTCCTCTCCAAATGAAATGAACTTCCTTATATAGAGGAAGGGTCTTGCGAAG

GATAGTGGGATTGTGCGTCATCCCTTACGTCAGTGGAGATATCACATCAATCCACTTGCTTTGAAGACGT

GGTTGGAACGTCTTCTTTTTCCACGATGCTCCTCGTGGGTGGGGGTCCATCTTTGGGACCACTGTCGGCA

GAGGCATCTTCAACGATGGCCTTTCCTTTATCGCAATGATGGCATTTGTAGGAGCCACCTTCCTTTTCCA

CTATCTTCACAATAAAGTGACAGATAGCTGGGCAATGGAATCCGAGGAGGTTTCCGGATATTACCCTTTG

TTGAAAAGTCTCAATGAATTAATTCGGGTCTTGCGAAGGATAGTGGGATTGTGCGTCATCCCTTACGTCA

GTGGAGATATCACATCAATCCACTTGCTTTGAAGACGTGGTTGGAACGTCTTCTTTTTCCACGATGCTCC

TCGTGGGTGGGGGTCCATCTTTGGGACCACTGTCGGCAGAGGCATCTTCAACGATGGCCTTTCCTTTATC

GCAATGATGGCATTTGTAGGAGCCACCTTCCTTTTCCACTATCTTCACAATAAAGTGACAGATAGCTGGG

CAATGGAATCCGAGGAGGTTTCCGGATATTACCCTTTGTTGAAAAGTCTCAATAAGCTTCTGCAGCATGC

ACTATTTTCAGAAGAAGTTCCCAATAGTAGTCCAAAATTTTTGTAACGAAGGGAGCATAATAGTTACATG

CAAAGGAAAACTGCCATTCTTTAGAGGGGATGCTTGTTTAAGAACAAAAAATATATCACTTTCTTTTGTT

CCAAGTCATTGCGTATTTTTTTAAAAATATTTGTTCCTTCGTATATTTCGAGCTTCAATCACTTTATGGT

TCTTTGTATTCTGGCTTTGCTGTAAATCGTAGCTAACCTTCTTCCTAGCAGAAATTATTAATACTTGGGA

TATTTTTTTAGAATCAAGTAAATTACATATTACCACCACATCGAGCTGCTTTTAAATTCATATTACAGCC

ATATAGGCTTGATTCATTTTGCAAAATTTCCAGGATATTGACAACGTTAACTTAATAATATCTTGAAATA

TTAAAGCTATTATGATTAGGGGTGCAAATGGACCGAGTTGGTTCGGTTTATATCAAAATCAAACCAAACC

AACTATATCGGTTTGGATTGGTTCGGTTTTGCCGGGTTTTCAGCATTTTCTGGTTTTTTTTTTGTTAGAT

GAATATTATTTTAATCTTACTTTGTCAAATTTTTGATAAGTAAATATATGTGTTAGTAAAAATTAATTTT

TTTTACAAACATATGATCTATTAAAATATTCTTATAGGAGAATTTTCTTAATAACACATGATATTTATTT

ATTTTAGTCGTTTGACTAATTTTTCGTTGATGTACACTTTCAAAGTTAACCAAATTTAGTAATTAAGTAT

AAAAATCAATATGATACCTAAATAATGATATGTTCTATTTAATTTTAAATTATCGAAATTTCACTTCAAA

TTCGAAAAAGATATATAAGAATTTTGATAGATTTTGACATATGAATATGGAAGAACAAAGAGATTGACGC

ATTTTAGTAACACTTGATAAGAAAGTGATCGTACAACCAATTATTTAAAGTTAATAAAAATGGAGCACTT

CATATTTAACGAAATATTACATGCCAGAAGAGTCGCAAATATTTCTAGATATTTTTTAAAGAAAATTCTA

TAAAAAGTCTTAAAGGCATATATATAAAAACTATATATTTATATTTTGGTTTGGTTCGAATTTGTTTTAC

TCAATACAAAACTAAATTAGACCAAATATAATTGGGATTTTTAATCGAGGTACCGGCACTGGCCGTCGTT

TTACAACGTCGTGACTGGGAAAACCCTGGCGTTACCCAACTTAATCGCCTTGCAGCACATCCCCCTTTCG

CCAGCTGGCGTAATAGCGAAGAGGCCCGCACCGATCGCCCTTCCCAACAGTTGCGCAGCCTGAATGGCGA

ATGCTAGAGCAGCTTGAGCTTGGATCAGATTGTCGTTTCCCGCCTTCAGTTTAGCTTCATGGAGTCAAAG

ATTCAAATAGAGGACCTAACAGAACTCGCCGTAAAGACTGGCGAACAGTTCATACAGAGTCTCTTACGAC

TCAATGACAAGAAGAAAATCTTCGTCAACATGGTGGAGCACGACACACTTGTCTACTCCAAAAATATCAA

AGATACAGTCTCAGAAGACCAAAGGGCAATTGAGACTTTTCAACAAAGGGTAATATCCGGAAACCTCCTC

GGATTCCATTGCCCAGCTATCTGTCACTTTATTGTGAAGATAGTGGAAAAGGAAGGTGGCTCCTACAAAT

GCCATCATTGCGATAAAGGAAAGGCCATCGTTGAAGATGCCTCTGCCGACAGTGGTCCCAAAGATGGACC

CCCACCCACGAGGAGCATCGTGGAAAAAGAAGACGTTCCAACCACGTCTTCAAAGCAAGTGGATTGATGT

GATATCTCCACTGACGTAAGGGATGACGCACAATCCCACTATCCTTCGCAAGACCCTTCCTCTATATAAG

GAAGTTCATTTCATTTGGAGAGAACACGGGGGACTCTTGACCATGGTAGATCTGAGGGTAAATTTCTAGT

TTTTCTCCTTCATTTTCTTGGTTAGGACCCTTTTCTCTTTTTATTTTTTTGAGCTTTGATCTTTCTTTAA

ACTGATCTATTTTTTAATTGATTGGTTATGGTGTAAATATTACATAGCTTTAACTGATAATCTGATTACT

TTATTTCGTGTGTCTATGATGATGATGATAGTTACAGAACCGACGACTCGTCCGTCCTGTAGAAACCCCA

ACCCGTGAAATCAAAAAACTCGACGGCCTGTGGGCATTCAGTCTGGATCGCGAAAACTGTGGAATTGATC

AGCGTTGGTGGGAAAGCGCGTTACAAGAAAGCCGGGCAATTGCTGTGCCAGGCAGTTTTAACGATCAGTT

CGCCGATGCAGATATTCGTAATTATGCGGGCAACGTCTGGTATCAGCGCGAAGTCTTTATACCGAAAGGT

TGGGCAGGCCAGCGTATCGTGCTGCGTTTCGATGCGGTCACTCATTACGGCAAAGTGTGGGTCAATAATC

AGGAAGTGATGGAGCATCAGGGCGGCTATACGCCATTTGAAGCCGATGTCACGCCGTATGTTATTGCCGG

GAAAAGTGTACGTATCACCGTTTGTGTGAACAACGAACTGAACTGGCAGACTATCCCGCCGGGAATGGTG

ATTACCGACGAAAACGGCAAGAAAAAGCAGTCTTACTTCCATGATTTCTTTAACTATGCCGGAATCCATC

GCAGCGTAATGCTCTACACCACGCCGAACACCTGGGTGGACGATATCACCGTGGTGACGCATGTCGCGCA

AGACTGTAACCACGCGTCTGTTGACTGGCAGGTGGTGGCCAATGGTGATGTCAGCGTTGAACTGCGTGAT

GCGGATCAACAGGTGGTTGCAACTGGACAAGGCACTAGCGGGACTTTGCAAGTGGTGAATCCGCACCTCT

GGCAACCGGGTGAAGGTTATCTCTATGAACTCGAAGTCACAGCCAAAAGCCAGACAGAGTCTGATATCTA

CCCGCTTCGCGTCGGCATCCGGTCAGTGGCAGTGAAGGGCCAACAGTTCCTGATTAACCACAAACCGTTC

TACTTTACTGGCTTTGGTCGTCATGAAGATGCGGACTTACGTGGCAAAGGATTCGATAACGTGCTGATGG

TGCACGACCACGCATTAATGGACTGGATTGGGGCCAACTCCTACCGTACCTCGCATTACCCTTACGCTGA

AGAGATGCTCGACTGGGCAGATGAACATGGCATCGTGGTGATTGATGAAACTGCTGCTGTCGGCTTTCAG

CTGTCTTTAGGCATTGGTTTCGAAGCGGGCAACAAGCCGAAAGAACTGTACAGCGAAGAGGCAGTCAACG

GGGAAACTCAGCAAGCGCACTTACAGGCGATTAAAGAGCTGATAGCGCGTGACAAAAACCACCCAAGCGT

GGTGATGTGGAGTATTGCCAACGAACCGGATACCCGTCCGCAAGGTGCACGGGAATATTTCGCGCCACTG

GCGGAAGCAACGCGTAAACTCGACCCGACGCGTCCGATCACCTGCGTCAATGTAATGTTCTGCGACGCTC

ACACCGATACCATCAGCGATCTCTTTGATGTGCTGTGCCTGAACCGTTATTACGGATGGTATGTCCAAAG

CGGCGATTTGGAAACGGCAGAGAAGGTACTGGAAAAAGAACTTCTGGCCTGGCAGGAGAAACTGCATCAG

CCGATTATCATCACCGAATACGGCGTGGATACGTTAGCCGGGCTGCACTCAATGTACACCGACATGTGGA

GTGAAGAGTATCAGTGTGCATGGCTGGATATGTATCACCGCGTCTTTGATCGCGTCAGCGCCGTCGTCGG

TGAACAGGTATGGAATTTCGCCGATTTTGCGACCTCGCAAGGCATATTGCGCGTTGGCGGTAACAAGAAA

GGGATCTTCACTCGCGACCGCAAACCGAAGTCGGCGGCTTTTCTGCTGCAAAAACGCTGGACTGGCATGA

ACTTCGGTGAAAAACCGCAGCAGGGAGGCAAACAAGCTAGCCACCACCACCACCACCACGTGTGAATTAC

AGGTGACCAGCTCGAATTTCCCCGATCGTTCAAACATTTGGCAATAAAGTTTCTTAAGATTGAATCCTGT

TGCCGGTCTTGCGATGATTATCATATAATTTCTGTTGAATTACGTTAAGCATGTAATAATTAACATGTAA

TGCATGACGTTATTTATGAGATGGGTTTTTATGATTAGAGTCCCGCAATTATACATTTAATACGCGATAG

AAAACAAAATATAGCGCGCAAACTAGGATAAATTATCGCGCGCGGTGTCATCTATGTTACTAGATCGGGA

ATTAAACTATCAGTGTTTGACAGGATATATTGGCGGGTAAACCTAAGAGAAAAGAGCGTTTATTAGAATA

ACGGATATTTAAAAGGGCGTGAAAAGGTTTATCCGTTCGTCCATTTGTATGT

>gi|29568874|gb|AY178048.1| Expression vector pYPX145, complete sequence

GCATGCCAACCACAGGGTTCCCCTCGGGATCAAAGTACTTTGATCCAACCCCTCCGCTGCTATAGTGCAG

TCGGCTTCTGACGTTCAGTGCAGCCGTCTTCTGAAAACGACATGTCGCACAAGTCCTAAGTTACGCGACA

GGCTGCCGCCCTGCCCTTTTCCTGGCGTTTTCTTGTCGCGTGTTTTAGTCGCATAAAGTAGAATACTTGC

GACTAGAACCGGAGACATTACGCCATGAACAAGAGCGCCGCCGCTGGCCTGCTGGGCTATGCCCGCGTCA

GCACCGACGACCAGGACTTGACCAACCAACGGGCCGAACTGCACGCGGCCGGCTGCACCAAGCTGTTTTC

CGAGAAGATCACCGGCACCAGGCGCGACCGCCCGGAGCTGGCCAGGATGCTTGACCACCTACGCCCTGGC

GACGTTGTGACAGTGACCAGGCTAGACCGCCTGGCCCGCAGCACCCGCGACCTACTGGACATTGCCGAGC

GCATCCAGGAGGCCGGCGCGGGCCTGCGTAGCCTGGCAGAGCCGTGGGCCGACACCACCACGCCGGCCGG

CCGCATGGTGTTGACCGTGTTCGCCGGCATTGCCGAGTTCGAGCGTTCCCTAATCATCGACCGCACCCGG

AGCGGGCGCGAGGCCGCCAAGGCCCGAGGCGTGAAGTTTGGCCCCCGCCCTACCCTCACCCCGGCACAGA

TCGCGCACGCCCGCGAGCTGATCGACCAGGAAGGCCGCACCGTGAAAGAGGCGGCTGCACTGCTTGGCGT

GCATCGCTCGACCCTGTACCGCGCACTTGAGCGCAGCGAGGAAGTGACGCCCACCGAGGCCAGGCGGCGC

GGTGCCTTCCGTGAGGACGCATTGACCGAGGCCGACGCCCTGGCGGCCGCCGAGAATGAACGCCAAGAGG

AACAAGCATGAAACCGCACCAGGACGGCCAGGACGAACCGTTTTTCATTACCGAAGAGATCGAGGCGGAG

ATGATCGCGGCCGGGTACGTGTTCGAGCCGCCCGCGCACGTCTCAACCGTGCGGCTGCATGAAATCCTGG

CCGGTTTGTCTGATGCCAAGCTGGCGGCCTGGCCGGCCAGCTTGGCCGCTGAAGAAACCGAGCGCCGCCG

TCTAAAAAGGTGATGTGTATTTGAGTAAAACAGCTTGCGTCATGCGGTCGCTGCGTATATGATGCGATGA

GTAAATAAACAAATACGCAAGGGGAACGCATGAAGGTTATCGCTGTACTTAACCAGAAAGGCGGGTCAGG

CAAGACGACCATCGCAACCCATCTAGCCCGCGCCCTGCAACTCGCCGGGGCCGATGTTCTGTTAGTCGAT

TCCGATCCCCAGGGCAGTGCCCGCGATTGGGCGGCCGTGCGGGAAGATCAACCGCTAACCGTTGTCGGCA

TCGACCGCCCGACGATTGACCGCGACGTGAAGGCCATCGGCCGGCGCGACTTCGTAGTGATCGACGGAGC

GCCCCAGGCGGCGGACTTGGCTGTGTCCGCGATCAAGGCAGCCGACTTCGTGCTGATTCCGGTGCAGCCA

AGCCCTTACGACATATGGGCCACCGCCGACCTGGTGGAGCTGGTTAAGCAGCGCATTGAGGTCACGGATG

GAAGGCTACAAGCGGCCTTTGTCGTGTCGCGGGCGATCAAAGGCACGCGCATCGGCGGTGAGGTTGCCGA

GGCGCTGGCCGGGTACGAGCTGCCCATTCTTGAGTCCCGTATCACGCAGCGCGTGAGCTACCCAGGCACT

GCCGCCGCCGGCACAACCGTTCTTGAATCAGAACCCGAGGGCGACGCTGCCCGCGAGGTCCAGGCGCTGG

CCGCTGAAATTAAATCAAAACTCATTTGAGTTAATGAGGTAAAGAGAAAATGAGCAAAAGCACAAACACG

CTAAGTGCCGGCCGTCCGAGCGCACGCAGCAGCAAGGCTGCAACGTTGGCCAGCCTGGCAGACACGCCAG

CCATGAAGCGGGTCAACTTTCAGTTGCCGGCGGAGGATCACACCAAGCTGAAGATGTACGCGGTACGCCA

AGGCAAGACCATTACCGAGCTGCTATCTGAATACATCGCGCAGCTACCAGAGTAAATGAGCAAATGAATA

AATGAGTAGATGAATTTTAGCGGCTAAAGGAGGCGGCATGGAAAATCAAGAACAACCAGGCACCGACGCC

GTGGAATGCCCCATGTGTGGAGGAACGGGCGGTTGGCCAGGCGTAAGCGGCTGGGTTGTCTGCCGGCCCT

GCAATGGCACTGGAACCCCCAAGCCCGAGGAATCGGCGTGACGGTCGCAAACCATCCGGCCCGGTACAAA

TCGGCGCGGCGCTGGGTGATGACCTGGTGGAGAAGTTGAAGGCCGCGCAGGCCGCCCAGCGGCAACGCAT

CGAGGCAGAAGCACGCCCCGGTGAATCGTGGCAAGCGGCCGCTGATCGAATCCGCAAAGAATCCCGGCAA

CCGCCGGCAGCCGGTGCGCCGTCGATTAGGAAGCCGCCCAAGGGCGACGAGCAACCAGATTTTTTCGTTC

CGATGCTCTATGACGTGGGCACCCGCGATAGTCGCAGCATCATGGACGTGGCCGTTTTCCGTCTGTCGAA

GCGTGACCGACGAGCTGGCGAGGTGATCCGCTACGAGCTTCCAGACGGGCACGTAGAGGTTTCCGCAGGG

CCGGCCGGCATGGCCAGTGTGTGGGATTACGACCTGGTACTGATGGCGGTTTCCCATCTAACCGAATCCA

TGAACCGATACCGGGAAGGGAAGGGAGACAAGCCCGGCCGCGTGTTCCGTCCACACGTTGCGGACGTACT

CAAGTTCTGCCGGCGAGCCGATGGCGGAAAGCAGAAAGACGACCTGGTAGAAACCTGCATTCGGTTAAAC

ACCACGCACGTTGCCATGCAGCGTACGAAGAAGGCCAAGAACGGCCGCCTGGTGACGGTATCCGAGGGTG

AAGCCTTGATTAGCCGCTACAAGATCGTAAAGAGCGAAACCGGGCGGCCGGAGTACATCGAGATCGAGCT

AGCTGATTGGATGTACCGCGAGATCACAGAAGGCAAGAACCCGGACGTGCTGACGGTTCACCCCGATTAC

TTTTTGATCGATCCCGGCATCGGCCGTTTTCTCTACCGCCTGGCACGCCGCGCCGCAGGCAAGGCAGAAG

CCAGATGGTTGTTCAAGACGATCTACGAACGCAGTGGCAGCGCCGGAGAGTTCAAGAAGTTCTGTTTCAC

CGTGCGCAAGCTGATCGGGTCAAATGACCTGCCGGAGTACGATTTGAAGGAGGAGGCGGGGCAGGCTGGC

CCGATCCTAGTCATGCGCTACCGCAACCTGATCGAGGGCGAAGCATCCGCCGGTTCCTAATGTACGGAGC

AGATGCTAGGGCAAATTGCCCTAGCAGGGGAAAAAGGTCGAAAAGGTCTCTTTCCTGTGGATAGCACGTA

CATTGGGAACCCAAAGCCGTACATTGGGAACCGGAACCCGTACATTGGGAACCCAAAGCCGTACATTGGG

AACCGGTCACACATGTAAGTGACTGATATAAAAGAGAAAAAAGGCGATTTTTCCGCCTAAAACTCTTTAA

AACTTATTAAAACTCTTAAAACCCGCCTGGCCTGTGCATAACTGTCTGGCCAGCGCACAGCCGAAGAGCT

GCAAAAAGCGCCTACCCTTCGGTCGCTGCGCTCCCTACGCCCCGCCGCTTCGCGTCGGCCTATCGCGGCC

GCTGGCCGCTCAAAAATGGCTGGCCTACGGCCAGGCAATCTACCAGGGCGCGGACAAGCCGCGCCGTCGC

CACTCGACCGCCGGCGCCCACATCAAGGCACCCTGCCTCGCGCGTTTCGGTGATGACGGTGAAAACCTCT

GACACATGCAGCTCCCGGAGACGGTCACAGCTTGTCTGTAAGCGGATGCCGGGAGCAGACAAGCCCGTCA

GGGCGCGTCAGCGGGTGTTGGCGGGTGTCGGGGCGCAGCCATGACCCAGTCACGTAGCGATAGCGGAGTG

TATACTGGCTTAACTATGCGGCATCAGAGCAGATTGTACTGAGAGTGCACCATATGCGGTGTGAAATACC

GCACAGATGCGTAAGGAGAAAATACCGCATCAGGCGCTCTTCCGCTTCCTCGCTCACTGACTCGCTGCGC

TCGGTCGTTCGGCTGCGGCGAGCGGTATCAGCTCACTCAAAGGCGGTAATACGGTTATCCACAGAATCAG

GGGATAACGCAGGAAAGAACATGTGAGCAAAAGGCCAGCAAAAGGCCAGGAACCGTAAAAAGGCCGCGTT

GCTGGCGTTTTTCCATAGGCTCCGCCCCCCTGACGAGCATCACAAAAATCGACGCTCAAGTCAGAGGTGG

CGAAACCCGACAGGACTATAAAGATACCAGGCGTTTCCCCCTGGAAGCTCCCTCGTGCGCTCTCCTGTTC

CGACCCTGCCGCTTACCGGATACCTGTCCGCCTTTCTCCCTTCGGGAAGCGTGGCGCTTTCTCATAGCTC

ACGCTGTAGGTATCTCAGTTCGGTGTAGGTCGTTCGCTCCAAGCTGGGCTGTGTGCACGAACCCCCCGTT

CAGCCCGACCGCTGCGCCTTATCCGGTAACTATCGTCTTGAGTCCAACCCGGTAAGACACGACTTATCGC

CACTGGCAGCAGCCACTGGTAACAGGATTAGCAGAGCGAGGTATGTAGGCGGTGCTACAGAGTTCTTGAA

GTGGTGGCCTAACTACGGCTACACTAGAAGGACAGTATTTGGTATCTGCGCTCTGCTGAAGCCAGTTACC

TTCGGAAAAAGAGTTGGTAGCTCTTGATCCGGCAAACAAACCACCGCTGGTAGCGGTGGTTTTTTTGTTT

GCAAGCAGCAGATTACGCGCAGAAAAAAAGGATCTCAAGAAGATCCTTTGATCTTTTCTACGGGGTCTGA

CGCTCAGTGGAACGAAAACTCACGTTAAGGGATTTTGGTCATGCATGATATATCTCCCAATTTGTGTAGG

GCTTATTATGCACGCTTAAAAATAATAAAAGCAGACTTGACCTGATAGTTTGGCTGTGAGCAATTATGTG

CTTAGTGCATCTAATCGCTTGAGTTAACGCCGGCGAAGCGGCGTCGGCTTGAACGAATTTCTAGCTAGAG

GATCGCACCAATAACTGCCTTAAAAAAATTACGCCCCGCCCTGCCACTCATCGCAGTACTGTTGTAATTC

ATTAAGCATTCTGCCGACATGGAAGCCATCACAAACGGCATGATGAACCTGAATCGCCAGCGGCATCAGC

ACCTTGTCGCCTTGCGTATAATATTTGCCCATTGTGAAAACGGGGGCGAAGAAGTTGTCCATATTGGCCA

CGTTTAAATCAAAACTGGTGAAACTCACCCAGGGATTGGCTGAGACGAAAAACATATTCTCAATAAACCC

TTTAGGGAAATAGGCCAGGTTTTCACCGTAACACGCCACATCTTGCGAATATATGTGTAGAAACTGCCGG

AAATCGTCGTGGTATTCACTCCAGAGCGATGAAAACGTTTCAGTTTGCTCATGGAAAACGGTGTAACAAG

GGTGAACACTATCCCATATCACCAGCTCACCGTCTTTCATTGCCATACGGAACTCCGGATGAGCATTCAT

CAGGCGGGCAAGAATGTGAATAAAGGCCGGATAAAACTTGTGCTTATTTTTCTTTACGGTCTTTAAAAAG

GCCGTAATATCCAGCTGAACGGTCTGGTTATAGGTACATTGAGCAACTGACTGAAATGCCTCAAAATGTT

CTTTACGATGCCATTGGGATATATCAACGGTGGTATATCCAGTGATTTTTTTCTCCATGATGTTTAACTT

TGTTTTAGGGCGACTGCCCTGCTGCGTAACATCGTTGCTGCTCCATAACATCAAACATCGACCCACGGCG

TAACGCGCTTGCTGCTTGGATGCCCGAGGCATAGACTGTACCCCAAAAAAACATGTCATAACAAGAAGCC

ATGAAAACCGCCACTGCGCCGTTACCACCGCTGCGTTCGGTCAAGGTTCTGGACCAGTTGCGTGACGGCA

GTTACGCTACTTGCATTACAGCTTACGAACCGAACGAGGCTTATGTCCACTGGGTTCGTGCCCGAATTGA

TCACAGGCAGCAACGCTCTGTCATCGTTACAATCAACATGCTACCCTCCGCGAGATCATCCGTGTTTCAA

ACCCGGCAGCTTAGTTGCCGTTCTTCCGAATAGCATCGGTAACATGAGCAAAGTCTGCCGCCTTACAACG

GCTCTCCCGCGCTGGTGGCAGGATATATTGTGGTGTAAACAAATTGACGCTTAGACAACTTAATAACACA

TTGCGGACGTTTTTAATGTACTGAATTAACGCCGAATTAATTCGGGGGATCTGGATTTTAGTACTGGATT

TTGGTTTTAGGAATTAGAAATTTTATTGATAGAAGTATTTTACAAATACAAATACATACTAAGGGTTTCT

TATATGCTCAACACATGAGCGAAACCCTATAGGAACCCTAATTCCCTTATCTGGGAACTACTCACACATT

ATTATGGAGAAACTCGAGTCAGAAGAACTCGTCAAGAAGGCGATAGAAGGCGATCCGCTGCGAATCGGGA

GCGGCGATACCGTAAAGCACGAGGAAGCGGTCAGCCCATTCGCCGCCAAGCTCTTCAGCAATATCACGGG

TAGCCAACGCTATGTCCTGATAGCGGTCCGCCACACCCAGCCGGCCACAGTCGATGAATCCAGAAAAGCG

TATAGTGCCCATCGGTTGCGATACAGGACTATCGCCAGGCGGTGTGGGTCGGCCGGTGTCAGCTACTTAG

GTCTTTTCGCGCCATTTTCCACCATGATATTCGGCAAGCAGGCATCGCCATGGGTCACGACGAGATCCTC

GCCGTCGGGCATGCGCGCCTTGAGCCTGGCGAACAGTTCGGCTGGCGCGAGCCCCTGATGCTCTTCGTCC

AGATCATCCTGATCGACAAGACCGGCTTCCACTCGGACCGCTTGTCAAGCCGACCGCGCTCGGGGACTAC

GAGAAGCAGGTCTAGTAGGACTAGCTGTTCTGGCCGAAGGATCCGAGTACGTGCTCGCTCGATGCGATGT

TTCGCTTGGTGGTCGTATGGGCAGGTAGCCGGATCAAGCGTATGCAGCCGCCGCATTGCATCAGCCATGA

TGGATACTTTCTCGGCAGGAGCAAGGTGAGATGACAGGAGATCCTGCCCCGGCACTTCGCCCAATAGCAG

CCAGTCCCTTCCCGCTTCAGTGACAACGTCGAGCACAGCTGCGCAAGGAACGCCCGTCGTGGCCAGCCAC

GATAGCCGCGCTGCCTCGTCTTGCAGTTCATTCAGGGCACCGGACAGGTCGGTCTTGACAAAAAGAACCG

GGCGCCCCTGCGCTGACAGCCGGAACACGGCGGCATCAGAGCAGCCGATTGTCTGTTGTGCCCAGTCATA

GCCGAATAGCCTCTCCACCCAAGCGGCCGGAGAACCTGCGTGCAATCCATCTTGTTCAATTCGACGGTTC

TGTAACTATCATCATCATCATAGACACACGAAATAAAGTAATCAGATTATCAGTTAAAGCTATGTAATAT

TTACACCATAACCAATCAATTAAAAAATAGATCAGTTTAAAGAAAGATCAAAGCTCAAAAAAATAAAAAG

AGAAAAGGGTCCTAACCAAGAAAATGAAGGAGAAAAACTAGAAATTTACCGTCAGATCCATTGTTGGGCC

CTGTAATTGTAAATAGTAATTGTAATGTTGTTTGTTGTTTGTTGTTGTTGGTAATTGTTGTAAAAATACG

TGTCCTCTCCAAATGAAATGAACTTCCTTATATAGAGGAAGGGTCTTGCGAAGGATAGTGGGATTGTGCG

TCATCCCTTACGTCAGTGGAGATATCACATCAATCCACTTGCTTTGAAGACGTGGTTGGAACGTCTTCTT

TTTCCACGATGCTCCTCGTGGGTGGGGGTCCATCTTTGGGACCACTGTCGGCAGAGGCATCTTCAACGAT

GGCCTTTCCTTTATCGCAATGATGGCATTTGTAGGAGCCACCTTCCTTTTCCACTATCTTCACAATAAAG

TGACAGATAGCTGGGCAATGGAATCCGAGGAGGTTTCCGGATATTACCCTTTGTTGAAAAGTCTCAATGA

ATTCCTATTTTCAGAAGAAGTTCCCAATAGTAGTCCAAAATTTTTGTAACGAAGGGAGCATAATAGTTAC

ATGCAAAGGAAAACTGCCATTCTTTAGAGGGGATGCTTGTTTAAGAACAAAAAATATATCACTTTCTTTT

GTTCCAAGTCATTGCGTATTTTTTTAAAAATATTTGTTCCTTCGTATATTTCGAGCTTCAATCACTTTAT

GGTTCTTTGTATTCTGGCTTTGCTGTAAATCGTAGCTAACCTTCTTCCTAGCAGAAATTATTAATACTTG

GGATATTTTTTTAGAATCAAGTAAATTACATATTACCACCACATCGAGCTGCTTTTAAATTCATATTACA

GCCATATAGGCTTGATTCATTTTGCAAAATTTCCAGGATATTGACAACGTTAACTTAATAATATCTTGAA

ATATTAAAGCTATTATGATTAGGGGTGCAAATGGACCGAGTTGGTTCGGTTTATATCAAAATCAAACCAA

ACCAACTATATCGGTTTGGATTGGTTCGGTTTTGCCGGGTTTTCAGCATTTTCTGGTTTTTTTTTTGTTA

GATGAATATTATTTTAATCTTACTTTGTCAAATTTTTGATAAGTAAATATATGTGTTAGTAAAAATTAAT

TTTTTTTACAAACATATGATCTATTAAAATATTCTTATAGGAGAATTTTCTTAATAACACATGATATTTA

TTTATTTTAGTCGTTTGACTAATTTTTCGTTGATGTACACTTTCAAAGTTAACCAAATTTAGTAATTAAG

TATAAAAATCAATATGATACCTAAATAATGATATGTTCTATTTAATTTTAAATTATCGAAATTTCACTTC

AAATTCGAAAAAGATATATAAGAATTTTGATAGATTTTGACATATGAATATGGAAGAACAAAGAGATTGA

CGCATTTTAGTAACACTTGATAAGAAAGTGATCGTACAACCAATTATTTAAAGTTAATAAAAATGGAGCA

CTTCATATTTAACGAAATATTACATGCCAGAAGAGTCGCAAATATTTCTAGATATTTTTTAAAGAAAATT

CTATAAAAAGTCTTAAAGGCATATATATAAAAACTATATATTTATATTTTGGTTTGGTTCGAATTTGTTT

TACTCAATACAAAACTAAATTAGACCAAATATAATTGGGATTTTTAATCGACCCGGGAGGCCTACTAGTC

GACCGCGGCGATCTAGTAACATAGATGACACCGCGCGCGATAATTTATCCTAGTTTGCGCGCTATATTTT

GTTTTCTATCGCGTATTAAATGTATAATTGCGGGACTCTAATCATAAAAACCCATCTCATAAATAACGTC

ATGCATTACATGTTAATTATTACATGCTTAACGTAATTCAACAGAAATTATATGATAATCATCGCAAGAC

CGGCAACAGGATTCAATCTTAAGAAACTTTATTGCCAAATGTTTGAACGATCGGGGAAATTCGAGCTCTC

ATTGTTTGCCTCCCTGCTGCGGTTTTTCACCGAAGTTCATGCCAGTCCAGCGTTTTTGCAGCAGAAAAGC

CGCCGACTTCGGTTTGCGGTCGCGAGTGAAGATCCCTTTCTTGTTACCGCCAACGCGCAATATGCCTTGC

GAGGTCGCAAAATCGGCGAAATTCCATACCTGTTCACCGACGACGGCGCTGACGCGATCAAAGACGCGGT

GATACATATCCAGCCATGCACACTGATACTCTTCACTCCACATGTCGGTGTACATTGAGTGCAGCCCGGC

TAACGTATCCACGCCGTATTCGGTGATGATAATCGGCTGATGCAGTTTCTCCTGCCAGGCCAGAAGTTCT

TTTTCCAGTACCTTCTCTGCCGTTTCCAAATCGCCGCTTTGGACATACCATCCGTAATAACGGTTCAGGC

ACAGCACATCAAAGAGATCGCTGATGGTATCGGTGTGAGCGTCGCAGAACATTACATTGACGCAGGTGAT

CGGACGCGTCGGGTCGAGTTTACGCGTTGCTTCCGCCAGTGGCGCGAAATATTCCCGTGCACCTTGCGGA

CGGGTATCCGGTTCGTTGGCAATACTCCACATCACCACGCTTGGGTGGTTTTTGTCACGCGCTATCAGCT

CTTTAATCGCCTGTAAGTGCGCTTGCTGAGTTTCCCCGTTGACTGCCTCTTCGCTGTACAGTTCTTTCGG

CTTGTTGCCCGCTTCGAAACCAATGCCTAAAGAGAGGTTAAAGCCGACAGCAGCAGTTTCATCAATCACC

ACGATGCCATGTTCATCTGCCCAGTCGAGCATCTCTTCAGCGTAAGGGTAATGCGAGGTACGGTAGGAGT

TGGCCCCAATCCAGTCCATTAATGCGTGGTCGTGCACCATCAGCACGTTATCGAATCCTTTGCCACGCAA

GTCCGCATCTTCATGACGACCAAAGCCAGTAAAGTAGAACGGTTTGTGGTTAATCAGGAACTGTTCGCCC

TTCACTGCCACTGACCGGATGCCGACGCGAAGCGGGTAGATATCACACTCTGTCTGGCTTTTGGCTGTGA

CGCACAGTTCATAGAGATAACCTTCACCCGGTTGCCAGAGGTGCGGATTCACCACTTGCAAAGTCCCGCT

AGTGCCTTGTCCAGTTGCAACCACCTGTTGATCCGCATCACGCAGTTCAACGCTGACATCACCATTGGCC

ACCACCTGCCAGTCAACAGACGCGTGGTTACAGTCTTGCGCGACATGCGTCACCACGGTGATATCGTCCA

CCCAGGTGTTCGGCGTGGTGTAGAGCATTACGCTGCGATGGATTCCGGCATAGTTAAAGAAATCATGGAA

GTAAGACTGCTTTTTCTTGCCGTTTTCGTCGGTAATCACCATTCCCGGCGGGATAGTCTGCCAGTTCAGT

TCGTTGTTCACACAAACGGTGATACGTACACTTTTCCCGGCAATAACATACGGCGTGACATCGGCTTCAA

ATGGCGTATAGCCGCCCTGATGCTCCATCACTTCCTGATTATTGACCCACACTTTGCCGTAATGAGTGAC

CGCATCGAAACGCAGCACGATACGCTGGCCTGCCCAACCTTTCGGTATAAAGACTTCGCGCTGATACCAG

ACGTTGCCCGCATAATTACGAATATCTGCATCGGCGAACTGATCGTTAAAACTGCCTGGCACAGCAATTG

CCCGGCTTTCTTGTAACGCGCTTTCCCACCAACGCTGATCAATTCCACAGTTTTCGCGATCCAGACTGAA

TGCCCACAGGCCGTCGAGTTTTTTGATTTCACGGGTTGGGGTTTCTACAGGACGTAACATGGATCCTGTA

ATTGTAAATAGTAATTGTAATGTTGTTTGTTGTTTGTTGTTGTTGGTAATTGTTGTAAAAATACGTGTCC

TCTCCAAATGAAATGAACTTCCTTATATAGAGGAAGGGTCTTGCGAAGGATAGTGGGATTGTGCGTCATC

CCTTACGTCAGTGGAGATATCACATCAATCCACTTGCTTTGAAGACGTGGTTGGAACGTCTTCTTTTTCC

ACGATGCTCCTCGTGGGTGGGGGTCCATCTTTGGGACCACTGTCGGCAGAGGCATCTTCAACGATGGCCT

TTCCTTTATCGCAATGATGGCATTTGTAGGAGCCACCTTCCTTTTCCACTATCTTCACAATAAAGTGACA

GATAGCTGGGCAATGGAATCCGAGGAGGTTTCCGGATATTACCCTTTGTTGAAAAGTCTCAATGAATTAA

TTCGGGTCTTGCGAAGGATAGTGGGATTGTGCGTCATCCCTTACGTCAGTGGAGATATCACATCAATCCA

CTTGCTTTGAAGACGTGGTTGGAACGTCTTCTTTTTCCACGATGCTCCTCGTGGGTGGGGGTCCATCTTT

GGGACCACTGTCGGCAGAGGCATCTTCAACGATGGCCTTTCCTTTATCGCAATGATGGCATTTGTAGGAG

CCACCTTCCTTTTCCACTATCTTCACAATAAAGTGACAGATAGCTGGGCAATGGAATCCGAGGAGGTTTC

CGGATATTACCCTTTGTTGAAAAGTCTCAATAAGCTTCTGCAGCATGCACTATTTTCAGAAGAAGTTCCC

AATAGTAGTCCAAAATTTTTGTAACGAAGGGAGCATAATAGTTACATGCAAAGGAAAACTGCCATTCTTT

AGAGGGGATGCTTGTTTAAGAACAAAAAATATATCACTTTCTTTTGTTCCAAGTCATTGCGTATTTTTTT

AAAAATATTTGTTCCTTCGTATATTTCGAGCTTCAATCACTTTATGGTTCTTTGTATTCTGGCTTTGCTG

TAAATCGTAGCTAACCTTCTTCCTAGCAGAAATTATTAATACTTGGGATATTTTTTTAGAATCAAGTAAA

TTACATATTACCACCACATCGAGCTGCTTTTAAATTCATATTACAGCCATATAGGCTTGATTCATTTTGC

AAAATTTCCAGGATATTGACAACGTTAACTTAATAATATCTTGAAATATTAAAGCTATTATGATTAGGGG

TGCAAATGGACCGAGTTGGTTCGGTTTATATCAAAATCAAACCAAACCAACTATATCGGTTTGGATTGGT

TCGGTTTTGCCGGGTTTTCAGCATTTTCTGGTTTTTTTTTTGTTAGATGAATATTATTTTAATCTTACTT

TGTCAAATTTTTGATAAGTAAATATATGTGTTAGTAAAAATTAATTTTTTTTACAAACATATGATCTATT

AAAATATTCTTATAGGAGAATTTTCTTAATAACACATGATATTTATTTATTTTAGTCGTTTGACTAATTT

TTCGTTGATGTACACTTTCAAAGTTAACCAAATTTAGTAATTAAGTATAAAAATCAATATGATACCTAAA

TAATGATATGTTCTATTTAATTTTAAATTATCGAAATTTCACTTCAAATTCGAAAAAGATATATAAGAAT

TTTGATAGATTTTGACATATGAATATGGAAGAACAAAGAGATTGACGCATTTTAGTAACACTTGATAAGA

AAGTGATCGTACAACCAATTATTTAAAGTTAATAAAAATGGAGCACTTCATATTTAACGAAATATTACAT

GCCAGAAGAGTCGCAAATATTTCTAGATATTTTTTAAAGAAAATTCTATAAAAAGTCTTAAAGGCATATA

TATAAAAACTATATATTTATATTTTGGTTTGGTTCGAATTTGTTTTACTCAATACAAAACTAAATTAGAC

CAAATATAATTGGGATTTTTAATCGAGGTACCTAATGTAATGTGAAATTGCTATCCAAAAGGCACCTAAT

TTTGTCCACCGTTCAAAGGAAAGGACAAGGAAGTAGTAGCGTGTAGGTTTGGTGCTGTACAAAATAAGCA

AGACACGTGTTGCCTTATTATAGGATAATCCATAAGGCAATTTCGTCTTAAGTCGGCCATTGCACCTTTA

AAAGGAGCCTCTTTGTTCCCAAAATCTTCATCCTTTGATTTCTCTATTCTCAATATCTCCTCAATTTTTC

TCTAGTCTTCAAACACTTCTCAAGGTACATTAACTTCTTCTTTCTTTTTGTTCCTCTTATTTTATGCTAC
[truncated: 1,879,546 more chars]
